# Alcohols as Efficient Intermolecular Initiators for a Highly Stereoselective Polyene Cyclisation Cascade

**DOI:** 10.1002/chem.202203732

**Published:** 2023-01-30

**Authors:** Daniya Aynetdinova, Reece Jacques, Kirsten E. Christensen, Timothy J. Donohoe

**Affiliations:** ^1^ Department of Chemistry University of Oxford Chemistry Research Laboratory Oxford OX1 3TA UK; ^2^ Early Chemical Development, Medicinal Chemistry R&D Vertex Pharmaceuticals Abington OX14 4RW UK

**Keywords:** Alcohols, carbocations, HFIP, Lewis acids, polyene cyclisation, steroids

## Abstract

The use of benzylic and allylic alcohols in HFIP solvent together with Ti(O^i^Pr)_4_ has been shown to trigger a highly stereoselective polyene cyclisation cascade. Three new carbon‐carbon bonds are made during the process and complete stereocontrol of up to five new stereogenic centers is observed. The reaction is efficient, has high functional group tolerance and is atom‐economic generating water as a stoichiometric by‐product. A new polyene substrate‐class is employed, and subsequent mechanistic studies indicate a stereoconvergent mechanism. The products of this reaction can be used to synthesize steroid‐analogues in a single step.

## Graphical abstract

**A stereoselective polyene cyclisation cascade** was enabled by using HFIP as a solvent. The process is triggered by a cation generated from an alcohol in situ and leads to the formation of three new C‐C bonds and up to five new stereocenters as a single diastereomer. The cyclised products can be derivatised to obtain steroidal analogues in a single step.

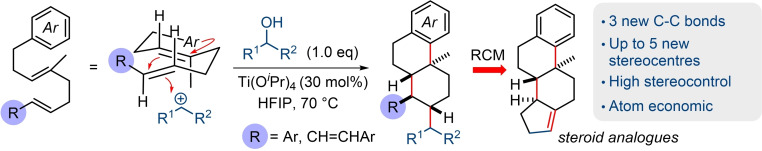

## Introduction

**Scheme 1:**
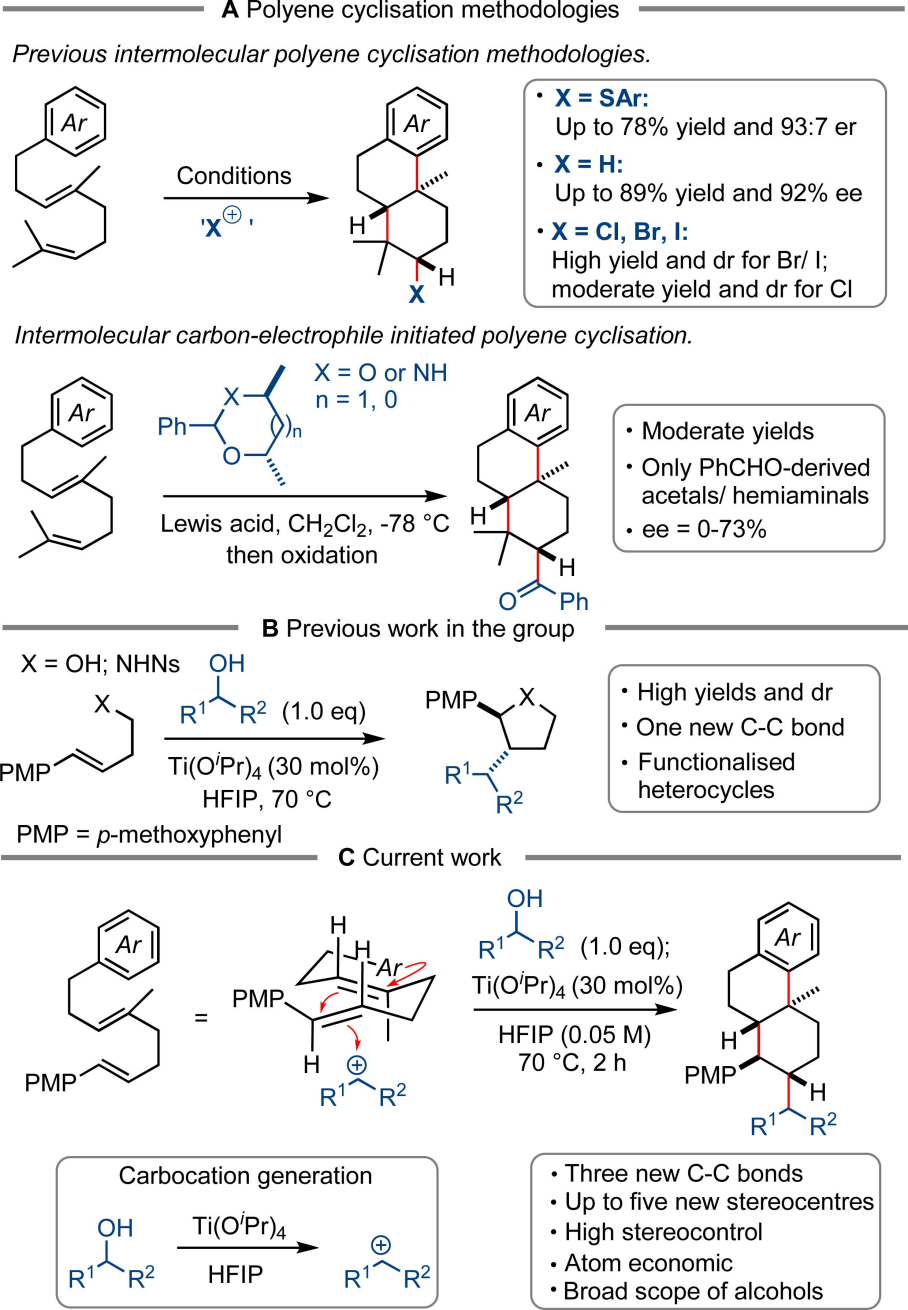
(**A**) Previous polyene cyclisation methodologies; (**B**) Previous work in the group; (**C**) Current work.

The exploration of diverse chemical space is highly important in modern synthetic and pharmaceutical chemistry.^[1]^ One of the most challenging aspects of such an exploration lies in the rapid and stereoselective construction of complex three‐dimensional molecules.^[2]^ Methodologies which are able to achieve this are of great value owing to the high number of biologically active natural products and pharmaceuticals bearing an sp^3^‐rich carbon skeleton, such as steroids and polycyclic terpenoids.[^3a^, ^3b^, ^3c^] In nature, steroids and terpene‐derived molecules are made via a polyene cyclisation cascade, catalyzed by enzymes which ensure the selective formation of a single stereoisomer.^[4]^ The ability to construct multiple stereocenters in such a manner has an obvious synthetic utility and so polyene cyclisation has received a lot of attention from the scientific community. Various electrophilic reagents have been used to initiate cyclisation, including halonium reagents,[^5a^, ^5b^, ^5c^, ^5d^, ^5e^, ^5f^, ^5g^, ^5h^, ^5i^, ^5j^] Lewis and Brønsted acids[^6a^, ^6b^, ^6c^, ^6d^, ^6e^, ^6f^, ^6g^, ^6h^, ^6i^, ^6j^, ^6k^, ^6l^, ^6m^, ^6n^] and sulfenium ions (Scheme 1A).[^7a^, ^7b^, ^7c^] However, the use of carbon electrophiles to trigger cyclisation cascades and form a new exocyclic carbon‐carbon (C−C) bond has been largely unexplored. To the best of our knowledge, only acetals have been used as intermolecular carbon‐based electrophilic initiators in polyene cyclisation, as pioneered by Loh.[^8a^, ^8b^, ^8c^] Using benzaldehyde‐derived acetals and *N*‐acetals Loh and co‐workers obtained polycyclic products in moderate to high yields with up to 93 : 7 diastereomeric ratio (*dr*) at the new exocyclic benzylic stereocenter; an asymmetric version using chiral *N*‐acetals gave products with up to 73 % ee. While this method allowed stereoselective cyclisation using benzaldehyde‐derived acetals and *N*‐acetals, it does not demonstrate the use of any other type of initiator for cyclisation. Moreover, in some cases the efficiency of the polyene cyclisation was reduced by undesired monocyclisation and moderate diastereomeric ratios.

In this regard, we wanted to investigate the use of readily available alcohols as a new class of intermolecular initiator in polyene cyclisation cascades. Our inspiration came from previous work in the group in which the stereoselective synthesis of tetrahydrofurans^[9a]^ and pyrrolidines^[9b]^ was achieved using carbocations generated in situ by ionization of alcohols^[10]^ in HFIP solvent (Scheme 1B).^[11]^ Here, we proposed that HFIP could enable alcohols to form a cation and engage with the most electron‐rich alkene of a polyene substrate, thus initiating a stereoselective cascade and merge the two molecules, forming an exocyclic C−C bond (Scheme 1C). Herein, we report the realisation of an intermolecular carbon‐electrophile initiated polyene cyclisation cascade with the formation of three new C−C bonds and excellent stereocontrol of up to five stereogenic centers. This process also has high atom economy with water being generated as a by‐product. For the first time a broad range of intermolecular carbon‐based initiators is reported. Moreover, this methodology uses a different substrate class from the conventional geraniol‐derived polyenes which also enables the rapid construction of steroid‐like cores thus demonstrating the utility and applicability of the transformation.

## Results and Discussion

We began our studies by optimizing the reaction using 4‐methoxybenzyl alcohol and diene **1** as a model system (Table 1). As a starting point the use of sub‐stoichiometric Ti(O^*i*^Pr)_4_ in HFIP^[9a]^ resulted in product **2 a** being formed as a single diastereomer in 48 % isolated yield (Table 1, Entry 1). The remaining mass balance comprised of a complex mixture of compounds that could not be identified. Lowering the concentration to 0.05 M improved the yield to 65 % (Entry 2), while further decrease of the concentration diminished the yield (Entry 3). The same effect was observed when the temperature was lowered to 40 °C (Entry 4). Since diene **1** has only limited stability under the reaction conditions (see below), the negative effect of lowering the reaction temperature or diluting the mixture can be explained by the reduced reaction rate versus substrate decomposition. Increasing the amount of Ti(O^*i*^Pr)_4_ did not improve the yield above 65 % (Entry 5). Moreover, replacing Ti(O^*i*^Pr)_4_ with PPh_4_PBF_4_, a salt which was reported to improve the cation stabilizing ability of HFIP,^[12]^ furnished a complex reaction mixture with only 38 % of product **2 a** being isolated (Entry 6). Interestingly, removal of Ti(O^*i*^Pr)_4_ did not shut down the reaction and was only slightly detrimental to the reaction yield (Entry 7). In addition, we performed a full screen of alternative solvents with Ti(O^*i*^Pr)_4_ additive (eg ^*i*^PrOH, MeOH, DMF, MeCN, DCE, toluene, see Supporting Information) but none of them gave any product **2 a** at all. Given the marginally beneficial role of Ti(O^*i*^Pr)_4_ we also examined other Lewis acids in HFIP (eg Zn(OAc)_2_, Zn(OTf)_2_, AlCl_3_) and also Brønsted acids, but again no improvement in yield with respect to the optimal conditions shown in Table 1, Entry 2 was found. These results highlight the unique properties of HFIP in facilitating polyene cyclisations,[^5b^, ^7a^, ^12^, ^13^] which is here initiated by an S_N_1 reaction of 4‐methoxybenzyl alcohol, see below.

**Table 1:** Reaction optimisation.

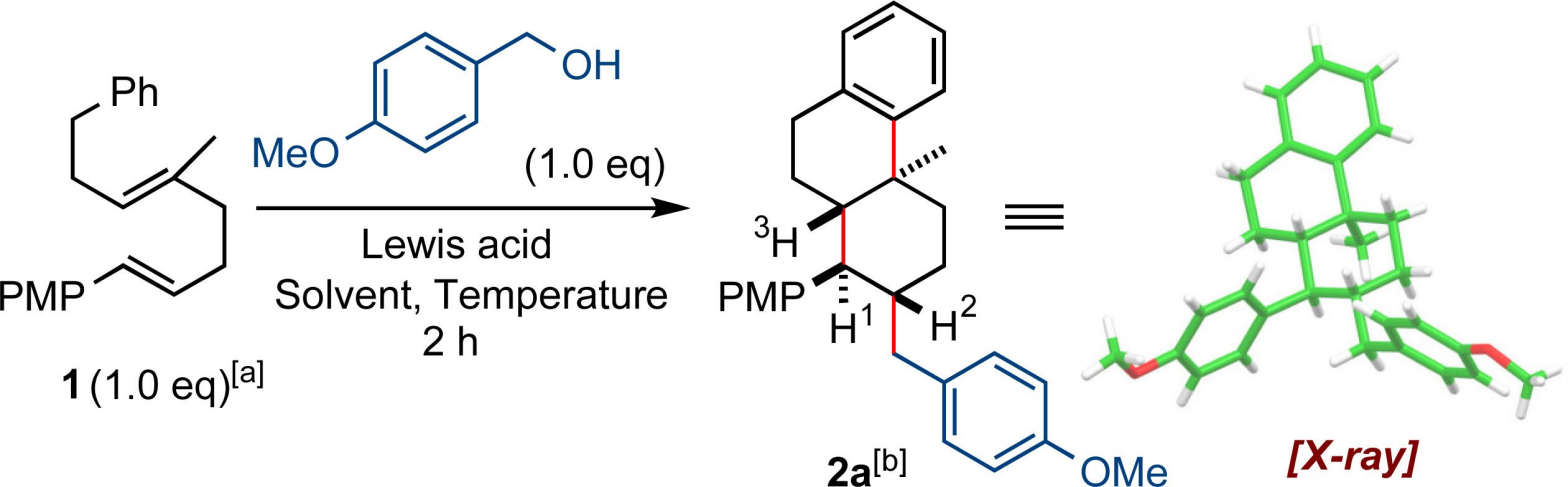						
	Reaction solvent	Conc [M]	T [°C]	Lewis acid	Loading [mol %]	Yield of **2 a**[%]^[c]^
1	HFIP	0.1	70	Ti(O^*i*^Pr)_4_	30	48
**2**	**HFIP**	**0.05**	**70**	**Ti(O** ^***i***^ **Pr)_4_**	**30**	**65**
3	HFIP	0.02	70	Ti(O^*i*^Pr)_4_	30	40
4	HFIP	0.05	40	Ti(O^*i*^Pr)_4_	30	37
5	HFIP	0.05	70	Ti(O^*i*^Pr)_4_	50	52
6	HFIP	0.05	70	PPh_4_PBF_4_	30	38
7	HFIP	0.05	70	–	0	49

[a] All reactions were performed on 25 mg scale. [b] Relative stereochemistry for **2 a** confirmed by NOESY, ^3^ *J* coupling constant between H‐1, H‐2 and H‐3 and single crystal X‐ray diffraction. [c] Isolated yields.

**Scheme 2:**
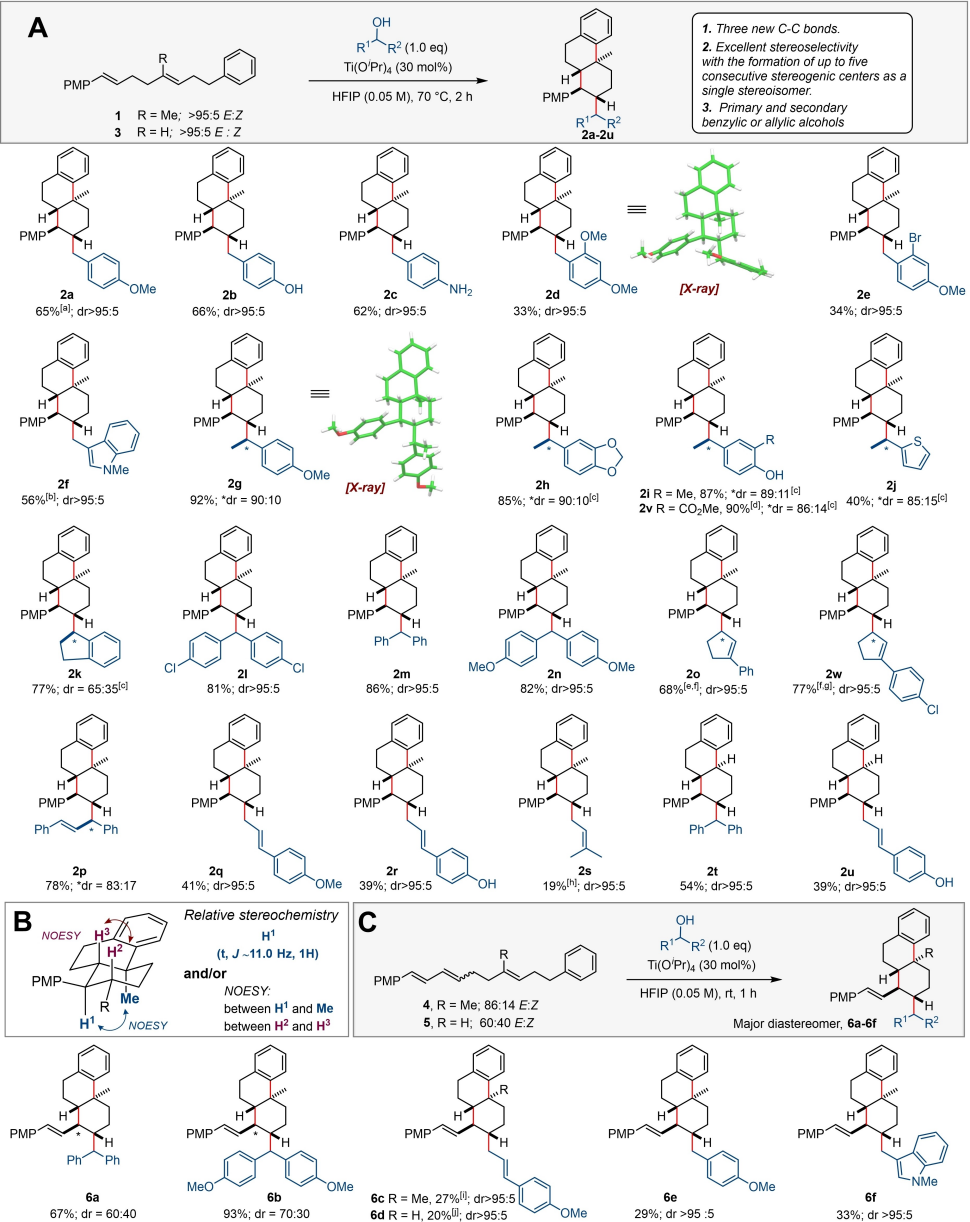
(**A**) Substrate scope using dienes **1** and **3**; (**B**) Evidence for the relative stereochemistry; (**C**) Substrate scope using trienes **4** and **5**; Reactions performed on 0.10 mmol scale unless otherwise stated; *dr* determined by ^1^H NMR spectroscopy of the crude reaction mixture unless otherwise stated; [a] Reaction performed on 0.082 mmol scale; [b] Reaction conducted at rt for 2.5 h; [c] *dr* determined by ^1^H NMR spectroscopy after column chromatography; [d] Reaction performed overnight; [e] Reaction performed at rt for 4 h; [f] The exocyclic stereochemistry of **2 o** and **2 w** could not be determined unambiguously; [g] Reaction performed at rt for 1.5 h. Reaction of the transposed allylic alcohol gave the same product **2 w** in 44 % yield, see Supporting Information; [h] Reaction performed with 2.0 equiv. of alcohol; [i] Reaction performed overnight without Ti(O^*i*^Pr)_4_; [j] Reaction performed with 10 mol% of Ti(O^*i*^Pr)_4_.

With the optimized conditions in hand, we set out to evaluate the scope of this transformation using diene **1** (Scheme 2A). A diverse number of electron‐rich primary and secondary alcohols furnished products **2 a**–**2 w** in up to 92 % yield and with complete stereocontol of up to five newly formed stereocenters. When non‐symmetrical secondary alcohols were used, good to excellent stereocontol was achieved at the exocyclic centre. Notably, products **2 o** and **2 w** were formed in 68 % and 77 % yields respectively with five newly formed contiguous stereocenters, isolated as single diastereomers. Different functional groups on the initiator, such as free hydroxyl (**2 b**, **2 r**, **2 v** and **2 i**), amine (**2 c**), ester (**2 v**), acetal (**2 h**), halogens (**2 e**, **2 l** and **2w**), alkenes (**2 o**–**2 s**), indole (**2 f**), and thiophene (**2 j**) were well tolerated under the reaction conditions. We noted that primary alcohols were generally less effective than secondary alcohols in this methodology, resulting in more complex reaction mixtures with variable amounts of polyalkylated side‐products being observed. This observation can be explained by the comparably lower stability of primary carbocations, and hence higher reactivity compared to their secondary counterparts. Nevertheless, products **2 a**–**2 f** were obtained in useful synthetic yields with excellent diastereoselectivity. Secondary benzylic alcohols also worked well in this methodology; both cyclic and acyclic alcohols could be engaged in the cyclisation cascade furnishing products **2 g**–**2 n** in excellent yields. The formation of **2 v** in 90 % yield also displays some tolerance for electron withdrawing groups on the alcohol. Higher diastereoselectivity at the additional exocyclic centre was observed for non‐symmetrical alcohols furnishing more stabilised carbocations: from 65 : 35 (**2 k**) with 1‐indanol to 90 : 10 (**2 g**) with 1‐(4‐methoxyphenyl)ethan‐1‐ol. For benzhydrol type alcohols electron‐rich, neutral, and electron poor alcohols were highly successful in promoting the polyene cyclisation and afforded products **2 n**, **2 m**, and **2 l** in 82 %, 86 % and, 81 % yield respectively. Allylic alcohols could also be engaged in the cyclisation cascade furnishing products **2 o**–**2 s** and **2 w** with an external C−C double bond which can be used as a synthetic handle for further derivatisation. Secondary cyclic and acyclic allylic alcohols gave products **2 o**, **2 p** and, **2 w** in good yields and with high diastereoselectivity at the exocyclic centre, while primary allylic alcohols afforded products **2 q**–**2 s** as single diastereomers in moderate yields.

Experiments which utilised **1** together with primary, secondary, or tertiary alcohols lacking an adjacent activating alkene or arene unit were unsuccessful and did not lead to the formation of products with a new exocyclic C−C bond, see Supporting Information.

Finally, we were pleased to find that the developed alkylative polyene cyclisation methodology does not require a trisubstituted internal alkene and works well for a much less described^[14]^ disubstituted alkene as well; products **2 t** and **2 u** were formed as single diastereomers in 54 % and 39 % yield respectively using diene **3**.

The relative configuration of the substituents on the ring structure for products **2 a**–**2 w** was determined by NOESY analysis, together with a ^3^ *J* coupling constant of approximately 11 Hz between protons H‐1, H‐2 and H‐3, and was confirmed by a number of single crystal X‐ray diffraction studies.^[15]^ Note that the PMP group and the new exocyclic substituent occupy equatorial positions whereas the protons H‐1, H‐2 and H‐3 are axial. This relative configuration is consistent with a chair‐like transition state during cyclisation of the *E*, *E*‐isomer **1** together with an equatorial intermolecular attack of the carbocation (see Scheme 4). The configuration at the exocyclic carbon centre for products **2 h**–**2 k**, **2 p**, and **2 v** was assigned by analogy to product **2 g**, for which a single crystal X‐ray diffraction structure was obtained.^[15]^

After showing that a broad variety of allylic and benzyl alcohols can act as competent electrophilic partners for the alkylative polyene cyclisation cascade of alkenes **1** and **3**, we decided to explore the 6‐*endo*‐trig cyclisation of a triene substrate **4** with an extended PMP motif to set up an exocyclic double bond which could be used for further derivatisation, including construction of an additional ring structure present in multiple steroid and gonane molecules, see below (Scheme 2C).^[16]^ For this purpose alkene **4** was synthesised in four steps (see Supporting Information) in an 86 : 14 *E : Z* ratio and then subjected to the cyclisation conditions. We were pleased to find that benzylic and cinnamyl alcohols could be engaged in the alkylative cyclisation cascade, although the yields of products **6 a**–**6 f** were lower compared to alkene **1**. Secondary alcohols furnished products **6 a** and **6 b** in good yields, however moderate diastereoselectivity was observed at the allylic position. Products **6 c**–**6 f** were isolated as single diastereomers albeit in lower yields compared to cyclisation products with secondary alcohols. Moreover, triene **5** containing a disubstituted internal alkene could also be engaged in the reaction with (*E*)‐3‐(4‐methoxyphenyl)prop‐2‐en‐1‐ol, although product **6 d** was isolated in 20 % yield. This alkylative cyclisation enabled by an electron rich diene motif is an exciting development in the polyene cyclisation area because the products can be derivatized at the exocyclic position: this is often inaccessible during more conventional cyclisation reactions which typically install a gem‐dimethyl group at this position.

**Scheme 3:**
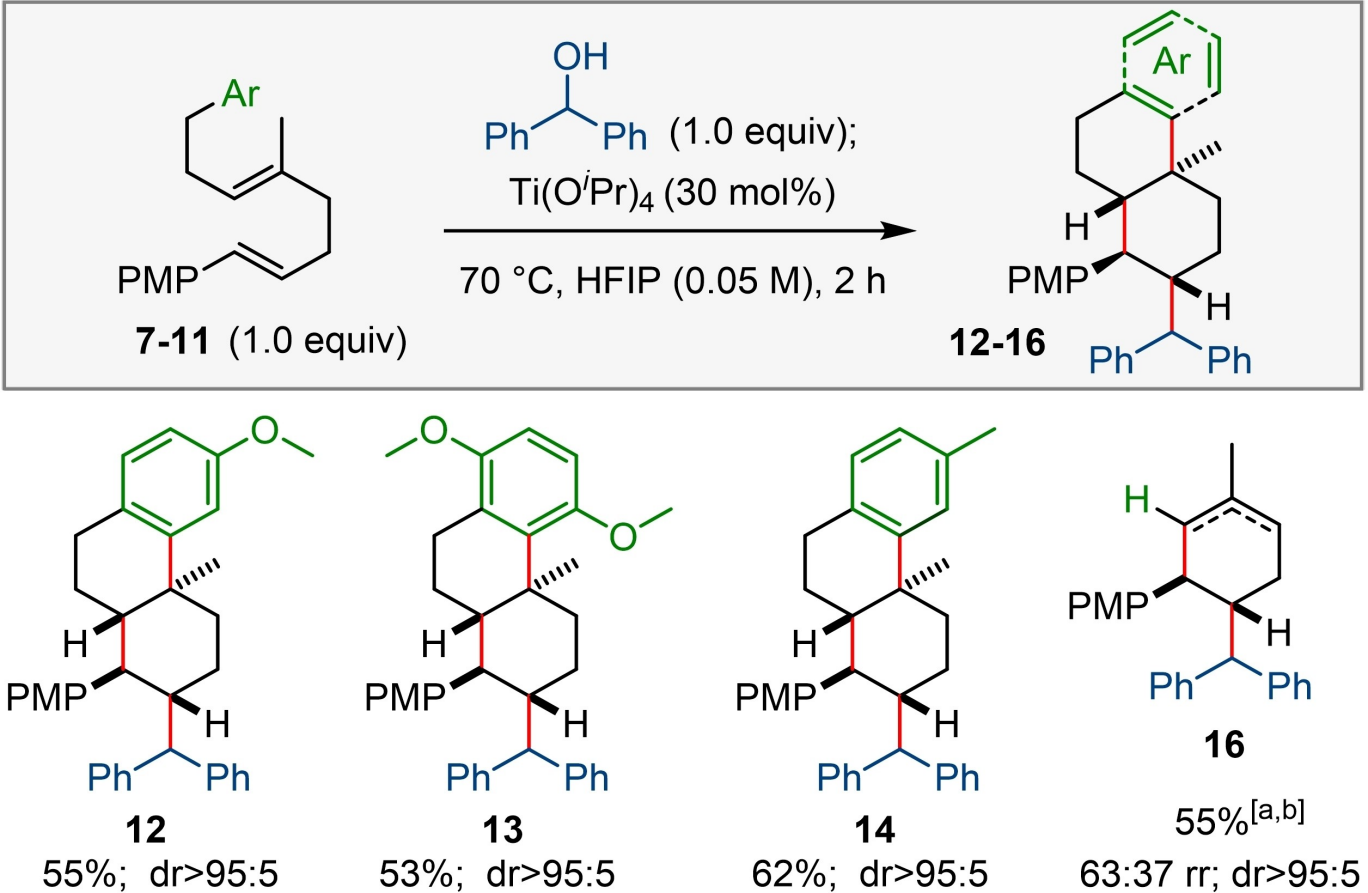
The scope of aryl terminating groups; [a] rr determined after column chromatography; [b] Reaction conditions: 0.02 M, overnight. See Supporting Information for X‐ray crystal structures of compounds **12** and **14** which confirms their structures.

Next, we explored the versatility of the alkylative polyene cyclisation by varying the aromatic terminating groups (Scheme 3). Pleasingly, the cyclisation worked well for substrates, encompassing electron‐rich arenes; products **12**–**14** were obtained as single diastereomers in good yields. When a *para*‐chloro substituted arene was used, a complex mixture of diastereomers **15** was isolated (see Supporting Information). This result can be explained by the reduced propensity of an electron deficient arene to participate in cyclisation, resulting in the formation of an intermediate carbocation which can undergo non‐selective cyclisation. Finally, if no aromatic terminating nucleophile is present at all, alkylative cyclisation proceeds with excellent diastereoselectivity to form cyclohexene **16** in 55 % as a mixture of alkene regioisomers (63 : 37 rr) presumably as a result of an unselective E_1_ elimination termination event.

Further experiments were then designed to shed light on the mechanism of this new alkylative polyene cyclisation. The first step of the cascade is ionisation of the alcohol to form a stabilized cation. This is supported by the observations that electron deficient benzyl alcohols (eg 4‐cyanobenzyl alcohol) are unreactive in the reaction and also because transposed isomers of unsymmetrical allylic alcohols give the same reaction products (see **2 w**). Table 1 shows that a Lewis acid additive helps the reaction but is not essential. This is consistent with a Lewis acid and/or solvent (HFIP) assisted loss of water from an alcohol that gives rise to a carbocation which can then initiate cyclisation.^[17]^ It has also been suggested in the literature that HFIP may aid polyene cyclisations by virtue of a hydrophobic effect favouring reactive conformations of the substrate.[^5b^, ^7a^, ^12^] While this may be another role for the solvent, we were unable to obtain reliable data on the conformation of **1** in deuterated HFIP because of substrate decomposition.

**Scheme 4:**
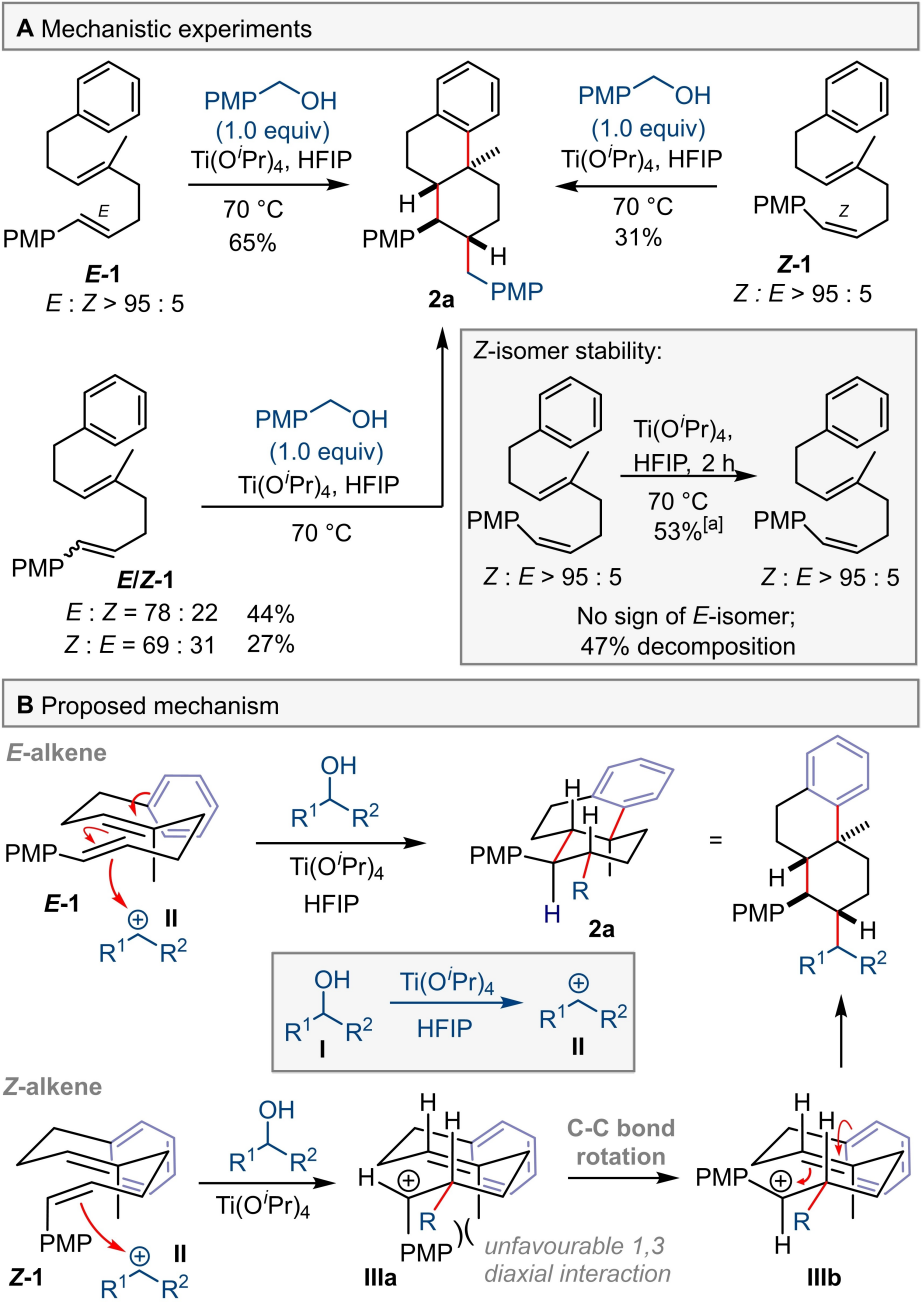
Mechanistic experiments. [a] NMR yield using 1,1,2,2‐tetrachloroethane as an internal standard.

We were also interested in the mechanism of the cyclisation itself, and the consequences of using stereoisomers of **1** were explored (Scheme 4A). When the *Z*‐isomer of diene **1** was treated under the standard reaction conditions the tricycle **2 a** produced was identical to that obtained from the reaction of *E*‐**1**; for both reactions, only a single isomer was observed. Interestingly, the product **2 a** has retained the *trans* configuration of *E*‐**1** when either *Z*‐**1** or mixtures of *E‐* **1** and *Z*‐**1** are used. The same outcome was observed when benzhydrol was used as a source of carbocation initiator (see Supporting Information). To probe this convergence of stereochemical information *Z*‐**1** was subjected to the reaction conditions, but in the absence of a carbocation initiator. After 2 h, the *Z*‐configuration of recovered starting material was unchanged, although significant decomposition had occured.

It is proposed that the stereoconvergent formation of product **2 a** may be the result of different cyclisation mechanisms for *Z*‐**1** and *E*‐**1** (Scheme 4B). In the case of *E*‐**1**, a concerted mechanism is possible in which three new C−C bonds are formed simultaneously via a chair‐like transition state (TS). For *Z*‐**1** we suggest a stepwise mechanism which leads to a carbocation **IIIa** that does not undergo cyclisation due to unfavourable 1,3‐diaxial interactions between the Me and PMP groups. Instead, C−C bond rotation occurs to bring the PMP group to an equatorial position **IIIb**, followed by cyclisation. Of course, it is also possible that *E*‐**1** reacts with the cation directly to form **IIIb** prior to cyclisation. The degree of concertedness in forming the second ring by means of aryl ring trapping is also of interest. We have depicted this as a concerted process tied to formation of the first six membered ring, although it is recognised that this might not be the case. Interestingly, when an electron deficient arene ring was used as a terminating group the reaction proceeded but with low stereoselectivity at the decalin ring junction, together with the formation of monocyclic products (see Supporting Information). This observation is supporting evidence for the mixtures that arise when the decalin ring system is formed via a non‐concerted process.

**Scheme 5:**
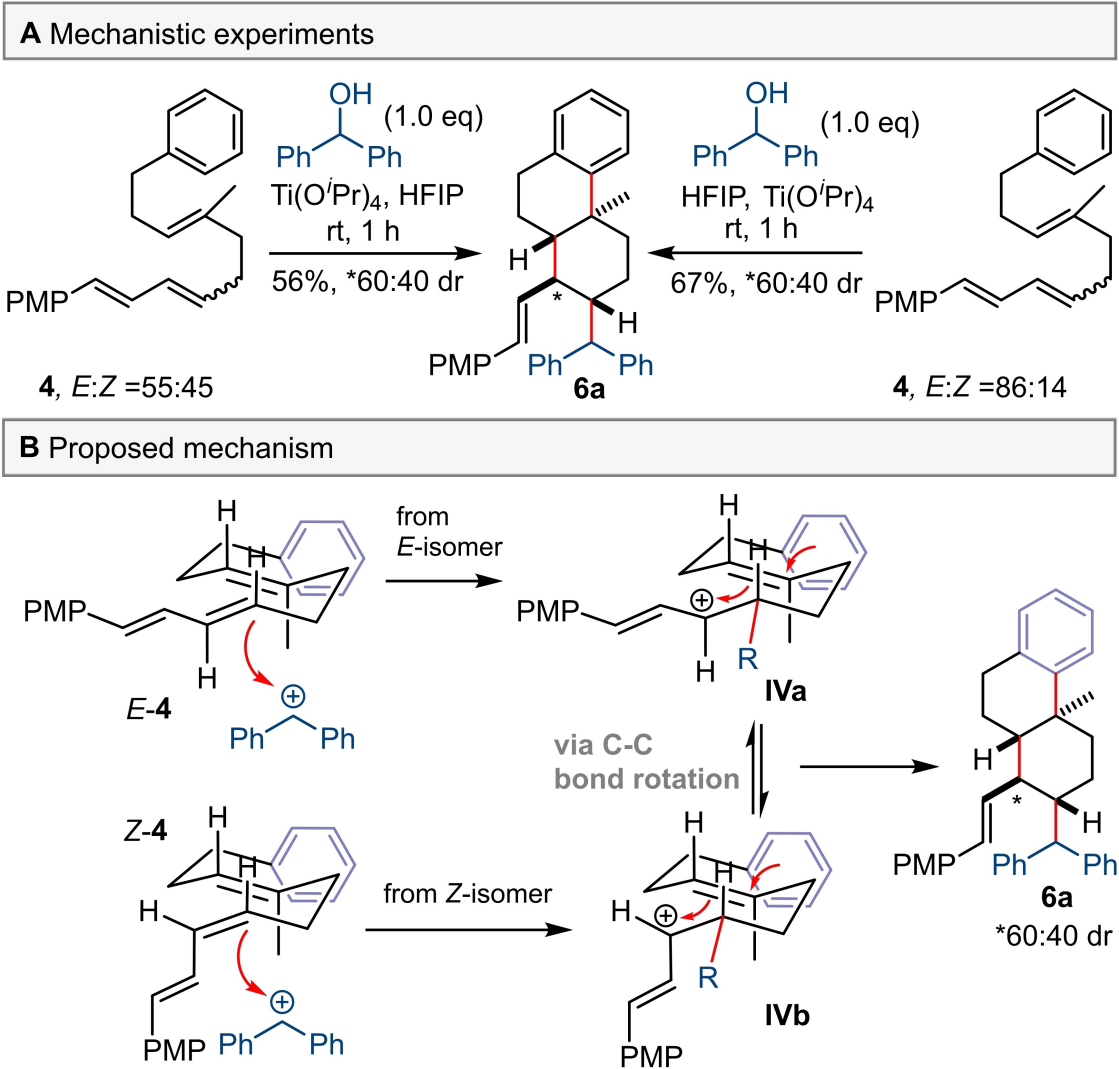
Mechanistic experiments and proposed mechanism. *Dr* was determined by ^1^H NMR spectroscopy of the crude reaction mixture.

Next, we studied the cyclisation of triene **4** using benzhydrol as an alkylating agent. Subjecting triene **4**, as a 86 : 14 *E : Z* mixture of alkene stereoisomers, to the reaction conditions furnished product **6 a** in 67 % yield with 60 : 40 *dr* at the allylic position (Scheme 5A). When a 55 : 45 *E : Z* mixture of triene **4** was used, product **6 a** was isolated in a slightly lower yield but with exactly the same *dr* at the allylic position. To check if alkene isomerisation prior to alkylation was responsible for the stereoconvergence, triene **4** in 86 : 14 *E : Z* ratio was subjected to the reaction conditions but without alcohol present. No erosion of *E : Z* ratio was observed, although only 69 % of triene **4** remained after 1 h.

These observations suggest a stepwise mechanism in which both *E‐* and *Z*‐isomers undergo alkylation with a benzhydrol derived carbocation to form highly stabilised carbocation **IV**. This carbocation could then undergo C−C bond rotation which scrambles any *E* or *Z* stereochemistry from the former alkene, prior to the ensuing cyclisation. Moreover, we suggest that 1,3‐(pseudo)diaxial strain between the axial Me and the vinyl‐PMP group will be smaller than that for the PMP group, allowing the cyclisation reaction of the *cis* configured intermediate (i.e. ***Z***‐**IV**) to proceed and rationalising the observed loss of stereoselectivity. When primary benzylic alcohols were used for the alkylative cyclisation of a triene **4**, the yields were lower and no minor diastereomers were isolated or observed (Scheme 2B). However, with the low yields we do not have confidence in the inherent selectivity of the reaction, because it is possible that any minor diastereomer may have decomposed in situ.

**Scheme 6:**
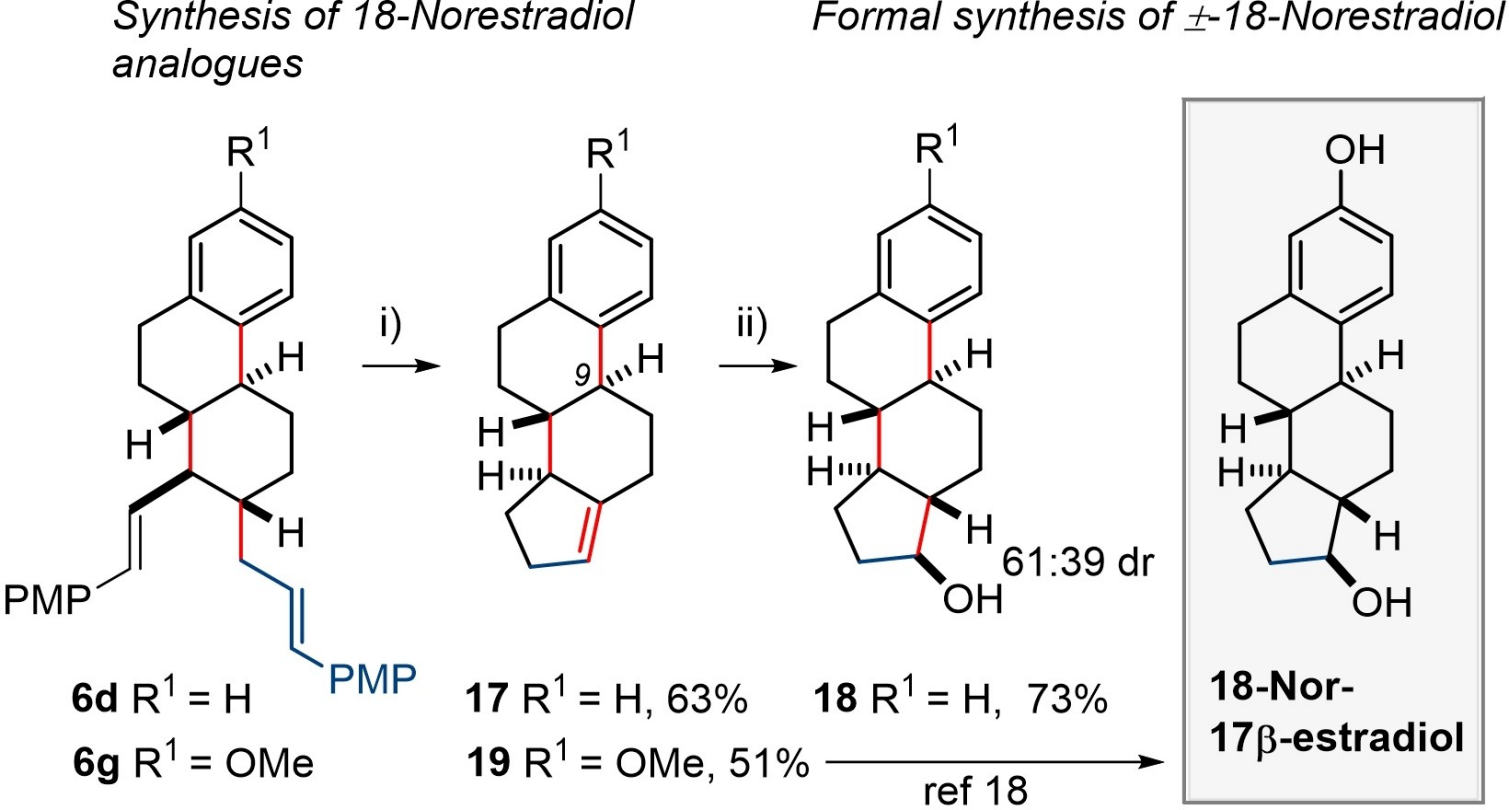
Derivatisation reactions. i) HG‐II (30 mol %), PhMe, 110 °C, overnight; ii) catecholborane (2.5 equiv.), LiBH_4_ (cat), C_6_H_6_, reflux, 24 h; then aq. NaOH (3 M), H_2_O_2_ (30 %), rt, 4 h.

Finally, we utilised this methodology to make a steroid ring system by employing a ring closing metathesis (RCM) reaction on the versatile vinyl PMP alkene activating group (see compounds **6 a**–**f** in Scheme 2). By employing **6 d** as a starting material, RCM enabled us to connect the alkene functionalities and, after alkene isomerization in situ, gave rise to formation of **17**, which contains the steroidal carbocyclic ring system, in 63 % yield, Scheme 6. Repetition of the RCM reaction on compound **6 g** (see Supporting Information) allowed for the formation of **19** (this compound has been previously prepared by a lengthy degradation of estradiol itself). Because compound **19** has been transformed into 18‐nor‐estradiol in two steps,^[18]^ our synthetic work constitutes a formal synthesis of this unnatural steroidal analogue whose congeners have been reported to possess interesting biological activity.[^19a^, ^19b^, ^19c^, ^19d^, ^19e^] In this regard, it is interesting to note the stereoselective hydroboration reaction^[18]^ of **17**, using conditions reported in the literature. In our hands, **17** gave the two *syn* addition products exclusively, in a 61 : 39 ratio, with the major product **18** possessing the desired *trans* 6,5‐ring junction. The success of this reaction sequence should facilitate the synthesis of other more diverse steroid analogues in the future. In this regard, compound **6 c** was also subjected to the RCM/isomerization sequence, giving rise to the formation of steroid analogues possessing a C‐9 methyl group (see Supporting Information).

## Conclusion

We have developed a new methodology using carbon electrophiles as intermolecular initiators for a highly stereoselective polyene cyclisation cascade. We have demonstrated that HFIP enables alcohols to trigger such a cascade process with the formation of three new C−C bonds and complete control of up to five new stereogenic centres is observed. A broad range of readily available alcohols was engaged in alkylative polyene cyclisation generating water as a by‐product. The products of this methodology could be derivatized in a single step to obtain the cores of steroid‐analogues.

## Experimental Section

**General experimental**: Microwave vials and vial caps (containing a resealing Silicone/PTFE septum) were purchased from Kinesis (Cole‐Palmer) and were used without flame‐drying. Unless otherwise stated, all other reactions were performed in flame‐dried glassware equipped with a stir bar using standard Schlenk techniques under an atmosphere of N_2_. All reagents and solvents were purchased and used as supplied from Sigma‐Aldrich (now Merck KGaA), Thermo Fisher Scientific (including Alfa Aesar and Acros Organics), Fluorochem, Honeywell, Tokyo Chemical Industry, Apollo Scientific, Manchester Organics (part of Navin Fluorine Int. Ltd.) and Strem Chemicals. Anhydrous DMSO and Et_3_N were purchased from Sigma‐Aldrich in Sure/SealTM bottles. Anhydrous Et_2_O, CH_2_Cl_2_, MeOH, THF and toluene were obtained from MBRAUN SPS‐5 solvent purification system by passage through double filtration columns under N_2_. 1,1,1,3,3,3‐Hexafluoro‐2‐propanol (HFIP) was purchased from Fluorochem. Ti(O*i*Pr)_4_ (≥97 %) was purchased from Sigma‐Aldrich. Thin layer chromatography (TLC) was performed on pre‐coated aluminium (200 μm) Merck Kieselgel 60 F254 plates, visualised using UV irradiation (*λ*=254 nm) and/or staining with potassium permanganate (KMnO4), vanillin, or phosphomolybdic acid (PMA) solutions. Purification by flash column chromatography was performed with Merck Kieselgel 60 (40–63 μm) or (15–40 μm) silica gel using head pressure by means of a nitrogen line. Systematic names were generated by the software ChemDraw according to the guidelines specified by the International Union of Pure and Applied Chemistry (IUPAC). Melting points (M.p.) were obtained using a Eisco Melting Point Apparatus 230 V, 50–60 Hz and are uncorrected. Melting points were measured on a mixture when compounds could not be separated by column chromatography. Fourier‐transform infrared (FTIR) spectra were recorded from evaporated films on a Bruker Tensor 27 spectrometer equipped with a Pike Miracle Attenuated Total Reflectance (ATR) sampling accessory. ^1^H and ^13^C NMR experiments were carried out using Bruker NMR spectrometers (400, 500 or 600 MHz) in the deuterated solvent stated, using the residual non‐deuterated solvent signal as an internal reference. Chemical shifts (δ) are given in ppm and coupling constants (*J*) are quoted to the nearest 0.1 hertz (Hz) and are presented as observed. Resonances are described as s (singlet), d (doublet), t (triplet), q (quartet), br (broad singlet), dd (double of doublets), dt (doublet of triplets), dq (doublet of quartets), td (triplet of doublets), tt (triplet of triplets), tq (triplet of quartets), ddd (doublet of doublets of doublets), ddt (doublet of doublets of triplets), dddd (doublet of doublet of doublet of doublets), qd (quartet of doublets) and m (multiplet). ^1^H and ^13^C NMR peaks for diastereomers were assigned major or minor. For unseparable mixtures of diastereomers (>80 : 20 *dr*) both isomers are reported separately. When ^1^H and ^13^C NMR data for the major diastereoisomer is reported, any signals that overlap with the minor isomer have been integrated without the contribution from the minor isomer being included. In such a case, signals of the minor isomer are not reported. For equimolar mixtures of diastereomers (*dr*≤70 : 30) ^1^H NMR data for isomers is reported together. High resolution mass spectrometry (HRMS) under Supporting Information conditions were recorded on a Thermo Exactive Orbitrap mass spectrometer equipped with a Waters Equity LC system, a Bruker MicroToF mass spectrometer equipped with an Agilent 1100 HPLC pump and autosampler, or on a Waters Xevo Quadrupole Time of Flight (Q‐ToF) mass spectrometer. Atmospheric pressure chemical ionisation (APCI) HRMS were recorded on the abovementioned Thermo Exactive spectrometer under identical conditions using N_2_ as the reagent gas. Electron impact ionisation (EI) HRMS were performed on an Agilent 7200 Quadrupole Q‐ToF mass spectrometer equipped with a direct insertion probe supplied by Scientific Instrument Manufacturer (SIM) GmbH. Instrument control and data processing were performed using the software Agilent MassHunter. Unless otherwise specified, the mass reported for HRMS is the mass‐to‐charge ratio containing the most abundant isotopes, with each value to 4 or 5 decimal places and within 5 ppm of the calculated mass. Single crystal X‐ray diffraction was performed by Dr. Kirsten E. Christensen on a (Rigaku) Oxford Diffraction/Agilent Supernovae A diffractometer using Cu‐K_α_ radiation (*λ*=1.54184 Å) and a graphite monochromator. Samples were mounted on perfluoropoly‐ethyl ether oil and cooled by a Cryostream N_2_ open‐flow cooling device to 150 K throughout the data collection process. The diffraction patterns were integrated and reduced using the software CrysAlisPro. The software CRYSTALS for Microsoft Windows was used to obtain ab initio solutions (using SuperFlip embedded within CRYSTALS) and carry out structure refinement. The images of the solved structures were generated with the softwares Mercury and Ortep3 and represent displacement ellipsoid plots of the best‐fit model drawn at 50 % probability level.

**General experimental procedure for the cyclisation cascade using diene substrates**: The diene (1.00 equiv.) and the corresponding alcohol (1.00 equiv.) were transferred to a microwave vial which was then purged with nitrogen. The balloon of nitrogen was removed and HFIP followed by a stock solution of Ti(O*i*Pr)_4_ in HFIP (0.30 equiv., 0.066 M) were added to obtain an overall 0.050 M solution of the diene substrate. The solution was heated at 70 °C in an oil bath for 2 h, then cooled to rt and diluted with water (3 mL). The obtained suspension was transferred to a separatory funnel and extracted with CH_2_Cl_2_ (3×4 mL). The organic layers were combined, washed with brine and volatiles were removed in vacuo. The crude product was purified by flash column chromatography using the appropriate mixture of eluents.

**(±)‐(1*R*,2*R*,4*aS*,10*aS*)‐2‐(4‐Methoxybenzyl)‐1‐(4‐methoxyphenyl)‐4** ***a*** **‐methyl‐1,2,3,4,4** ***a*** **,9,10,10** ***a*** **‐octahydrophenanthrene 2 a**: Diene **1** (25.0 mg, 81.6 μmol, 1.00 equiv.), 4‐methoxybenzyl alcohol (11.3 mg, 81.6 μmol, 1.00 equiv.) and Ti(O*i*Pr)_4_ (7.4 μL, 25 μmol, 0.30 equiv.) in HFIP (1.6 mL, 0.050 M) were subjected to the general procedure. The crude product was purified by flash column chromatography (SiO_2_; 60 Å, 15–40 μm, pentane : Et_2_O; 24 : 1) to furnish compound **2 a** as a colourless oil which crystallised on standing (22.7 mg, 65 %). ^**1**^ **H NMR** (400 MHz, CDCl_3_): δ 7.30 (dd, *J*=7.8, 1.5 Hz, 1H), 7.20 (dd, *J*=8.7, 2.2 Hz, 1H), 7.16–6.95 (m, 5H), 6.91 (d, *J*=8.6 Hz, 2H), 6.84 (dd, *J*=8.3, 2.9 Hz, 1H), 6.77 (d, *J*=8.6 Hz, 2H), 3.84 (s, 3H), 3.77 (s, 3H), 2.80–2.61 (m, 2H), 2.51 (dd, *J*=13.4, 2.8 Hz, 1H), 2.38–2.25 (m, 2H), 2.00 (dd, *J*=13.4, 10.1 Hz, 1H), 1.78–1.65 (m, 3H), 1.55–1.34 (m, 3H), 1.31–1.22 (m, 1H), 1.19 (s, 3H); ^**13**^ **C NMR** (101 MHz, CDCl_3_): δ 158.1, 157.7, 148.1, 136.7, 135.7, 133.6, 132.7, 130.1 (2 C), 129.3, 125.9, 125.7, 125.6, 124.7, 115.3, 113.6 (2 C), 112.8, 55.4, 55.3, 50.5, 47.8, 46.1, 40.4, 37.6 (2 C), 29.6, 27.5, 22.7 (2 C); **M.p**: 139 °C; **IR** (film) *ν*_max_: 2929, 2834, 1611, 1510, 1301, 1243, 1177, 1027, 829, 809, 761 cm^−1^; **HRMS** (ESI): calculated for C_30_H_35_O_2_ [M+H]^+^ requires m/z 427.2632, found m/z 427.2632 (*Δ*=0.06 ppm).

**(±)‐(1*R*,2*S*,4*aS*,10*aS*)‐2‐Benzhydryl‐6‐methoxy‐1‐(4‐methoxyphenyl)‐4** ***a*** **‐methyl‐1,2,3,4,4** ***a*** **,9,10,10** ***a*** **‐octahydrophenanthrene 12**: Diene **7** (24.8 mg, 73.8 μmol, 1.00 equiv.), benzhydrol (13.6 mg, 73.8 μmol, 1.00 equiv.) and Ti(O*i*Pr)_4_ (6.70 μL, 22.6 μmol, 0.306 equiv.) in HFIP (1.5 mL, 0.050 M) were subjected to the general procedure. The crude product was purified by flash column chromatography (SiO_2_; 60 Å, 15–40 μm; pentane : Et_2_O; 24 : 1) to furnish compound **12** as a colourless foam (20.5 mg, 55 %). ^**1**^ **H NMR** (400 MHz, CDCl_3_): δ 7.35–7.07 (m, 11H), 6.95–6.80 (m, 5H), 6.67 (dd, *J*=8.3, 2.7 Hz, 1H), 3.94 (d, *J*=3.1 Hz, 1H), 3.84 (s, 3H), 3.78 (s, 3H), 2.70–2.48 (m, 3H), 2.43–2.29 (m, 2H), 1.97–1.86 (m, 1H), 1.77–1.60 (m, 3H), 1.38–1.24 (m, 1H), 1.16 (ddt, *J*=13.2, 7.7, 2.5 Hz, 1H), 1.00 (s, 3H); ^**13**^ **C NMR** (101 MHz, CDCl_3_): δ 158.1, 157.7, 149.1, 144.7, 141.7, 136.1, 133.3, 131.0 (2 C), 130.0, 128.4 (2 C), 128.1 (2 C), 127.9 (2 C), 127.8, 126.3, 126.1, 125.7, 115.1, 112.8, 110.9, 110.6, 55.4 (2 C), 51.8, 49.1, 47.9, 47.4, 38.0, 37.8, 28.7, 23.9, 22.8 (2 C); **IR** (film) *ν*_max_: 2939, 1610, 1510, 1250, 1178, 1038, 908, 731, 704 cm^−1^; **M.p**. 168–170 °C; **HRMS** (ESI): calculated for C_36_H_38_O_2_Na [M+Na]^+^ requires m/z 525.2764, found m/z 525.2767 (*Δ*=0.63 ppm).

**General experimental procedure for the cyclisation cascade using triene substrates**: The triene (1.00 equiv.) and the corresponding alcohol (1.00 equiv.) were transferred to a microwave vial which was then purged with nitrogen. The balloon of nitrogen was removed and HFIP followed by a stock solution of Ti(O*i*Pr)_4_ in HFIP (0.30 equiv., 0.066 M) were added to obtain an overall 0.050 M solution of a polyene substrate. The solution was stirred at rt for 1 h, then diluted with water (3 mL). The obtained suspension was transferred to a separatory funnel and extracted with CH_2_Cl_2_ (3×4 mL). The organic layers were combined, washed with brine and volatiles were removed in vacuo. Crude product was purified by flash column chromatography using the appropriate mixture of eluents.

**(±)‐(1*R*,2*S*,4a*S*,10a*S*)‐2‐Benzhydryl‐1‐((*E*)‐4‐methoxystyryl)‐4a‐methyl‐1,2,3,4,4a,9,10,10a‐octahydrophenanthrene (major)‐6a and (±)‐(1*S*,2*S*,4a*S*,10a*S*)‐2‐Benzhydryl‐1‐((*E*)‐4‐methoxystyryl)‐4a‐methyl‐1,2,3,4,4a,9,10,10a‐octahydrophenanthrene (minor)‐6a**: Triene **4** (33.2 mg, 0.100 mmol, 1.00 equiv.), benzhydrol (18.4 mg, 0.100 mmol, 1.00 equiv.) and Ti(O*i*Pr)_4_ (9.0 μL, 30 μmol, 0.30 equiv.) in HFIP (2.0 mL, 0.050 M) were subjected to the general procedure. The crude product was purified by flash column chromatography (SiO_2_; pentane : Et_2_O; 24 : 1) to furnish compound **6 a** as an inseparable mixture of two diastereomers at the exocyclic allylic position as a colourless oil (33.2 mg, 67 %, 60 : 40 *dr*). ^**1**^ **H NMR** (600 MHz, CDCl_3_): δ 7.36–7.03 (m, 16H_maj_, 16H_min_), 6.92 (d, *J*=8.6 Hz, 2H_min_), 6.88 (d, *J*=8.6 Hz, 2H_maj_), 6.32–6.23 (m, 1H_maj_, 1H_min_), 5.78–5.68 (m, 1H_maj_, 1H_min_), 4.42 (d, *J*=3.9 Hz, 1H_maj_), 3.85 (s, 3H_min_), 3.84 (s, 3H_maj_), 3.66 (d, *J*=11.8 Hz, 1H_min_), 2.97–2.77 (m, 2H_maj_, 2H_min_), 2.51 (tt, *J*=11.5, 4.0 Hz, 1H_min_), 2.42 (dt, J=9.3, 4.2 Hz, 1H_min_), 2.39–2.32 (m, 1H_maj_, 1H_min_), 2.32–2.24 (m, 1H_maj_), 2.14 (q, *J*=10.2 Hz, 1H_maj_), 1.94 (ddt, *J*=13.0, 6.1, 2.8 Hz, 1H_maj_), 1.89–1.76 (m, 1H_maj_, 2H_min_), 1.67–1.52 (m, 3H_maj_, 3H_min_ ), 1.50–1.41 (m, 1H_maj_, 1H_min_), 1.24 (s, 3H_min_), 1.00 (s, 3H_maj_); ^**13**^ **C NMR** (151 MHz, CDCl_3_) **for the major diastereomer** (from the mixture): δ 159.0, 147.8, 144.9, 142.5, 135.7, 132.2 (2 C), 130.6 (2 C), 130.5, 129.3, 128.7 (2 C), 128.3 (2 C), 128.0 (2 C), 127.3 (2 C), 126.2, 125.7 (2 C), 125.6, 124.7, 114.1 (2 C), 55.5, 53.1, 47.1, 46.8, 46.5, 37.8, 37.1, 29.7, 24.3, 23.1, 22.9. **Selected peaks for the minor diastereomer** (from the mixture): 159.0, 149.2, 144.6, 143.4, 135.5, 132.6, 131.0, 129.2, 128.7, 128.2, 127.3, 126.1 (2 C), 125.8, 125.6, 125.5, 124.5, 114.1, 56.4, 47.4, 46.4, 45.2, 38.8, 37.4, 29.8, 26.1, 24.7, 23.7; **IR** (film) ν_max_: 3026, 2933, 2361, 1607, 1510, 1492, 1450, 1295,1250, 1174, 1034, 970, 761, 737, 704 cm^−1^; **HRMS** (ESI): calculated for C_37_H_39_O [M+H]^+^ requires m/z 499.2995, found m/z 499.2992 (*Δ*=−0.70 ppm).

### Synthesis of an 18‐nor‐estradiol precursor 19 by ring‐closing metathesis and hydroboration‐oxidation protocol of compound 17

**(±)‐(8*S*,9*S*,14*S*)‐3‐Methoxy‐7,8,9,11,12,14,15,16‐octahydro‐6H‐cyclopenta[*a*]phenanthrene 19**: Alkene **S22** (20.0 mg, 40.4 μmol, 1.00 equiv.) and 2^nd^ generation Hoveyda‐Grubbs catalyst (7.6 mg, 12.1 μmol, 0.300 equiv.) were transferred to a microwave vial and purged with nitrogen for 15 min. Balloon of nitrogen was removed and degassed PhMe (0.81 mL) was added. The solution was stirred at 110 °C overnight, cooled to rt and volatiles were removed in vacuo. The crude product was purified by flash column chromatography (SiO_2_; pentane to pentane : Et_2_O; 100 : 0 to 98 : 2) to afford product **19** as a colourless oil (5.3 mg, 51 %). ^**1**^ **H NMR** (600 MHz, CDCl_3_) δ 7.24 (d, *J*=8.6 Hz, 1H), 6.72 (dd, *J*=8.7, 2.8 Hz, 1H), 6.62 (d, *J*=2.8 Hz, 1H), 5.34–5.28 (m, 1H), 3.78 (s, 3H), 2.88–2.76 (m, 2H), 2.64 (ddd, *J*=14.2, 4.2, 2.0 Hz, 1H), 2.50–2.40 (m, 2H), 2.35–2.25 (m, 3H), 2.25–2.13 (m, 2H), 1.98 (ddt, *J*=12.8, 5.4, 2.8 Hz, 1H), 1.52–1.38 (m, 2H), 1.21 (qd, *J*=11.9, 4.3 Hz, 1H), 1.04 (qd, *J*=10.6, 2.7 Hz, 1H); ^**13**^ **C NMR** (101 MHz, CDCl_3_): δ 157.4, 145.3, 138.5, 132.3, 127.4, 121.1, 113.8, 111.9, 55.3, 51.3, 49.2, 42.3, 32.0, 31.5, 30.6, 29.2, 28.7, 27.9; **IR** (film) ν_max_: 2927, 2854, 2360, 1611, 1501, 1254, 1237, 1048, 909, 802, 735 cm^−1^; **HRMS** (ESI): calculated for C_18_H_23_O [M+H]^+^ requires m/z 255.1743, found m/z 255.1744 (*Δ*=0.35 ppm).

**(±)‐(8*S*,9*S*,13*S*,14*S*,17*S*)‐7,8,9,11,12,13,14,15,16,17‐Decahydro‐6H‐cyclopenta[a]phenanthren‐17‐ol 18 (major) and (±)‐(8*S*,9*S*,13*R*,14*S*,17*R*)‐7,8,9,11,12,13,14,15,16,17‐Decahydro‐6H‐cyclopenta[a]phenanthren‐17‐ol 18 (minor)**: Alkene **17** (6.1 mg, 27 μmol, 1.0 equiv.) and LiBH_4_ (cat) were transferred to a microwave vial which was then purged with nitrogen. Catecholborane (7.3 μL, 68 μmol, 2.5 equiv.) as a stock solution in C_6_H_6_ (0.40 mL, 0.17 M) was then added in one portion and the obtained suspension was stirred at reflux for 24 h. Mixture was then cooled to rt and aq NaOH (3 M, 0.2 mL) followed by aq H_2_O_2_ (30 %, 0.2 mL) were added in one portion. The obtained mixture was vigorously stirred at rt for 4 h, diluted with water (3 mL) and CH_2_Cl_2_ (3 mL). The organic layer was removed, and the aqueous layer was extracted with CH_2_Cl_2_ (3×3 mL). Organic layers were combined, washed with brine, and dried over Na_2_SO_4_. The crude product was purified by flash column chromatography (SiO_2_; pentane : Et_2_O; 3 : 2) to furnish an inseparable mixture of alcohols **18 (major)** and **18 (minor)** as a white solid (4.8 mg, 61 : 39 *dr*, 73 %). ^**1**^ **H NMR** (600 MHz, CDCl_3_): δ 7.35–7.28 (m, 1H_maj_, 1H_min_), 7.18–7.05 (m, 3H_maj_, 3H_min_), 4.13 (td, *J*=8.7, 6.7 Hz, 1H_min_), 3.86 (td, *J*=8.6, 6.1 Hz, 1H_maj_), 2.93–2.79 (m, 2H_maj_, 2H_min_), 2.61–2.54 (m, 1H_maj_), 2.36–2.28 (m, 1H_maj_, 2H_min_), 2.25–2.20 (m, 1H_maj_), 2.20–2.08 (m, 1H_maj_, 1H_min_), 2.05 (dq, *J*=14.0, 3.1 Hz, 1H_min_), 2.01–1.89 (m, 1H_maj_, 3H_min_), 1.89–1.76 (m, 1H_maj_, 2H_min_), 1.64–1.49 (m, 1H_maj_, 2H_min_), 1.46–1.20 (m, 6H_maj_, 2H_min_), 1.15 (qd, *J*=10.8, 6.5 Hz, 1H_maj_), 1.02 (qd, *J*=11.1, 2.7 Hz, 1H_min_); ^**13**^ **C NMR** (151 MHz, CDCl_3_) **for the major diastereomer** (from the mixture): δ 140.3, 137.0, 129.2, 125.8, 125.8, 125.7, 78.0, 53.6, 48.1, 45.8, 43.6, 33.1, 30.6, 29.8, 29.0, 27.5, 26.7. **Peaks for the minor diastereomer** (from the mixture): δ 140.4, 137.2, 129.1, 126.1, 125.8, 125.7, 74.3, 47.6, 44.0, 42.7, 41.7, 33.0, 30.3, 28.1, 26.8, 26.5, 24.9; **M.p**: 120–130 °C; **IR** (film) ν_max_: 3315, 2930, 2863, 2360, 2341, 1489, 1451, 1073, 912, 742 cm^−1^; **HRMS** (GC EI): calculated for C_17_H_22_O [M]^+^ requires m/z 242.16652, found m/z 242.16707 (*Δ*=3.21 ppm).

## Supporting Information

The Supporting Information is available free of charge from LINK etc.

Deposition Numbers 2191594 (**2 a**), 2191595 (**2 d**), 2191596 (**2 g**), 2191597 (**12**), 2191598 (**14**) contain the supplementary crystallographic data for this paper. These data are provided free of charge by the joint Cambridge Crystallographic Data Centre and Fachinformationszentrum Karlsruhe Access Structures service.

## Author Contribution Section

TJD and DA conceived the project and DA carried out all synthetic experimental work and compound characterisation. RJ assisted TJD with the running of the project. TJD and DA prepared the manuscript. KEC performed the X‐ray structure analysis.

## Conflict of interest

The authors declare no conflict of interest.

1

## Supporting information

As a service to our authors and readers, this journal provides supporting information supplied by the authors. Such materials are peer reviewed and may be re‐organized for online delivery, but are not copy‐edited or typeset. Technical support issues arising from supporting information (other than missing files) should be addressed to the authors.

Supporting Information

## Data Availability

The data that support the findings of this study are available in the supplementary material of this article.
