# Supplementary material for: Alcohols as Efficient Intermolecular Initiators for a Highly Stereoselective Polyene Cyclisation Cascade
Source: Chemistry. 2023 Jan 30;29(13):e202203732. doi: 10.1002/chem.202203732 (PMC10946764; doi:10.1002/chem.202203732)

Chemistry–A European Journal

Supporting Information

Alcohols as Efficient Intermolecular Initiators for a Highly Stereoselective Polyene Cyclisation Cascade

Daniya Aynetdinova, Reece Jacques, Kirsten E. Christensen, and Timothy J. Donohoe*

Contents

1.0 General remarks ... 2

2.0 Reaction optimisation. ... 5

3.0 Unsuccessful alcohols for the alkylative polyene cyclisation. ... 7

4.0 Experimental procedures. ... 8

*4.1 Synthesis of E-**1**, **7-10** and Z-**1**.* ... 8

*4.2 Synthesis of **11**.* ... 17

*4.3 Synthesis of diene **3**.* ... 20

*4.4 Synthesis of E/Z-**4** and E/Z-**5**.* ... 24

4.5 Synthesis of alcohols using modified literature procedures. ... 28

*4.6 Cyclisation cascade methodology: synthesis of **2a**-**2w**.* ... 32

*4.7 Cyclisation cascade methodology: synthesis of **6a-6f**.* ... 57

*4.8 Cyclisation cascade methodology: synthesis of **12-16.*** ... 64

*4.9 Derivatisation of **6d**: synthesis of an 18-norsteroid analogue **18**.* ... 69

*4.10 Formal synthesis of a 18-nor-estradiol precursor **19**.* ... 71

*4.11 Synthesis of a steroid analogue with a C-9 methyl group and ozonolysis of **2q**.* ... 79

5.0 Control and mechanistic experiments. ... 81

5.1 Stereoconvergent experiments. ... 81

*5.2 Stability of Z-**1**, E-**1**, E/Z-**4** under the reaction conditions.* ... 82

5.3 Reaction with electron-deficient benzyl alcohol. ... 83

5.4 Reaction with transposed isomers of unsymmetrical allylic alcohol. ... 84

6.0 Single crystal X-ray diffraction for compounds 2a, 2d, 2g, 12 and **14**. ... 86

7.0 ^{1}H, ^{13}C and representative 2D NMR spectra. ... 91

7.1 Polyene cyclisation precursors and intermediates ... 91

*7.2 Products **2a-2w**.* ... 126

*7.3 Products **6a**-**6f**.* ... 194

*7.4 Products **12**-**16**.* ... 213

*7.5 Derivatisation: products **17** and **18**.* ... 226

*7.6 Formal synthesis of a 18-nor-estradiol precursor **19**.* ... 234

*7.7 Steroid analogue with a C-9 methyl group **S23** and aldehyde **S24**.* ... 257

*7.6 Reaction of E/Z-**1** with benzhydrol.* ... 262

1.0 General remarks

Reaction Setup: Microwave vials and vial caps (containing a resealing Silicone/PTFE septum) were purchased from Kinesis (Cole-Palmer) and were used without flame-drying. Unless otherwise stated, all other reactions were performed in flame-dried glassware equipped with a stir bar using standard Schlenk techniques under an atmosphere of N_2. Room temperature (rt) refers to 20-25 °C. Temperatures of 0 °C were obtained using an ice/water bath. Temperatures of –17 °C were obtained using a salt/ice bath. Temperatures of –78 °C were obtained using a dry ice/acetone bath. Reflux conditions were obtained using an oil bath equipped with a contact thermometer. Other temperatures were obtained using a Julabo FT902 immersion cooler.

Reagents and solvents: Unless detailed below or otherwise stated, all reagents and solvents were purchased and used as supplied from Sigma-Aldrich (now Merck KGaA), Thermo Fisher Scientific (including Alfa Aesar and Acros Organics), Fluorochem, Honeywell, Tokyo Chemical Industry, Apollo Scientific, Manchester Organics (part of Navin Fluorine Int. Ltd.) and Strem Chemicals.

Anhydrous DMSO and Et_3N were purchased from Sigma-Aldrich in Sure/SealTM bottles. Anhydrous Et_2O, CH_2Cl_2, MeOH, THF and toluene were obtained from MBRAUN SPS-5 solvent purification system by passage through double filtration columns under N_2. 1,1,1,3,3,3-Hexafluoro-2-propanol (HFIP) was purchased from Fluorochem. $Ti(O\textit{i}Pr)_4$ (≥97%) was purchased from Sigma-Aldrich. Alcohols were purchased from commercial suppliers or prepared using standard literature procedures unless otherwise stated.

Chromatography: Thin layer chromatography (TLC) was performed on pre-coated aluminium (200 μm) Merck Kieselgel 60 F254 plates, visualised using UV irradiation (λ = 254 nm) and/or staining with potassium permanganate (KMnO4), vanillin, or phosphomolybdic acid (PMA) solutions. Purification by flash column chromatography was performed with Merck Kieselgel 60 (40– 63 μm) or (15–40 μm) silica gel using head pressure by means of a nitrogen line. All solvents used for chromatography were HPLC grade or equivalent and supplied by Honeywell, Sigma-Aldrich or Thermo Fisher Scientific.

Nomenclature: Systematic names were generated by the software ChemDraw according to the guidelines specified by the International Union of Pure and Applied Chemistry (IUPAC). Conventional names are also provided for reference where applicable. All compounds reported are racemates, and stereodescriptors drawn in the structures are relative. For meso and other symmetric achiral cyclic compounds, the prefixes cis and trans are used for clarity where appropriate.

Compound Numbering: Where applicable, atom numbering shown does not follow IUPAC numbering and is therefore different to the compound name.

Melting points: Melting points (M.p.) were obtained using a Eisco Melting Point Apparatus 230V, 50-60Hz and are uncorrected. Melting points were measured on a mixture when compounds could not be separated by column chromatography.

Infrared spectroscopy: Fourier-transform infrared (FT-IR) spectra were recorded from evaporated films on a Bruker Tensor 27 spectrometer equipped with a Pike Miracle Attenuated Total Reflectance (ATR) sampling accessory. Absorption maxima are quoted in wavenumbers (v_{max}) with units of cm^{-1} and for the range of 3600–600 cm^{-1}. IR spectra were measured on a mixture when compounds could not be separated by column chromatography.

NMR spectroscopy: ^{1}H and ^{13}C NMR experiments were carried out using Bruker NMR spectrometers (400, 500 or 600 MHz) in the deuterated solvent stated, using the residual non-deuterated solvent signal as an internal reference. Chemical shifts (δ) are given in ppm and coupling constants (*J*) are quoted to the nearest 0.1 hertz (Hz) and are presented as observed. Resonances are described as s (singlet), d (doublet), t (triplet), q (quartet), br (broad singlet), dd (double of doublets), dt (doublet of triplets), dq (doublet of quartets), td (triplet of doublets), tt (triplet of triplets), tq (triplet of quartets), ddd (doublet of doublets of doublets), ddt (doublet of doublets of triplets), dddd (doublet of doublet of doublet of doublets), qd (quartet of doublets) and m (multiplet). ^{1}H and ^{13}C NMR peaks for diastereomers were assigned major or minor. Assignments were made with the assistance of gCOSY, gHSQC, gHMBC or NOESY NMR spectra. For unseparable mixtures of diastereomers (>80:20 dr) both isomers are reported separately. When ^{1}H and ^{13}C NMR data for the major diastereoisomer is reported, any signals that overlap with the minor isomer have been integrated without the contribution from the minor isomer being included. In such a case, signals of the minor isomer are not reported. For equimolar mixtures of diastereomers (dr ≤ 70:30) ^{1}H NMR data for isomers is reported together.

Mass spectrometry (MS): High resolution mass spectrometry (HRMS) under ESI conditions were recorded on a Thermo Exactive Orbitrap mass spectrometer equipped with a Waters Equity LC system, a Bruker MicroToF mass spectrometer equipped with an Agilent 1100 HPLC pump and autosampler, or on a Waters Xevo Quadrupole Time of Flight (Q-ToF) mass spectrometer. The Thermo Exactive system employs a flow rate of 0.2 mL min–1 using H_2O:MeOH:HCOOH (10:89.9:0.1) as eluent, with a heated electrospray ionisation (HESI-II) probe and has a resolution of 50,000 FWHM. The Bruker system uses the built-in electrospray source, while the Waters system runs on a lock-mass mode with ESI performed by a secondary electrospray source, both using conditions identical to the Thermo Exactive

system. Instrument control and data processing were performed using the softwares Thermo Xcalibur for the Thermo Exactive system, Compass DataAnalysis 4.0 for the Bruker system, and MassLynx for the Waters system. Atmospheric pressure chemical ionisation (APCI) HRMS were recorded on the abovementioned Thermo Exactive spectrometer under identical conditions using N_2 as the reagent gas. Electron impact ionisation (EI) HRMS were performed on an Agilent 7200 Quadrupole Q-ToF mass spectrometer equipped with a direct insertion probe supplied by Scientific Instrument Manufacturer (SIM) GmbH. Instrument control and data processing were performed using the software Agilent MassHunter. All HRMS conditions were adjusted for maximum sensitivity, with an accuracy of better than 5 ppm for 24 h following external calibration on the day of analysis. Unless otherwise specified, the mass reported for HRMS is the mass-to-charge ratio containing the most abundant isotopes, with each value to 4 or 5 decimal places and within 5 ppm of the calculated mass. HRMS data was collected on a mixture when compounds could not be separated by column chromatography.

X-Ray Crystallography: Single crystal X-ray diffraction was performed by Dr. Kirsten E. Christensen on a (Rigaku) Oxford Diffraction/Agilent Supernovae A diffractometer using Cu-K_α radiation (λ = 1.54184 Å) and a graphite monochromator. Samples were mounted on perfluoropoly-ethyl ether oil and cooled by a Cryostream N_2 open-flow cooling device to 150 K throughout the data collection process. The diffraction patterns were integrated and reduced using the software CrysAlisPro. The software CRYSTALS for Microsoft Windows was used to obtain ab initio solutions (using SuperFlip embedded within CRYSTALS) and carry out structure refinement. The images of the solved structures were generated with the softwares Mercury and Ortep3 and represent displacement ellipsoid plots of the best-fit model drawn at 50% probability level.

2.0 Reaction optimisation.

Method: Diene **1** (1.00 equiv), 4-methoxybenzyl alcohol (1.00 equiv) and the corresponding Lewis acid (if solid, see **Table 1**) were transferred to a microwave vial which was then purged with nitrogen. The balloon of nitrogen was removed, and solvent was added to obtain the desired concentration of diene **1** (see **Table 1**). Lewis or Brønsted acid was then added (if liquid, see **Table 1**) in one portion and the obtained solution was heated at 70 °C in an oil bath for 2 h, then cooled to rt and diluted with water (3 mL). The obtained suspension was transferred to a separatory funnel and extracted with CH_2Cl_2 (3 × 4 mL). The organic layers were combined, washed with brine and volatiles were removed *in vacuo*. The crude product was purified by flash column chromatography (SiO_2; 60 Å, 15–40 μm, pentane:Et_2O; 24:1) to furnish product **3a**.

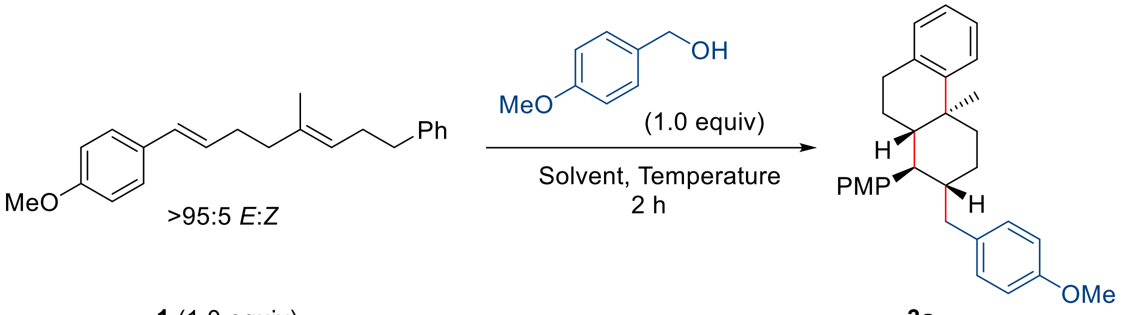

1 (1.0 equiv) **3a**

	Reaction solvent	Conc (M)	T (°C)	Lewis acid	Loading (mol%)	Yield of 3a (%)[a]
1	HFIP	0.1	70	Ti(O*i*Pr)$_4$	30	48
**2**	**HFIP**	**0.05**	**70**	**Ti(O*i*Pr)$_4$**	**30**	**65**
3	HFIP	0.05	70	Ti(O*i*Pr)$_4$	30	25[b]
4	HFIP	0.02	70	Ti(O*i*Pr)$_4$	30	40
5	HFIP	0.05	40	Ti(O*i*Pr)$_4$	30	37
6	HFIP	0.05	80	Ti(O*i*Pr)$_4$	30	40
7	HFIP	0.05	70	Ti(O*i*Pr)$_4$	50	52
8	HFIP	0.05	70	Ti(O*i*Pr)$_4$	20	53
9	HFIP	0.05	70	PPh_4PBF_4	30	38
10	HFIP	0.05	70	$Zn(OTf)_2$	30	34
11	HFIP	0.05	70	$Zn(OAc)_2$	30	45
12	HFIP	0.05	70	$AlCl_3$	30	15
13	HFIP	0.05	70	TFA	30	31
14	HFIP	0.05	70	-	0	49
15	*i*-PrOH	0.05	70	Ti(O*i*Pr)$_4$	30	0[c]
16	DMF	0.05	70	Ti(O*i*Pr)$_4$	30	0[c]
17	EtOAc	0.05	70	Ti(O*i*Pr)$_4$	30	0[c]
18	MeCN	0.05	70	Ti(O*i*Pr)$_4$	30	0[c]
19	MeOH	0.05	70	Ti(O*i*Pr)$_4$	30	0[c]
20	PhMe	0.05	70	Ti(O*i*Pr)$_4$	30	0[c]
21	DCE	0.05	70	Ti(O*i*Pr)$_4$	30	0[c]

Table 1. Reaction optimisation; All reactions were performed on 25 mg scale; Relative stereochemistry for **3a** was confirmed by NOESY analysis and X-ray crystal structure; [a] Isolated yields are reported; [b] Reaction performed with 1.5 equiv of 4-methoxybenzyl alcohol; [c] no conversion over 2 and 24 h.

3.0 Unsuccessful alcohols for the alkylative polyene cyclisation.

Diene **1** (1.00 equiv) and the corresponding alcohol (1.00 equiv) were transferred to a microwave vial which was then purged with nitrogen. The balloon of nitrogen was removed and HFIP followed by a stock solution of Ti(O*i*Pr)$_4$ (30 mol%) in HFIP (0.066 M) were added to obtain 0.05 M solution of a diene **1** in HFIP. The obtained solution was stirred at rt or heated at 70 °C in an oil bath for 2-24 h, then cooled to rt and diluted with water (3 mL). The obtained suspension was transferred to a separatory funnel and extracted with CH_2Cl_2 (3 × 4 mL). The organic layers were combined, washed with brine and volatiles were removed *in vacuo*. The crude reaction mixture was analysed by NMR.

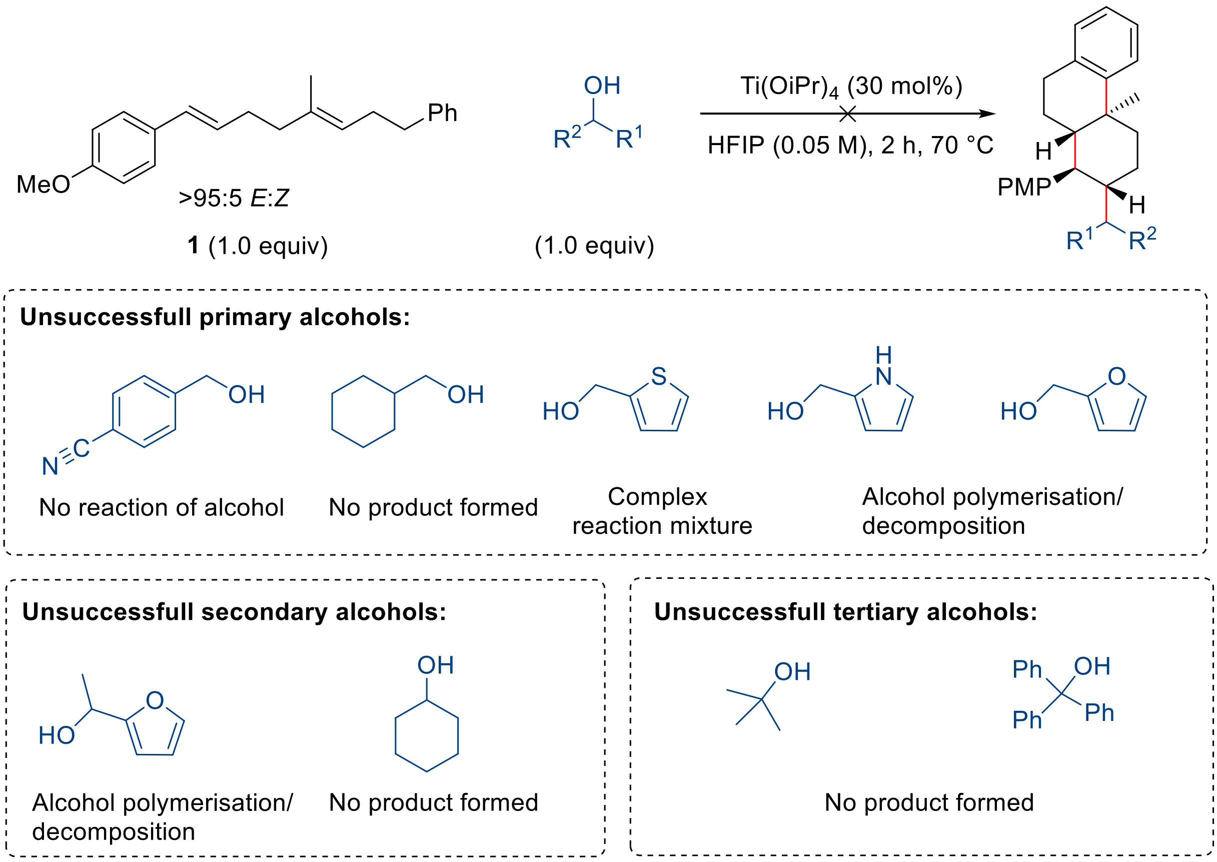

4.0 Experimental procedures.

*4.1 Synthesis of E-**1**, **7-10** and Z-**1**.*

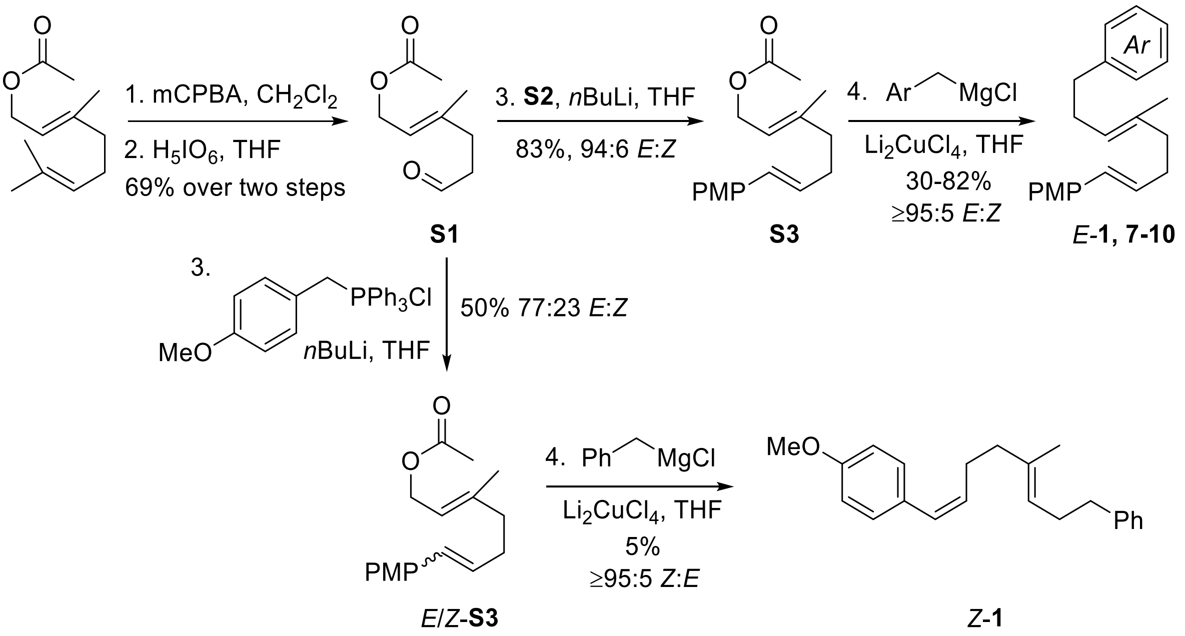

(*E*)-3-Methyl-6-oxohex-2-en-1-yl acetate S1.

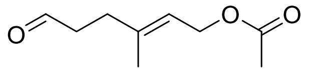

S1
$C_9H_{14}O_3$
170.21 g mol^{-1}

According to a literature procedure,[1] to a solution of geranyl acetate (0.41 g, 2.1 mmol, 1.0 equiv) in CH_2Cl_2 (10 mL) at -10 °C was added m-CPBA (77% wt, 0.46 g, 2.1 mmol, 1.0 equiv) portionwise. The resulting slurry was warmed to rt, stirred for 2 h and then poured into 2 M NaOH (10 mL). The layers were separated, and the aqueous phase was extracted with CH_2Cl_2 (2 × 10 mL). The organic layers were combined, washed with brine (30 mL) and dried over Na_2SO_4. Solvent was removed *in vacuo* and the crude product was used in the next step without further purification. The crude product was redissolved in THF (1.5 mL) and then added dropwise to a solution of H_5IO_6 (0.47 g, 2.1 mmol, 1.0 equiv) in THF (1 mL) at 0 °C. Suspension was stirred at the same temperature for 1.5 h, poured into sat. aq. $NaHCO_3$ solution (5 mL) and then extracted with Et_2O (3 X 5 mL). The combined organic layers were washed with brine, dried over Na_2SO_4 and volatiles were removed *in vacuo*. The residue was purified by flash chromatography (SiO_2; pentane:Et_2O; 4:1) to furnish aldehyde **S1** as a colourless oil (0.25 g, 69% over two steps).

^{1}H NMR (400 MHz, $CDCl_3$): δ 9.78 (t, *J* = 1.7 Hz, 1H), 5.36 (tq, *J* = 7.0, 1.3 Hz, 1H), 4.58 (d, *J* = 7.0 Hz, 2H), 2.58 (td, *J* = 7.7, 1.6 Hz, 2H), 2.38 (t, *J* = 7.5 Hz, 2H), 2.05 (s, 3H), 1.72 (s, 3H).

^{13}C NMR (101 MHz, $CDCl_3$): δ 201.8, 171.2, 140.1, 119.5, 61.2, 41.9, 31.6, 21.2, 16.7.

The analytical data are consistent with those previously reported in the literature.[1]

Tributyl(4-methoxybenzyl)phosphonium chloride S2.

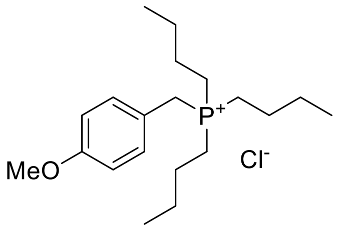

S2
$C_{20}H_{36}OPCl$
358.93 g mol^{-1}

4-Methoxybenzyl chloride (2.07 mL, 15.3 mmol, 1.05 equiv) and tri-*n*-butylphosphine (3.60 mL, 14.6 mmol, 1.00 equiv) were dissolved in MeCN (6 mL) and the solution was heated at 60 °C overnight. Volatiles were removed *in vacuo* and the residue was washed with pentane (3 × 10 mL). The remaining solid was dried under vacuum to obtain tri-*n*-butylphosphonium salt **S2** as a white waxy solid (4.96 g, 95%).

^{1}H NMR (400 MHz, $CDCl_3$): δ 7.41 – 7.31 (m, 2H), 6.95 – 6.85 (m, 2H), 4.17 (d, *J* = 14.6 Hz, 2H), 3.80 (s, 3H), 2.49 – 2.31 (m, 6H), 1.55 – 1.36 (m, 12H), 0.94 (t, *J* = 6.9 Hz, 9H).

^{13}C NMR (101 MHz, $CDCl_3$): δ 159.7 (d, *J* = 3.6 Hz), 131.3 (d, *J* = 4.8 Hz, 2C), 120.1 (d, *J* = 8.7 Hz), 114.9 (d, *J* = 2.8 Hz, 2C), 55.4, 26.2 (d, *J* = 45.3 Hz), 24.0 (d, *J* = 15.5 Hz, 3C), 23.8 (d, *J* = 4.8 Hz, 3C), 18.6 (d, *J* = 46.5 Hz, 3C), 13.5 (3C).

^{31}P (162 MHz, $CDCl_3$): δ 31.4

IR (film) ν_{max}: 3386, 2960, 2934, 2873, 2360, 1611, 1514, 1465, 1250, 1181, 1030, 844 cm^{-1}.

HRMS (ESI): calculated for $C_{20}H_{36}OP$ $[M]^+$ requires m/z 323.2498, found m/z 323.2500 (Δ = 0.62 ppm).

[1] S. J. Partridge, D. M. Withall, J. C. Caulfield, J. A. Pickett, R. A. Stockman, N. J. Oldham, M. A. Birkett, *Chem. Eur. J.* **2021**, *27*, 7231–7234.

(2*E*,6*E*)-7-(4-Methoxyphenyl)-3-methylhepta-2,6-dien-1-yl acetate *E*-S3 and (2*E*,6*Z*)-7-(4-Methoxyphenyl)-3-methylhepta-2,6-dien-1-yl acetate *Z*-S3.

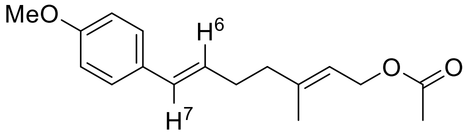

E-**S3** (Major)
$C_{17}H_{22}O_3$
274.36 g mol^{-1}
$^3J_{H\text{-}6,\,H\text{-}7}$ = 15.8 Hz

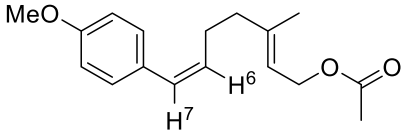

Z-**S3** (Minor)
$C_{17}H_{22}O_3$
274.36 g mol^{-1}
$^3J_{H\text{-}6,\,H\text{-}7}$ = 11.6 Hz

Tributyl(4-methoxybenzyl)phosphonium chloride **S2** (5.06 g, 14.1 mmol, 1.50 equiv) was suspended in THF (200 mL) and cooled to 0 °C. *n*BuLi (5.6 mL, 2.5 M in hexanes, 14 mmol, 1.5 equiv) was then added dropwise and the obtained suspension was allowed to stir at 0 °C for 30 min. After that mixture was cooled to -78 °C and aldehyde **S1** (1.60 g, 9.41 mmol, 1.00 equiv) in THF (18 mL) was added dropwise at the same temperature. The reaction mixture was allowed to warm to rt overnight and then quenched with sat. aq. NH_4Cl (20 mL). Mixture was diluted with water (100 mL) and extracted with Et_2O (3 × 100 mL). The organic layers were combined, washed with brine and volatiles were removed *in vacuo.* The crude product was purified by flash column chromatography (SiO_2, pentane:Et_2O; 9:1) to furnish an inseparable mixture of alkenes *E*-**S3** and *Z*-**S3** as a colourless oil (2.14 g, 83%, 94:6 *E*:*Z*).

Major *E*-S3 (from the mixture)**:**

^{1}H NMR (400 MHz, $CDCl_3$): δ 7.26 (d, *J* = 8.7 Hz, 2H), 6.83 (d, *J* = 8.7 Hz, 2H), 6.33 (d, *J* = 15.8 Hz, 1H), 6.04 (dt, *J* = 15.8, 6.8 Hz, 1H), 5.39 (tq, *J* = 7.1, 1.3 Hz, 1H), 4.59 (d, *J* = 7.1 Hz, 2H), 3.80 (s, 3H), 2.36 – 2.28 (m, 2H), 2.24 – 2.15 (m, 2H), 2.04 (s, 3H), 1.74 (3, 3H).

^{13}C NMR (101 MHz, $CDCl_3$): δ 171.3, 158.9, 141.9, 130.7, 129.7, 127.9, 127.2 (2C), 118.9, 114.1 (2C), 61.5, 55.4, 39.5, 31.3, 21.2, 16.6.

Characteristic peaks for the minor *Z*-S3 (from the mixture):

^{1}H NMR (400 MHz, $CDCl_3$): δ 7.21 (d, J = 8.7 Hz, 2H), 6.87 (d, *J* = 8.7 Hz, 2H), 5.53 (dt, *J* = 11.6, 7.2, Hz, 1H), 3.81 (s, 3H), 2.50 – 2.42 (m, 2H), 2.05 (s, 3H), 1.69 (s, 3H).

^{13}C NMR (101 MHz, $CDCl_3$): δ 130.0, 113.8.

IR (film) v_{max}: 2935, 1735, 1608, 1510, 1366, 1232, 1175, 1033, 964, 844 cm^{-1}.

HRMS (ESI): calculated for $C_{17}H_{22}O_3Na$ $[M+Na]^+$ requires m/z 297.1461, found m/z 297.1463 (Δ = 0.62 ppm).

1-Methoxy-4-((1*E*,5*E*)-5-methyl-8-phenylocta-1,5-dien-1-yl)benzene *E*-1.

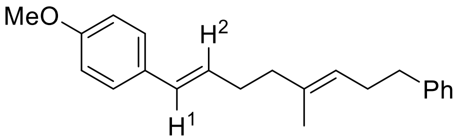

***E*-1**
$C_{22}H_{26}O$
306.45 g mol^{-1}
$^{3}J_{H-1, H-2}$ = 15.8 Hz

Mixture of alkenes *E*-**S3** and *Z*-**S3** (0.170 g, 0.620 mmol, 1.00 equiv, 94:6 *E*:*Z*) was dissolved in THF (10 mL) and cooled to 0 °C. Li_2CuCl_4 (1.9 mL, 0.10 M in THF, 0.19 mmol, 0.30 equiv) was then added at the same temperature. To the obtained solution BnMgCl (2.8 mL, 2.0 M in THF, 5.6 mmol, 9.0 equiv) was added dropwise over 15 min keeping temperature at 0 °C. The reaction mixture was allowed to stir at the same temperature for 3 h, quenched with sat. aq. NH_4Cl (3 mL), diluted with water (10 mL) and extracted with Et_2O (3 × 10 mL). The organic layers were combined, washed with brine (30 mL) and volatiles were removed *in vacuo* to obtain the crude product which was purified by flash column chromatography (SiO_2, 60 Å, 15–40 µm; pentane:Et_2O; 49:1) to furnish compound **1** as a colourless oil which solidified on standing (0.151 g, 80%, >95:5 *E:Z*).

^{1}H NMR (400 MHz, $CDCl_3$): δ 7.31 – 7.22 (m, 4H), 7.21 – 7.14 (m, 3H), 6.84 (d, *J* = 8.8 Hz, 2H), 6.33 (d, *J* = 15.8 Hz, 1H), 6.06 (dt, *J* = 15.8, 6.8 Hz, 1H), 5.27 – 5.19 (m, 1H), 3.80 (s, 3H), 2.68 – 2.59 (m, 2H), 2.37 – 2.23 (m, 4H), 2.17 – 2.08 (m, 2H), 1.58 (s, 3H).

^{13}C NMR (101 MHz, $CDCl_3$): δ 158.8, 142.5, 135.4, 130.9, 129.3, 128.7, 128.6 (2C), 128.4 (2C), 127.1 (2C), 125.8, 124.2, 114.1 (2C), 55.4, 39.7, 36.3, 31.8, 30.1, 16.2.

M.p. 34-35 °C.

IR (film) ν_{max}: 2915, 2361, 1608, 1511, 1248, 1175, 1038, 965 cm^{-1}.

HRMS (ESI): calculated for $C_{22}H_{27}O$ $[M+H]^+$ requires m/z 307.2056, found m/z 307.2057 (Δ = 0.25 ppm).

General procedure A: Synthesis of 7-10.

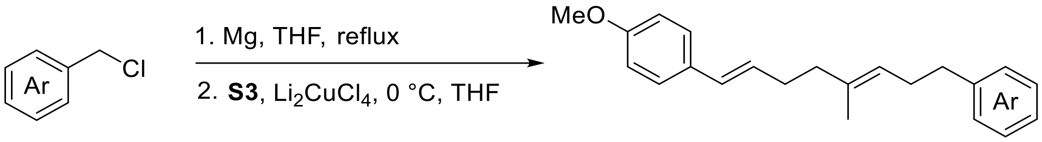

A flame-dried round-bottomed flask was charged with magnesium turnings (18 equiv) and catalytic amount of iodine. THF (0.42 M wrt benzyl chloride) was added followed by the dropwise addition of benzyl chloride (9.0 equiv) at such a rate to maintain gentle reflux. After the addition was complete the resulting suspension was stirred at rt for 2 h. The prepared benzyl magnesium chloride solution was used in the next step immediately.

Mixture of acetates (*E*)-**S3** and (*Z*)-**S3** (1.0 equiv, 94:6 *E*:*Z*) was dissolved in THF (0.03 M) and cooled to 0 °C. To the obtained solution Li_2CuCl_4 (0.10 M in THF, 0.20 equiv) was added in one portion followed by the dropwise addition of the corresponding benzyl magnesium chloride (3.0 equiv). The reaction was allowed to stir at 0 °C for 1 h and then progress was montitored by TLC. If starting material was present, additional portion of benzyl magnesium chloride (3.0 equiv) was added dropwise and the solution was allowed to stir for another 2 h. The reaction mixture was quenched with sat. aq. NH_4Cl (5 mL), diluted with water (10 mL) and extracted with Et_2O (3 × 10 mL). The organic layers were combined, washed with brine (30 mL) and volatiles were removed *in vacuo* to obtain the crude product which was purified by flash column chromatography using the appropriate mixture of eluents.

4,4'-((1*E*,5*E*)-5-Methylocta-1,5-diene-1,8-diyl)bis(methoxybenzene) 7.

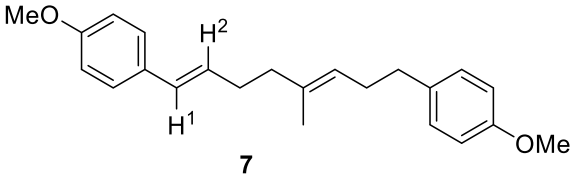

7
$C_{23}H_{28}O_2$
336.48 g mol^{-1}
$^3J_{H\text{-}1,\,H\text{-}2}$ = 15.8 Hz

4-Methoxybenzyl chloride (0.30 mL, 2.2 mmol, 9.0 equiv) was used to prepare the Grignard reagent which was reacted with (*E*/*Z*)-**S3** (67.2 mg, 0.245 mmol, 1.00 equiv, 94:6 *E*:*Z*), Li_2CuCl_4 (0.49 mL, 0.10 M in THF, 49 µmol, 0.20 equiv) in THF (3.9 mL) according to the general procedure **A**. The crude product was purified by flash column chromatography (SiO_2; 60 Å, 15–40 µm, PhMe:pentane; 4:1) to obtain diene **7** as a colourless oil (24.5 mg, 30%, >95:5 *E:Z*).

^{1}H NMR (400 MHz, $CDCl_3$): δ 7.29 – 7.23 (m, 2H), 7.09 (d, *J* = 8.6 Hz, 2H), 6.88 – 6.78 (m, 4H), 6.33 (dd, *J* = 15.9, 1.6 Hz, 1H), 6.05 (dt, *J* = 15.8, 6.8 Hz, 1H), 5.26 – 5.14 (m, 1H), 3.80 (s, 3H), 3.78 (s, 3H), 2.58 (dd, *J* = 8.7, 6.7 Hz, 2H), 2.32 – 2.23 (m, 4H), 2.16 – 2.08 (m, 2H), 1.58 (s, 3H).

^{13}C NMR (151 MHz, $CDCl_3$): δ 158.8, 157.8, 135.3, 134.6, 130.9, 129.5 (2C), 129.3, 128.7, 127.1 (2C), 124.3, 114.1 (2C), 113.8 (2C), 55.4 (2C), 39.7, 35.3, 31.8, 30.3, 16.2.

IR (film) ν_{max}: 2931, 2835, 1609, 1511, 1246, 1176, 1036, 828 cm^{-1}.

HRMS (ESI): calculated for $C_{23}H_{29}O_2$ $[M+H]^+$ requires m/z 337.2162, found m/z 337.2161 (Δ = -0.33 ppm).

1-Methoxy-4-((1E,5E)-5-methyl-8-(p-tolyl)octa-1,5-dien-1-yl)benzene 8.

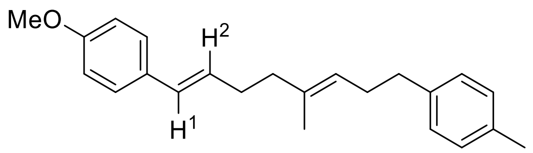

8
$C_{23}H_{28}O$
320.48 g mol^{-1}
$^{3}J_{H\text{-}1,\,H\text{-}2}$ = 15.7 Hz

4-Methylbenzyl chloride (0.65 mL, 4.9 mmol, 9.0 equiv) was used to prepare the Grignard reagent which was reacted with (*E*/*Z*)-**S3** (0.150 g, 0.548 mmol, 1.00 equiv, 94:6 *E*:*Z*), Li_2CuCl_4 (1.10 mL, 0.10 M in THF, 0.110 mmol, 0.200 equiv) in THF (8.8 mL) according to the general procedure **A.** The crude product was purified by flash column chromatography (SiO_2; 60 Å, 15–40 µm, pentane:Et_2O; 49:1) to obtain diene **8** as a colourless oil (0.120 g, 68%, >95:5 *E:Z*).

^{1}H NMR (400 MHz, $CDCl_3$): δ 7.30 – 7.24 (m, 2H), 7.11 – 7.06 (m, 4H), 6.84 (d, *J* = 8.7 Hz, 2H), 6.33 (dt, *J* = 15.7, 1.4 Hz, 1H), 6.06 (dt, *J* = 15.7, 6.8 Hz, 1H), 5.23 (tq, *J* = 7.1, 1.3 Hz, 1H), 3.80 (s, 3H), 2.60 (dd, *J* = 8.9, 6.7 Hz, 2H), 2.36 – 2.24 (m, 7H), 2.13 (dd, *J* = 8.9, 6.2 Hz, 2H), 1.60 (s, 3H).

^{13}C NMR (101 MHz, $CDCl_3$): δ 158.8, 139.4, 135.3, 135.2, 130.9, 129.3, 129.0 (2C), 128.7, 128.5 (2C), 127.1 (2C), 124.3, 114.0 (2C), 55.4, 39.7, 35.8, 31.8, 30.2, 21.2, 16.2.

IR (film) ν_{max}: 3029, 2360, 1608, 1511, 1248, 1175, 1037, 764 cm^{-1}.

HRMS (ESI): calculated for $C_{23}H_{28}ONa$ $[M+Na]^+$ requires m/z 343.2032, found m/z 343.2031 (Δ = -0.44 ppm).

1,4-Dimethoxy-2-((3*E*,7*E*)-8-(4-methoxyphenyl)-4-methylocta-3,7-dien-1-yl)benzene 9.

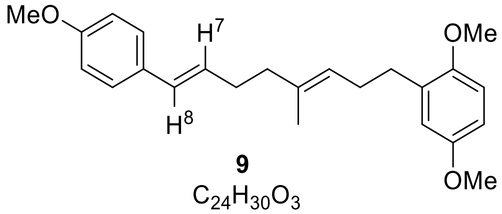

9
$C_{24}H_{30}O_3$
366.50 g mol^{-1}
$^3J_{\text{H-7, H-8}}$ = 15.7 Hz

2,5-Dimethoxybenzyl chloride (0.92 g, 4.9 mmol, 9.0 equiv) was used to prepare the Grignard reagent which was reacted with (*E*/*Z*)-**S3** (0.150 g, 0.548 mmol, 1.00 equiv, 94:6 *E*:*Z*), Li_2CuCl_4 (1.10 mL, 0.10 M in THF, 0.110 mmol, 0.200 equiv) in THF (8.8 mL) according to the general procedure **A.** The crude product was purified by flash column chromatography (SiO_2; 60 Å, 15–40 µm, pentane:Et_2O; 47:3) to obtain diene **9** as a colourless oil (0.143 g, 71%, 95:5 *E:Z*).

^{1}H NMR (400 MHz, $CDCl_3$): δ 7.29 – 7.24 (m, 2H), 6.86 – 6.80 (m, 2H), 6.79 – 6.66 (m, 3H), 6.33 (dt, *J* = 15.6, 1.5 Hz, 1H), 6.07 (dt, *J* = 15.7, 6.8 Hz, 1H), 5.29 – 5.23 (m, 1H), 3.80 (s, 3H), 3.78 (s, 3H), 3.76 (s, 3H), 2.66 – 2.55 (m, 2H), 2.33 – 2.22 (m, 4H), 2.13 (dd, *J* = 9.1, 6.2 Hz, 2H), 1.61 (s, 3H).

^{13}C NMR (101 MHz, $CDCl_3$): δ 158.8, 153.6, 152.0, 135.2, 132.2, 130.9, 129.2, 128.8, 127.1 (2C), 124.6, 116.5, 114.0 (2C), 111.3, 110.9, 56.1, 55.8, 55.4, 39.8, 31.9, 30.8, 28.4, 16.1.

IR (film) ν_{max}: 2933, 1608, 1510, 1500, 1464, 1247, 1223, 1176, 1049, 1035, 800 cm^{-1}.

HRMS (ESI): calculated for $C_{24}H_{30}O_3Na$ $[M+Na]^+$ requires m/z 389.2087, found m/z 389.2088 (*Δ* = 0.30 ppm).

1-Chloro-4-((3*E*,7*E*)-8-(4-methoxyphenyl)-4-methylocta-3,7-dien-1-yl)benzene 10.

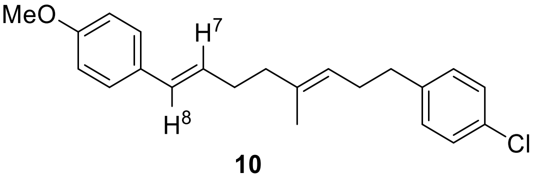

10
$C_{22}H_{25}OCl$
340.89 g mol^{-1}
$^3J_{\text{H-7, H-8}}$ = 15.7 Hz

4-Chlorobenzyl chloride (0.79 g, 4.9 mmol, 9.0 equiv) was used to prepare the Grignard reagent which was reacted with (*E*/*Z*)-**S3** (0.150 g, 0.548 mmol, 1.00 equiv, 94:6 *E*:*Z*), Li_2CuCl_4 (1.10 mL, 0.10 M in THF, 0.110 mmol, 0.200 equiv) in THF (8.8 mL) according to the general

procedure **A**. The crude product was purified by flash column chromatography (SiO_2; pentane:Et_2O; 49:1) to obtain diene **10** as a colourless oil (0.154 g, 82%, >95:5 *E:Z*).

^{1}H NMR (400 MHz, $CDCl_3$): δ 7.29 – 7.18 (m, 4H), 7.13 – 7.05 (m, 2H), 6.87 – 6.80 (m, 2H), 6.32 (dt, *J* = 15.9, 1.6 Hz, 1H), 6.04 (dt, *J* = 15.7, 6.8 Hz, 1H), 5.18 (tq, *J* = 7.2, 1.4 Hz, 1H), 3.80 (s, 3H), 2.60 (dd, *J* = 8.4, 6.9 Hz, 2H), 2.34 – 2.22 (m, 4H), 2.12 (dd, *J* = 8.8, 6.3 Hz, 2H), 1.57 (s, 3H).

^{13}C NMR (101 MHz, $CDCl_3$): δ 158.8, 140.8, 135.8, 131.5, 130.9, 130.0 (2C), 129.3, 128.6, 128.4 (2C), 127.1 (2C), 123.7, 114.1 (2C), 55.4, 39.7, 35.5, 31.7, 29.8, 16.2.

M.p. 43 °C

IR (film) ν_{max}: 2931, 2361, 2341, 1608, 1510, 1492, 1248, 1175, 1091, 1036, 965, 818 cm^{-1}.

HRMS: A stable molecular ion could not be detected by ESI, APCI or EI.

(2*E*,6*E*)-7-(4-Methoxyphenyl)-3-methylhepta-2,6-dien-1-yl acetate *E*-S3 and (2*E*,6*Z*)-7-(4-methoxyphenyl)-3-methylhepta-2,6-dien-1-yl acetate *Z*-S3.

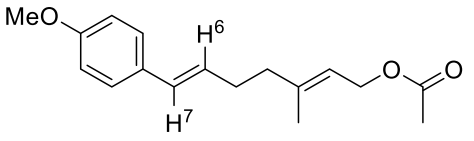

E-**S3** (Major)
$C_{17}H_{22}O_3$
274.36 g mol^{-1}
$^3J_{H\text{-}6,\,H\text{-}7}$ = 15.8 Hz

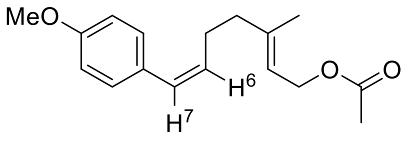

Z-**S3** (Minor)
$C_{17}H_{22}O_3$
274.36 g mol^{-1}
$^3J_{H\text{-}6,\,H\text{-}7}$ = 11.6 Hz

(4-Methoxybenzyl)(triphenyl)phosphonium chloride (0.628 g, 1.50 mmol, 1.50 equiv) was suspended in THF (5 mL) and cooled to -78 °C. *n*BuLi (0.600 mL, 2.5 M in hexanes, 1.50 mmol, 1.50 equiv) was then added dropwise and the obtained suspension was allowed to stir at -78 °C for 30 min. Aldehyde **S1** (0.171 g, 1.00 mmol, 1.00 equiv) in THF (1.5 mL) was added dropwise at the same temperature. The reaction mixture was allowed to warm to rt overnight and then quenched with sat. aq. NH_4Cl (10 mL). Mixture was diluted with water (5 mL) and extracted with Et_2O (3 × 15 mL). The organic layers were combined, washed with brine and volatiles were removed *in vacuo.* The crude product was purified by flash column chromatography (SiO_2, pentane:Et_2O; 9:1) to furnish an inseparable mixture of alkenes *E*-**S3** and *Z*-**S3** as a colourless oil (0.138 g, 50%, 77:23 *E*:*Z*).

Major *E*-S3 (from the mixture)**:**

^{1}H NMR (400 MHz, $CDCl_3$): δ 7.26 (d, *J* = 8.7 Hz, 2H), 6.83 (d, *J* = 8.7 Hz, 2H), 6.33 (d, *J* = 15.8 Hz, 1H), 6.04 (dt, *J* = 15.8, 6.8 Hz, 1H), 5.39 (tq, *J* = 7.1, 1.3 Hz, 1H), 4.59 (d, *J* = 7.1 Hz, 2H), 3.80 (s, 3H), 2.36 – 2.28 (m, 2H), 2.24 – 2.15 (m, 2H), 2.04 (s, 3H), 1.74 (3, 3H).

^{13}C NMR (101 MHz, $CDCl_3$): δ 171.2, 158.9, 141.9, 130.7, 129.7, 128.0, 127.2 (2C), 118.9, 114.1 (2C), 61.5, 55.4, 39.6, 31.3, 21.2, 16.6.

Characteristic peaks for the minor *Z*-S3 (from the mixture):

^{1}H NMR (400 MHz, $CDCl_3$): δ 7.21 (d, J = 8.7 Hz, 2H), 6.87 (d, *J* = 8.7 Hz, 2H), 5.53 (dt, *J* = 11.6, 7.2, Hz, 1H), 3.81 (s, 3H), 2.50 – 2.42 (m, 2H), 2.05 (s, 3H), 1.69 (s, 3H).

^{13}C NMR (101 MHz, $CDCl_3$): δ 141.7, 130.4, 130.0, 128.8, 113.7, 39.7, 26.8.

The analytical data are consistent with the data for the previously reported mixture of *E*-**S3** and *Z*-**S3** mixture (94:6 *E*:*Z*).

1-Methoxy-4-((1*E*,5*E*)-5-methyl-8-phenylocta-1,5-dien-1-yl)benzene *E*-1 and 1-Methoxy-4-((1Z,5E)-5-methyl-8-phenylocta-1,5-dien-1-yl)benzene *Z*-1.

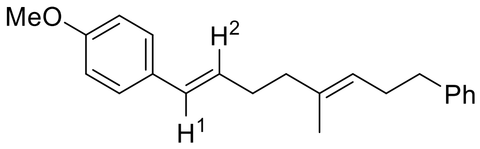

E-**1** (Major)
$C_{22}H_{26}O$
306.45 g mol^{-1}
$^3J_{H\text{-}1, H\text{-}2}$ = 15.8 Hz

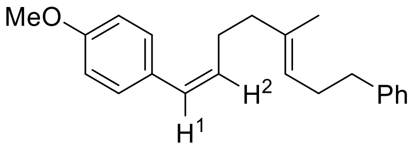

Z-**1** (Minor)
$C_{22}H_{26}O$
306.45 g mol^{-1}
$^3J_{H\text{-}1, H\text{-}2}$ = 11.7 Hz

Mixture of alkenes *E*-**S3** and *Z*-**S3** (0.120 g, 0.437 mmol, 1.00 equiv, 77:23 *E*:*Z*) was dissolved in THF (15 mL) and cooled to 0 °C. Li_2CuCl_4 (0.44 mL, 0.10 M in THF, 44 µmol, 0.10 equiv) was then added at the same temperature. To the obtained solution BnMgCl (0.66 mL, 2.0 M in THF, 1.3 mmol, 3.0 equiv) was added dropwise over 5 min keeping temperature at 0 °C. The reaction mixture was allowed to stir at the same temperature for 3 h, quenched with sat. aq. NH_4Cl (3 mL), diluted with water (10 mL) and extracted with Et_2O (3 × 10 mL). The organic layers were combined, washed with brine (30 mL) and volatiles were removed *in vacuo* to obtain the crude product which was purified by flash column chromatography (SiO_2, 60 Å, 15–40 µm**;** pentane:Et_2O; 49:1) to furnish compound *E*-**1** as a colourless oil which solidified on standing (44.3 mg, 33%, >95:5 *E:Z*) and *Z*-**1** as a colourless oil (7.0 mg, 5%,>95:5 *Z:E*).

Data for *E*-1:

^{1}H NMR (400 MHz, $CDCl_3$): δ 7.31 – 7.22 (m, 4H), 7.21 – 7.14 (m, 3H), 6.84 (d, *J* = 8.8 Hz, 2H), 6.33 (d, *J* = 15.8 Hz, 1H), 6.06 (dt, *J* = 15.8, 6.8 Hz, 1H), 5.27 – 5.29 (m, 1H), 3.80 (s, 3H), 2.64 (t, *J* = 7.5, 6.8 Hz, 2H), 2.37 – 2.23 (m, 4H), 2.17 – 2.08 (m, 2H), 1.58 (s, 3H).

^{13}C NMR (101 MHz, $CDCl_3$): δ 158.8, 142.5, 135.4, 130.9, 129.3, 128.7, 128.6 (2C), 128.4 (2C), 127.1 (2C), 125.8, 124.2, 114.1 (2C), 55.4, 39.7, 36.3, 31.8, 30.1, 16.2.

The analytical data for *E*-**1** are consistent with the previously reported for this compound obtained via a different method.

Data for *Z*-1:

^{1}H NMR (400 MHz, $CDCl_3$): δ 7.30 – 7.14 (m, 7H), 6.87 (d, *J* = 8.9 Hz, 2H), 6.34 (dt, *J* = 11.7, 2.0 Hz, 1H), 5.53 (dt, *J* = 11.7, 7.1 Hz, 1H), 5.21 (tq, *J* = 7.2, 1.4 Hz, 1H), 3.82 (s, 3H), 2.64 (t, *J* = 7.0 Hz, 2H), 2.41 (dtd, *J* = 8.3, 7.1, 1.9 Hz, 2H), 2.35 – 2.25 (m, 2H), 2.11 (t, *J* = 7.70 Hz, 2H), 1.54 (s, 3H).

^{13}C NMR (151 MHz, $CDCl_3$) δ 158.3, 142.5, 135.3, 131.1, 130.6, 130.1 (2C), 128.6 (2C), 128.4 (3C), 125.8, 124.3, 113.7 (2C), 55.4, 39.9, 36.2, 30.1, 27.2, 16.1.

IR (film) ν_{max}: 2917, 1608, 1510, 1247, 1176, 1026, 838, 698 cm^{-1}.

HRMS (ESI): calculated for $C_{22}H_{27}O$ $[M+H]^+$ requires m/z 307.2056, found m/z 307.2057 (Δ = 0.35 ppm).

*4.2 Synthesis of **11**.*

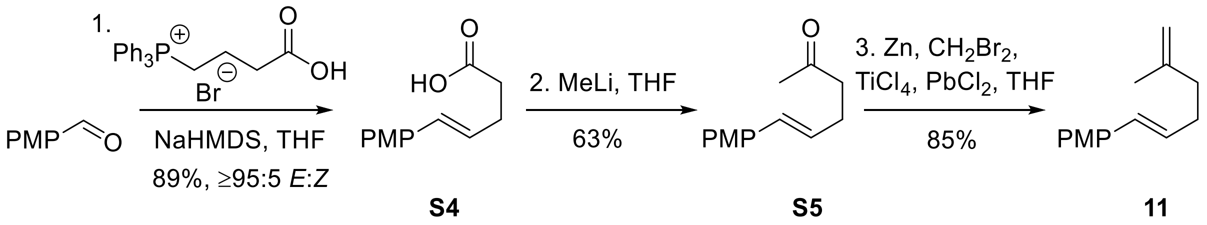

(*E*)-5-(4-Methoxyphenyl)pent-4-enoic acid S4.

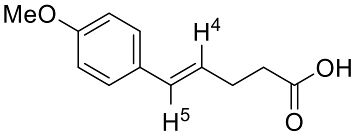

S4
$C_{12}H_{14}O_3$
206.24 g mol^{-1}
^3J $_{H\text{-}4,\,H\text{-}5}$ = 15.9 Hz

According to a literature procedure,[2] to a solution of (3-carboxypropyl)triphenylphosphonium bromide (5.67 g, 13.2 mmol, 1.19 equiv) in THF (130 mL) NaHMDS (13.3 mL, 2.00 M in THF, 26.6 mmol, 2.40 equiv) was added dropwise at 0 °C. The solution was stirred for 30 min at the same temperature, then cooled to -78 °C. 4-Methoxybenzaldehyde (1.35 mL, 11.1 mmol, 1.00 equiv) was added dropwise and the reaction was allowed to warm to rt overnight. Water (70 mL) and Et_2O (70 mL) were added. The aqueous layer was separated, acidified with 1.0 M aq. HCl to pH = 1 and extracted with EtOAc (3 × 100 mL). The combined organic layers were dried over Na_2SO_4, filtered and concentrated *in vacuo*. The crude product was washed with pentane (3 × 20 mL) to afford carboxylic acid **S4** as a white solid (2.03 g, 89%).

^{1}H NMR (400 MHz, $CDCl_3$): δ 7.28 (d, *J* = 8.8 Hz, 2H), 6.84 (d, *J* = 8.8 Hz, 2H), 6.39 (d, *J* = 15.9 Hz, 1H), 6.12 – 5.99 (m, 1H), 3.80 (s, 3H), 2.55 – 2.50 (m, 4H).

^{13}C NMR (101 MHz, $CDCl_3$): δ 179.0, 159.1, 130.7, 130.3, 127.4 (2C), 126.0, 114.1 (2C), 55.4, 33.9, 28.1.

The analytical data are consistent with those previously reported in the literature.[2]

(*E*)-6-(4-Methoxyphenyl)hex-5-en-2-one S5.

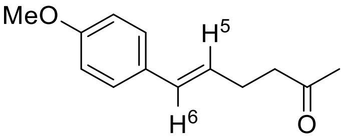

S5
$C_{13}H_{16}O_2$
204.27 g mol^{-1}
^3J $_{H\text{-}5,\,H\text{-}6}$ = 15.8 Hz

Carboxylic acid **S4** (2.43 g, 11.8 mmol, 1.00 equiv) was suspended in THF (58 mL) and cooled to 0 °C. MeLi (19 mL, 1.6 M in Et_2O, 30 mmol**,** 2.5 equiv) was then added dropwise at the

[2] I. Colomer, R. C. Barcelos, K. E. Christensen, T. J. Donohoe, *Org. Lett.* **2016**, *18*, 5880–5883.

same temperature and the obtained suspension was allowed to warm to rt overnight. Mixture was poured into the solution of conc. HCl (3.5 mL) in water (110 mL) and extracted with Et_2O (3 × 100 mL). The organic layers were combined, washed with brine and dried over Na_2SO_4. Volatiles were removed *in vacuo* and the crude product was purified by flash column chromatography (SiO_2; pentane:Et_2O; 3:2) to furnish the ketone **S5** as a yellow oil (1.51 g, 63%).

^{1}H NMR (400 MHz, $CDCl_3$): δ 7.26 (d, J = 8.8 Hz, 2H), 6.83 (d, J = 8.8 Hz, 2H), 6.35 (d, J = 15.8 Hz, 1H), 6.04 (dt, J = 15.8, 6.8 Hz, 1H), 3.80 (s, 3H), 2.64 – 2.56 (m, 2H), 2.50 – 2.41 (m, 2H), 2.17 (s, 3H).

^{13}C NMR (101 MHz, $CDCl_3$): δ 208.4, 159.0, 130.4, 130.3, 127.3 (2C), 126.7, 114.1 (2C), 55.4, 43.5, 30.2, 27.3.

The analytical data are consistent with those previously reported in the literature.[3]

(*E*)-1-Methoxy-4-(5-methylhexa-1,5-dien-1-yl)benzene 11.

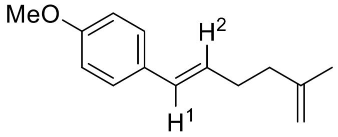

11
$C_{14}H_{18}O$
202.30 g mol^{-1}
$^3J_{\text{H-1, H-2}}$ = 15.8 Hz

According to a literature procedure,[4] zinc (3.43 g, 52.5 mmol, 25.0 equiv) and $PbCl_2$ (cat) were weighed into a round bottom flask which was then subjected to three evacuate-refill cycles with N_2. THF (35 mL) and CH_2Br_2 (1.21 mL,17.4 mmol, 8.29 equiv) were then added and the obtained suspension was cooled to -25 °C. $TiCl_4$ (1.10 mL, 10.0 mmol, 4.76 equiv) was then added dropwise and the mixture was allowed to warm to -10 °C and then stirred at the same temperature for 2 h. Ketone **S5** (0.429 g, 2.10 mmol, 1.00 equiv) dissolved in THF (12 mL) was added dropwise and the reaction mixture was warmed to 0 °C and stirred at the same temperature for 0.5 h. Mixture was poured onto ice cold aq. HCl (1 M, 12 mL), diluted with water (10 mL) and extracted with Et_2O (3 × 20 mL). The organic layers were combined, washed with brine and dried over Na_2SO_4. Volatiles were removed *in vacuo* and the crude

[3] P. Calleja, Ó. Pablo, B. Ranieri, M.Gaydou, A. Pitaval, M. Moreno, M. Raducan, A. M. Echavarren, *Chem. Eur. J.* **2016**, *22*, 13613–13618.
[4] L. Lombardo, *Tetrahedron Lett.* **1982**, *23*, 4293–4296.

product was purified by flash column chromatography (SiO_2; pentane:Et_2O = 9:1) to furnish alkene **11** as a colourless oil (0.361 g, 85%).

^{1}H NMR (400 MHz, $CDCl_3$): δ 7.27 (d, *J* = 8.8 Hz, 2H), 6.83 (d, *J* = 8.8 Hz, 2H), 6.35 (dt, *J* = 15.8, 1.5 Hz, 1H), 6.08 (dt, *J* = 15.8, 6.8 Hz, 1H), 4.78 – 4.69 (m, 2H), 3.80 (s, 3H), 2.38 – 2.29 (m, 2H), 2.21 – 2.14 (m, 2H), 1.76 (s, 3H).

^{13}C NMR (101 MHz, $CDCl_3$): δ 158.8, 145.5, 130.8, 129.4, 128.4, 127.2 (2C), 114.1 (2C), 110.3, 55.4, 37.8, 31.3, 22.7.

The analytical data are consistent with those previously reported in the literature.[5]

*4.3 Synthesis of diene **3**.*

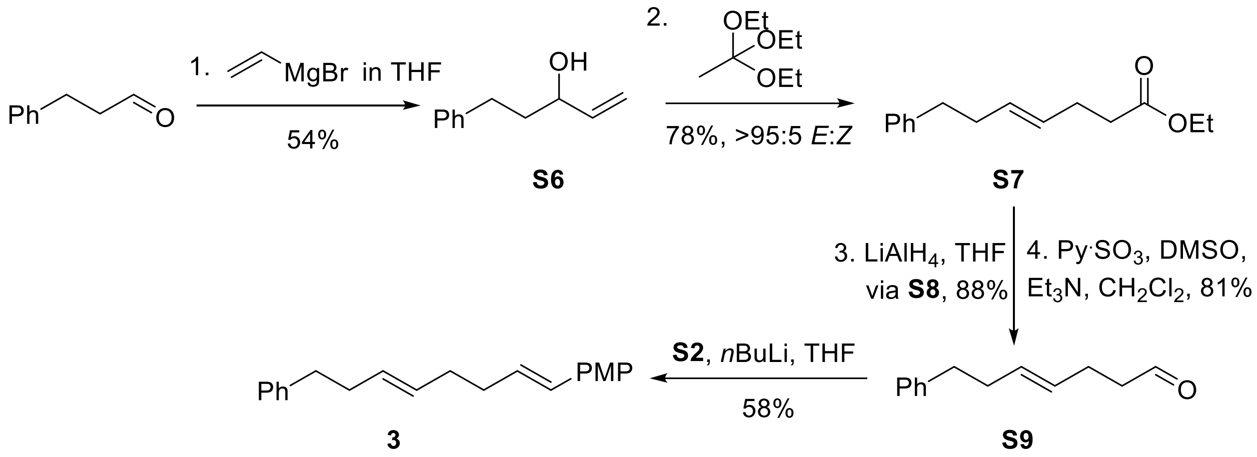

5-Phenylpent-1-en-3-ol S6.

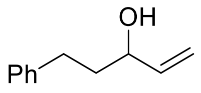

S6
$C_{11}H_{14}O$
162.23 g mol^{-1}

According to a literature procedure,[6] 3-phenylpropanal (1.33 mL, 10.1 mmol, 1.00 equiv) in Et_2O (10 mL) was added dropwise to the cooled to -78 °C solution of vinyl magnesium bromide (12 mL, 1.0 M in THF, 12 mmol, 1.2 equiv). The reaction mixture was stirred at the same temperature for 3 h and then at rt for 0.5 h. The solution was quenched with aq. sat. NH_4Cl (5 mL) and diluted with water (15 mL). The organic layer was separated and the aqueous layer

[5] Y. Sumida, S. Hayashi, K. Hirano, H. Yorimitsu, K. Oshima, *Org. Lett.* **2008**, *10*, 1629–1632.

was extracted with Et_2O (3 × 10 mL). The organic layers were combined, washed with brine, dried over anhydrous Na_2SO_4 and concentrated *in vacuo*. The crude product was purified by flash column chromatography (SiO_2; pentane:Et_2O; 7:3) to furnish allylic alcohol **S6** as a pale yellow oil (0.882 g, 54%).

^{1}H NMR (400 MHz, $CDCl_3$): δ 7.35 – 7.24 (m, 2H), 7.24 – 7.15 (m, 3H), 5.91 (ddd, *J* = 16.8, 10.4, 6.2 Hz, 1H), 5.25 (dt, *J* = 17.2, 1.4 Hz, 1H), 5.14 (dt, *J* = 10.4, 1.4 Hz, 1H), 4.19 – 4.08 (m, 1H), 2.84 – 2.63 (m, 2H), 1.94 – 1.77 (m, 2H).

^{13}C NMR (101 MHz, $CDCl_3$): δ 142.0, 141.1, 128.6 (4C), 126.0, 115.1, 72.6, 38.7, 31.8.

The analytical data are consistent with those previously reported in the literature.[6]

Ethyl (*E*)-7-phenylhept-4-enoate S7.

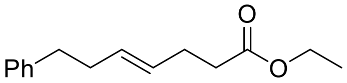

S7
$C_{15}H_{20}O_2$
232.32 g mol^{-1}

According to a literature procedure,[7] a solution of the allylic alcohol **S6** (0.882 g, 5.44 mmol, 1.00 equiv) and propionic acid (31 µL) in triethyl orthoacetate (13 mL) was heated with stirring at 145 °C for 24 h. The solution was cooled to room temperature, poured into water (10 mL) and extracted with Et_2O (3 × 15 mL). The organic layers were combined, washed successively with aq. HCl (1 M, 2 × 30 mL) and brine (30 mL) and dried over anhydrous Na_2SO_4. Volatiles were removed *in vacuo* and the crude product was purified by flash column chromatography (SiO_2; pentane:Et_2O; 19:1) to furnish ester **S7** as a colourless oil (1.05 g, 78%).

^{1}H NMR (400 MHz, $CDCl_3$): δ 7.32 – 7.24 (m, 2H), 7.22 – 7.13 (m, 3H), 5.59 – 5.36 (m, 2H), 4.13 (q, *J* = 7.1 Hz, 2H), 2.70 – 2.61 (m, 2H), 2.40 – 2.25 (m, 6H), 1.25 (t, *J* = 7.2 Hz, 3H).

^{13}C NMR (101 MHz, $CDCl_3$): δ 173.4, 142.1, 130.9, 128.9, 128.6 (2C), 128.4 (2C), 125.9, 60.4, 36.1, 34.5 (2C), 28.0, 14.4.

The analytical data are consistent with those previously reported in the literature.[7]

---

[6] M. Lafrance, M. Roggen, E. M. Carreira, *Angew. Chem. Int. Ed.* **2012**, *51,* 3470–3473.

[7] C. U. Grunanger, B. Breit, *Angew. Chem. Int. Ed.* **2010**, *49,* 967–970.

(*E*)-4-Methyl-7-phenylhept-4-en-1-ol S8.

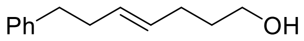

S8
$C_{13}H_{18}O$
190.29 g mol^{-1}

According to a literature procedure,[8] ester **S7** (0.500 g, 2.15 mmol, 1.00 equiv) in THF (5 mL) was added dropwise to the suspension of $LiAlH_4$ (81.6 mg, 2.15 mmol, 1.00 equiv) in THF (10 mL) at 0 °C. The reaction mixture was stirred at the same temperature for 3 h followed by the subsequent addition of water (82 µL), 15% aq. NaOH (82 µL) and water (0.25 mL). The obtained suspension was stirred at rt for 30 min, transferred to a separatory funnel, diluted with an additional amount of water (15 mL) and extracted with Et_2O (3 × 15 mL). The organic layers were combined, washed with brine and dried over anhydrous Na_2SO_4. Volatiles were removed *in vacuo* and the crude product was purified by flash column chromatography (SiO_2; pentane:Et_2O; 1:1) to furnish alcohol **S8** as a colourless oil (0.361 g, 88%).

^{1}H NMR (400 MHz, $CDCl_3$): δ 7.31 – 7.24 (m, 2H), 7.22 – 7.12 (m, 3H), 5.59 – 5.35 (m, 2H), 3.61 (t, *J* = 6.5 Hz, 2H), 2.70 – 2.62 (m, 2H), 2.37 – 2.24 (m, 2H), 2.12 – 2.01 (m, 2H), 1.66 – 1.56 (m, 2H).

^{13}C NMR (101 MHz, $CDCl_3$): δ 142.2, 130.4, 130.3, 128.6 (2C), 128.4 (2C), 125.9, 62.7, 36.2, 34.5, 32.5, 29.0.

The analytical data are consistent with those previously reported in the literature.[8]

(*E*)-7-Phenylhept-4-enal S9.

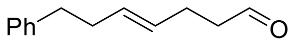

S9
$C_{13}H_{16}O$
188.27 g mol^{-1}

Alcohol **S8** (1.58 g, 8.30 mmol, 1.00 equiv) was dissolved in a mixture of CH_2Cl_2 (16 mL), DMSO (16 mL) and Et_3N (5.7 mL) at 0 °C. To the obtained solution $SO_3 \cdot$ py complex (3.77 g, 23.7 mmol, 2.86 equiv) was added in one portion. Reaction mixture was allowed to stir at 0 °C for 2.5 h and then diluted with water (20 mL). Organic phase was separated, and aqueous phase was extracted with CH_2Cl_2 (3 × 20 mL). Organic layers were combined, washed with brine and dried over anhydrous Na_2SO_4. Volatiles were removed *in vacuo* and the crude

[8] C. U. Grunanger, B. Breit, *Angew. Chem. Int. Ed.* **2010**, *49,* 967–970.

product was purified by flash column chromatography (SiO_2; pentane:Et_2O; 22:3) to furnish aldehyde **S9** as a colourless oil (1.26 g, 81%).

^{1}H NMR (400 MHz, $CDCl_3$): δ 9.74 (t, *J* = 1.7 Hz, 1H), 7.31 – 7.23 (m, 2H), 7.21 – 7.12 (m, 3H), 5.58 – 5.36 (m, 2H), 2.66 (dd, *J* = 8.8, 6.6 Hz, 2H), 2.51 – 2.42 (m, 2H), 2.37 – 2.25 (m, 4H).

^{13}C NMR (101 MHz, $CDCl_3$): δ 202.5, 142.0, 131.1, 128.7, 128.6 (2C), 128.4 (2C), 125.9, 43.6, 36.0, 34.4, 25.3.

IR (film) ν_{max}: 2922, 1725, 1496, 1454, 1276, 970, 749, 700 cm^{-1}.

HRMS (APCI): calculated for $C_{13}H_{17}O$ $[M+H]^+$ requires m/z 189.1274, found m/z 189.1274 (Δ= -0.18 ppm).

1-Methoxy-4-((1*E*,5E)-8-phenylocta-1,5-dien-1-yl)benzene 3.

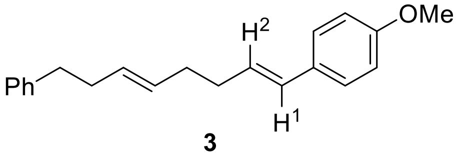

3
$C_{21}H_{24}O$
292.42 g mol^{-1}
$^3J_{H-1, H-2}$ = 15.8 Hz

Tributyl(4-methoxybenzyl)phosphonium chloride **S2** (0.400 g, 1.11 mmol, 1.50 equiv) was suspended in THF (16 mL) and cooled to 0 °C. *n*BuLi (0.44 mL, 2.5 M in hexanes, 1.1 mmol, 1.5 equiv) was then added dropwise and the obtained suspension was allowed to stir at 0 °C for 30 min. After that mixture was cooled to -78 °C and aldehyde **S9** (0.140 g, 0.74 mmol, 1.00 equiv) in THF (1.4 mL) was added dropwise at the same temperature. The reaction mixture was allowed to warm to rt overnight and then quenched with sat. aq. NH_4Cl (5 mL). Mixture was diluted with water (10 mL) and extracted with Et_2O (3 × 15 mL). The organic layers were combined, washed with brine and volatiles were removed *in vacuo.* The crude product was purified by flash column chromatography (SiO_2, pentane:Et_2O; 49:1) to furnish diene **3** as a white solid (0.126 g, 58%, >95:5 *E*:*Z*).

^{1}H NMR (400 MHz, $CDCl_3$): δ 7.31 – 7.24 (m, 4H), 7.21 – 7.14 (m, 3H), 6.84 (d, *J* = 8.7 Hz, 2H), 6.32 (d, *J* = 15.8 Hz, 1H), 6.06 (dt, *J* = 15.8, 6.7 Hz, 1H), 5.55 – 5.43 (m, 2H), 3.80 (s, 3H), 2.67 (dd, *J* = 8.9, 6.6 Hz, 2H), 2.36 – 2.28 (m, 2H), 2.27 – 2.19 (m, 2H), 2.18 – 2.10 (m, 2H).

^{13}C NMR (101 MHz, $CDCl_3$): δ 158.8, 142.3, 130.8, 130.4, 130.1, 129.5, 128.6 (2C), 128.4 (3C), 127.1 (2C), 125.8, 114.0 (2C), 55.4, 36.2, 34.6, 33.2, 32.7.

IR (film) ν_{max}: 2915, 2360, 1608, 1511, 1454, 1248, 1175, 1036, 966, 909, 747, 699 cm^{-1}.

M.p. 45-47 °C

HRMS (ESI): calculated for $C_{21}H_{25}O$ $[M+H]^+$ requires m/z 293.1900, found m/z 293.1900 (*Δ* = 0.03 ppm).

*4.4 Synthesis of E/Z-**4** and E/Z-**5**.*

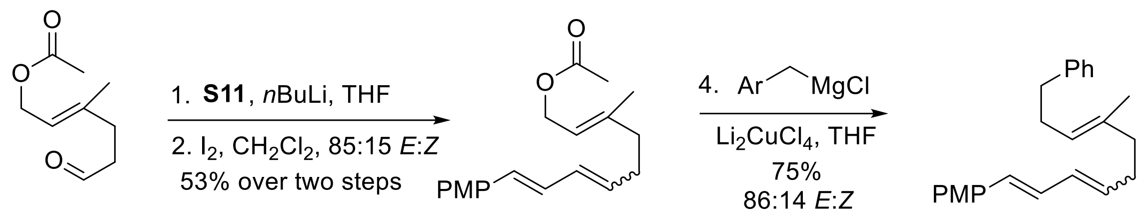

S1 *E*/*Z*-**S10** *E*/*Z*-**4**

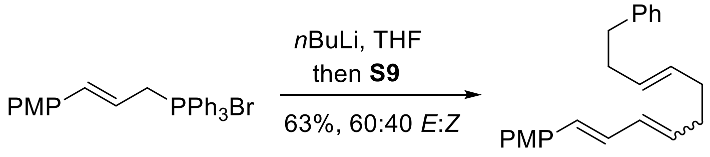

S11 *E*/*Z*-**5**

(*E*)-(3-(4-Methoxyphenyl)allyl)triphenylphosphonium bromide S11.

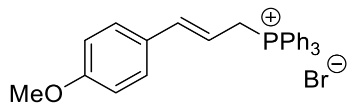

S11
$C_{28}H_{26}OPBr$
489.39 g mol^{-1}

(*E*)-3-(4-Methoxyphenyl)prop-2-en-1-ol (3.00 g, 18.3 mmol, 1.00 equiv) and triphenylphosphine hydrobromide (6.14 g, 17.9 mmol, 0.98 eq) were combined in MeCN (52 mL) and the obtained solution was heated under reflux overnight. The reaction mixture was allowed to cool to rt overnight and volatiles were removed *in vacuo.* The residue was washed with pentane (3 × 20 mL) and dried under vacuum. The obtained yellow solid **S11** was used in the next step without purification (8.80 g, quant).[9]

[9] Product is impure but sufficient yields (53-63%) were achieved in the next step using unpurified **S11**. Recrystallisation (Et_2O, CH_2Cl_2, -20 °C, **34%**) was performed to obtain clean sample of salt **S11**, however, it did not improve the yield of the following Wittig reaction.

^{1}H NMR (400 MHz, $CDCl_3$): δ 7.88 – 7.55 (m, 15H), 7.16 – 7.04 (m, 2H), 6.80 – 6.60 (m, 3H), 5.84 – 5.73 (m, 1H), 4.99 – 4.69 (m, 2H), 3.73 (s, 3H).

^{13}C NMR (101 MHz, $CDCl_3$): δ 159.9, 139.8 (d, *J* = 13.5 Hz), 135.1 (d, *J* = 3.0 Hz, 3C), 134.1 (d, *J* = 9.8 Hz, 6C), 132.2, 130.41 (d, *J* = 12.5 Hz, 6C), 127.9 (d, *J* = 2.3 Hz, 2C), 118.1 (d, *J* = 85.5 Hz, 3C), 114.1 (2C), 110.9 (d, *J* = 10.9 Hz), 55.4, 28.41 (d, *J* = 48.9 Hz).

^{31}P (162 MHz, $CDCl_3$): δ 21.1

HRMS (ESI): calculated for $C_{28}H_{26}OP$ $[M]^+$ requires m/z 409.1716, found m/z 409.716 (*Δ* = 0.05 ppm).

(2*E*,6*E*,8*E*)-9-(4-Methoxyphenyl)-3-methylnona-2,6,8-trien-1-yl acetate (*E*)-S10 and (2*E*,6*Z*,8*E*)-9-(4-Methoxyphenyl)-3-methylnona-2,6,8-trien-1-yl acetate (*Z*)-S10.

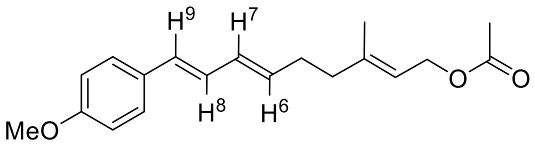

(*E*)-**S10**
$C_{19}H_{24}O_3$
300.40 g mol^{-1}
$^{3}J_{H\text{-}9,\,H\text{-}8}$ = 15.5 Hz
$^{3}J_{H\text{-}7,\,H\text{-}6}$ = 14.8 Hz

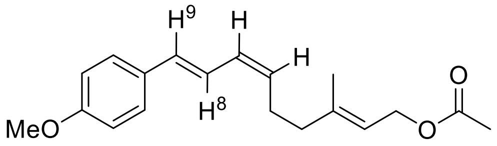

(*Z*)-**S10**
$C_{19}H_{24}O_3$
300.40 g mol^{-1}
$^{3}J_{H\text{-}9,\,H\text{-}8}$ = 15.6 Hz

(*E*)-(3-(4-Methoxyphenyl)allyl)triphenylphosphonium bromide (2.15 g, 4.39 mmol, 1.50 equiv) was suspended in THF (21 mL) and cooled to -78 °C. *n*BuLi (1.76 mL, 2.5 M in hexanes, 4.40 mmol, 1.50 equiv) was added dropwise and the obtained suspension was allowed to stir at the same temperature for 30 min. Aldehyde **S1** (0.497 g, 2.92 mmol, 1.00 equiv) in THF (10 mL) was added dropwise at -78 °C and the reaction mixture was allowed to warm to rt overnight. The reaction was quenched with sat. aq. NH_4Cl (7 mL), diluted with water (20 mL) and extracted with Et_2O (3 × 30 mL). The organic layers were combined, washed with brine and volatiles were removed *in vacuo.* The crude product was purified by flash column chromatography (SiO_2, pentane:Et_2O; 4:1) to furnish an inseparable mixture of alkenes (*E*)-**S10** and (*Z*)-**S10** as a pale yellow oil (0.482 g, 56:44 *E*:*Z*).

The mixture of alkenes (*E*)-**S10 and** (*Z*)-**S10** (0.482 g, 1.61 mmol, 1.00 equiv, 56:44 *E*:*Z*) was dissolved in CH_2Cl_2 (3.3 mL) and I_2 (20.5 mg, 80.8 µmol, 0.0502 equiv) was added in one portion. The solution was stirred at rt for 30 min, quenched with sat. aq. $Na_2S_2O_3$ (1 mL), diluted with water (5 mL) and extracted with CH_2Cl_2 (3 × 5 mL). Organic layers were combined, washed with brine and volatiles were removed *in vacuo* to obtain an inseparable mixture of

alkenes (*E*)-**S10 and** (*Z*)-**S10** as a yellow oil which did not require any further purification (0.469 g, 53% over two steps, 85:15 *E*:*Z*).[10]

Major (*E*)-S10 (from the mixture)**:**

^{1}H NMR (400 MHz, $CDCl_3$): δ 7.31 (d, *J* = 8.7 Hz, 2H), 6.84 (d, *J* = 8.7 Hz, 2H), 6.61 (dd, *J* = 15.5, 10.3 Hz, 1H), 6.40 (d, *J* = 15.7 Hz, 1H), 6.19 (ddt, *J* = 14.8, 10.3, 1.4 Hz, 1H), 5.73 (dt, *J* = 14.6, 6.9 Hz, 1H), 5.37 (tq, *J* = 7.0, 1.3 Hz, 1H), 4.59 (d, *J* = 7.1 Hz, 2H), 3.80 (s, 3H), 2.31 – 2.23 (m, 2H), 2.20 – 2.11 (m, 2H), 2.05 (s, 3H), 1.72 (s, 3H).

^{13}C NMR (101 MHz, $CDCl_3$): δ 171.3, 159.1, 141.8, 133.5, 131.2, 130.5, 130.1, 127.5 (2C), 127.4, 118.9, 114.2 (2C), 61.5, 55.4, 39.4, 31.1, 21.2, 16.6.

Characteristic peaks for minor (*Z*)-S10 (from the mixture)**:**

^{1}H NMR (400 MHz, $CDCl_3$): δ 7.35 (d, *J* = 8.7 Hz, 2H), 6.48 (d, *J* = 15.6 Hz, 1H), 3.81 (s, 3H), 2.46 – 2.37 (m, 2H), 2.05 (s, 3H), 1.74 (s, 3H).

^{13}C NMR (101 MHz, $CDCl_3$): δ 127.7, 122.4, 114.2, 26.3.

IR (film) ν_{max}: 2935, 1733, 1602, 1512, 1250, 1175, 1031, 911, 732 cm^{-1}.

HRMS (ESI): calculated for $C_{19}H_{25}O_3$ $[M+H]^+$ requires m/z 301.1798, found m/z 301.1795 (*Δ* = -1.0 ppm).

1-Methoxy-4-((1*E*,3*E*,7*E*)-7-methyl-10-phenyldeca-1,3,7-trien-1-yl)benzene (*E*)-4 and 1-Methoxy-4-((1E,3*Z*,7*E*)-7-methyl-10-phenyldeca-1,3,7-trien-1-yl)benzene (*Z*)-4.

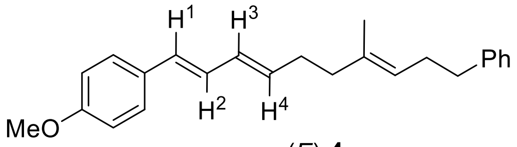

(*E*)-**4**
$C_{24}H_{28}O$
332.49 g mol^{-1}
$^{3}J_{H\text{-}1, H\text{-}2}$ = 15.6 Hz
$^{3}J_{H\text{-}3, H\text{-}4}$ = 15.1 Hz

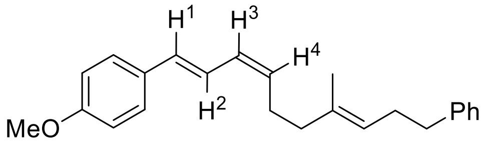

(*Z*)-**4**
$C_{24}H_{28}O$
332.49 g mol^{-1}
$^{3}J_{H\text{-}1, H\text{-}2}$ = 15.5 Hz
$^{3}J_{H\text{-}3, H\text{-}4}$ = 10.8 Hz

Mixture of alkenes *E*-**S10** and *Z*-**S10** (0.681 g, 2.27 mmol, 1.00 equiv, 85:15 *E*:*Z*) was dissolved in THF (35 mL) and cooled to 0 °C. Li_2CuCl_4 (6.80 mL, 0.10 M in THF, 0.680 mmol, 0.300 equiv) was then added at the same temperature. To the obtained solution BnMgCl (10.2 mL, 2.0 M in THF, 20.4 mmol, 8.99 equiv) was added dropwise over 5 min keeping

[10] E. E. Schultz, V. N. G. Lindsay, R. Sarpong, *Angew. Chem. Int. Ed.* **2014**, *53*, 9904 –9908.

temperature at 0 °C. The reaction mixture was allowed to stir at the same temperature for 3 h, quenched with sat. aq. NH_4Cl (10 mL), diluted with water (30 mL) and extracted with Et_2O (3 × 40 mL). The organic layers were combined, washed with brine (100 mL) and volatiles were removed *in vacuo* to obtain the crude product which was purified by flash column chromatography (SiO_2, 60 Å, 15–40 µm**;** pentane:Et_2O; 97:3) to furnish an inseparable mixture of trienes (*E*)-**4** and (*Z*)-**4** as a colourless waxy solid (0.565 g, 75%, 86:14 *E*:*Z*).

Major (*E*)-4 (from the mixture)**:**

^{1}H NMR (400 MHz, $CDCl_3$): δ 7.33 – 7.25 (m, 4H), 7.22 – 7.17 (m, 3H), 6.84 (d, *J* = 8.8 Hz, 2H), 6.62 (dd, *J* = 15.6, 10.3 Hz, 1H), 6.39 (d, *J* = 15.6 Hz, 1H), 6.18 (dd, *J* = 15.1, 10.3 Hz, 1H), 5.74 (dt, *J* = 15.1, 6.9 Hz, 1H), 5.24 – 5.17 (m, 1H), 3.81 (s, 3H), 2.65 (dd, *J* = 8.8, 6.7 Hz, 2H), 2.36 – 2.28 (m, 2H), 2.26 – 2.19 (m, 2H), 2.12 – 2.05 (m, 2H), 1.57 (s, 3H).

^{13}C NMR (101 MHz, $CDCl_3$): δ 159.1, 142.5, 135.3, 134.3, 130.9, 130.7, 129.8, 128.6 (2C), 128.4 (2C), 127.6, 127.4 (2C), 125.8, 124.2, 114.2 (2C), 55.4, 39.6, 36.2, 31.6, 30.0, 16.1.

Characteristic peaks for minor (*Z*)-4 (from the mixture)**:**

^{1}H NMR (400 MHz, $CDCl_3$): δ 7.35 (d, *J* = 8.8 Hz, 2H), 6.48 (d, *J* = 15.5 Hz, 1H), 5.43 (dt, J = 10.8, 7.5 Hz, 1H), 3.82 (s, 3H), 1.59 (s, 3H).

IR (film) ν_{max}: 2930, 1604, 1510, 1454, 1250, 1174, 1035, 986, 827, 748, 699 cm^{-1}.

HRMS (GC EI): calculated for $C_{24}H_{28}O$ $[M]^{\cdot+}$ requires 332.21347, found m/z 332.21309 (*Δ* = -1.1 ppm).

1-Methoxy-4-((1*E*,3*E*,7*E*)-10-phenyldeca-1,3,7-trien-1-yl)benzene (*E*)-5 and 1-Methoxy-4-((1*E*,3*Z*,7*E*)-10-phenyldeca-1,3,7-trien-1-yl)benzene (*Z*)-5.

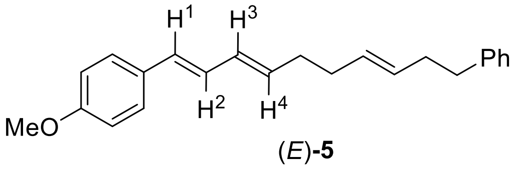

(*E*)-**5**
$C_{23}H_{26}O$
318.46 g mol^{-1}
$^3J_{H\text{-}1, H\text{-}2}$ = 15.6 Hz
$^3J_{H\text{-}3, H\text{-}4}$ = 15.0 Hz

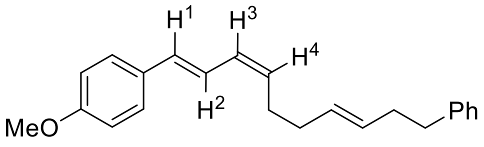

(*Z*)-**5**
$C_{23}H_{26}O$
318.46 g mol^{-1}
$^3J_{H\text{-}1, H\text{-}2}$ = 15.5 Hz

(*E*)-(3-(4-Methoxyphenyl)allyl)triphenylphosphonium bromide (0.778 g, 1.59 mmol, 1.50 equiv) was suspended in THF (7.4 mL) and cooled to -78 °C. *n*BuLi (0.64 mL, 2.5 M in hexanes, 1.6 mmol, 1.5 equiv) was added dropwise and the obtained suspension was allowed to stir at the same temperature for 30 min. Aldehyde **S9** (0.200 g, 1.06 mmol, 1.00 equiv) in

THF (3.8 mL) was added dropwise at -78 °C and the reaction mixture was allowed to warm to rt overnight. The reaction was quenched with sat. aq. NH_4Cl (3 mL), diluted with water (10 mL) and extracted with Et_2O (3 × 15 mL). The organic layers were combined, washed with brine and volatiles were removed *in vacuo.* The crude product was purified by flash column chromatography (SiO_2, pentane:Et_2O; 24:1) to furnish an inseparable mixture of alkenes (*E*)-**5** and (*Z*)-**5** as a colourless waxy solid (0.211 g, 63%, 60:40 *E*:*Z*).

^{1}H NMR (400 MHz, $CDCl_3$): δ 7.39 – 7.14 (m, 7H_{maj}, 7H_{min}), 6.97 – 6.82 (m, 2H_{maj}, 3H_{min}), 6.63 (dd, *J* = 15.6, 10.3 Hz, 1H_{maj}), 6.49 (d, *J* = 15.5 Hz, 1H_{min}), 6.40 (d, *J* = 15.6 Hz, 1H_{maj}), 6.24 – 6.11 (m, 1H_{maj}, 1H_{min}), 5.75 (dt, *J* = 15.0, 6.7 Hz, 1H_{maj}), 5.59 – 5.37 (m, 2H_{maj}, 3H_{min}), 3.87 – 3.76 (m, 3H_{maj}, 3H_{min}), 2.73 – 2.63 (m, 2H_{maj}, 2H_{min}), 2.39 – 2.27 (m, 2H_{maj}, 4H_{min}), 2.23 – 2.07 (m, 4H_{maj}, 2H_{min}).

^{13}C NMR (101 MHz, $CDCl_3$) **for the major diastereomer** (from the mixture): δ 159.1, 142.2, 134.0, 131.1, 130.6, 130.3, 130.2, 129.8, 128.6 (2C), 128.4 (2C), 127.7, 127.4 (2C), 125.8, 114.2 (2C), 55.4, 36.2, 34.5, 33.0, 32.6. **Selected peaks for the minor diastereomer** (from the mixture): δ 159.3, 131.8, 131.4, 130.3, 129.2, 127.6, 122.7, 114.2, 36.2, 34.6, 32.8.

IR (film) ν_{max}: 2932, 2361, 2341, 1604, 1510, 1251, 1175, 1034, 986, 909, 732, 699 cm^{-1}.

HRMS calculated for $C_{23}H_{27}O$ $[M+H]^+$ requires m/z 319.2056, found m/z 319.2056 (Δ = -0.14 ppm).

4.5 Synthesis of alcohols using modified literature procedures.

Methyl 2-hydroxy-5-(1-hydroxyethyl)benzoate S12.

S12
$C_{10}H_{12}O_4$
196.20 g mol^{-1}

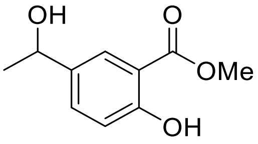

Methyl 5-acetyl-2-hydroxybenzoate (0.300 g, 1.54 mmol, 1.00 equiv) was dissolved in MeOH (5 mL) and cooled to 0 °C. To the obtained solution $NaBH_4$ (0.117 g, 3.09 mmol, 2.00 equiv) was added portionwise and the obtained mixture was allowed to stir at 0 °C for 2 h. Water (10 mL) and Et_2O (10 mL) were then added. The organic layer was separated and the aqueous layer was extracted with Et_2O (3 X 10 mL). The organic layers were combined, washed with brine and dried over Na_2SO_4. Volatiles were removed *in vacuo* to obtain methyl 2-hydroxy-5-(1-hydroxyethyl)benzoate **S12** as a colourless oil which did not require any further purification (0.271 g, 90%).

^{1}**H NMR** (400 MHz, $CDCl_3$): δ 10.70 (s, 1H), 7.85 (d, *J* = 2.1 Hz, 1H), 7.48 (dd, *J* = 8.6, 2.3 Hz, 1H), 6.97 (dd, *J* = 8.6, 1.2 Hz, 1H), 4.94 – 4.81 (m, 1H), 3.95 (s, 3H), 1.84 – 1.73 (br m, 1H), 1.48 (d, *J* = 6.4 Hz, 3H).

^{13}**C NMR** (101 MHz, $CDCl_3$): δ 170.6, 161.1, 136.7, 133.3, 126.8, 117.9, 112.2, 69.8, 52.4, 25.3.

The analytical data are consistent with those previously reported in the literature.[11]

1-(4-Chlorophenyl)cyclopent-2-en-1-ol S13.

S13
$C_{11}H_{11}ClO$
194.66 g mol^{-1}

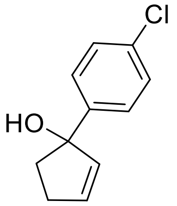

1-Bromo-4-chlorobenzene (0.230 g, 1.20 mmol, 1.00 equiv) was dissolved in THF (2.5 mL) and cooled to -78 °C. To the solution *n*BuLi (0.53 mL, 2.5 M in hexanes, 1.3 mmol, 1.1 equiv) was added dropwise and the obtained suspension was allowed to stir at -78 °C for 30 min. Cyclopent-2-en-1-one (0.10 mL, 1.2 mmol, 1.0 equiv) in THF (0.5 mL) was then added dropwise at the same temperature and the suspension was allowed to stir at -78 °C for 2 h. The reaction mixture was quenched with sat. aq. NH_4Cl (2 mL), diluted with water (5 mL) and Et_2O (5 mL). The organic layer was separated and the aqueous layer was extracted with Et_2O (3 x 5 mL). The organic layers were combined, washed with brine and dried over Na_2SO_4. Volatiles were removed *in vacuo* and the crude product was purified by flash column chromatography (SiO_2, pentane:Et_2O; 3:1) to get alcohol **S13** as a colourless oil (0.161 g, 69%).

^{1}**H NMR** (400 MHz, C_6D_6): δ 7.21 – 7.11 (m, 4H), 5.64 (dt, *J* = 5.1, 2.4 Hz, 1H), 5.45 (dt, *J* = 5.7, 2.1 Hz, 1H), 2.19 (dddt, *J* = 15.7, 7.8, 4.9, 2.2 Hz, 1H), 2.06 – 1.85 (m, 3H), 1.26 – 1.14 (m, 1H).

^{13}**C NMR** (101 MHz, $CDCl_3$): δ 146.4, 137.0 (2C), 134.4 (2C), 132.7, 128.5, 126.9, 86.3, 42.5, 31.5.

The analytical data are consistent with those previously reported in the literature.[12]

---

[11] B. T. Cho, S. K. Kang, M. S. Kim, S. R. Ryu, D. K. An, *Tetrahedron* **2006**, *62*, 8164–8168.
[12] H. Mayr, T. Bug, M. F. Gotta, N. Hering, B. Irrgang, B. Janker, B. Kempf, R. Loos, A. R. Ofial, G. Remennikov, H. Schimmel, *J. Am. Chem. Soc.* **2001**, *39*, 9500–9512.

3-(4-Chlorophenyl)cyclopent-2-en-1-one S14.

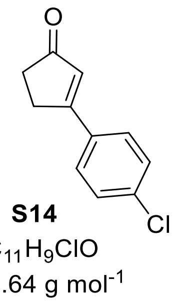

S14
$C_{11}H_9ClO$
192.64 g mol^{-1}

1-Bromo-4-chlorobenzene (1.61 g, 8.4 mmol, 2.00 equiv) was dissolved in THF (12 mL) and cooled to -78 °C. *n*BuLi (3.4 mL, 2.5 M in hexanes, 8.5 mmol, 2.0 equiv) was added dropwise and the obtained suspension was stirred at the same temperature for 30 min. The reaction mixture was warmed to 0 °C and 3-ethoxycyclopent-2-en-1-one (0.50 mL, 4.2 mmol, 1.0 equiv) in THF (3 mL) was then added dropwise. The obtained suspension was warmed to rt, stirred overnight and then quenched with sat. aq. NH_4Cl (3 mL). The mixture was diluted with water (15 mL) and extracted with Et_2O (3 × 15 mL). The organic layers were combined, washed with brine (45 mL) and dried over Na_2SO_4. Volatiles were removed *in vacuo* and the crude product was purified by flash column chromatography (SiO_2; pentane:Et_2O; 100:0 to 0:100) to obtain ketone **S14** as a brown oil (0.375 g, 46%).

^{1}H NMR (400 MHz, $CDCl_3$): δ 7.63 – 7.53 (m, 2H), 7.48 – 7.39 (m, 2H), 6.55 (t, *J* = 1.8 Hz, 1H), 3.06 – 2.97 (m, 2H), 2.66 – 2.52 (m, 2H).

^{13}C NMR (101 MHz, $CDCl_3$): δ 209.2, 172.5, 137.4, 132.7, 129.4 (2C), 128.2 (2C), 128.0, 35.4, 28.8.

IR (film) ν_{max}: 2919, 1704, 1687, 1599, 1490, 1408, 1188, 1092, 1013, 825, 717 cm^{-1}.

HRMS (ESI): calculated for $C_{11}H_9ClONa$ $[M+Na]^+$ requires m/z 215.0234, found m/z 215.0230 (*Δ* = - 1.95 ppm).

3-(4-Chlorophenyl)cyclopent-2-en-1-ol S15.

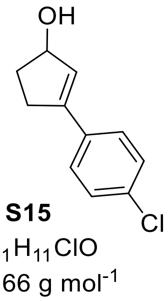

S15
$C_{11}H_{11}ClO$
194.66 g mol^{-1}

$CeCl_3 \times 7\ H_2O$ (0.677 g, 1.82 mmol, 1.03 equiv) was added in one portion to the solution of ketone **S14** (0.340 g, 1.76 mmol, 1.00 equiv) in MeOH (6.3 mL) at 0 °C. To the obtained suspension $NaBH_4$ (69 mg, 1.8 mmol, 1.0 equiv) was added portionwise and the reaction mixture was stirred at the same temperature until judged to be complete by TLC. The mixture was then diluted with water (10 mL) and extracted with CH_2Cl_2 (3 × 10 mL). The organic layers were combined, washed with brine (30 mL) and dried over Na_2SO_4. Volatiles were removed *in vacuo* and the crude product was purified by flash column chromatography (SiO_2; CH_2Cl_2:Et_2O; 9:1) to obtain allylic alcohol **S15** as a pale yellow solid (0.219 g, 64%).

^{1}H NMR (400 MHz, CD_2Cl_2): δ 7.48 – 7.37 (m, 2H), 7.36 – 7.26 (m, 2H), 6.22 (q, *J* = 2.1 Hz, 1H), 5.02 – 4.90 (m, 1H), 2.92 – 2.79 (m, 1H), 2.67 – 2.55 (m, 1H), 2.44 (dddd, *J* = 13.5, 8.8, 7.3, 4.6 Hz, 1H), 1.83 (dddd, *J* = 13.8, 8.9, 5.1, 4.0 Hz, 1H), 1.65 (br, 1H).

^{13}C NMR (101 MHz, CD_2Cl_2): δ 144.9, 134.8, 133.8, 128.9 (3C), 127.8 (2C), 78.1, 34.2, 31.8.

M.p: 91-93 °C

IR (film) ν_{max}: 3317, 2971, 2360, 2341, 1775, 1493, 1335, 1094, 1050, 821 cm^{-1}.

HRMS (ESI): A stable molecular ion could not be detected by ESI, APCI or EI.

*4.6 Cyclisation cascade methodology: synthesis of **2a**-**2w**.*

General procedure B.

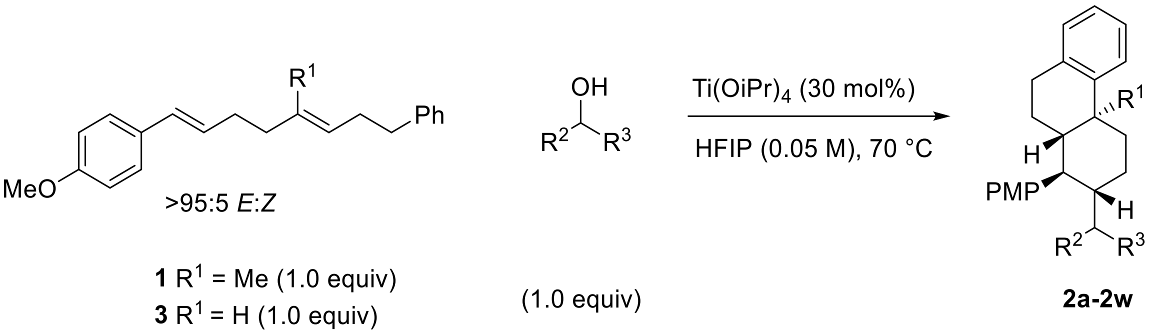

Diene **1, 3** or **7-11** (1.00 equiv) and the corresponding alcohol (1.00 equiv) were transferred to a microwave vial which was then purged with nitrogen. The balloon of nitrogen was removed and HFIP followed by a stock solution of Ti(O*i*Pr)$_4$ in HFIP (0.30 equiv, 0.066 M) were added to obtain an overall 0.050 M solution of a diene substrate. The solution was heated at 70 °C in an oil bath for 2 h, then cooled to rt and diluted with water (3 mL). The obtained suspension was transferred to a separatory funnel and extracted with CH_2Cl_2 (3 × 4 mL). The organic layers were combined, washed with brine and volatiles were removed *in vacuo*. The crude product was purified by flash column chromatography using the appropriate mixture of eluents.

(±)-(1*R*,2*R*,4*aS*,10*aS*)-2-(4-Methoxybenzyl)-1-(4-methoxyphenyl)-4*a*-methyl-1,2,3,4,4*a*,9,10,10*a*-octahydrophenanthrene 2a.

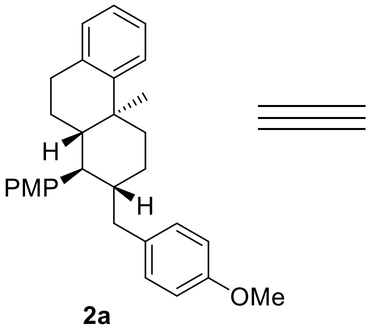

2a
$C_{30}H_{34}O_2$
426.60 g mol^{-1}

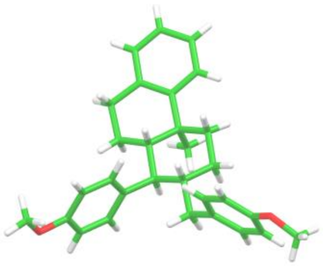

[X-ray]

Diene **1** (25.0 mg, 81.6 µmol, 1.00 equiv), 4-methoxybenzyl alcohol (11.3 mg, 81.6 µmol, 1.00 equiv) and Ti(O*i*Pr)$_4$ (7.4 µL, 25 µmol, 0.30 equiv) in HFIP (1.6 mL, 0.050 M) were subjected to the general procedure **B**. The crude product was purified by flash column chromatography (SiO_2; 60 Å, 15–40 µm, pentane:Et_2O; 24:1) to furnish compound **2a** as a colourless oil which crystallised on standing (22.7 mg, 65%).

^{1}H NMR (400 MHz, $CDCl_3$): δ 7.30 (dd, *J* = 7.8, 1.5 Hz, 1H), 7.20 (dd, *J* = 8.7, 2.2 Hz, 1H), 7.16 – 6.95 (m, 5H), 6.91 (d, *J* = 8.6 Hz, 2H), 6.84 (dd, *J* = 8.3, 2.9 Hz, 1H), 6.77 (d, *J* = 8.6 Hz, 2H), 3.84 (s, 3H), 3.77 (s, 3H), 2.80 – 2.61 (m, 2H), 2.51 (dd, *J* = 13.4, 2.8 Hz, 1H), 2.38 – 2.25 (m, 2H), 2.00 (dd, *J* = 13.4, 10.1 Hz, 1H), 1.78 – 1.65 (m, 3H), 1.55 – 1.34 (m, 3H), 1.31 – 1.22 (m, 1H), 1.19 (s, 3H).

^{13}C NMR (101 MHz, $CDCl_3$): δ 158.1, 157.7, 148.1, 136.7, 135.7, 133.6, 132.7, 130.1 (2C), 129.3, 125.9, 125.7, 125.6, 124.7, 115.3, 113.6 (2C), 112.8, 55.4, 55.3, 50.5, 47.8, 46.1, 40.4, 37.6 (2C), 29.6, 27.5, 22.7 (2C).

M.p: 139 °C.

IR (film) $ν_{max}$: 2929, 2834, 1611, 1510, 1301, 1243, 1177, 1027, 829, 809, 761 cm^{-1}.

HRMS (ESI): calculated for $C_{30}H_{35}O_2$ $[M+H]^+$ requires m/z 427.2632, found m/z 427.2632 (Δ = 0.06 ppm).

(±)-4-(((1*R*,2*R*,4a*S*,10a*S*)-1-(4-Methoxyphenyl)-4*a*-methyl-1,2,3,4,4*a*,9,10,10*a*-octahydrophenanthren-2-yl)methyl)phenol 2b.

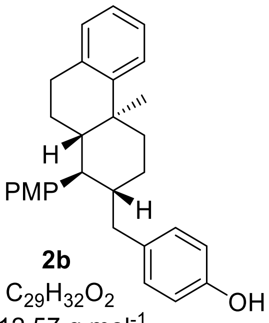

2b
$C_{29}H_{32}O_2$
412.57 g mol^{-1}

Diene **1** (30.6 mg, 0.100 mmol, 1.00 equiv), (2,4-dimethoxyphenyl)methanol (12.4 mg, 0.100 mmol, 1.00 equiv) and $Ti(O\textit{i}Pr)_4$ (9.0 µL, 30 µmol, 0.30 equiv) in HFIP (2.0 mL, 0.050 M) were subjected to the general procedure **B**. The crude product was purified by flash column chromatography (SiO_2; pentane:Et_2O; 7:3) to furnish compound **2b** as a sticky oil (27.3 mg, 66%).[13]

^{1}H NMR (400 MHz, $CDCl_3$): δ 7.30 (d, *J* = 7.8 Hz, 1H), 7.24 – 6.92 (m, 6H), 6.90 – 6.80 (m, 3H), 6.69 (d, *J* = 8.3 Hz, 2H), 4.73 (br, 1H), 3.84 (s, 3H), 2.82 – 2.61 (m, 2H), 2.50 (dd, *J* = 13.5, 2.7 Hz, 1H), 2.38 – 2.24 (m, 2H), 1.99 (dd, *J* = 13.5, 10.1 Hz, 1H), 1.78 – 1.63 (m, 3H), 1.56 – 1.35 (m, 3H), 1.32 – 1.22 (m, 1H), 1.19 (s, 3H).

---

[13] Relative stereochemistry is determined by analogy to product **2a**.

^{13}C NMR (101 MHz, $CDCl_3$): δ 158.0, 153.5, 148.1, 136.7, 135.7, 133.7, 132.6, 130.2 (2C), 129.3, 125.9, 125.7, 125.6, 124.7, 115.3, 115.0 (2C), 112.8, 55.4, 50.5, 47.8, 46.1, 40.4, 37.5 (2C), 29.6, 27.4, 22.7 (2C).

IR (film) ν_{max}: 3400, 2923, 2853, 1611, 1511, 1447, 1240, 1177, 1037, 830, 763 cm^{-1}.

HRMS (ESI): calculated for $C_{29}H_{31}O_2$ $[M-H]^-$ requires m/z 411.2330, found m/z 411.2316 (Δ = -3.37 ppm).

(±)-4-(((1*R*,2*R*,4*aS*,10*aS*)-1-(4-Methoxyphenyl)-4*a*-methyl-1,2,3,4,4*a*,9,10,10*a*-octahydrophenanthren-2-yl)methyl)aniline 2c.

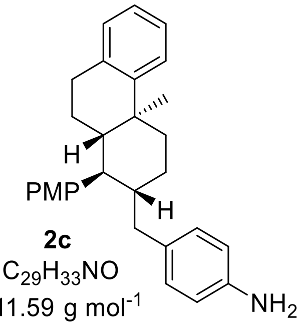

2c
$C_{29}H_{33}NO$
411.59 g mol^{-1}

Diene **1** (30.6 mg, 0.100 mmol, 1.00 equiv), (4-aminophenyl)methanol (12.3 mg, 0.100 mmol, 1.00 equiv) and $Ti(O\textit{i}Pr)_4$ (9.0 µL, 30.0 µmol, 0.30 equiv) in HFIP (2.0 mL, 0.050 M) were subjected to the general procedure **B**. The crude product was purified by flash column chromatography (SiO_2; CH_2Cl_2:Et_2O; 9:1) to furnish compound **2c** as a brown oil (25.4 mg, 62%).[14]

^{1}H NMR (400 MHz, $CDCl_3$): δ 7.30 (dd, *J* = 7.8, 1.5 Hz, 1H), 7.22 – 6.94 (m, 6H), 6.83 (dd, *J* = 8.2, 2.8 Hz, 1H), 6.78 (d, *J* = 8.3 Hz, 2H), 6.57 (d, *J* = 8.3 Hz, 2H), 3.83 (s, 3H), 3.52 (br, 2H), 2.80 – 2.61 (m, 2H), 2.46 (dd, *J* = 13.3, 2.8 Hz, 1H), 2.35 – 2.25 (m, 2H), 1.94 (dd, *J* = 13.4, 10.2 Hz, 1H), 1.79 – 1.63 (m, 3H), 1.55 – 1.34 (m, 3H), 1.30 – 1.22 (m, 1H), 1.18 (s, 3H).

^{13}C NMR (101 MHz, $CDCl_3$): δ 158.0, 148.1, 144.1, 136.8, 135.7, 132.7, 131.6, 130.0 (2C), 129.3, 125.9, 125.7, 125.5, 124.8, 115.3, 115.1 (2C), 112.7, 55.4, 50.5, 47.8, 46.1, 40.5, 37.6 (2C), 29.6, 27.4, 22.7 (2C).

IR (film) ν_{max}: 3369, 2925, 1611, 1512, 1267, 1243, 1178, 1037, 909, 829, 732 cm^{-1}.

HRMS (ESI): calculated for $C_{29}H_{34}NO$ $[M+H]^+$ requires m/z 412.2635, found m/z 412.2629 (Δ = -1.38 ppm).

---

[14] Relative stereochemistry is determined by analogy to product **2a**.

(±)-(1*R*,2*R*,4*aS*,10*aS*)-2-(2,4-Dimethoxybenzyl)-1-(4-methoxyphenyl)-4*a*-methyl-1,2,3,4,4*a*,9,10,10*a*-octahydrophenanthrene 2d.

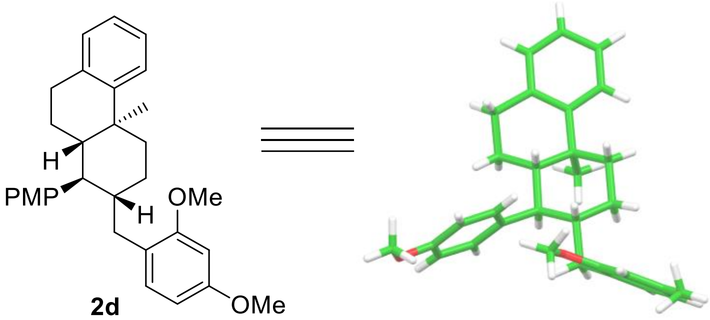

2d
$C_{31}H_{36}O_3$
456.63 g mol^{-1}

[X-ray]

Diene **1** (30.6 mg, 0.100 mmol, 1.00 equiv), (2,4-dimethoxyphenyl)methanol (16.8 mg, 0.100 mmol, 1.00 equiv) and Ti(O*i*Pr)$_4$ (9.0 µL, 30 µmol, 0.30 equiv) in HFIP (2.0 mL, 0.050 M) were subjected to the general procedure **B**. The crude product was purified by flash column chromatography (SiO_2; 60 Å, 15–40 µm**;** pentane:Et_2O; 9:1) to furnish compound **2d** as a white solid (15.0 mg, 33%).

^{1}H NMR (400 MHz, $CDCl_3$): δ 7.30 (dd, *J* = 7.8, 1.4 Hz, 1H), 7.21 (dd, *J* = 8.6, 2.3 Hz, 1H), 7.16 – 7.04 (m, 3H), 7.03 – 6.92 (m, 2H), 6.89 – 6.78 (m, 2H), 6.42 – 6.31 (m, 2H), 3.84 (s, 3H), 3.77 (s, 3H), 3.69 (s, 3H), 2.80 – 2.60 (m, 2H), 2.46 (dd, *J* = 13.2, 2.9 Hz, 1H), 2.36 – 2.23 (m, 2H), 2.04 (dd, *J* = 13.2, 10.6 Hz, 1H), 1.86 – 1.63 (m, 3H), 1.53 – 1.35 (m, 3H), 1.32 – 1.19 (m, 4H).

^{13}C NMR (101 MHz, $CDCl_3$): δ 159.0, 158.6, 158.0, 148.3, 136.8, 135.8, 132.5, 130.9, 129.2, 126.4, 125.6, 125.5, 124.8, 122.5, 115.0, 112.4, 103.7, 98.5, 55.4, 55.3, 55.2, 50.9, 47.9, 44.3, 37.7, 37.6, 35.0, 29.7, 27.5, 22.8, 22.7.

M.p: 119-121 °C.

IR (film) ν_{max}: 2931, 1611, 1586, 1508, 1463, 1207, 1155, 1039, 909, 824, 761, 731 cm^{-1}.

HRMS (ESI): calculated for $C_{31}H_{37}O_3$ $[M+H]^+$ requires m/z 457.2737, found m/z 457.2737 (*Δ* = 0.05 ppm).

(±)-(1*R*,2*R*,4a*S*,10a*S*)-2-(2-Bromo-4-methoxybenzyl)-1-(4-methoxyphenyl)-4*a*-methyl-1,2,3,4,4*a*,9,10,10*a*-octahydrophenanthrene 2e.

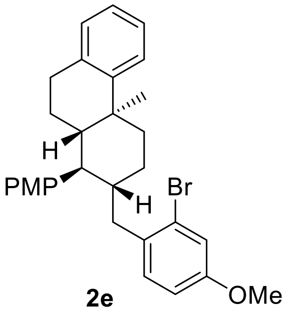

2e
$C_{30}H_{33}BrO_2$
505.50 g mol^{-1}

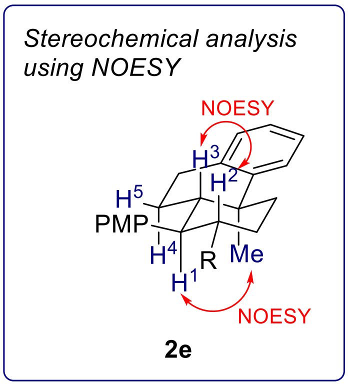

Diene **1** (30.6 mg, 0.100 mmol, 1.00 equiv), (2-bromo-4-methoxyphenyl)methanol (21.6 mg, 0.100 mmol, 1.00 equiv) and Ti(O*i*Pr)$_4$ (9.0 µL, 30 µmol, 0.30 equiv) in HFIP (2.0 mL, 0.050 M) were subjected to the general procedure **B**. The crude product was purified by flash column chromatography (SiO_2; 60 Å, 15–40 µm**;** pentane:Et_2O; 49:1) to furnish compound **2e** as a colourless oil (17.4 mg, 34%).

^{1}H NMR (400 MHz, $CDCl_3$): δ 7.29 (dd, *J* = 7.8, 1.5 Hz, 1H), 7.24 (dd, *J* = 8.6, 2.2 Hz, 1H), 7.17 – 6.99 (m, 5H), 6.97 – 6.89 (m, 2H), 6.83 (dd, *J* = 8.2, 2.8 Hz, 1H), 6.74 (dd, *J* = 8.4, 2.6 Hz, 1H), 3.83 (s, 3H), 3.75 (s, 3H), 2.80 – 2.63 (m, 2H), 2.58 (dd, *J* = 13.5, 3.4 Hz, 1H), 2.40 – 2.27 (m, 2H), 2.23 (dd, *J* = 13.6, 11.0 Hz, 1H), 1.95 – 1.82 (m, 1H), 1.76 (ddd, *J* = 12.4, 11.3, 3.0 Hz, 1H), 1.67 – 1.36 (m, 4H), 1.32 – 1.18 (m, 4H).

^{13}C NMR (101 MHz, $CDCl_3$): δ 158.2 (2C), 148.1, 136.2, 135.7, 132.9, 132.4, 131.8, 129.3, 126.4, 125.7, 125.6, 124.8, 124.7, 117.7, 115.2, 113.5, 112.6, 55.6, 55.4, 51.0, 47.7, 44.3, 40.5, 37.5 (2C), 29.6, 27.3, 22.8, 22.7.

NOESY- 2D (600 MHz, $CDCl_3$): between H-1 and Me, between H-3 and H-2.

Note: the appearance of ^{1}H NMR signal for H-3: 1.76 (ddd, *J* = 12.4, 11.3, 3.0 Hz, 1H, H-3) is indicative of a trans-diaxial arrangement between H-3 and H-1, H-3 and H-4 and equatorial arrangement between H-3 and H-5.

IR (film) ν_{max}: 2932, 2361, 2343, 1606, 1511, 1492, 1241, 1178, 1038, 824, 761, 728 cm^{-1}.

HRMS (ESI): calculated for $C_{30}H_{34}O_2Br$ $[M+H]^+$ requires m/z 505.1737, found m/z 505.1738 (*Δ* = 0.25 ppm).

(±)-3-((1*R*,2*R*,4*aS*,10*aS*)-1-(4-Methoxyphenyl)-4*a*-methyl-1,2,3,4,4*a*,9,10,10*a*-octahydrophenanthren-2-yl)methyl)-1-methyl-1H-indole 2f.

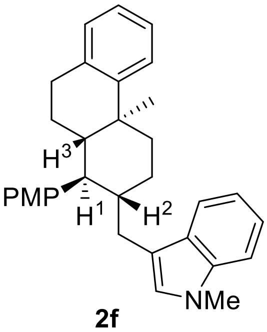

2f

$C_{32}H_{35}NO$

449.64 g mol^{-1}

Diene **1** (30.6 mg 0.100 mmol, 1.00 equiv), (1-methyl-1H-indol-3-yl)methanol[15] (16.1 mg, 0.100 mmol, 1.00 equiv) and Ti(O*i*Pr)$_4$ (9.0 µL, 30 µmol, 0.30 equiv) in HFIP (2.0 mL, 0.050 M) were subjected to the general procedure **B** except that the reaction was performed at rt and for 2.5 h. The crude product was purified by flash column chromatography (SiO_2; 60 Å, 15–40 µm**;** pentane:Et_2O; 19.1:0.9) to furnish compound **2f** as a colourless oil (25.1 mg, 56%).

^{1}H NMR (400 MHz, $CDCl_3$): δ 7.34 – 7.23 (m, 4H), 7.22 – 6.99 (m, 7H), 6.88 (dd, *J* = 8.3, 2.6 Hz, 1H), 6.74 (s, 1H), 3.87 (s, 3H), 3.73 (s, 3H), 2.82 – 2.63 (m, 3H), 2.39 (t, *J* = 11.1 Hz, 1H), 2.34 – 2.15 (m, 2H), 1.94 – 1.71 (m, 3H), 1.54 – 1.39 (m, 3H), 1.35 – 1.28 (m, 1H), 1.23 (s, 3H).

^{13}C NMR (101 MHz, $CDCl_3$): δ 158.1, 148.2, 137.0 (2C), 135.7, 132.7, 129.3, 128.4, 127.0, 126.1, 125.7, 125.5, 124.7, 121.3, 119.4, 118.5, 115.3, 114.0, 112.7, 109.1, 55.4, 50.7, 47.8, 45.0, 37.6 (2C), 32.6, 30.5, 29.7, 28.2, 22.8, 22.7.

NOESY- 2D (600 MHz, $CDCl_3$): between H-1 and Me.

Note: the appearance of ^{1}H NMR signal for H-1: 2.39 (t, *J* = 11.1 Hz, 1H, H-1) is indicative of a trans-diaxial arrangement between H-1 and H-2; H-1 and H-3.

IR (film) ν_{max}: 2927, 1611, 1510, 1244, 1177, 1038, 908, 823, 761, 734 cm^{-1}.

HRMS (ESI): calculated for $C_{32}H_{36}ON$ $[M+H]^+$ requires m/z 450.2791, found m/z 450.2792 (*Δ* = 0.14 ppm).

[15] M. A. J. Dubois, R. A. Croft, Y. Ding, C. Choi, D. R. Owen, J. A. Bulla, J. J. Mousseau, *RSC Med. Chem*. **2021**, *12*, 2045–2052.

(±)-(1*R*,2*R*,4a*S*,10a*S*)-1-(4-Methoxyphenyl)-2-((*S*)-1-(4-methoxyphenyl)ethyl)-4a-methyl-1,2,3,4,4a,9,10,10a-octahydrophenanthrene (major)-2g and (±)-(1*R*,2*R*,4a*S*,10a*S*)-1-(4-Methoxyphenyl)-2-((*R*)-1-(4-methoxyphenyl)ethyl)-4a-methyl-1,2,3,4,4a,9,10,10a-octahydrophenanthrene (minor)-2g.

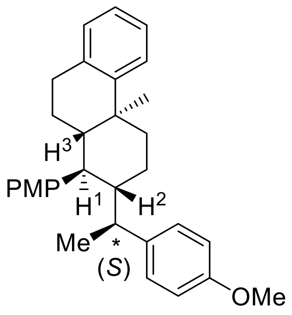

2g (major);

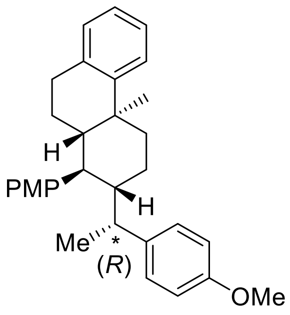

2g (minor);

$C_{31}H_{36}O_2$
440.63 g mol^{-1}
*dr = 90:10

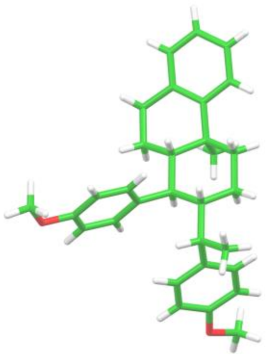

[X-ray of the major diastereomer]

Diene **1** (30.6 mg 0.100 mmol, 1.00 equiv), 1-(4-methoxyphenyl)ethan-1-ol (15.2 mg, 0.100 mmol, 1.00 equiv) and Ti(O*i*Pr)$_4$ (9.0 µL, 30 µmol, 0.30 equiv) in HFIP (2.0 mL, 0.050 M) were subjected to the general procedure **B**. The crude product was purified by flash column chromatography (SiO_2; pentane:Et_2O; 24:1) to furnish compound **2g** as an inseparable mixture of two diastereomers at the exocyclic position as a pale yellow oil (40.3 mg, 92%, 90:10 dr).[16,17]

Major diastereomer (from the mixture)**:**

^{1}H NMR (400 MHz, $CDCl_3$): δ 7.33 – 7.28 (m, 1H), 7.20 – 7.10 (m, 2H), 7.10 – 6.94 (m, 6H), 6.91 (dd, *J* = 8.2, 2.8 Hz, 1H), 6.84 (d, *J* = 8.6 Hz, 2H), 3.87 (s, 3H), 3.83 (s, 3H), 2.74 – 2.53 (m, 3H), 2.40 (dt, *J* = 12.6, 3.5 Hz, 1H), 2.21 (t, *J* = 11.2 Hz, 1H), 1.95 (dt, *J* = 13.0, 3.6 Hz, 1H), 1.85 (tt, *J* = 11.7, 3.0 Hz, 1H), 1.68 – 1.55 (m, 2H), 1.43 (td, *J* = 12.9, 4.2 Hz, 1H), 1.35 – 1.25 (m, 1H), 1.23 – 1.10 (m, 4H), 0.91 (s, 3H).

^{13}C NMR (101 MHz, $CDCl_3$): δ 158.0, 157.8, 148.0, 136.7, 135.8, 135.7, 133.3, 129.9 (2C), 129.2, 126.3, 125.6, 125.5, 124.7, 115.1, 113.0 (2C), 112.7, 55.4, 55.3, 49.3, 49.0, 47.6, 39.0, 37.8, 37.5, 29.7, 22.7, 22.6, 22.1, 20.3.

NOESY- 2D (600 MHz, $CDCl_3$): between H-1 and Me.

---

[16] Relative stereochemistry at the exocyclic benzylic position for the major diastereomer was determined by single crystal X-ray diffraction followed by ^{1}H NMR analysis of the single crystal used for the diffraction experiment.
[17] dr determined by ^{1}H NMR of the crude reaction mixture.

Note: the appearance of ^{1}H NMR signal for H-1: 2.21 (t, *J* = 11.2 Hz, 1H, H-1) is indicative of a trans-diaxial arrangement between H-1 and H-2; H-1 and H-3.

Characteristic peaks for the minor diastereomer (from the mixture)**:**

^{1}H NMR (400 MHz, $CDCl_3$): δ 3.86 (s, 3H), 3.79 (s, 3H).

^{13}C NMR (101 MHz, $CDCl_3$): δ 157.6, 139.0, 136.5, 132.1, 128.6, 125.9, 115.5, 113.4, 50.5, 48.2, 38.0, 22.8, 11.5.

IR (film) ν_{max}: 2932, 1610, 1510, 1463, 1245, 1178, 1037, 908, 828, 761, 730 cm^{-1}.

HRMS (ESI): calculated for $C_{31}H_{37}O_2$ $[M+H]^+$ requires m/z 441.2788, found m/z 441.2787 (*Δ* = -0.18 ppm).

(±)-5-((*S*)-1-((1*R*,2*R*,4*aS*,10*aS*)-1-(4-Methoxyphenyl)-4*a*-methyl-1,2,3,4,4*a*,9,10,10*a*-octahydrophenanthren-2-yl)ethyl)benzo[d][1,3]dioxole (major)-2h and (±)-5-((*R*)-1-((1*R*,2*R*,4*aS*,10*aS*)-1-(4-Methoxyphenyl)-4*a*-methyl-1,2,3,4,4*a*,9,10,10*a*-octahydrophenanthren-2-yl)ethyl)benzo[d][1,3]dioxole (minor)-2h.

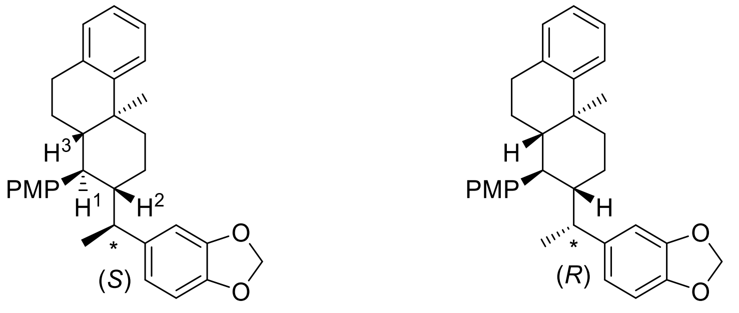

2h (major) **2h** (minor)

$C_{31}H_{34}O_3$
454.61 g mol^{-1}
*dr = 90:10

Diene **1** (30.6 mg 0.100 mmol, 1.00 equiv), 1-(benzo[d][1,3]dioxol-5-yl)ethan-1-ol[18] (16.6 mg, 0.100 mmol, 1.00 equiv) and $Ti(O\textit{i}Pr)_4$ (9.0 µL, 30 µmol, 0.30 equiv) in HFIP (2.0 mL, 0.050 M) were subjected to the general procedure **B**. The crude product was purified by flash column chromatography (SiO_2; pentane:Et_2O; 19:1) to furnish compound **2h** as an inseparable mixture of two diastereomers at the exocyclic position as a bage foam (38.5 mg, 85%, 90:10 dr)[19,20].

Major diastereomer (from the mixture)**:**

[18] A. Joncour, A. Décor, J. M. Liu, M. E. Tran Huu Dau, O. Baudoin, *Chem. Eur. J.* **2007**, *13*, 5450 – 5465.
[19] Relative stereochemistry of the major and minor diastereomers is determined by analogy to compound **2g**.
[20] dr determined by ^{1}H NMR after column chromatography due to the overlapping signals in the ^{1}H spectrum of the crude mixture.

^{1}H NMR (400 MHz, $CDCl_3$): δ 7.30 (dd, *J* = 7.9, 1.4 Hz, 1H), 7.18 – 7.04 (m, 4H), 7.02 – 6.86 (m, 3H), 6.74 (d, *J* = 8.0 Hz, 1H), 6.64 (d, *J* = 1.7 Hz, 1H), 6.54 (dd, *J* = 8.0, 1.7 Hz, 1H), 5.95 (s, 2H), 3.86 (s, 3H), 2.75 – 2.51 (m, 3H), 2.40 (dt, *J* = 12.7, 3.5 Hz, 1H), 2.25 (t, *J* = 11.2 Hz, 1H), 1.97 – 1.78 (m, 2H), 1.67 – 1.54 (m, 2H), 1.43 (td, *J* = 13.1, 3.4 Hz, 1H), 1.38 – 1.24 (m, 1H), 1.19 – 1.08 (m, 4H), 0.94 (s, 3H).

^{13}C NMR (101 MHz, $CDCl_3$): δ 158.0, 147.9, 147.0, 145.5, 137.7, 136.6, 135.7, 133.3, 129.2, 126.3, 125.6, 125.5, 124.7, 122.0, 115.2, 112.7, 109.3, 107.5, 100.8, 55.3, 49.3, 49.0, 47.5, 39.6, 37.8, 37.5, 29.6, 22.8, 22.6, 22.3, 20.3.

Note: the appearance of ^{1}H NMR signal for H-1: 2.25 (t, *J* = 11.2 Hz, 1H, H-1) is indicative of a trans-diaxial arrangement between H-1 and H-2; H-1 and H-3.

Characteristic peaks for the minor diastereomer (from the mixture)**:**

^{1}H NMR (400 MHz, $CDCl_3$): δ 6.49 (dd, *J* = 8.0, 1.8 Hz, 1H), 5.91 (s, 2H), 3.85 (s, 3H), 1.21 (s, 3H).

^{13}C NMR (101 MHz, $CDCl_3$): δ 132.1, 128.2, 120.3, 115.4, 108.4, 107.8, 50.6, 48.2, 38.6, 37.6, 11.6.

M.p: 75-81 °C

IR (film) ν_{max}: 3027, 2934, 1610, 1510, 1488, 1440, 1239, 1039, 909, 812, 762, 732 cm^{-1}.

HRMS (ESI): calculated for $C_{31}H_{35}O_3[M+H]^+$ requires m/z 455.2581, found m/z 455.2583 (Δ = 0.44 ppm).

(±)-4-((1*S*)-1-((1*R*,4a*S*,10a*S*)-1-(4-Methoxyphenyl)-4a-methyl-1,2,3,4,4a,9,10,10a-octahydrophenanthren-2-yl)ethyl)-2-methylphenol (major)-2i and (±)-4-((1*R*)-1-((1*R*,4a*S*,10a*S*)-1-(4-Methoxyphenyl)-4a-methyl-1,2,3,4,4a,9,10,10a-octahydrophenanthren-2-yl)ethyl)-2-methylphenol (minor)-2i.

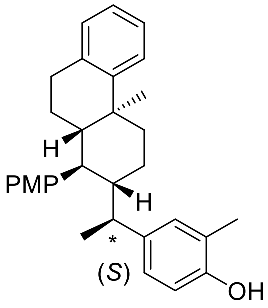

2i (major)

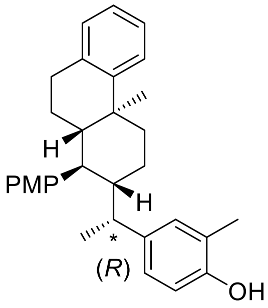

2i (minor)

$C_{31}H_{36}O_2$
440.63 g mol^{-1}
*dr = 89:11

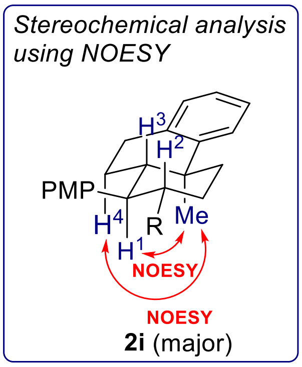

Diene **1** (30.6 mg 0.100 mmol, 1.00 equiv), 4-(1-hydroxyethyl)-2-methylphenol[21] (15.2 mg, 0.100 mmol, 1.00 equiv) and $Ti(O\textit{i}Pr)_4$ (9.0 µL, 30 µmol, 0.30 equiv) in HFIP (2.0 mL, 0.050 M) were subjected to the general procedure **B**. The crude product was purified by flash column chromatography (SiO_2; pentane:Et_2O; 7:3) to furnish compound **2i** as an inseparable mixture of two diastereomers at the exocyclic position as a pale yellow oil (38.1 mg, 87%, 89:11 dr)[22,23].

Major diastereomer (from the mixture)**:**

^{1}H NMR (400 MHz, $CDCl_3$): δ 7.31 (dd, *J* = 7.8, 1.4 Hz, 1H), 7.18 – 6.88 (m, 7H), 6.86 – 6.79 (m, 2H), 6.69 (d, *J* = 7.9 Hz, 1H), 4.64 (br, 1H), 3.87 (s, 3H), 2.76 – 2.51 (m, 3H), 2.40 (dt, *J* = 12.6, 3.4 Hz, 1H), 2.29 – 2.14 (m, 4H), 1.94 (dq, *J* = 13.3, 3.6 Hz, 1H), 1.84 (tt, *J* = 11.8, 3.2 Hz, 1H), 1.67 – 1.54 (m, 2H), 1.50 – 1.38 (m, 1H), 1.37 – 1.25 (m, 1H), 1.20 – 1.09 (m, 4H), 0.92 (s, 3H).

^{13}C NMR (101 MHz, $CDCl_3$): δ 157.9, 151.9, 148.0, 136.7, 135.9, 135.7, 133.4, 131.7, 129.2, 127.5, 126.3, 125.6, 125.5, 124.7, 122.5, 115.0, 114.2, 112.7, 55.4, 49.2, 49.0, 47.5, 39.1, 37.8, 37.5, 29.6, 22.8, 22.6, 22.2, 20.3, 16.1.

NOESY- 2D (600 MHz, $CDCl_3$): between H-1 and Me; between H-4 and Me.

---

[21] M. A. J. Dubois, R. A. Croft, Y. Ding, C. Choi, D. R. Owen, J. A. Bulla, J. J. Mousseau, *RSC Med. Chem*. **2021**, *12*, 2045–2052.
[22] Relative stereochemistry of the major and minor diastereomers is determined by analogy to compound **2g**.
[23] dr determined by ^{1}H NMR after column chromatography due to the overlapping signals in the ^{1}H spectrum of the crude mixture.

Note: the appearance of ^{1}H NMR signal for H-2: 1.84 (tt, *J* = 11.8, 3.2 Hz, 1H, H-2) is indicative of a trans-diaxial arrangement between H-1 and H-2.

Characteristic peaks for the minor diastereomer (from the mixture)**:**

^{1}H NMR (400 MHz, $CDCl_3$): δ 3.86 (s, 3H), 1.21 (s, 3H).

^{13}C NMR (101 MHz, $CDCl_3$): δ 151.7, 139.0, 136.5, 132.1, 130.3, 115.5, 114.4, 50.4, 48.2, 38.0, 11.6.

IR (film) ν_{max}: 3426, 2932, 2361, 1510, 1243, 908, 825, 732 cm^{-1}.

HRMS (ESI): calculated for $C_{31}H_{36}O_2Na$ $[M+Na]^+$ requires m/z 463.2608, found m/z 463.2603 (Δ = -0.88 ppm).

(±)-2-((*S*)-1-((1*R*,2*S*,4*aS*,10*aS*)-1-(4-Methoxyphenyl)-4a-methyl-1,2,3,4,4*a*,9,10,10*a*-octahydrophenanthren-2-yl)ethyl)thiophene (major)-2j and (±)-2-((*R*)-1-((1*R*,2*S*,4*aS*,10*aS*)-1-(4-Methoxyphenyl)-4*a*-methyl-1,2,3,4,4*a*,9,10,10*a*-octahydrophenanthren-2-yl)ethyl)thiophene (minor)-2j.

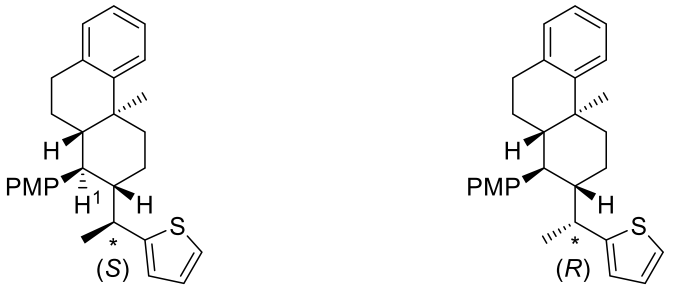

2j (major) **2j** (minor)

$C_{28}H_{32}OS$
416.62 g mol^{-1}
*dr = 85:15

Diene **1** (30.6 mg 0.100 mmol, 1.00 equiv), (*R*)-1-(thiophen-2-yl)ethan-1-ol (12.8 mg, 0.100 mmol, 1.00 equiv) and Ti(O*i*Pr)$_4$ (9.0 µL, 30 µmol, 0.30 equiv) in HFIP (2.0 mL, 0.050 M) were subjected to the general procedure **B**. The crude product was purified by flash column chromatography (SiO_2; 60 Å, 15–40 µm**;** pentane:Et_2O; 49:1) to furnish compound **2j** as an inseparable mixture of two diastereomers as a colourless oil (16.6 mg, 40%, 85:15 dr).[24,25]

[24] dr determined by ^{1}H NMR after column chromatography due to the overlapping signals in the ^{1}H spectrum of the crude mixture.
[25] Relative stereochemistry of the major and minor diastereomers is determined by analogy to compound **2g**.

Major diastereomer (from the mixture)**:**

^{1}H NMR (400 MHz, $CDCl_3$): δ 7.34 – 7.29 (m, 1H), 7.19 – 6.92 (m, 8H), 6.89 – 6.84 (m, 1H), 6.73 – 6.68 (m, 1H), 3.85 (s, 3H), 2.98 (qd, *J* = 7.3, 2.5 Hz, 1H), 2.75 – 2.58 (m, 2H), 2.49 – 2.39 (m, 2H), 1.94 – 1.76 (m, 2H), 1.70 – 1.53 (m, 3H), 1.40 – 1.11 (m, 5H), 1.00 (s, 3H).

^{13}C NMR (101 MHz, $CDCl_3$): δ 158.1, 147.9, 147.2, 136.2, 135.7, 133.4, 129.3, 126.1 (2C), 125.7, 125.6, 125.0, 124.8, 123.0, 115.2, 112.7, 55.3, 49.2, 48.6, 47.3, 37.6, 37.5, 36.1, 29.7, 22.8, 22.6, 21.9, 21.8.

NOESY- 2D (600 MHz, $CDCl_3$): between H-1 and Me.

Characteristic peaks for the minor diastereomer (from the mixture)**:**

^{1}H NMR (400 MHz, $CDCl_3$): δ 3.83 (s, 3H), 2.84 (dq, *J* = 7.1, 2.5 Hz, 1H), 1.21 (s, 3H).

^{13}C NMR (101 MHz, $CDCl_3$): δ 136.0, 132.0, 126.5, 125.9, 125.6, 124.7, 122.9, 122.4, 115.5, 113.0, 51.1, 48.2, 47.3, 35.3, 29.6, 22.5, 20.7, 13.3.

IR (film) ν_{max}: 2933, 1610, 1510, 1246, 1177, 1038, 908, 825, 731, 694 cm^{-1}.

HRMS (APCI): calculated for $C_{28}H_{33}OS$ $[M+H]^+$ requires m/z 417.2247, found m/z 417.2247 (*Δ* = -0.01 ppm).

(±)-(1*R*,2*R*,4*aS*,10*aS*)-2-((*S*)-2,3-Dihydro-1H-inden-1-yl)-1-(4-methoxyphenyl)-4*a*-methyl-1,2,3,4,4*a*,9,10,10*a*-octahydrophenanthrene (major)-2k and (±)-(1*R*,2*R*,4*aS*,10*aS*)-2-((*R*)-2,3-Dihydro-1H-inden-1-yl)-1-(4-methoxyphenyl)-4*a*-methyl-1,2,3,4,4*a*,9,10,10*a*-octahydrophenanthrene (minor)-2k.

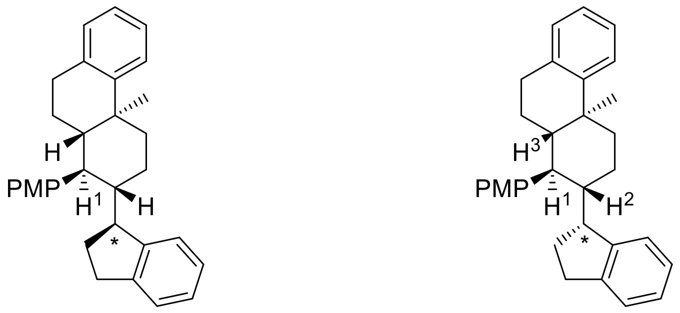

2k (major) **2k** (minor)

$C_{31}H_{34}O$
422.61 g mol^{-1}
dr = 65:35

Diene **1** (30.6 mg 0.100 mmol, 1.00 equiv), 2,3-dihydro-1H-inden-1-ol (13.4 mg, 0.100 mmol, 1.00 equiv) and Ti(O*i*Pr)$_4$ (9.0 µL, 30 µmol, 0.30 equiv) in HFIP (2.0 mL, 0.050 M) were

subjected to the general procedure **B**. The crude product was purified by flash column chromatography (SiO_2; pentane:Et_2O; 49:1) to furnish compound **2k** as an inseparable mixture of two diastereomers as a white foam (32.6 mg, 77%, 65:35 dr).[26,27]

^{1}H NMR (600 MHz, $CDCl_3$): δ 7.48 (d, *J* = 7.2 Hz, $1H_{maj}$), 7.38 – 6.93 (m, $10H_{maj}$, $11H_{min}$), 6.90 – 6.82 (m, $1H_{maj}$, $1H_{min}$), 3.89 – 3.80 (m, $3H_{maj}$, $3H_{min}$), 3.11 (ddd, *J* = 8.5, 5.4, 2.1 Hz, $1H_{maj}$), 3.02 – 2.87 (m, $2H_{maj}$, $2H_{min}$), 2.84 – 2.66 (m, $3H_{maj}$, $3H_{min}$), 2.54 (t, *J* = 11.3 Hz, $1H_{min}$), 2.40 – 2.30 (m, $1H_{maj}$, $1H_{min}$), 2.26 – 2.20 (m, $1H_{min}$), 2.14 – 1.97 (m, $1H_{maj}$, $2H_{min}$), 1.97 – 1.90 (m, $1H_{maj}$), 1.88 – 1.78 (m, $1H_{maj}$, $1H_{min}$), 1.77 – 1.69 (m, $2H_{maj}$), 1.67 – 1.42 (m, $2H_{maj}$, $4H_{min}$), 1.41 – 1.27 (m, $2H_{maj}$, $1H_{min}$), 1.23 (s, $3H_{min}$), 1.12 (s, $3H_{maj}$).

^{13}C NMR (101 MHz, $CDCl_3$) **for the major diastereomer** (from the mixture): δ 158.0, 147.9, 145.7, 145.0, 136.6, 135.7, 132.5, 129.3, 126.3 (2C), 125.9, 125.7, 125.6, 125.2, 124.8, 124.7, 115.4, 112.8, 55.3, 49.8, 48.5, 47.7, 45.8, 37.7, 37.6, 32.3, 31.6, 29.7, 22.9, 22.8, 22.0. **Selected peaks for the minor diastereomer** (from the mixture): δ 158.1, 148.0, 146.3, 144.4, 136.4, 132.1, 129.3, 126.1, 126.0, 125.9, 125.6, 124.7, 124.3, 123.4, 115.2, 112.9, 55.3, 48.0, 47.9, 46.8, 46.3, 37.6, 37.5, 32.0, 29.6, 25.1, 22.9, 22.8, 21.0.

NOESY- 2D (600 MHz, $CDCl_3$): between H-1_{maj} and Me_{maj}, between H-1_{min} and Me_{min}.

Note: the appearance of ^{1}H NMR signal for H-1_{min}: 2.54 (t, *J* = 11.3 Hz, 1H, H-1_{min}) is indicative of a trans-diaxial arrangement between H-1_{min} and H-2_{min}; H-1_{min} and H-3_{min}.

M.p. 67-77 °C

IR (film) $ν_{max}$: 2936, 1511, 1244, 1177, 1038, 908, 830, 757, 729 cm^{-1}.

HRMS (APCI): calculated for $C_{31}H_{35}O$ $[M+H]^+$ requires m/z 423.2682, found m/z 423.2681 (*Δ* = - 0.40 ppm).

[26] dr determined after column chromatography due to the overlapping signals in the ^{1}H spectrum of the crude mixture.
[27] Relative stereochemistry of the major and minor diastereomers is determined by analogy to compound **2g**.

(±)-(1*R*,2*S*,4a*S*,10a*S*)-2-(Bis(4-chlorophenyl)methyl)-1-(4-methoxyphenyl)-4a-methyl-1,2,3,4,4a,9,10,10a-octahydrophenanthrene 2l.

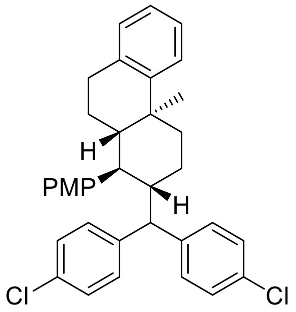

2l
$C_{35}H_{34}Cl_2O$
541.56 g mol^{-1}

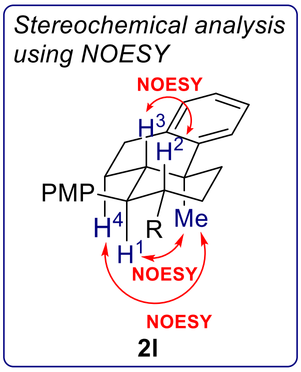

Diene **1** (30.6 mg 0.100 mmol, 1.00 equiv), bis(4-chlorophenyl)methanol (25.3 mg, 0.100 mmol, 1.00 equiv) and Ti(O*i*Pr)$_4$ (9.0 µL, 30 µmol, 0.30 equiv) in HFIP (2.0 mL, 0.050 M) were subjected to the general procedure **B**. The crude product was purified by flash column chromatography (SiO_2; pentane:Et_2O; 49:1) to furnish compound **2l** as a white foam (43.6 mg, 81%).

^{1}H NMR (400 MHz, CD_2Cl_2): δ 7.34 – 6.97 (m, 13H), 6.94 – 6.78 (m, 3H), 3.89 (d, *J* = 3.6 Hz, 1H), 3.81 (s, 3H), 2.76 – 2.49 (m, 3H), 2.43 – 2.31 (m, 2H), 1.91 – 1.80 (m, 1H), 1.74 (ddd, *J* = 12.4, 10.9, 2.7 Hz, 1H), 1.69 – 1.58 (m, 2H), 1.41 – 1.29 (m, 1H), 1.21 – 1.11 (m, 1H), 1.02 (s, 3H).

^{13}C NMR (101 MHz, CD_2Cl_2): δ 158.6, 148.1, 143.2, 140.6, 135.9 (2C), 133.5, 132.5, 132.4 (2C), 131.7, 130.1 (2C), 129.5, 128.5 (4C), 126.4, 125.9, 125.8, 124.9, 115.5, 113.2, 55.6, 51.7, 49.3, 48.5, 47.5, 38.1, 37.8, 29.8, 24.5, 22.9 (2C).

NOESY- 2D (600 MHz, CD_2Cl_2): between H-1 and Me, between H-2 and H-3**,** between H-4 and Me.

M.p: 95-97 °C

IR (film) ν_{max}: 2935, 1510, 1490, 1245, 1177, 1091, 1037, 1013, 953, 829, 761, 712 cm^{-1}.

HRMS (APCI): calculated for $C_{35}H_{35}Cl_2O$ $[M+H]^+$ requires m/z 541.2059, found m/z 541.2059 (*Δ* = - 0.07 ppm).

(±)-(1*R*,2*S*,4a*S*,10a*S*)-2-(Bis(4-methoxyphenyl)methyl)-1-(4-methoxyphenyl)-4a-methyl-1,2,3,4,4a,9,10,10a-octahydrophenanthrene 2m.

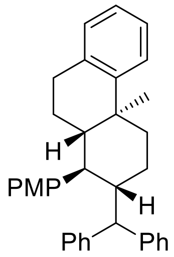

2m
$C_{35}H_{36}O$
472.67 g mol^{-1}

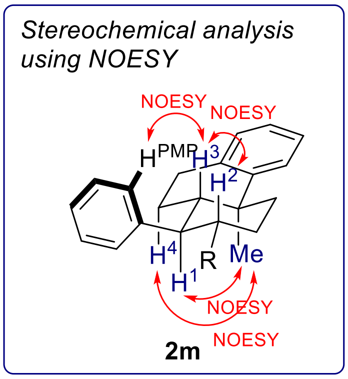

Diene **1** (30.6 mg 0.100 mmol, 1.00 equiv), benzhydrol (18.4 mg, 0.100 mmol, 1.00 equiv) and Ti(O*i*Pr)$_4$ (9.0 µL, 30 µmol, 0.30 equiv) in HFIP (2.0 mL, 0.050 M) were subjected to the general procedure **B**. The crude product was purified by flash column chromatography (SiO_2; pentane:Et_2O; 49:1) to furnish compound **2m** as a colourless sticky foam (40.5 mg, 86%).

^{1}H NMR (400 MHz, $CDCl_3$): δ 7.35 – 7.19 (m, 7H), 7.17 – 7.05 (m, 7H), 7.03 – 6.98 (m, 1H), 6.95 – 6.88 (m, 2H), 6.84 (dd, *J* = 8.3, 2.8 Hz, 1H), 3.95 (d, *J* = 3.1 Hz, 1H), 3.84 (s, 3H), 2.77 – 2.53 (m, 3H), 2.43 – 2.33 (m, 2H), 1.99 – 1.89 (m, 1H), 1.78 – 1.61 (m, 3H), 1.39 – 1.27 (m, 1H), 1.22 – 1.13 (m, 1H), 1.01 (s, 3H).

^{13}C NMR (101 MHz, $CDCl_3$): δ 158.1, 147.8, 144.7, 141.8, 136.0, 135.6, 133.3, 131.0 (2C), 129.3, 128.4 (2C), 128.1 (2C), 127.9 (2C), 126.3, 126.1, 125.7 (2C), 125.6, 124.6, 115.1, 112.8, 55.3, 51.8, 49.1, 47.9, 47.4, 37.9, 37.6, 29.6, 23.9, 22.9, 22.7.

NOESY- 2D (600 MHz, $CDCl_3$): between H-1 and Me, between H-2 and H-3, between H-4 and Me, between H^{PMP} and H-3, between H^{PMP} and H-2.

IR (film) ν_{max}: 2935, 1610, 1510, 1494, 1148, 1245, 1177, 1035, 908, 731, 702 cm^{-1}.

HRMS (APCI): calculated for $C_{35}H_{36}ONa$ $[M+Na]^+$ requires m/z 495.2658, found m/z 496.2656 (*Δ* = -0.47 ppm).

(±)-(1*R*,2*S*,4*aS*,10*aS*)-2-(Bis(4-methoxyphenyl)methyl)-1-(4-methoxyphenyl)-4*a*-methyl-1,2,3,4,4*a*,9,10,10*a*-octahydrophenanthrene 2n.

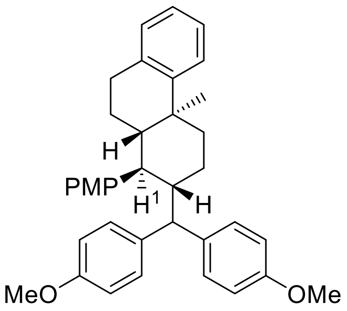

2n
$C_{37}H_{40}O_3$
532.72 g mol^{-1}

Diene **1** (30.6 mg 0.100 mmol, 1.00 equiv), bis(4-methoxyphenyl)methanol (24.4 mg, 0.100 mmol, 1.00 equiv) and Ti(O*i*Pr)$_4$ (9.0 µL, 30 µmol, 0.30 equiv) in HFIP (2.0 mL, 0.050 M) were subjected to the general procedure **B**. The crude product was purified by flash column chromatography (SiO_2; pentane:Et_2O; 41:9) to furnish compound **2n** as a pink oil which crystallised on standing (43.5 mg, 82%).[28]

^{1}H NMR (400 MHz, $CDCl_3$): δ 7.31 (d, *J* = 7.8 Hz, 1H), 7.24 (dd, *J* = 8.8, 2.1 Hz, 1H), 7.18 – 6.99 (m, 7H), 6.96 – 6.82 (m, 5H), 6.77 (d, *J* = 8.7 Hz, 2H), 3.88 – 3.81 (m, 7H), 3.77 (s, 3H), 2.78 – 2.57 (m, 2H), 2.51 (tt, *J* = 11.7, 3.4 Hz, 1H), 2.43 – 2.31 (m, 2H), 1.98 – 1.89 (m, 1H), 1.78 – 1.59 (m, 3H), 1.41 – 1.29 (m, 1H), 1.26 – 1.14 (m, 1H), 1.03 (s, 3H).

^{13}C NMR (101 MHz, $CDCl_3$): δ 158.0 (2C), 157.5, 147.9, 137.0, 136.2, 135.7, 134.0, 133.2, 131.8 (2C), 129.2 (3C), 126.1, 125.6 (2C), 124.6, 115.1, 113.4 (2C), 113.2 (2C), 112.8, 55.3 (3C), 50.1, 49.1, 47.9, 47.6, 37.9, 37.6, 29.6, 23.7, 22.9, 22.7.

NOESY- 2D (600 MHz, $CDCl_3$): between H-1 and Me.

M.p: 81-85 °C

IR (film) ν_{max}: 2933, 1609, 1509, 1463, 1244, 1176, 1036, 909, 830, 761, 731 cm^{-1}.

HRMS (ESI): calculated for $C_{37}H_{40}O_3Na$ $[M+Na]^+$ requires m/z 555.2870, found m/z 555.2871 (Δ = 0.16 ppm).

[28] Relative stereochemistry is determined by analogy to compound **2m**.

(±)-(1*R*,2*R*,4a*S*,10a*S*)-1-(4-Methoxyphenyl)-4a-methyl-2-(3-phenylcyclopent-2-en-1-yl)-1,2,3,4,4a,9,10,10a-octahydrophenanthrene 2o.

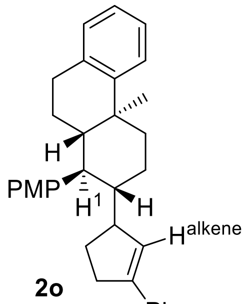

2o
$C_{33}H_{36}O$
448.65 g mol[-1]
*dr>95:5

Diene **1** (30.6 mg 0.100 mmol, 1.00 equiv), 1-phenylcyclopent-2-en-1-ol[29] (16.0 mg, 0.100 mmol, 1.00 equiv) and Ti(O*i*Pr)$_4$ (9.0 µL, 30 µmol, 0.30 equiv) in HFIP (2.0 mL, 0.050 M) were subjected to the general procedure **B** except that the reaction was performed at rt and for 4 h. The crude product was purified by flash column chromatography (SiO_2; 60 Å, 15–40 µm**;** pentane:Et_2O; 49:1) to furnish compound **2o** as a single detectable diastereomer as a pale brown foam (30.4 mg, 68%, >95:5 d.r.).[30]

^{1}H NMR (400 MHz, $CDCl_3$): δ 7.47 – 7.41 (m, 2H), 7.37 – 7.29 (m, 3H), 7.25 – 7.20 (m, 1H), 7.19 – 7.06 (m, 4H), 7.05 – 6.99 (m, 1H), 6.94 (dd, *J* = 8.6, 2.8 Hz, 1H), 6.80 (dd, *J* = 8.3, 2.8 Hz, 1H), 6.15 (d, *J* = 2.1 Hz, 1H), 3.81 (s, 3H), 2.81 – 2.48 (m, 6H), 2.44 – 2.35 (m, 1H), 1.98 – 1.88 (m, 1H), 1.87 – 1.79 (m, 1H), 1.79 – 1.52 (m, 5H), 1.47 – 1.35 (m, 1H), 1.31 – 1.23 (m, 1H), 1.18 (s, 3H).

^{13}C NMR (101 MHz, $CDCl_3$): δ 158.0, 148.1, 143.0, 137.0, 136.8, 135.8, 132.3, 129.3, 128.4 (2C), 127.0, 126.9, 126.1, 125.7 (3C), 125.6, 124.8, 115.3, 112.7, 55.4, 48.5, 48.4, 48.0, 47.9, 37.7, 37.6, 33.2, 29.6, 27.9, 22.9, 22.8, 22.7.

NOESY- 2D (600 MHz, $CDCl_3$): between H-1 and Me, between H-1 and H^{alkene}.

M.p: 85 °C

IR (film) ν_{max}: 2932, 1511, 1244, 1177, 1038, 908, 827, 757, 731, 693 cm^{-1}.

HRMS (APCI): calculated for $C_{33}H_{37}O$ $[M+H]^+$ requires m/z 449.2839, found m/z 449.2837 (*Δ* = -0.46 ppm).

29 Y. Zhu, I. Colomer, A. L. Thompson, T. J. Donohoe, *J. Am. Chem. Soc*. **2019**, *141*, 6489–6493.
30 The exocyclic stereochemistry could not be unambiguously determined.

(±)-(1*R*,2*R*,4*aS*,10*aS*)-2-((*S,E*)-1,3-Diphenylallyl)-1-(4-methoxyphenyl)-4*a*-methyl-1,2,3,4,4*a*,9,10,10*a*-octahydrophenanthrene (major)-2p and (±)-(1*R*,2*R*,4*aS*,10*aS*)-2-((*R,E*)-1,3-Diphenylallyl)-1-(4-methoxyphenyl)-4*a*-methyl-1,2,3,4,4*a*,9,10,10*a*-octahydrophenanthrene (minor)-2p.

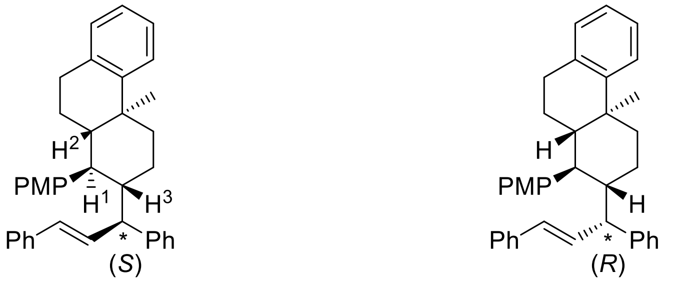

2p (major) $C_{37}H_{38}O$ 498.71 g mol^{-1} *dr = 83:17 **2p** (minor)

Diene **1** (30.6 mg 0.100 mmol, 1.00 equiv), (*E*)-1,3-diphenylprop-2-en-1-ol (21.0 mg, 0.100 mmol, 1.00 equiv) and Ti(O*i*Pr)$_4$ (9.0 µL, 30 µmol, 0.30 equiv) in HFIP (2.0 mL, 0.050 M) were subjected to the general procedure **B**. The crude product was purified by flash column chromatography (SiO_2; pentane:Et_2O; 49:1) to furnish compound **2p** as a an inseparable mixture of two diastereomers at the exocyclic benzylic position as a white foam (38.9 mg, 78%, 83:17 dr).[31,32]

Major diastereomer (from the mixture)**:**

^{1}H NMR (600 MHz, $CDCl_3$): δ 7.37 – 6.87 (m, 18H), 6.51 (dd, *J* = 15.8, 7.7 Hz, 1H), 6.22 (dd, *J* = 15.8, 1.3 Hz, 1H), 3.86 (s, 3H), 3.46 (dd, *J* = 7.7, 2.9 Hz, 1H), 2.79 – 2.61 (m, 2H), 2.45 – 2.40 (m, 1H), 2.32 (t, *J* = 11.1 Hz, 1H), 2.29 – 2.22 (m, 1H), 2.07 – 2.01 (m, 1H), 1.72 – 1.64 (m, 3H), 1.39 – 1.29 (m, 1H), 1.23 – 1.17 (m, 1H), 0.99 (s, 3H).

^{13}C NMR (101 MHz, $CDCl_3$): δ 158.1, 147.8, 141.6, 137.9, 136.1, 135.6, 133.9, 133.5, 129.7 (2C), 129.6, 129.2, 128.5 (2C), 128.0 (2C), 127.0, 126.4, 126.3, 126.2 (2C), 125.7, 125.6, 124.7, 115.2, 112.8, 55.3, 50.6, 49.1, 49.0, 47.5, 37.8, 37.5, 29.6, 23.9, 22.7, 22.5.

NOESY- 2D (600 MHz, $CDCl_3$): between H-1 and Me.

Note: the appearance of ^{1}H NMR signal for H-1: 2.32 (t, *J* = 11.1 Hz, 1H, H-1) is indicative of a trans-diaxial arrangement between H-1 and H-2; H-1 and H-3.

Characteristic peaks for the minor diastereomer (from the mixture):

---

[31] dr determined by ^{1}H NMR of the crude reaction mixture.
[32] Relative stereochemistry of the major and minor diastereomers is determined by analogy to compound **2g**.

^{1}H NMR (600 MHz, $CDCl_3$): δ 7.50 – 7.47 (m, 2H), 7.43 – 7.39 (m, 3H), 6.72 (dd, 1H, *J* = 15.7, 10.2 Hz), 6.31 (d, *J* = 15.7 Hz, 1H), 3.90 (s, 3H), 3.41 (dd, *J* = 10.2, 3.0 Hz, 1H), 1.21 (s, 3H).

^{13}C NMR (101 MHz, $CDCl_3$): δ 158.2, 144.9, 137.9, 135.7, 133.6, 133.0, 128.7, 128.2, 127.9, 127.6, 127.4, 126.1, 125.9, 115.3, 55.4, 50.0, 49.4, 48.3, 47.7, 37.6, 37.5, 22.8, 21.7.

M.p: 88 °C

IR (film) ν_{max}: 2933, 1510, 1244, 1177, 1037, 908, 760, 730, 699 cm^{-1}.

HRMS (APCI): calculated for $C_{37}H_{39}O$ $[M+H]^+$ requires m/z 499.2995, found m/z 499.2999 (*Δ* = 0.82 ppm).

(±)-(1*R*,2*R*,4a*S*,10a*S*)-1-(4-Methoxyphenyl)-2-((*E*)-3-(4-methoxyphenyl)allyl)-4*a*-methyl-1,2,3,4,4*a*,9,10,10*a*-octahydrophenanthrene 2q.

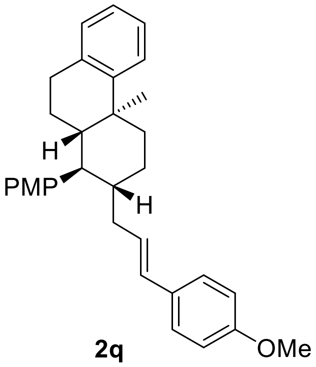

2q
$C_{32}H_{36}O_2$
452.64 g mol^{-1}

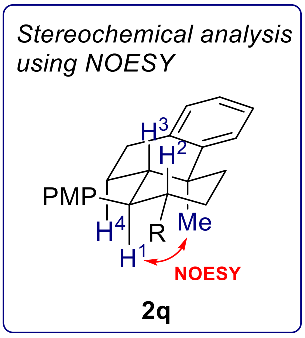

Diene **1** (30.6 mg, 0.100 mmol, 1.00 equiv), (*E*)-3-(4-methoxyphenyl)prop-2-en-1-ol[33] (16.4 mg, 0.100 mmol, 1.00 equiv) and $Ti(OiPr)_4$ (9.0 µL, 30 µmol, 0.30 equiv) in HFIP (2.0 mL, 0.050 M) were subjected to the general procedure **B**. The crude product was purified by flash column chromatography (SiO_2; pentane:Et_2O; 24:1) to furnish compound **2q** as a colourless oil (18.5 mg, 41%).

^{1}H NMR (400 MHz, $CDCl_3$): δ 7.34 (dd, *J* = 7.8, 1.4 Hz, 1H), 7.24 (d, *J* = 8.7 Hz, 2H), 7.18 – 6.99 (m, 5H), 6.97 – 6.89 (m, 1H), 6.87 – 6.77 (m, 3H), 6.17 (d, *J* = 15.7 Hz, 1H), 5.95 (ddd, *J* = 15.8, 8.2, 6.4 Hz, 1H), 3.83 (s, 3H), 3.80 (s, 3H), 2.80 – 2.60 (m, 2H), 2.44 – 2.36 (m, 1H), 2.32 (t, *J* = 11.0 Hz, 1H), 2.06 (dddd, *J* = 13.5, 6.1, 3.2, 1.5 Hz, 1H), 2.00 – 1.89 (m, 1H), 1.86 – 1.53 (m, 5H), 1.47 – 1.33 (m, 1H), 1.30 – 1.22 (m, 1H), 1.20 (s, 3H).

[33] Y. Zhu, I. Colomer, A. L. Thompson, T. J. Donohoe, *J. Am. Chem. Soc.* **2019**, *141*, 6489–6493.

^{13}C NMR (101 MHz, $CDCl_3$): δ 158.8, 158.0, 148.1, 136.5, 135.7, 132.5, 130.9, 130.6, 129.3, 127.1 (3C), 126.0, 125.7, 125.6, 124.8, 115.1, 114.0 (2C), 112.7, 55.4, 55.3, 49.8, 47.7, 44.2, 38.2, 37.7, 37.5, 29.6, 27.9, 22.7, 22.6.

NOESY- 2D (600 MHz, $CDCl_3$): between H-1 and Me.

Note: the appearance of ^{1}H NMR signal for H-1: 2.32 (t, J = 11.0 Hz, 1H, H-1) is indicative of a trans-diaxial arrangement between H-1 and H-2; H-1 and H-3.

IR (film) ν_{max}: 2925, 1608, 1510, 1464, 1245, 1176, 1037, 966, 909, 833, 760, 732 cm^{-1}.

HRMS (APCI): calculated for $C_{32}H_{37}O_2$ $[M+H]^+$ requires m/z 453.2788, found m/z 453.2785 (Δ = -0.69 ppm).

(±)-4-((*E*)-3-((1*R*,2*R*,4*aS*,10*aS*)-1-(4-Methoxyphenyl)-4*a*-methyl-1,2,3,4,4*a*,9,10,10*a*-octahydrophenanthren-2-yl)prop-1-en-1-yl)phenol 2r.

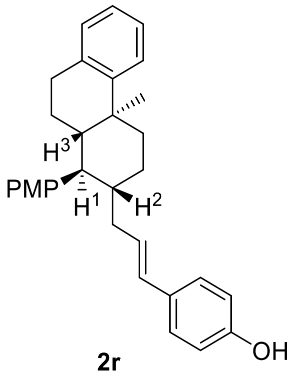

2r
$C_{31}H_{34}O_2$
438.61 g mol^{-1}

Diene **1** (30.6 mg, 0.100 mmol, 1.00 equiv), (*E*)-3-(4-methoxyphenyl)prop-2-en-1-ol[34] (15.0 mg, 0.100 mmol, 1.00 equiv) and $Ti(OiPr)_4$ (9.0 µL, 30 µmol, 0.30 equiv) in HFIP (2.0 mL, 0.050 M) were subjected to the general procedure **B**. The crude product was purified by flash column chromatography (SiO_2; pentane:Et_2O; 7:3) to furnish compound **2r** as a colourless oil (17.3 mg, 39%).[35]

^{1}H NMR (400 MHz, CD_2Cl_2): δ 7.33 (dd, J = 7.9, 1.4 Hz, 1H), 7.22 – 6.97 (m, 7H), 6.93 (dd, J = 8.5, 2.8 Hz, 1H), 6.82 (dd, J = 8.2, 2.9 Hz, 1H), 6.74 (d, J = 8.6 Hz, 2H), 6.15 (d, J = 15.7 Hz, 1H), 5.95 (ddd, J = 15.7, 8.1, 6.4 Hz, 1H), 5.02 – 4.86 (m, 1H), 3.80 (s, 3H), 2.79 – 2.58

[34] C. Han, Z. Huang, C. Zheng, L. Wan, Y. Lai, S. Peng, K. Ding, H. Ji, Y. Zhang, *Eur. J. of Med. Chem*. **2013**, *66*, 82–90.
[35] Relative stereochemistry is determined by analogy to compound **2q**.

(m, 2H), 2.39 (dd, J = 9.3, 3.2 Hz, 1H), 2.33 (t, J = 11.0 Hz, 1H), 2.07 – 1.99 (m, 1H), 1.97 – 1.90 (m, 1H), 1.86 – 1.53 (m, 5H), 1.46 – 1.33 (m, 1H), 1.26 – 1.14 (m, 4H).

^{13}C NMR (101 MHz, CD_2Cl_2): δ 158.4, 155.1, 148.5, 136.9, 136.1, 132.7, 131.3, 130.6, 129.5, 127.5 (2C), 127.4, 126.4, 125.9, 125.7, 125.0, 115.6 (2C), 115.3, 113.0, 55.6, 50.0, 48.0, 44.4, 38.4, 38.0, 37.8, 29.9, 28.2, 22.9, 22.8.

Note: the appearance of ^{1}H NMR signal for H-1: 2.33 (t, J = 11.0 Hz, 1H, H-1) is indicative of a trans-diaxial arrangement between H-1 and H-2; H-1 and H-3.

IR (film) ν_{max}: 3386, 2917, 2849, 1610, 1511, 1241, 1177, 908, 731 cm^{-1}

HRMS (ESI): calculated for $C_{31}H_{33}O_2$ $[M\text{-}H]^-$ requires m/z 437.2486, found m/z 437.2470 (Δ = -3.78 ppm).

(±)-(1*R*,2*R*,4*aS*,10*aS*)-1-(4-Methoxyphenyl)-4*a*-methyl-2-(3-methylbut-2-en-1-yl)-1,2,3,4,4*a*,9,10,10*a*-octahydrophenanthrene 2s.

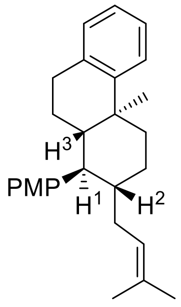

2s
$C_{27}H_{34}O$
374.57 g mol^{-1}

Diene **1** (30.6 mg, 0.100 mmol, 1.00 equiv), 3-methylbut-2-en-1-ol (17.2 mg, 0.200 mmol, 2.00 equiv) and $Ti(OiPr)_4$ (9.0 µL, 30 µmol, 0.30 equiv) in HFIP (2.0 mL, 0.050 M) were subjected to the general procedure **B** except that the reaction was performed with 2.00 equiv of alcohol and for 50 min. The crude product was purified by flash column chromatography (SiO_2; 60 Å, 15–40 µm**;** pentane:Et_2O; 97:3) to furnish compound **2s** as a single detectable diastereomer as a colourless oil (7.0 mg, 19%).[36]

^{1}H NMR (600 MHz, $CDCl_3$): δ 7.33 (dd, J = 7.8, 1.4 Hz, 1H), 7.18 – 7.05 (m, 3H), 7.05 – 6.94 (m, 2H), 6.93 – 6.85 (m, 1H), 6.84 – 6.75 (m, 1H), 5.05 (ddt, J = 8.1, 6.5, 1.5 Hz, 1H), 3.81

[36] Relative stereochemistry is determined by analogy to compound **2q**.

(s, 3H), 2.78 – 2.58 (m, 2H), 2.36 (dt, *J* = 12.2, 3.1 Hz, 1H), 2.27 (t, *J* = 10.8 Hz, 1H), 1.94 – 1.85 (m, 1H), 1.84 – 1.76 (m, 1H), 1.75 – 1.15 (m, 16H).

^{13}C NMR (101 MHz, $CDCl_3$): δ 157.9, 148.3, 136.8, 135.8, 132.4, 132.1, 129.3, 126.0, 125.7, 125.5, 124.8, 122.9, 115.0, 112.7, 55.4, 49.8, 47.7, 44.6, 37.8, 37.6, 33.0, 29.6, 27.7, 26.0, 22.8, 22.7, 17.9.

Note: the appearance of ^{1}H NMR signal for H-1: 2.27 (t, *J* = 10.8 Hz, 1H, H-1) is indicative of a trans-diaxial arrangement between H-1 and H-2; H-1 and H-3.

IR (film) ν_{max}: 2925, 1611, 1511,1450, 1246, 1177, 1040, 909, 827, 733 cm^{-1}.

HRMS (ESI): calculated for $C_{27}H_{35}O$ $[M+H]^+$ requires m/z 375.2682, found m/z 375.2682 (*Δ* = -0.05 ppm).

(±)-(1*S*,2*S*,4*aS*,10*aR*)-2-Benzhydryl-1-(4-methoxyphenyl)-1,2,3,4,4*a*,9,10,10*a*-octahydrophenanthrene 2t.

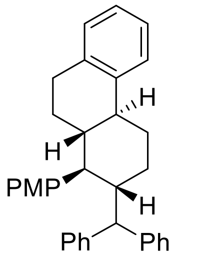

2t
$C_{34}H_{34}O$
458.65 g mol^{-1}

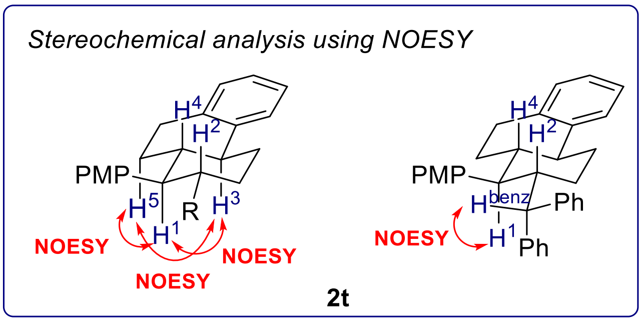

Diene **3** (29.2 mg, 0.100 mmol, 1.00 equiv), benzhydrol (18.4 mg, 0.100 mmol, 1.00 equiv) and Ti(O*i*Pr)$_4$ (9.0 µL, 30 µmol, 0.30 equiv) in HFIP (2.0 mL, 0.050 M) were subjected to the general procedure **B**. The crude product was purified by flash column chromatography (SiO_2; 60 Å, 15–40 µm; pentane:Et_2O; 24:1) to furnish compound **2t** as a colourless foam (24.6 mg, 54%).

^{1}H NMR (400 MHz, $CDCl_3$): δ 7.35 – 7.20 (m, 7H), 7.17 – 7.06 (m, 8H), 7.02 (dd, *J* = 7.2, 1.6 Hz, 1H), 6.94 – 6.86 (m, 2H), 3.99 (d, *J* = 2.9 Hz, 1H), 3.84 (s, 3H), 2.72 – 2.53 (m, 4H), 2.44 – 2.33 (m, 1H), 2.27 – 2.13 (m, 2H), 1.60 – 1.41 (m, 4H), 1.30 – 1.15 (m, 1H).

^{13}C NMR (101 MHz, $CDCl_3$): δ 158.1, 144.8, 141.7, 140.4, 137.1, 135.9, 131.2 (2C), 129.0, 128.4 (2C), 128.1 (2C), 127.8 (2C), 126.3, 125.7 (3C), 125.6, 114.0 (2C), 55.4, 53.2, 51.6, 47.6, 46.7, 43.6, 31.0, 30.2, 28.8, 27.8.[37]

NOESY- 2D (600 MHz, $CDCl_3$): between H-1 and H-5; between H-1 and H-3; between H-3 and H-5; between H-1 and H^{benz}.

IR (film) ν_{max}: 2934, 1610, 1511, 1494, 1450, 1246, 1177, 1035, 909, 826, 732, 702 cm^{-1}.

M.p: 136-139 °C

HRMS (ESI): calculated for $C_{34}H_{34}ONa$ $[M+Na]^+$ requires m/z 481.2502, found m/z 481.2503 (*Δ* = 0.18 ppm).

(±)-4-((*E*)-3-((1*R*,2*R*,4*aS*,10*aR*)-1-(4-Methoxyphenyl)-1,2,3,4,4*a*,9,10,10*a*-octahydrophenanthren-2-yl)prop-1-en-1-yl)phenol 2u.

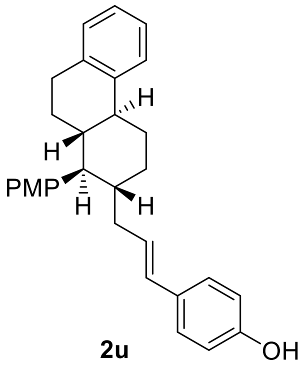

2u
$C_{30}H_{32}O_2$
424.58 g mol^{-1}

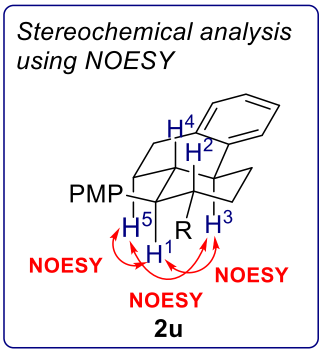

Diene **3** (29.2 mg, 0.100 mmol, 1.00 equiv), (*E*)-4-(3-hydroxyprop-1-en-1-yl)phenol[38] (15.0 mg, 0.100 mmol, 1.00 equiv) and $Ti(OiPr)_4$ (9.0 µL, 30 µmol, 0.30 equiv) in HFIP (2.0 mL, 0.050 M) were subjected to the general procedure **B**. The crude product was purified by flash column chromatography (SiO_2; 60 Å, 15–40 µm; pentane:Et_2O; 3:2) to furnish compound **2u** as a colourless oil (16.5 mg, 39%).

^{1}H NMR (400 MHz, $CDCl_3$): δ 7.35 (d, *J* = 7.7 Hz, 1H), 7.22 – 7.01 (m, 7H), 6.96 – 6.82 (m, 2H), 6.79 – 6.72 (m, 2H), 6.14 (d, *J* = 15.7 Hz, 1H), 5.94 (ddd, *J* = 15.7, 7.9, 6.4 Hz, 1H), 4.75 (br, 1H), 3.83 (s, 3H), 2.76 – 2.64 (m, 2H), 2.63 – 2.54 (m, 1H), 2.54 – 2.43 (m, 1H), 2.22 – 1.98 (m, 3H), 1.84 – 1.67 (m, 2H), 1.60 – 1.33 (m, 4H), 1.32 – 1.18 (m, 1H).

[37] Two PMP carbons are missing due to coalescence in the ^{13}C NMR spectrum.
[38] C. Han, Z. Huang, C. Zheng, L. Wan, Y. Lai, S. Peng, K. Ding, H. Ji, Y. Zhang, *Eur. J. Med. Chem*. **2013**, *66*, 82–90.

^{13}C NMR (101 MHz, $CDCl_3$): δ 158.0, 154.6, 140.5, 137.2, 136.4, 131.1, 130.5, 129.0, 127.3 (2C), 127.1, 125.7 (3C), 115.5 (2C), 55.4, 55.2, 46.2, 43.8, 43.7, 38.1, 32.4, 30.8, 30.2, 27.8.[39]

NOESY- 2D (600 MHz, $CDCl_3$): between H-1 and H-3; between H-3 and H-5; between H-1 and H-5.

HRMS (ESI): calculated for $C_{30}H_{33}O_2$ $[M+H]^+$ requires m/z 425.2475, found m/z 425.2475 (Δ = -0.09 ppm).

(±)-Methyl 2-hydroxy-5-((*S*)-1-((1*R*,2*R*,4*aS*,10*aS*)-1-(4-methoxyphenyl)-4*a*-methyl-1,2,3,4,4*a*,9,10,10*a*-octahydrophenanthren-2-yl)ethyl)benzoate (major)-2v and (±)-Methyl 2-hydroxy-5-((*R*)-1-((1*R*,2*R*,4*aS*,10*aS*)-1-(4-methoxyphenyl)-4*a*-methyl-1,2,3,4,4*a*,9,10,10*a*-octahydrophenanthren-2-yl)ethyl)benzoate (minor)-2v.

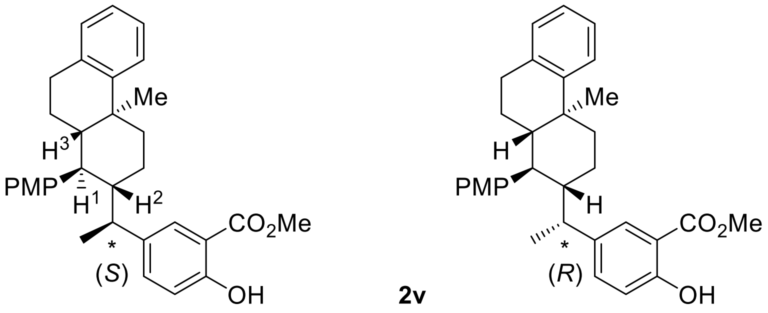

2v
$C_{32}H_{36}O_4$
484.64 g mol^{-1}
*dr = 86:14

Diene **3** (30.6 mg, 0.100 mmol, 1.00 equiv), methyl 2-hydroxy-5-(1-hydroxyethyl)benzoate **S12** (19.6 mg, 0.100 mmol, 1.00 equiv) and Ti(O*i*Pr)$_4$ (9.0 µL, 30 µmol, 0.30 equiv) in HFIP (2.0 mL, 0.050 M) were subjected to the general procedure **B** except the reaction was performed overnight. The crude product was purified by flash column chromatography (SiO_2; pentane:Et_2O; 83:17) to furnish compound **2v** as an inseparable mixture of two diastereomers as a colourless oil (43.5 mg, 90%, 86:14 dr).[40,41]

Major diastereomer (from the mixture)**:**

^{1}H NMR (400 MHz, $CDCl_3$): δ 10.60 (s, 1H), 7.51 (d, *J* = 2.3 Hz, 1H), 7.29 (dd, *J* = 7.9, 1.5 Hz, 1H), 7.23 (dd, *J* = 8.6, 2.3 Hz, 1H), 7.16 – 6.84 (m, 8H), 4.00 (s, 3H), 3.85 (s, 3H), 2.75 – 2.52

---

[39] Four PMP carbons are missing due to the coalescence in the ^{13}C NMR spectrum.
[40] dr determined by ^{1}H NMR after column chromatography due to the overlapping signals in the ^{1}H spectrum of the crude mixture.
[41] Relative stereochemistry of the major and minor diastereomers is determined by analogy to compound **2g**.

(m, 3H), 2.40 (dt, J = 12.8, 3.5 Hz, 1H), 2.15 (t, J = 11.2 Hz, 1H), 1.95 (dq, J = 13.1, 3.6 Hz, 1H), 1.87 (tt, J = 11.8, 3.2 Hz, 1H), 1.66 – 1.54 (m, 2H), 1.46 – 1.08 (m, 6H), 0.91 (s, 3H).

^{13}C NMR (101 MHz, $CDCl_3$): δ 170.9, 159.8, 158.0, 147.8, 136.4, 136.3, 135.7, 134.4, 133.3, 129.9, 129.2, 126.3, 125.7, 125.5, 124.7, 116.7, 114.8, 112.9, 111.5, 55.3, 52.4, 49.1, 48.9, 47.5, 39.1, 37.7, 37.4, 29.6, 22.7, 22.5, 22.2, 19.6.

Note: the appearance of ^{1}H NMR signal for H-1: 2.15 (t, J = 11.2 Hz, 1H, H-1) is indicative of a trans-diaxial arrangement between H-1 and H-2; H-1 and H-3.

Characteristic peaks for the minor diastereomer (from the mixture):

^{1}H NMR (400 MHz, $CDCl_3$): δ 3.95 (s, 3H).

^{13}C NMR (101 MHz, $CDCl_3$): δ 137.5, 135.7, 132.0, 127.9, 117.2, 115.5, 112.8, 50.3, 48.2, 47.6, 38.0, 37.6, 20.1, 11.5.

IR (film) $ν_{max}$: 3203, 2935, 1677, 1511, 1490, 1441, 1248, 1212, 1039, 909, 735 cm^{-1}.

HRMS (ESI): calculated for $C_{32}H_{37}O_4$ $[M+H]^+$ requires m/z 485.2686, found m/z 485.2684 (Δ = -0.54 ppm).

(±)-(1*R*,2*R*,4a*S*,10a*S*)-2-(3-(4-chlorophenyl)cyclopent-2-en-1-yl)-1-(4-methoxyphenyl)-4a-methyl-1,2,3,4,4a,9,10,10a-octahydrophenanthrene 2w.

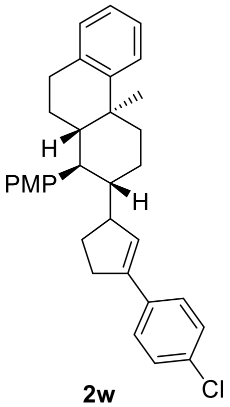

2w
$C_{33}H_{35}ClO$
483.09 g mol^{-1}
*dr>95:5

Diene **1** (30.6 mg, 0.100 mmol, 1.00 equiv), 1-(4-chlorophenyl)cyclopent-2-en-1-ol **S13** (19.5 mg, 0.100 mmol, 1.00 equiv) and $Ti(OiPr)_4$ (9.0 µL, 30 µmol, 0.30 equiv) in HFIP (2.0 mL, 0.050 M) were subjected to the general procedure **B** except that the reaction was performed at rt and for 1.5 h. The crude product was purified by flash column chromatography (SiO_2; 60

Å, 15–40 μm; pentane:Et_2O; 24:1) to furnish compound **2w** as a single detectable diastereomer as a bage foam (37.4 mg, 77%, >95:5 d.r.).[42]

^{1}H NMR (400 MHz, $CDCl_3$): δ 7.39 – 7.22 (m, 5H), 7.20 – 6.99 (m, 5H), 6.95 (dd, *J* = 8.7, 2.8 Hz, 1H), 6.80 (dd, J = 8.2, 2.8 Hz, 1H), 6.17 – 6.08 (m, 1H), 3.81 (s, 3H), 2.82 – 2.60 (m, 4H), 2.59 – 2.45 (m, 2H), 2.40 (dt, *J* = 12.2, 3.3 Hz, 1H), 1.99 – 1.88 (m, 1H), 1.84 (dq, *J* = 13.2, 3.3 Hz, 1H), 1.79 – 1.35 (m, 6H), 1.32 – 1.23 (m, 1H), 1.19 (s, 3H).

^{13}C NMR (101 MHz, $CDCl_3$): δ 158.0, 148.0, 141.8, 136.7, 135.7, 135.4, 132.5, 132.2, 129.3, 128.4 (2C), 127.8, 127.0 (2C), 126.1, 125.7, 125.6, 124.8, 115.3, 112.7, 55.3, 48.4, 48.2 (2C), 47.9, 37.6, 37.5, 33.1, 29.6, 27.8, 23.1, 22.8, 22.6.

IR (film) v_{max}: 2937, 1511, 1491, 1245, 1177, 1093, 1038, 909, 818, 734 cm^{-1}.

M.p: 75-78 °C

HRMS (ESI): calculated for $C_{33}H_{36}OCl$ $[M+H]^+$ requires m/z 483.2449, found m/z 483.2451 (Δ = 0.36 ppm).

*4.7 Cyclisation cascade methodology: synthesis of **6a-6f**.*

General procedure C.

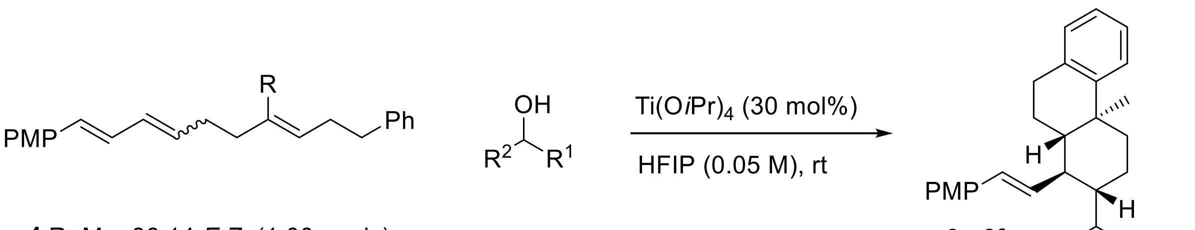

4 R=Me; 86:14 *E*:*Z;* (1.00 equiv)
5 R=H; 60:40 *E*:*Z;* (1.00 equiv)

(1.00 eq)

6a-6f
(Major dr)

Triene **4** (1.00 equiv, 86:14 *E*:*Z*) or **5** (1.00 equiv, 53:47 *E*:*Z)* and the corresponding alcohol (1.00 equiv) were transferred to a microwave vial which was then purged with nitrogen. The balloon of nitrogen was removed and HFIP followed by a stock solution of Ti(O*i*Pr)$_4$ in HFIP (0.30 equiv, 0.066 M) were added to obtain an overall 0.050 M solution of a polyene substrate. The solution was stirred at rt for 1 h, then diluted with water (3 mL). The obtained suspension was transferred to a separatory funnel and extracted with CH_2Cl_2 (3 × 4 mL). The organic layers were combined, washed with brine and volatiles were removed *in vacuo*. Crude product was purified by flash column chromatography using the appropriate mixture of eluents.

[42] The exocyclic stereochemistry could not be unambiguously determined.

(±)-(1*R*,2S,4aS,10aS)-2-Benzhydryl-1-((E)-4-methoxystyryl)-4a-methyl-1,2,3,4,4a,9,10,10a-octahydrophenanthrene (major)-6a and (±)-(1*S*,2S,4aS,10aS)-2-Benzhydryl-1-((*E*)-4-methoxystyryl)-4a-methyl-1,2,3,4,4a,9,10,10a-octahydrophenanthrene (minor)-6a.

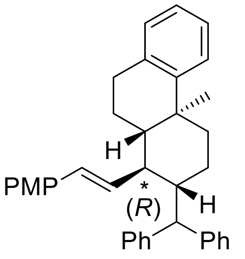

6a (major)

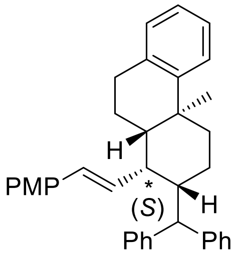

6a (minor)

$C_{37}H_{38}O$
498.7100 g mol^{-1}
*dr = 60:40

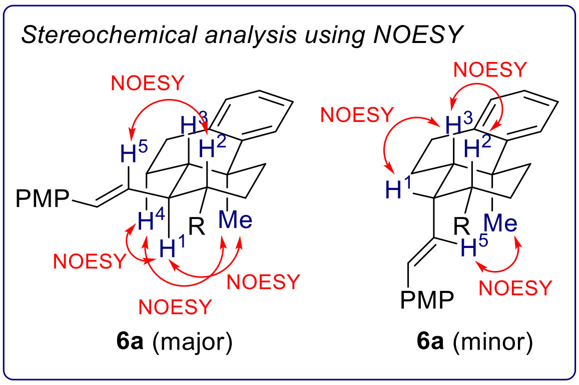

Triene **4** (33.2 mg, 0.100 mmol, 1.00 equiv), benzhydrol (18.4 mg, 0.100 mmol, 1.00 equiv) and Ti(O*i*Pr)$_4$ (9.0 µL, 30 µmol, 0.30 equiv) in HFIP (2.0 mL, 0.050 M) were subjected to the general procedure **C**. The crude product was purified by flash column chromatography (SiO$_2$; pentane:Et$_2$O; 24:1) to furnish compound **6a** as an inseparable mixture of two diastereomers at the exocyclic allylic position as a colourless oil (33.2 mg, 67%, 60:40 dr).[43]

^{1}H NMR (600 MHz, CDCl$_3$): δ 7.36 – 7.03 (m, 16H$_{maj}$, 16H$_{min}$), 6.92 (d, *J* = 8.6 Hz, 2H$_{min}$), 6.88 (d, *J* = 8.6 Hz, 2H$_{maj}$), 6.32 – 6.23 (m, 1H$_{maj}$, 1H$_{min}$), 5.78 – 5.68 (m, 1H$_{maj}$, 1H$_{min}$), 4.42 (d, *J* = 3.9 Hz, 1H$_{maj}$), 3.85 (s, 3H$_{min}$), 3.84 (s, 3H$_{maj}$), 3.66 (d, *J* = 11.8 Hz, 1H$_{min}$), 2.97 – 2.77 (m, 2H$_{maj}$, 2H$_{min}$), 2.51 (tt, *J* = 11.5, 4.0 Hz, 1H$_{min}$), 2.42 (dt, J = 9.3, 4.2 Hz, 1H$_{min}$), 2.39 – 2.32 (m, 1H$_{maj}$, 1H$_{min}$), 2.32 – 2.24 (m, 1H$_{maj}$), 2.14 (q, *J* = 10.2 Hz, 1H$_{maj}$), 1.94 (ddt, *J* = 13.0, 6.1, 2.8 Hz, 1H$_{maj}$), 1.89 – 1.76 (m, 1H$_{maj}$, 2H$_{min}$), 1.67 – 1.52 (m, 3H$_{maj}$, 3H$_{min}$), 1.50 – 1.41 (m, 1H$_{maj}$, 1H$_{min}$), 1.24 (s, 3H$_{min}$), 1.00 (s, 3H$_{maj}$).

^{13}C NMR (151 MHz, CDCl$_3$) **for the major diastereomer** (from the mixture): δ 159.0, 147.8, 144.9, 142.5, 135.7, 132.2 (2C), 130.6 (2C), 130.5, 129.3, 128.7 (2C), 128.3 (2C), 128.0 (2C), 127.3 (2C), 126.2, 125.7 (2C), 125.6, 124.7, 114.1 (2C), 55.5, 53.1, 47.1, 46.8, 46.5, 37.8, 37.1, 29.7, 24.3, 23.1, 22.9. **Selected peaks for the minor diastereomer** (from the mixture): 159.0, 149.2, 144.6, 143.4, 135.5, 132.6, 131.0, 129.2, 128.7, 128.2, 127.3, 126.1 (2C), 125.8, 125.6, 125.5, 124.5, 114.1, 56.4, 47.4, 46.4, 45.2, 38.8, 37.4, 29.8, 26.1, 24.7, 23.7.

NOESY- 2D (600 MHz, CDCl$_3$): between H-1$_{maj}$ and Me$_{maj}$, between H-1$_{maj}$ and H-4$_{maj}$, between H-4$_{maj}$ and Me$_{maj}$, between H-5$_{maj}$ and H-2$_{maj}$, between H-1$_{maj}$ and H^{benz};

[43] dr determined by ^{1}H NMR of the crude reaction mixture.

Between H-5$_{min}$ and Me$_{min}$, between H-1$_{min}$ and H-3$_{min}$, between H-3$_{min}$ and H-2$_{min}$.

IR (film) ν_{max}: 3026, 2933, 2361, 1607, 1510, 1492, 1450, 1295,1250, 1174, 1034, 970, 761, 737, 704 cm^{-1}.

HRMS (ESI): calculated for $C_{37}H_{39}O$ $[M+H]^+$ requires m/z 499.2995, found m/z 499.2992 (Δ = -0.70 ppm).

(±)-(1*R*,2*S*,4*aS*,10*aS*)-2-(Bis(4-methoxyphenyl)methyl)-1-((*E*)-4-methoxystyryl)-4*a*-methyl-1,2,3,4,4*a*,9,10,10*a*-octahydrophenanthrene (major)-6b and (±)-(1*S*,2*S*,4*aS*,10*aS*)-2-(Bis(4-methoxyphenyl)methyl)-1-((*E*)-4-methoxystyryl)-4*a*-methyl-1,2,3,4,4*a*,9,10,10*a*-octahydrophenanthren (minor)-6b.

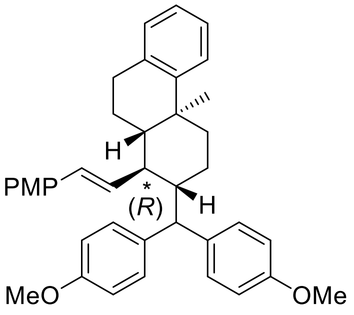

6b (major)

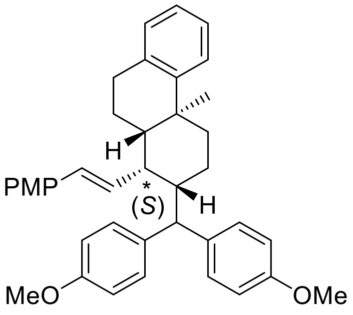

6b (minor)

$C_{39}H_{42}O_3$
558.76 g mol^{-1}
*dr = 70:30

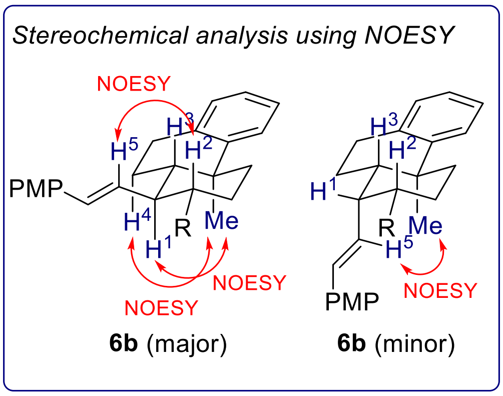

Triene **4** (33.2 mg, 0.100 mmol, 1.00 equiv), bis(4-methoxyphenyl)methanol (24.4 mg, 0.100 mmol, 1.00 equiv) and Ti(O*i*Pr)$_4$ (9.0 µL, 30 µmol, 0.30 equiv) in HFIP (2.0 mL, 0.050 M) were subjected to the general procedure **C**. The crude product was purified by flash column chromatography (SiO_2; pentane:Et_2O; 22:3) to furnish compound **6b** as an inseparable mixture of two diastereomers at the exocyclic allylic position as a colourless oil (52.0 mg, 93%, 70:30 dr).[44,45.]

^{1}H NMR (600 MHz, $CDCl_3$): δ 7.37 – 7.28 (m, 3H$_{maj}$, 3H$_{min}$), 7.25 – 7.04 (m, 7H$_{maj}$, 7H$_{min}$), 6.98 – 6.79 (m, 6H$_{maj}$, 6H$_{min}$), 6.33 – 6.22 (m, 1H$_{maj}$, 1H$_{min}$), 5.83 – 5.70 (m, 1H$_{maj}$, 1H$_{min}$), 4.33 (d, *J* = 3.9 Hz, 1H$_{maj}$), 3.89 – 3.83 (m, 6H$_{maj}$, 3H$_{min}$), 3.82 (s, 3H$_{min}$), 3.79 – 3.75 (m, 3H$_{maj}$, 3H$_{min}$), 3.58 (d, *J* = 11.7 Hz, 1H$_{min}$), 2.99 – 2.79 (m, 2H$_{maj}$, 2H$_{min}$), 2.46 – 2.32 (m, 1H$_{maj}$, 3H$_{min}$), 2.26 – 2.18 (m, 1H$_{maj}$), 2.12 (q, *J* = 10.2 Hz, 1H$_{maj}$), 1.95 (ddt, *J* = 12.7, 6.2, 2.8 Hz, 1H$_{maj}$), 1.89 –

[44] dr determined by ^{1}H NMR of the crude reaction mixture.
[45] Relative stereochemistry of **6b** (minor) is assigned by analogy to **6a** (minor).

1.77 (m, 1H$_{maj}$, 2H$_{min}$), 1.68 – 1.52 (m, 3H$_{maj}$, 3H$_{min}$), 1.51 – 1.42 (m, 1H$_{maj}$, 1H$_{min}$), 1.25 (s, 3H$_{min}$), 1.02 (s, 3H$_{maj}$).

^{13}C NMR (101 MHz, $CDCl_3$) **for the major diastereomer** (from the mixture): δ 158.9, 157.9, 157.5, 147.8, 137.2, 135.7, 134.8, 132.4, 132.0, 131.4 (2C), 130.6, 129.4 (2C), 129.3, 127.2 (2C), 125.6, 125.5, 124.7, 114.0 (2C), 113.6 (2C), 113.3 (2C), 55.5, 55.3 (2C), 51.4, 46.9, 46.7 (2C), 37.8, 37.1, 29.7, 24.0, 23.1, 22.9. **Selected peaks for the minor diastereomer** (from the mixture): 158.9, 157.8 (2C), 149.2, 136.0, 135.5, 132.5, 131.0, 129.3, 129.2, 128.8, 127.3, 125.8 (2C), 125.4, 124.5, 114.1, 114.0, 113.6, 54.5, 47.4, 46.4, 45.6, 38.8, 37.4, 29.8, 26.0, 24.7, 23.7.

NOESY- 2D (600 MHz, $CDCl_3$): between H-1$_{maj}$ and Me$_{maj}$, between H-5$_{maj}$ and H-2$_{maj}$, between H-4$_{maj}$ and Me$_{maj}$; Between H-5$_{min}$ and Me$_{min}$.

Note: the appearance of ^{1}H NMR signal for H-1$_{maj}$: 2.12 (q, *J* = 10.2 Hz, 1H, H-1) is indicative of a trans-diaxial arrangement between H-1$_{maj}$ and H-2$_{maj}$; H-1$_{maj}$ and H-3$_{maj}$.

IR (film) $ν_{max}$: 2932, 2361, 2341, 1608, 1509, 1298, 1247, 1176, 1035, 835 cm^{-1}.

HRMS (ESI): calculated for $C_{39}H_{43}O_3$ $[M+H]^+$ requires m/z 559.3207, found m/z 559.3204 (*Δ* = -0.42 ppm).

(±)-(1*S*,2*R*,4*aS*,10*aS*)-2-((*E*)-3-(4-Methoxyphenyl)allyl)-1-((*E*)-4-methoxystyryl)-4$_{a}$-methyl-1,2,3,4,4*a*,9,10,10*a*-octahydrophenanthrene 6c.

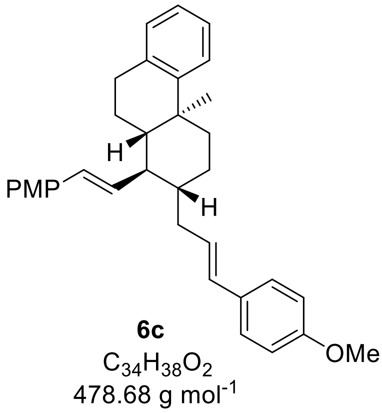

6c
$C_{34}H_{38}O_2$
478.68 g mol^{-1}

Triene **4** (33.2 mg, 0.100 mmol, 1.00 equiv), (*E*)-3-(4-methoxyphenyl)prop-2-en-1-ol[46] (16.4 mg, 0.100 mmol, 1.00 equiv) in HFIP (2.0 mL, 0.050 M) were subjected to the general procedure **C** except the reaction was performed overnight and without Ti(O*i*Pr)$_4$. The crude

[46] Y. Zhu, I. Colomer, A. L. Thompson, T. J. Donohoe, *J. Am. Chem. Soc*. **2019**, *141*, 6489–6493.

product was purified by flash column chromatography (SiO_2; 60 Å, 15–40 µm; pentane:Et_2O; 23:2) to furnish compound **6c** as a colourless oil (12.7 mg, 27%).[47]

^{1}H NMR (400 MHz, $CDCl_3$): δ 7.38 – 7.24 (m, 5H), 7.17 – 7.02 (m, 3H), 6.91 – 6.81 (m, 4H), 6.39 – 6.26 (m, 2H), 6.09 (ddd, *J* = 15.3, 8.1, 6.3 Hz, 1H), 5.74 (dd, *J* = 15.8, 9.4 Hz, 1H), 3.83 (s, 3H), 3.81 (s, 3H), 2.90 – 2.74 (m, 2H), 2.54 – 2.43 (m, 1H), 2.39 – 2.27 (m, 1H), 2.09 – 1.79 (m, 4H), 1.54 – 1.33 (m, 5H), 1.14 (s, 3H).

^{13}C NMR (101 MHz, $CDCl_3$) δ 158.9, 158.8, 148.0, 135.8, 132.3, 131.4, 131.0, 130.7, 130.6, 129.3, 127.2 (3C), 127.1 (2C), 125.7, 125.6, 124.9, 114.1 (4C), 55.5, 55.4, 48.1, 46.3, 42.6, 38.4, 37.6, 37.1, 29.8, 27.4, 23.0 (2C).

IR (film) ν_{max}: 2931, 1607, 1510, 1294, 1248, 1175, 1036, 967, 909, 761, 730 cm^{-1}.

HRMS (ESI): calculated for $C_{34}H_{39}O_2$ $[M+H]^+$ requires m/z 479.2945, found m/z 479.2944 (*Δ* = -0.12 ppm).

(±)-(1*S*,2*R*,4*aS*,10*aS*)-2-((*E*)-3-(4-Methoxyphenyl)allyl)-1-((*E*)-4-methoxystyryl)-1,2,3,4,4*a*,9,10,10*a*-octahydrophenanthrene 6d.

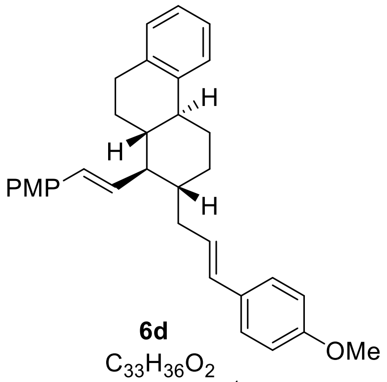

6d
$C_{33}H_{36}O_2$
464.65 g mol^{-1}

Triene **5** (31.8 mg, 0.100 mmol, 1.00 equiv), (*E*)-3-(4-methoxyphenyl)prop-2-en-1-ol[48] (16.4 mg, 0.100 mmol, 1.00 equiv) and Ti(O*i*Pr)$_4$ (3.0 µL, 10 µmol, 0.10 equiv) in HFIP (2.0 mL, 0.050 M) were subjected to the general procedure **C** except the reaction was performed using 10 mol% (0.10 equiv) of Ti(OiPr)$_4$. The crude product was purified by flash column chromatography (SiO_2; 60 Å, 15–40 µm; pentane:Et_2O; 23:2) to furnish compound **6d** as a colourless oil (9.5 mg, 20%).[49]

[47] Relative stereochemistry is assigned by analogy to compound **6f.**
[48] Y. Zhu, I. Colomer, A. L. Thompson, T. J. Donohoe, *J. Am. Chem. Soc.* **2019**, *141*, 6489–6493.
[49] Relative stereochemistry is assigned by analogy to compound **6f.**

^{1}H NMR (400 MHz, $CDCl_3$): δ 7.38 – 7.24 (m, 5H), 7.19 – 7.02 (m, 3H), 6.92 – 6.77 (m, 4H), 6.40 – 6.25 (m, 2H), 6.08 (ddd, *J* = 15.4, 8.2, 6.5 Hz, 1H), 5.80 (dd, *J* = 15.8, 9.6 Hz, 1H), 3.82 (s, 3H), 3.80 (s, 3H), 2.89 – 2.70 (m, 2H), 2.61 – 2.35 (m, 3H), 2.15 – 1.95 (m, 3H), 1.79 (q, *J* = 9.7 Hz, 1H), 1.51 – 1.41 (m, 1H), 1.38 – 1.22 (m, 4H).

^{13}C NMR (101 MHz, $CDCl_3$) δ 159.0, 158.8, 140.5, 137.3, 131.8, 131.5, 130.9, 130.8, 130.6, 129.1, 127.3 (2C), 127.2, 127.1 (2C), 125.8, 125.7 (2C), 114.1 (4C), 55.5 (2C), 53.4, 44.5, 43.2, 42.3, 38.4, 31.8, 30.8, 30.3, 28.0.

IR (film) ν_{max}: 2913, 2836, 2360, 1608, 1464, 1248, 1175, 1036, 967, 909, 734 cm^{-1}.

HRMS (ESI): calculated for $C_{33}H_{37}O_2$ $[M+H]^+$ requires m/z 465.2788, found m/z 465.2785 (*Δ* = -0.59 ppm).

(±)-(1*S*,2*R*,4a*S*,10a*S*)-2-(4-Methoxybenzyl)-1-((*E*)-4-methoxystyryl)-4*a*-methyl-1,2,3,4,4*a*,9,10,10*a*-octahydrophenanthrene 6e.

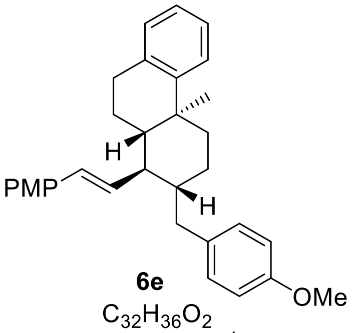

6e
$C_{32}H_{36}O_2$
452.64 g mol^{-1}

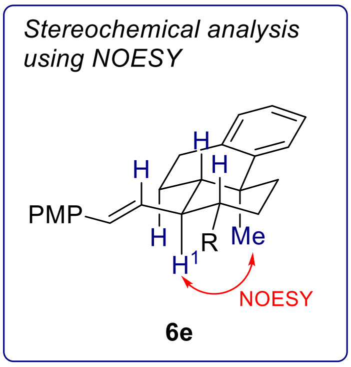

Triene **4** (33.2 mg, 0.100 mmol, 1.00 equiv), 4-(hydroxymethyl)phenol (12.4 mg, 0.100 mmol, 1.00 equiv) and Ti(O*i*Pr)$_4$ (9.0 µL, 30 µmol, 0.30 equiv) in HFIP (2.0 mL, 0.050 M) were subjected to the general procedure **C**. The crude product was purified by flash column chromatography (SiO_2; pentane:Et_2O; 19:1) to furnish compound **6e** as a colourless oil (13.0 mg, 29%).[50]

^{1}H NMR (400 MHz, $CDCl_3$): δ 7.36 (d, *J* = 8.7 Hz, 2H), 7.29 – 7.26 (m, 1H), 7.16 – 7.02 (m, 5H), 6.89 (d, *J* = 8.7 Hz, 2H), 6.81 (d, *J* = 8.6 Hz, 2H), 6.39 (d, *J* = 15.8 Hz, 1H), 5.80 (dd, *J* = 15.8, 9.4 Hz, 1H), 3.83 (s, 3H), 3.78 (s, 3H), 3.03 (dd, *J* = 13.6, 3.1 Hz, 1H), 2.91 – 2.74 (m, 2H), 2.31 – 2.22 (m, 1H), 2.15 (dd, *J* = 13.6, 9.6 Hz, 1H), 2.05 – 1.90 (m, 2H), 1.72 – 1.63 (m, 1H), 1.54 – 1.33 (m, 5H), 1.13 (s, 3H).

[50] Relative stereochemistry is assigned by analogy to compound **6f**.

^{13}C NMR (101 MHz, $CDCl_3$) δ 159.0, 157.8, 148.0, 135.7, 133.7, 132.5, 131.7, 130.6, 130.2 (2C), 129.3, 127.3 (2C), 125.7, 125.5, 124.8, 114.2 (2C), 113.6 (2C), 55.5, 55.4, 48.8, 46.4, 44.4, 40.8, 37.5, 37.1, 29.8, 27.1, 23.1, 23.0.

NOESY- 2D (600 MHz, $CDCl_3$): between H-1 and Me.

IR (film) ν_{max}: 2931, 2360, 1608, 1510, 1247, 1175, 1036, 969, 836, 761 cm^{-1}.

HRMS (ESI): calculated for $C_{32}H_{37}O_2$ $[M+H]^+$ requires m/z 453.2788, found m/z 453.2787 (*Δ* = -0.13 ppm).

(±)-3-(((1*S*,2*R*,4a*S*,10a*S*)-1-((*E*)-4-Methoxystyryl)-4*a*-methyl-1,2,3,4,4a,9,10,10*a*-octahydrophenanthren-2-yl)methyl)-1-methyl-1H-indole 6f.

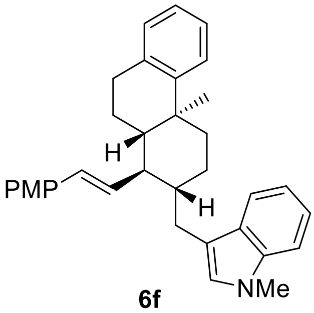

6f
$C_{34}H_{37}NO$
475.68 g mol^{-1}

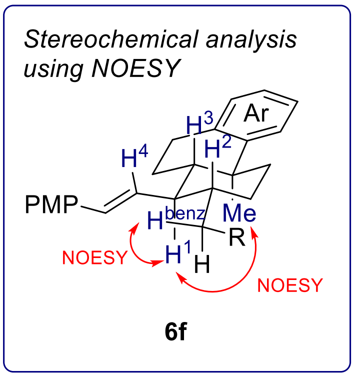

Triene **4** (33.2 mg, 0.100 mmol, 1.00 equiv), (1-methyl-1H-indol-3-yl)methanol[51] (16.1 mg, 0.10 mmol, 1.00 equiv) and $Ti(O\textit{i}Pr)_4$ (9.0 µL, 30 µmol, 0.30 equiv) in HFIP (2.0 mL, 0.050 M) were subjected to the general procedure **C**. The crude product was purified by flash column chromatography (SiO_2; 60 Å, 15–40 µm; pentane:Et_2O; 93:7) to furnish compound **6f** as a colourless oil (15.6 mg, 33%).

^{1}H NMR (400 MHz, $CDCl_3$): δ 7.55 (dd, *J* = 7.9, 1.1 Hz, 1H), 7.39 (d, *J* = 8.7 Hz, 2H), 7.30 – 7.25 (m, 2H), 7.21 (ddd, *J* = 8.1, 6.8, 1.1 Hz, 1H), 7.15 – 7.01 (m, 4H), 6.91 (d, *J* = 8.7 Hz, 2H), 6.81 (s, 1H), 6.44 (d, *J* = 15.8 Hz, 1H), 5.89 (dd, *J* = 15.8, 9.4 Hz, 1H), 3.84 (s, 3H), 3.73 (s, 3H), 3.19 (dd, *J* = 14.2, 3.2 Hz, 1H), 2.93 – 2.74 (m, 2H), 2.38 (dd, *J* = 14.4, 9.8 Hz, 1H), 2.30 – 2.19 (m, 1H), 2.07 (q, *J* = 10.0 Hz, 1H), 2.02 – 1.92 (m, 1H), 1.90 – 1.80 (m, 1H), 1.63 – 1.35 (m, 5H), 1.16 (s, 3H).

[51] M. A. J. Dubois, R. A. Croft, Y. Ding, C. Choi, D. R. Owen, J. A. Bulla, J. J. Mousseau, *RSC Med. Chem.* **2021**, *12*, 2045–2052.

^{13}**C NMR** (101 MHz, $CDCl_3$) δ 158.9, 148.1, 137.1, 135.8, 132.7, 131.5, 130.7, 129.3, 128.5, 127.3 (2C), 127.1, 125.7, 125.5, 124.8, 121.3, 119.5, 118.5, 114.2 (2C), 114.1, 109.2, 55.5, 49.0, 46.4, 43.5, 37.6, 37.1, 32.7, 30.7, 29.9, 27.9, 23.1 (2C).

NOESY- 2D (600 MHz, CD_2Cl_2): between H-1 and Me, between H-1 and H^{benz}.

Note: the appearance of ^{1}H NMR signal for H-1: 2.07 (q, *J* = 10.0 Hz, 1H, H-1) is indicative of a trans-diaxial arrangement between H-1 and H-2; H-1 and H-3, H-1 and H-4.

IR (film) ν_{max}: 3054, 2931, 1607, 1510, 1468, 1249, 1175, 1035, 970, 835, 761, 739 cm^{-1}.

HRMS (ESI): calculated for $C_{34}H_{38}ON$ $[M+H]^+$ requires m/z 476.2948, found m/z 476.2945 (Δ = -0.62 ppm).

*4.8 Cyclisation cascade methodology: synthesis of **12-16.***

(±)-(1*R*,2*S*,4a*S*,10a*S*)-2-Benzhydryl-6-methoxy-1-(4-methoxyphenyl)-4*a*-methyl-1,2,3,4,4*a*,9,10,10*a*-octahydrophenanthrene 12.

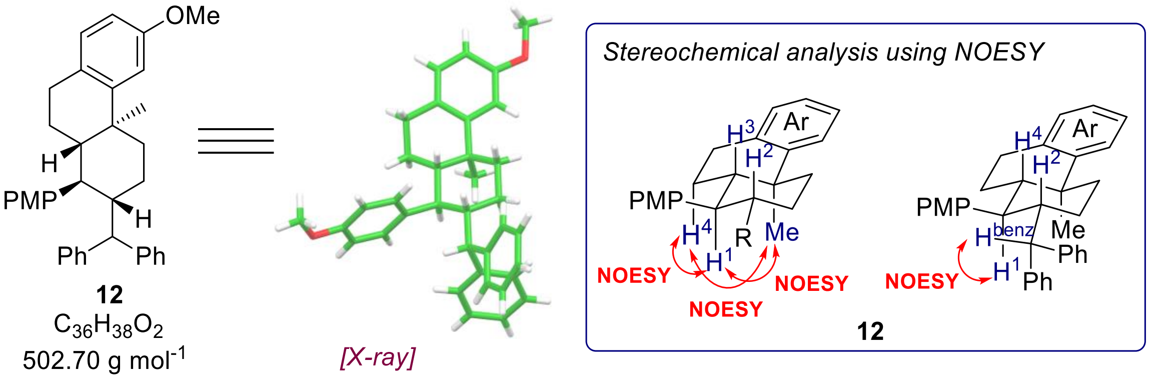

12
$C_{36}H_{38}O_2$
502.70 g mol^{-1}

Diene **7** (24.8 mg, 73.8 µmol, 1.00 equiv), benzhydrol (13.6 mg, 73.8 µmol, 1.00 equiv) and $Ti(O\textit{i}Pr)_4$ (6.70 µL, 22.6 µmol, 0.306 equiv) in HFIP (1.5 mL, 0.050 M) were subjected to the general procedure **B**. The crude product was purified by flash column chromatography (SiO_2; 60 Å, 15–40 µm; pentane:Et_2O; 24:1) to furnish compound **12** as a colourless foam (20.5 mg, 55%).

^{1}**H NMR** (400 MHz, $CDCl_3$): δ 7.35 – 7.07 (m, 11H), 6.95 – 6.80 (m, 5H), 6.67 (dd, *J* = 8.3, 2.7 Hz, 1H), 3.94 (d, *J* = 3.1 Hz, 1H), 3.84 (s, 3H), 3.78 (s, 3H), 2.70 – 2.48 (m, 3H), 2.43 – 2.29 (m, 2H), 1.97 – 1.86 (m, 1H), 1.77 – 1.60 (m, 3H), 1.38 – 1.24 (m, 1H), 1.16 (ddt, *J* = 13.2, 7.7, 2.5 Hz, 1H), 1.00 (s, 3H).

^{13}C NMR (101 MHz, $CDCl_3$): δ 158.1, 157.7, 149.1, 144.7, 141.7, 136.1, 133.3, 131.0 (2C), 130.0, 128.4 (2C), 128.1 (2C), 127.9 (2C), 127.8, 126.3, 126.1, 125.7, 115.1, 112.8, 110.9, 110.6, 55.4 (2C), 51.8, 49.1, 47.9, 47.4, 38.0, 37.8, 28.7, 23.9, 22.8 (2C).

NOESY- 2D (600 MHz, $CDCl_3$): between H-1 and Me, between H-1 and H-4, between H-4 and Me, between H-1 and H^{benz}.

IR (film) ν_{max}: 2939, 1610, 1510, 1250, 1178, 1038, 908, 731, 704 cm^{-1}.

M.p. 168-170 °C

HRMS (ESI): calculated for $C_{36}H_{38}O_2Na$ $[M+Na]^+$ requires m/z 525.2764, found m/z 525.2767 (Δ = 0.63 ppm).

(±)-(1*R*,2*S*,4*aS*,10*aS*)-2-Benzhydryl-5,8-dimethoxy-1-(4-methoxyphenyl)-4*a*-methyl-1,2,3,4,4*a*,9,10,10*a*-octahydrophenanthrene 13.

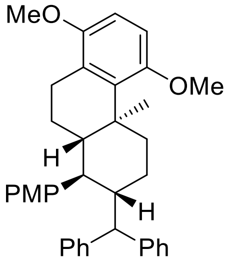

13
$C_{37}H_{40}O_3$
532.72 g mol^{-1}

Diene **9** (36.6 mg, 0.100 mmol, 1.00 equiv), benzhydrol (18.4 mg, 0.100 mmol, 1.00 equiv) and $Ti(O\textit{i}Pr)_4$ (9.0 µL, 30 µmol, 0.30 equiv) in HFIP (2.0 mL, 0.050 M) were subjected to the general procedure **B**. The crude product was purified by flash column chromatography (SiO_2; 60 Å, 15–40 µm; pentane:Et_2O; 23:2) to furnish compound **13** as a sticky oil (28.4 mg, 53%).[52]

^{1}H NMR (400 MHz, $CDCl_3$): δ 7.35 – 7.08 (m, 11H), 6.96 – 6.81 (m, 3H), 6.68 (d, *J* = 9.0 Hz, 1H), 6.62 (d, *J* = 8.9 Hz, 1H), 3.93 (d, *J* = 3.0 Hz, 1H), 3.84 (s, 3H), 3.77 -3.70 (m, 6H), 3.22 (dt, *J* = 13.0, 3.6 Hz, 1H), 2.70 – 2.44 (m, 3H), 2.35 – 2.18 (m, 1H), 1.85 – 1.64 (m, 3H), 1.44 – 1.31 (m, 1H), 1.23 – 1.04 (m, 5H).

^{13}C NMR (101 MHz, $CDCl_3$): δ 158.0, 153.0, 151.7, 144.9, 142.0, 137.2, 136.5, 133.3, 131.0 (2C), 128.5 (2C), 128.0 (2C), 127.8 (2C), 127.6, 126.2 (2C), 125.5, 115.1, 112.8, 109.4, 107.1, 55.7, 55.6, 55.3, 52.1, 51.7, 47.8, 47.2, 39.1, 35.7, 26.3, 24.0, 21.5, 17.9.

[52] Relative stereochemistry is determined by analogy to compounds **12** and **14**.

IR (film) ν_{max}: 2936, 1510, 1462, 1251, 1059, 1037, 909, 732, 703 cm^{-1}.

HRMS (ESI): calculated for $C_{37}H_{41}O_3$ $[M+H]^+$ requires m/z 533.3050, found m/z 533.3051 (Δ = 0.06 ppm).

(±)-(1*R*,2*S*,4*aS*,10*aS*)-2-Benzhydryl-1-(4-methoxyphenyl)-4*a*,6-dimethyl-1,2,3,4,4*a*,9,10,10*a*-octahydrophenanthrene 14.

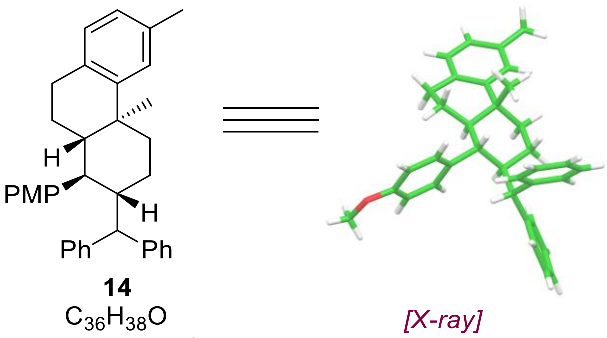

14
$C_{36}H_{38}O$
486.70 g mol^{-1}

Diene **8** (32.0 mg, 0.100 mmol, 1.00 equiv), benzhydrol (18.4 mg, 0.100 mmol, 1.00 equiv) and $Ti(O\textit{i}Pr)_4$ (9.0 µL, 30 µmol, 0.30 equiv) in HFIP (2.0 mL, 0.050 M) were subjected to the general procedure **B**. The crude product was purified by flash column chromatography (SiO_2; pentane:Et_2O; 49:1) to furnish compound **14** as a colourless foam (30.3 mg, 62%).[53]

^{1}H NMR (400 MHz, $CDCl_3$): δ 7.34 – 7.19 (m, 6H), 7.16 – 7.08 (m, 6H), 6.95 – 6.80 (m, 5H), 3.94 (d, *J* = 3.2 Hz, 1H), 3.83 (s, 3H), 2.72 – 2.51 (m, 3H), 2.41 – 2.27 (m, 5H), 1.98 – 1.88 (m, 1H), 1.77 – 1.60 (m, 3H), 1.38 – 1.26 (m, 1H), 1.15 (ddt, *J* = 13.1, 7.8, 2.6 Hz, 1H), 1.00 (s, 3H).

^{13}C NMR (101 MHz, $CDCl_3$): δ 158.0, 147.7, 144.7, 141.8, 136.1, 134.9, 133.3, 132.5, 131.0 (2C), 129.2, 128.4 (2C), 128.1 (2C), 127.9 (2C), 126.5, 126.3, 126.1, 125.6, 125.2, 115.1, 112.8, 55.3, 51.8, 49.2, 47.9, 47.4, 37.9, 37.5, 29.1, 23.9, 22.9, 22.8, 21.4.

M.p. 141-143 °C

IR (film) ν_{max}: 2938, 1610, 1510, 1247, 1177, 1035, 909, 830, 732, 703 cm^{-1}.

HRMS (ESI): calculated for $C_{36}H_{38}ONa$ $[M+Na]^+$ requires m/z 509.2815, found m/z 509.2816 (Δ = 0.19 ppm).

[53] Single crystal X-ray diffraction structure of enantiomer is shown.

(±)-(1*R*,2*S*,4*aS*,10*aS*)-2-Benzhydryl-6-chloro-1-(4-methoxyphenyl)-4*a*-methyl-1,2,3,4,4*a*,9,10,10*a*-octahydrophenanthrene 15 (from the mixture).

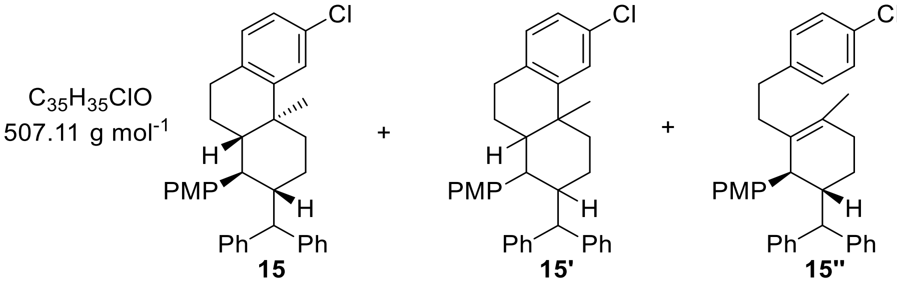

Diene **10** (34.0 mg, 0.100 mmol, 1.00 equiv), benzhydrol (18.4 mg, 0.100 mmol, 1.00 equiv) and $Ti(O\textit{i}Pr)_4$ (9.0 µL, 30 µmol, 0.30 equiv) in HFIP (2.0 mL, 0.050 M) were subjected to the general procedure **B**. The crude product was purified by flash column chromatography (SiO_2; 60 Å, 15–40 µm; pentane:Et_2O; 49:1) to furnish an inseparable mixture of compound **15,** diastereomer **15’** and alkene **15’’** as a colourless oil (**15**:**15’**:**15’’** = 66:16:18, 57% combined yield).[54,55]

Major diastereomer 15 (from the mixture)**:**

^{1}H NMR (600 MHz, CD_2Cl_2): δ 7.42 – 6.76 (m, 17H), 3.92 (d, *J* = 3.4 Hz, 1H), 3.81 (s, 3H), 2.70 – 2.63 (m, 1H), 2.60 – 2.51 (m, 2H), 2.39 (t, *J* = 11.2 Hz, 1H), 2.33 – 2.28 (m, 1H), 1.96 – 1.89 (m, 1H), 1.72 – 1.59 (m, 3H), 1.36 – 1.28 (m, 1H), 1.19 – 1.09 (m, 1H), 0.98 (s, 3H).

^{13}C NMR (101 MHz, CD_2Cl_2) δ 158.5, 150.2, 145.0, 142.2, 136.1, 134.6, 133.6, 131.3, 131.2 (2C), 130.9, 128.7 (2C), 128.4 (2C), 128.2 (2C), 126.6, 126.4, 125.9, 125.8, 125.1, 115.3, 113.1, 55.6, 52.3, 49.0, 48.2, 47.5, 38.0 (2C), 29.2, 24.4, 22.8 (2C).

Characteristic peaks for minor diastereomer 15’ (from the mixture):

^{1}H NMR (600 MHz, CD_2Cl_2): δ 3.76 (s, 3H), 3.68 (d, *J* = 4.6 Hz), 1.22 (s, 3H).

^{13}C NMR (101 MHz, CD_2Cl_2) δ 34.9

Characteristic peaks for alkene 15’’ (from the mixture):

^{1}H NMR (600 MHz, CD_2Cl_2): δ 3.78 (s, 3H), 3.19 – 3.16 (m, 1H), 1.83 (s, 3H).

^{13}C NMR (101 MHz, CD_2Cl_2) δ 138.3, 46.0, 19.1.

HRMS (ACI): calculated for $C_{35}H_{35}OClNa$ $[M+Na]^+$ requires m/z 529.2269, found m/z 529.2270 (*Δ* = 0.16 ppm).

---

[54] **15**:**15’**:**15’’** ratio was determined by ^{1}H NMR after column chromatography.
[55] Relative stereochemistry of the minor diastereomer could not be determined.

(±)-(1*R*,2*S*)-2-Benzhydryl-4'-methoxy-5-methyl-1,2,3,4-tetrahydro-1,1'-biphenyl (major)-16 and (±)-(1*S*,2*S*)-2-Benzhydryl-4'-methoxy-5-methyl-1,2,3,6-tetrahydro-1,1'-biphenyl (minor)-16.

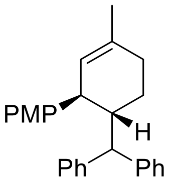

16
Major

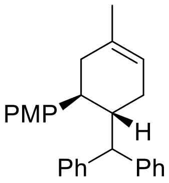

16'
Minor

$C_{27}H_{28}O$
368.52 g mol^{-1}
16:**16'** = 62:38 rr

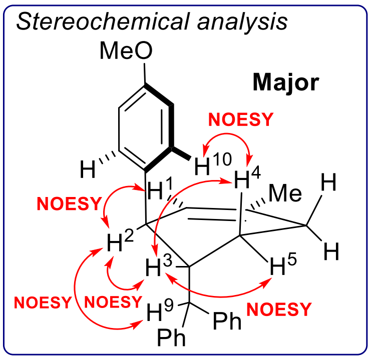

Diene **11** (32.2 mg, 0.159 mmol, 1.00 equiv), benzhydrol (29.3 mg, 0.159 mmol, 1.00 equiv) and Ti(O*i*Pr)$_4$ (14.5 µL, 49.0 µmol, 0.308 equiv) in HFIP (8.1 mL, 0.020 M) were subjected to the general procedure **B** except the reaction was performed at 70 °C overnight and at 0.02 M. The crude product was purified by flash column chromatography (SiO_2**;** pentane:Et_2O; 99:1 to 49:1) to furnish compounds **16** and **16'** as an inseparable mixture of alkene regioisomers as a colourless oil (32.1 mg, 55%, 63:37 rr).[56]

^{1}H NMR (400 MHz, $CDCl_3$): δ 748 – 7.39 (m, 2H$_{maj}$, 2H$_{min}$), 7.37 – 7.11 (m, 8H$_{maj}$, 8H$_{min}$), 7.08 (d, *J* = 8.7 Hz, 2H$_{min}$), 7.04 (d, *J* = 8.6 Hz, 2H$_{maj}$), 6.86 – 6.80 (m, 2H$_{maj}$, 2H$_{min}$), 5.39 – 5.33 (m, 1H$_{min}$), 5.31 (d, *J* = 3.7 Hz, 1H$_{maj}$), 3.98 – 3.90 (m, 1H$_{maj}$, 1H$_{min}$), 3.81 – 3.78 (m, 3H$_{maj}$, 3H$_{min}$), 3.21 – 3.10 (m, 1H$_{maj}$), 2.84 (dt, *J* = 6.3, 2.8 Hz, 1H$_{min}$), 2.68 – 2.54 (m, 1H$_{maj}$, 1H$_{min}$), 2.37 (dd, *J* = 17.5, 6.4 Hz, 1H$_{min}$), 2.09 – 1.95 (m, 1H$_{maj}$, 1H$_{min}$), 1.90 – 1.78 (m, 4H$_{maj}$, 4H$_{min}$), 1.74 – 1.63 (m, 1H$_{maj}$, 1H$_{min}$), 1.38 – 1.26 (m, 1H$_{maj}$).

^{13}C NMR (101 MHz, $CDCl_3$) **for the major diastereomer** (from the mixture): δ 158.0, 145.1, 144.1, 138.5, 135.0, 129.4 (2C), 128.7 (2C), 128.5 (2C), 128.4 (2C), 128.3 (2C), 126.3, 126.2, 121.7, 113.6 (2C), 55.4, 53.4, 43.6, 42.3, 26.3, 24.0, 19.8. **Selected peaks for the minor diastereomer** (from the mixture): δ 157.8, 144.6, 144.5, 139.2, 132.6, 128.5, 128.4, 128.3, 128.2, 126.2, 120.0, 113.7, 55.4, 53.6, 42.1, 38.5, 31.5, 23.9, 23.7.

NOESY (600 MHz, $CDCl_3$): between H-1$_{maj}$ and H-2$_{maj}$, between H-2$_{maj}$ and H-3$_{maj}$, between H-2$_{maj}$ and H-9$_{maj}$, between H-3$_{maj}$ and H-4$_{maj}$, between H-3$_{maj}$ and H-5$_{maj}$, between H-10$_{maj}$ and H-4$_{maj}$.

IR (film) ν_{max}: 2910, 1509, 1493, 1450, 1246, 1178, 1034, 829, 757, 747, 703 cm^{-1}.

[56] Ratio of alkene regioisomers was determined after column chromatography.

HRMS (ESI): calculated for $C_{27}H_{29}O$ $[M+H]^+$ requires m/z 369.2213, found m/z 369.2215 (*Δ* = 0.51 ppm).

4.9 Derivatisation of ***6d****: synthesis of an 18-norsteroid analogue* ***18****.*

(±)-(8*S*,9*S*,14*S*)-7,8,9,11,12,14,15,16-Octahydro-6H-cyclopenta[*a*]phenanthrene 17.

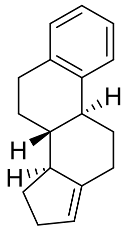

17
$C_{17}H_{20}$
224.35 g mol^{-1}

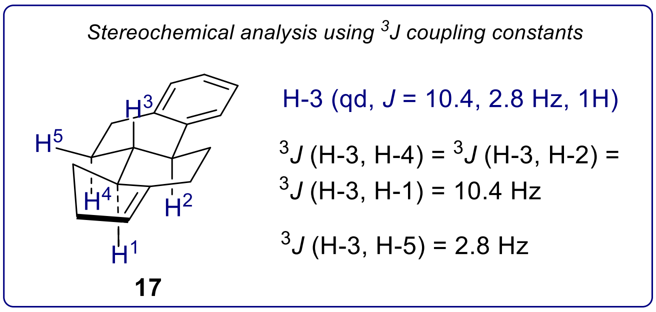

Alkene **6d** (9.2 mg, 20 µmol, 1.0 equiv) and 2nd generation Hoveyda-Grubbs catalyst (3.8 mg, 6.0 µmol, 0.30 equiv) were transferred to a microwave vial and purged with nitrogen for 15 min. Balloon of nitrogen was removed and degassed PhMe (0.42 mL) was added. The solution was stirred at 110 °C overnight, cooled to rt and volatiles were removed *in vacuo*. The crude product was purified by flash column chromatography (SiO_2; pentane) to afford product **17** as a colurless oil (2.8 mg, 63%).

^{1}H NMR (400 MHz, $CDCl_3$): δ 7.33 (d, *J* = 7.6 Hz, 1H), 7.21 – 7.01 (m, 3H), 5.34 – 5.29 (m, 1H), 2.92 – 2.77 (m, 2H), 2.66 (ddd, *J* = 14.1, 4.5, 2.2 Hz, 1H), 2.57 – 2.44 (m, 2H), 2.37 – 2.12 (m, 5H), 2.05 – 1.93 (m, 1H), 1.54 – 1.37 (m, 2H), 1.32 – 1.19 (m, 1H), 1.08 (qd, *J* = 10.4, 2.8 Hz, 1H).

^{13}C NMR (101 MHz, $CDCl_3$) δ 145.3, 140.0, 137.3, 129.2, 126.4, 125.8, 125.7, 121.1, 51.4, 49.0, 42.9, 31.8, 31.5, 30.3, 29.2, 28.8, 27.8.

IR (film) ν_{max}: 2925, 2854, 2360, 1489, 1450, 1433, 739 cm^{-1}.

HRMS (GC EI): calculated for $C_{17}H_{20}$ $[M]^+$ requires 224.15595, found m/z 224.15548 (*Δ* = -2.11 ppm).

(±)-(8*S*,9*S*,13*S*,14*S*,17*S*)-7,8,9,11,12,13,14,15,16,17-Decahydro-6H-cyclopenta[a]phenanthren-17-ol 18 (major) and (±)-(8*S*,9*S*,13*R*,14*S*,17*R*)-7,8,9,11,12,13,14,15,16,17-Decahydro-6H-cyclopenta[a]phenanthren-17-ol 18 (minor).

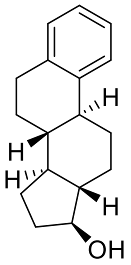

Major
18

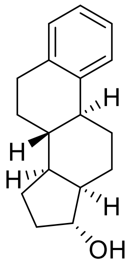

Minor
18

$C_{17}H_{22}O$
242.36 g mol^{-1}

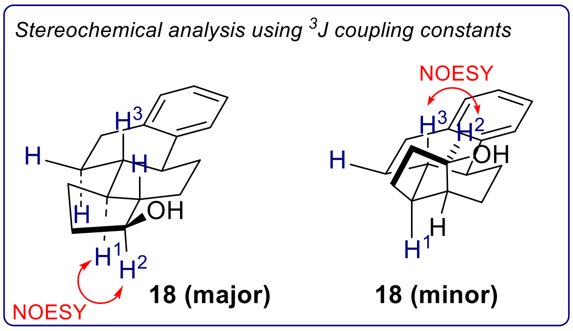

According to a modified literature procedure[57], alkene **17** (6.1 mg, 27 µmol, 1.0 equiv) and $LiBH_4$ (cat) were transferred to a microwave vial which was then purged with nitrogen. Catecholborane (7.3 µL, 68 µmol, 2.5 equiv) as a stock solution in C_6H_6 (0.40 mL, 0.17 M) was then added in one portion and the obtained suspension was stirred at reflux for 24 h. Mixture was then cooled to rt and aq NaOH (3M, 0.2 mL) followed by aq H_2O_2 (30%, 0.2 mL) were added in one portion. The obtained mixture was vigorously stirred at rt for 4 h, diluted with water (3 mL) and CH_2Cl_2 (3 mL). The organic layer was removed, and the aqueous layer was extracted with CH_2Cl_2 (3 × 3 mL). Organic layers were combined, washed with brine and dried over Na_2SO_4. The crude product was purified by flash column chromatography (SiO_2; pentane:Et_2O; 3:2) to furnish an inseparable mixture of alcohols **18 (major)** and **18 (minor)** as a white solid (4.8 mg, 61:39 dr, 73%).

^{1}H NMR (600 MHz, $CDCl_3$): δ 7.35 – 7.28 (m, $1H_{maj}$, $1H_{min}$), 7.18 – 7.05 (m, $3H_{maj}$, $3H_{min}$), 4.13 (td, *J* = 8.7, 6.7 Hz, $1H_{min}$), 3.86 (td, *J* = 8.6, 6.1 Hz, $1H_{maj}$), 2.93 – 2.79 (m, $2H_{maj}$, $2H_{min}$), 2.61 – 2.54 (m, $1H_{maj}$), 2.36 – 2.28 (m, $1H_{maj}$, $2H_{min}$), 2.25 – 2.20 (m, $1H_{maj}$), 2.20 – 2.08 (m, $1H_{maj}$, $1H_{min}$), 2.05 (dq, *J* = 14.0, 3.1 Hz, $1H_{min}$), 2.01 – 1.89 (m, $1H_{maj}$, $3H_{min}$), 1.89 – 1.76 (m, $1H_{maj}$, $2H_{min}$), 1.64 – 1.49 (m, $1H_{maj}$, $2H_{min}$), 1.46 – 1.20 (m, $6H_{maj}$, $2H_{min}$), 1.15 (qd, *J* = 10.8, 6.5 Hz, $1H_{maj}$), 1.02 (qd, *J* = 11.1, 2.7 Hz, $1H_{min}$).

^{13}C NMR (151 MHz, $CDCl_3$) **for the major diastereomer** (from the mixture): δ 140.3, 137.0, 129.2, 125.8, 125.8, 125.7, 78.0, 53.6, 48.1, 45.8, 43.6, 33.1, 30.6, 29.8, 29.0, 27.5, 26.7.

---

[57] (a) A. Arase, Y. Nunokawa, Y. Masuda, M. Hoshi, *J. Chem. Soc., Chem. Commun*. **1991**, *4*, 205-206; (b) C. E. Garrett, G. C. Fu, *J. Org. Chem*. **1996**, *61*, 3224-3225; (c) Y. Suseela, M. Periasamy, *J. Organomet. Chem*. **1993**, *450*, 47-52; (d) A.-P. Schaffner, B. Becattini, C. Ollivier, V. Weber, P. Renaud, *Synthesis* **2003**, 2740–2742; (e) A. Kuhl, H. Karele, W. Kreiser, *Helv. Chim. Acta* **1999**, *82*, 30–34.

Peaks for the minor diastereomer (from the mixture): δ 140.4, 137.2, 129.1, 126.1, 125.8, 125.7, 74.3, 47.6, 44.0, 42.7, 41.7, 33.0, 30.3, 28.1, 26.8, 26.5, 24.9.

NOESY- 2D (600 MHz, $CDCl_3$): between H-1_{maj} and H-2_{maj}; between H-2_{maj} and H-3_{maj}.

M.p: 120-130 °C.

IR (film) ν_{max}: 3315, 2930, 2863, 2360, 2341, 1489, 1451, 1073, 912, 742 cm^{-1}.

HRMS (GC EI): calculated for $C_{17}H_{22}O$ $[M]^+$ requires m/z 242.16652, found m/z 242.16707 (*Δ* = 3.21 ppm).

*4.10 Formal synthesis of a 18-nor-estradiol precursor **19**.*

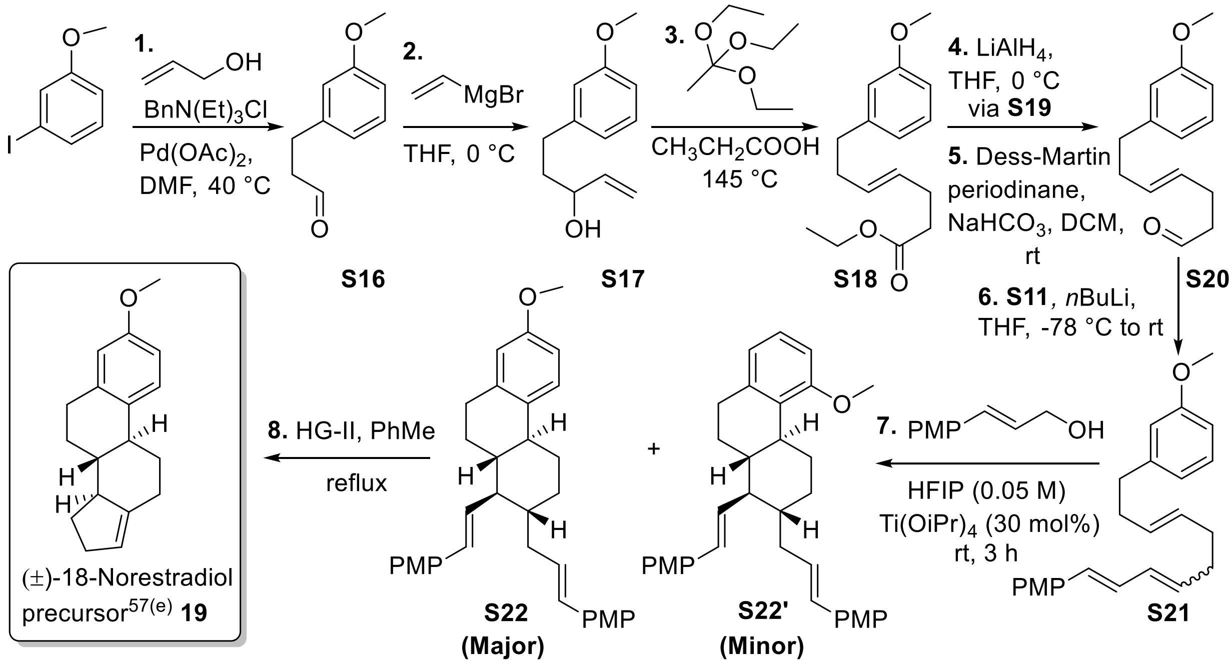

[57] (e) A. Kuhl, H. Karele, W. Kreiser, *Helv. Chim. Acta* **1999**, *82*, 30–34.

3-(3-Methoxyphenyl)propanal S16.

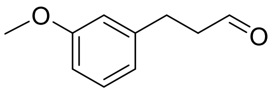

S16
$C_{10}H_{12}O_2$
164.20 g mol^{-1}

To a solution of 3-iodoanisole (2.00 mL, 16.8 mmol, 1.00 equiv) in DMF (30 mL) were added triethylbenzylammonium chloride (3.90 g, 17.1 mmol, 1.02 equiv), $NaHCO_3$ (2.88 g, 34.3 mmol, 2.04 equiv), allylalcohol (2.3 mL, 34 mmol, 2.0 equiv), and $Pd(OAc)_2$ (920 mg , 4.1

mmol, 0.24 equiv). The reaction mixture was stirred at 40°C for 12 h. After cooling to the rt, the mixture was poured into ice-water (30 mL) and extracted with CH_2Cl_2 (3 × 30 mL). The combined organic layers were washed with brine, dried over Na_2SO_4 and volatiles were removed *in vacuo* to furnish the crude product, which was purified by flash column chromatography (SiO_2; pentane:EtOAc; 9:1) to afford the aldehyde **S16** as a pale yellow oil (1.19 g, 43%).

^{1}H NMR (400 MHz, $CDCl_3$): δ 9.82 (t, *J* = 1.6 Hz, 1H), 7.21 (td, *J* = 7.6, 1.0 Hz, 1H), 6.83 – 6.71 (m, 3H), 3.80 (s, 3H), 2.94 (t, *J* = 7.5 Hz, 2H), 2.84 – 2.73 (m, 2H).

^{13}C NMR (101 MHz, $CDCl_3$): δ 201.7, 159.9, 142.1, 129.7, 120.7, 114.3, 111.7, 55.3, 45.3, 28.3.

The analytical data are consistent with those previously reported in the literature.[58]

5-(3-Methoxyphenyl)pent-1-en-3-ol S17.

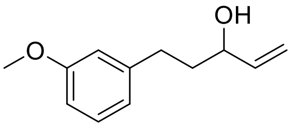

S17
$C_{12}H_{16}O_2$
192.26 g mol^{-1}

Aldehyde **S16** (1.19 g, 7.25 mmol, 1.00 equiv) was dissolved in THF (45 mL) and cooled to 0 °C. To the obtained solution vinyl magnesium bromide (9.5 mL, 1.0 M in THF, 9.5 mmol, 1.3 equiv) was added dropwise. The reaction mixture was stirred at the same temperature for 2 h and then quenched with aq. sat. NH_4Cl (5 mL) and diluted with water (30 mL). The organic layer was separated, and the aqueous layer was extracted with Et_2O (3 × 30 mL). The organic layers were combined, washed with brine, dried over anhydrous Na_2SO_4 and concentrated *in vacuo*. The crude product was purified by flash column chromatography (SiO_2; pentane:Et_2O; 7:3) to furnish allylic alcohol **S17** as a pale yellow oil (0.950 g, 68%).

^{1}H NMR (400 MHz, $CDCl_3$): δ 7.20 (t, *J* = 7.7 Hz, 1H), 6.85 – 6.69 (m, 3H), 5.91 (ddd, *J* = 16.9, 10.4, 6.1 Hz, 1H), 5.25 (dt, *J* = 17.1, 1.7 Hz, 1H), 5.14 (d, *J* = 10.3 Hz, 1H), 4.14 (q, *J* = 6.3 Hz, 1H), 3.80 (s, 3H), 2.79 – 2.61 (m, 2H), 1.92 – 1.77 (m, 2H).

^{13}C NMR (101 MHz, $CDCl_3$): δ 159.8, 143.7, 141.1, 129.5, 121.0, 115.1, 114.3, 111.3, 72.6, 55.3, 38.5, 31.8.

[58] W. Ren, W. Chang, J. Dai, Y. Shi, J. Li, Y. Shi, *J. Am. Chem. Soc.* **2016**, *138*, 14864–14867.

IR (film) ν_{max}: 3395, 2944, 1602, 1585, 1489, 1261, 1153, 1042, 993, 924, 782, 696 cm^{-1}.

HRMS (ESI): A stable molecular ion could not be detected by ESI, APCI or EI.

Ethyl (*E*)-7-(3-methoxyphenyl)hept-4-enoate S18.

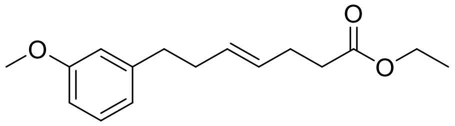

S18
$C_{16}H_{22}O_3$
262.35 g mol^{-1}

A solution of the allylic alcohol **S17** (0.937 g, 4.87 mmol, 1.00 equiv) and propionic acid (25 µL) in triethyl orthoacetate (12 mL) was heated with stirring at 145 °C for 24 h. The solution was cooled to room temperature, poured into water (10 mL) and extracted with Et_2O (3 × 15 mL). The organic layers were combined, washed successively with aq. HCl (1 M, 2 × 30 mL) and brine (30 mL) and dried over anhydrous Na_2SO_4. Volatiles were removed *in vacuo* and the crude product was purified by flash column chromatography (SiO_2; pentane:Et_2O; 23:2 to 9:1) to furnish ester **S18** as a colourless oil (1.13 g, 88%).

^{1}H NMR (400 MHz, $CDCl_3$): δ 7.23 – 7.13 (m, 1H), 6.80 – 6.69 (m, 3H), 5.57 – 5.38 (m, 2H), 4.12 (q, *J* = 7.1 Hz, 2H), 3.80 (s, 3H), 2.63 (dd, *J* = 9.0, 6.6 Hz, 2H), 2.38 – 2.24 (m, 6H), 1.25 (t, *J* = 7.2 Hz, 3H).

^{13}C NMR (101 MHz, $CDCl_3$): δ 173.4, 159.7, 143.8, 130.8, 129.3, 128.9, 121.0, 114.3, 111.2, 60.4, 55.3, 36.1, 34.4 (2C), 28.0, 14.4.

IR (film) ν_{max}: 2938, 1735, 1602, 1490, 1455, 1261, 1166, 1153, 1044, 971, 780, 696 cm^{-1}.

HRMS (ESI): calculated for $C_{16}H_{22}O_3Na$ $[M+Na]^+$ requires m/z 285.1461, found m/z 285.1462 (Δ = 0.28 ppm).

(*E*)-7-(3-Methoxyphenyl)hept-4-en-1-ol S19.

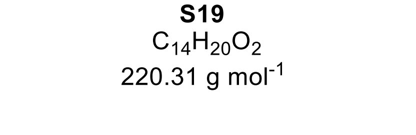

S19
$C_{14}H_{20}O_2$
220.31 g mol^{-1}

$LiAlH_4$ (0.163 g, 4.30 mmol, 1.00 equiv) was added portion-wise to the solution of ester **S18** (1.13 g, 4.31 mmol, 1.00 equiv) in THF (32 mL) at 0 °C. The reaction mixture was stirred at the same temperature for 3 h followed by the subsequent addition of water (0.16 mL), 15% aq. NaOH (0.16 mL) and water (0.48 mL). The obtained suspension was stirred at rt for 30 min, transferred to a separatory funnel, diluted with an additional amount of water (30 mL) and extracted with Et_2O (3 × 30 mL). The organic layers were combined, washed with brine and dried over anhydrous Na_2SO_4. Volatiles were removed *in vacuo* and the crude product was purified by flash column chromatography (SiO_2; pentane:Et_2O; 3:2 to 2:3) to furnish alcohol **S19** as a colourless oil (0.869 g, 92%).

^{1}H NMR (400 MHz, $CDCl_3$): δ 7.23 – 7.15 (m, 1H), 6.80 – 6.67 (m, 3H), 5.54 – 5.37 (m, 2H), 3.80 (s, 3H), 3.61 (t, *J* = 6.5 Hz, 2H), 2.65 (dd, *J* = 8.7, 6.7 Hz, 2H), 2.35 – 2.25 (m, 2H), 2.11 – 2.01 (m, 2H), 1.66 – 1.57 (m, 2H).

^{13}C NMR (101 MHz, $CDCl_3$): δ 159.7, 143.8, 130.4, 130.3, 129.3, 121.1, 114.4, 111.1, 62.6, 55.3, 36.2, 34.4, 32.5, 29.0.

IR (film) v_{max}: 3394, 2934, 2361, 2341, 1603, 1490, 1455, 1262, 1153, 1053, 870, 781 cm^{-1}.

HRMS (ESI): calculated for $C_{14}H_{20}O_2Na$ $[M+Na]^+$ requires m/z 243.1356, found m/z 243.1348 (*Δ* = -3.11 ppm).

(*E*)-7-(3-Methoxyphenyl)hept-4-enal S20.

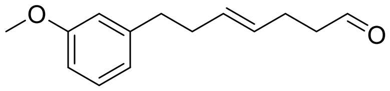

S20
$C_{14}H_{18}O_2$
218.30 g mol^{-1}

(E)-7-(3-Methoxyphenyl)hept-4-en-1-ol **S19** (0.331 g, 1.50 mmol, 1.00 equiv) was dissolved in CH_2Cl_2 (25 mL) and to the obtained solution $NaHCO_3$ (2.4 g, 29 mmol, 7.4 equiv) and Dess-Martin Periodinane (0.96 g, 2.3 mmol, 1.5 equiv) were added sequentially in one portion. The obtained suspension was stirred at rt until the complete consumption of the starting material, then quenched with sat. aq. $Na_2S_2O_3$ (7 mL) and stirred for additional 15 min. The organic layer was removed, and the aqueous layer was extracted with CH_2Cl_2 (3 × 10 mL). The organic layers were combined, washed with brine (30 mL) and dried over Na_2SO_4. Volatiles were removed *in vacuo* and the crude product was purified by flash column chromatography (SiO_2; pentane:Et_2O; 7:3) to furnish aldehyde **S20** as a colourless oil (0.263 g, 80%).

^{1}H NMR (400 MHz, $CDCl_3$): δ 9.74 (t, *J* = 1.7 Hz, 1H), 7.23 – 7.14 (m, 1H), 6.82 – 6.68 (m, 3H), 5.57 – 5.37 (m, 2H), 3.80 (s, 3H), 2.63 (dd, *J* = 8.9, 6.7 Hz, 2H), 2.52–2.42 (m, 2H), 2.38 – 2.24 (m, 4H).

^{13}C NMR (101 MHz, $CDCl_3$): δ 202.5, 159.7, 143.7, 131.1, 129.4, 128.6, 121.0, 114.4, 111.2, 55.3, 43.6, 36.0, 34.3, 25.3.

IR (film) ν_{max}: 2918, 2360, 1726, 1602, 1489, 1261, 1152, 1051, 970, 911, 733,, 696 cm^{-1}.

HRMS (ESI): calculated for $C_{14}H_{18}O_2Na$ $[M+Na]^+$ requires m/z 241.1199, found m/z 241.1193 (*Δ* = -2.51 ppm).

1-Methoxy-3-((3*E*,7*E*,9*E*)-10-(4-methoxyphenyl)deca-3,7,9-trien-1-yl)benzene (*E*)-S21 and 1-methoxy-3-((3*E*,7*Z*,9*E*)-10-(4-methoxyphenyl)deca-3,7,9-trien-1-yl)benzene (*Z*)-S21.

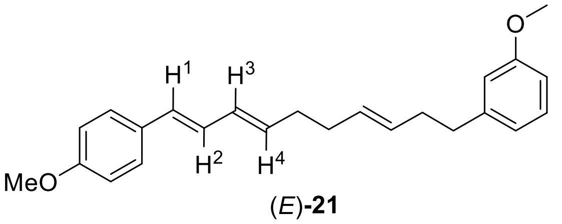

(*E*)-**21**
$C_{24}H_{28}O_2$
348.49 g mol^{-1}
$^3J_{H-1, H-2}$ = 15.6 Hz
$^3J_{H-3, H-4}$ = 14.4 Hz

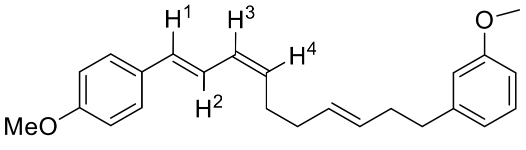

(*Z*)-**21**
$C_{24}H_{28}O_2$
348.49 g mol^{-1}
$^3J_{H-1, H-2}$ = 15.6 Hz

(*E*)-(3-(4-Methoxyphenyl)allyl)triphenylphosphonium bromide **S11** (0.884 g, 1.81 mmol, 1.50 equiv) was suspended in THF (9 mL) and cooled to -78 °C. *n*BuLi (0.72 mL, 2.5 M in hexanes, 1.8 mmol, 1.5 equiv) was added dropwise and the obtained suspension was allowed to stir at the same temperature for 30 min. Aldehyde **S20** (0.263 g, 1.20 mmol, 1.00 equiv) in THF (3 mL) was added dropwise at -78 °C and the reaction mixture was allowed to warm to rt overnight. The reaction was quenched with sat. aq. NH_4Cl (3 mL), diluted with water (10 mL) and extracted with Et_2O (3 × 15 mL). The organic layers were combined, washed with brine and volatiles were removed *in vacuo.* The crude product was purified by flash column chromatography (SiO_2, pentane:Et_2O; 24:1) to furnish an inseparable mixture of alkenes (*E*)-**21** and (*Z*)-**21** as a colourless waxy solid (0.244 g, 58%, 57:43 *E*:*Z*).

^{1}H NMR (400 MHz, $CDCl_3$): δ 7.39 – 7.29 (m, $2H_{maj}$, $2H_{min}$), 7.23 – 7.16 (m, $1H_{maj}$, $1H_{min}$), 6.97 – 6.70 (m, $5H_{maj}$, $6H_{min}$), 6.63 (dd, *J* = 15.6, 10.4 Hz, $1H_{maj}$), 6.52 – 6.36 (m, $1H_{maj}$, $1H_{min}$), 6.23 – 6.07 (m, $1H_{maj}$, $1H_{min}$), 5.75 (dt, *J* = 14.4, 6.7 Hz, $1H_{maj}$), 5.57 – 5.38 (m, $2H_{maj}$, $3H_{min}$),

3.85 – 3.76 (m, $6H_{maj}$, $6H_{min}$), 2.69 – 2.61 (m, $2H_{maj}$, $2H_{min}$), 2.39 – 2.26 (m, $2H_{maj}$, $4H_{min}$), 2.23 – 2.06 (m, $4H_{maj}$, $2H_{min}$).

^{13}C NMR (101 MHz, $CDCl_3$) **for the major diastereomer** (from the mixture): δ 159.7, 159.0, 143.9, 134.0, 131.1, 130.6, 130.3, 130.1, 129.8, 129.3, 127.5, 127.4 (2C), 121.1, 114.4, 114.2 (2C), 111.1, 55.4, 55.3, 36.3, 34.4, 33.0, 32.6. **Selected peaks for the minor diastereomer** (from the mixture): δ 159.2, 131.8, 131.4, 130.6, 127.7, 122.7, 114.3, 114.2, 34.5, 32.8, 28.1.

IR (film) v_{max}: 2935, 2360, 1604, 1510, 1252, 1174, 1152, 1037, 987, 736, 696 cm^{-1}.

HRMS (ESI): calculated for $C_{24}H_{29}O_2$ $[M+H]^+$ requires m/z 349.2162, found m/z 349.2166 (Δ = 1.12 ppm).

(±)-(1*S*,2*R*,4a*S*,10a*S*)-7-Methoxy-2-((*E*)-3-(4-methoxyphenyl)allyl)-1-((*E*)-4-methoxystyryl)-1,2,3,4,4*a*,9,10,10*a*-octahydrophenanthrene S22 (major) and (±)-(1*S*,2*R*,4a*S*,10a*S*)-5-methoxy-2-((*E*)-3-(4-methoxyphenyl)allyl)-1-((*E*)-4-methoxystyryl)-1,2,3,4,4*a*,9,10,10*a*-octahydrophenanthrene S22' (minor).

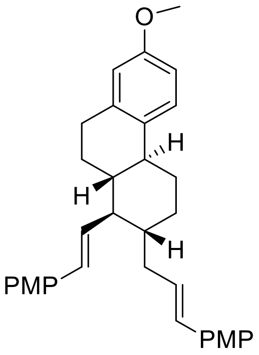

S22 (Major)
$C_{34}H_{38}O_3$
494.68 g mol^{-1}

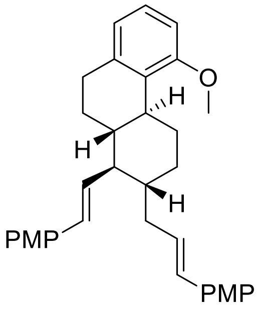

S22' (Minor)
$C_{34}H_{38}O_3$
494.68 g mol^{-1}

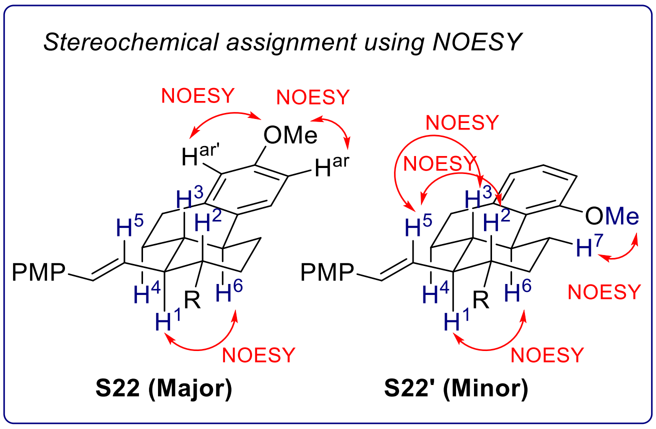

Triene **(*E*/*Z*)-S21** (40.0 mg, 0.115 mmol, 1.00 equiv, 57:43 *E*:*Z*) and trans-4-methoxycinnamyl alcohol (18.9 mg, 0.115 mmol, 1.00 equiv) were transferred to a microwave vial which was then purged with nitrogen. The balloon of nitrogen was removed, and HFIP (2.1 mL), followed by $Ti(O\textit{i}Pr)_4$ (3.4 µL, 11.5 µmol, 0.100 equiv) as a stock solution in HFIP (0.17 mL, 0.067 M), were added to obtain a 0.05 M solution of a triene **(*E*/*Z*)-S21**. The solution was stirred at rt for 3 h, then diluted with water (3 mL). The obtained suspension was transferred to a separatory funnel and extracted with CH_2Cl_2 (3 × 4 mL). The organic layers were combined, washed with brine and volatiles were removed *in vacuo*. The crude product was purified by flash column chromatography (SiO_2; 60 Å, 15–40 µm; pentane:Et_2O; 22:3) to furnish alkene **S22'** (minor, 3.0 mg, 5%) as a colourless oil and alkene **S22** (major, 10.1 mg, 18%) as a colourless oil.

Major regioisomer S22:

^{1}H NMR (500 MHz, $CDCl_3$): δ 7.38 – 7.33 (m, 2H), 7.28 – 7.26 (m, 2H), 7.25 – 7.21 (m, 1H), 6.92 – 6.79 (m, 4H), 6.72 (dd, *J* = 8.7, 2.8 Hz, 1H), 6.64 – 6.59 (m, 1H), 6.36 (d, *J* = 15.9 Hz, 1H), 6.30 (d, *J* = 15.8 Hz, 1H), 6.09 (ddd, *J* = 15.4, 8.2, 6.5 Hz, 1H), 5.80 (dd, *J* = 15.9, 9.6 Hz, 1H), 3.85 – 3.79 (m, 6H), 3.77 (s, 3H), 2.85 – 2.70 (m, 2H), 2.53 – 2.43 (m, 2H), 2.39 – 2.29 (m, 1H), 2.14 – 2.06 (m, 2H), 2.05 – 1.97 (m, 1H), 1.79 (q, *J* = 10.0 Hz, 1H), 1.50 – 1.39 (m, 1H), 1.35 – 1.20 (m,4H).

^{13}C NMR (125 MHz, $CDCl_3$): δ 159.0, 158.8, 157.6, 138.5, 132.8, 131.8, 131.5, 131.0, 130.7, 130.6, 127.3 (2C), 127.2, 127.1 (2C), 126.8, 114.1 (4C), 113.6, 111.8, 55.5, 55.4, 55.3, 53.3, 44.8, 42.6, 42.3, 38.5, 31.8, 31.0, 30.6, 28.1.

NOESY- 2D (500 MHz, $CDCl_3$): between H-1 and H-6; between OMe and H^{ar}; between OMe and $H^{ar'}$.

Note: the appearance of ^{1}H NMR signal for H-1: 1.79 (q, *J* = 10.0 Hz, 1H, H-1) is indicative of a trans-diaxial arrangement between H-1 and H-2, H-1 and H-3, H-1 and H-5.

IR (film) v_{max}: 2919, 1608, 1510, 1249, 1175, 1037, 968, 838 cm^{-1}.

HRMS (ESI): calculated for $C_{34}H_{39}O_3$ $[M+H]^+$ requires m/z 495.2894, found m/z 495.2893 (*Δ* = - 0.12 ppm).

Minor regioisomer S22':

^{1}H NMR (400 MHz, $CDCl_3$) δ 7.38 – 7.32 (m, 2H), 7.29 – 7.22 (m, 2H), 7.10 – 7.02 (m, 1H), 6.90 – 6.80 (m, 4H), 6.73 – 6.64 (m, 2H), 6.40 – 6.22 (m, 2H), 6.09 (ddd, *J* = 15.4, 8.2, 6.5 Hz, 1H), 5.82 (dd, *J* = 15.9, 9.6 Hz, 1H), 3.84 – 3.78 (m, 9H), 2.98 (dd, *J* = 12.6, 2.8 Hz, 1H), 2.83 – 2.63 (m, 2H), 2.56 – 2.43 (m, 2H), 2.09 – 1.85 (m, 4H), 1.53 – 1.46 (m, 1H), 1.42 – 1.30 (m, 2H), 1.17 – 1.00 (m, 2H).

^{13}C NMR (125 MHz, $CDCl_3$): δ 159.1, 158.9, 158.7, 140.2, 132.2, 131.2, 131.0, 130.7, 130.6, 129.1, 127.4, 127.2 (2C), 127.1 (2C), 126.2, 121.8, 114.1 (4C), 108.2, 55.5 (2C), 55.1, 53.5, 47.7, 43.3, 42.9, 38.5, 32.8, 32.1, 31.3, 26.9.

NOESY- 2D (500 MHz, $CDCl_3$): between H-1 and H-6; between H-5 and H-3; between H-5 and H-2; between H-7 and OMe.

IR (film) v_{max}: 2981, 2918, 2360, 1510, 1465, 1248, 1174, 967 cm^{-1}.

HRMS (ESI): calculated for $C_{34}H_{39}O_3$ $[M+H]^+$ requires m/z 495.2894, found m/z 495.2892 (*Δ* = -0.35 ppm).

(±)-(8*S*,9*S*,14*S*)-3-Methoxy-7,8,9,11,12,14,15,16-octahydro-6H-cyclopenta[*a*]phenanthrene 19.

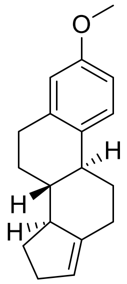

19
$C_{18}H_{22}O$
254.37 g mol^{-1}

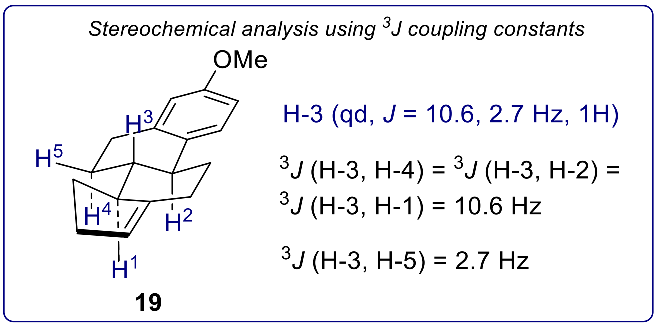

Alkene **S22** (20.0 mg**,** 40.4 µmol, 1.00 equiv) and 2nd generation Hoveyda-Grubbs catalyst (7.6 mg, 12.1 µmol, 0.300 equiv) were transferred to a microwave vial and purged with nitrogen for 15 min. Balloon of nitrogen was removed and degassed PhMe (0.81 mL) was added. The solution was stirred at 110 °C overnight, cooled to rt and volatiles were removed *in vacuo*. The crude product was purified by flash column chromatography (SiO_2; pentane to pentane:Et_2O; 100:0 to 98:2) to afford product **19** as a colurless oil (5.3 mg, 51%).

^{1}H NMR (600 MHz, $CDCl_3$) δ 7.24 (d, *J* = 8.6 Hz, 1H), 6.72 (dd, *J* = 8.7, 2.8 Hz, 1H), 6.62 (d, *J* = 2.8 Hz, 1H), 5.34 – 5.28 (m, 1H), 3.78 (s, 3H), 2.88 – 2.76 (m, 2H), 2.64 (ddd, *J* = 14.2, 4.2, 2.0 Hz, 1H), 2.50 – 2.40 (m, 2H), 2.35 – 2.25 (m, 3H), 2.25 – 2.13 (m, 2H), 1.98 (ddt, *J* = 12.8, 5.4, 2.8 Hz, 1H), 1.52 – 1.38 (m, 2H), 1.21 (qd, *J* = 11.9, 4.3 Hz, 1H), 1.04 (qd, *J* = 10.6, 2.7 Hz, 1H).

^{13}C NMR (101 MHz, $CDCl_3$): δ 157.4, 145.3, 138.5, 132.3, 127.4, 121.1, 113.8, 111.9, 55.3, 51.3, 49.2, 42.3, 32.0, 31.5, 30.6, 29.2, 28.7, 27.9.

IR (film) ν_{max}: 2927, 2854, 2360, 1611, 1501, 1254, 1237, 1048, 909, 802, 735 cm^{-1}.

HRMS (ESI): calculated for $C_{18}H_{23}O$ $[M+H]^+$ requires m/z 255.1743, found m/z 255.1744 (Δ = 0.35 ppm).

*4.11 Synthesis of a steroid analogue with a C-9 methyl group and ozonolysis of **2q**.*

(±)-(8*S*,9*S*,14*S*)-9-Methyl-7,8,9,11,12,14,15,16-octahydro-6H-cyclopenta[*a*]phenanthrene S23.

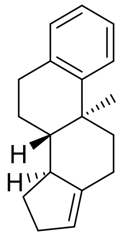

S23
$C_{18}H_{22}$
238.37 g mol^{-1}

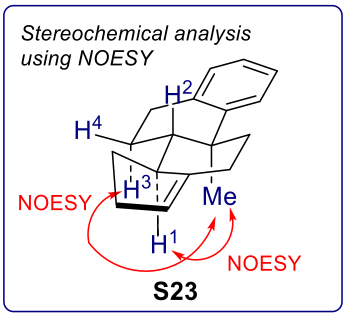

Alkene **6c** (25.2 mg**,** 52.6 µmol, 1.00 equiv) and 2nd generation Hoveyda-Grubbs catalyst (9.8 mg, 15.6 µmol, 0.30 equiv) were transferred to a microwave vial and purged with nitrogen for 15 min. Balloon of nitrogen was removed and degassed PhMe (1.1 mL) was added. The solution was stirred at 110 °C overnight, cooled to rt and volatiles were removed *in vacuo*. The crude product was purified by flash column chromatography (SiO_2; pentane) to afford product **S23** as a colurless oil (5.9 mg, 47%).

^{1}H NMR (600 MHz, $CDCl_3$): δ 7.32 (dd, *J* = 7.9, 1.4 Hz, 1H), 7.16 – 7.03 (m, 3H), 5.32 (t, *J* = 2.1 Hz, 1H), 2.92 – 2.83 (m, 2H), 2.53 – 2.43 (m, 2H), 2.40 – 2.31 (m, 2H), 2.31 – 2.24 (m, 2H), 2.23 – 2.16 (m, 1H), 1.83 – 1.77 (m, 1H), 1.67 – 1.58 (m, 1H), 1.46 – 1.37 (m, 2H), 1.25 (ddd, *J* = 12.4, 11.3, 2.8 Hz, 1H), 1.17 (s, 3H).

^{13}C NMR (101 MHz, $CDCl_3$) δ 147.5, 145.2, 135.7, 129.5, 125.8, 125.5, 125.4, 121.2, 50.9, 46.3, 38.0, 37.7, 31.2, 30.2 (2C), 25.0, 22.6, 22.2.

NOESY (600 MHz, $CDCl_3$): between H-1 and Me, between H-3 and Me.

Note: the appearance of ^{1}H NMR signal for H-2: 1.25 (ddd, *J* = 12.4, 11.3, 2.8 Hz, 1H, H-2) is indicative of a trans-diaxial arrangement between H-1 and H-2; H-3 and H-2 and equatorial/axial arrangement between H-4 and H-2.

IR (film) ν_{max}: 2929, 2844, 1488, 1433, 1373, 797, 758, 726 cm^{-1}.

HRMS (APCI): calculated for $C_{18}H_{23}$ $[M+H]^+$ requires m/z 239.1794, found m/z 239.1794 (*Δ* = 0.07 ppm).

(±)-2-((1*R*,2*R*,4*aS*,10*aS*)-1-(4-Methoxyphenyl)-4a-methyl-1,2,3,4,4*a*,9,10,10*a*-octahydrophenanthren-2-yl)acetaldehyde S24.

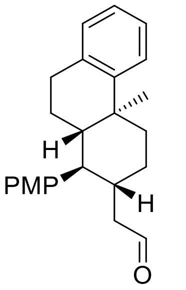

S24
$C_{24}H_{28}O_2$
348.49 g mol^{-1}

Alkene **2q** (27.6 mg, 61.0 µmol, 1.00 equiv) was dissolved in CH_2Cl_2 (20 mL) and the obtained solution was cooled to -78 °C. O_3/O_2 was bubbled through the solution for 5 min until the blue color was observed and then left to bubble through the solution for the additional 2 min. O_2 was bubbled until the blue color disappeared and then N_2 for the additional 10 min. The reaction mixture was quenched with DMS (1.8 mL) at -78 °C, left stirring at the same temperature for 30 min, warmed to rt and stirred for 4 h. Volatiles were removed *in vacuo* and the crude product was purified by flash column chromatography (SiO_2; 60 Å, 15–40 µm; pentane:Et_2O; 21:4) to afford aldehyde **S24** as a colurless oil (7.5 mg, 35%).

^{1}H NMR (400 MHz, $CDCl_3$): δ 9.45 (dd, *J* = 2.5, 1.6 Hz, 1H), 7.33 (dd, *J* = 7.8, 1.4 Hz, 1H), 7.19 – 6.96 (m, 5H), 6.91 (dd, *J* = 8.5, 2.8 Hz, 1H), 6.80 (dd, *J* = 8.3, 2.8 Hz, 1H), 3.81 (s, 3H), 2.80 – 2.63 (m, 2H), 2.42 – 2.28 (m, 2H), 2.28 – 2.20 (m, 1H), 2.18 – 2.06 (m, 2H), 1.95 – 1.87 (m, 1H), 1.79 (ddd, *J* = 12.5, 11.3, 3.0 Hz, 1H), 1.74 – 1.60 (m, 2H), 1.47 – 1.32 (m, 1H), 1.20 (m, 4H).

^{13}C NMR (101 MHz, $CDCl_3$) δ 202.6, 158.4, 147.7, 135.6, 135.4, 132.3, 129.4, 126.1, 125.8, 125.7, 124.7, 115.4, 113.1, 55.4, 49.7, 49.5, 47.5, 39.6, 37.5, 37.4, 29.5, 28.8, 22.7, 22.6.

IR (film) ν_{max}: 2935, 2359, 1721, 1611, 1512, 1247, 1178, 1036, 830, 762 cm^{-1}.

HRMS (ESI): calculated for $C_{24}H_{29}O_2$ $[M+H]^+$ requires m/z 349.2162, found m/z 349.2162 (*Δ* = -0.03 ppm).

5.0 Control and mechanistic experiments.

5.1 Stereoconvergent experiments.

Stereoconvergent synthesis of **2a**.

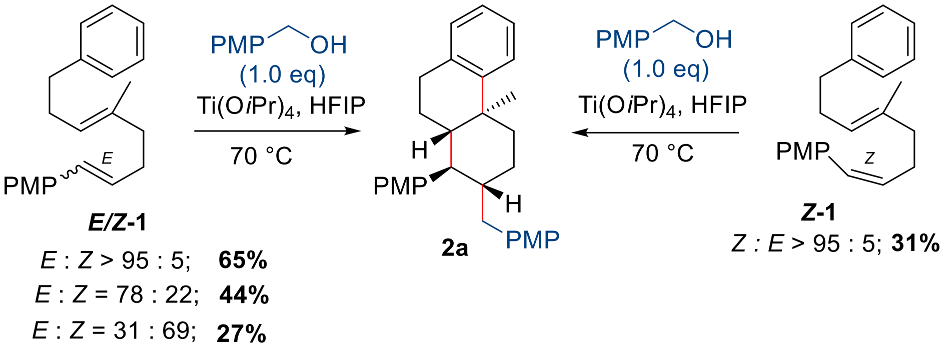

Experimental procedure: E/Z-**1** (0.100 mmol, 1.00 equiv), *E*-**1** (0.0816 mmol, 1.00 equiv) or Z-**1** (0.0816 mmol, 1.00 equiv), 4-methoxybenzyl alcohol (1.00 equiv) and Ti(O*i*Pr)$_4$ (0.30 equiv) in HFIP (0.050 M) were subjected to the general procedure **B**. The crude mixture was analysed by NMR and purified by flash column chromatography (SiO_2; 60 Å, 15–40 µm, pentane:Et_2O; 24:1) to furnish compound **2a** as a colourless oil which crystallised on standing. Only single diastereomer of **2a** could be detected in the crude ^{1}H and ^{13}C NMR prior to column chromatography.

Stereoconvergent synthesis of **2m**.

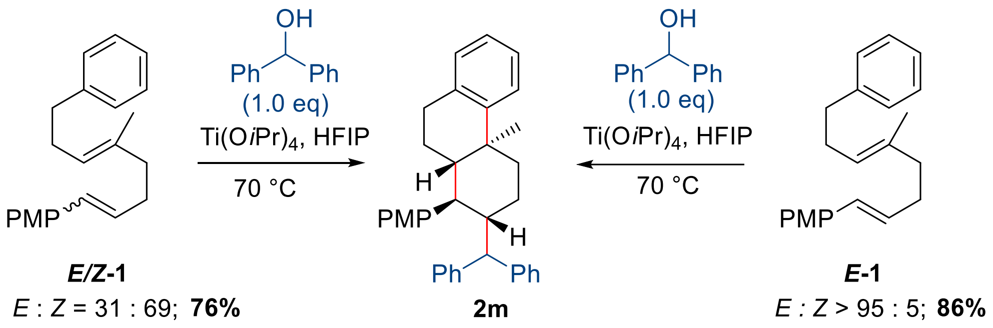

Experimental procedure: E/Z-**1** (0.100 mmol, 1.00 equiv) or *E*-**1** (0.100 mmol, 1.00 equiv), benzhydrol (1.00 equiv) and Ti(O*i*Pr)$_4$ (0.30 equiv) in HFIP (0.050 M) were subjected to the general procedure **B**. The crude mixture was analysed by NMR and purified by flash column chromatography (SiO_2; pentane:Et_2O; 49:1) to furnish compound **2m** as a foam. Only single diastereomer of **2m** could be detected in the crude prior to column chromatography.

Stereoconvergent synthesis of **6a**.

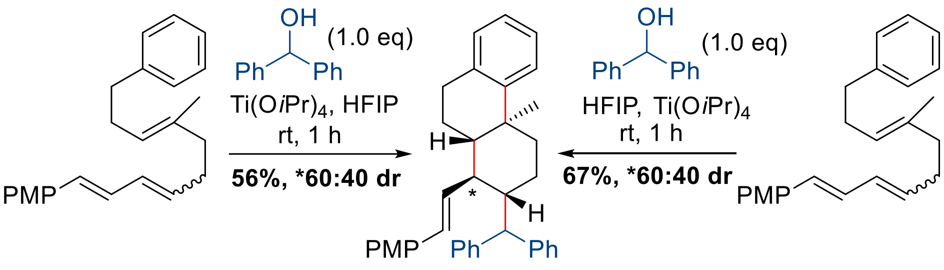

*Experimental procedure: E/Z-***4** (0.100 mmol, 1.00 equiv), benzhydrol (0.100 mmol, 1.00 equiv) and Ti(O*i*Pr)$_4$ (0.30 equiv) in HFIP (0.050 M) were subjected to the general procedure **C**. The crude product was purified by flash column chromatography (SiO_2; pentane:Et_2O; 24:1) to furnish compound **6a** as an inseparable mixture of two diastereomers at the exocyclic allylic position as a colourless oil (60:40 dr).[59] dr of the crude reaction mixture was found to be independent of the *E*/*Z* ratio of the triene **4**.

*5.2 Stability of Z-**1**, E-**1**, E/Z-**4** under the reaction conditions.*

Z-**1** stability/interconversion.

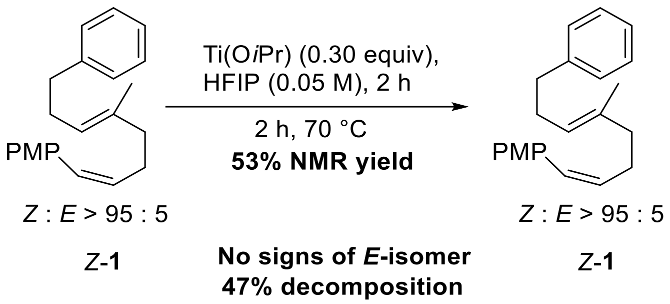

*Experimental procedure: Z-***1** (1.00 equiv) and Ti(O*i*Pr)$_4$ (0.30 equiv) in HFIP (0.050 M) were subjected to the general procedure **B** but without electrophile present. NMR analysis of the crude reaction mixture using 1,1,2,2-tetrachloroethane as an internal standard indicated the presence of *Z*-**1** (53% NMR yield) together with complex mixture of unidentified decomposition products. No *E*-isomer was detected.

[59] dr determined by ^{1}H NMR of the crude reaction mixture.

E-**1** stability/interconversion.

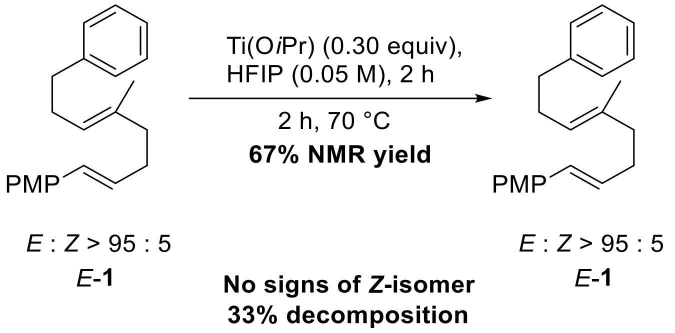

Experimental procedure: E-**1** (1.00 equiv) and Ti(O*i*Pr)$_4$ (0.30 equiv) in HFIP (0.050 M) were subjected to the general procedure **B** but without electrophile present. NMR analysis of the crude reaction mixture using 1,1,2,2-tetrachloroethane as an internal standard indicated the presence of *E*-**1** (67% NMR yield) together with complex mixture of unidentified decomposition products. No *Z*-isomer was detected.

E/Z-**4** stability/interconversion.

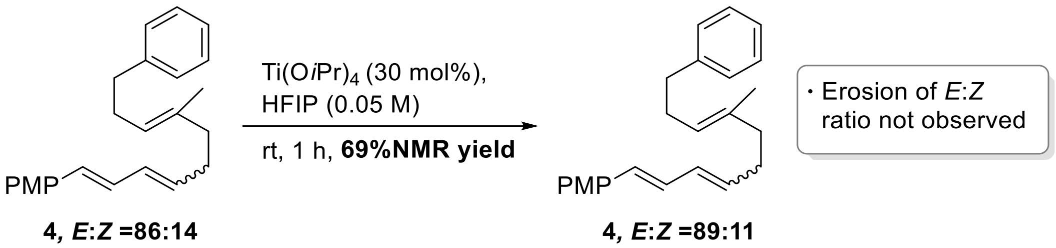

Experimental procedure: E/Z-**4** (1.00 equiv, 86:14 *E*/*Z*) and Ti(O*i*Pr)$_4$ (0.30 equiv) in HFIP (0.050 M) were subjected to the general procedure **C** but without electrophile present. NMR analysis of the crude reaction mixture using 1,1,2,2-tetrachloroethane as an internal standard indicated the presence of *E/Z*-**4** (67% NMR yield, 89:11 *E*/*Z*) together with complex mixture of unidentified decomposition products.

5.3 Reaction with electron-deficient benzyl alcohol.

When 4-cyanobenzyl alcohol was used in the reaction, no desired product was isolated or observed. Instead, the alcohol was isolated in quantitative yield after column chromatography together with the mixture of starting material **1** and protonated carbocycle **1'**. This is consistent with S_N1 mechanism: 4-cyanobenzyl alcohol does not ionise under the reaction conditions as it cannot form sufficiently stabilised carbocation. Instead, cyclisation proceeds by protonation of a diene **1** catalysed by HFIP solvent.

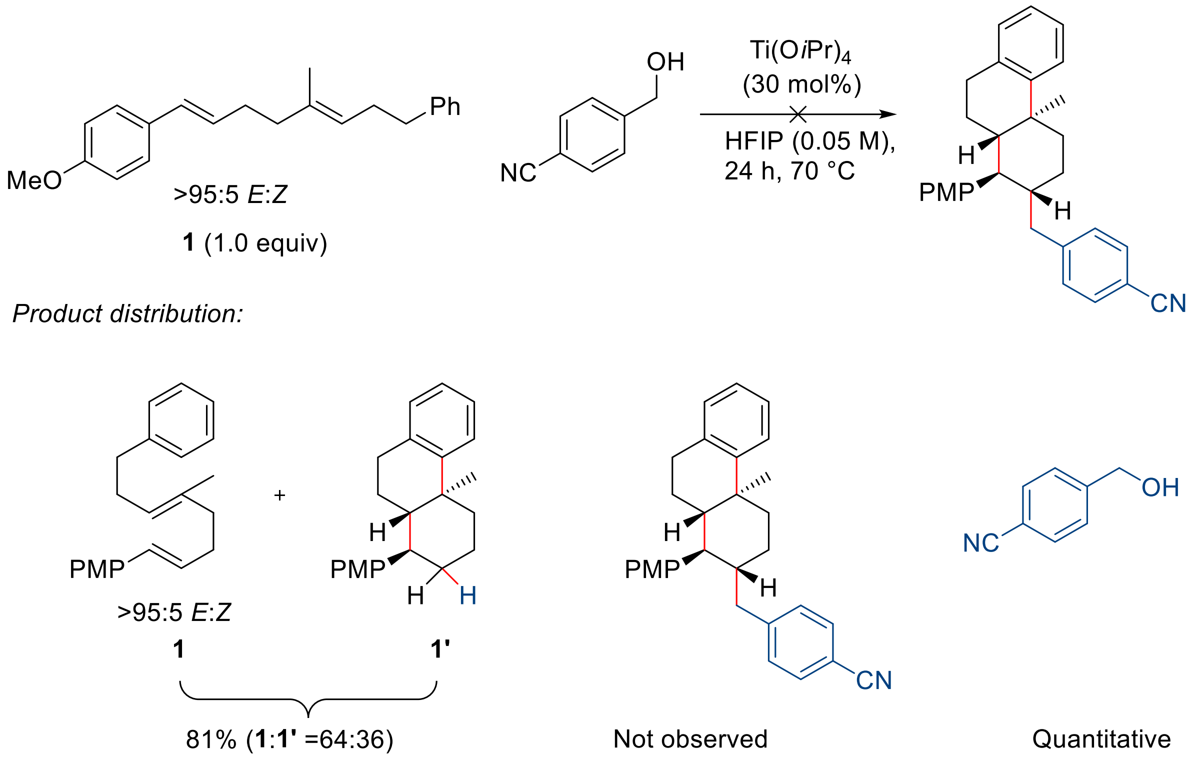

Experimental procedure: Diene **1** (30.6 mg, 0.100 mmol, 1.00 equiv), 4-cyanobenzyl alcohol (13.3 mg, 0.100 mmol, 1.00 equiv) and Ti(O*i*Pr)$_4$ (9.0 µL, 30 µmol, 0.30 equiv) in HFIP (2.0 mL, 0.050 M) were subjected to the general procedure **B**. The crude product was purified by flash column chromatography (SiO_2; 60 Å, 15–40 µm, pentane:Et_2O; 24:1 to 0:100) to furnish an inseparable mixture of **1** and **1'** (24.9 mg, 81%, **1**:**1'** = 64:36) as a colourless oil and 4-cyanobenzyl alcohol (quant) as a colourless oil.

5.4 Reaction with transposed isomers of unsymmetrical allylic alcohol.

The use of transposed isomers **S15** and **S13** of unsymmetrical allylic alcohol furnished the same product **2w** in 77% and 44% yield respectively**.** This is consistent with S_N1 reaction mechanism: **S15** and **S13** ionise under the reaction conditions to form the same delocalised carbocation **SI** which then initiates polyene cyclisation. The external C-C bond is formed at the least hindered (secondary) end of a delocalised system **SI**. This is consistent with our previous observation that a new exocyclic C-C bond could not be formed using tertiary carbocations, possibly due to steric hindrance.

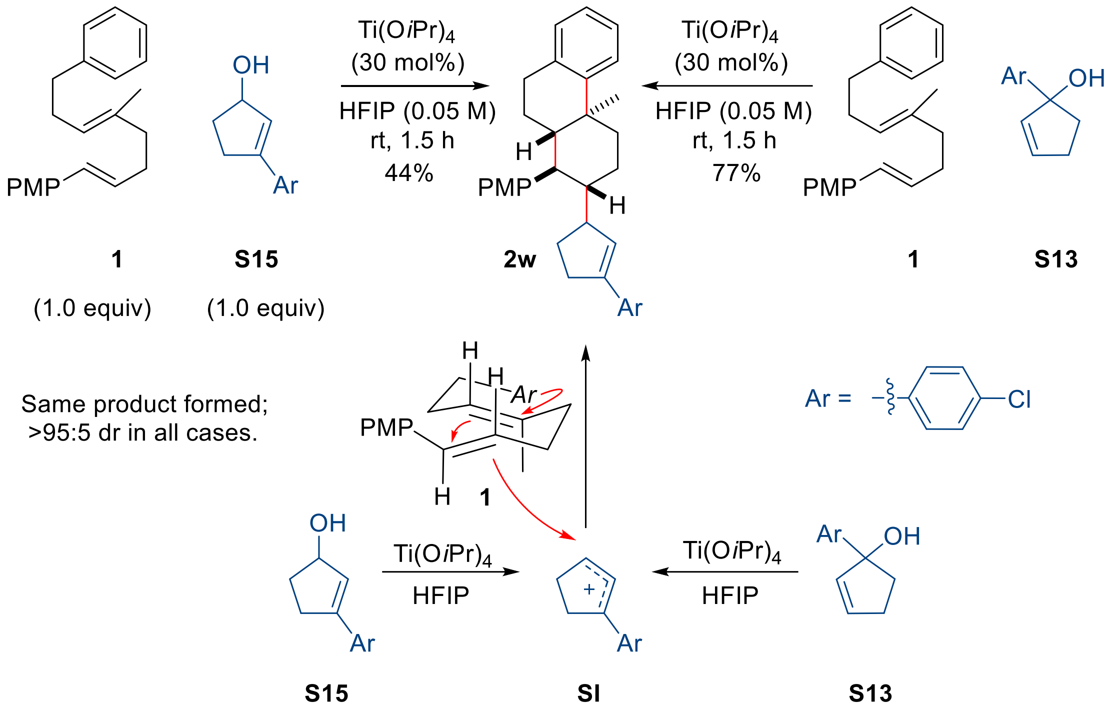

Experimental procedure: Diene **1** (30.6 mg, 0.100 mmol, 1.00 equiv), alcohol **S15** or **S13** (19.5 mg, 0.100 mmol, 1.00 equiv) and Ti(O*i*Pr)$_4$ (9.0 µL, 30 µmol, 0.30 equiv) in HFIP (2.0 mL, 0.050 M) were subjected to the general procedure **B** except the reaction was performed at rt and for 1.5 h. The crude product was purified by flash column chromatography (SiO_2; 60 Å, 15–40 µm, pentane:Et_2O; 24:1) to furnish compound **2w** as a single detectable diastereomer as a bage foam (77%, >95:5 d.r. for **S13** and 44%, >95:5 d.r. for **S15**).[60]

[60] The exocyclic stereochemistry could not be unambiguously determined.

6.0 Single crystal X-ray diffraction for compounds 2a, 2d, 2g, 12 and 14.

Single Crystal Data for **2g**

$C_{248}H_{288}O_{16}$

M_r 3525.00

150 K

Monoclinic *C* 2/c

a = 23.5285(2) Å, b = 23.8965(2) Å, c = 34.6674(4) Å

α= 90° β= 97.2217(9)° γ = 90°

V = 19337.1(3) Å^3

Data/restraints/parameters – 16043/ 313/1373

[R(int) = 0.058

Final R1 = 0.0921, wR2 = 0.2392 [I>2sigma(I)]

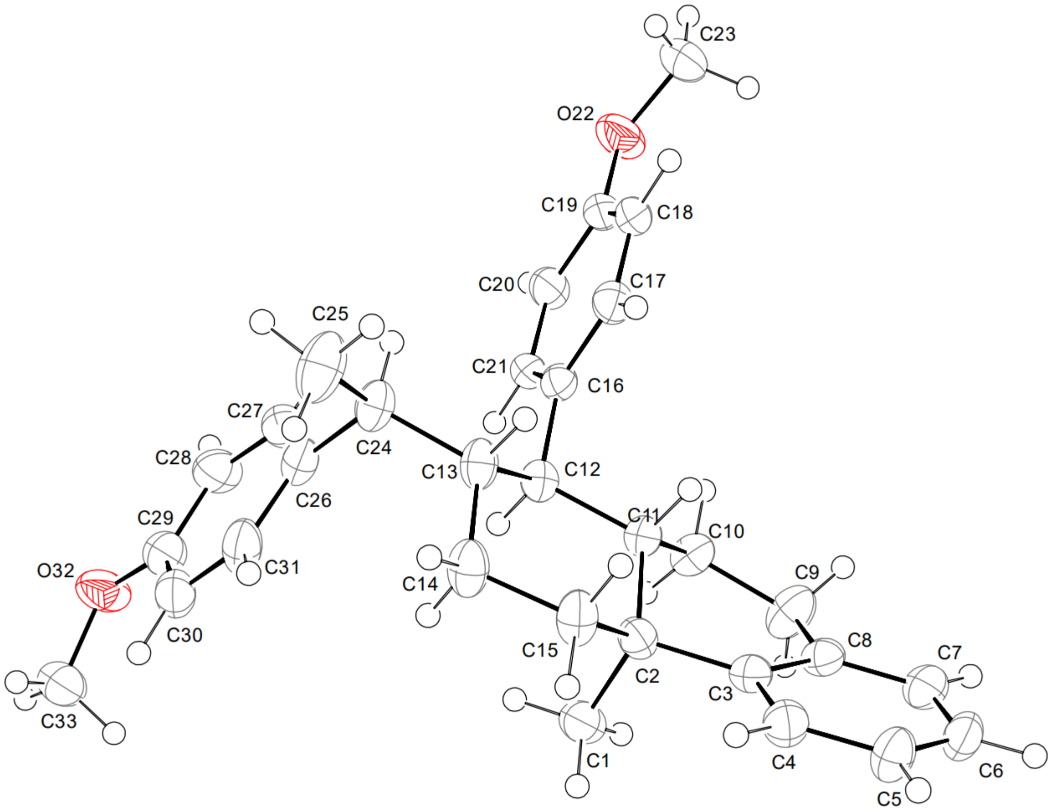

This structure contains four molecules in the asymmetric unit. For clarity only one molecule is shown. Additional molecules are off-set in the numbering scheme by 50 and any disorder is off-set by 500.

Single Crystal Data for **2d**

$C_{31}H_{36}O_3$

M_r 456.62

150 K

Monoclinic *P* 2_1/n

a = 5.75750(10) Å, b = 31.6859(6) Å, c = 13.7088(3) Å

α= 90° β= 94.198(2)° γ = 90°

V = 2494.21(8) Å^3

Data/restraints/parameters – 5158/ 0/ 307

R(int) = 0.034

R1 = 0.0397, wR2 = 0.0962 [I>2sigma(I)]

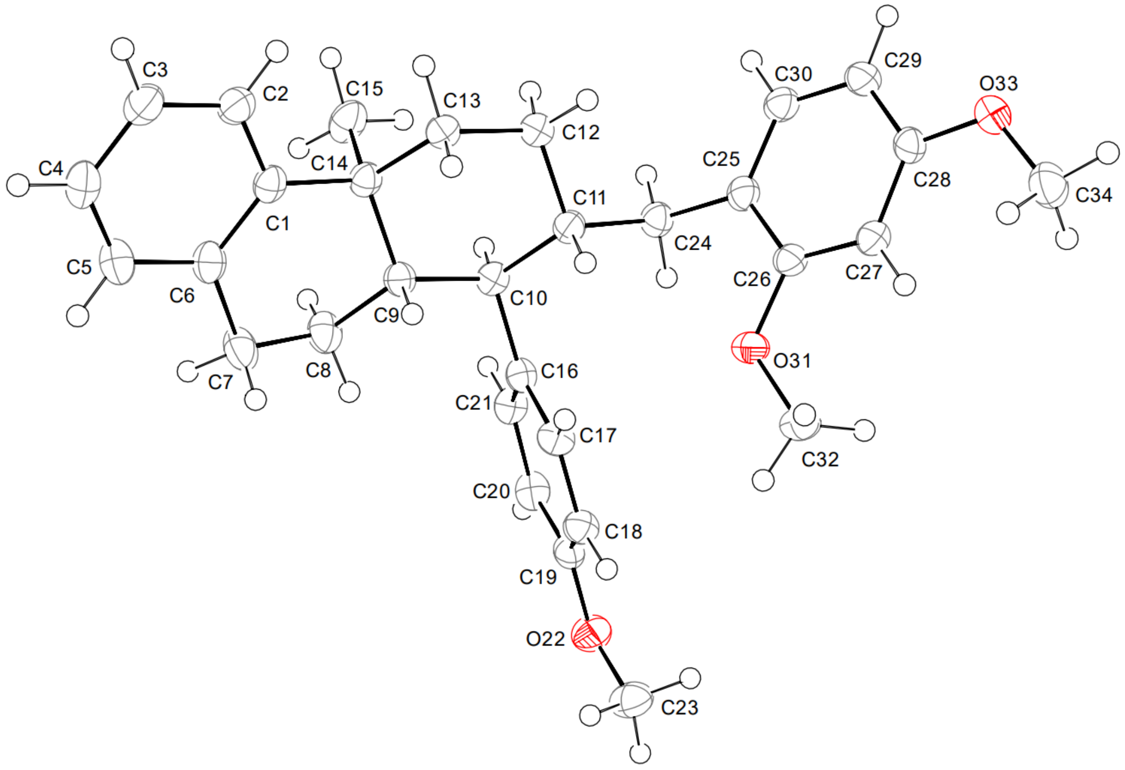

Single Crystal Data for **2a**

$C_{30}H_{34}O_2$

M_r 426.60

150 K

Monoclinic *P* 2_1/c

a = 10.0874(5) Å, b = 15.3761(6) Å, c = 15.3706 (6) Å

α= 90° β= 101.820(5)° γ = 90°

V = 2333.50(18) Å^3

Data/restraints/parameters – 4864/0/ 289

R(int) = 0.097

R1 = 0.0676, wR2 = 0.1701 [I>2sigma(I)]

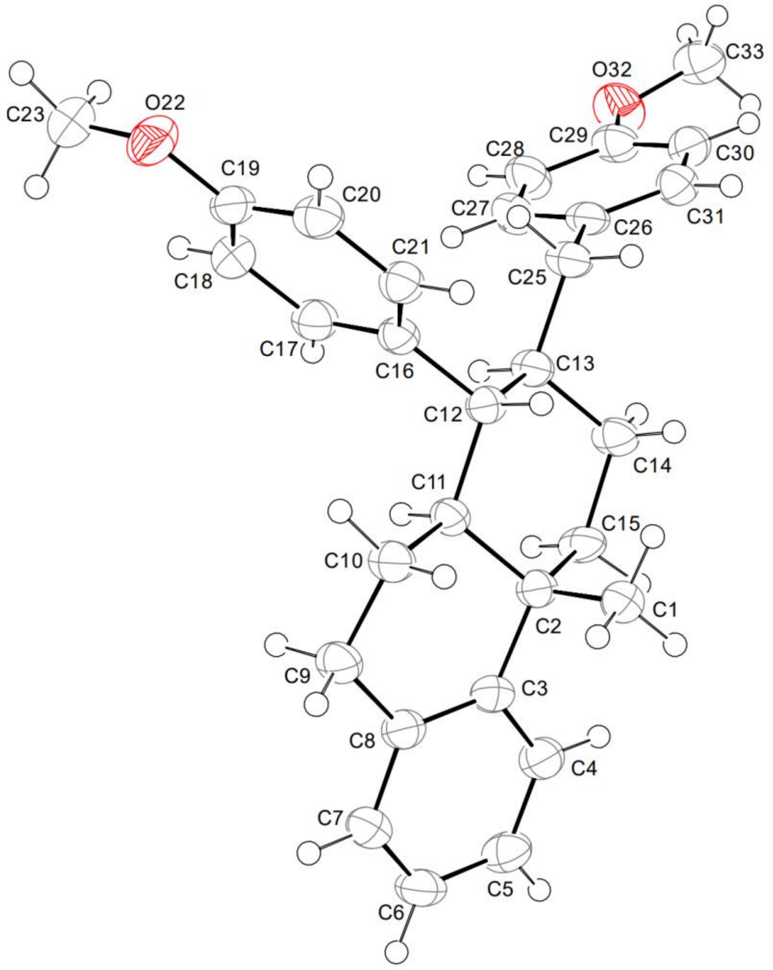

Single Crystal Data for **12**

$C_{36}H_{38}O_2$

M_r 502.70

150 K

Triclinic *P* -1

a = 10.1264(3) Å, b = 10.8819(3) Å, c = 13.4382(4) Å

α= 88.808(3)° β= 77.853(3)° γ = 76.137(3)°

V = 1404.81(7) Å^3

Data/restraints/parameters – 5813/0/343

R(int) = 0.025

R1 = 0.0359, wR2 = 0.0960 [I>2sigma(I)]

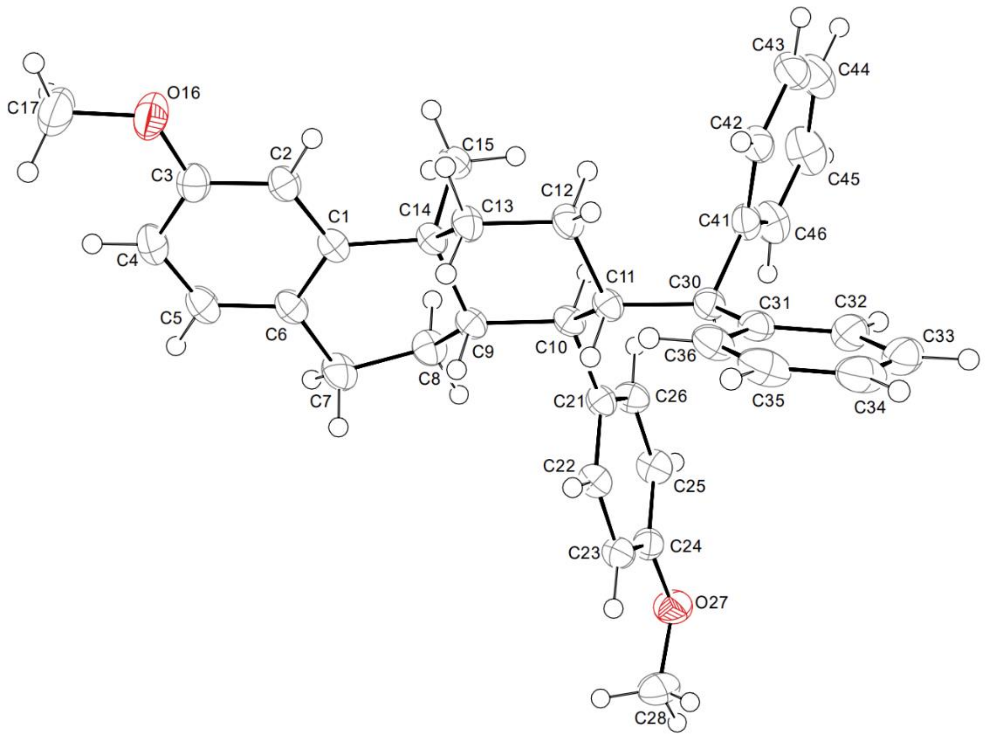

Single Crystal Data for **14**

$C_{36}H_{38}O$

M_r 486.70

150 K

Triclinic *P* -1

a = 10.0988(3) Å, b = 11.1576(4) Å, c = 12.6763(3) Å

α= 89.681(2)° β= 77.820(2)° γ = 76.018(3)°

V = 1353.27(7) Å^3

Data/restraints/parameters – 5521/0/335

R(int) = = 0.024

R1 = 0.0415, wR2 = 0.0970 [I>2sigma(I)]

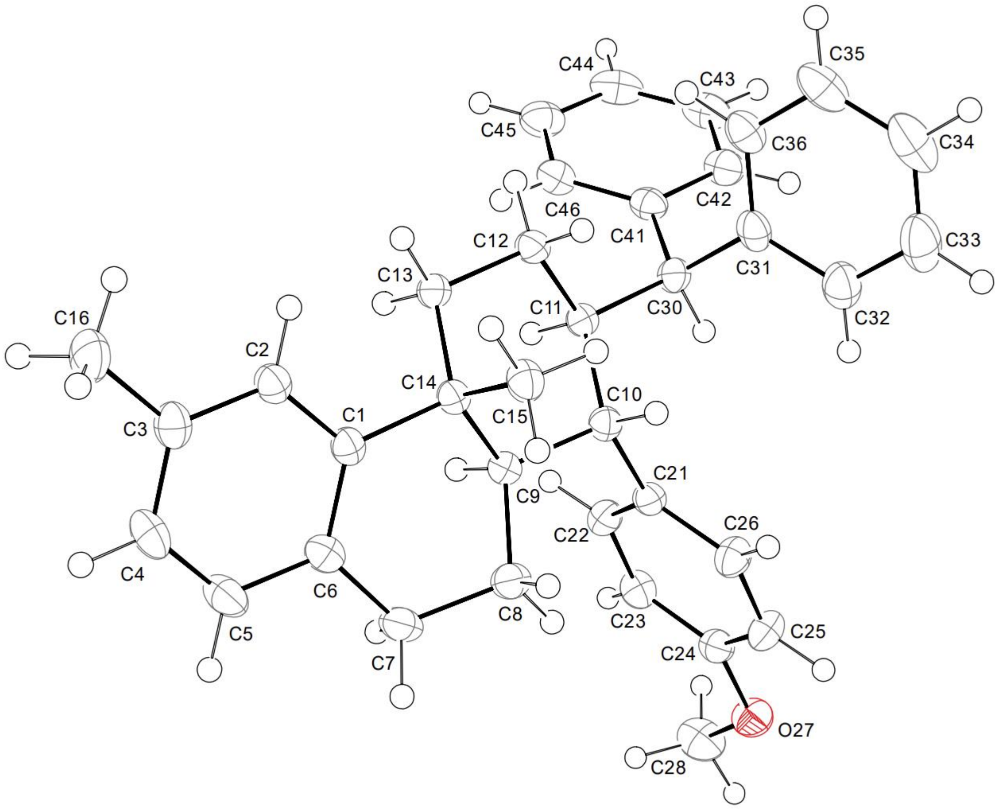

7.0 ^{1}H, ^{13}C and representative 2D NMR spectra.

7.1 Polyene cyclisation precursors and intermediates.

Tributyl(4-methoxybenzyl)phosphonium chloride S2.

^{1}H NMR (400 MHz, $CDCl_3$)

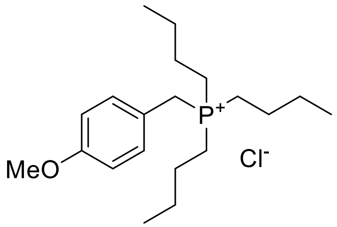

S2

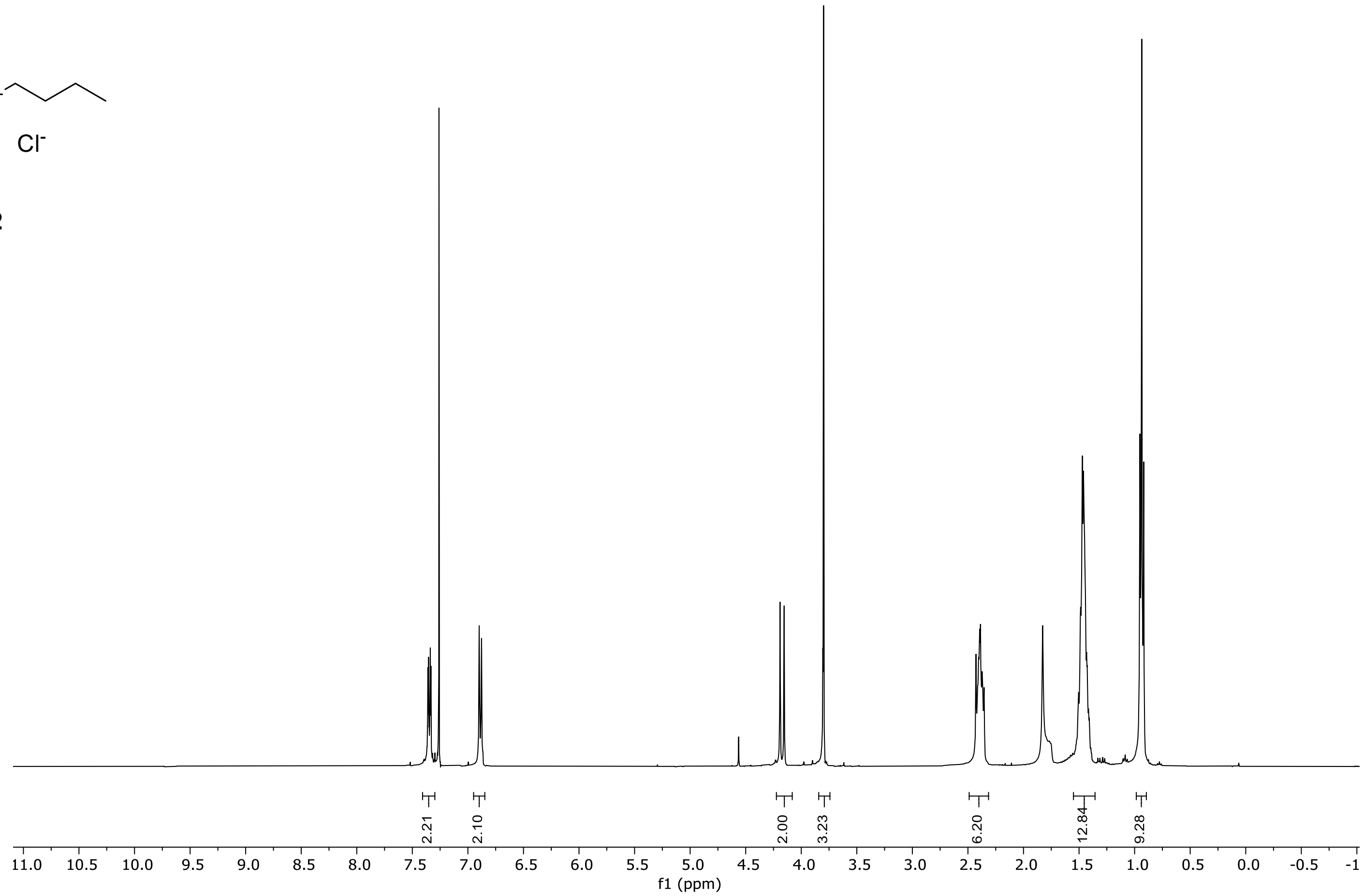

^{13}C NMR (101 MHz, $CDCl_3$)

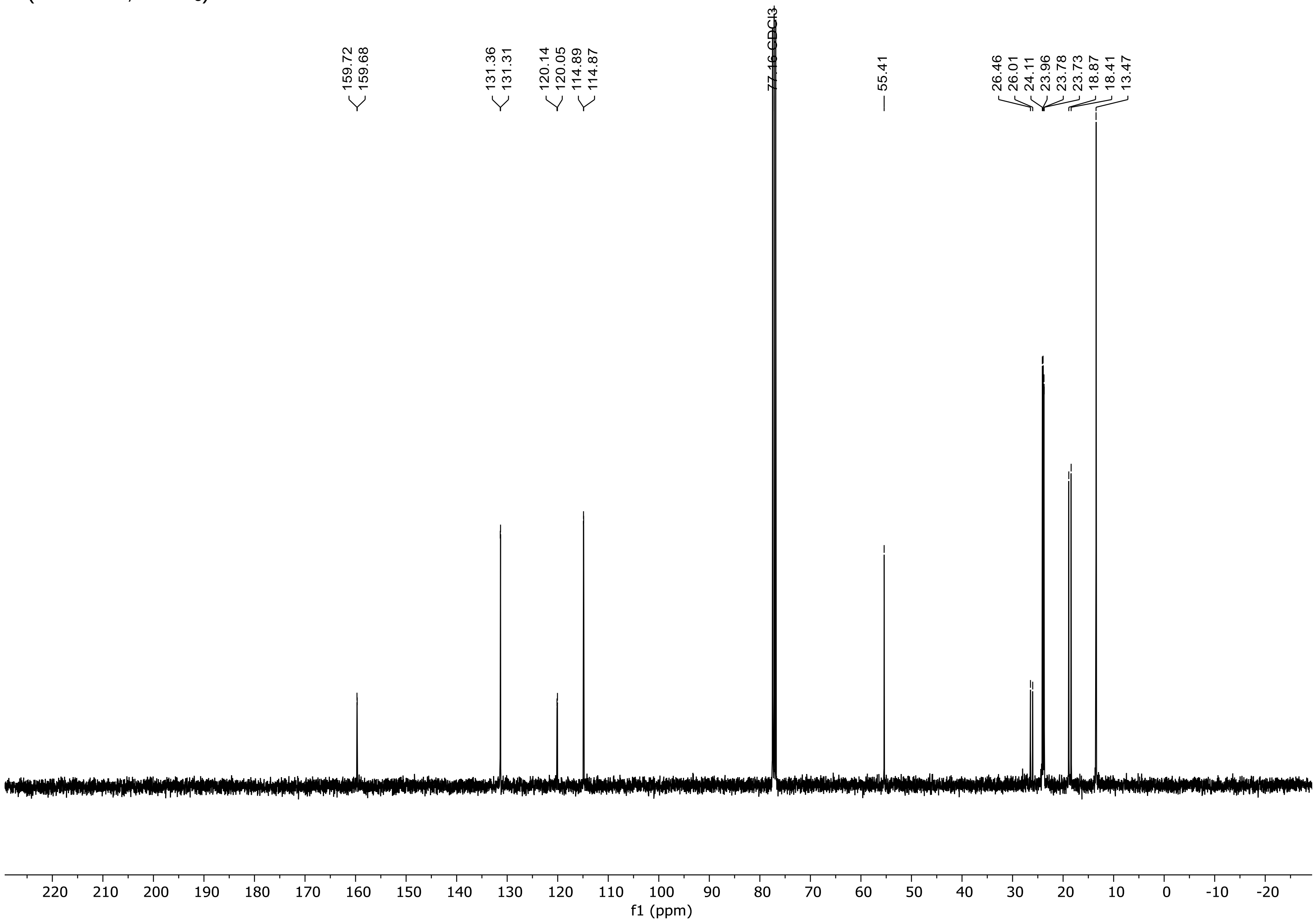

^{31}P NMR (162 MHz, $CDCl_3$)

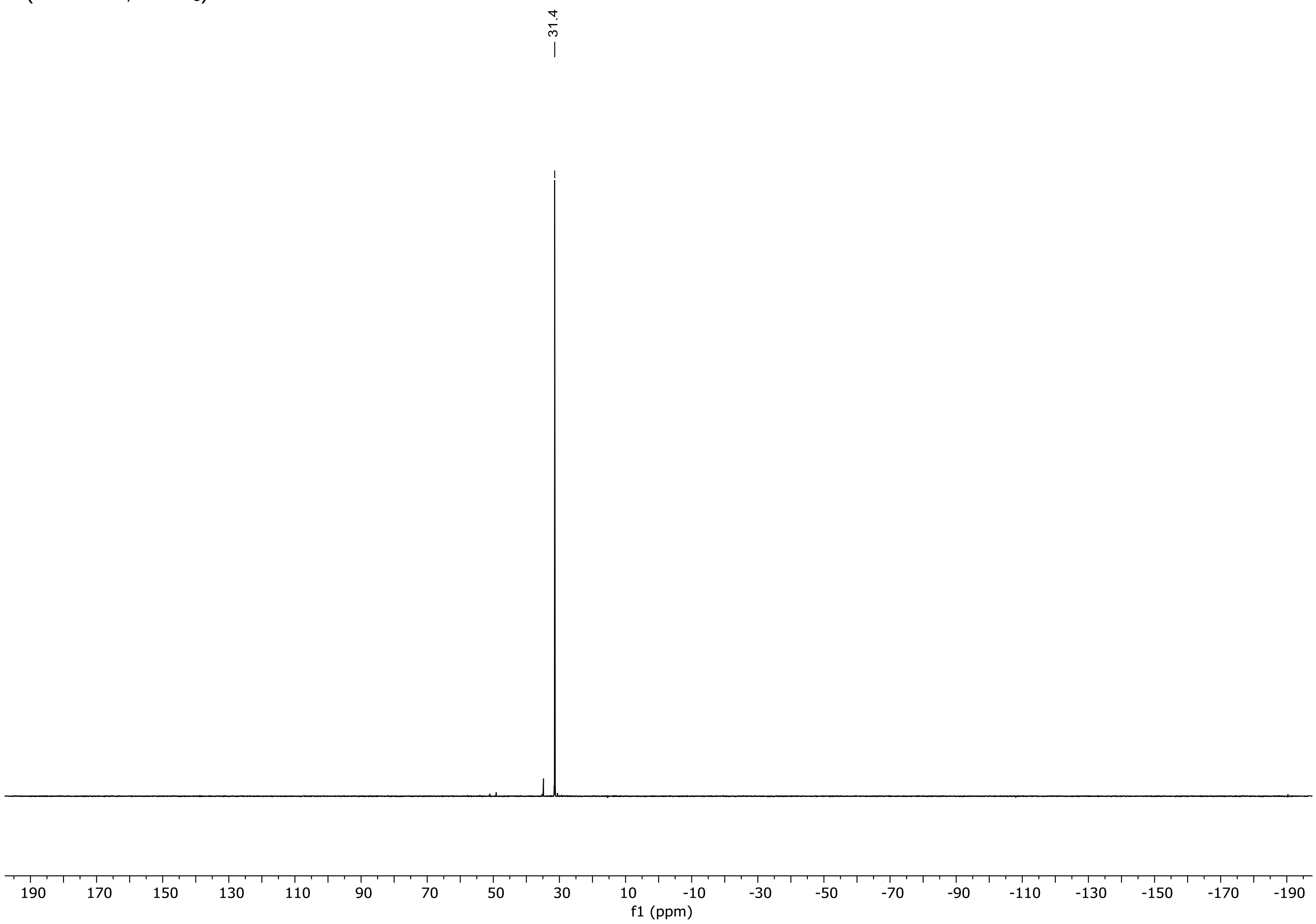

(2*E*,6*E*)-7-(4-methoxyphenyl)-3-methylhepta-2,6-dien-1-yl acetate *E*-S3 and (2*E*,6*Z*)-7-(4-methoxyphenyl)-3-methylhepta-2,6-dien-1-yl acetate *Z*-S3.

^{1}H NMR (400 MHz, $CDCl_3$)

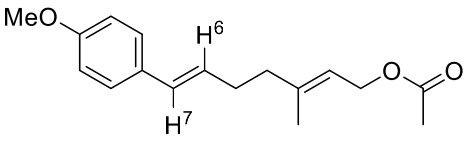

E-**S3** (Major)

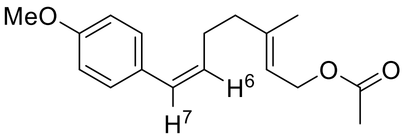

Z-**S3** (Minor)

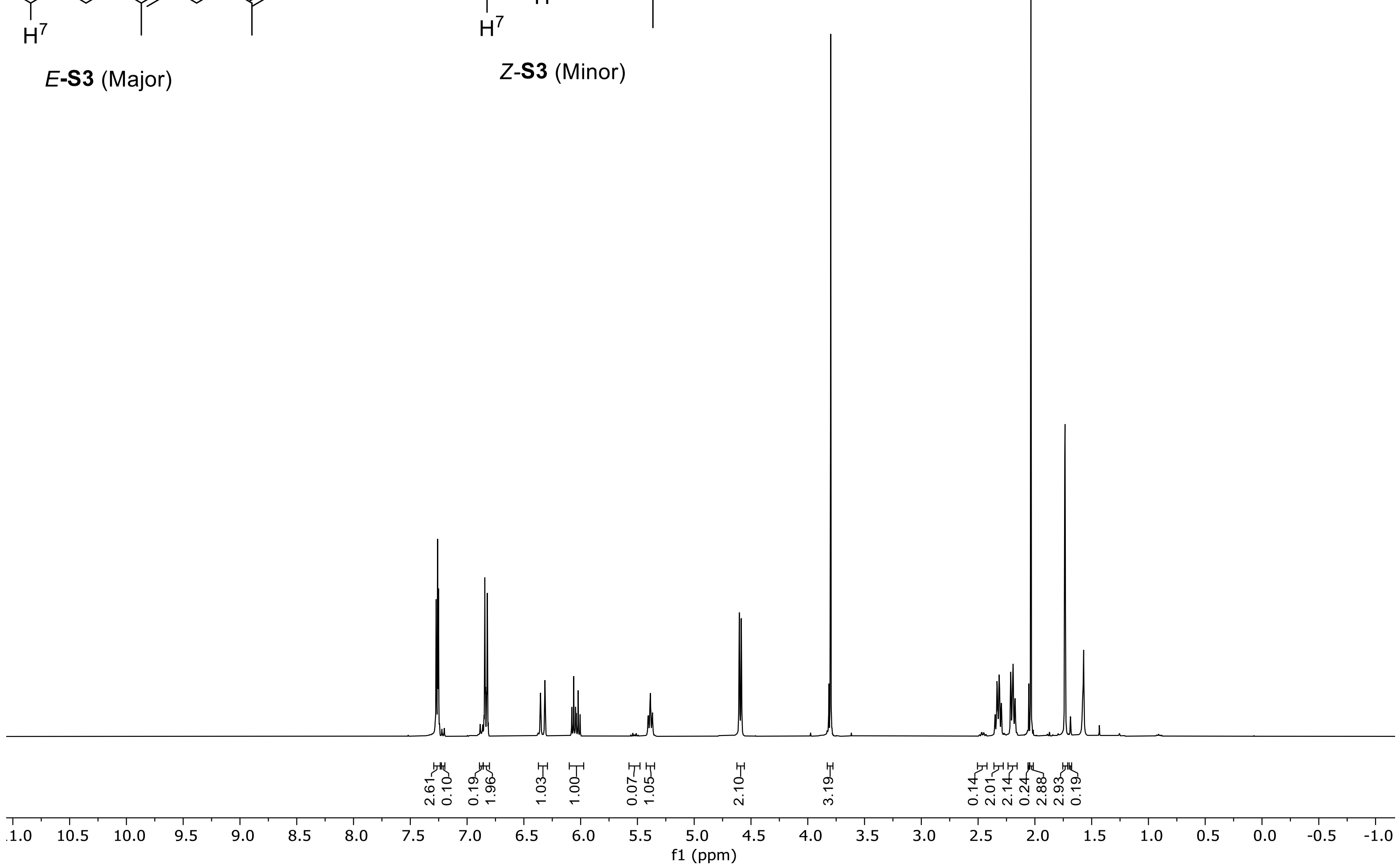

^{13}C NMR (101 MHz, $CDCl_3$)

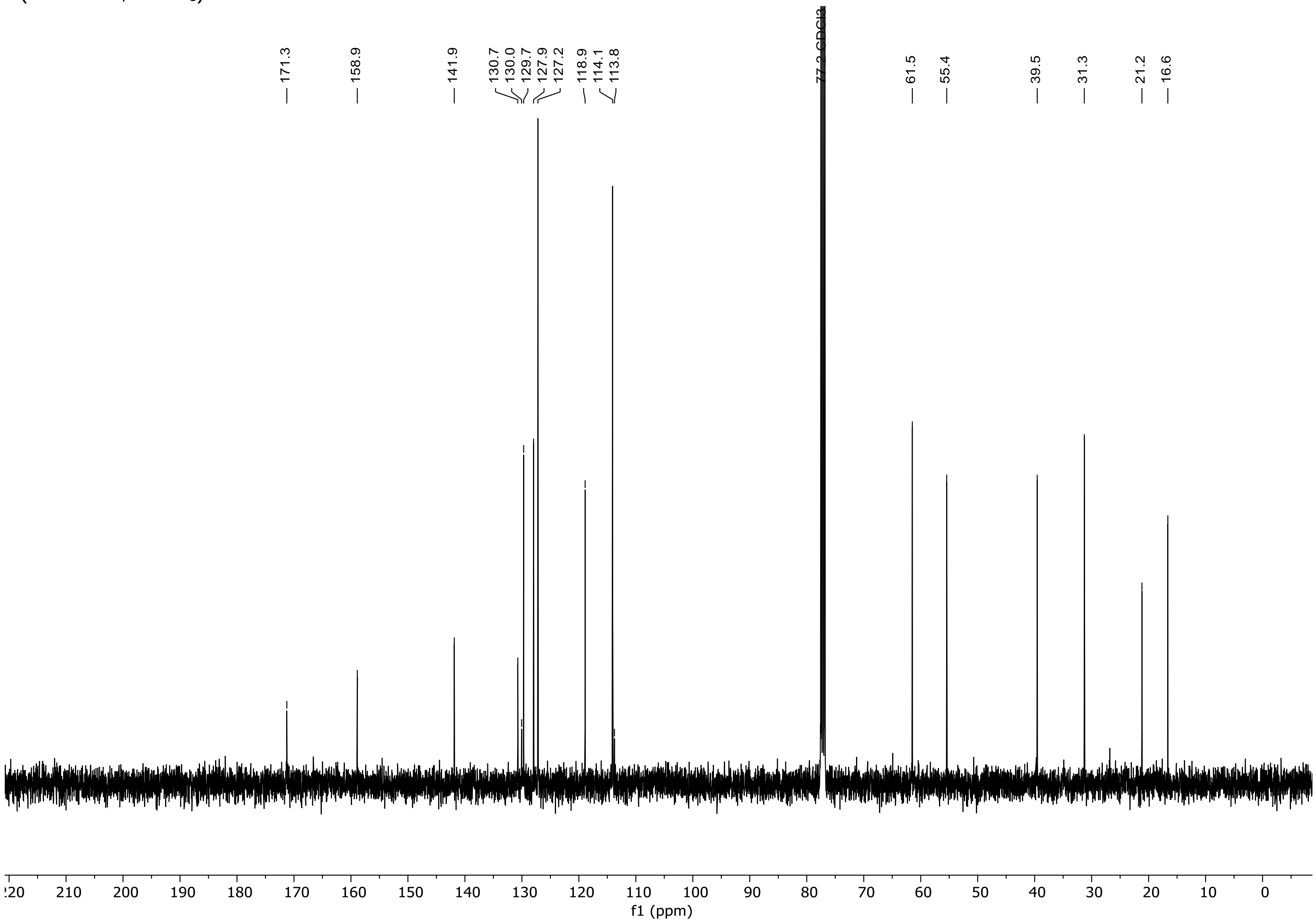

(*E*)-7-Phenylhept-4-enal S9.

^{1}H NMR (400 MHz, $CDCl_3$)

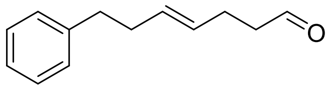

S9

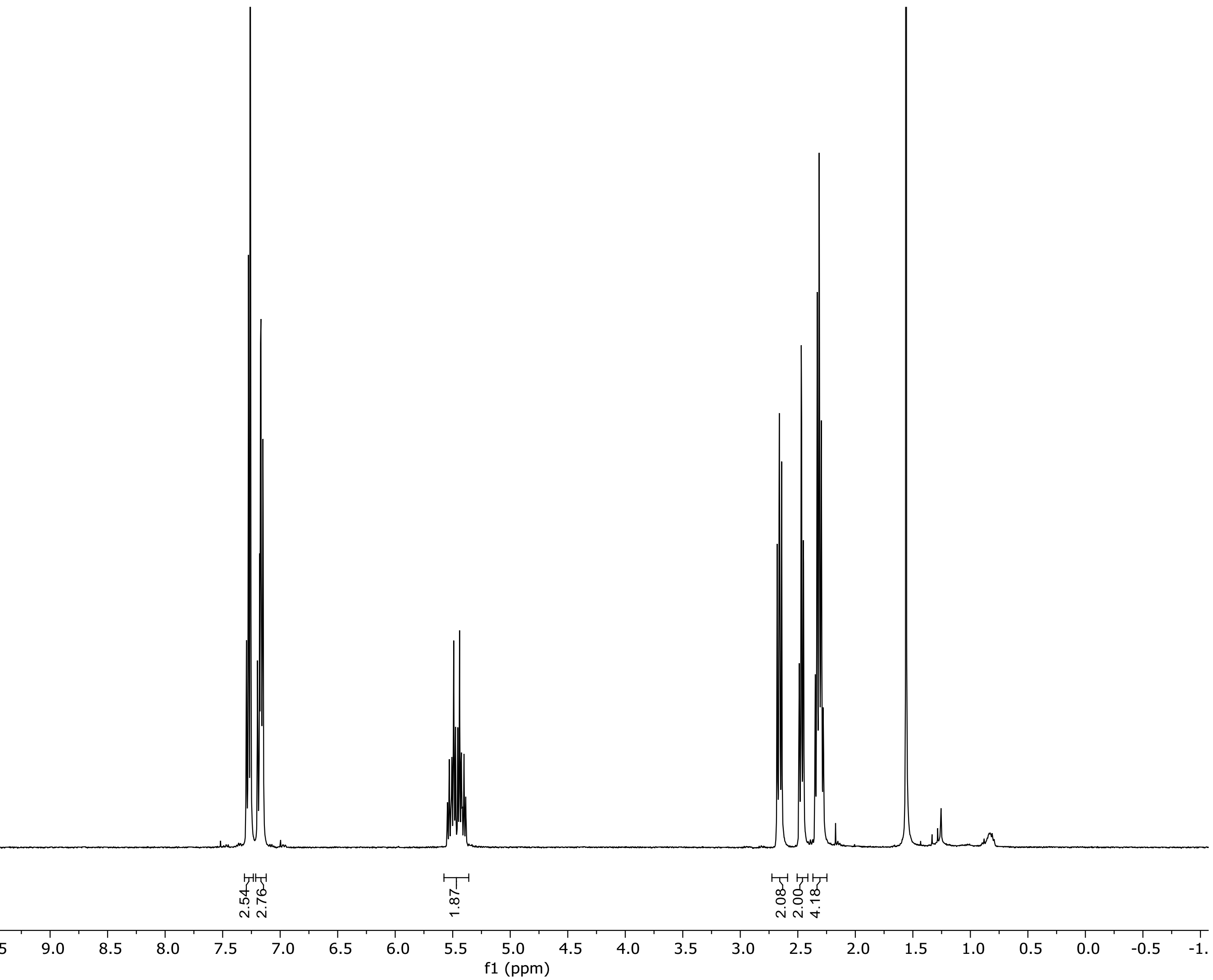

^{13}**C NMR** (101 MHz, $CDCl_3$)

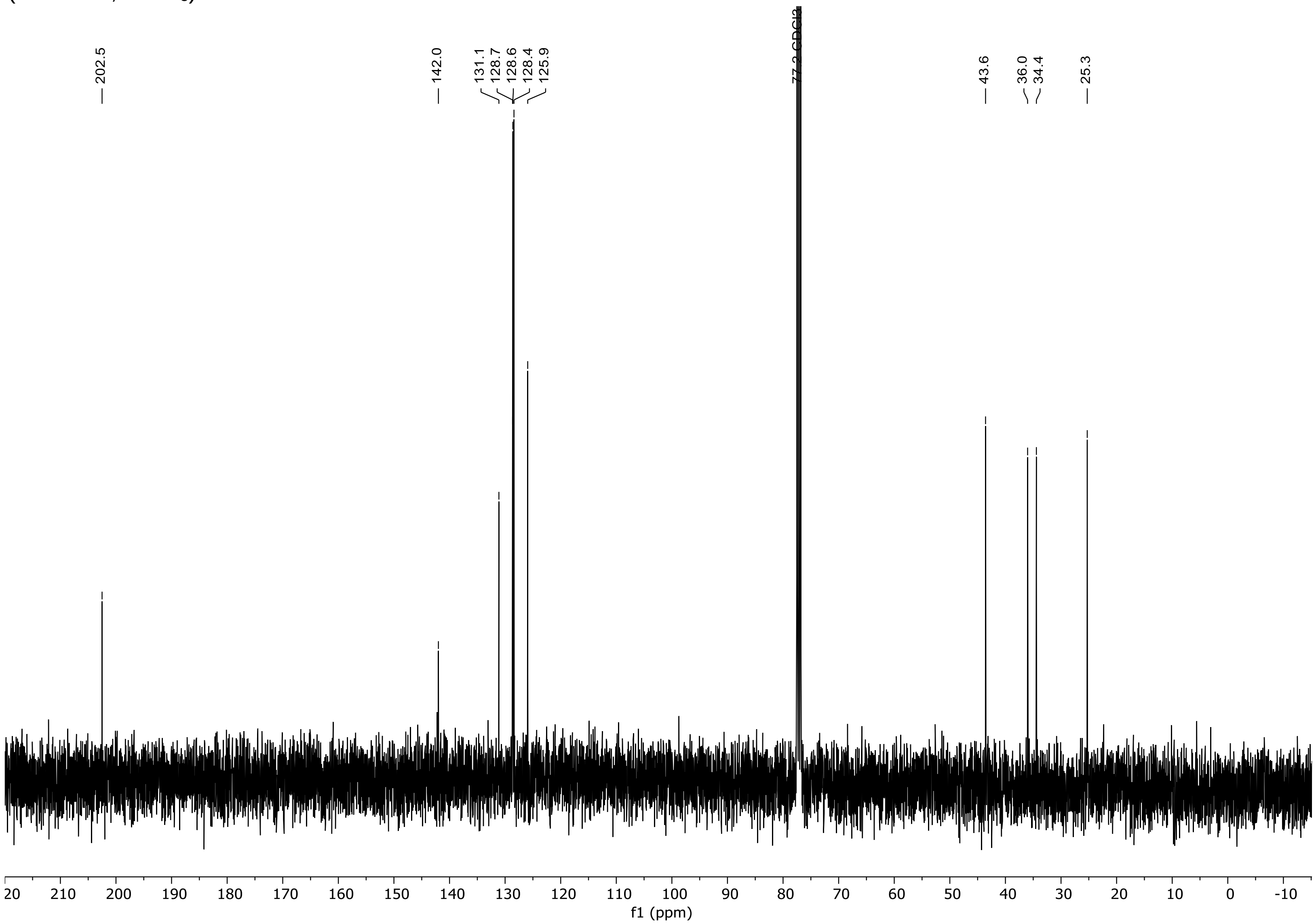

(2*E*,6*E*,8*E*)-9-(4-methoxyphenyl)-3-methylnona-2,6,8-trien-1-yl acetate (*E*)-S10 and (2*E*,6*Z*,8*E*)-9-(4-methoxyphenyl)-3-methylnona-2,6,8-trien-1-yl acetate (*Z*)-S10.

^{1}**H NMR** (400 MHz, $CDCl_3$)

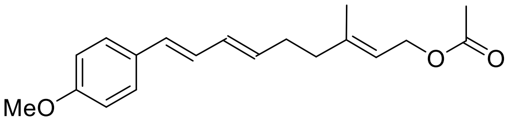

(***E***)**-**major

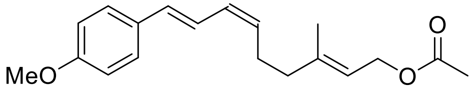

(***Z***)**-**minor

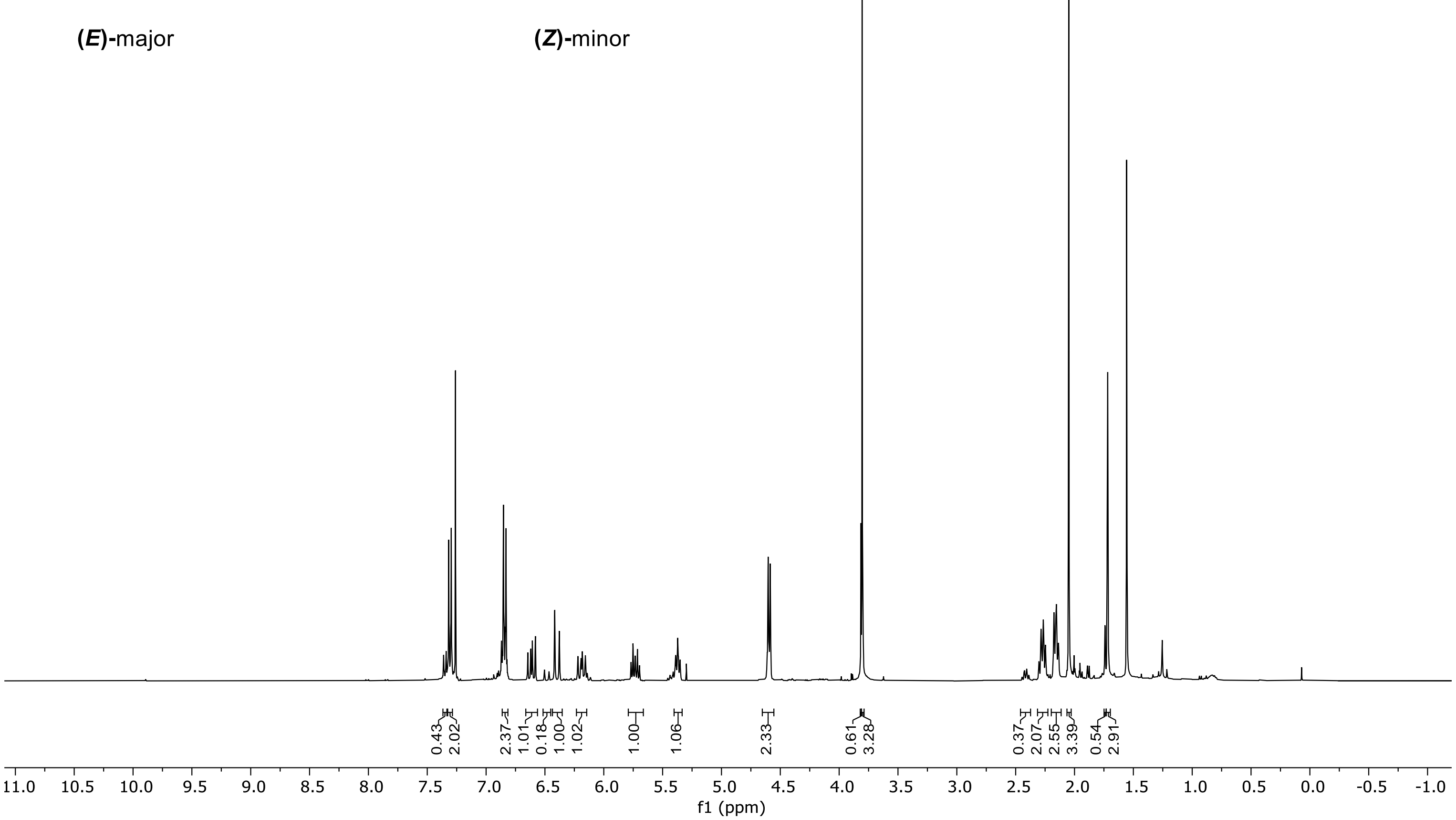

^{13}C NMR (101 MHz, $CDCl_3$)

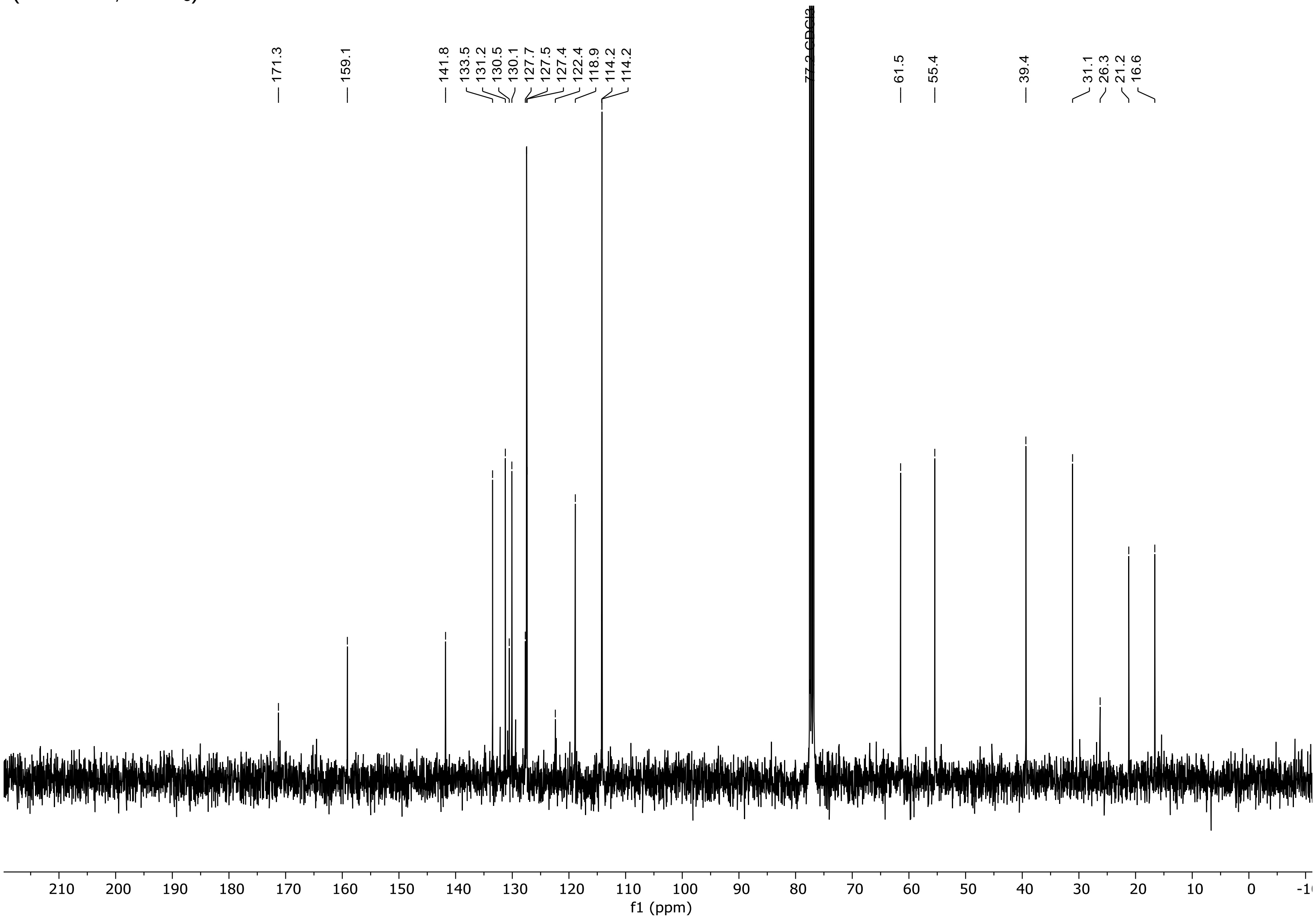

(*E*)-(3-(4-methoxyphenyl)allyl)triphenylphosphonium bromide S11 (used impure).

^{1}H NMR (400 MHz, $CDCl_3$)

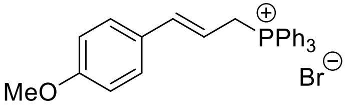

S11

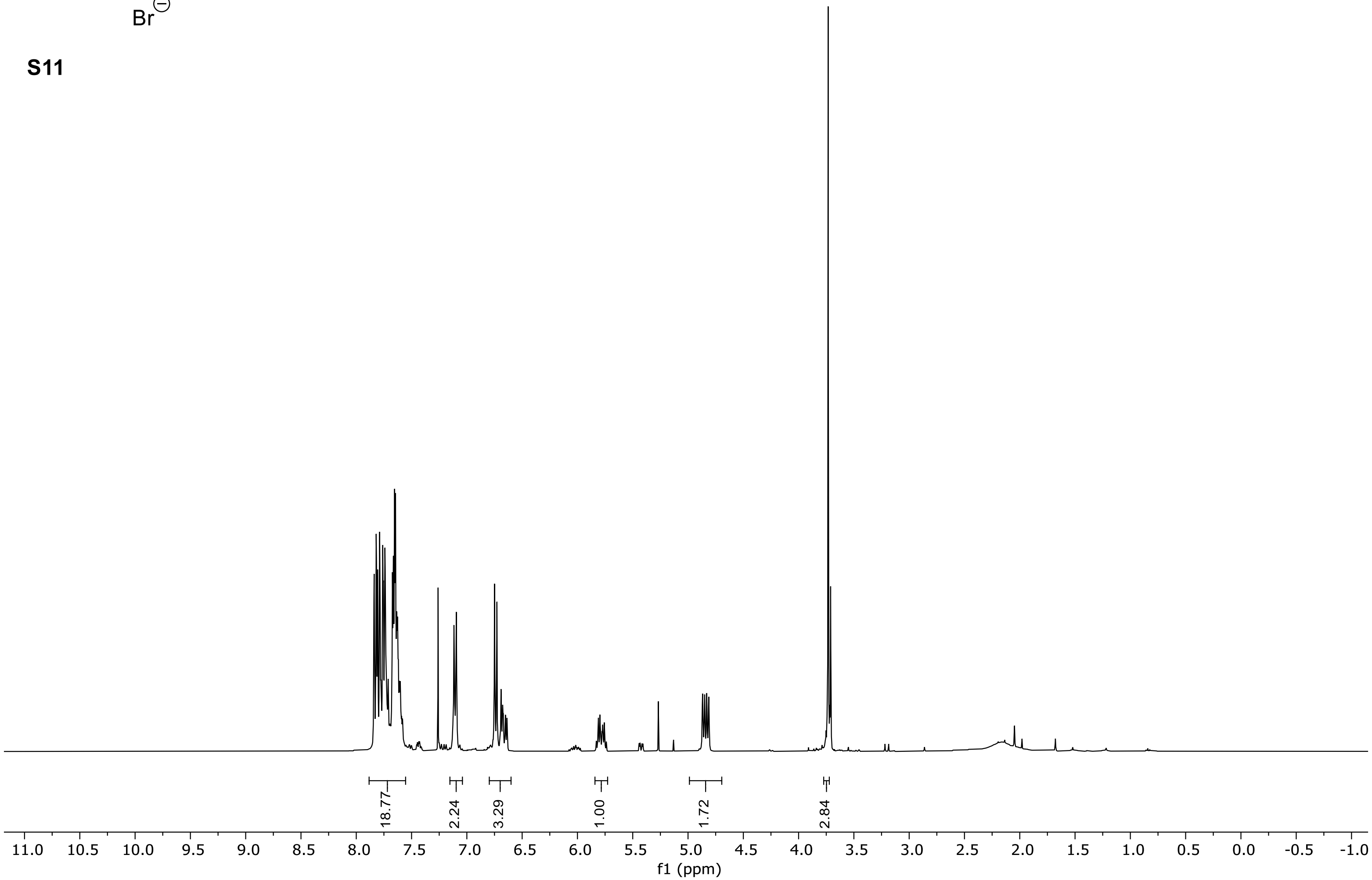

^{13}C NMR (101 MHz, $CDCl_3$)

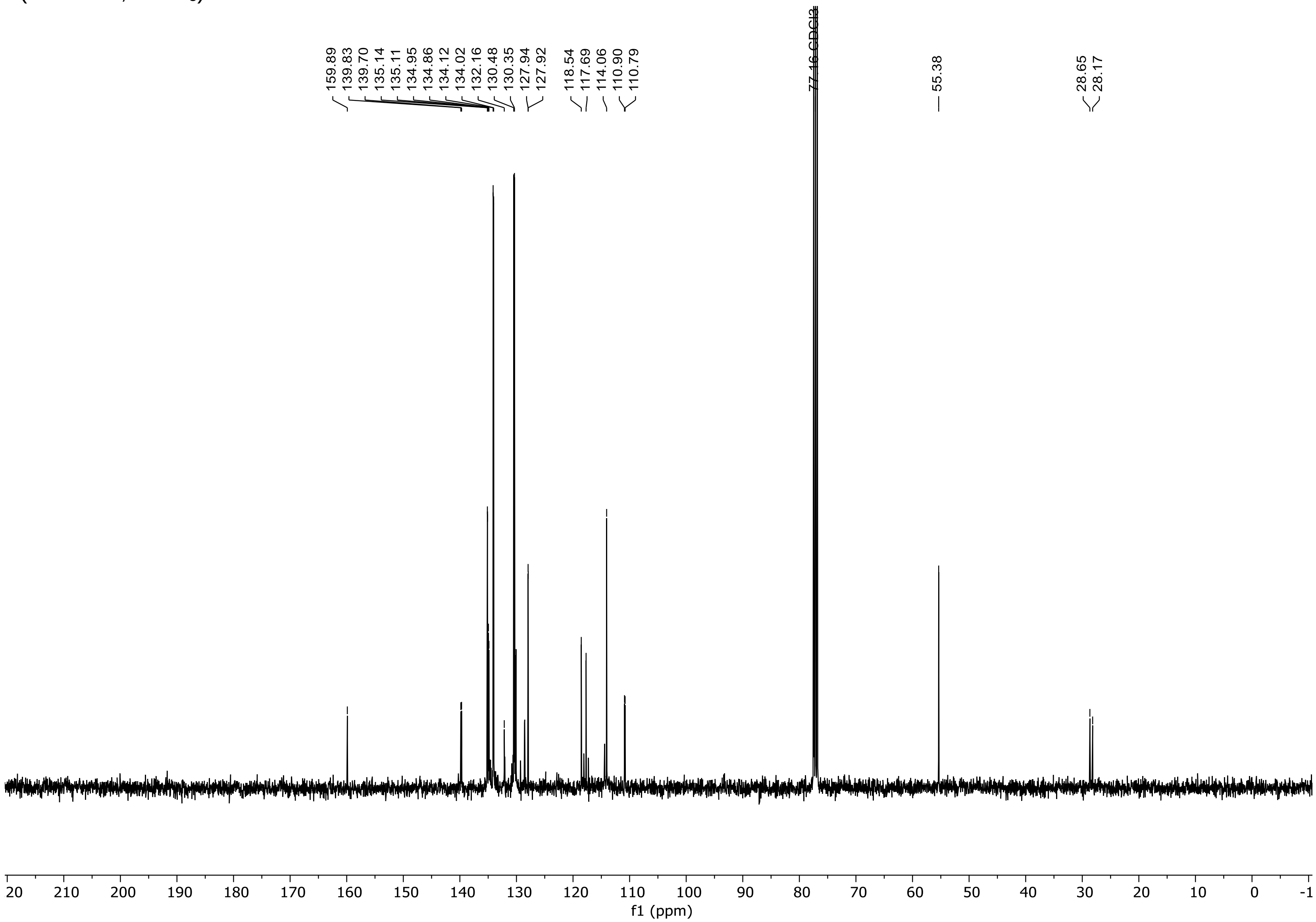

^{31}P (162 MHz, $CDCl_3$):

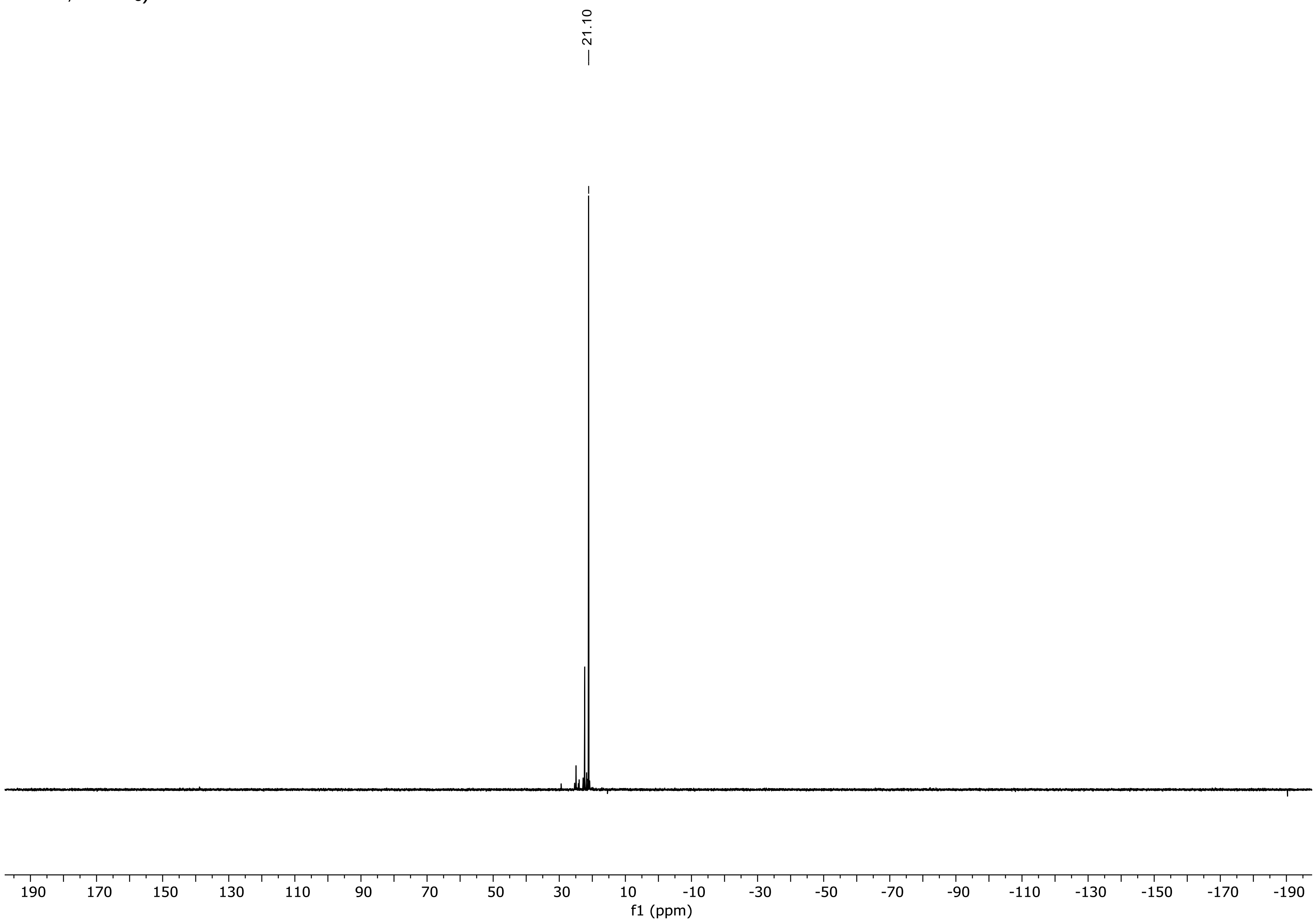

3-(4-Chlorophenyl)cyclopent-2-en-1-one S14.

^{1}H (400 MHz, $CDCl_3$)

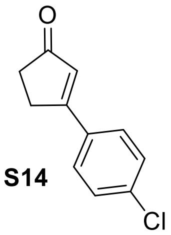

S14

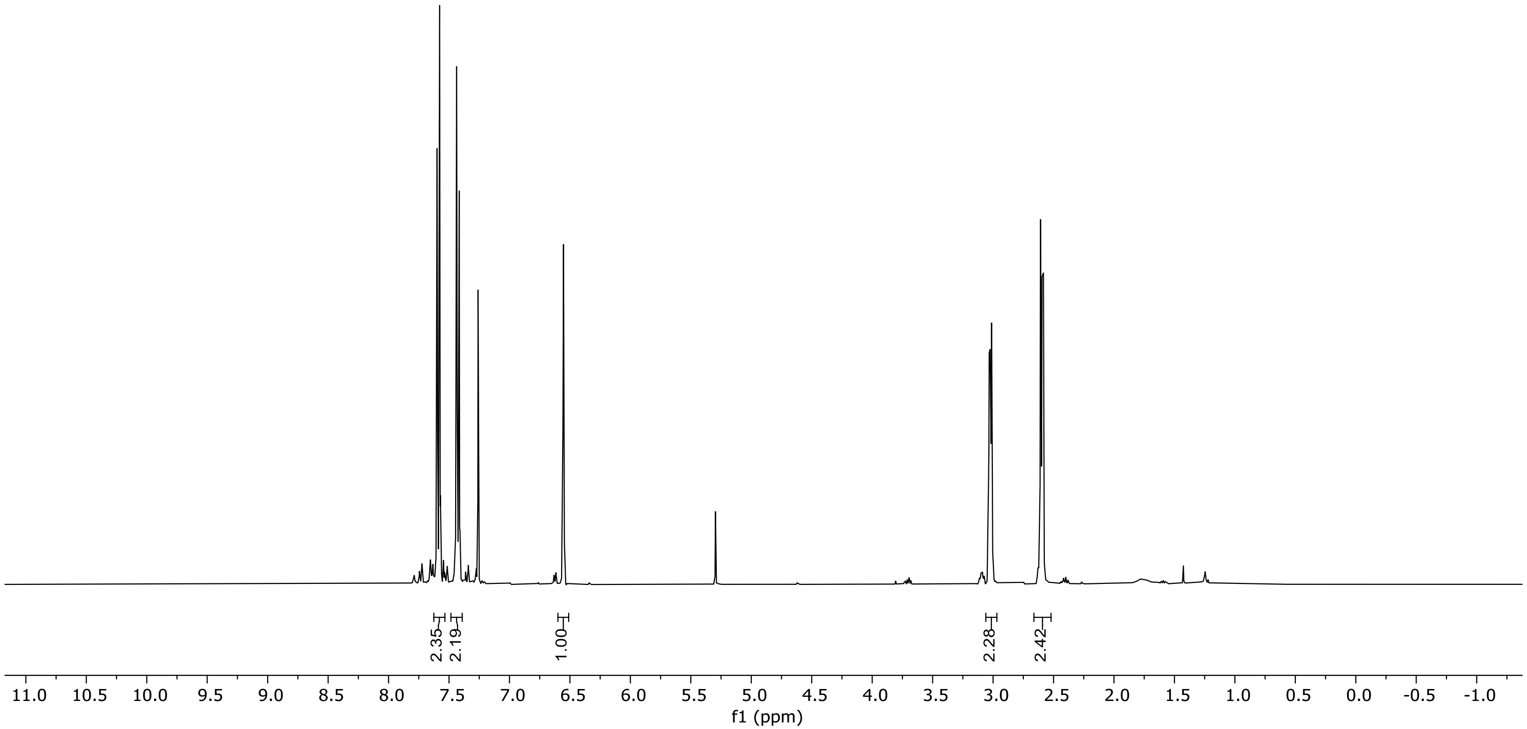

^{13}C NMR (101 MHz, $CDCl_3$)

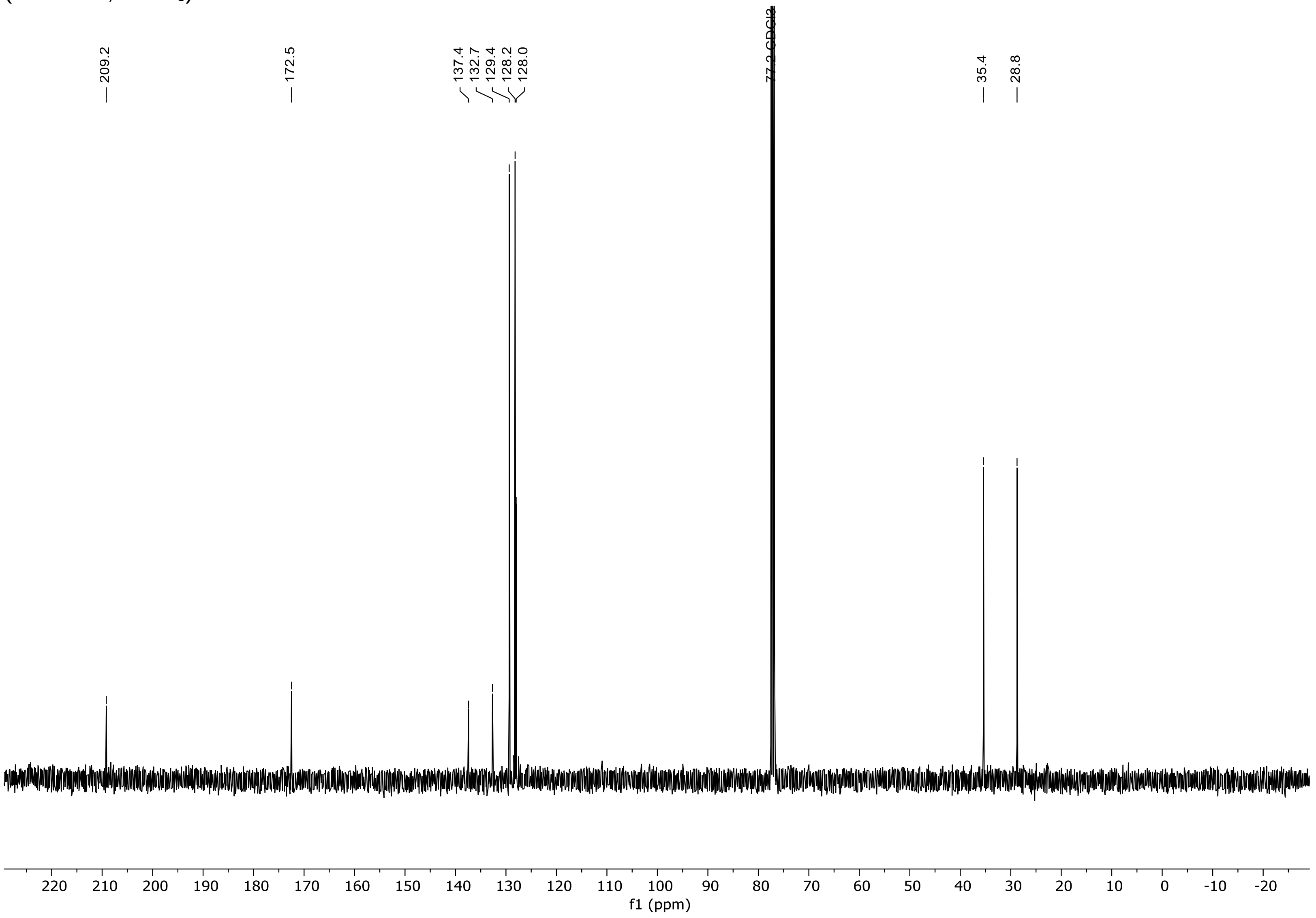

3-(4-Chlorophenyl)cyclopent-2-en-1-ol S15.

^{1}H (400 MHz, CD_2Cl_2)

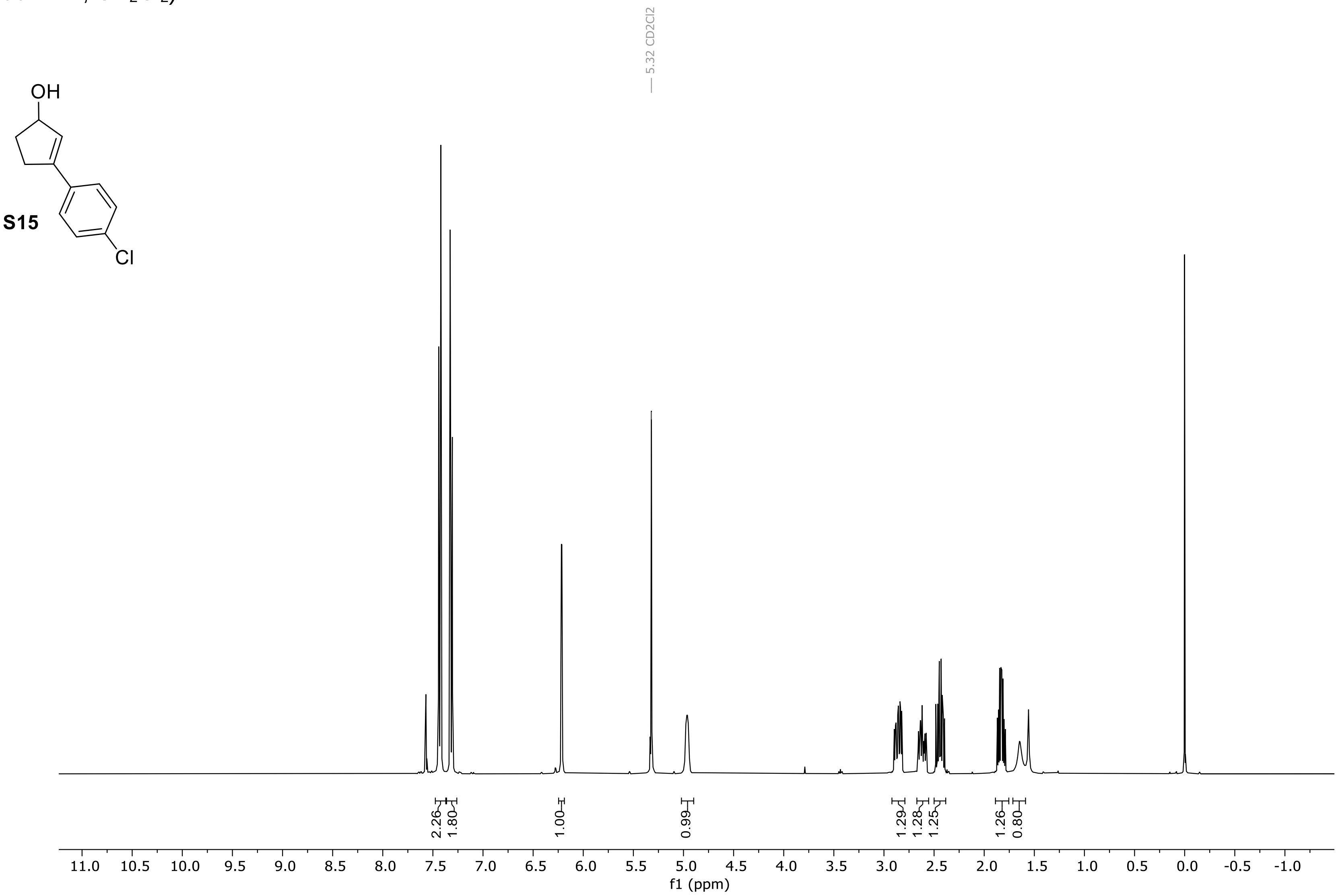

^{13}C NMR (101 MHz, CD_2Cl_2)

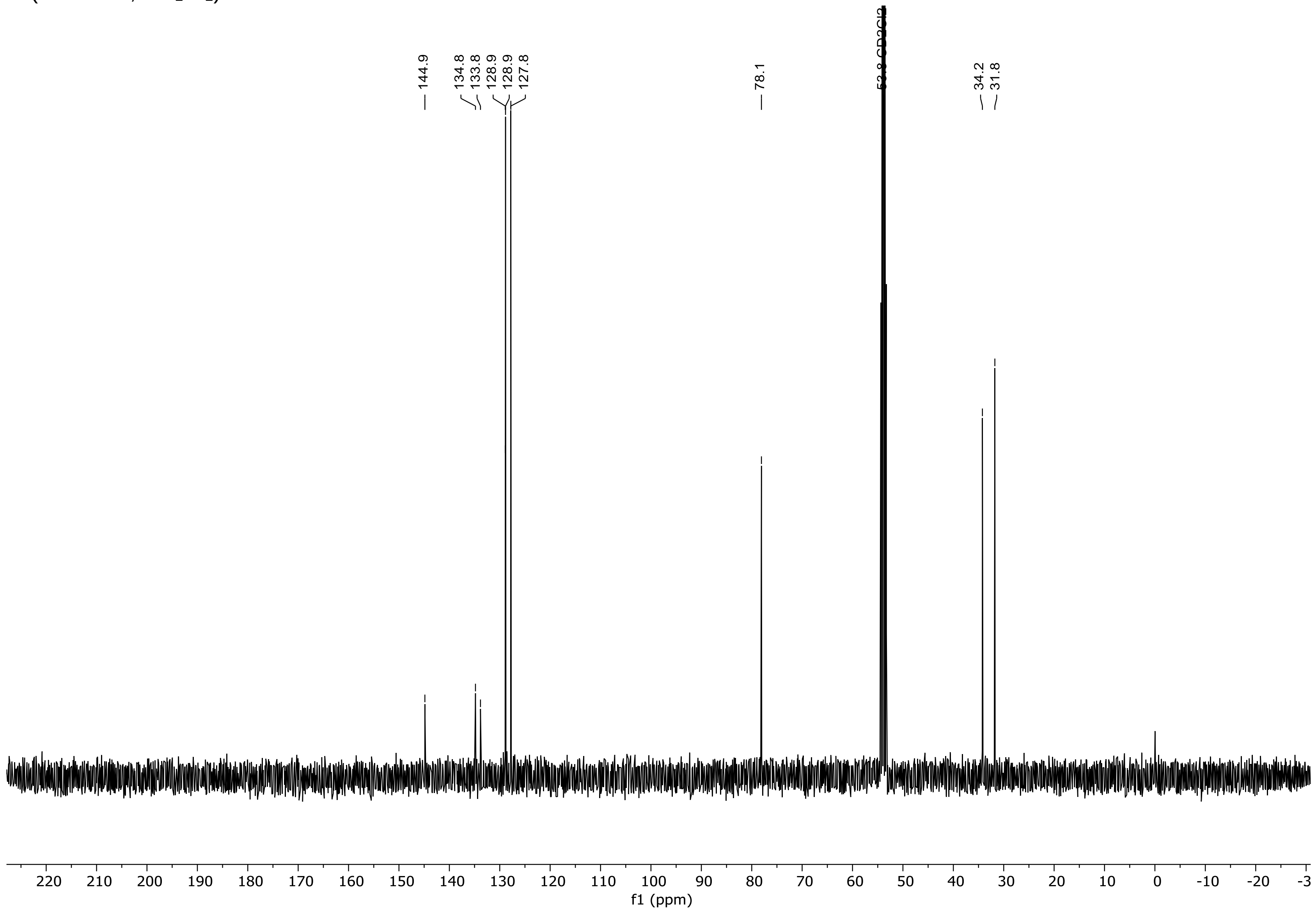

1-Methoxy-4-((1*E*,5*E*)-5-methyl-8-phenylocta-1,5-dien-1-yl)benzene 1.

^{1}H NMR (400 MHz, $CDCl_3$)

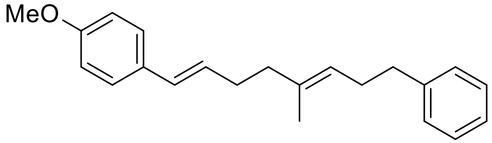

1

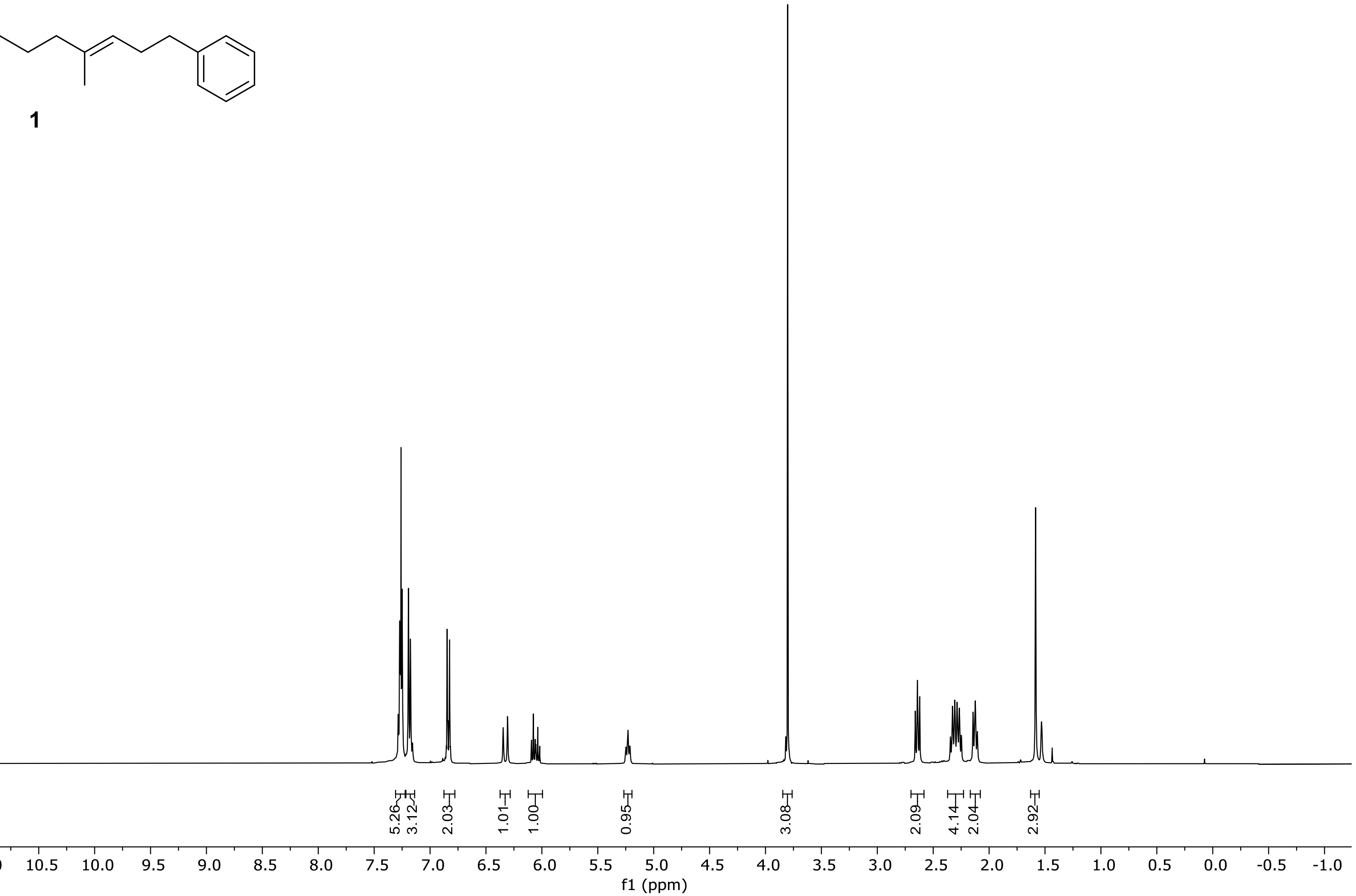

^{13}C NMR (101 MHz, $CDCl_3$)

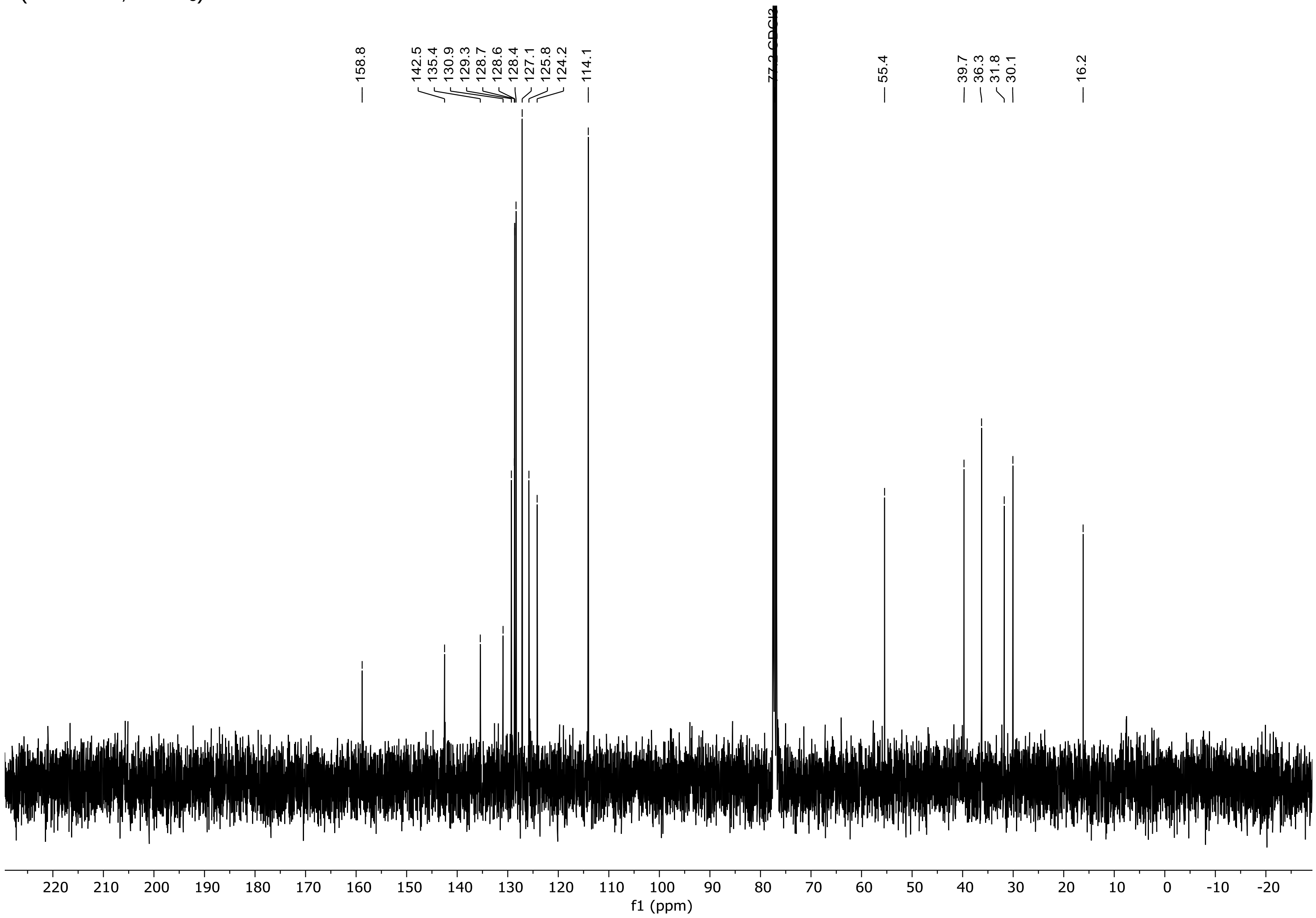

1-Methoxy-4-((1Z,5E)-5-methyl-8-phenylocta-1,5-dien-1-yl)benzene *Z*-1.

^{1}H NMR (400 MHz, $CDCl_3$)

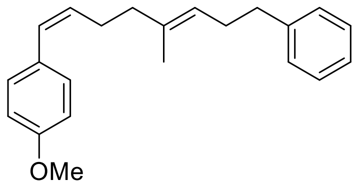

(*Z*)-1

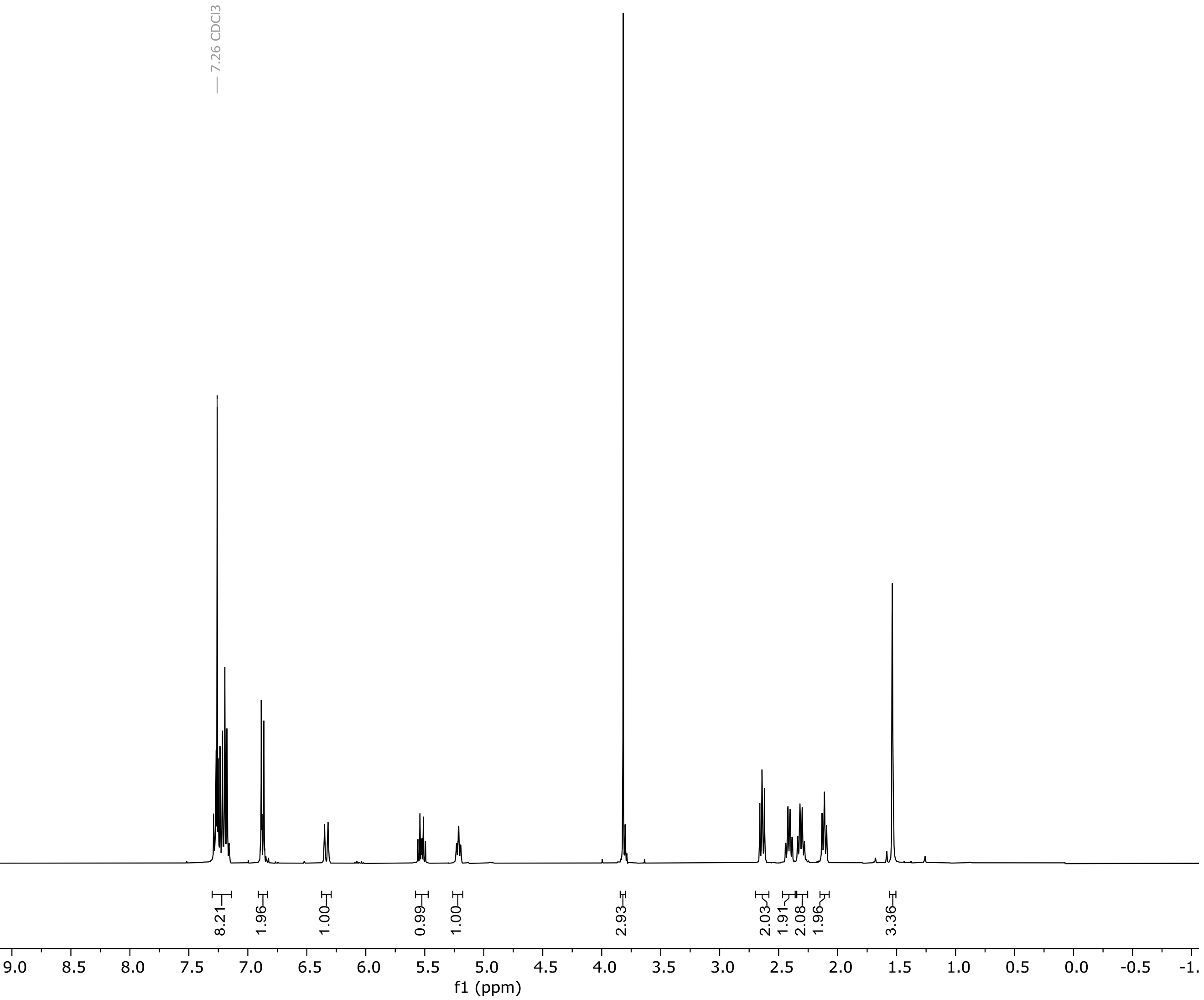

^{13}C NMR (151 MHz, $CDCl_3$)

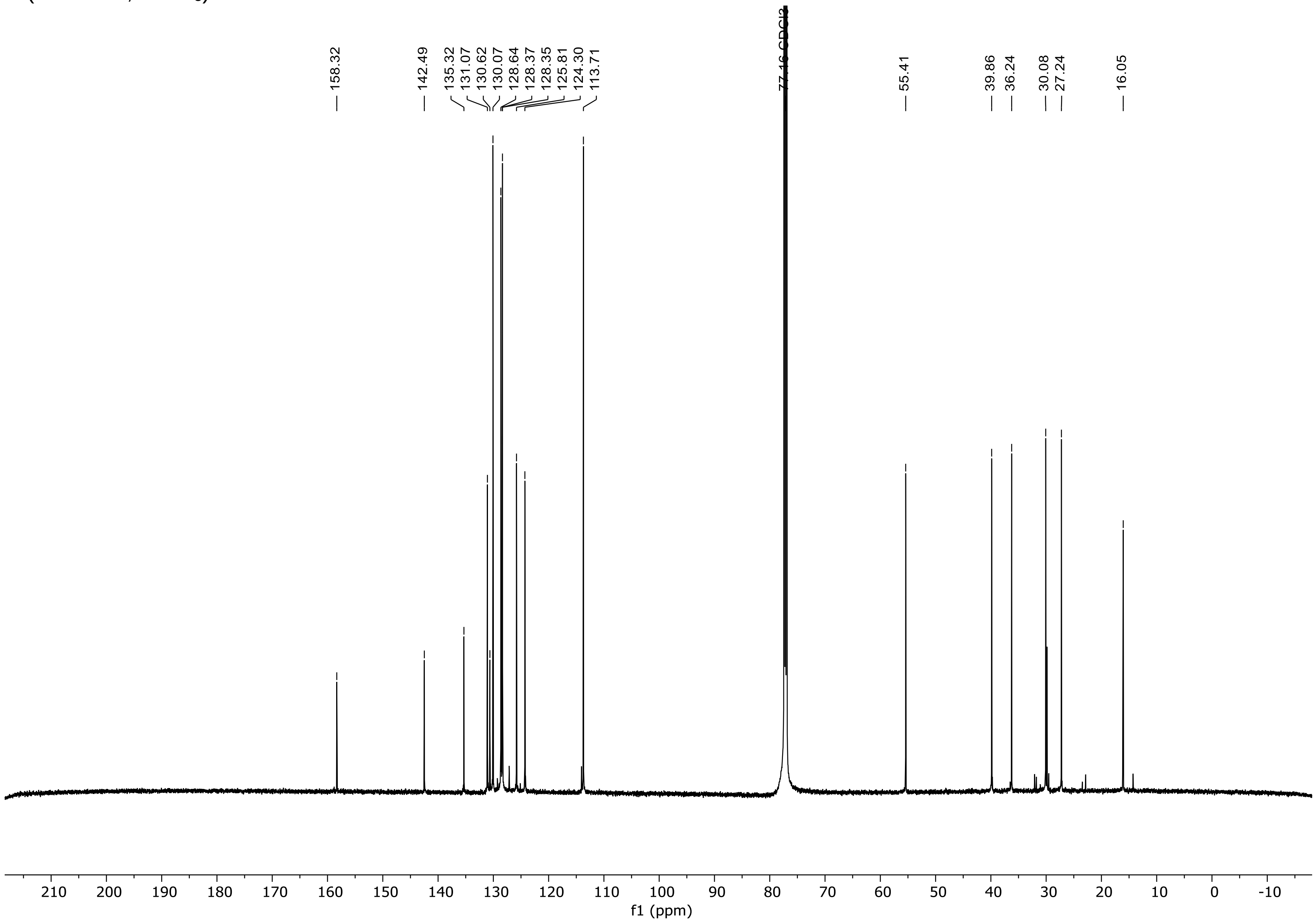

1-Methoxy-4-((1*E*,5E)-8-phenylocta-1,5-dien-1-yl)benzene 3.

^{1}H NMR (400 MHz, $CDCl_3$)

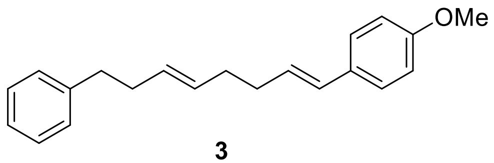

3

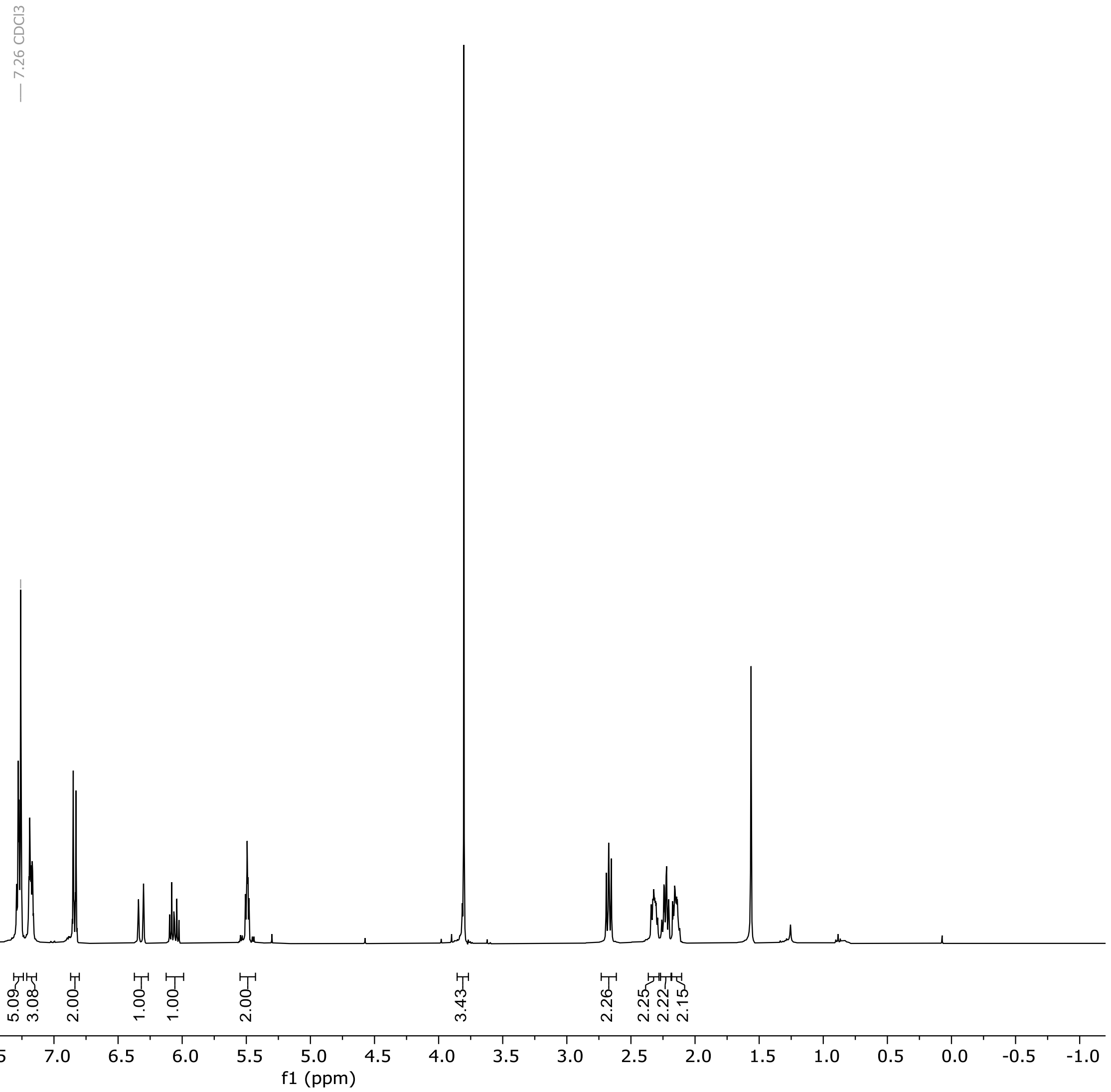

^{13}C NMR (101 MHz, $CDCl_3$)

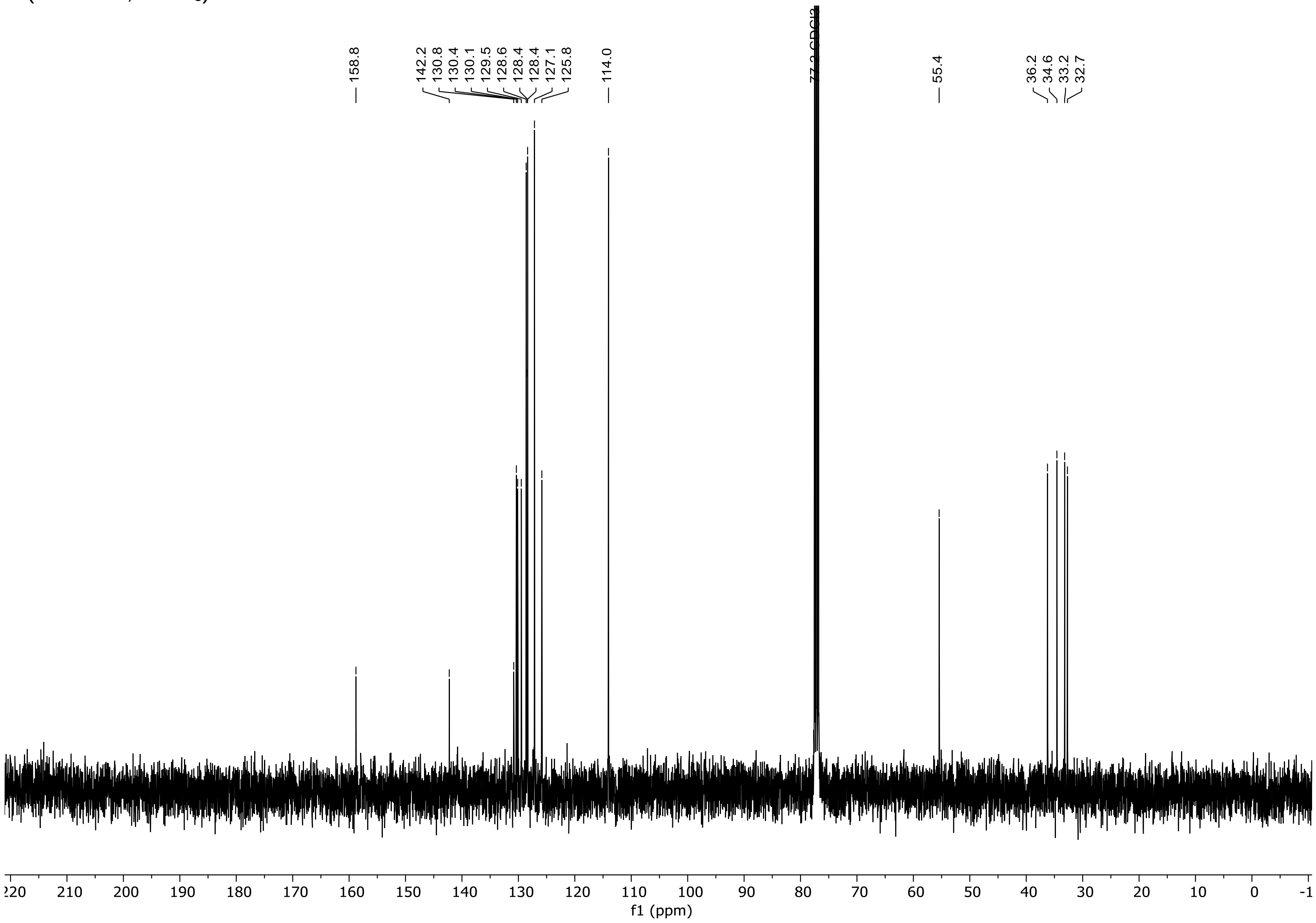

1-Methoxy-4-((1*E*,3*E*,7*E*)-7-methyl-10-phenyldeca-1,3,7-trien-1-yl)benzene (*E*)-4 and 1-methoxy-4-((1E,3*Z*,7*E*)-7-methyl-10-phenyldeca-1,3,7-trien-1-yl)benzene (*Z*)-4.

^{1}H NMR (400 MHz, $CDCl_3$)

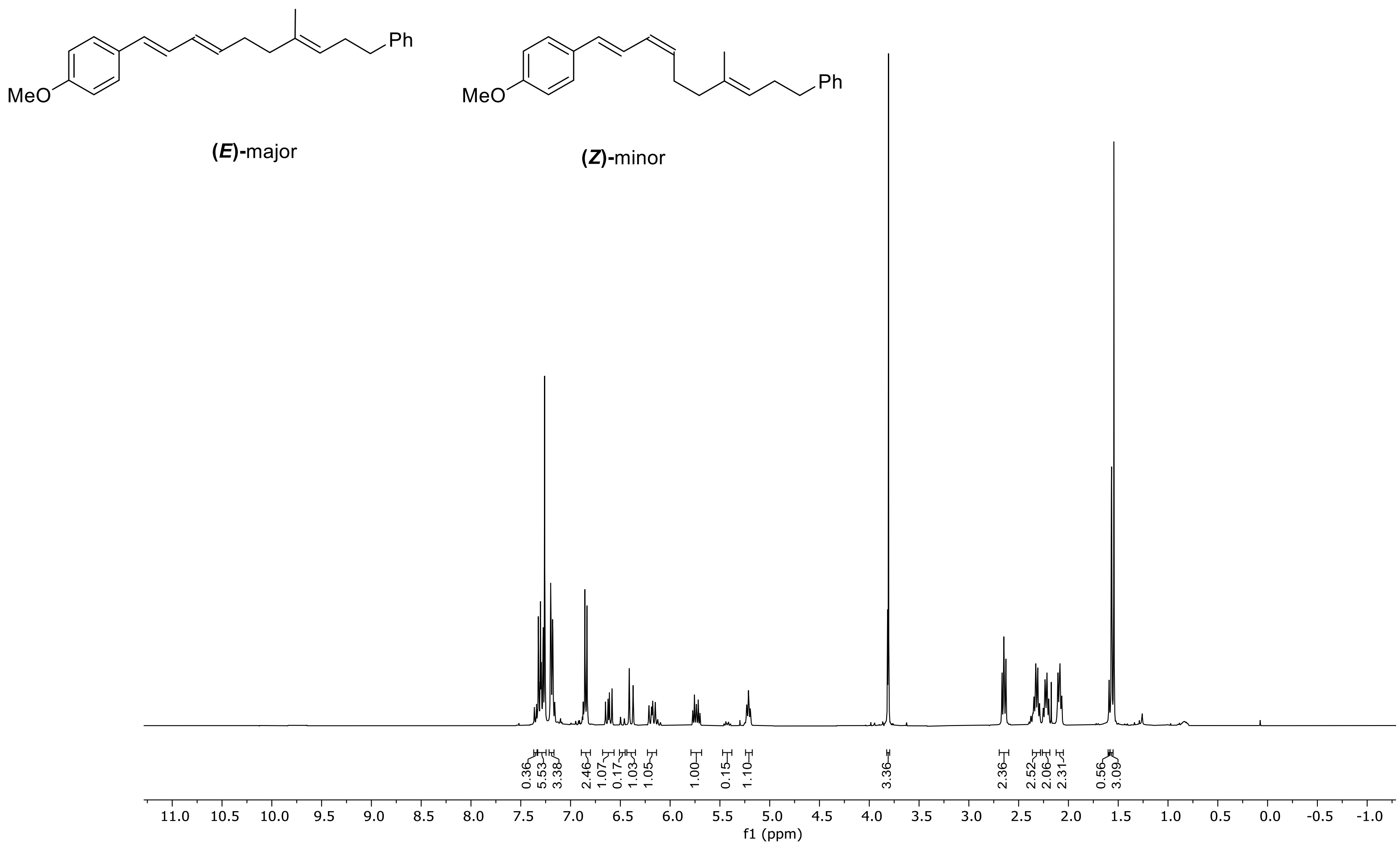

^{13}C NMR (101 MHz, $CDCl_3$)

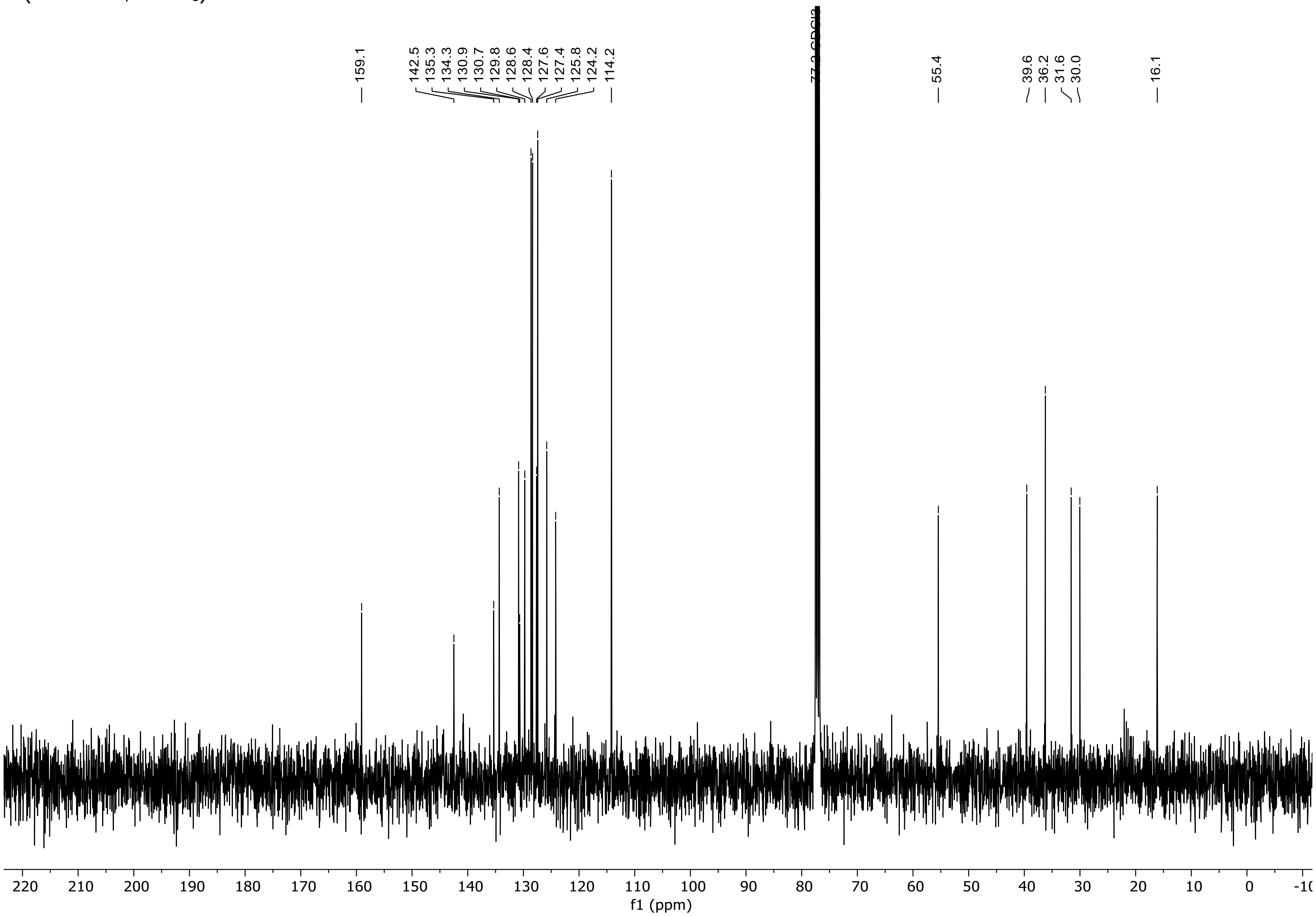

1-Methoxy-4-((1*E*,3*E*,7*E*)-10-phenyldeca-1,3,7-trien-1-yl)benzene (*E*)-5 and 1-Methoxy-4-((1*E*,3*Z*,7*E*)-10-phenyldeca-1,3,7-trien-1-yl)benzene (*Z*)-5.

^{1}H NMR (400 MHz, $CDCl_3$)

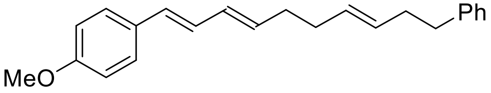

(*E*)-major

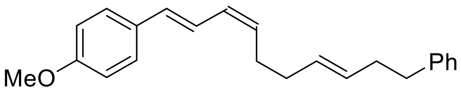

(*Z*)-minor

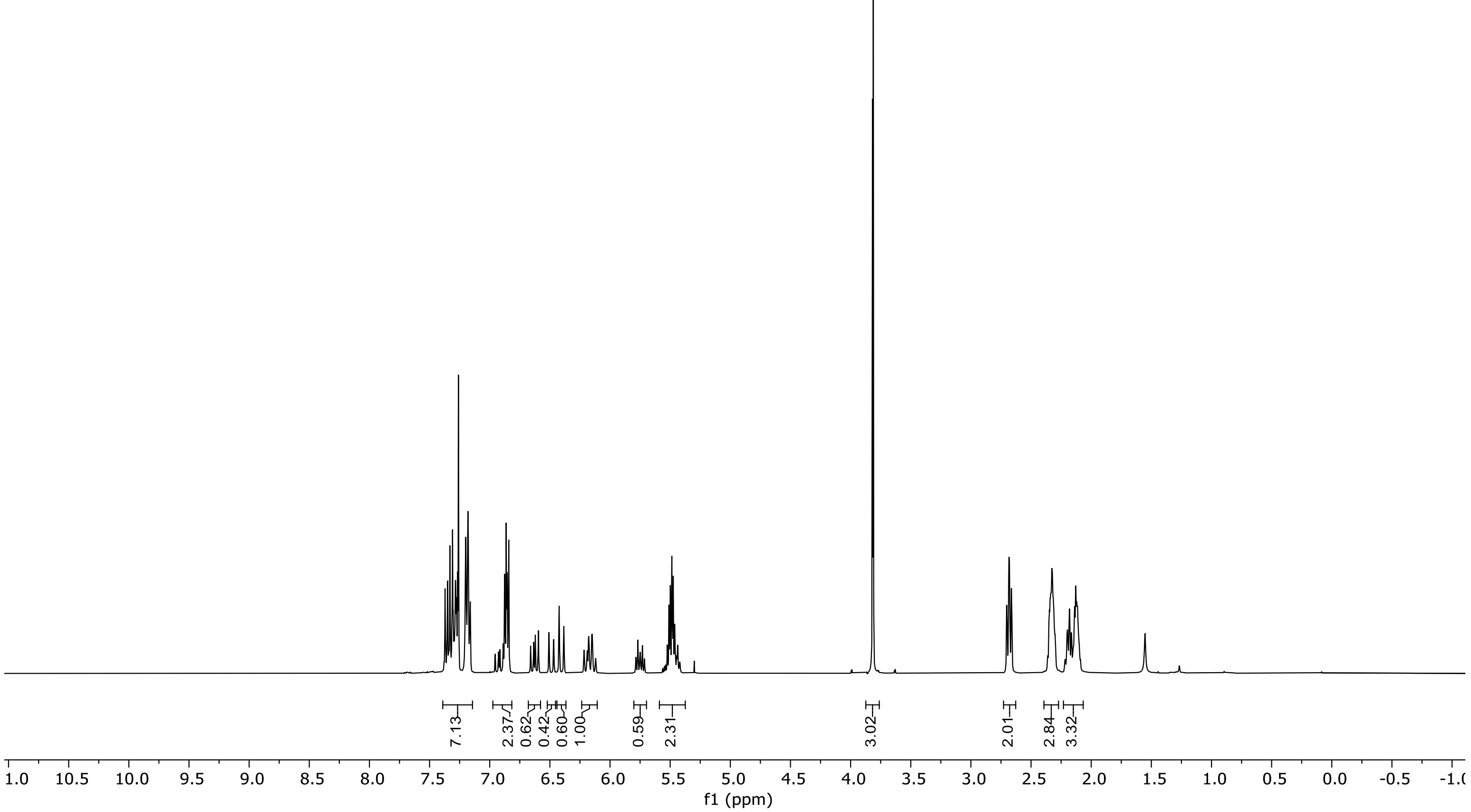

^{13}C NMR (101 MHz, $CDCl_3$)

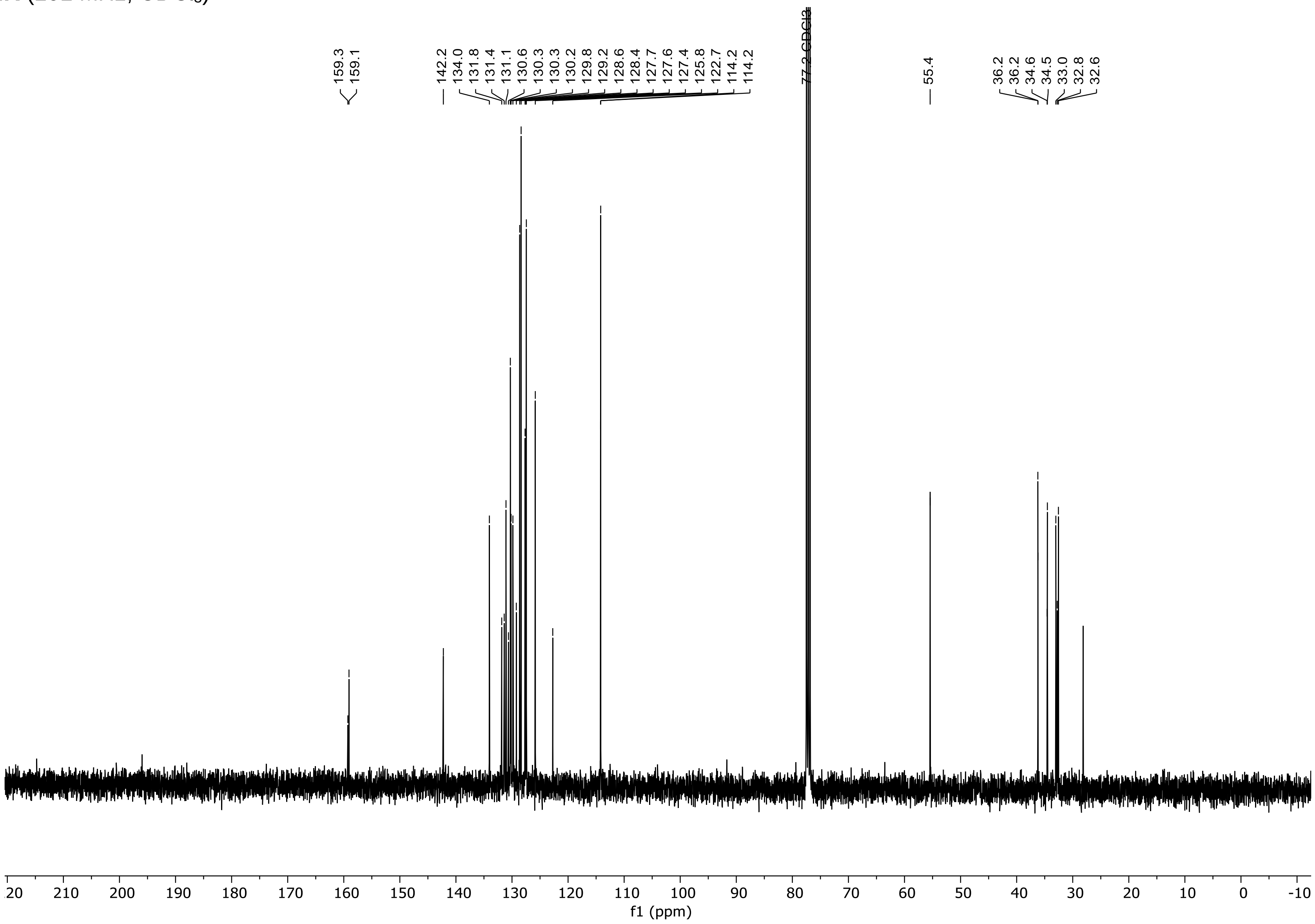

^{1}**H** (400 MHz)-^{13}**C** (101 MHz) **HSQC-2D** ($CDCl_3$)

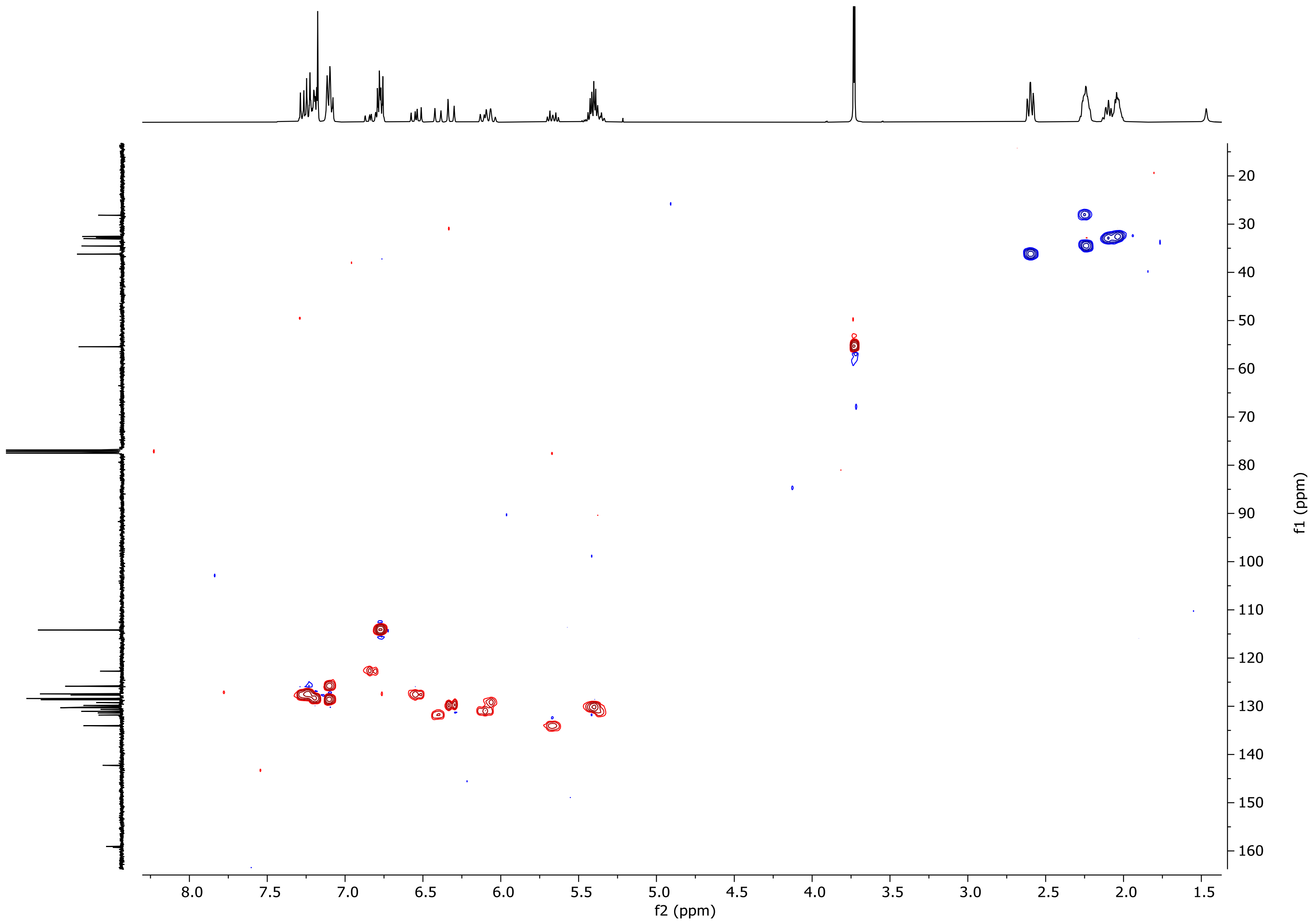

4,4'-((1*E*,5*E*)-5-methylocta-1,5-diene-1,8-diyl)bis(methoxybenzene) 7.

^{1}H NMR (400 MHz, $CDCl_3$)

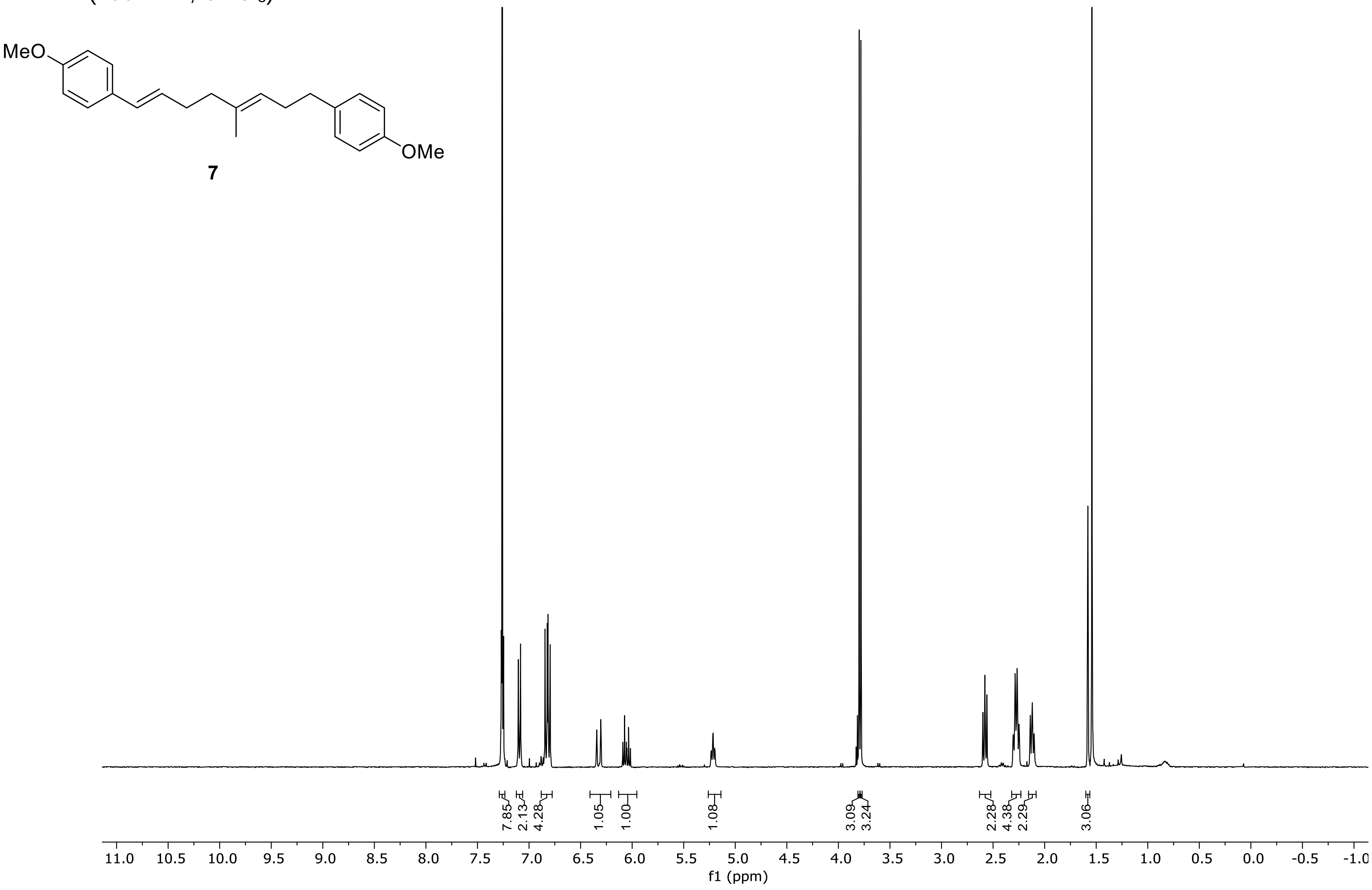

^{13}C NMR (151 MHz, $CDCl_3$)

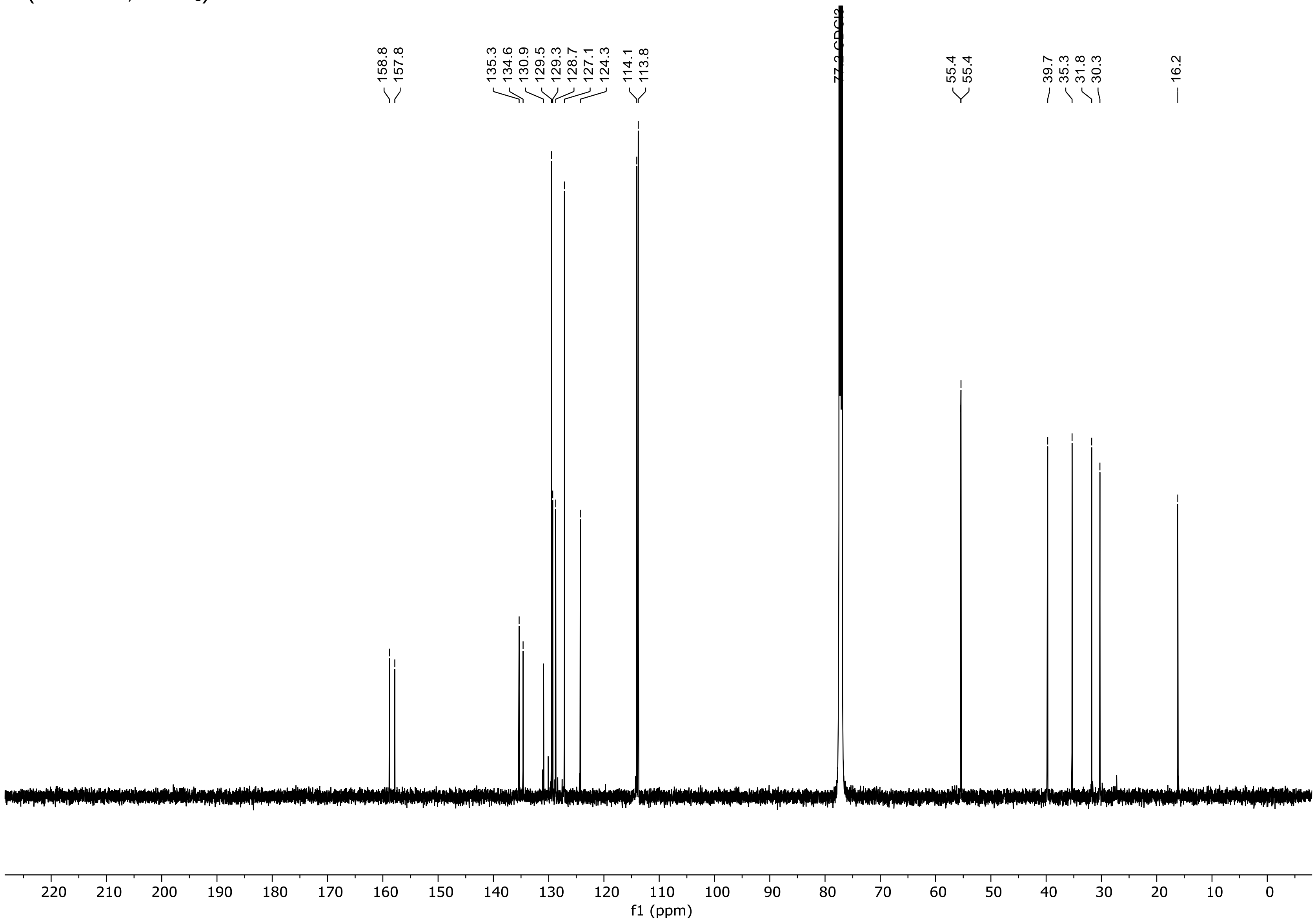

1-Methoxy-4-((1E,5E)-5-methyl-8-(p-tolyl)octa-1,5-dien-1-yl)benzene 8.

^{1}H NMR (400 MHz, $CDCl_3$)

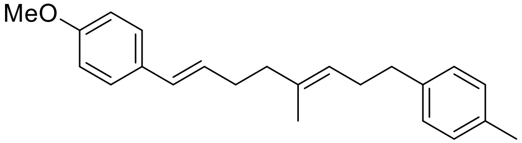

8

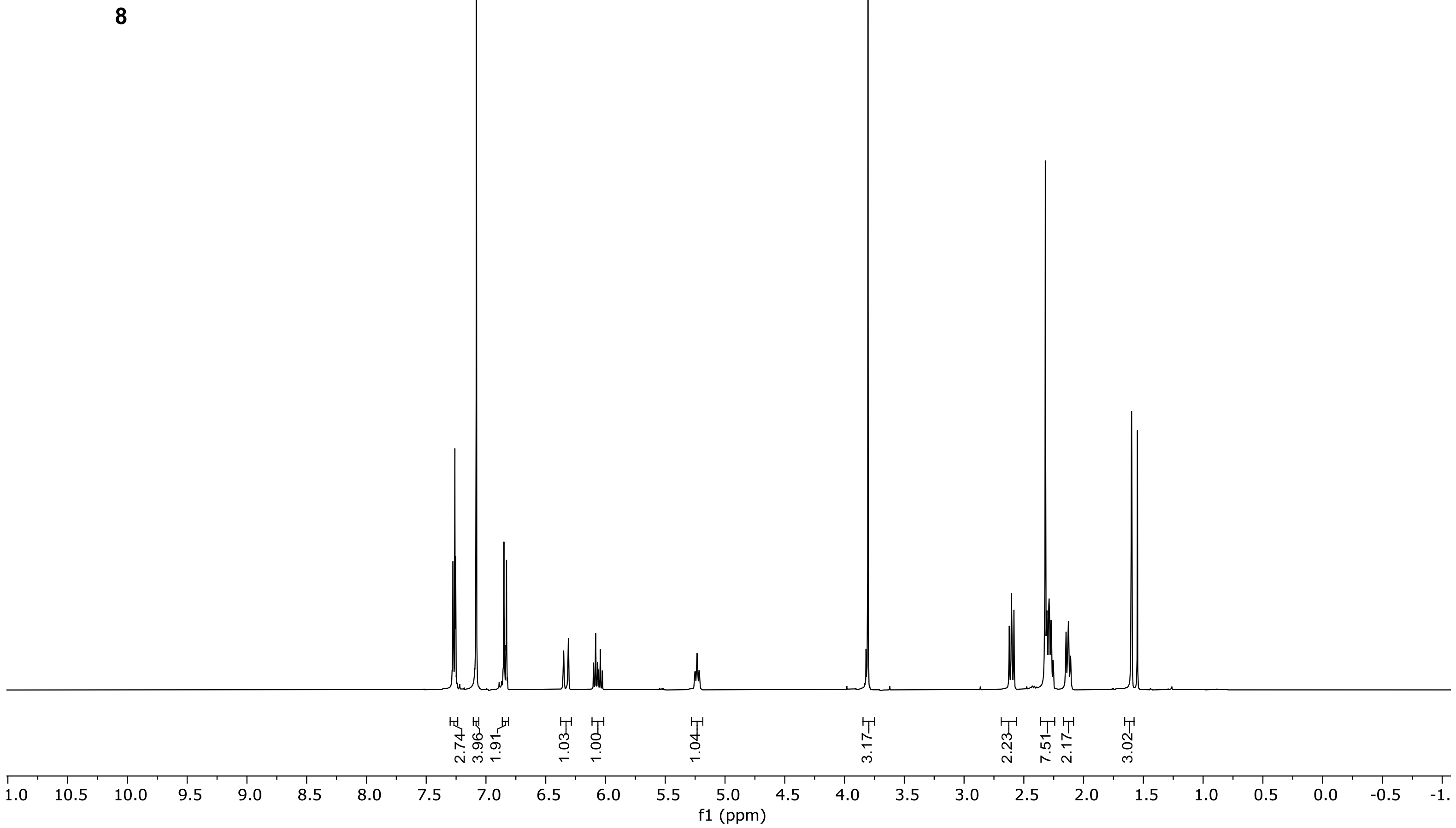

^{13}C NMR (101 MHz, $CDCl_3$)

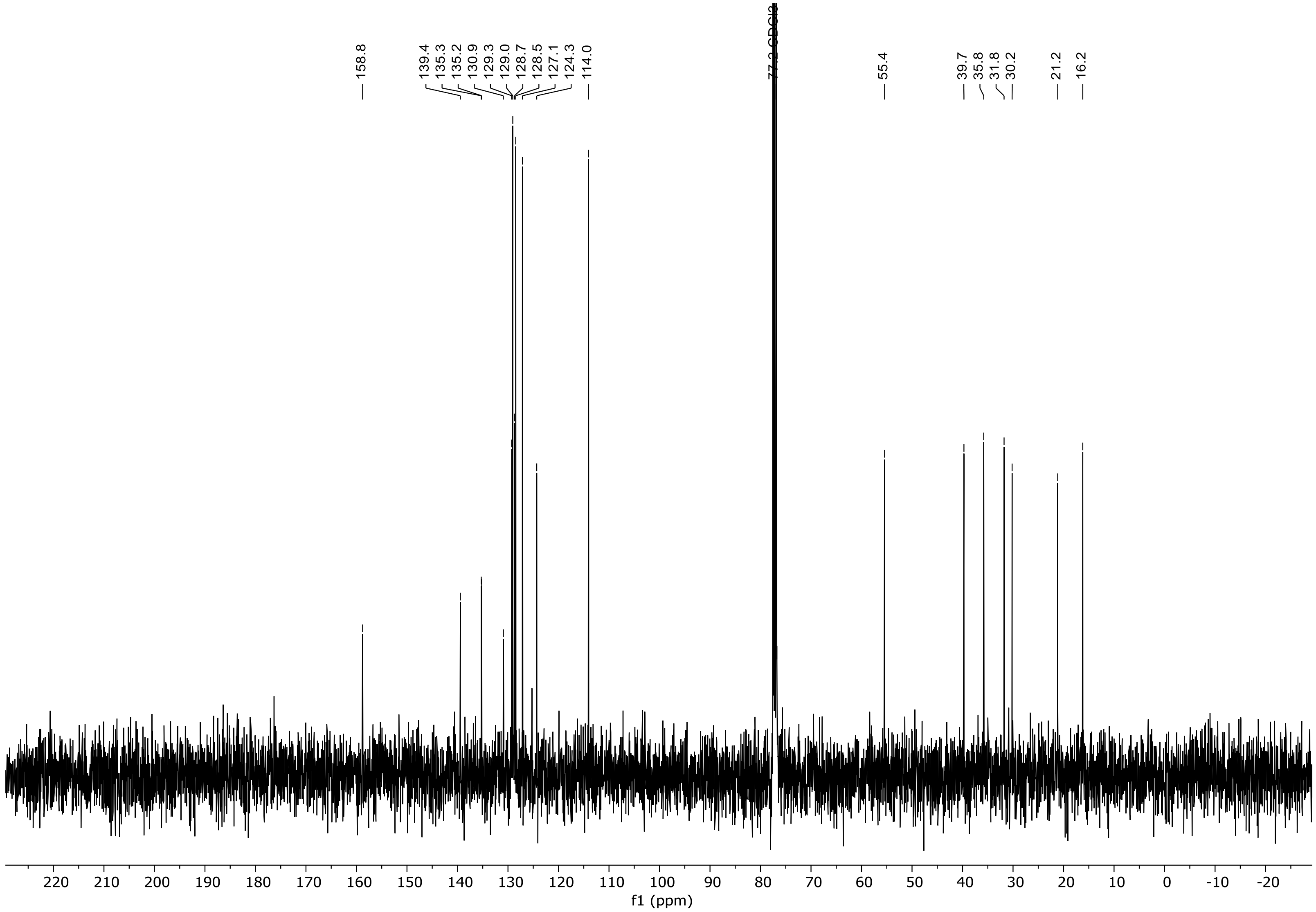

1,4-Dimethoxy-2-((3*E*,7*E*)-8-(4-methoxyphenyl)-4-methylocta-3,7-dien-1-yl)benzene 9.

^{1}H NMR (400 MHz, $CDCl_3$)

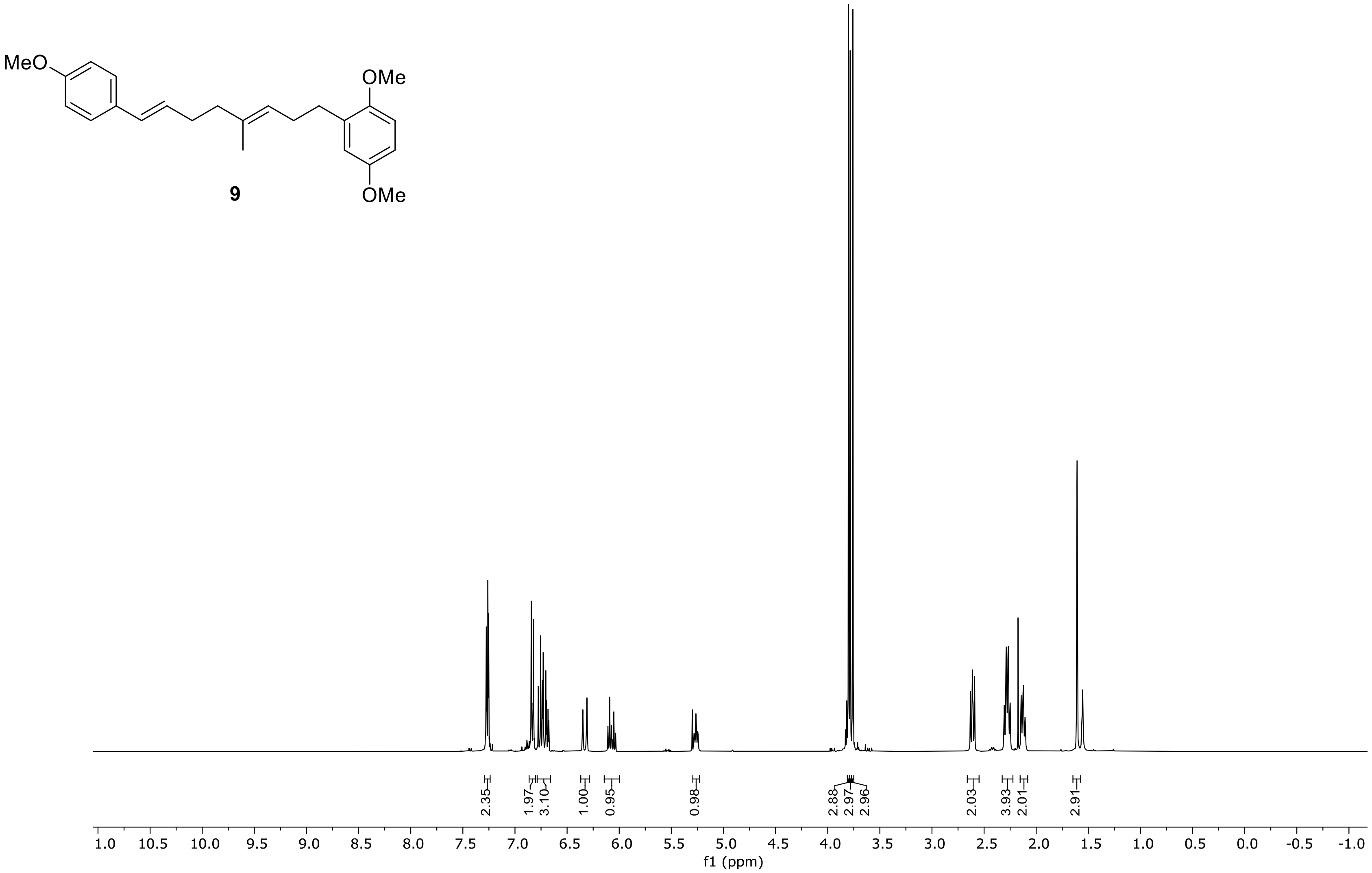

^{13}C NMR (101 MHz, $CDCl_3$)

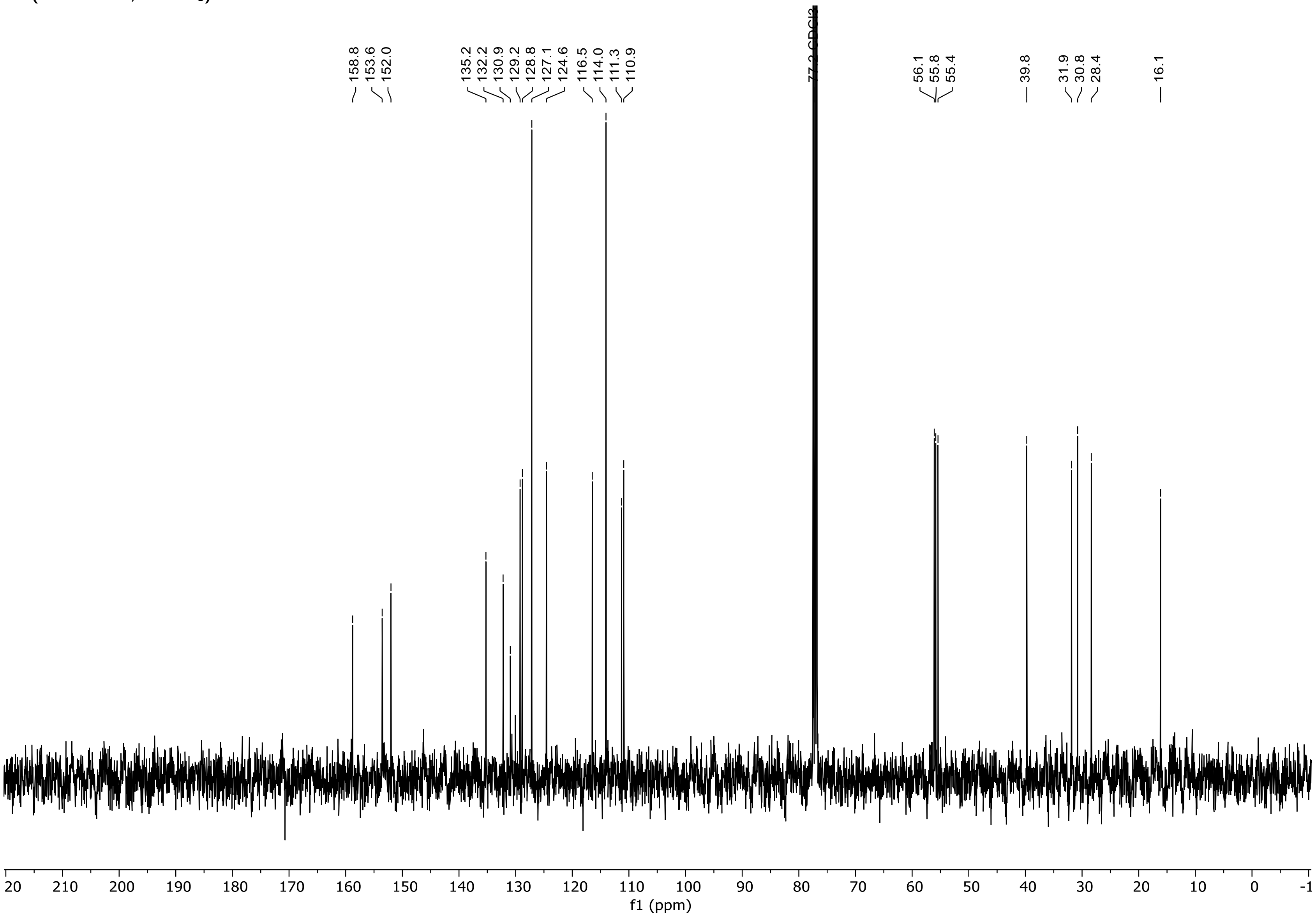

1-Chloro-4-((3*E*,7*E*)-8-(4-methoxyphenyl)-4-methylocta-3,7-dien-1-yl)benzene 10.

^{1}H NMR (400 MHz, $CDCl_3$)

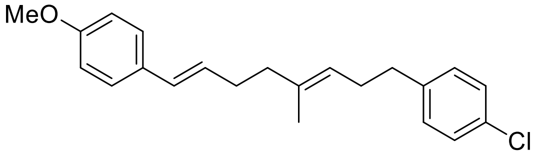

10

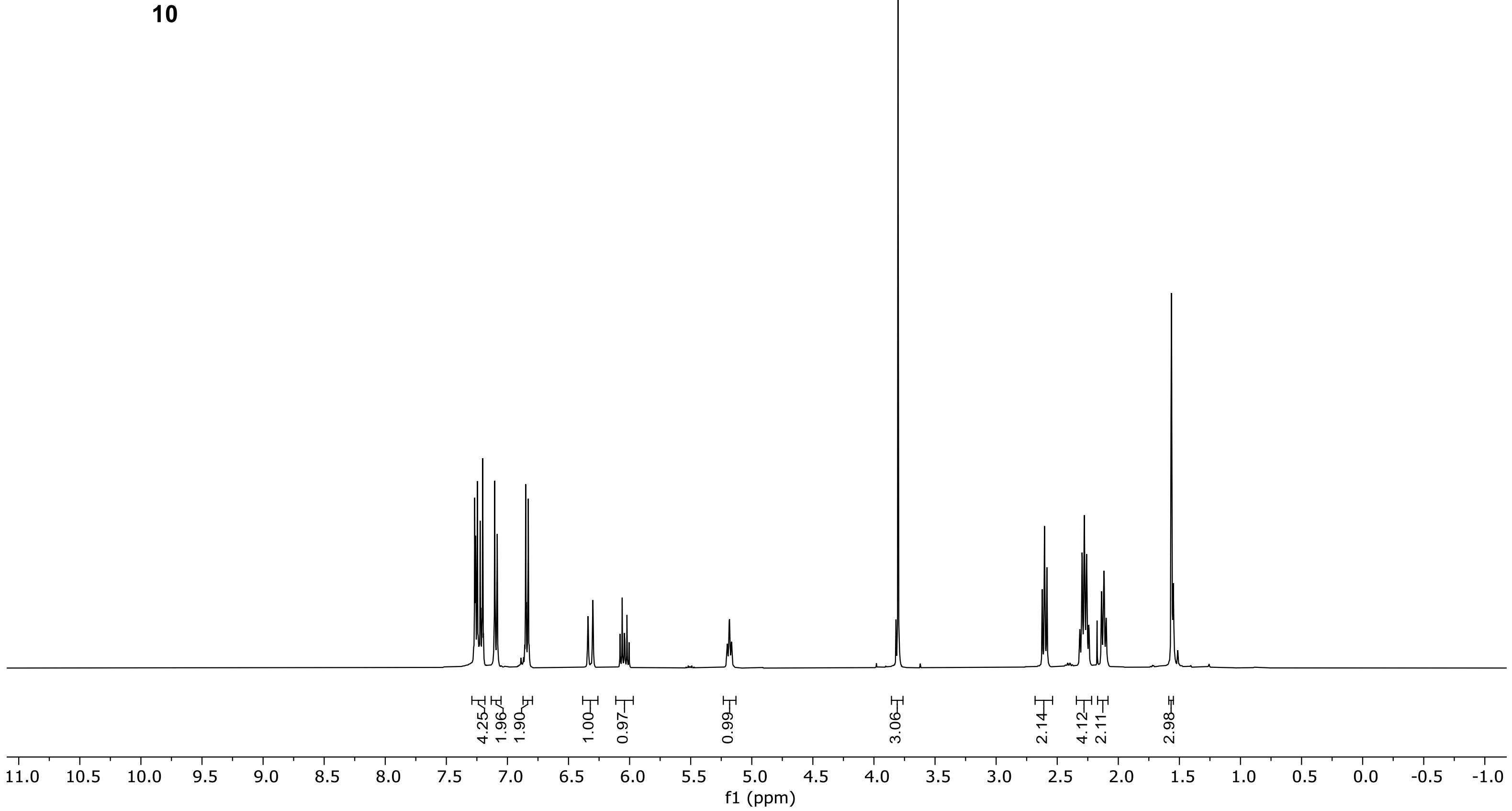

^{13}C NMR (101 MHz, $CDCl_3$)

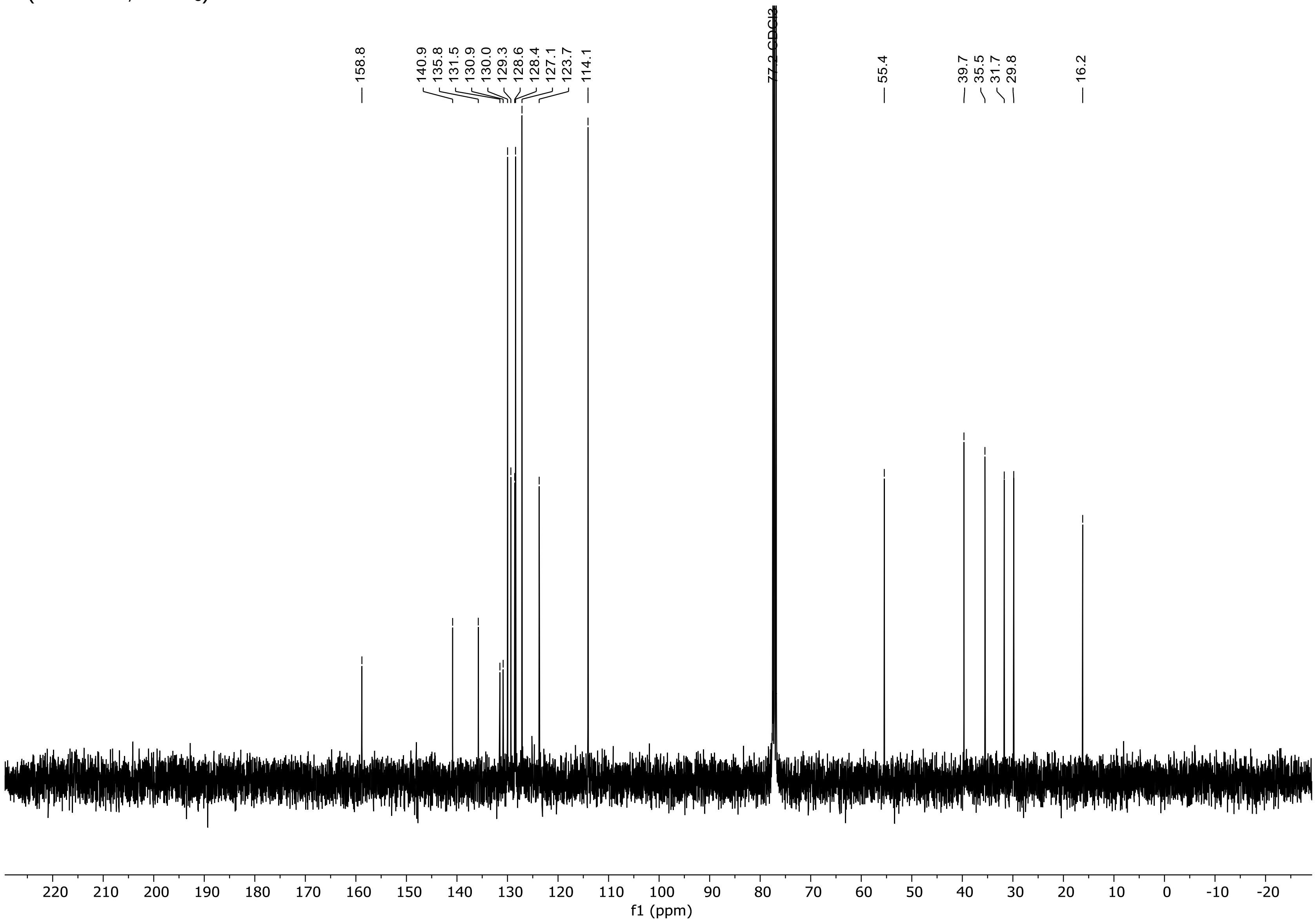

*7.2 Products **2a-2w**.*

(±)-(1*R*,2*R*,4a*S*,10a*S*)-2-(4-Methoxybenzyl)-1-(4-methoxyphenyl)-4*a*-methyl-1,2,3,4,4*a*,9,10,10*a*-octahydrophenanthrene 2a.

^{1}H NMR (400 MHz, $CDCl_3$)

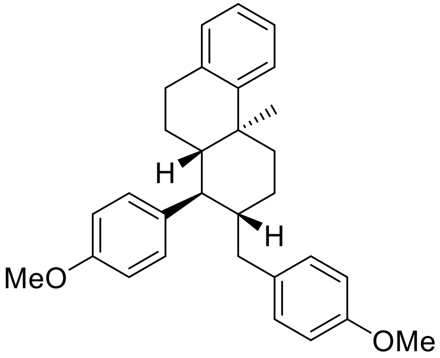

2a

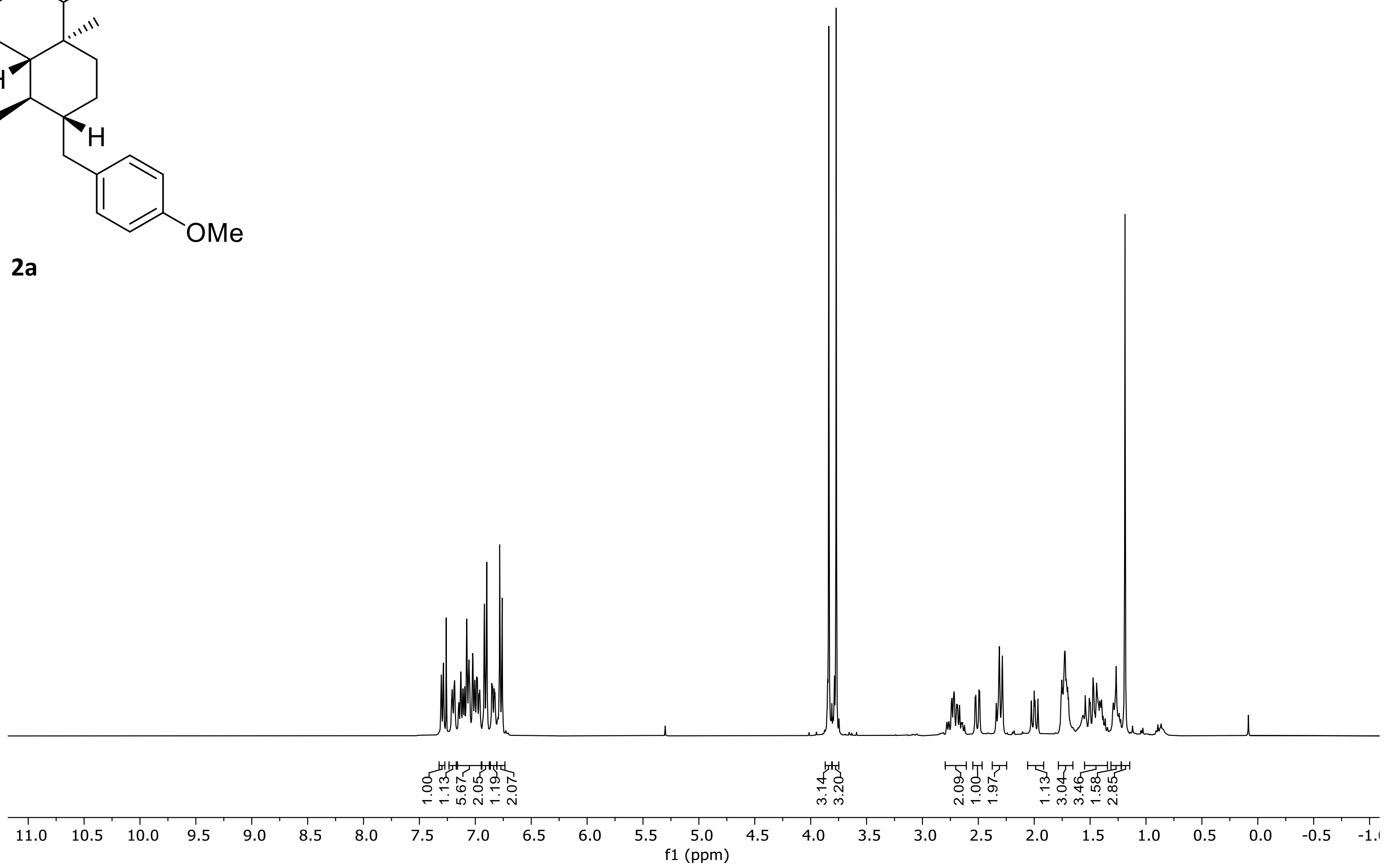

^{13}C NMR (101 MHz, $CDCl_3$)

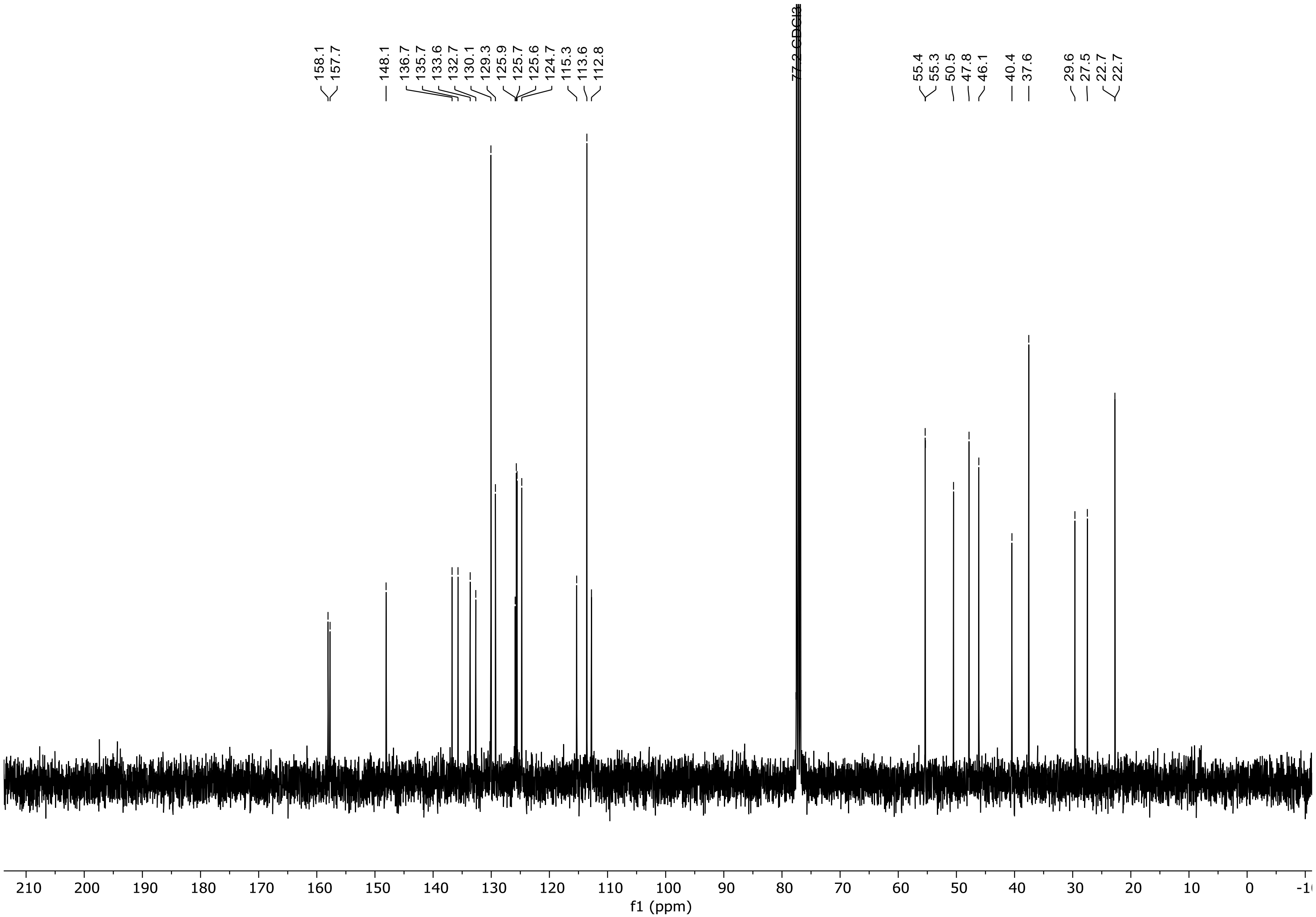

(±)-4-(((1*R*,2*R*,4*aS*,10*aS*)-1-(4-methoxyphenyl)-4*a*-methyl-1,2,3,4,4*a*,9,10,10*a*-octahydrophenanthren-2-yl)methyl)phenol 2b.

^{1}H NMR (400 MHz, $CDCl_3$)

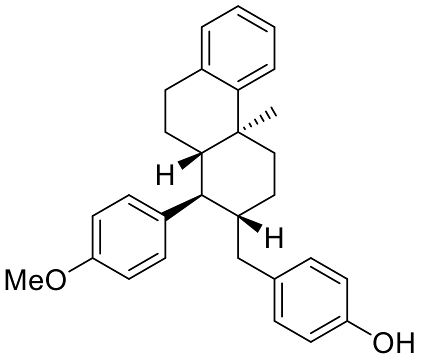

2b

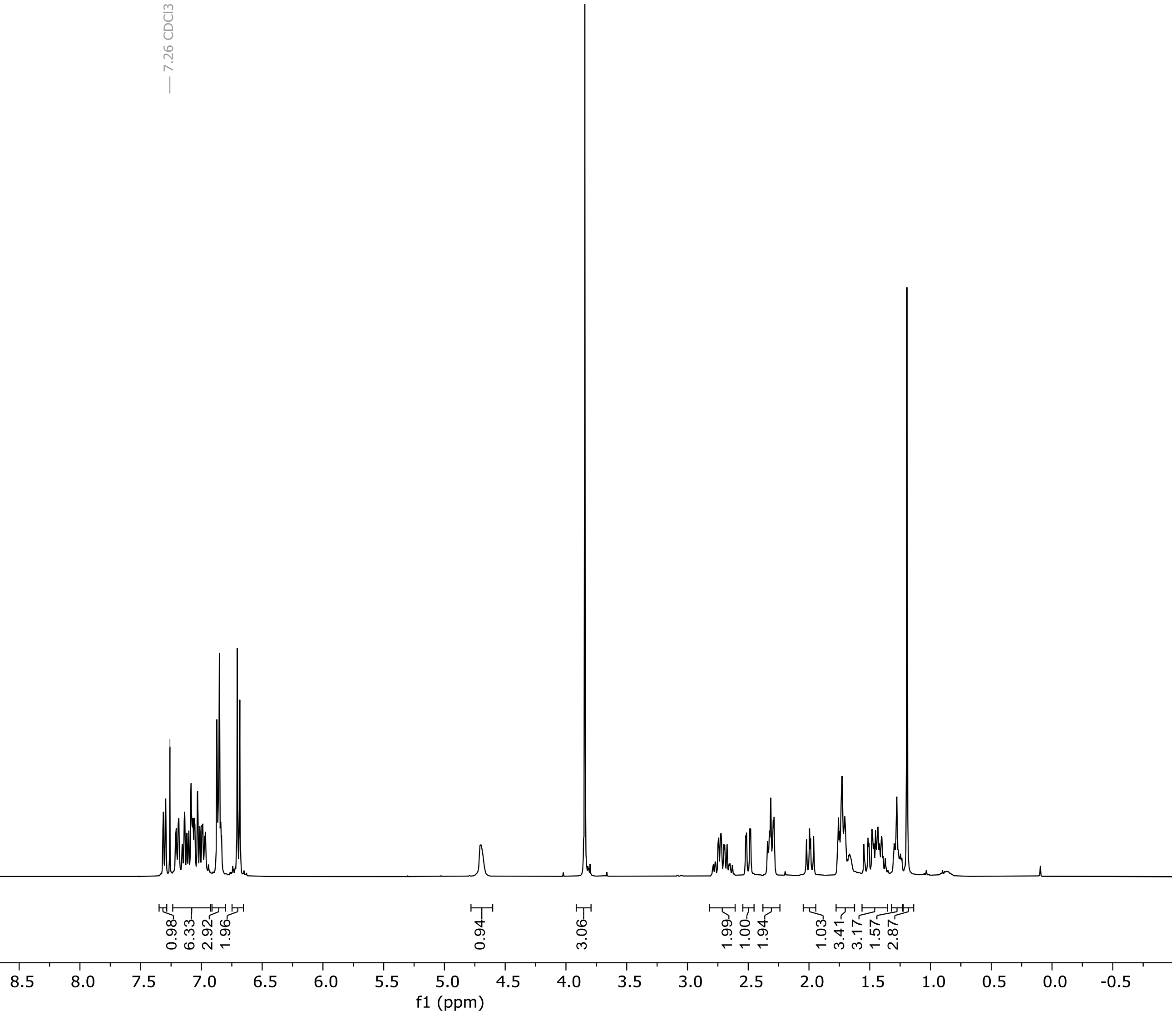

^{13}C NMR (101 MHz, $CDCl_3$)

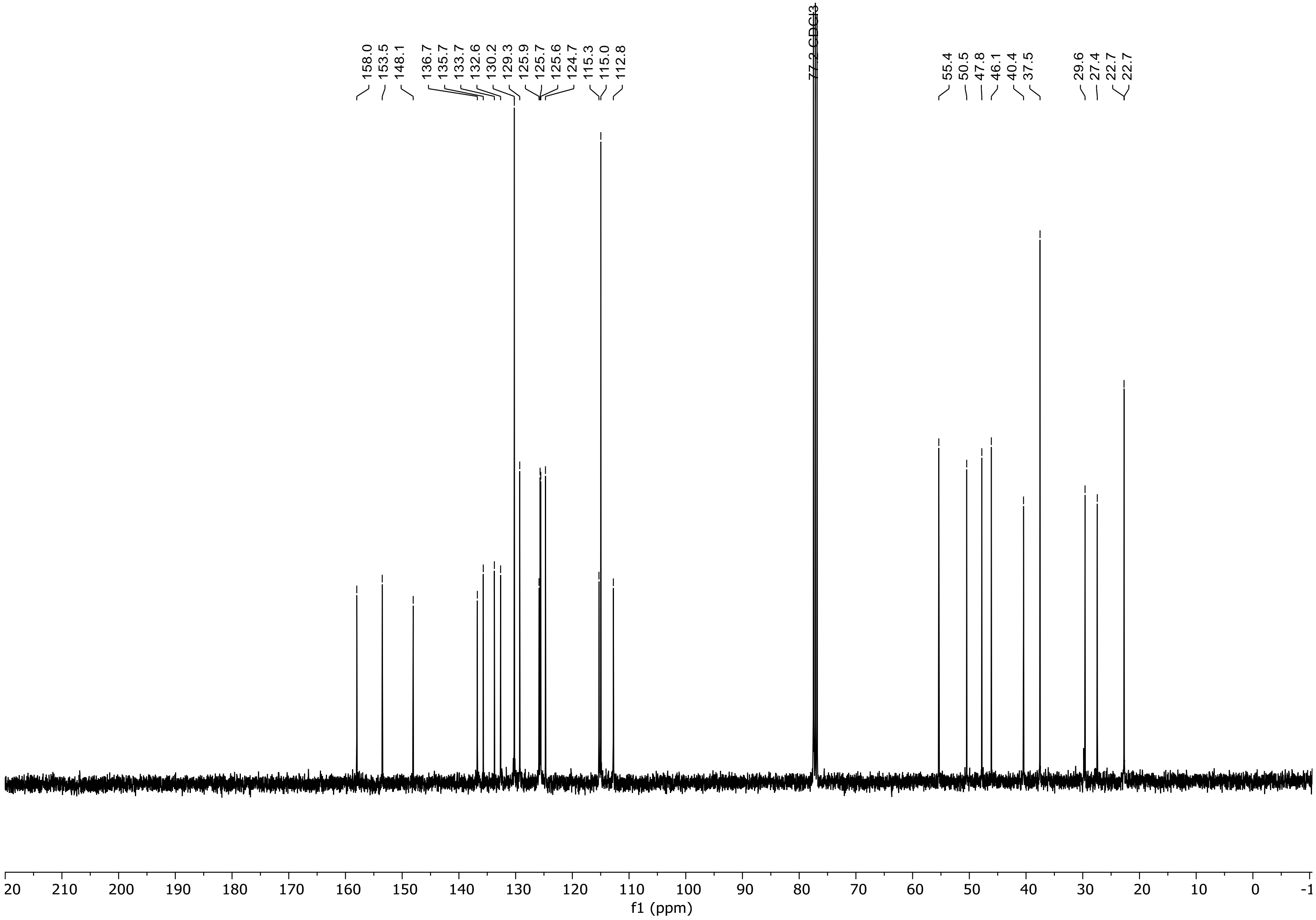

(±)-4-(((1*R*,2*R*,4*aS*,10*aS*)-1-(4-methoxyphenyl)-4*a*-methyl-1,2,3,4,4*a*,9,10,10*a*-octahydrophenanthren-2-yl)methyl)aniline 2c.

^{1}H NMR (400 MHz, $CDCl_3$)

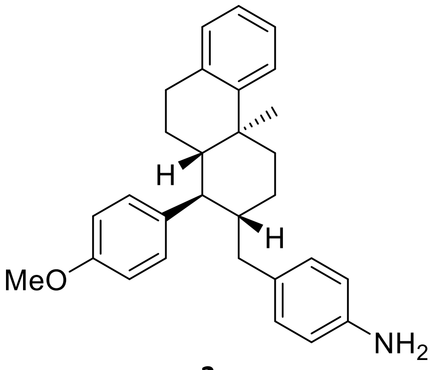

2c

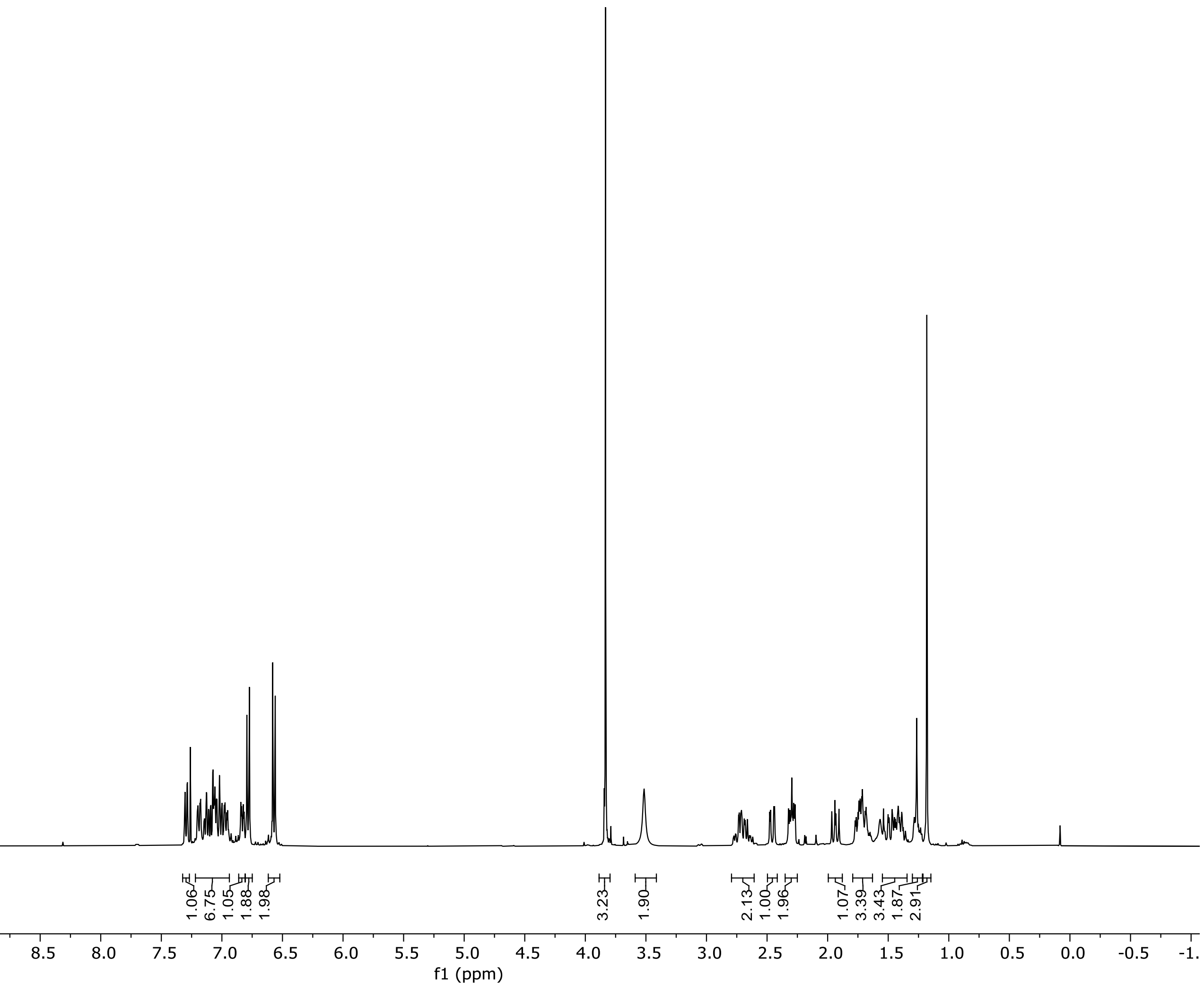

^{13}C NMR (101 MHz, $CDCl_3$)

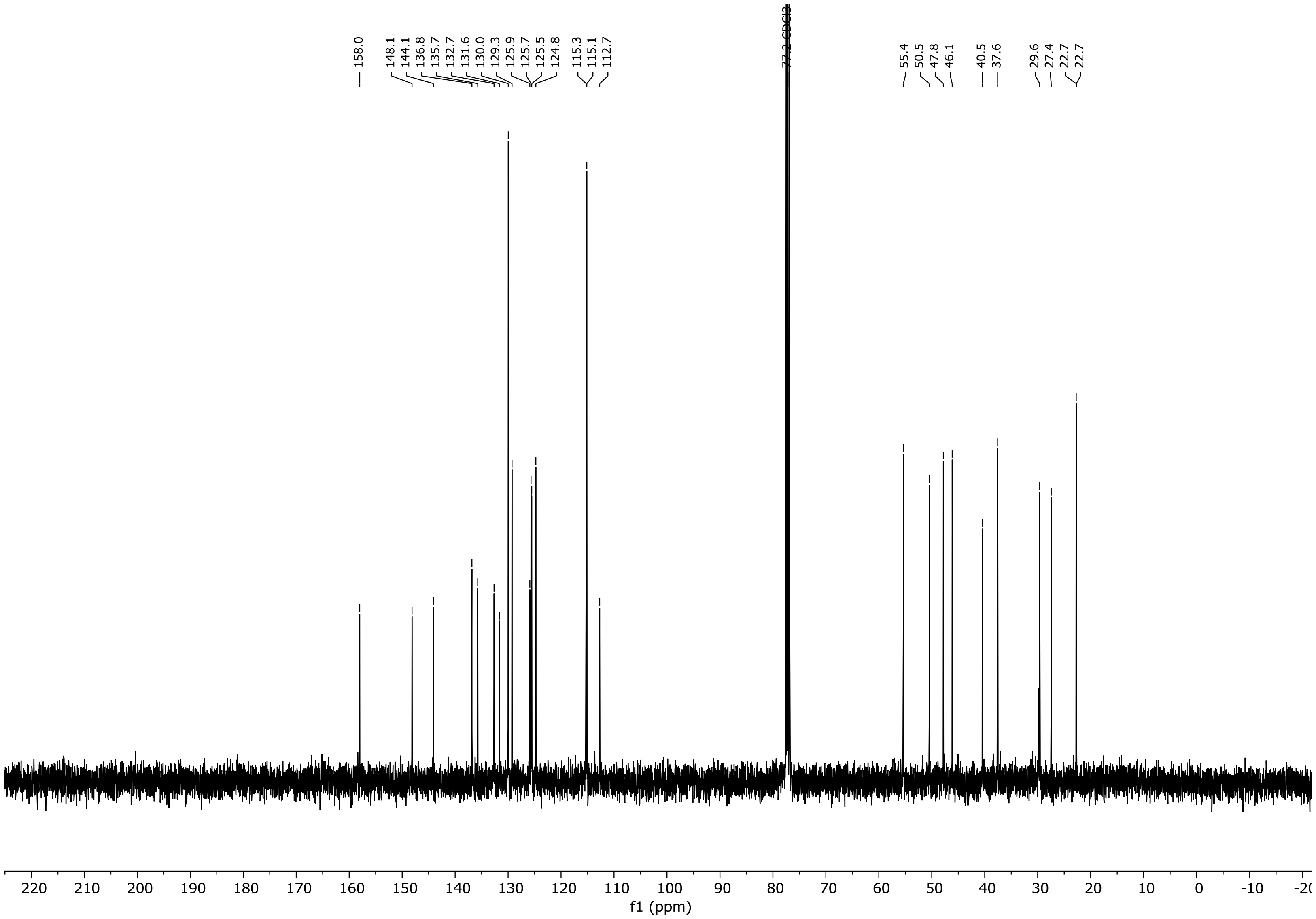

(±)-(1*R*,2*R*,4*aS*,10*aS*)-2-(2,4-Dimethoxybenzyl)-1-(4-methoxyphenyl)-4*a*-methyl-1,2,3,4,4*a*,9,10,10*a*-octahydrophenanthrene 2d.

^{1}H NMR (400 MHz, $CDCl_3$)

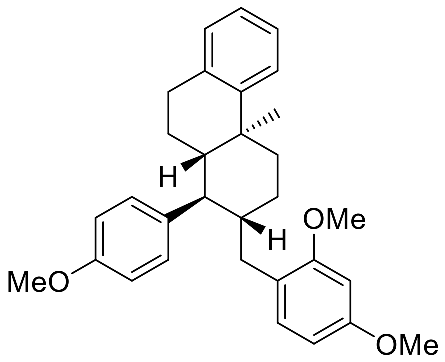

2d

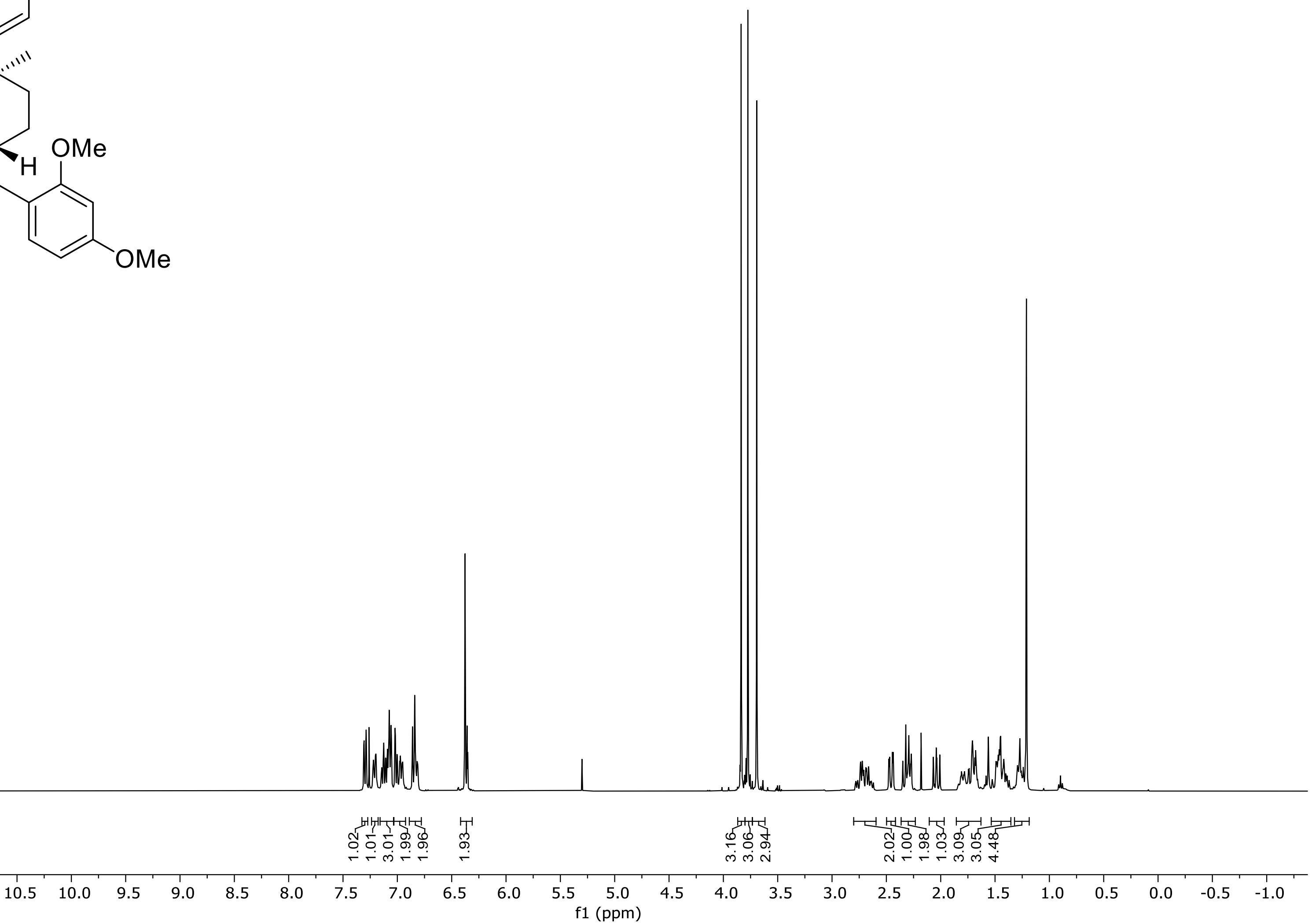

^{13}C NMR (101 MHz, $CDCl_3$)

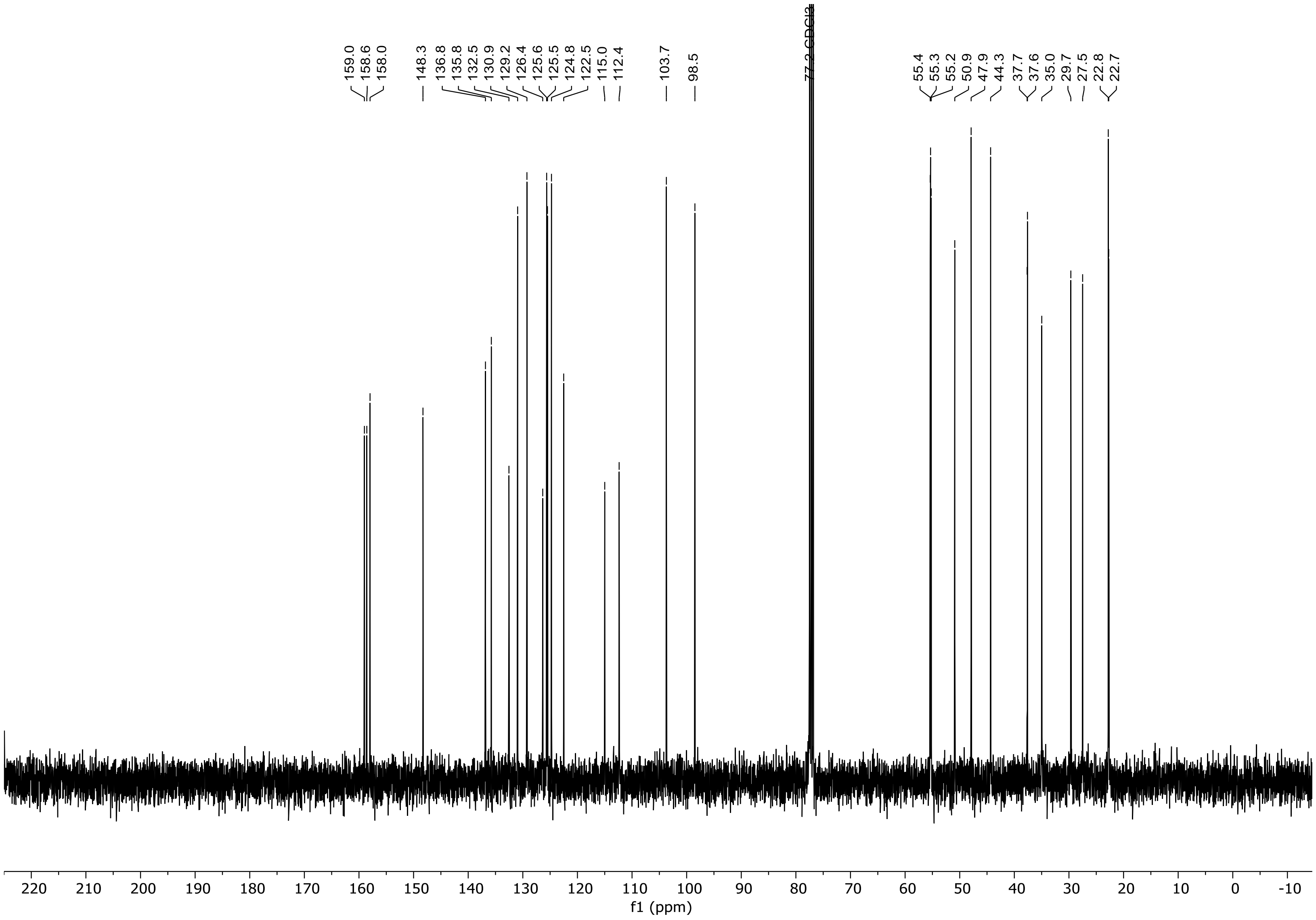

(±)-(1*R*,2*R*,4*aS*,10*aS*)-2-(2-Bromo-4-methoxybenzyl)-1-(4-methoxyphenyl)-4*a*-methyl-1,2,3,4,4*a*,9,10,10*a*-octahydrophenanthrene 2e.

^{1}H NMR (400 MHz, $CDCl_3$)

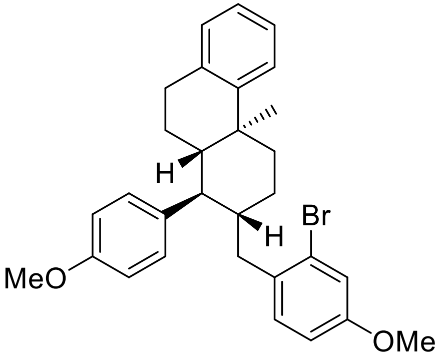

2e

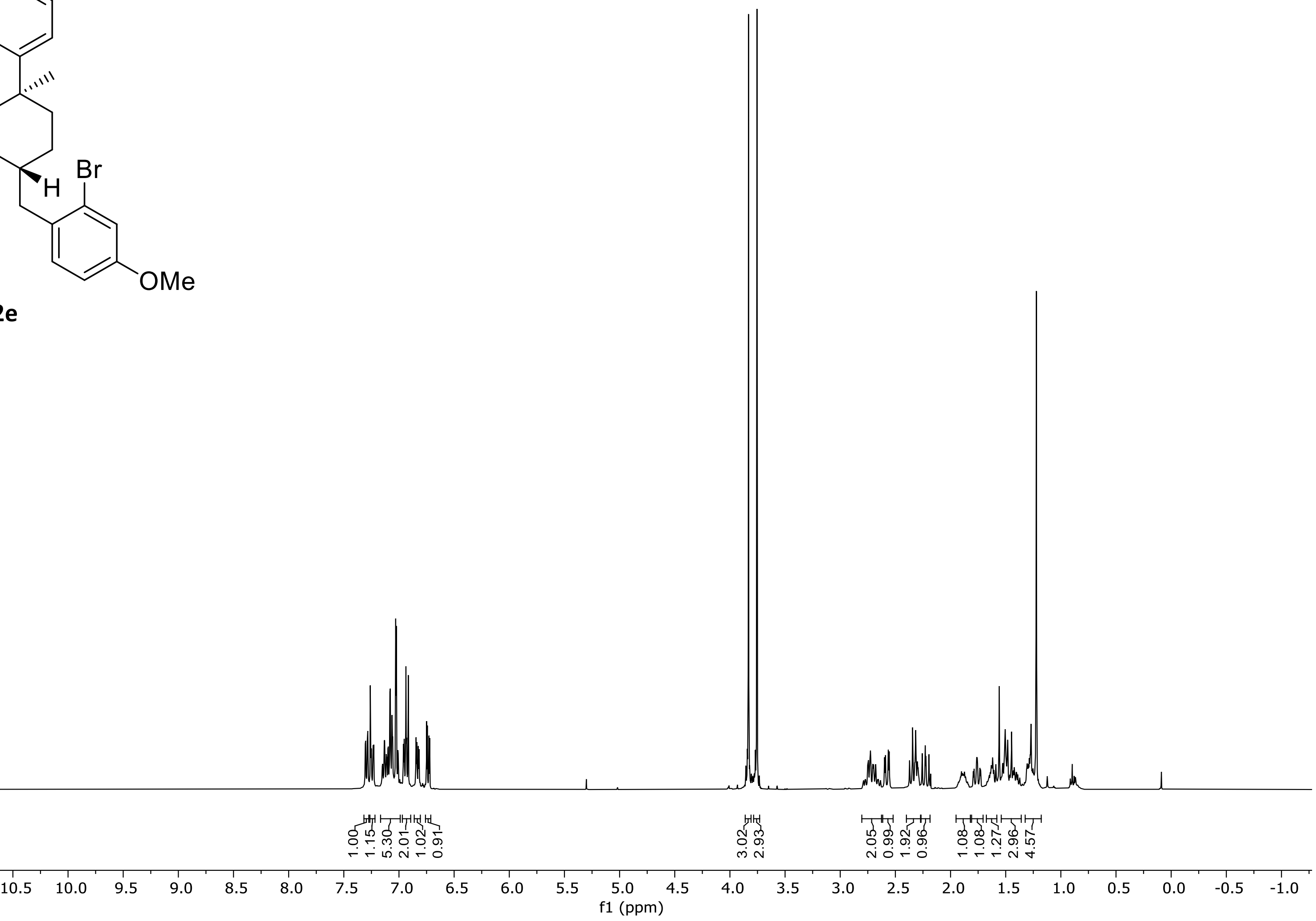

^{13}C NMR (101 MHz, $CDCl_3$)

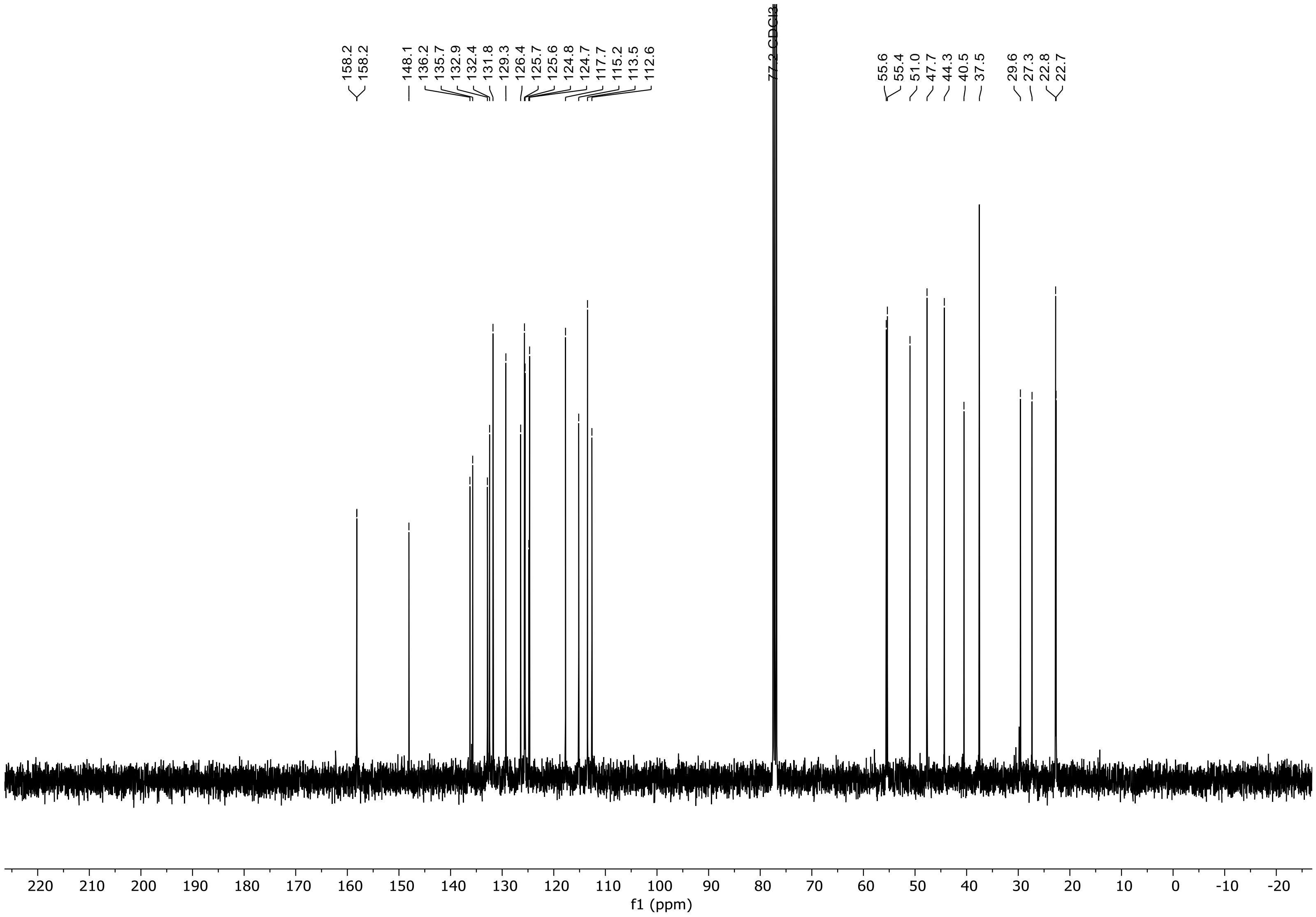

NOESY-2D (600 MHz, $CDCl_3$)

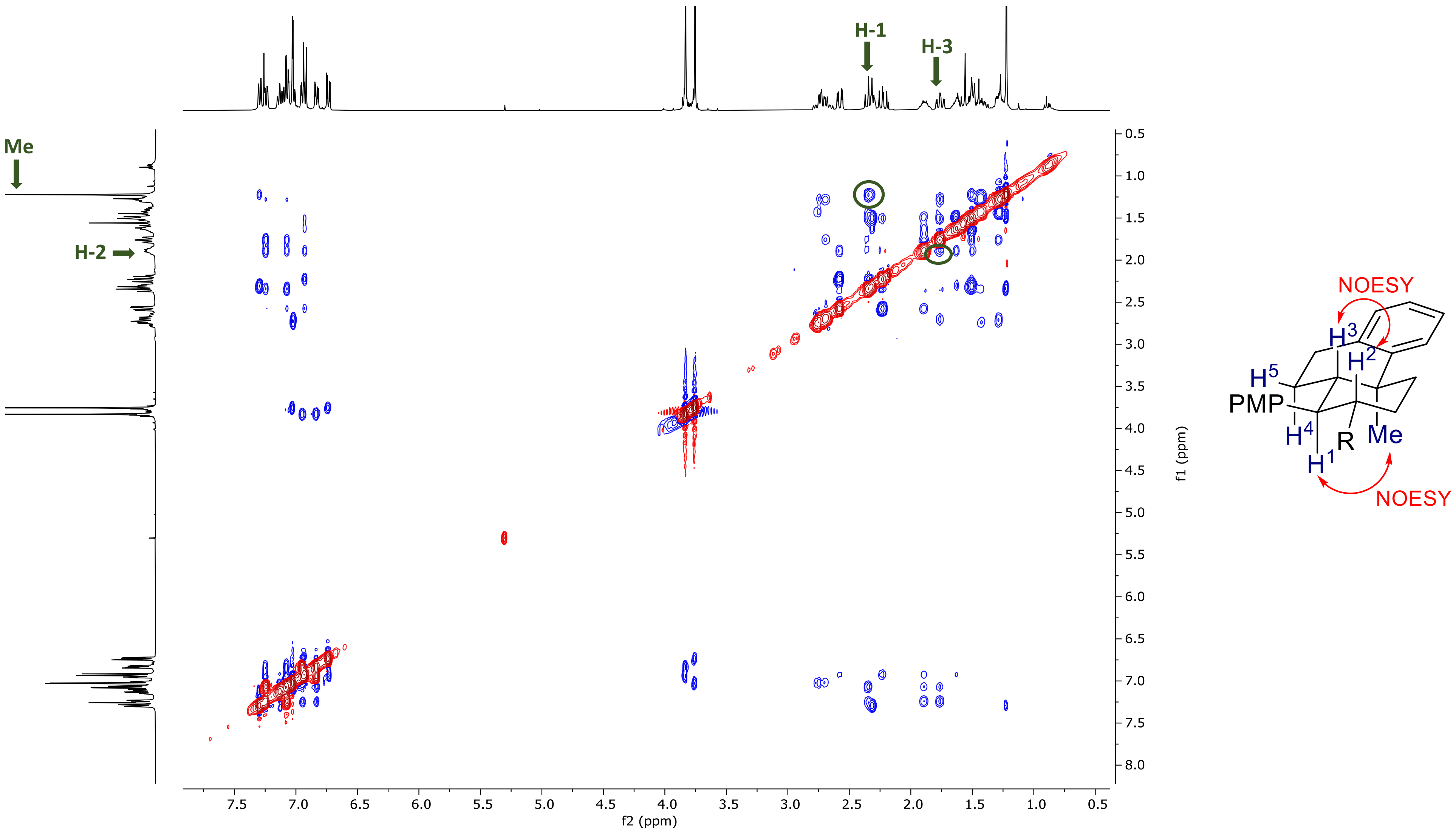

(±)-3-((1*R*,2*R*,4*aS*,10*aS*)-1-(4-Methoxyphenyl)-4*a*-methyl-1,2,3,4,4*a*,9,10,10*a*-octahydrophenanthren-2-yl)methyl)-1-methyl-1H-indole 2f.

^{1}H NMR (400 MHz, $CDCl_3$)

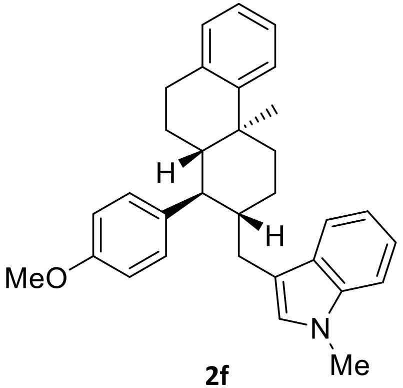

2f

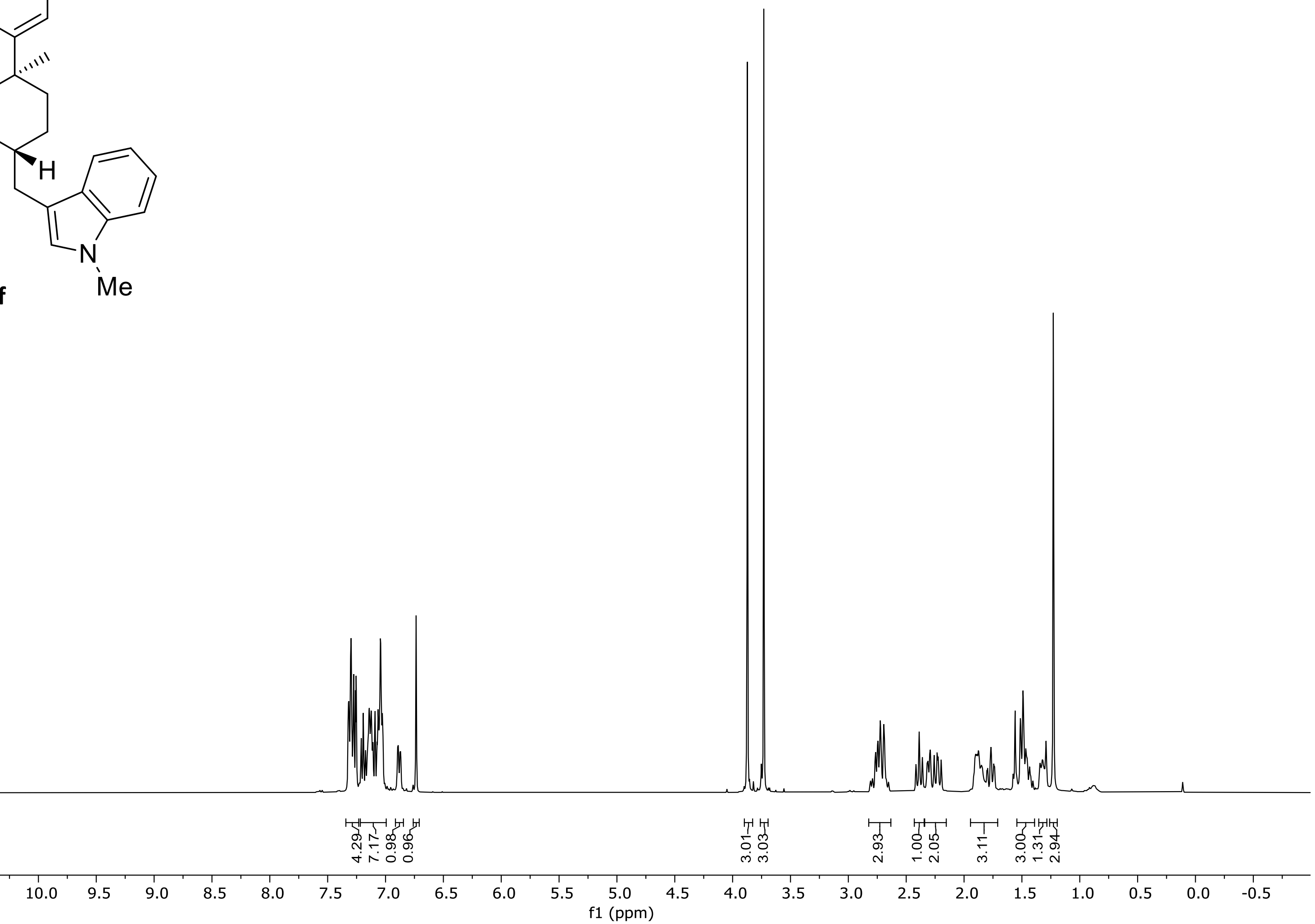

^{13}C NMR (101 MHz, $CDCl_3$)

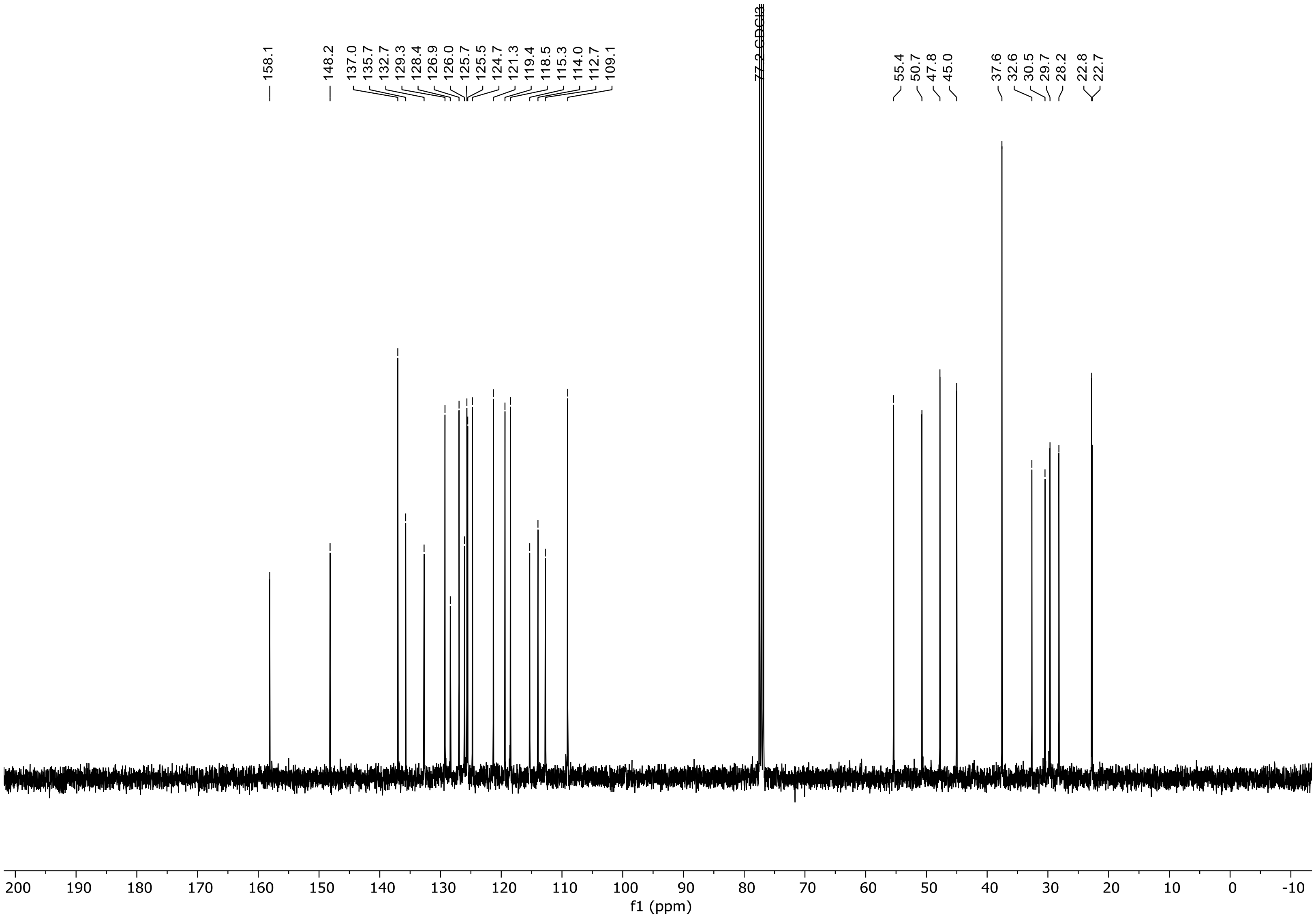

NOESY-2D (600 MHz, $CDCl_3$)

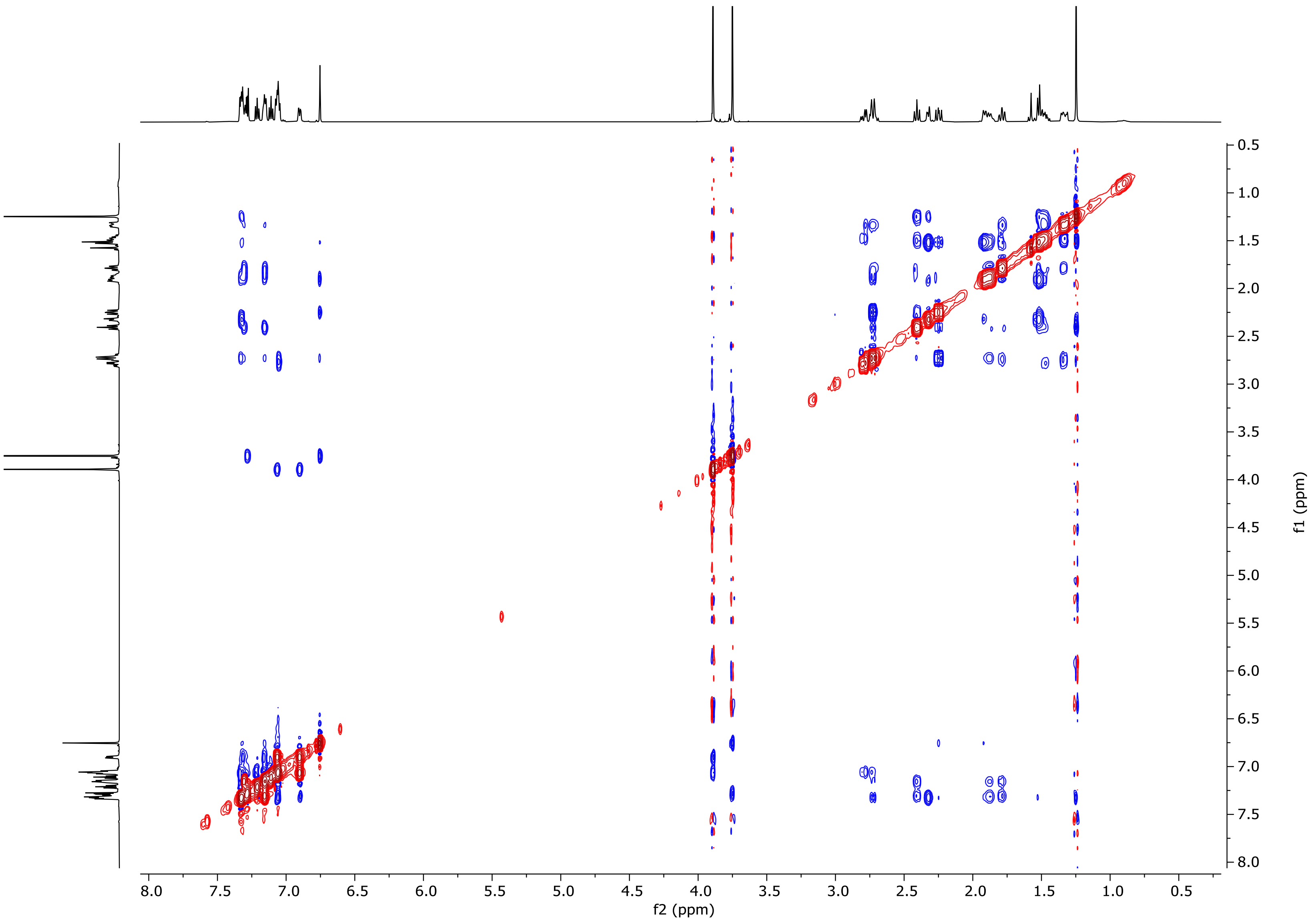

(±)-(1*R*,2*R*,4a*S*,10a*S*)-1-(4-Methoxyphenyl)-2-((*S*)-1-(4-methoxyphenyl)ethyl)-4*a*-methyl-1,2,3,4,4*a*,9,10,10*a*-octahydrophenanthrene (major)-2g and (±)-(1*R*,2*R*,4a*S*,10a*S*)-1-(4-Methoxyphenyl)-2-((*R*)-1-(4-methoxyphenyl)ethyl)-4*a*-methyl-1,2,3,4,4*a*,9,10,10*a*-octahydrophenanthrene (minor)-2g.

^{1}H NMR (400 MHz, $CDCl_3$)

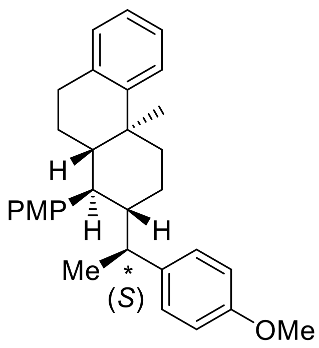

2g (major);

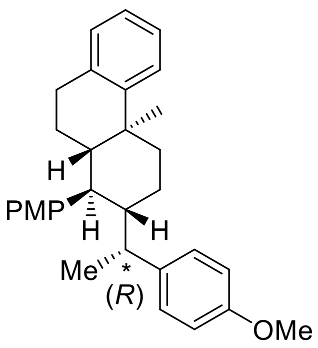

2g (minor);

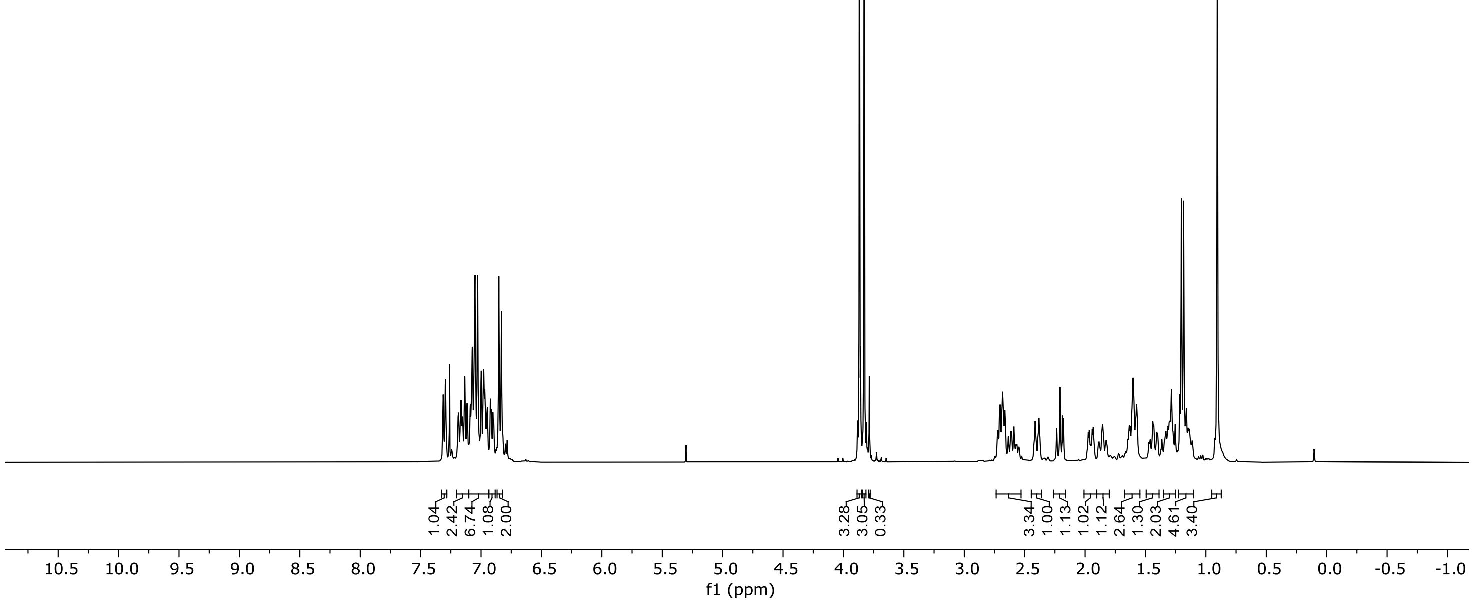

^{13}C NMR (101 MHz, $CDCl_3$)

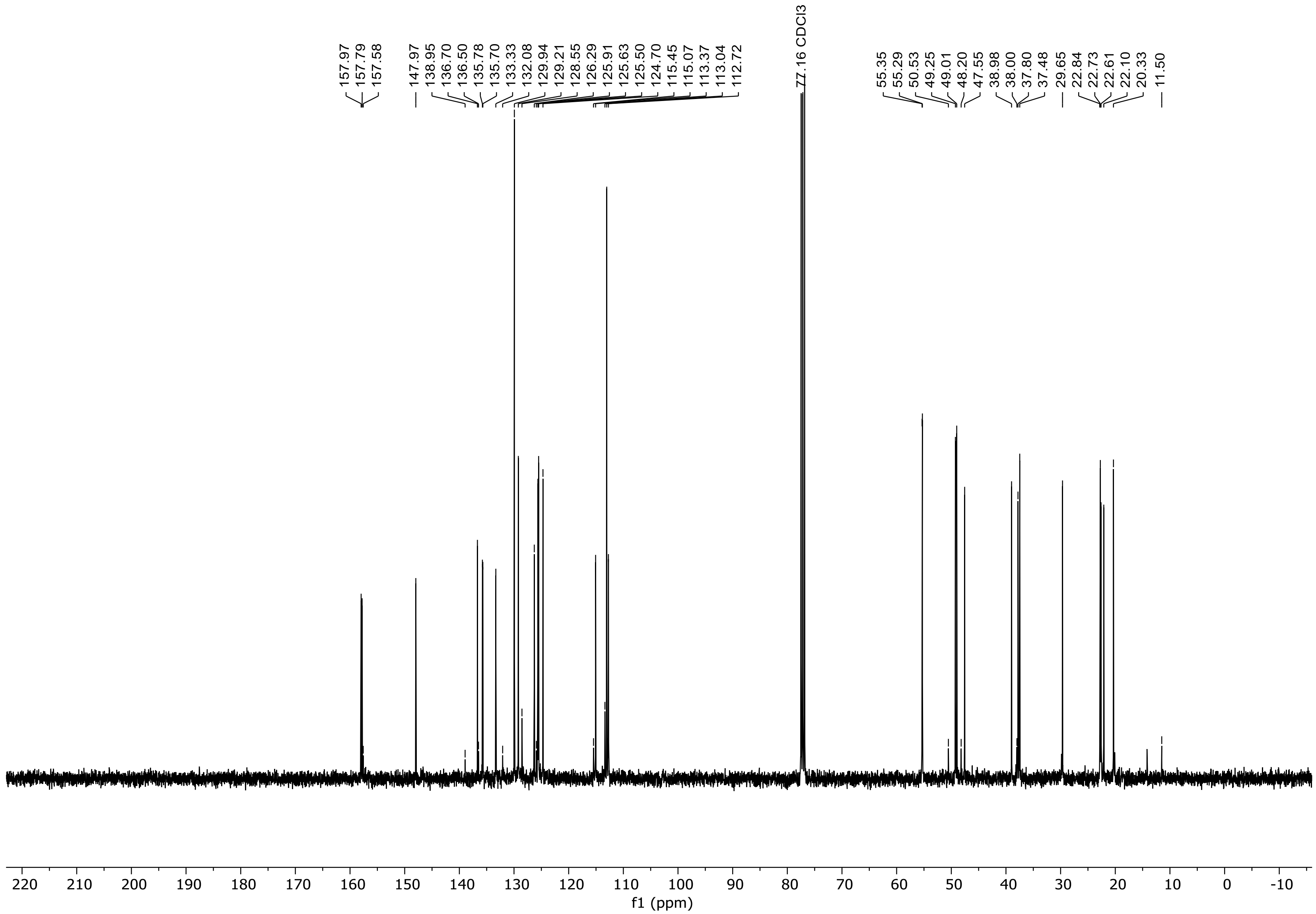

NOESY-2D (600 MHz, $CDCl_3$)

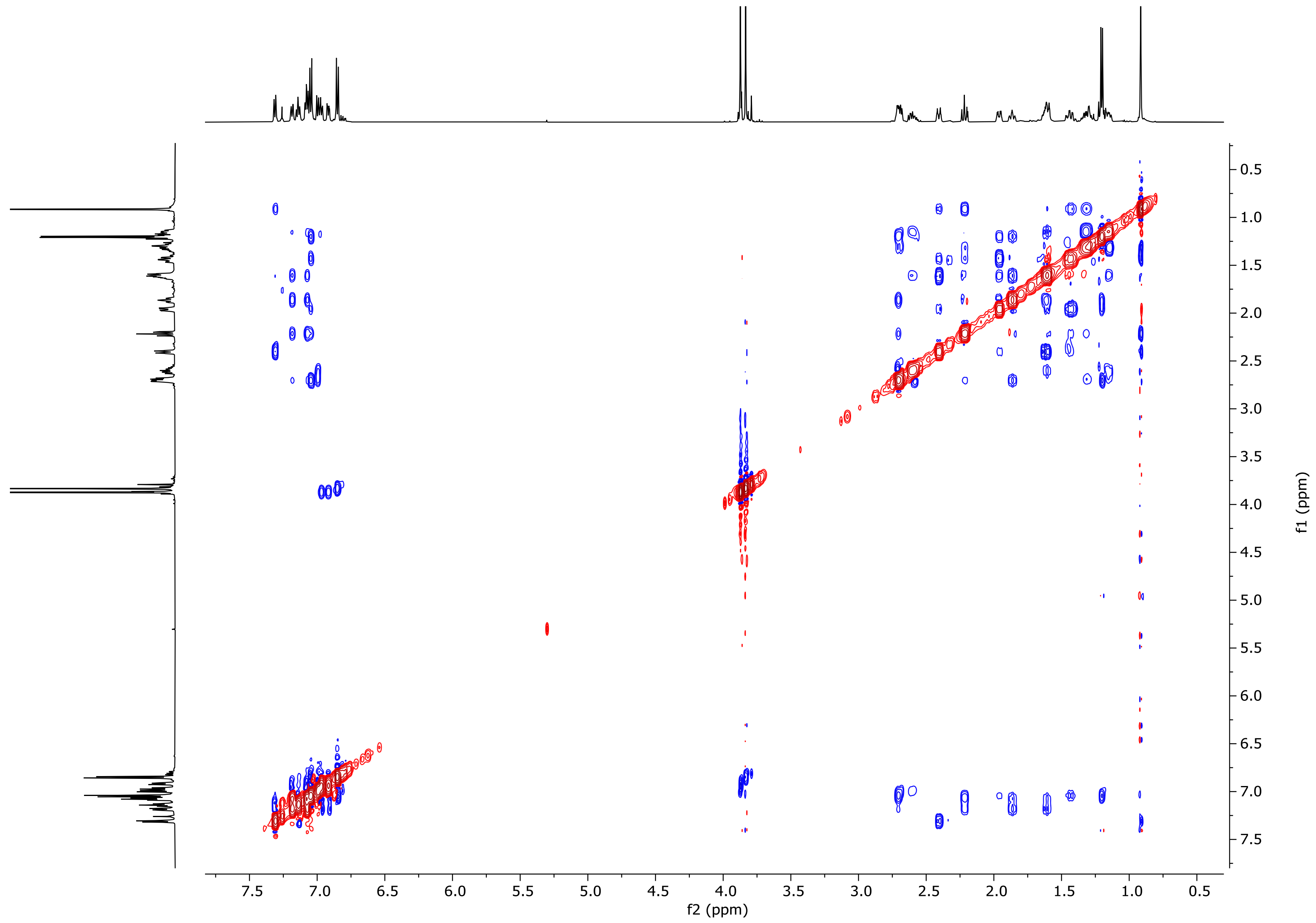

(±)-5-((*S*)-1-((1*R*,2*R*,4*aS*,10*aS*)-1-(4-Methoxyphenyl)-4*a*-methyl-1,2,3,4,4*a*,9,10,10*a*-octahydrophenanthren-2-yl)ethyl)benzo[d][1,3]dioxole (majo*r*)-2h and (±)-5-((*R*)-1-((1*R*,2*R*,4*aS*,10*aS*)-1-(4-methoxyphenyl)-4*a*-methyl-1,2,3,4,4*a*,9,10,10*a*-octahydrophenanthren-2-yl)ethyl)benzo[d][1,3]dioxole (minor)-2h.

^{1}H NMR (400 MHz, $CDCl_3$)

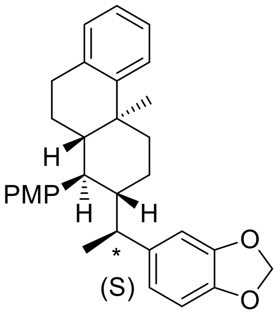

2h (major)

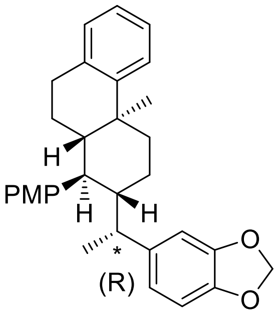

2h (minor)

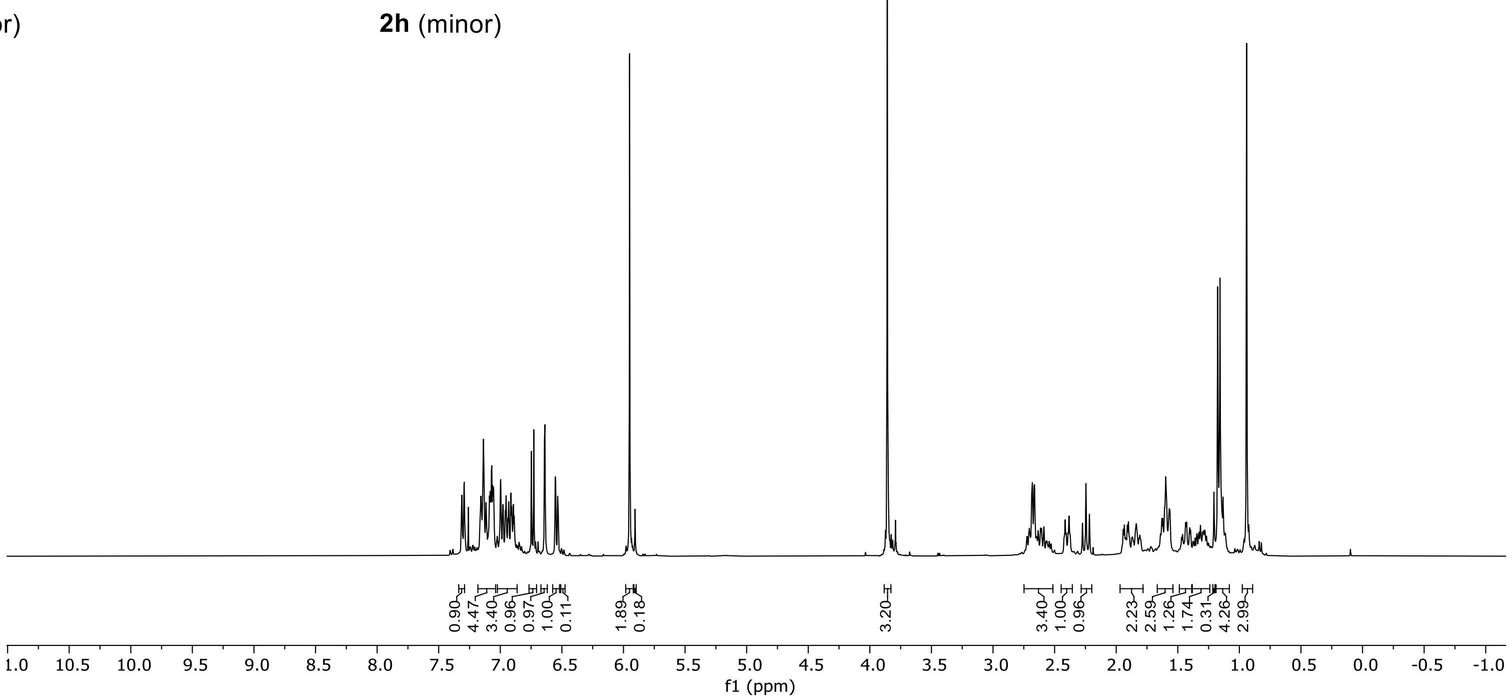

^{13}C NMR (101 MHz, $CDCl_3$)

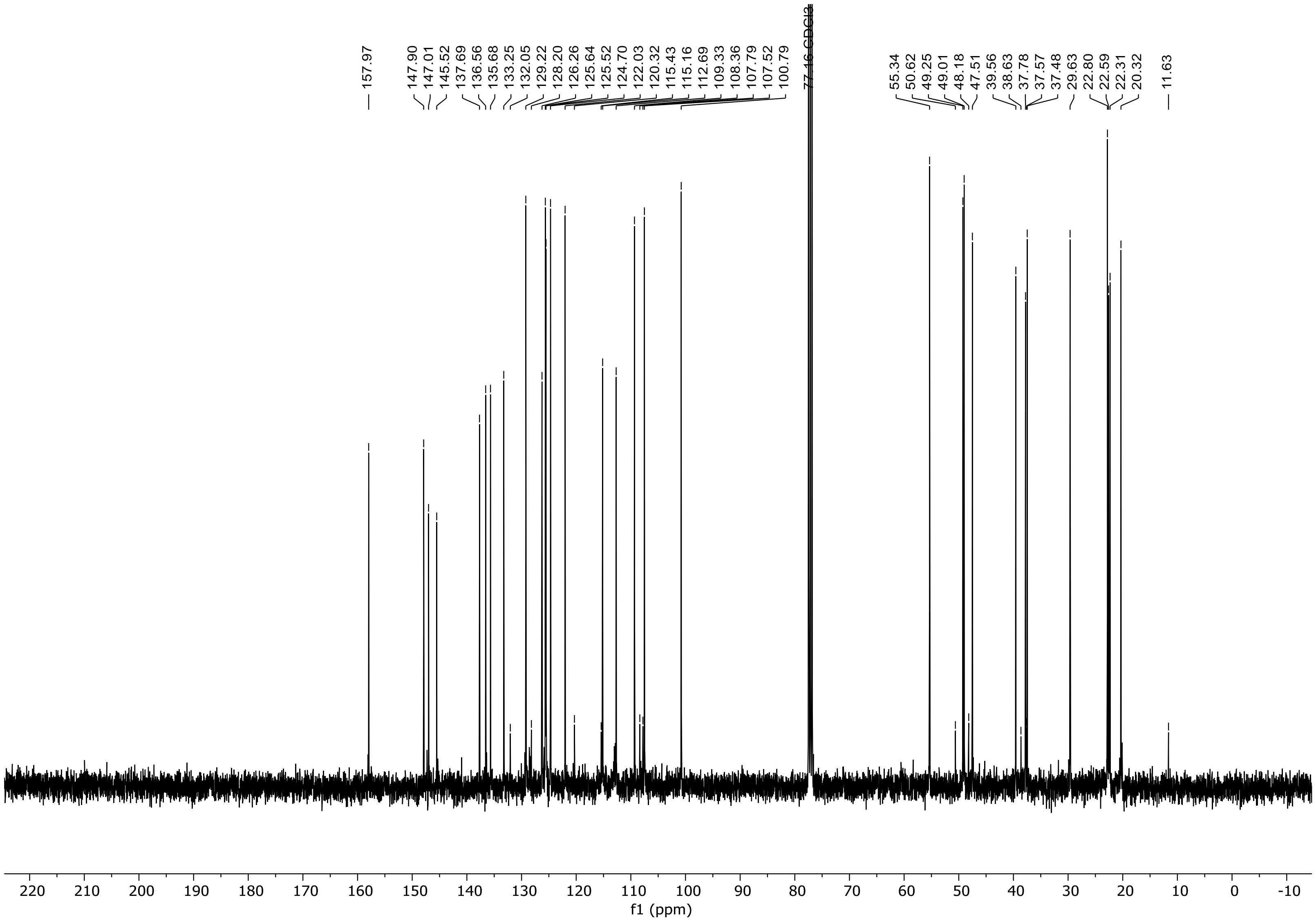

(±)-4-((1*S*)-1-((1*R*,4*aS*,10*aS*)-1-(4-Methoxyphenyl)-4*a*-methyl-1,2,3,4,4*a*,9,10,10*a*-octahydrophenanthren-2-yl)ethyl)-2-methylphenol (major)-2i and (±)-4-((1*R*)-1-((1*R*,4*aS*,10*aS*)-1-(4-methoxyphenyl)-4*a*-methyl-1,2,3,4,4*a*,9,10,10*a*-octahydrophenanthren-2-yl)ethyl)-2-methylphenol (minor)-2i.

^{1}H NMR (400 MHz, $CDCl_3$)

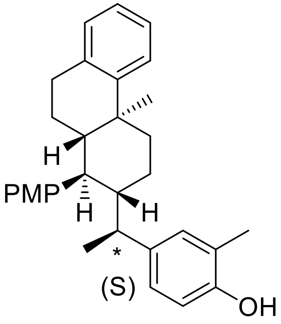

2i (major)

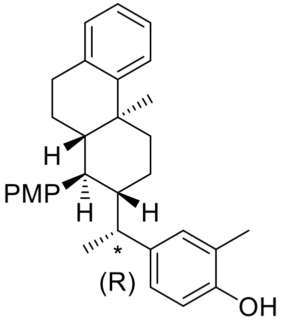

2i (minor)

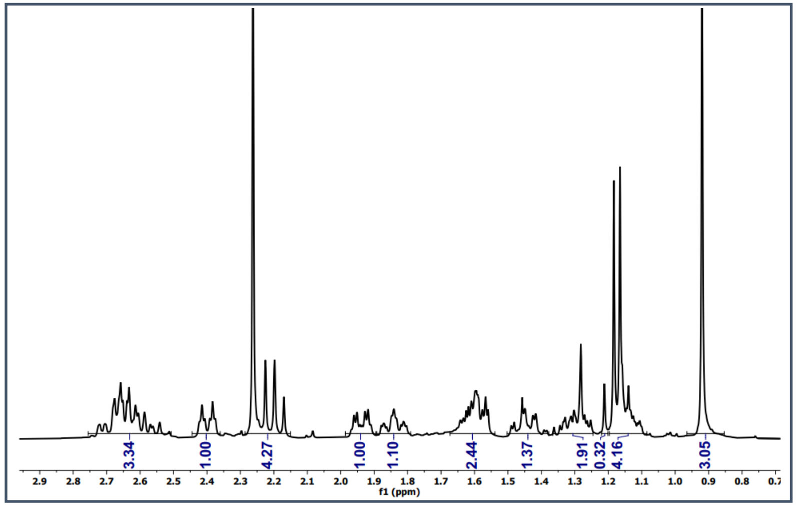

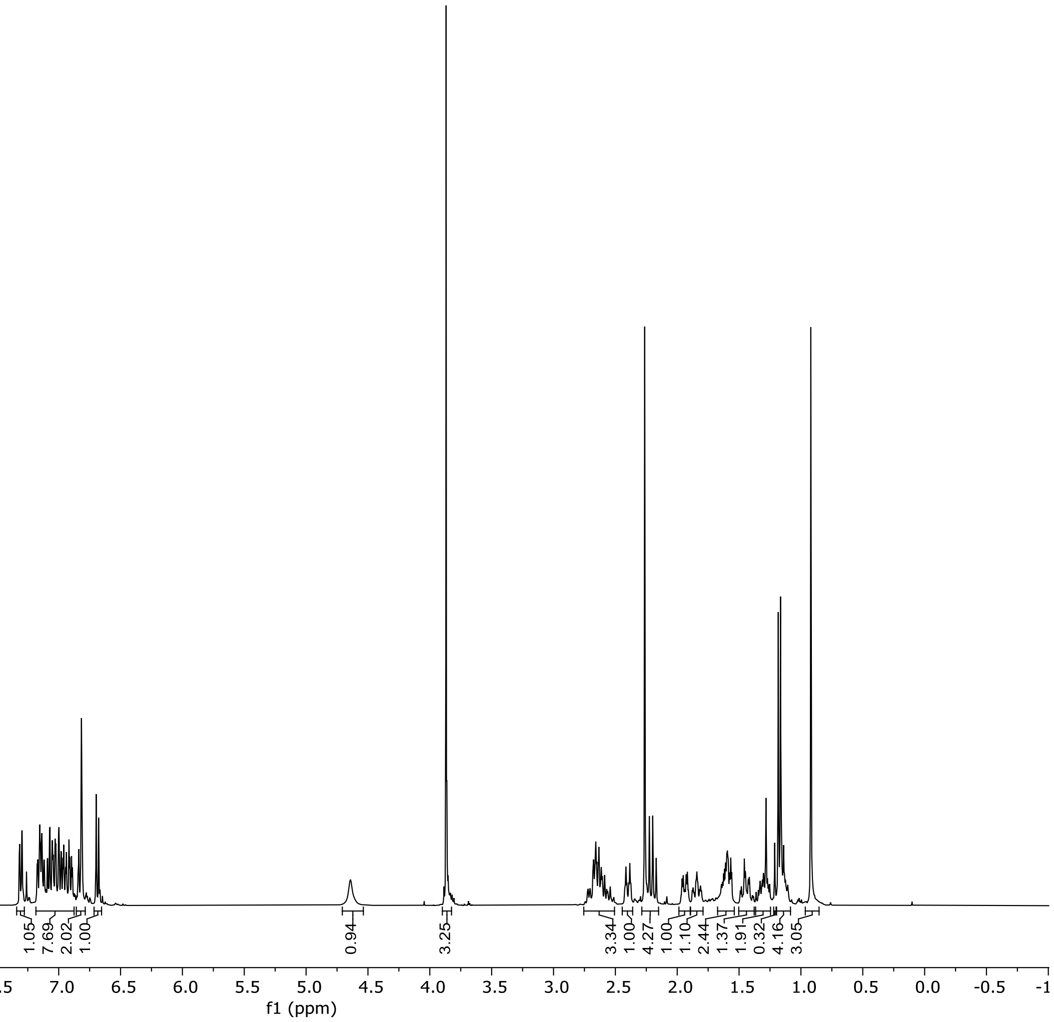

^{13}C NMR (101 MHz, $CDCl_3$)

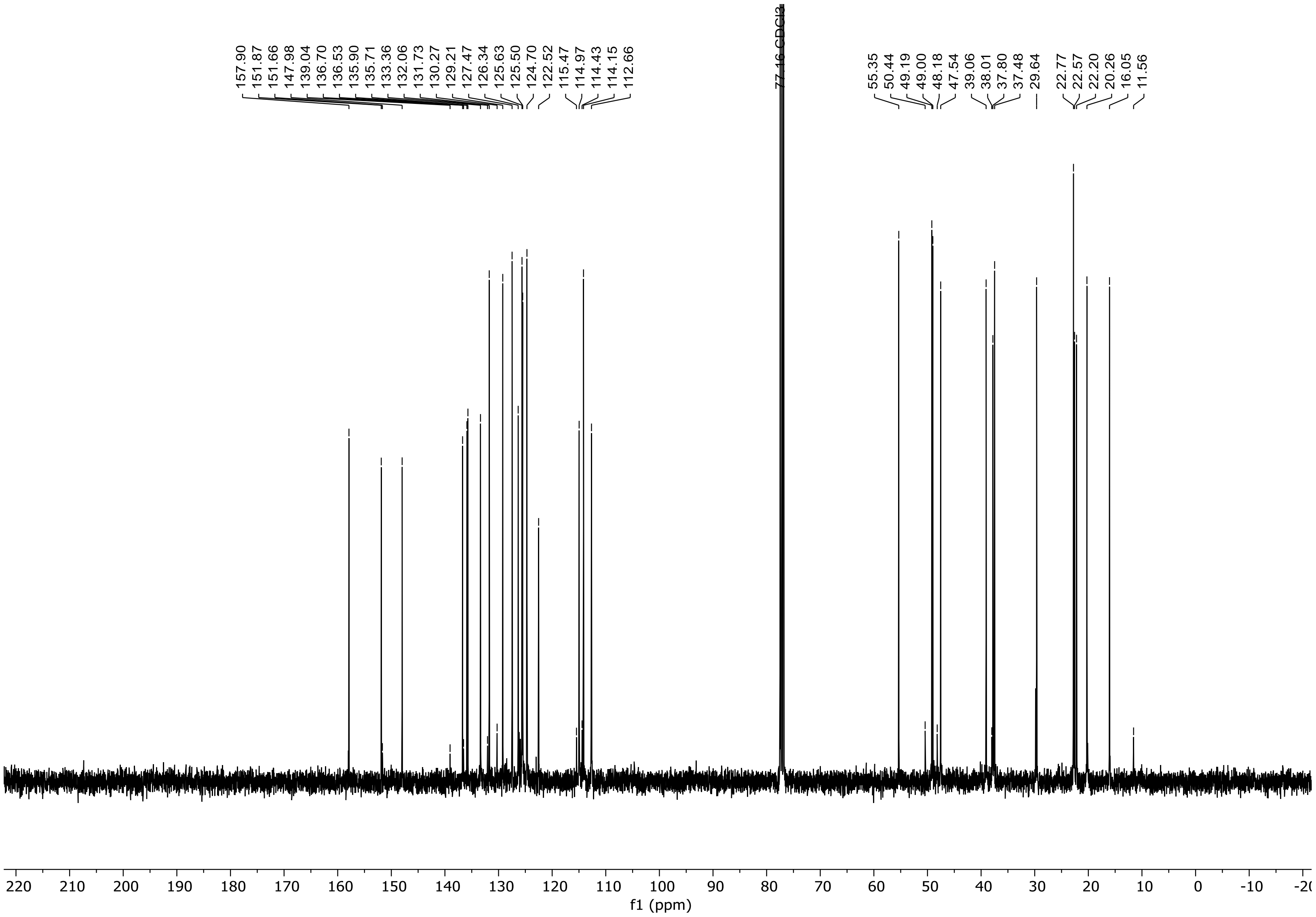

NOESY-2D (600 MHz, $CDCl_3$)

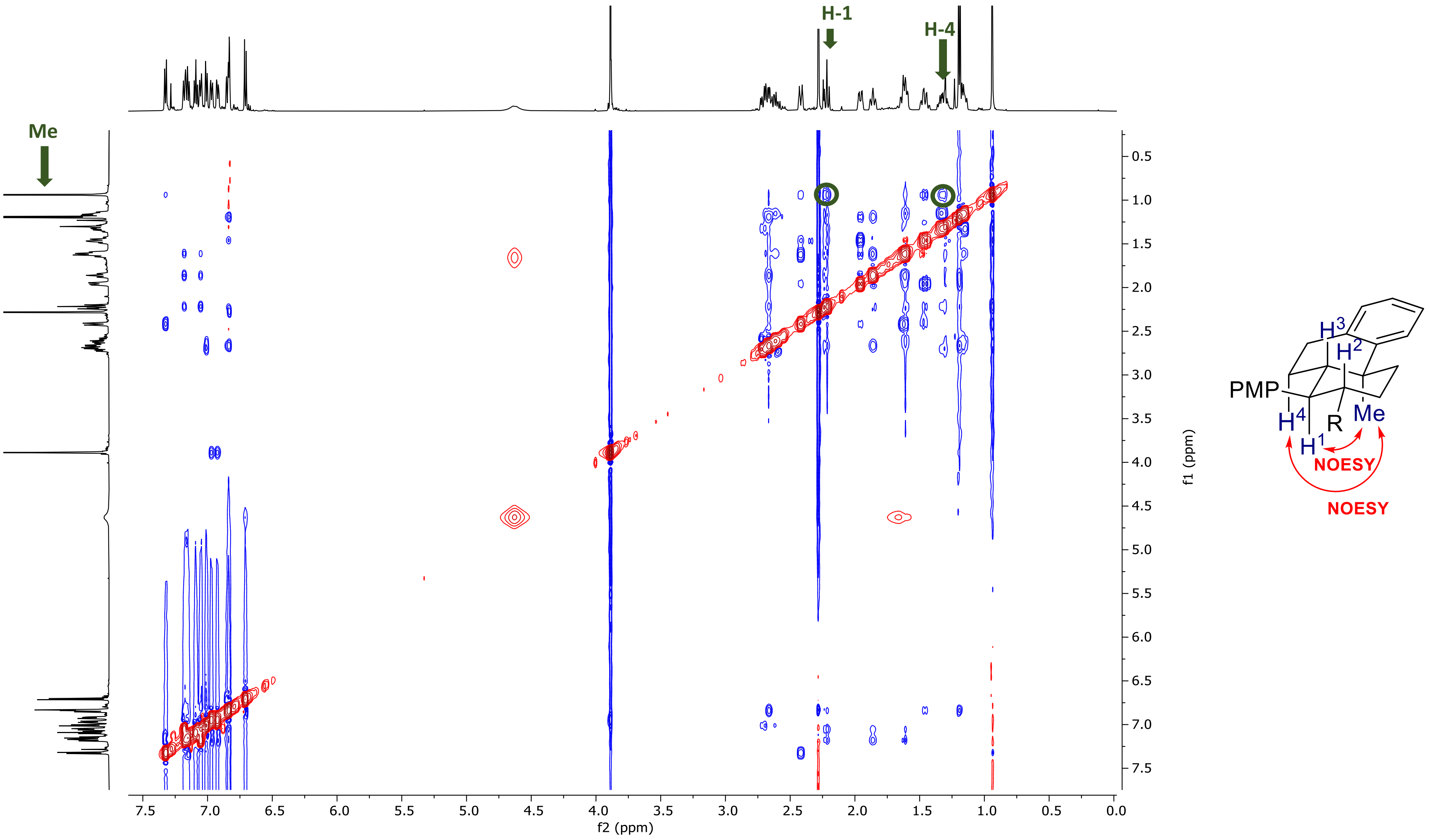

(±)-2-((*S*)-1-((1*R*,2*S*,4*aS*,10*aS*)-1-(4-Methoxyphenyl)-4a-methyl-1,2,3,4,4*a*,9,10,10*a*-octahydrophenanthren-2-yl)ethyl)thiophene (major)-2j and (±)-2-((*R*)-1-((1*R*,2*S*,4*aS*,10*aS*)-1-(4-Methoxyphenyl)-4*a*-methyl-1,2,3,4,4*a*,9,10,10*a*-octahydrophenanthren-2-yl)ethyl)thiophene (minor)-2j.

^{1}H NMR (400 MHz, $CDCl_3$)

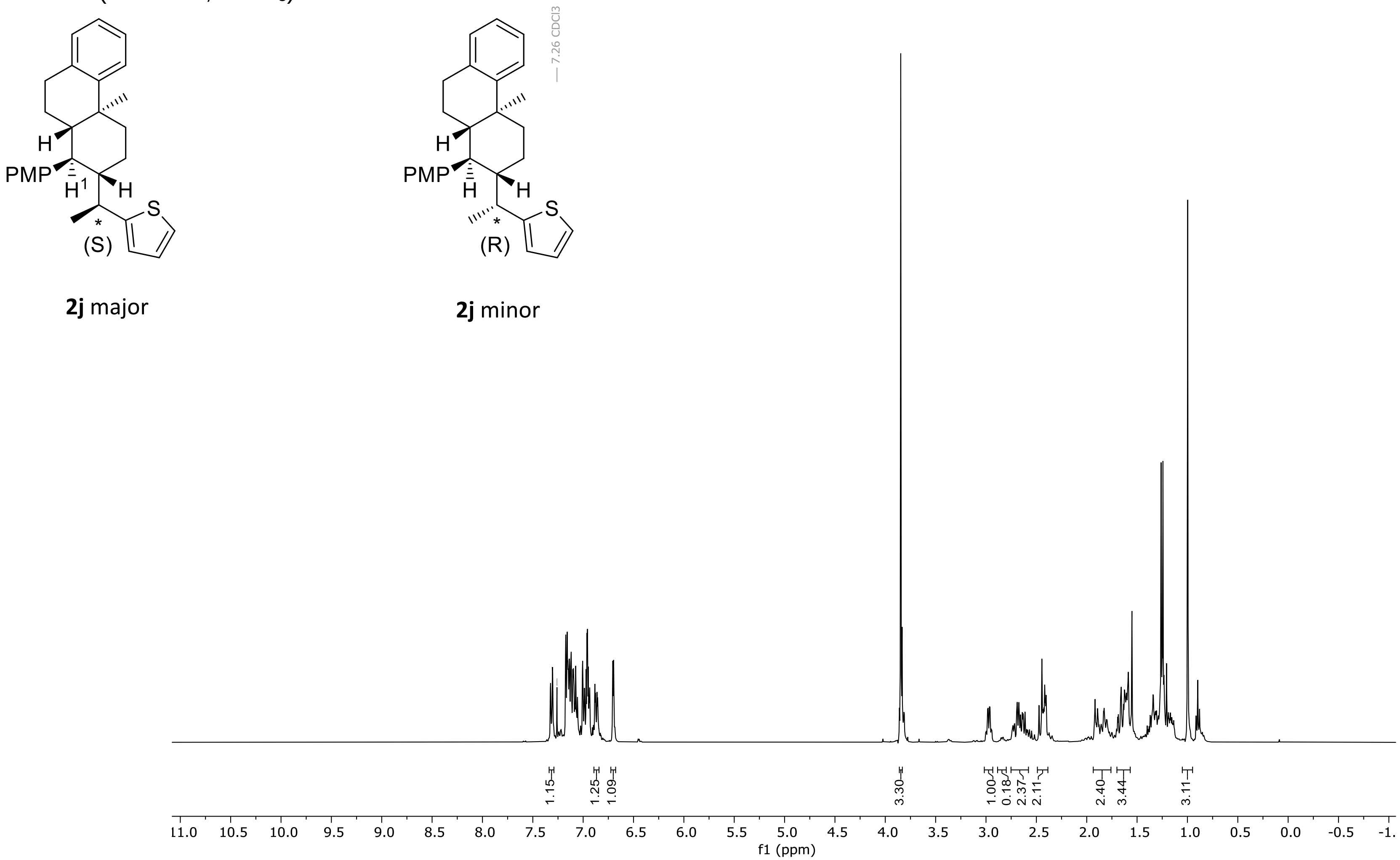

^{13}C NMR (101 MHz, $CDCl_3$)

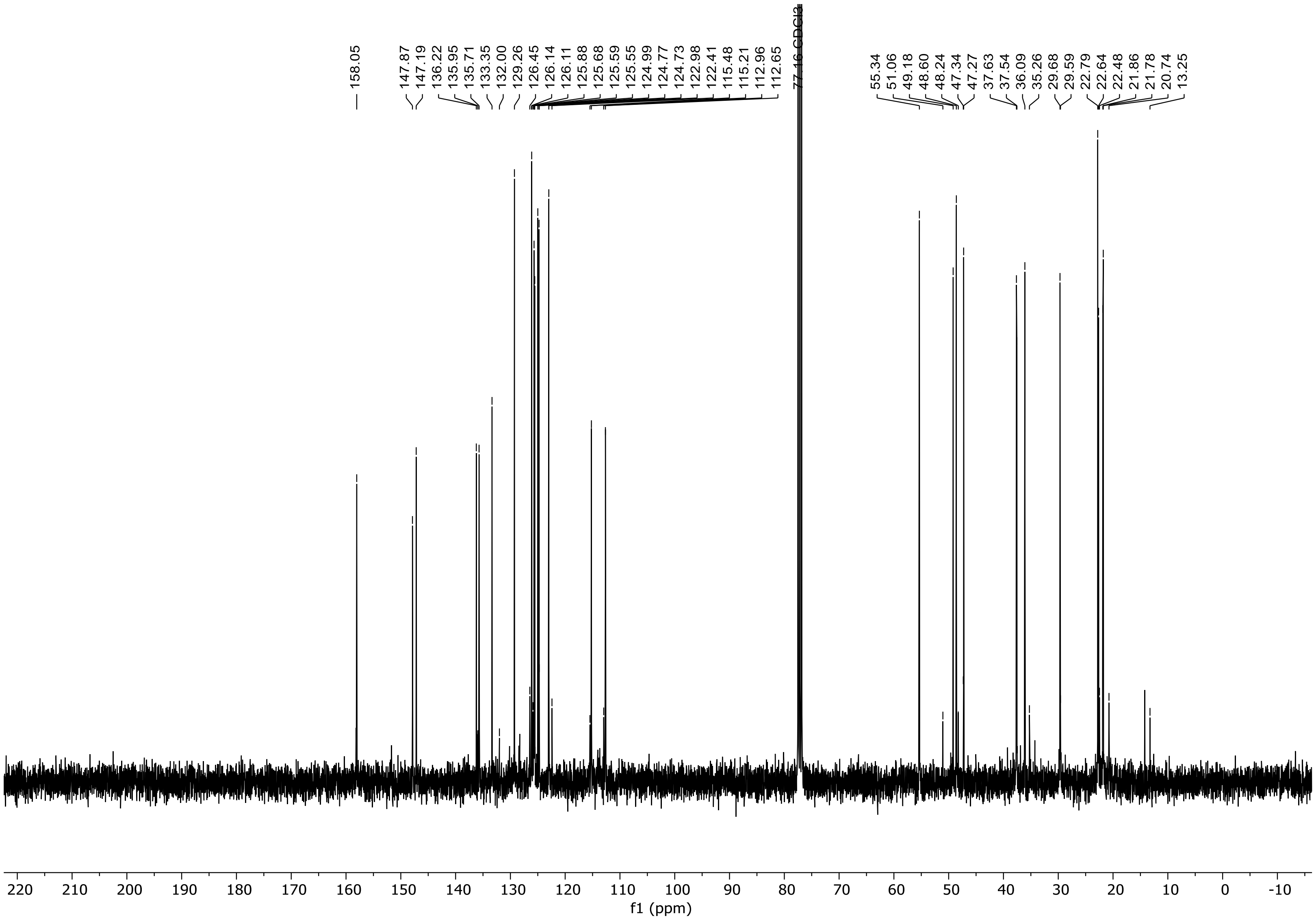

(±)-(1*R*,2*R*,4a*S*,10a*S*)-2-((*S*)-2,3-Dihydro-1H-inden-1-yl)-1-(4-methoxyphenyl)-4*a*-methyl-1,2,3,4,4*a*,9,10,10*a*-octahydrophenanthrene (major)-2k and (±)-(1*R*,2*R*,4a*S*,10a*S*)-2-((*R*)-2,3-Dihydro-1H-inden-1-yl)-1-(4-methoxyphenyl)-4*a*-methyl-1,2,3,4,4*a*,9,10,10*a*-octahydrophenanthrene (minor)-2k.

^{1}H NMR (600 MHz, $CDCl_3$)

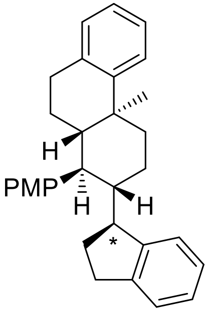

2k (major)

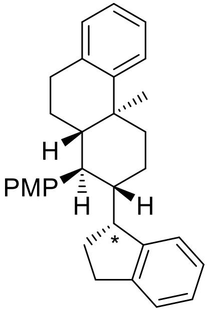

2k (minor)

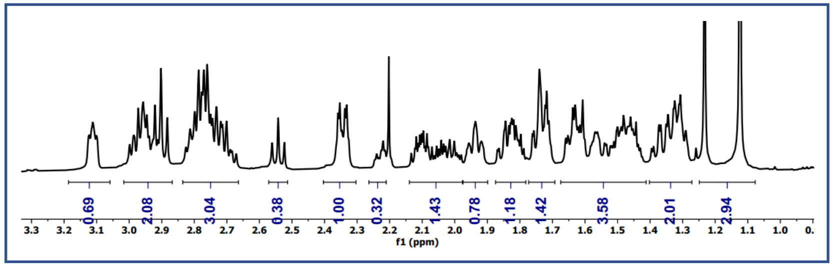

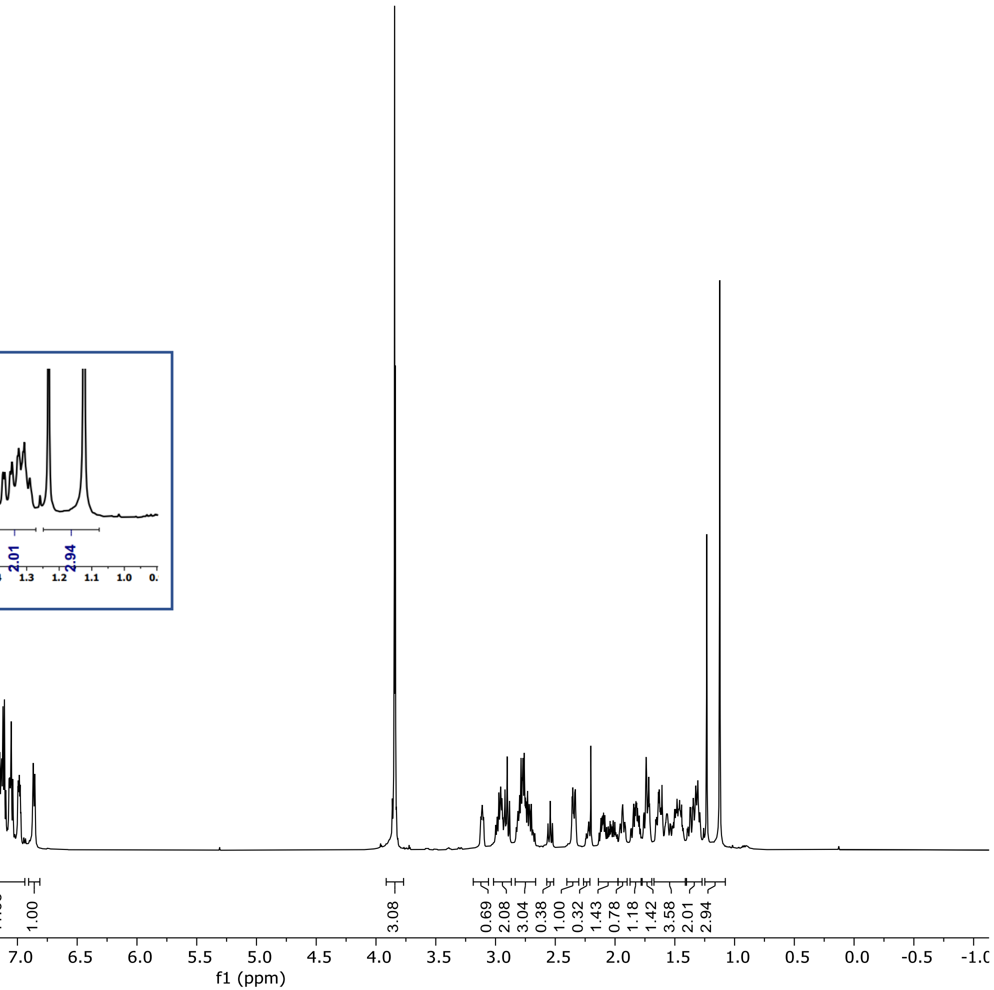

^{13}C NMR (101 MHz, $CDCl_3$)

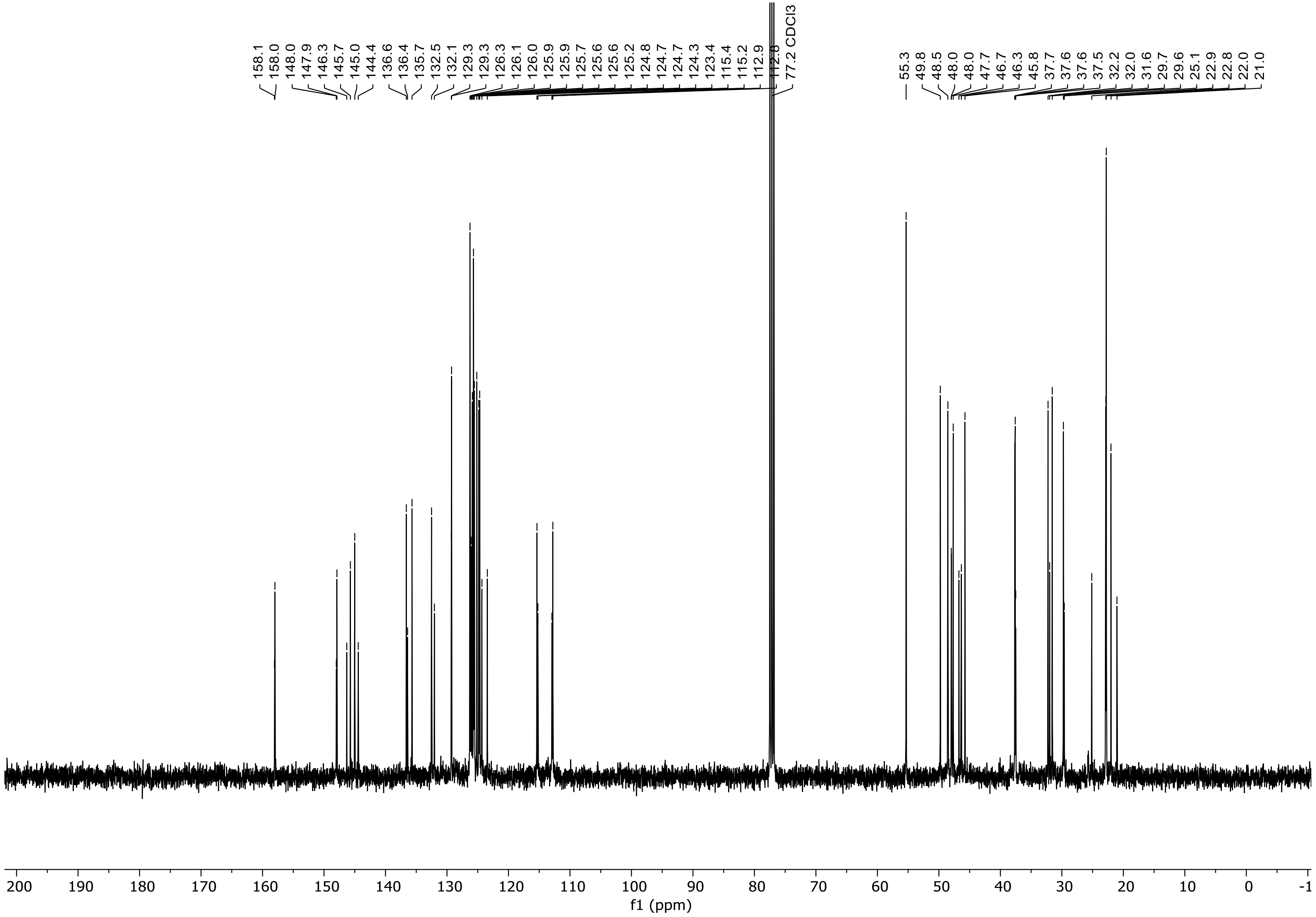

^{1}H (400 MHz)-**^{13}C** (101 MHz) **HSQC-2D** ($CDCl_3$)

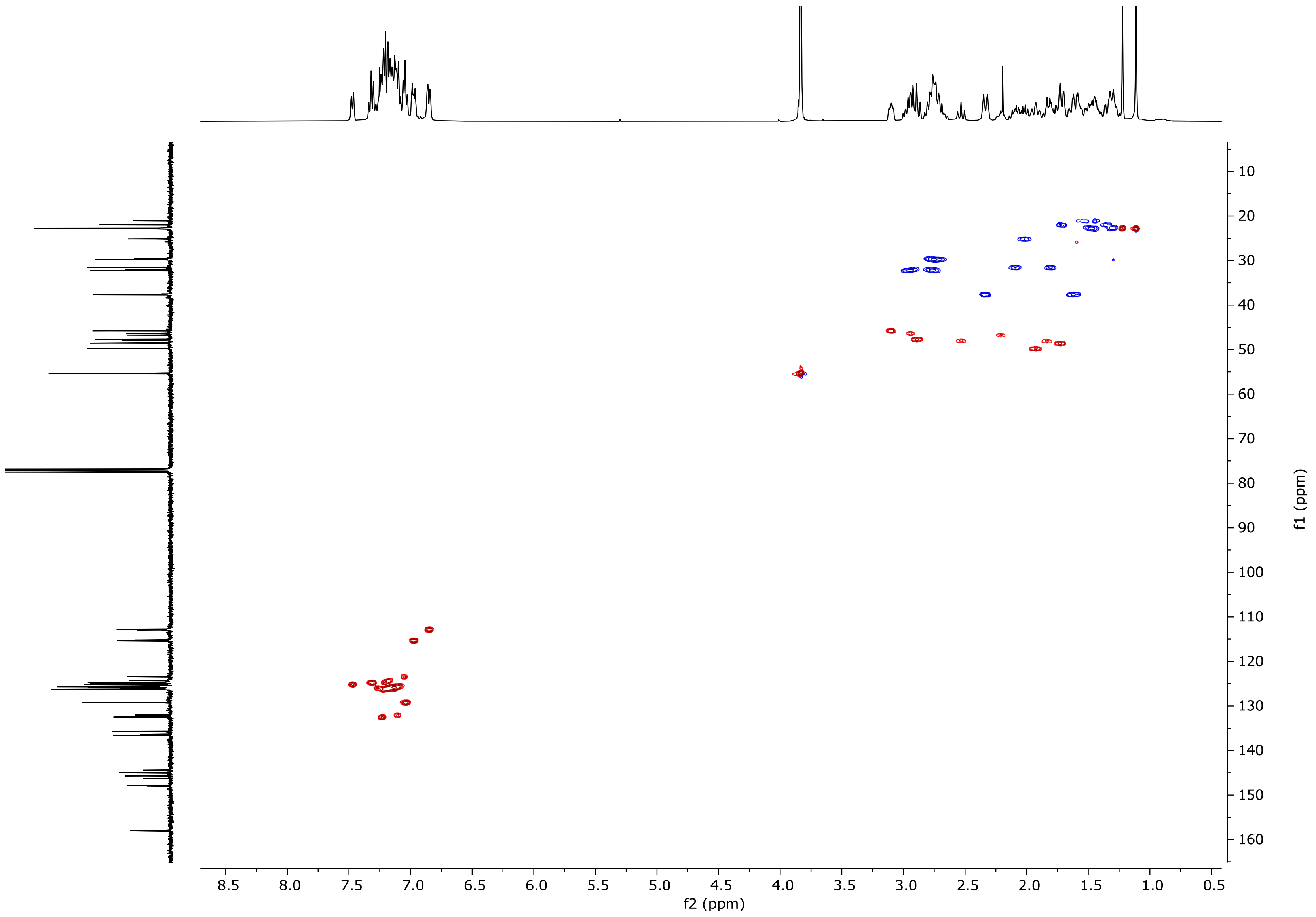

COSY-2D (400 MHz, $CDCl_3$)

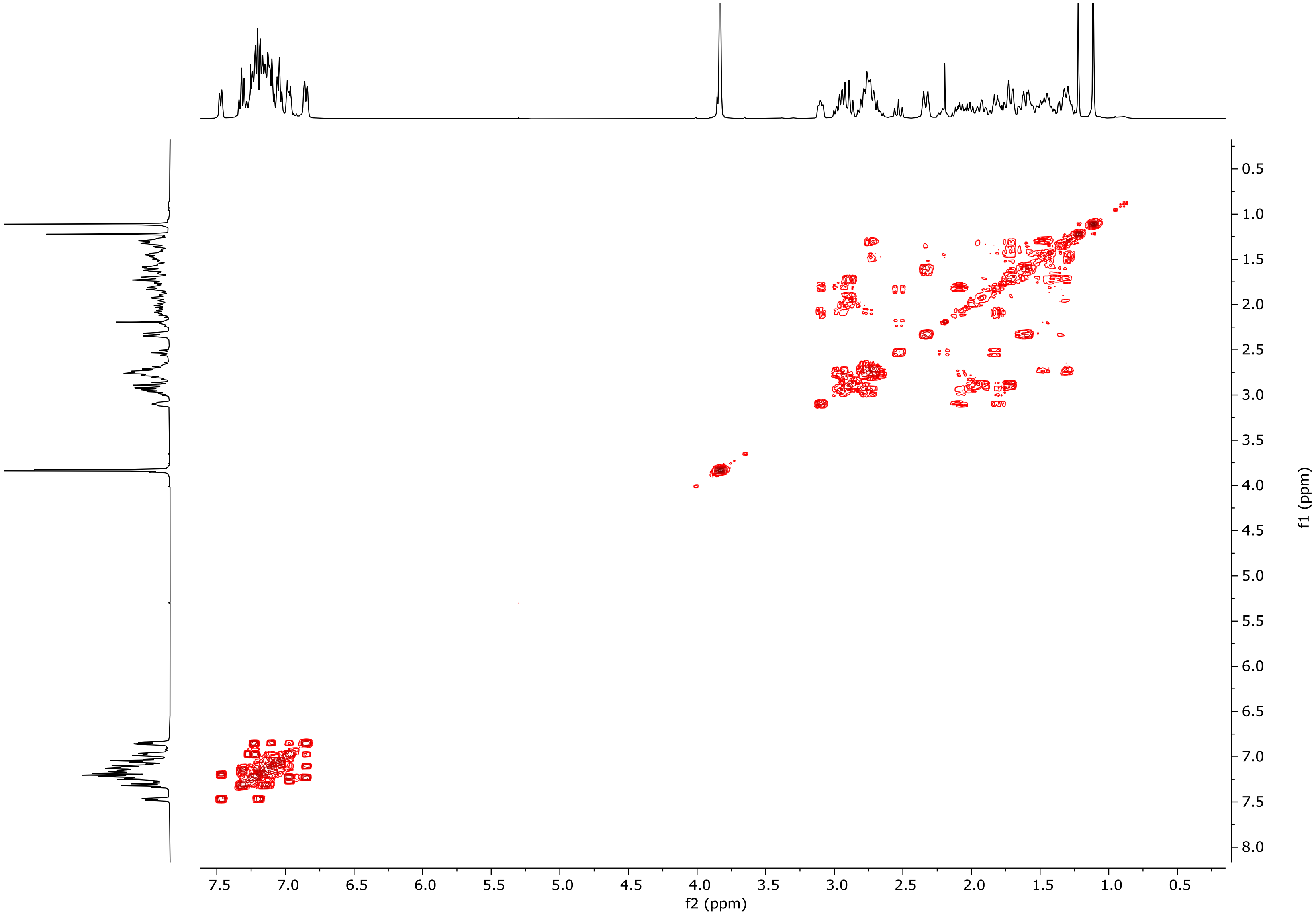

NOESY-2D (600 MHz, $CDCl_3$)

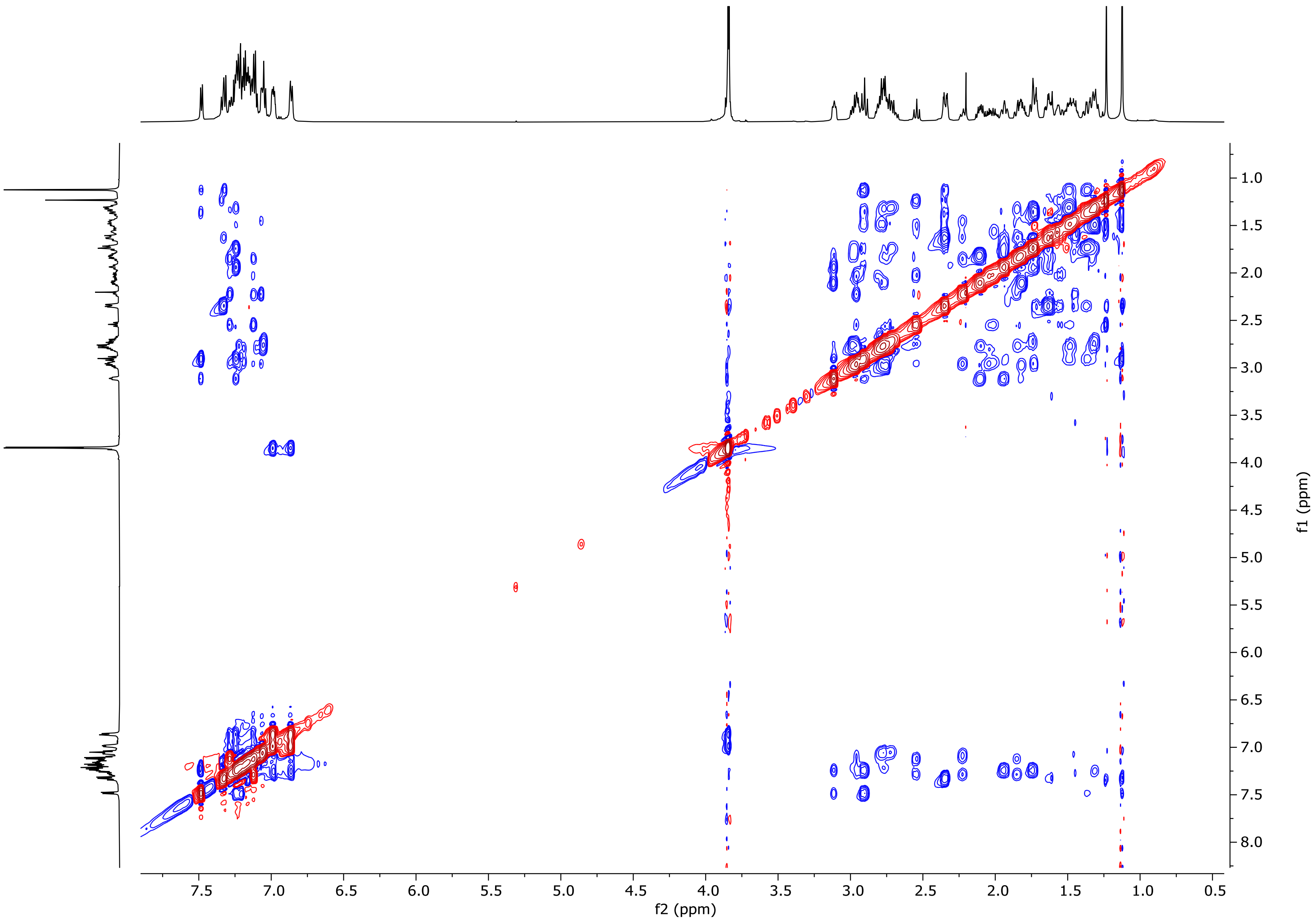

^{1}H (600 MHz)-^{13}C (151 MHz) **HMBC-2D** ($CDCl_3$)

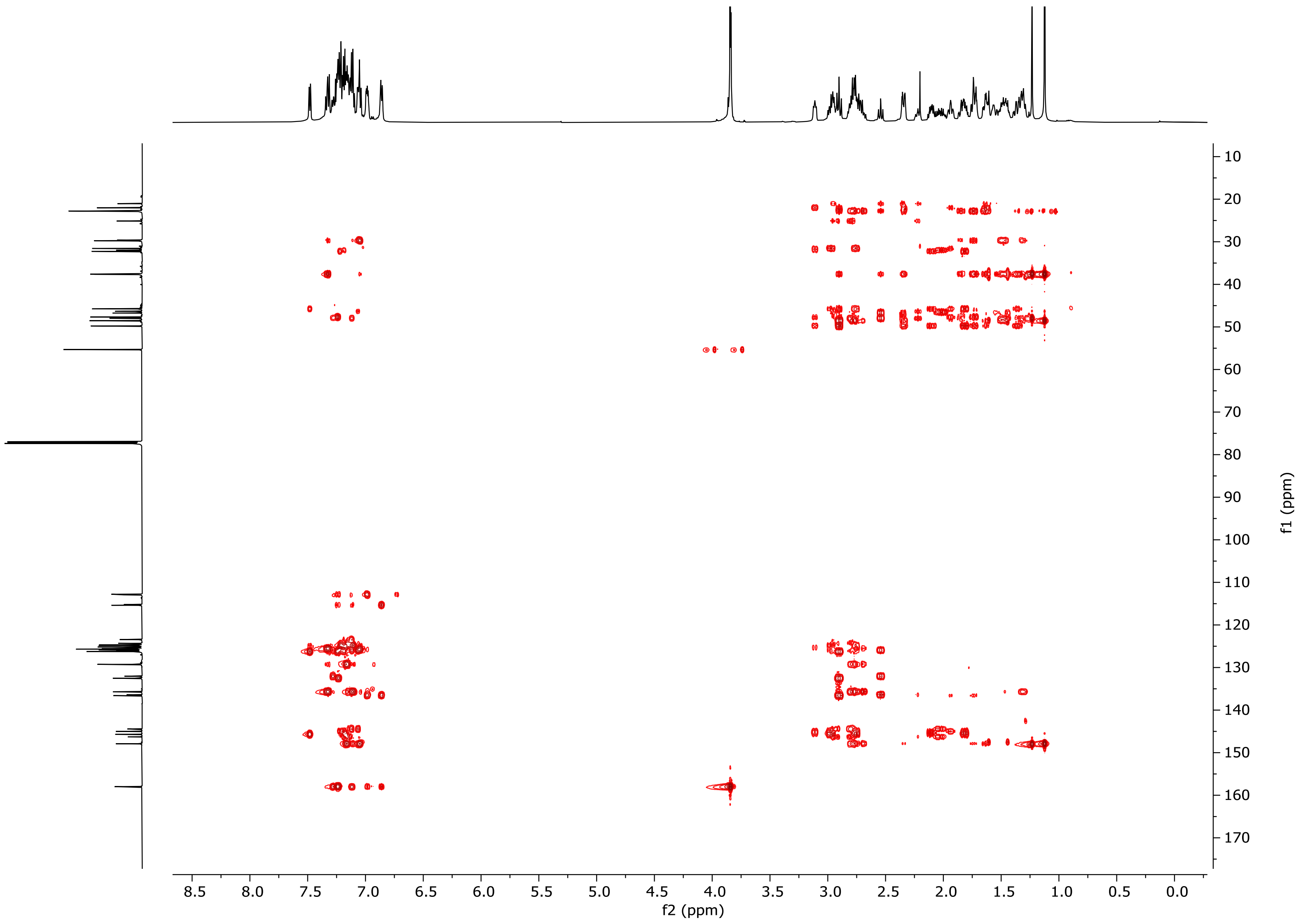

(±)-(1*R*,2*S*,4*aS*,10*aS*)-2-(Bis(4-chlorophenyl)methyl)-1-(4-methoxyphenyl)-4*a*-methyl-1,2,3,4,4*a*,9,10,10*a*-octahydrophenanthrene 2l.

^{1}H NMR (400 MHz, CD_2Cl_2)

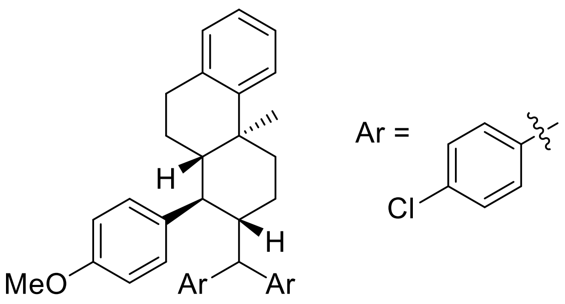

2l

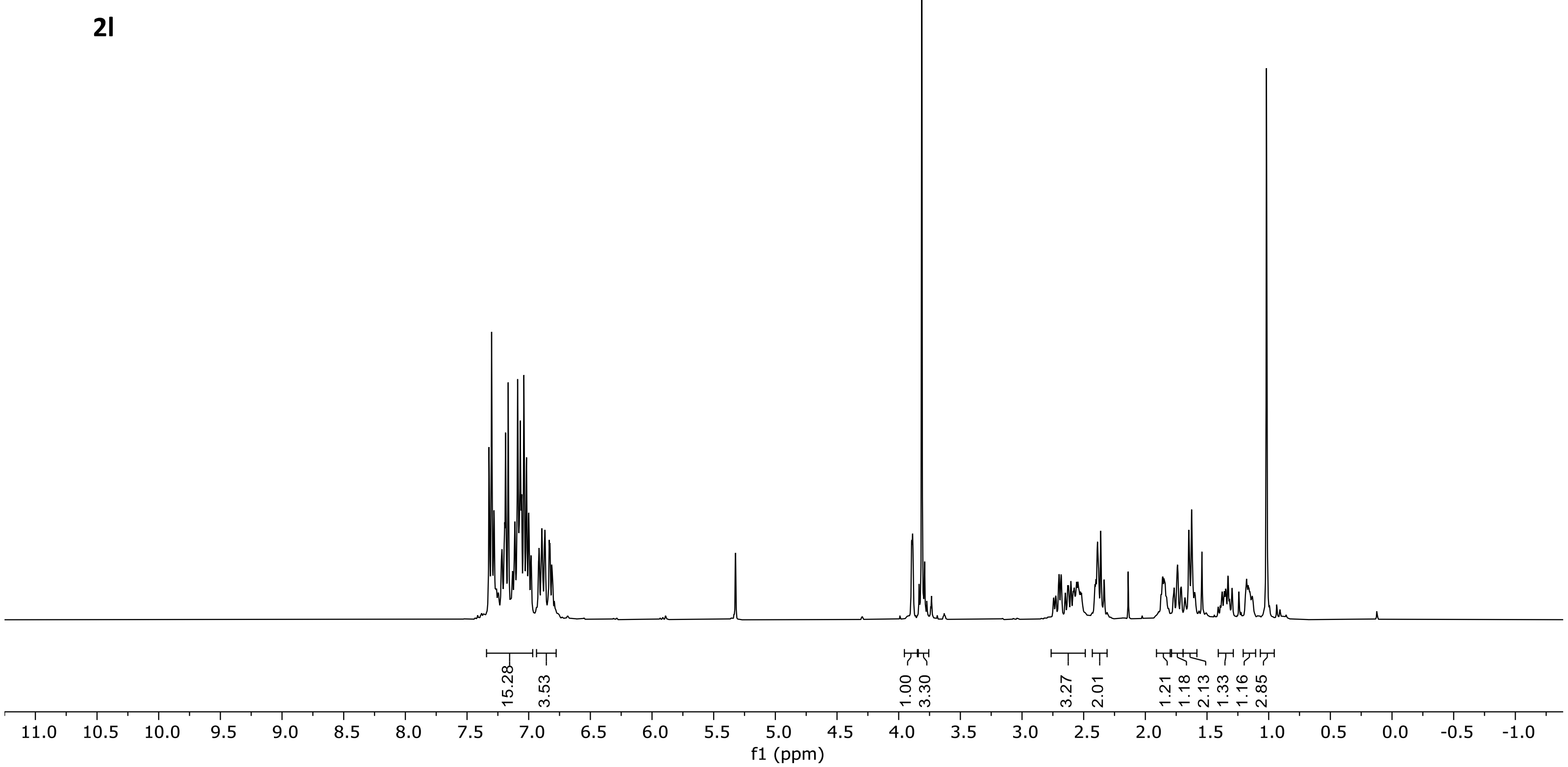

^{13}C NMR (101 MHz, CD_2Cl_2)

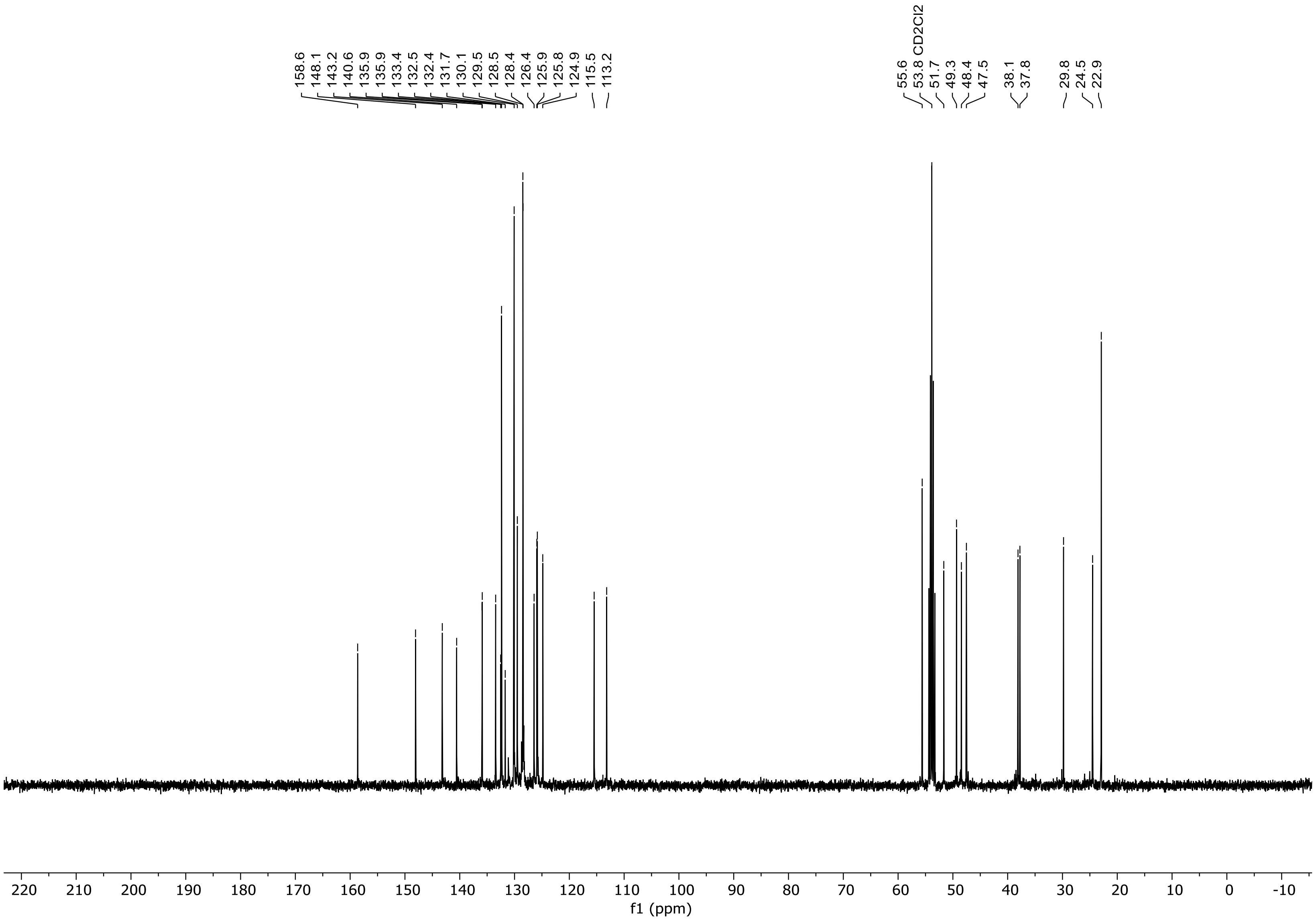

NOESY-2D (600 MHz, CD_2Cl_2)

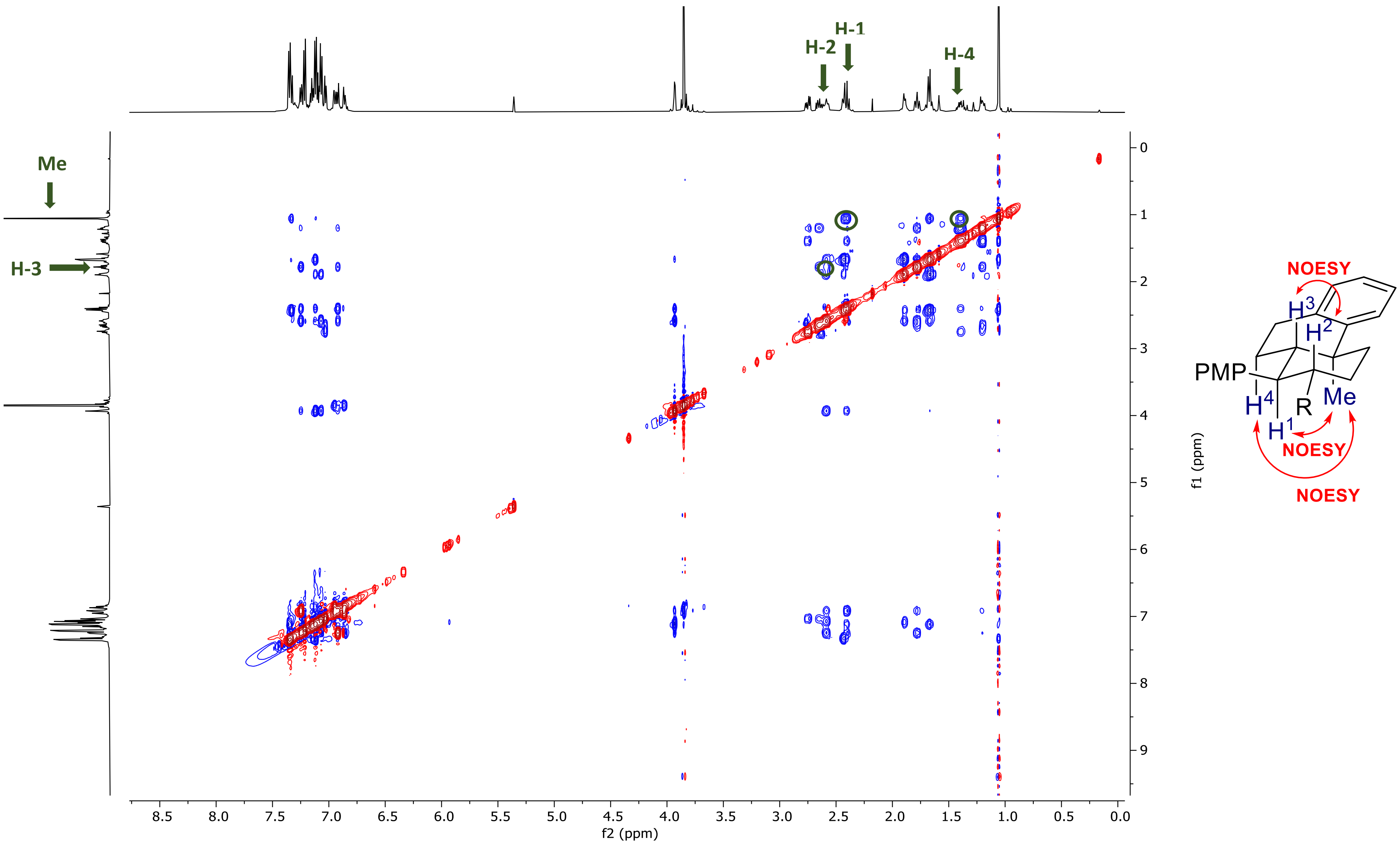

(±)-(1*R*,2*S*,4*aS*,10*aS*)-2-(Bis(4-methoxyphenyl)methyl)-1-(4-methoxyphenyl)-4*a*-methyl-1,2,3,4,4*a*,9,10,10*a*-octahydrophenanthrene 2m.

^{1}H NMR (400 MHz, $CDCl_3$)

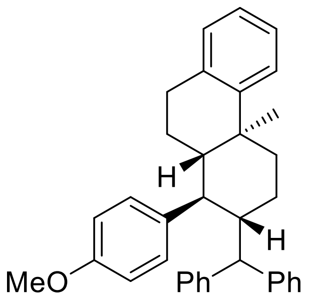

2m

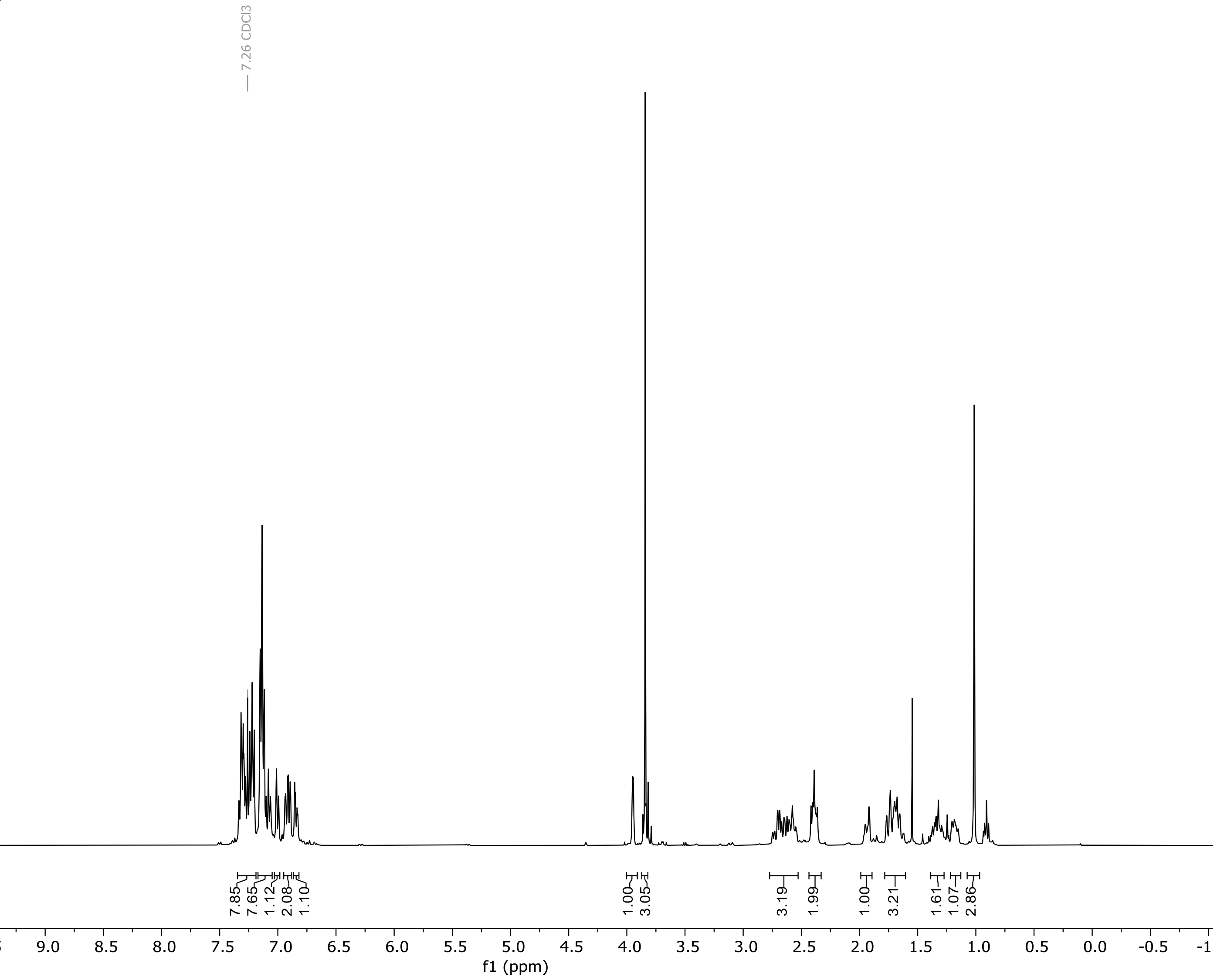

^{13}C NMR (101 MHz, $CDCl_3$)

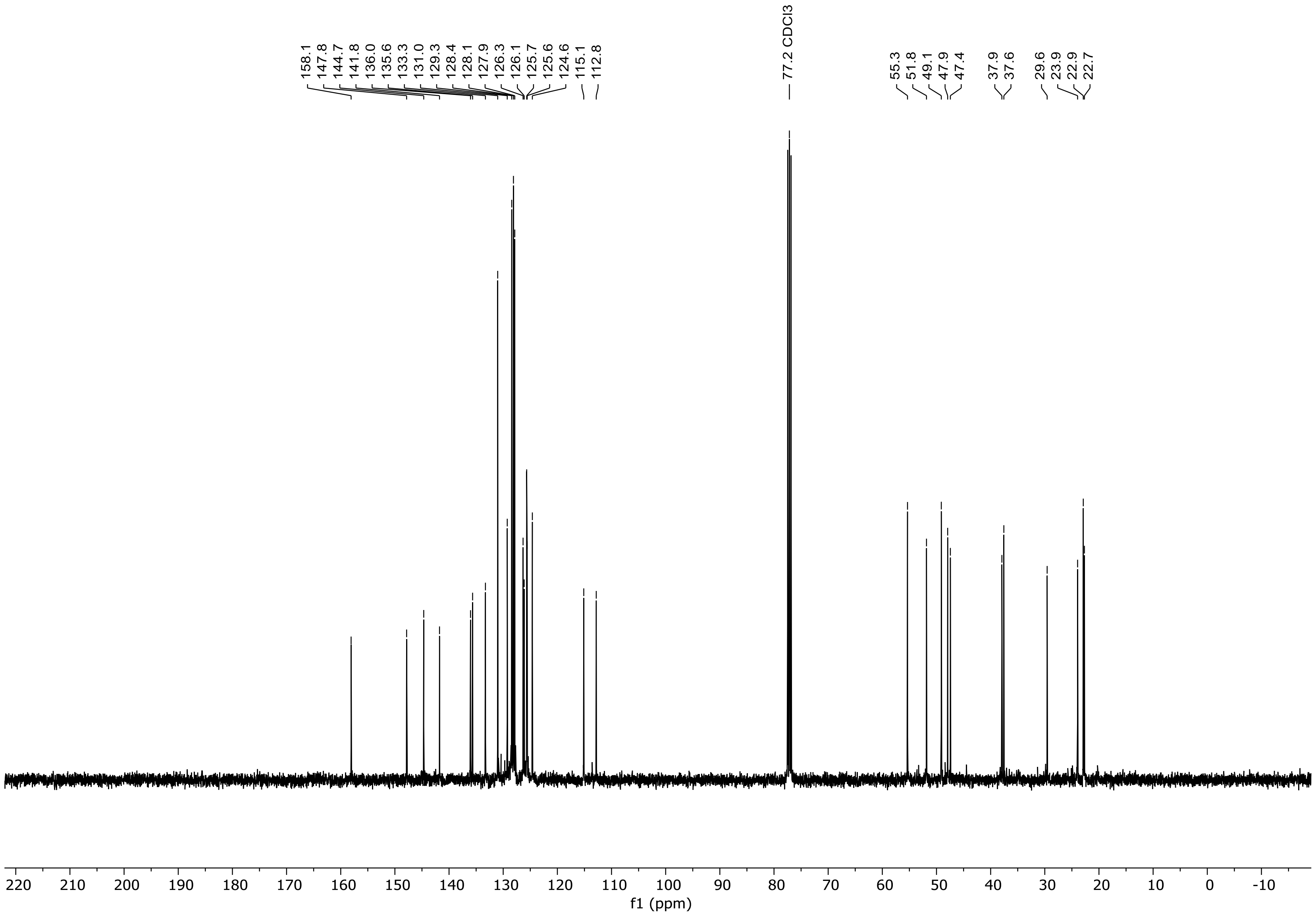

NOESY-2D (600 MHz, $CDCl_3$)

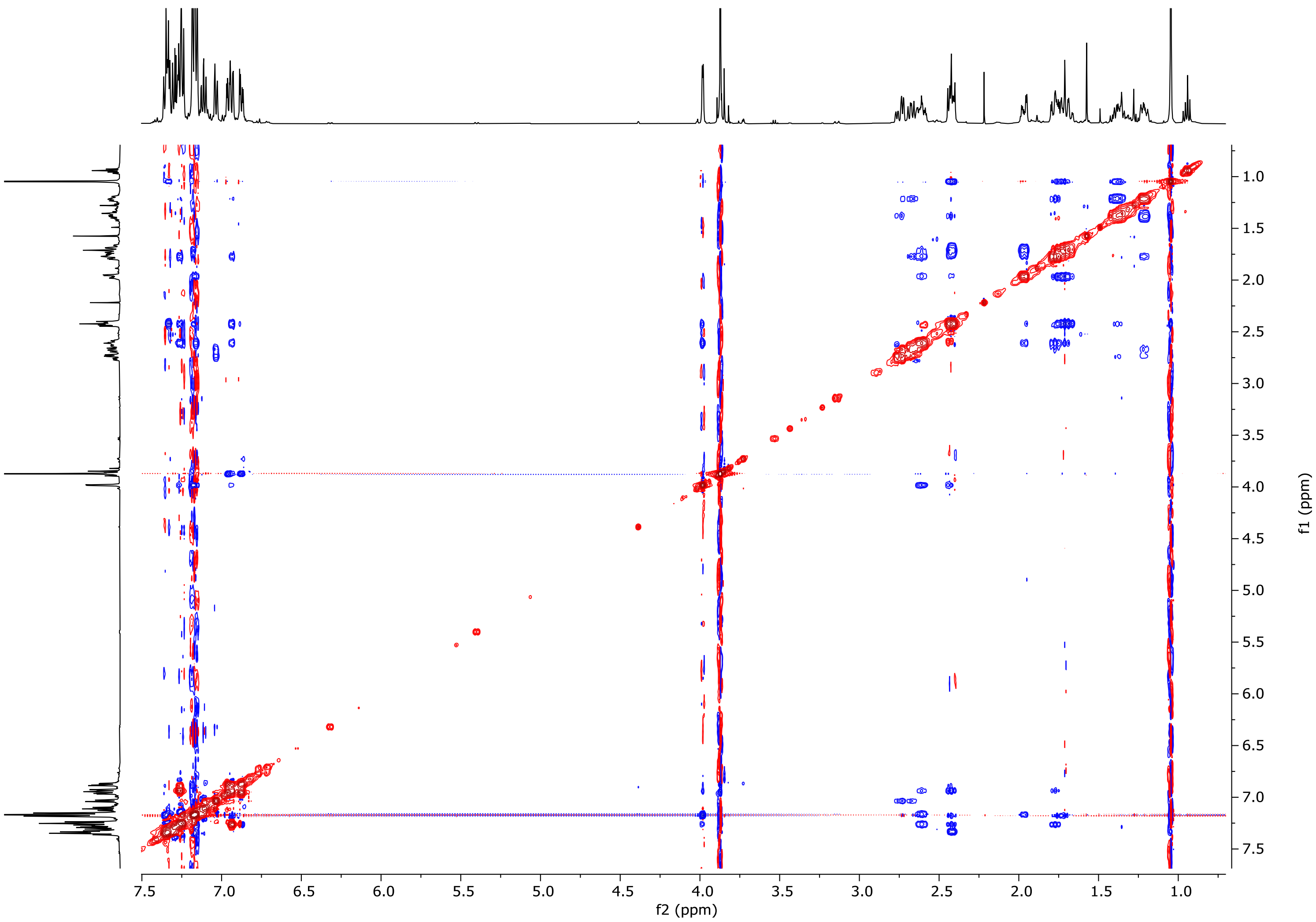

(±)-(1*R*,2*S*,4*aS*,10*aS*)-2-(Bis(4-methoxyphenyl)methyl)-1-(4-methoxyphenyl)-4*a*-methyl-1,2,3,4,4*a*,9,10,10*a*-octahydrophenanthrene 2n.

^{1}H NMR (400 MHz, $CDCl_3$)

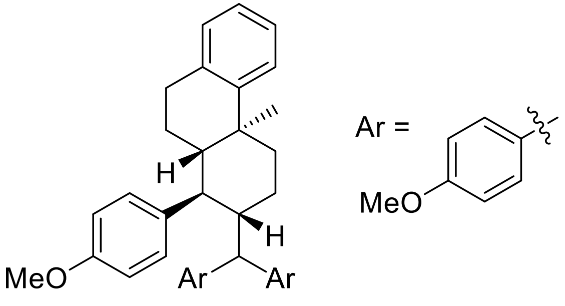

2n

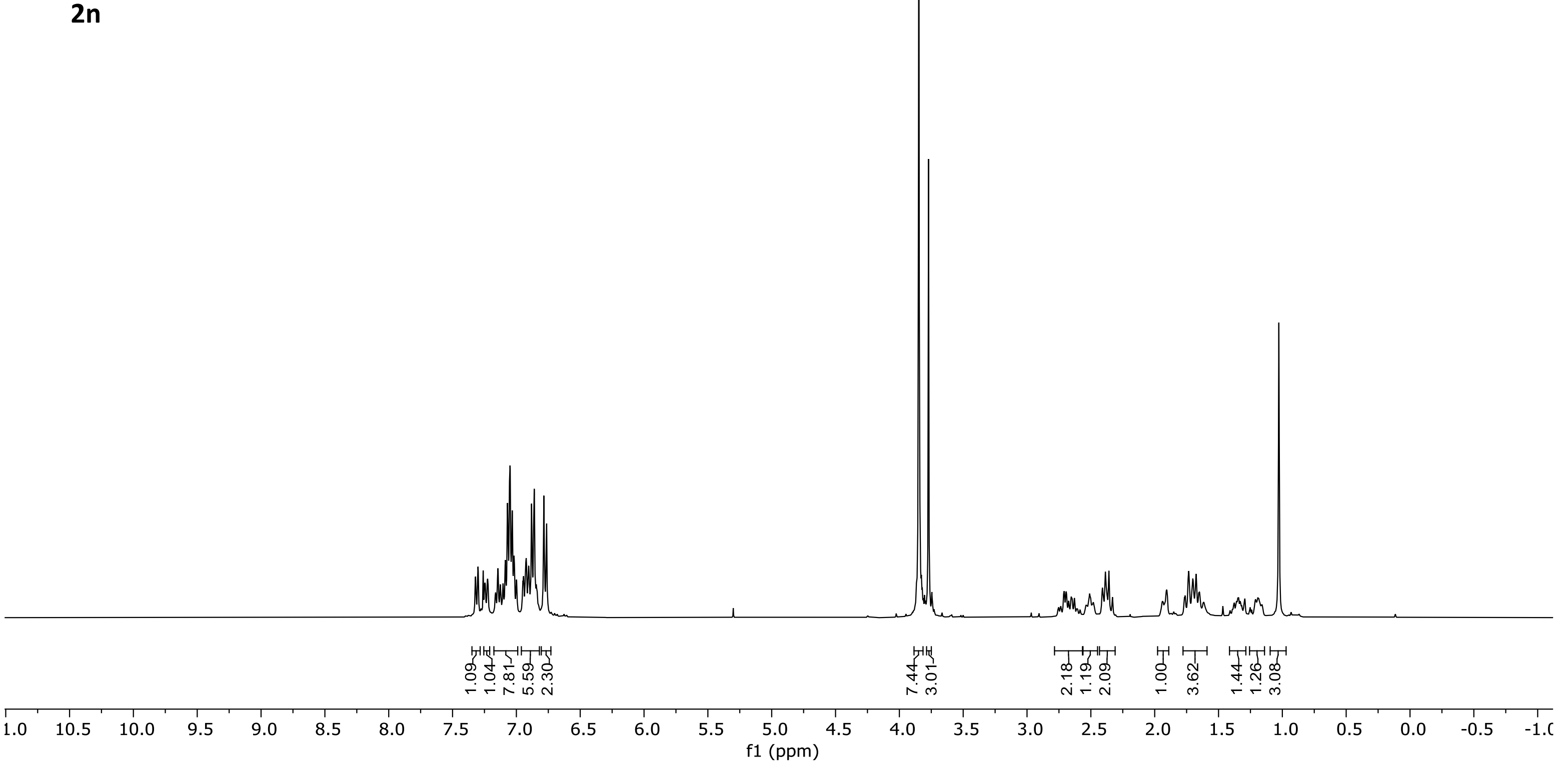

^{13}C NMR (101 MHz, $CDCl_3$)

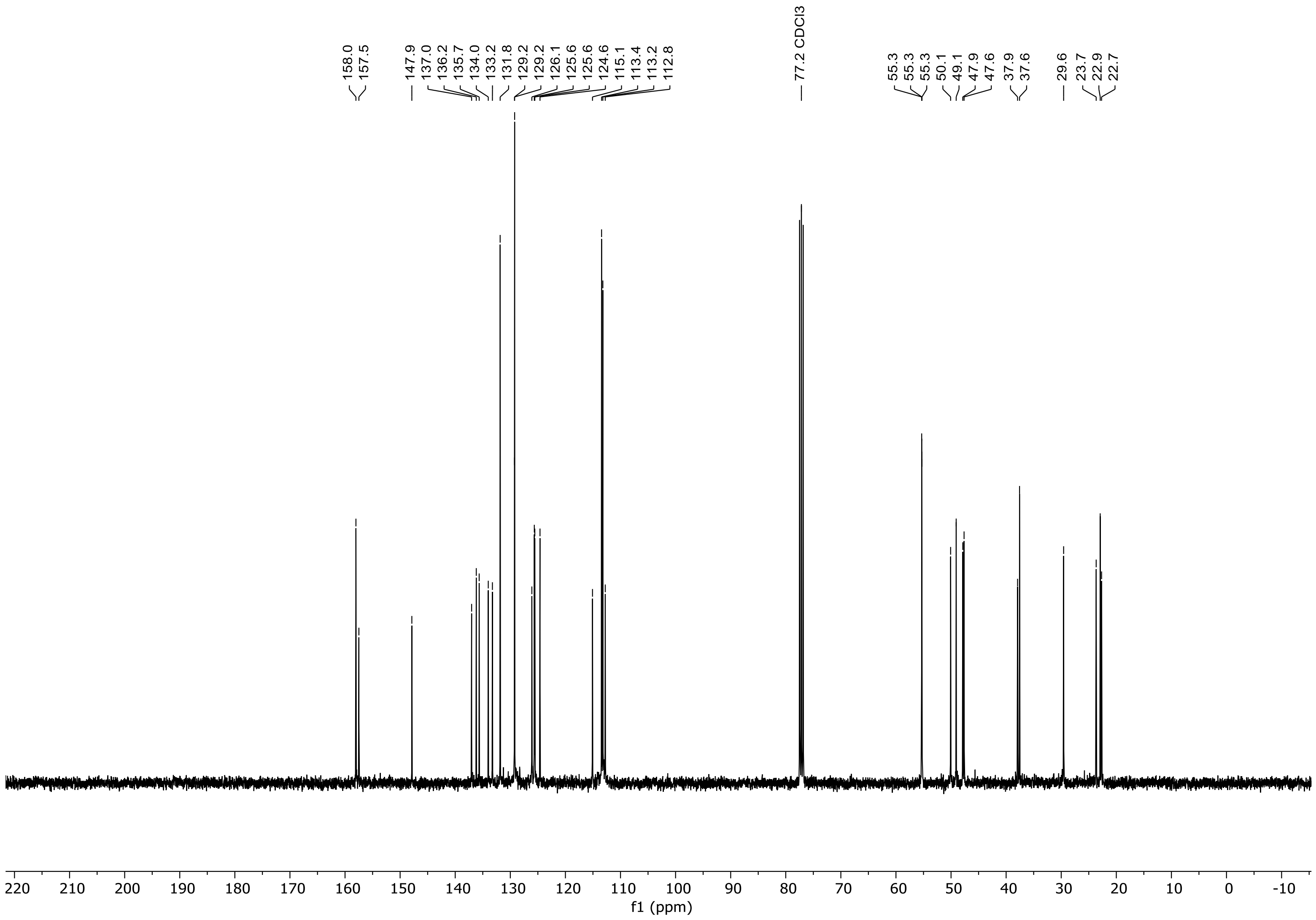

(±)-(1*R*,2*R*,4a*S*,10a*S*)-1-(4-Methoxyphenyl)-4a-methyl-2-(3-phenylcyclopent-2-en-1-yl)-1,2,3,4,4a,9,10,10a-octahydrophenanthrene 2o.

^{1}H NMR (400 MHz, $CDCl_3$)

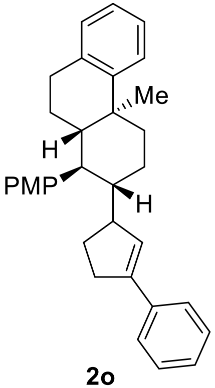

2o

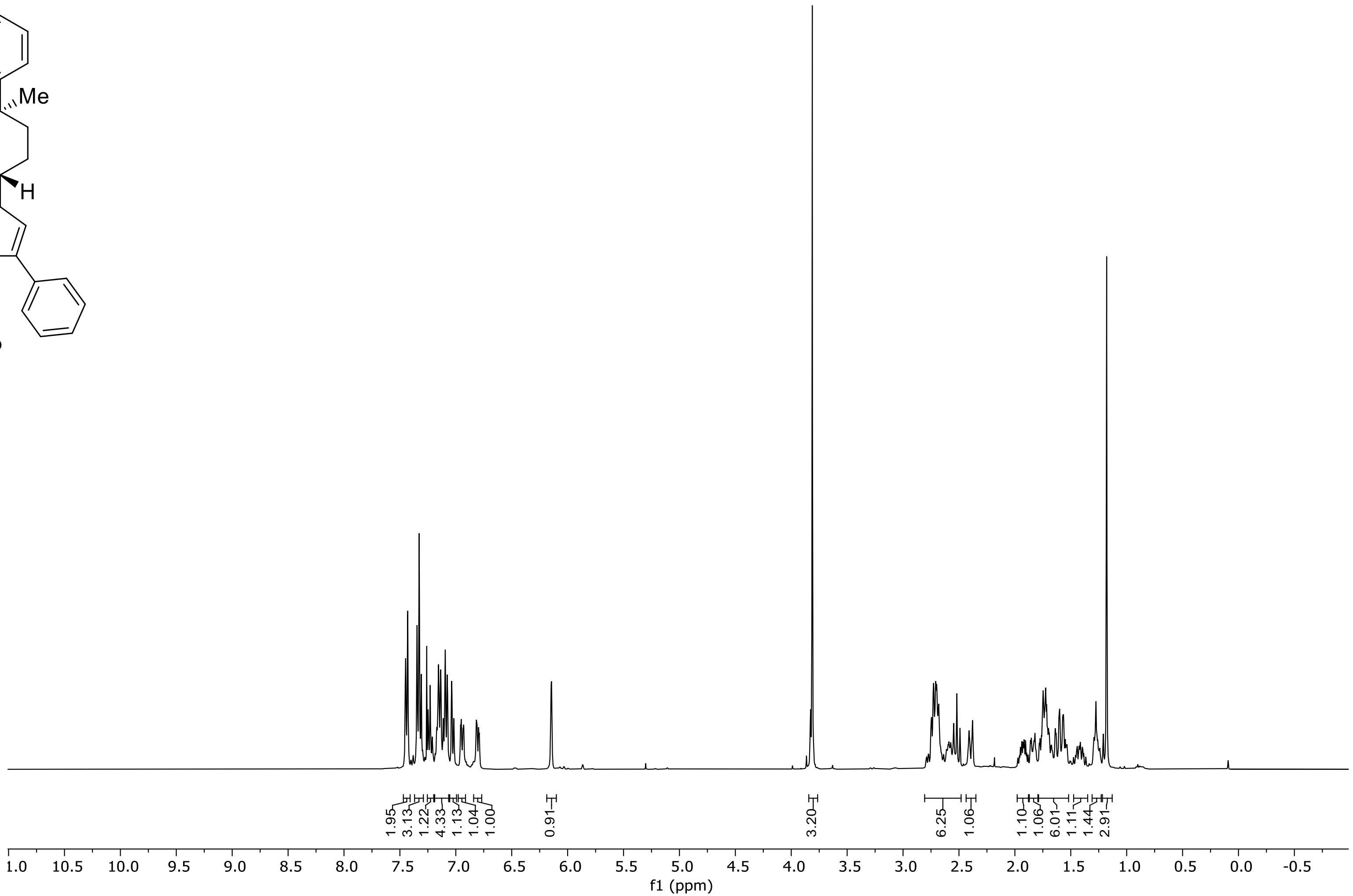

^{13}C NMR (101 MHz, $CDCl_3$)

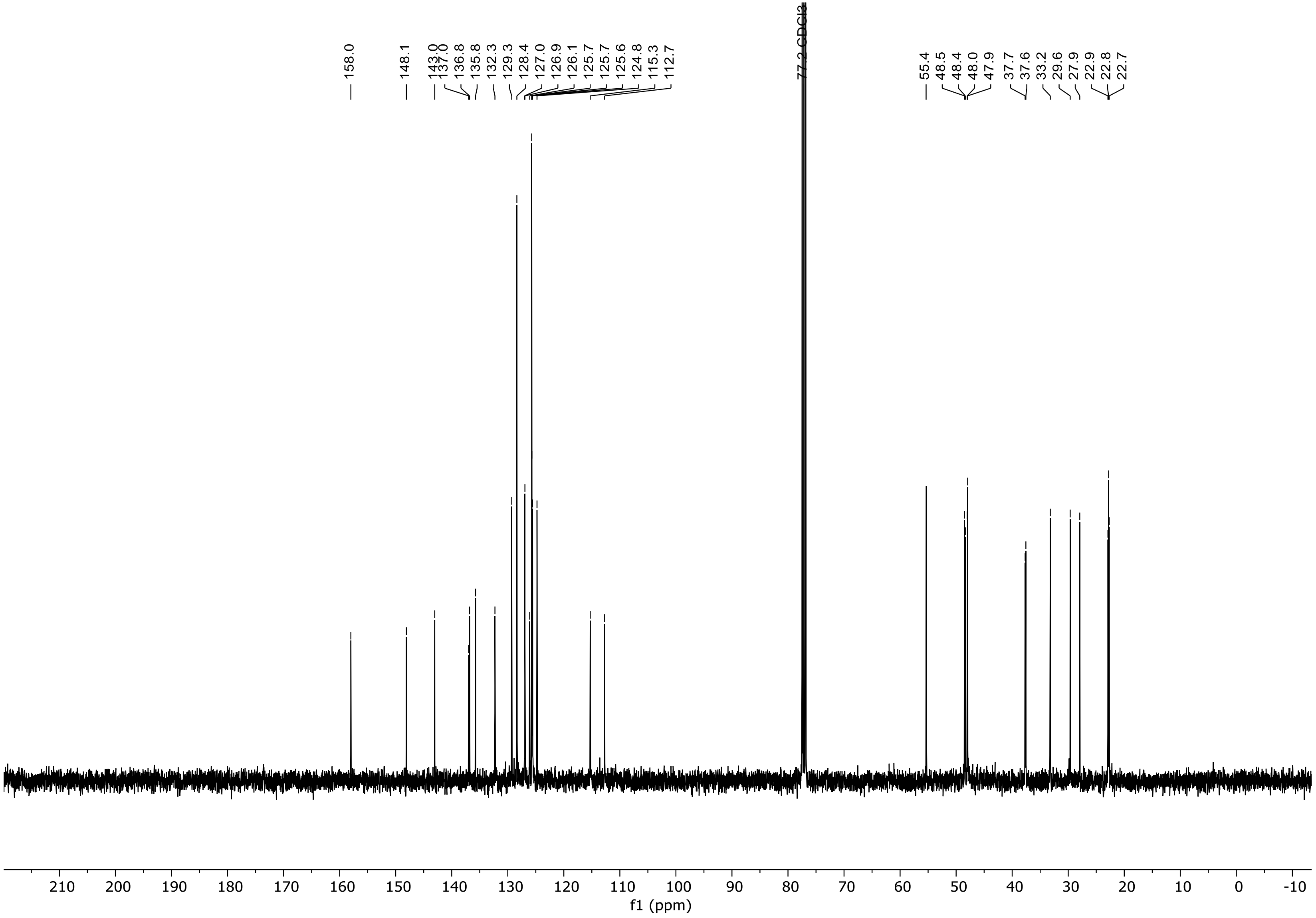

^{1}**H** (400 MHz)-^{13}**C** (101 MHz) **HSQC-2D** (CDCl$_3$)

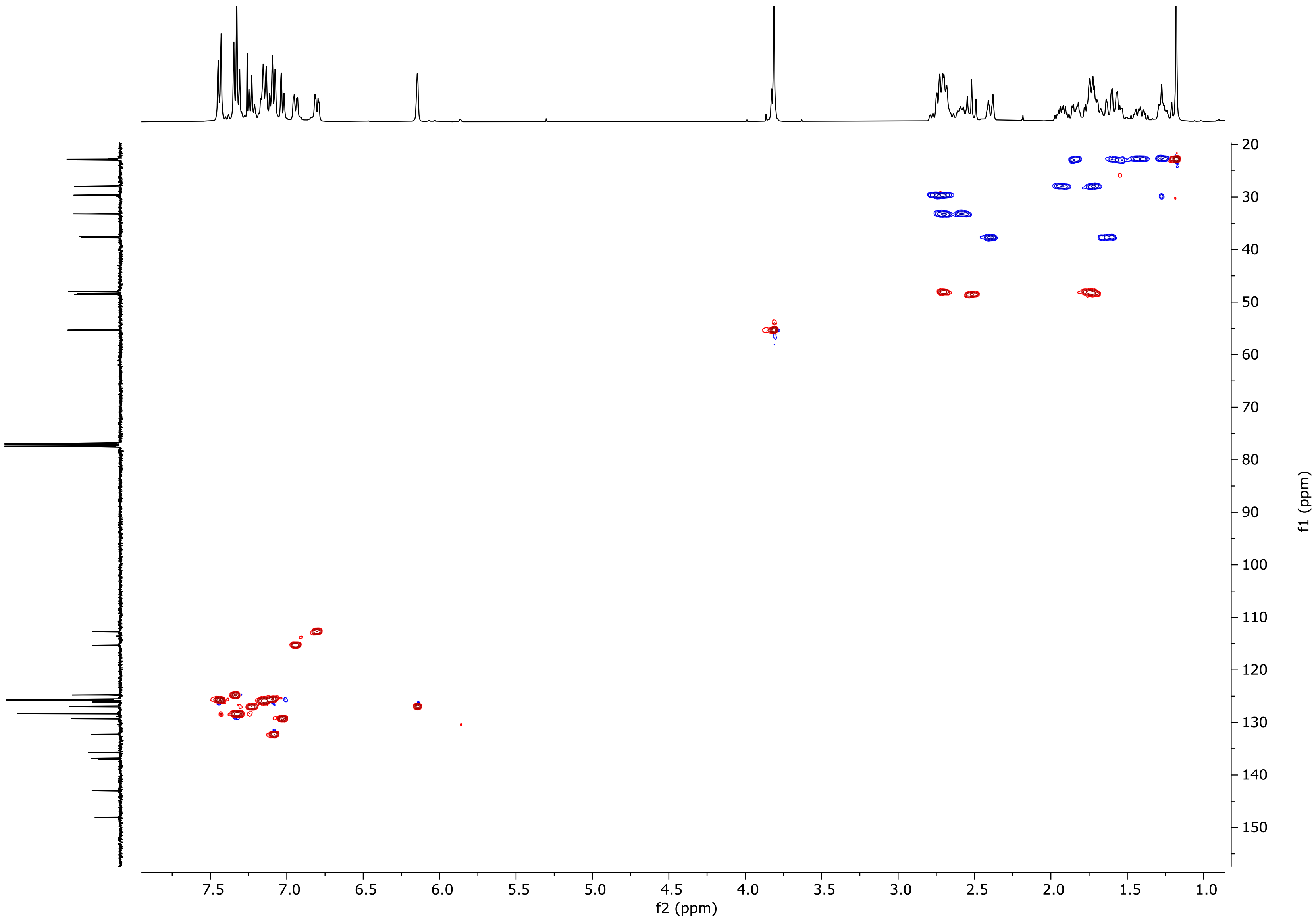

NOESY-2D (600 MHz, $CDCl_3$)

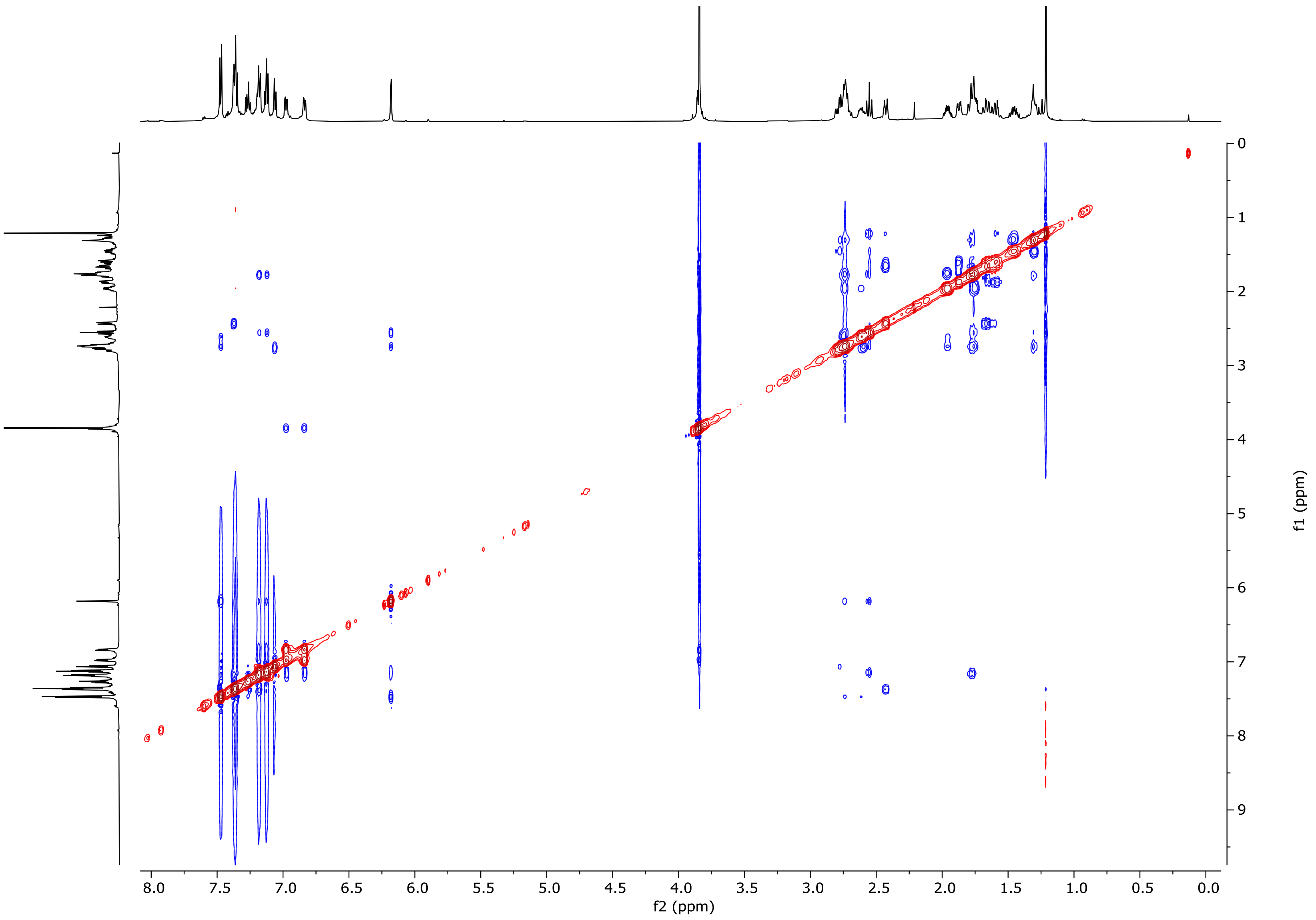

(±)-(1*R*,2*R*,4*aS*,10*aS*)-2-((*S,E*)-1,3-Diphenylallyl)-1-(4-methoxyphenyl)-4*a*-methyl-1,2,3,4,4*a*,9,10,10*a*-octahydrophenanthrene (majo*r*)-2p and (±)-(1*R*,2*R*,4*aS*,10*aS*)-2-((*R,E*)-1,3-diphenylallyl)-1-(4-methoxyphenyl)-4*a*-methyl-1,2,3,4,4*a*,9,10,10*a*-octahydrophenanthrene (mino*r*)-2p.

^{1}H NMR (600 MHz, $CDCl_3$)

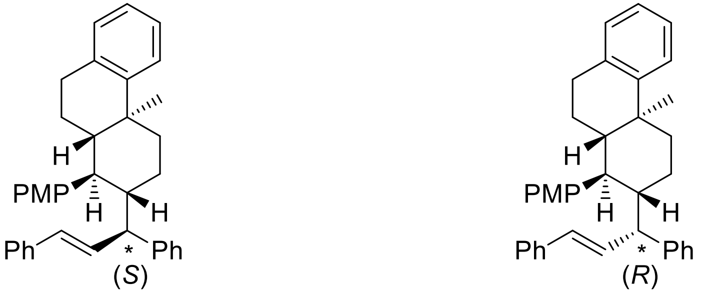

2p (major) **2p** (minor)

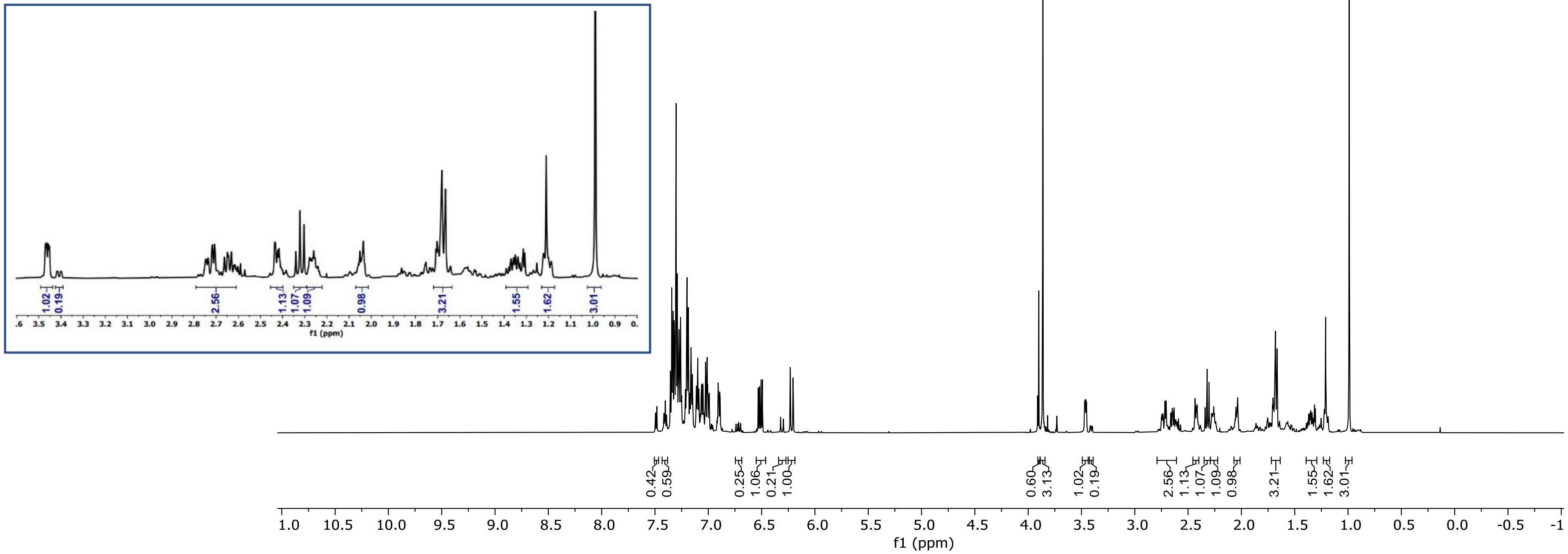

^{13}C NMR (101 MHz, $CDCl_3$)

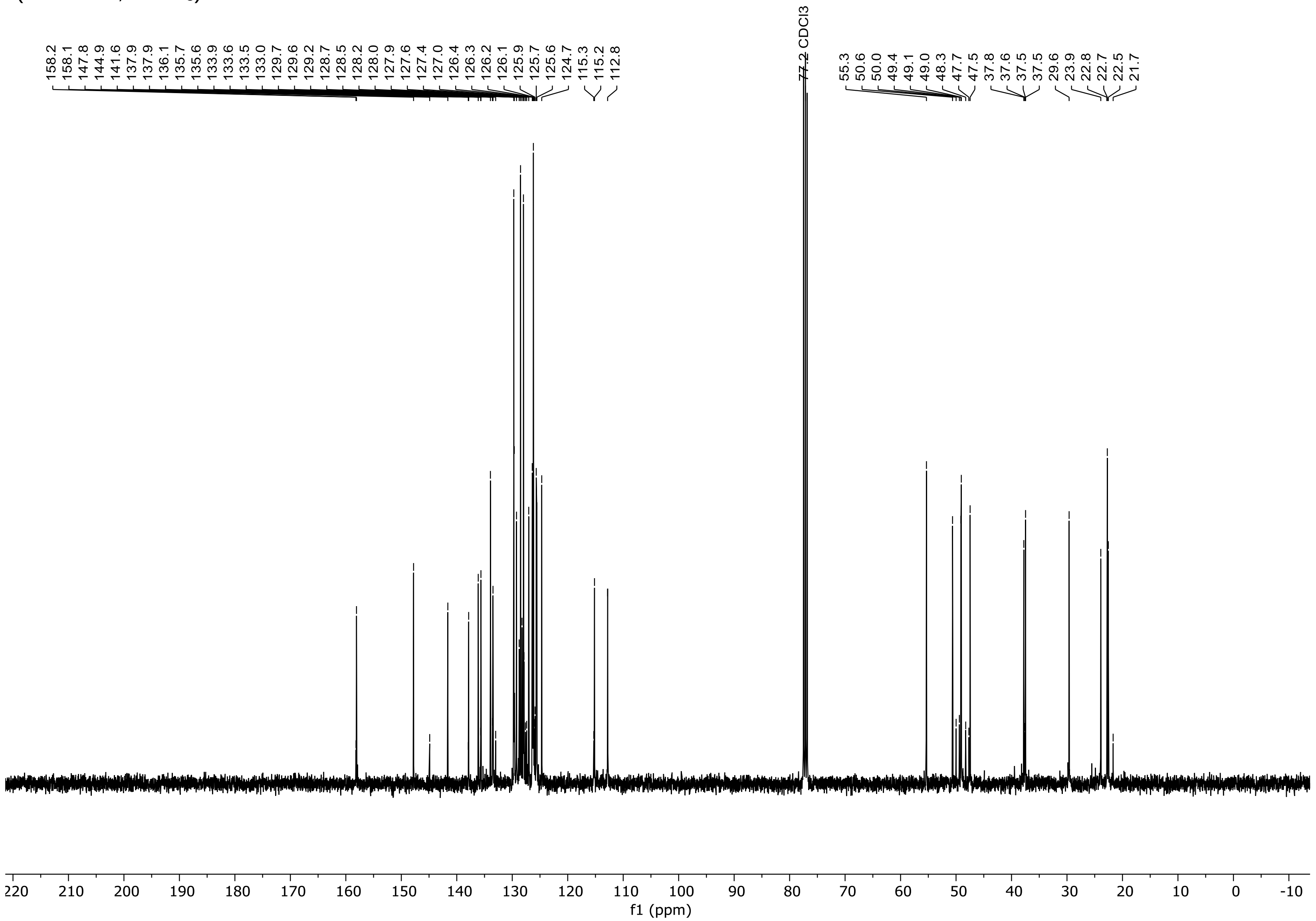

^{1}**H** (400 MHz)-^{13}**C** (101 MHz) **HSQC-2D** ($CDCl_3$)

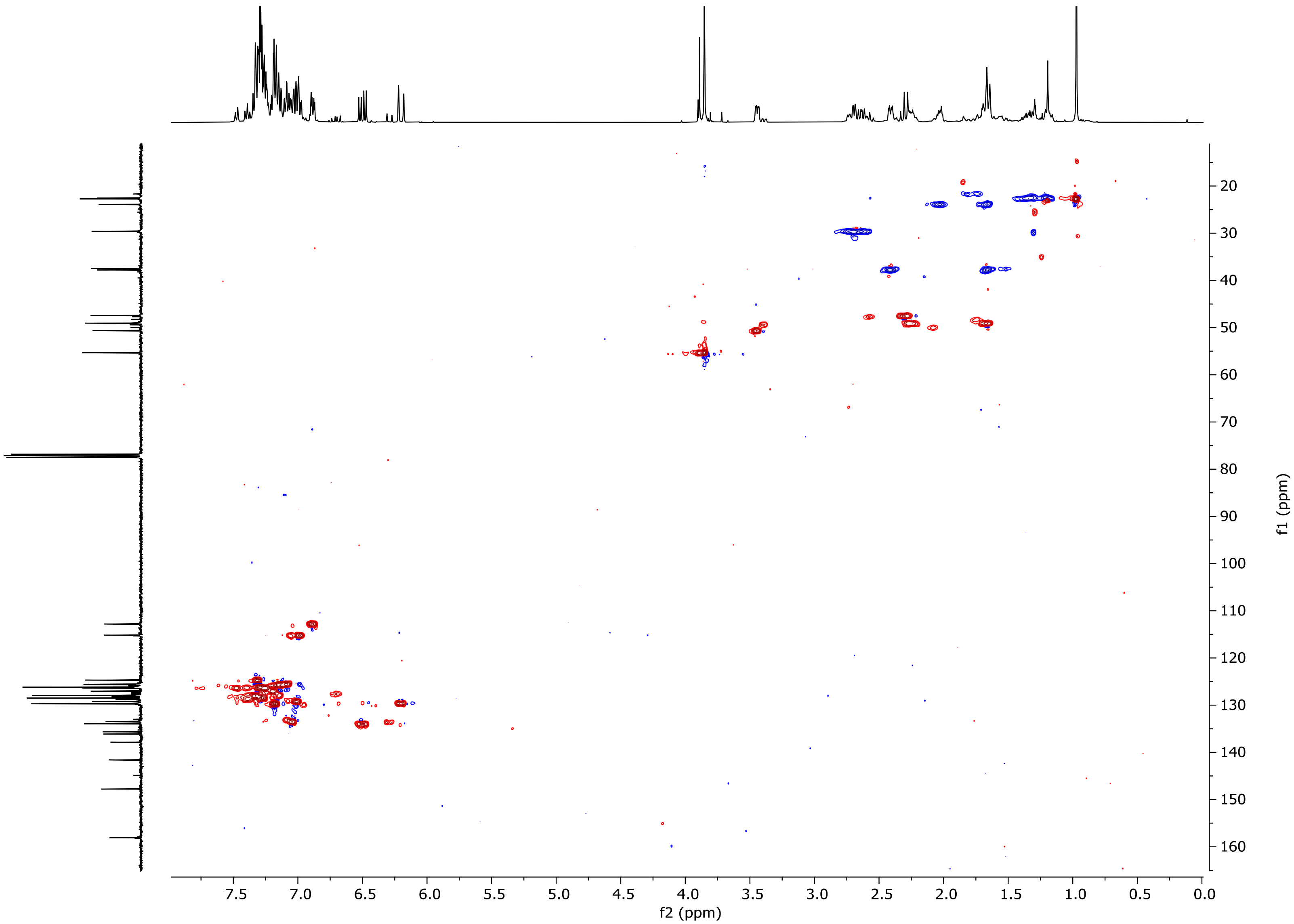

COSY-2D (400 MHz, $CDCl_3$)

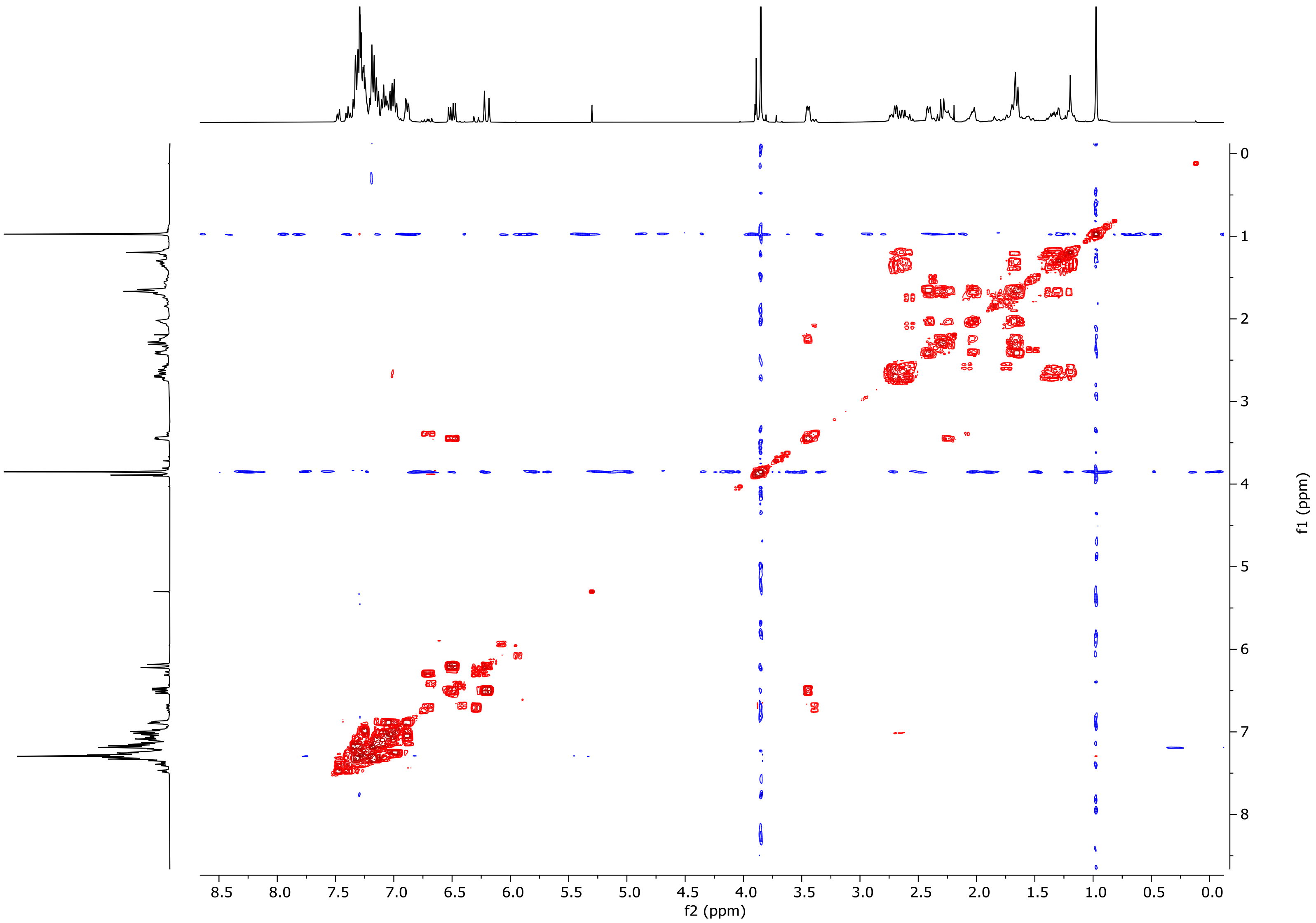

NOESY-2D (600 MHz, $CDCl_3$)

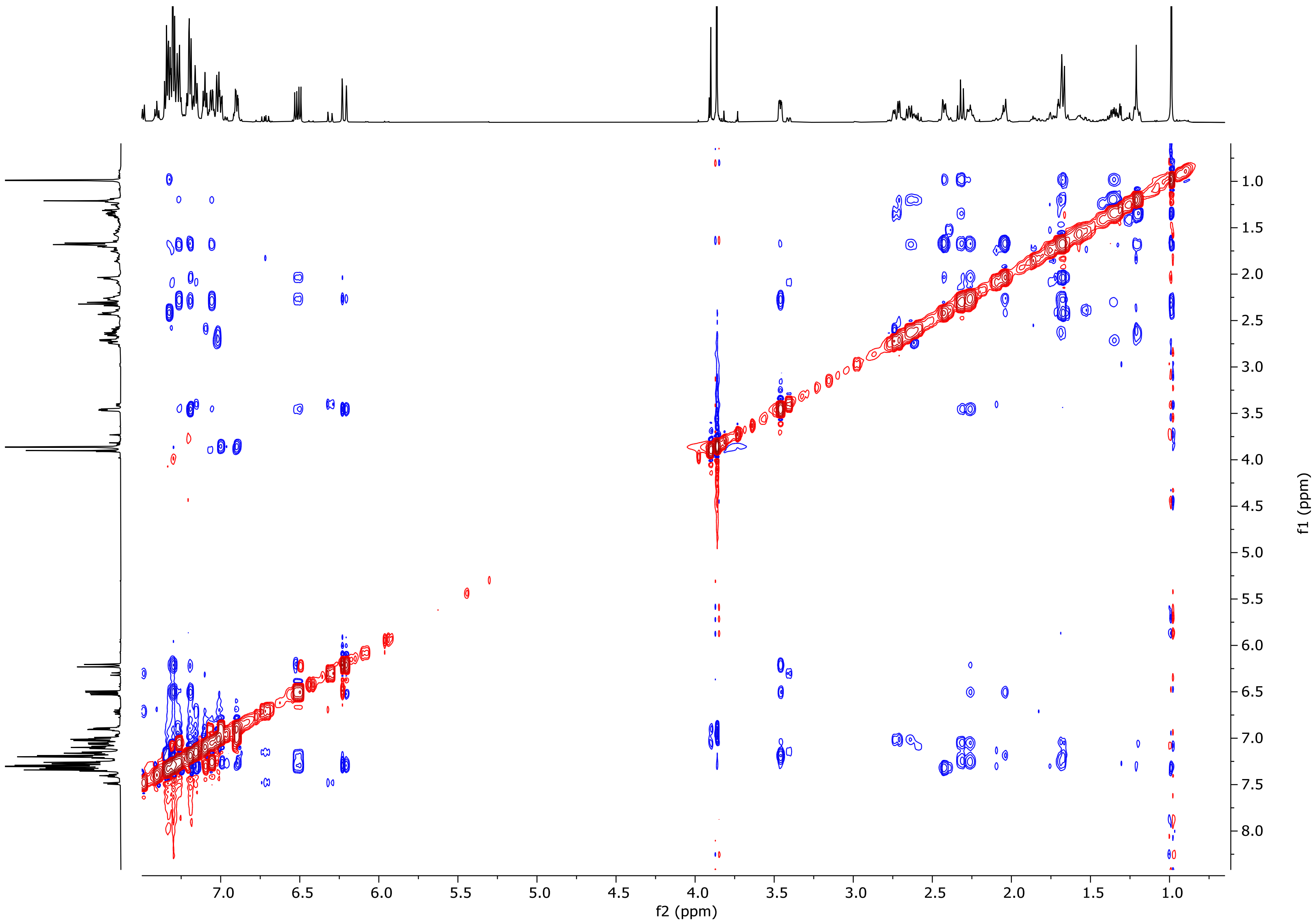

^{1}H (600 MHz)-^{13}C (151 MHz) **HMBC-2D** ($CDCl_3$)

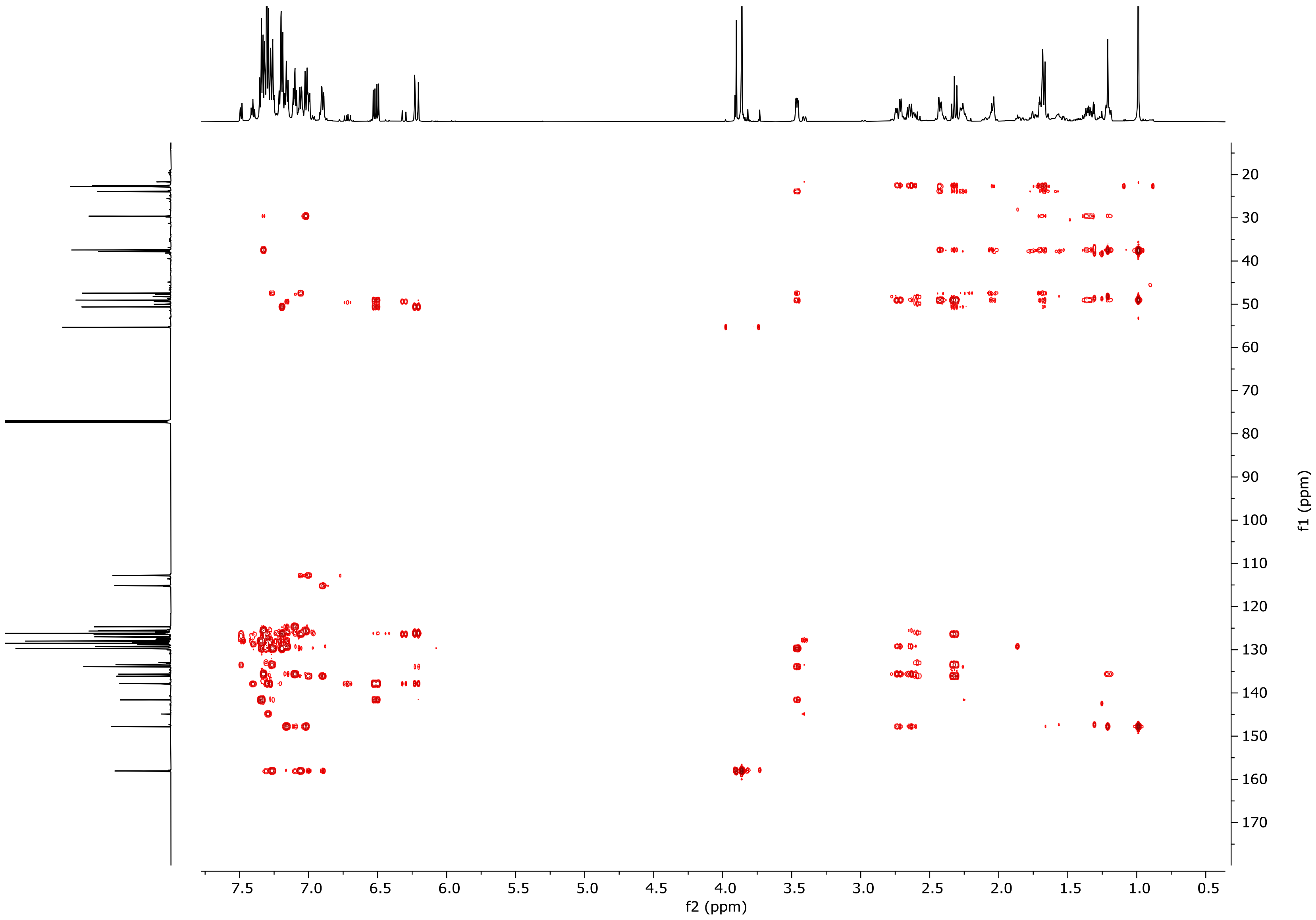

(±)-(1*R*,2*R*,4*aS*,10*aS*)-1-(4-Methoxyphenyl)-2-((*E*)-3-(4-methoxyphenyl)allyl)-4*a*-methyl-1,2,3,4,4*a*,9,10,10*a*-octahydrophenanthrene 2q.

^{1}H NMR (400 MHz, $CDCl_3$)

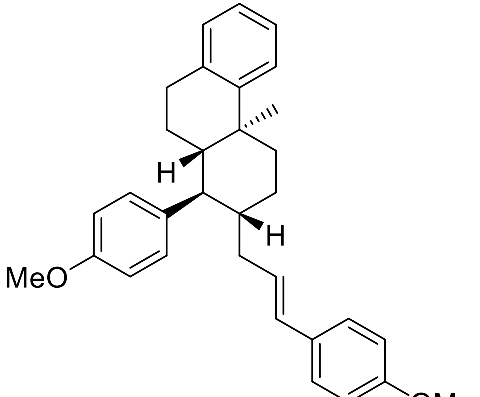

2q

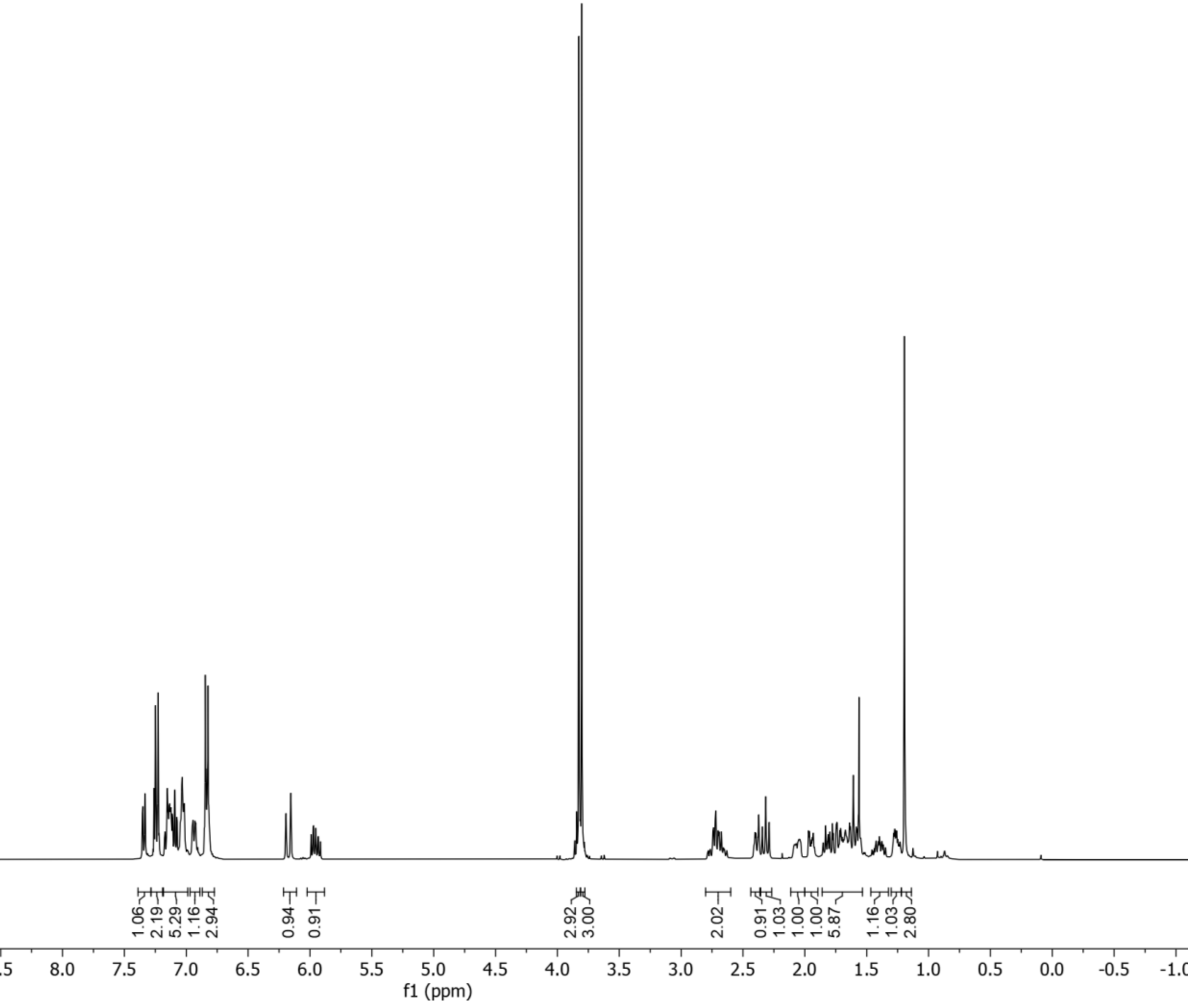

^{13}C NMR (101 MHz, $CDCl_3$)

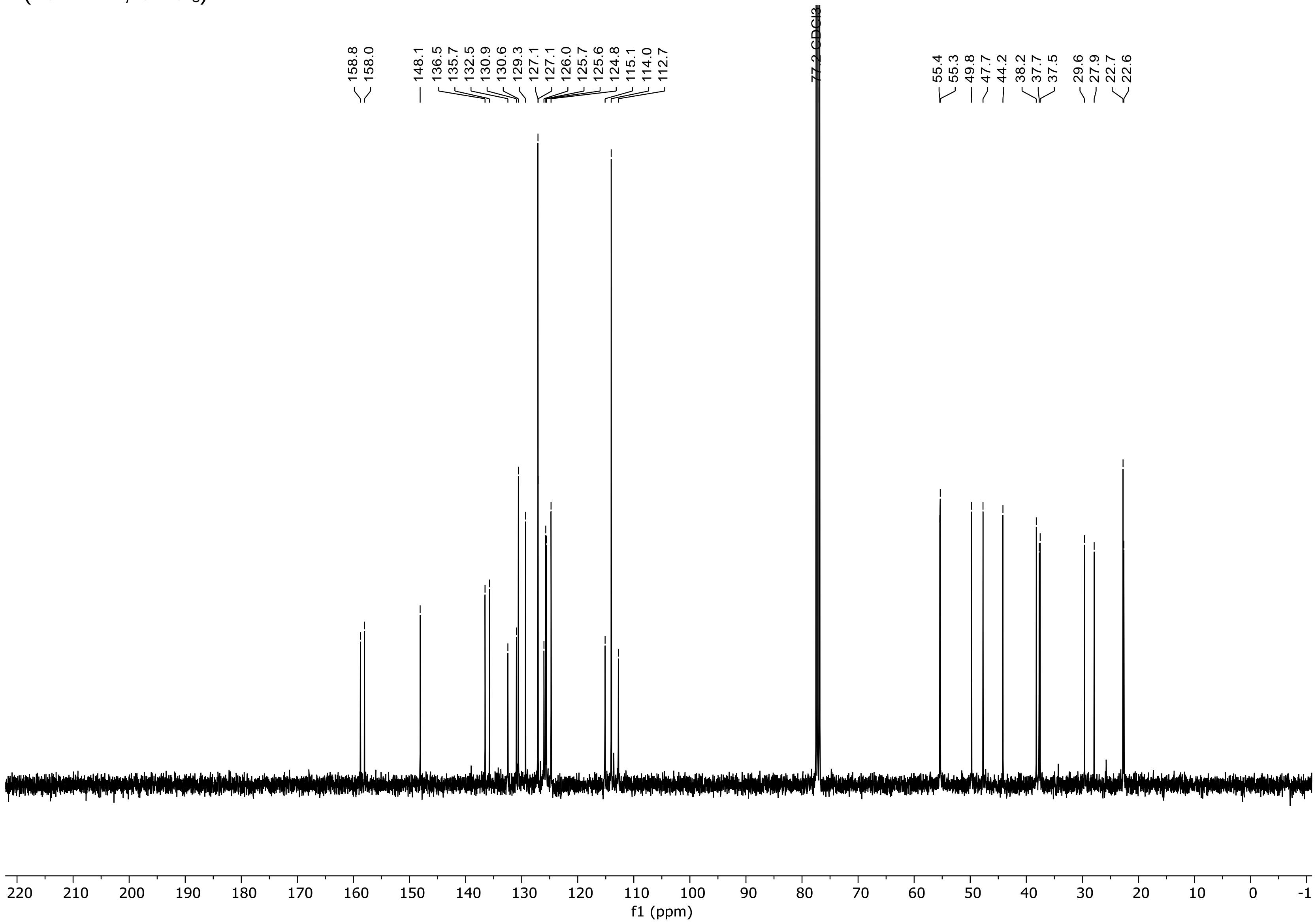

NOESY-2D (600 MHz, $CDCl_3$)

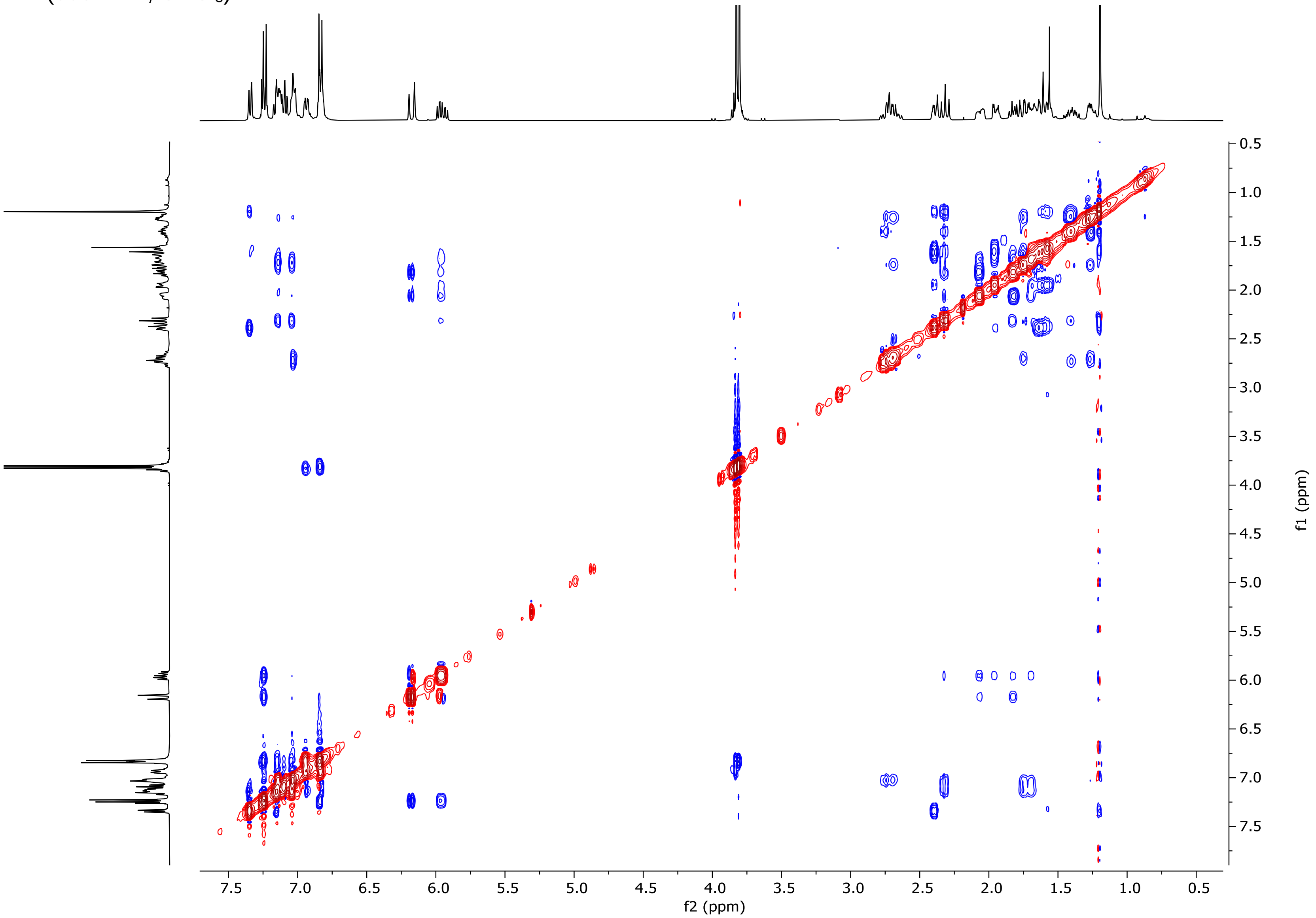

(±)-4-((*E*)-3-((1*R*,2*R*,4*aS*,10*aS*)-1-(4-methoxyphenyl)-4*a*-methyl-1,2,3,4,4*a*,9,10,10*a*-octahydrophenanthren-2-yl)prop-1-en-1-yl)phenol 2r.
^{1}H NMR (400 MHz, CD_2Cl_2)

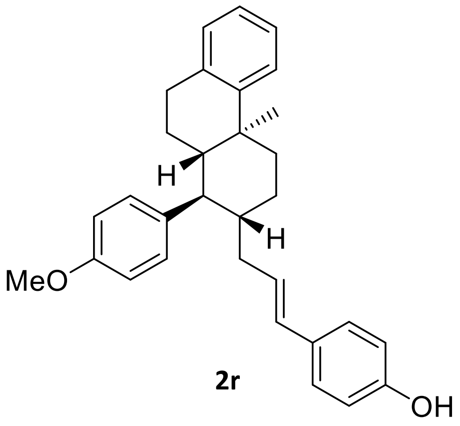

2r

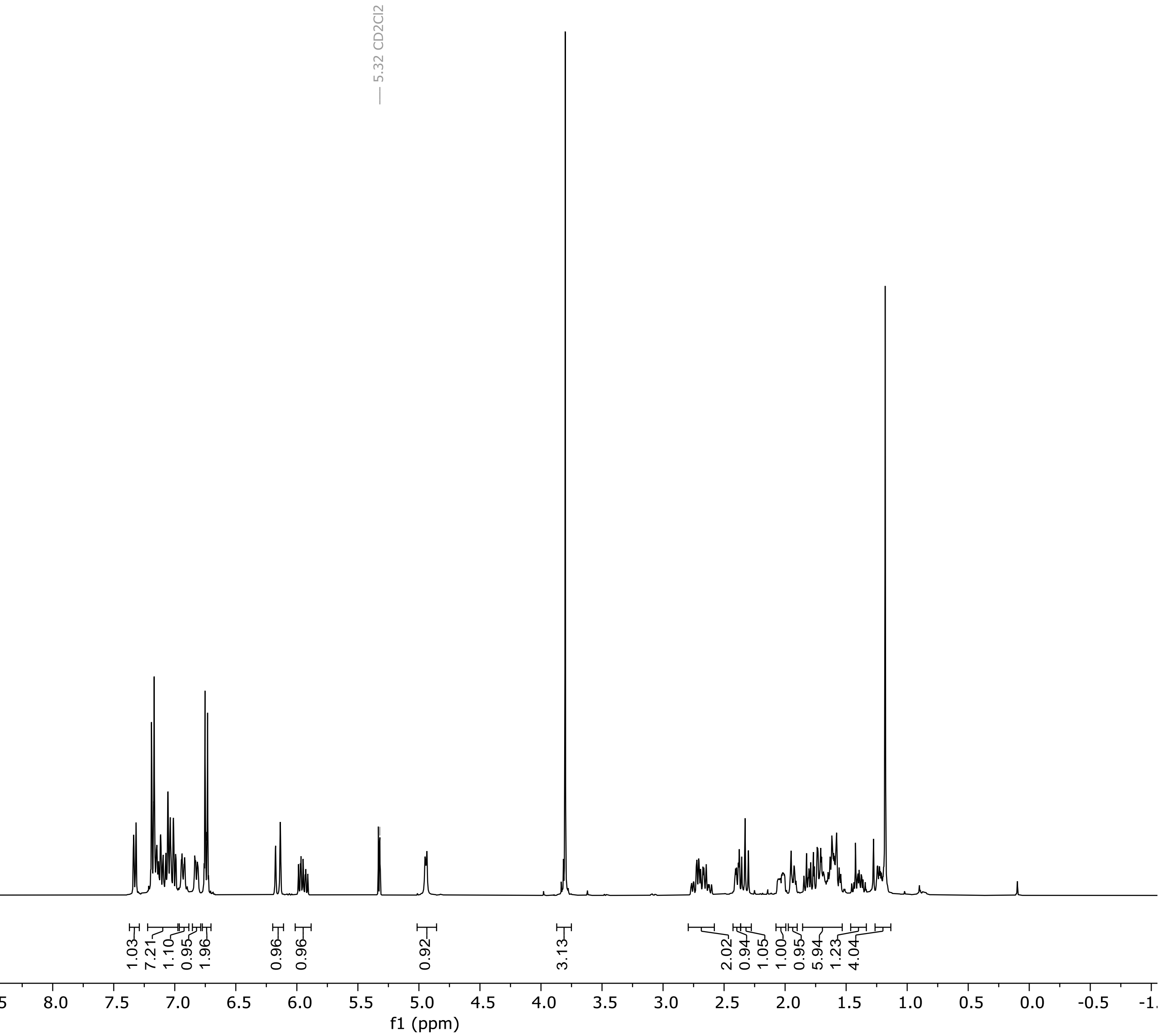

^{13}C NMR (101 MHz, CD_2Cl_2)

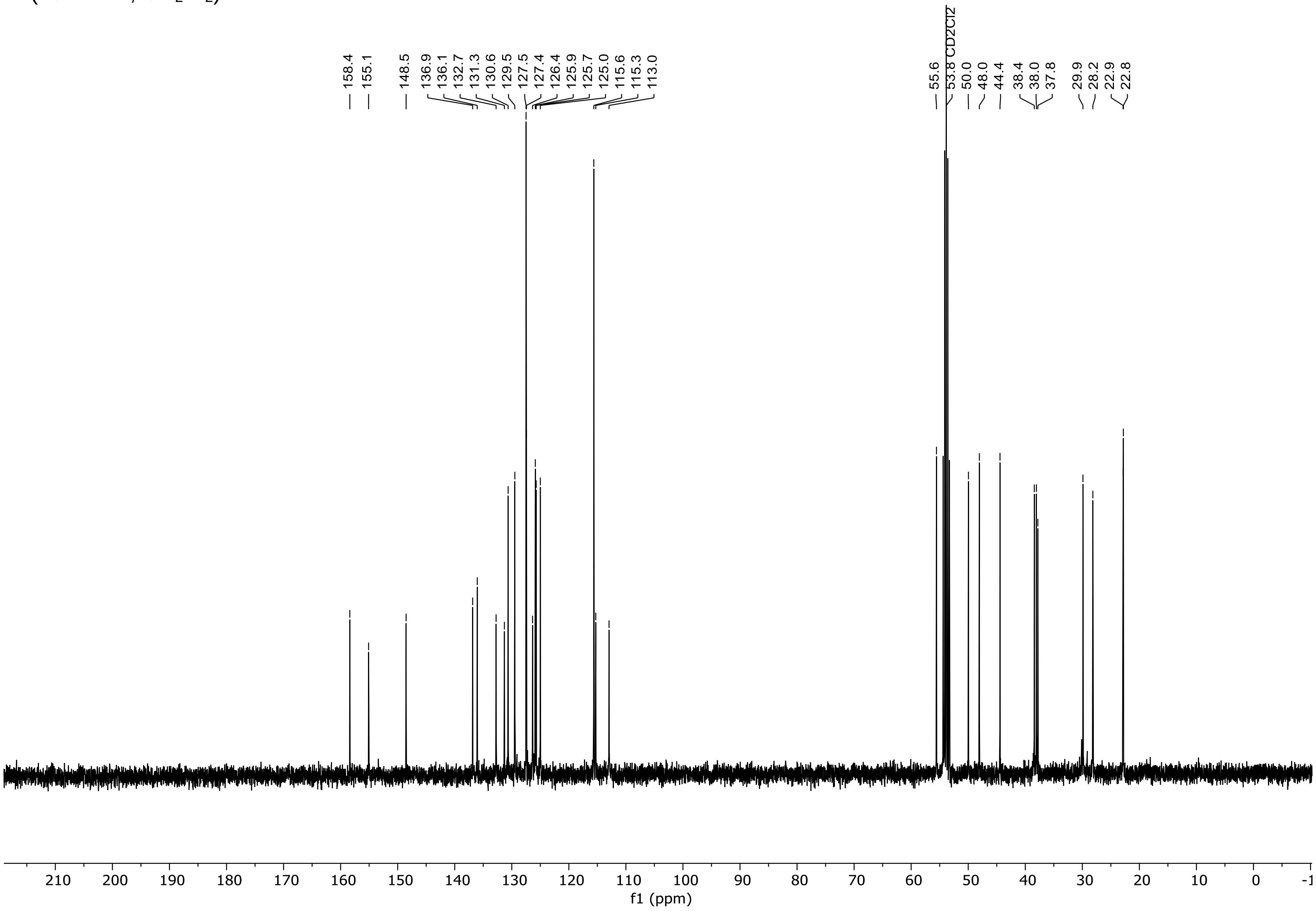

(1*R*,2*R*,4*aS*,10*aS*)-1-(4-methoxyphenyl)-4*a*-methyl-2-(3-methylbut-2-en-1-yl)-1,2,3,4,4*a*,9,10,10*a*-octahydrophenanthrene 2s.

^{1}H NMR (400 MHz, $CDCl_3$)

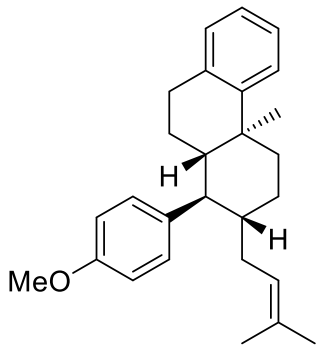

2s

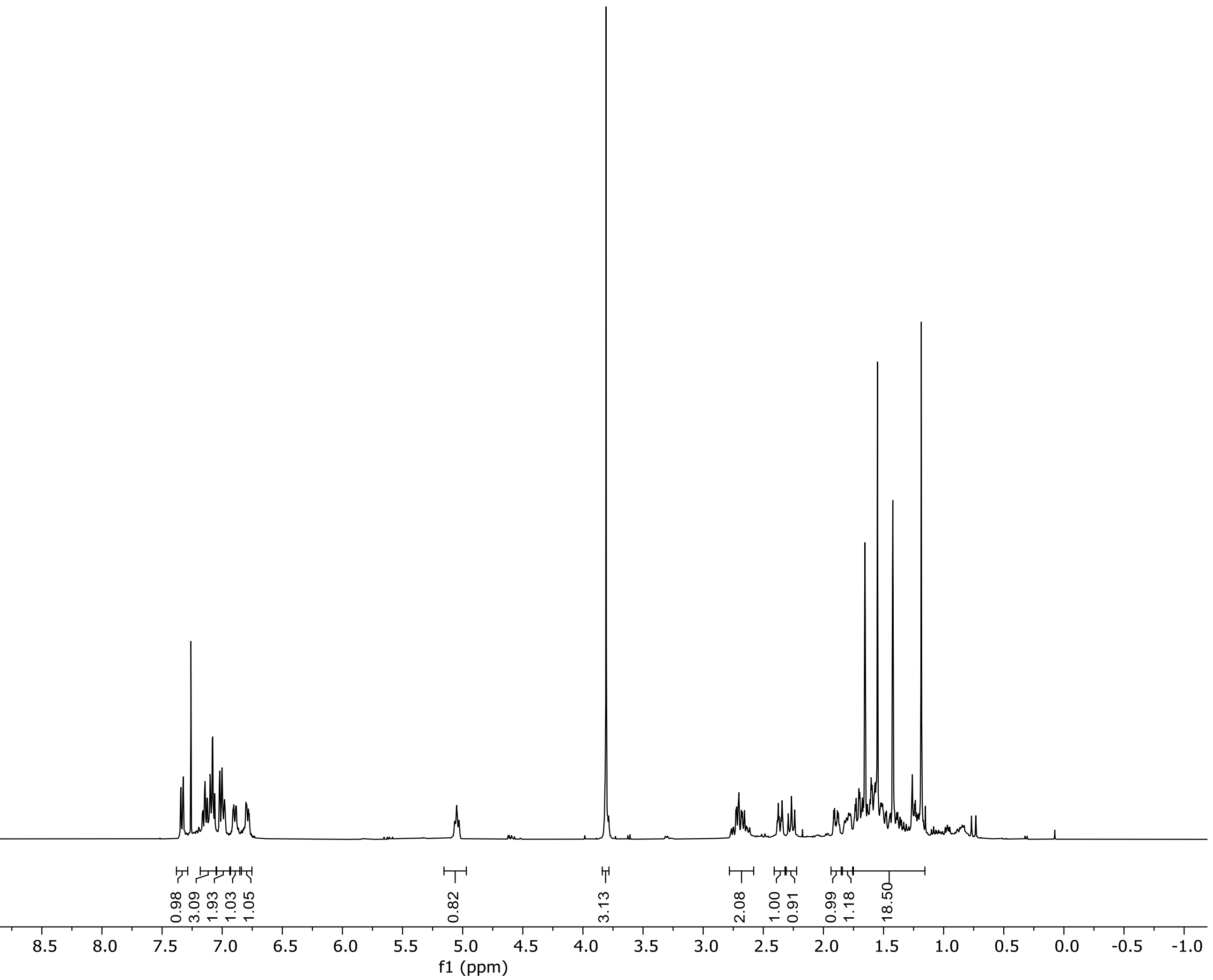

^{13}C NMR (101 MHz, $CDCl_3$)

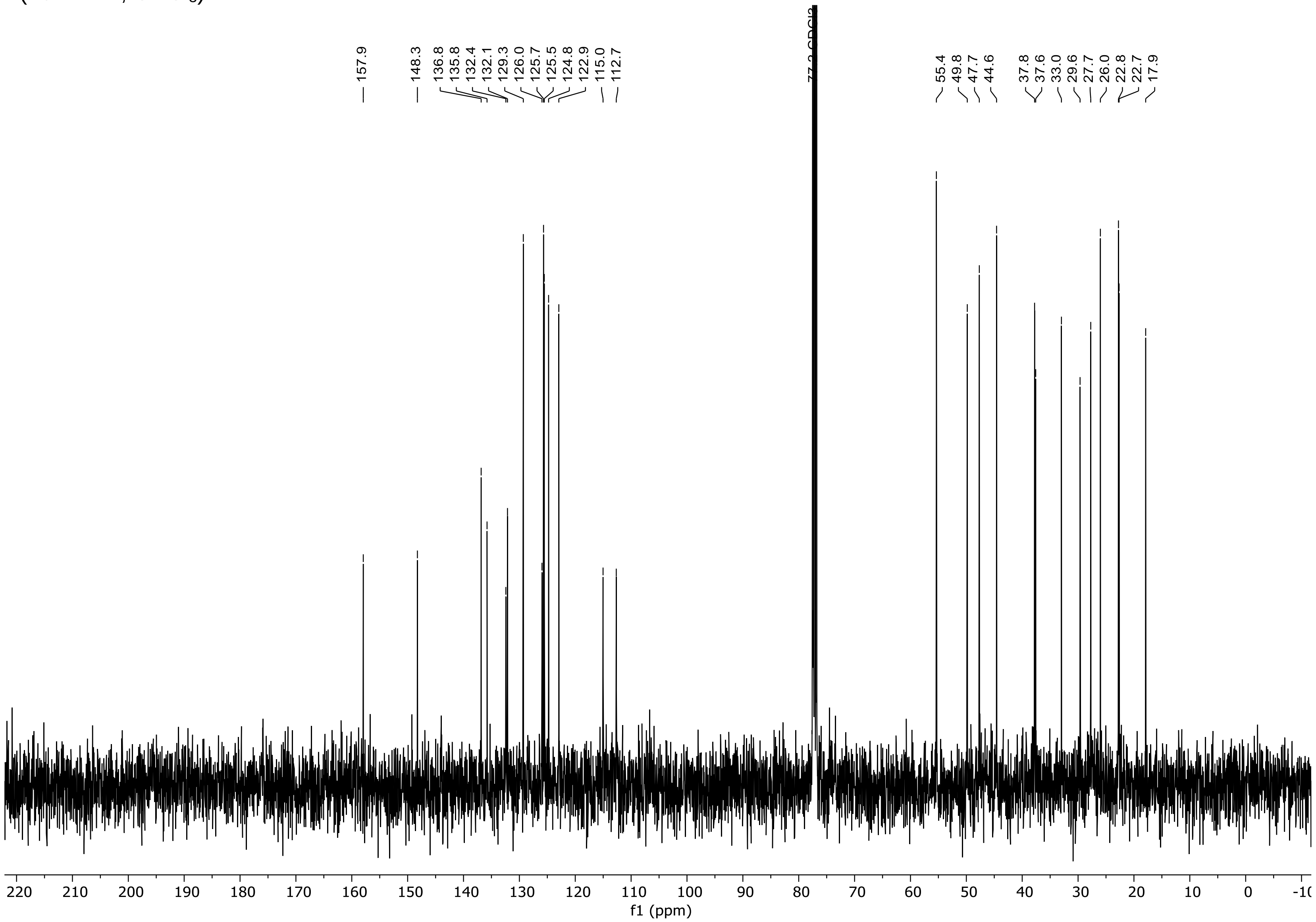

^{1}H (400 MHz)-^{13}C (101 MHz) **HSQC-2D** ($CDCl_3$)

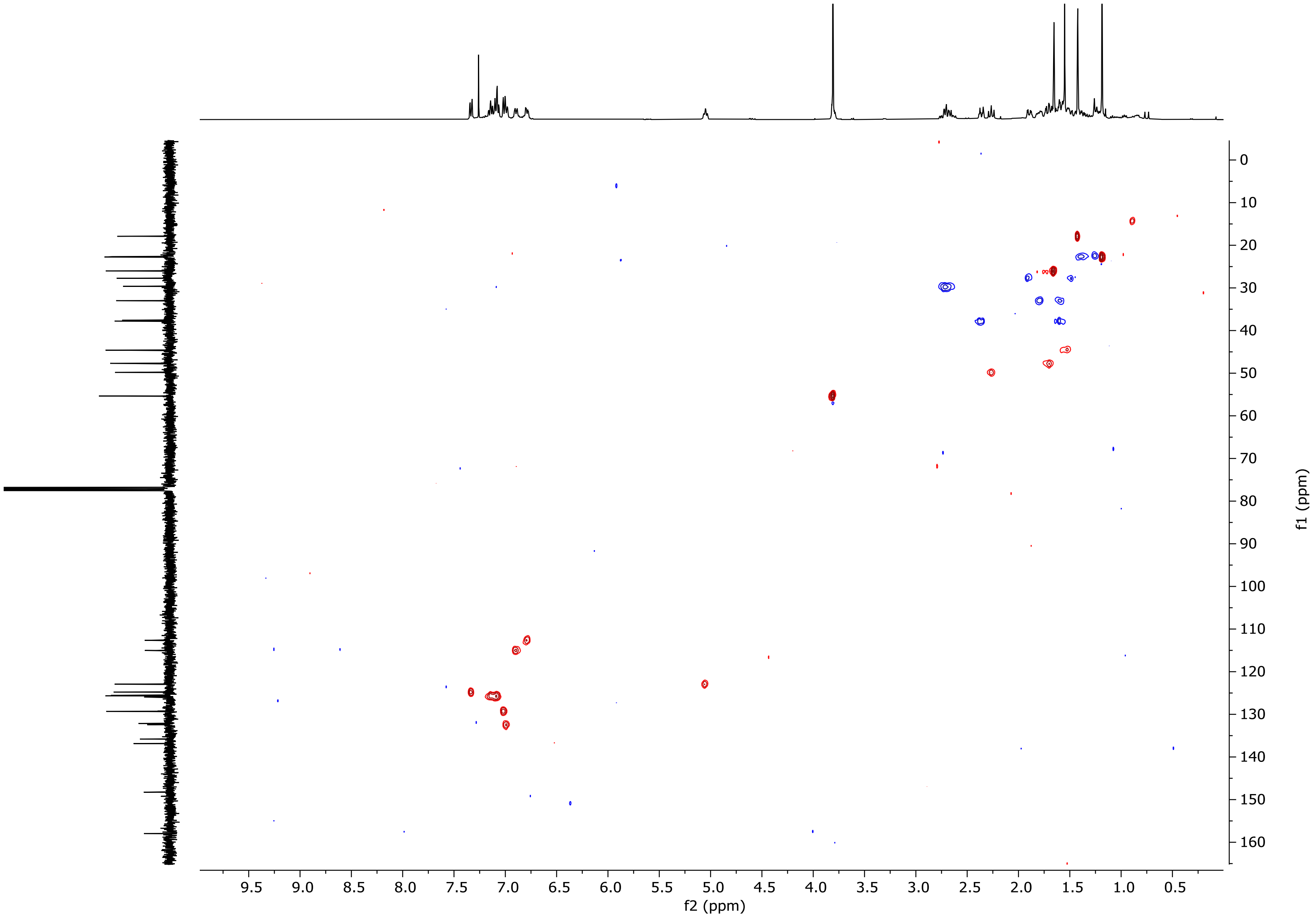

(±)-(1*S*,2*S*,4a*S*,10a*R*)-2-benzhydryl-1-(4-methoxyphenyl)-1,2,3,4,4a,9,10,10a-octahydrophenanthrene 2t.

^{1}H NMR (400 MHz, $CDCl_3$)

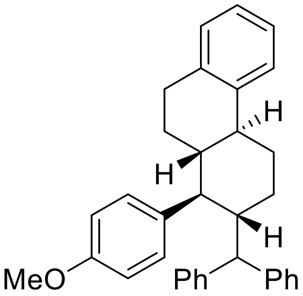

2t

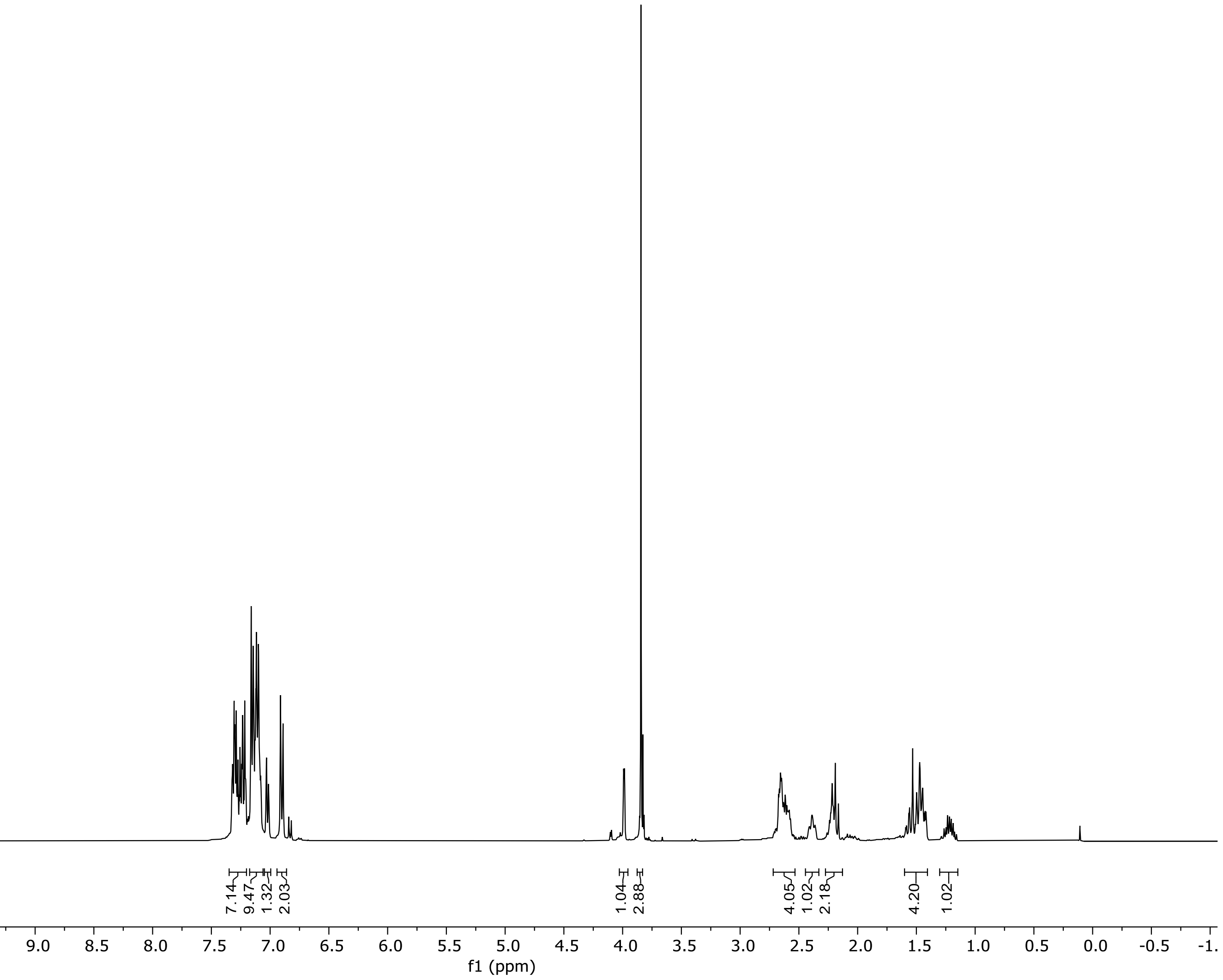

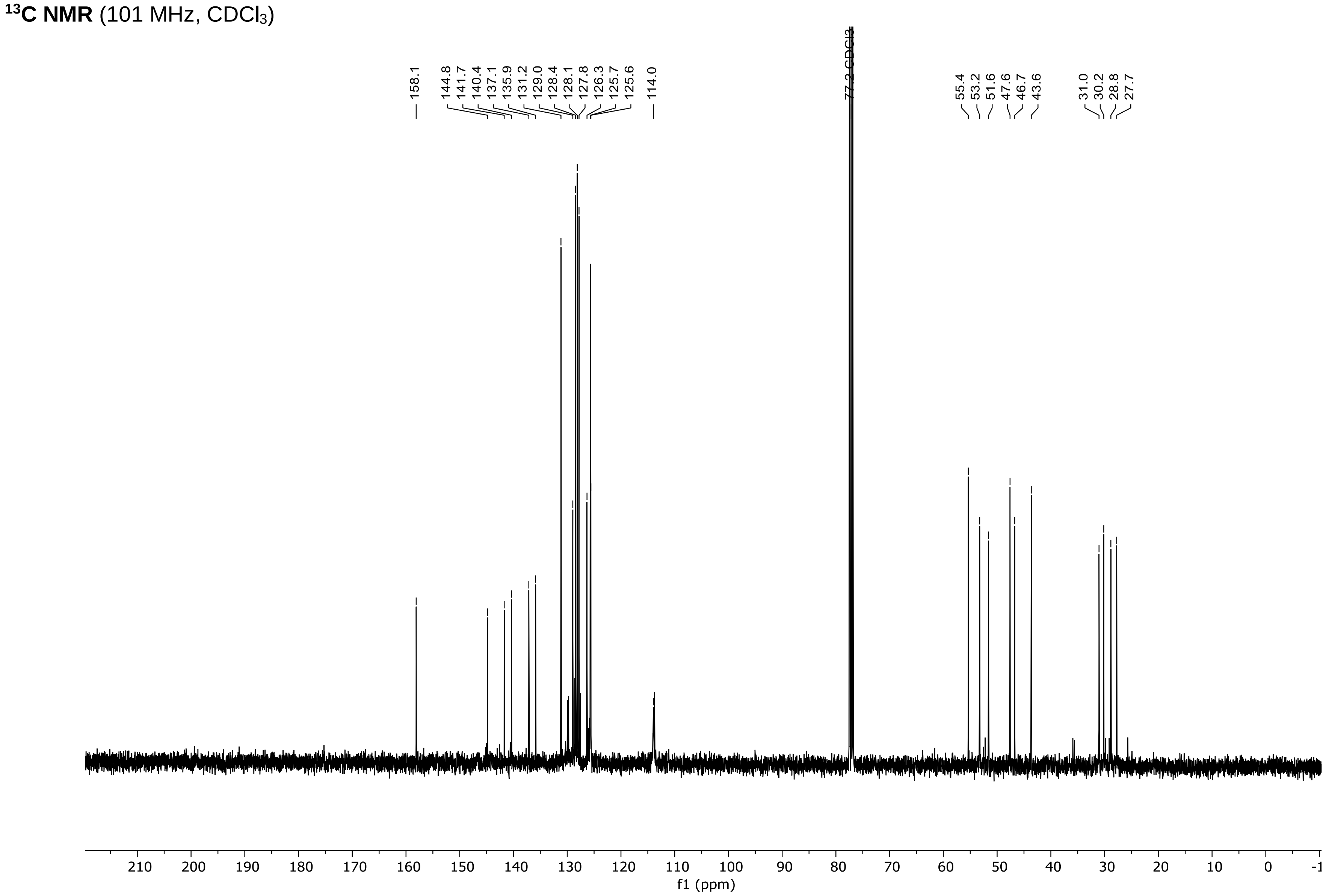
^{13}C NMR (101 MHz, CDCl$_3$)
158.1
144.8
141.7
140.4
137.1
135.9
131.2
129.0
128.4
128.1
127.8
126.3
125.7
125.6
114.0
77.2 CDCl3
55.4
53.2
51.6
47.6
46.7
43.6
31.0
30.2
28.8
27.7
210
200
190
180
170
160
150
140
130
120
110
100
90
80
70
60
50
40
30
20
10
0
-1
f1 (ppm)

^{1}**H** (400 MHz)-^{13}**C** (101 MHz) **HSQC-2D** ($CDCl_3$)

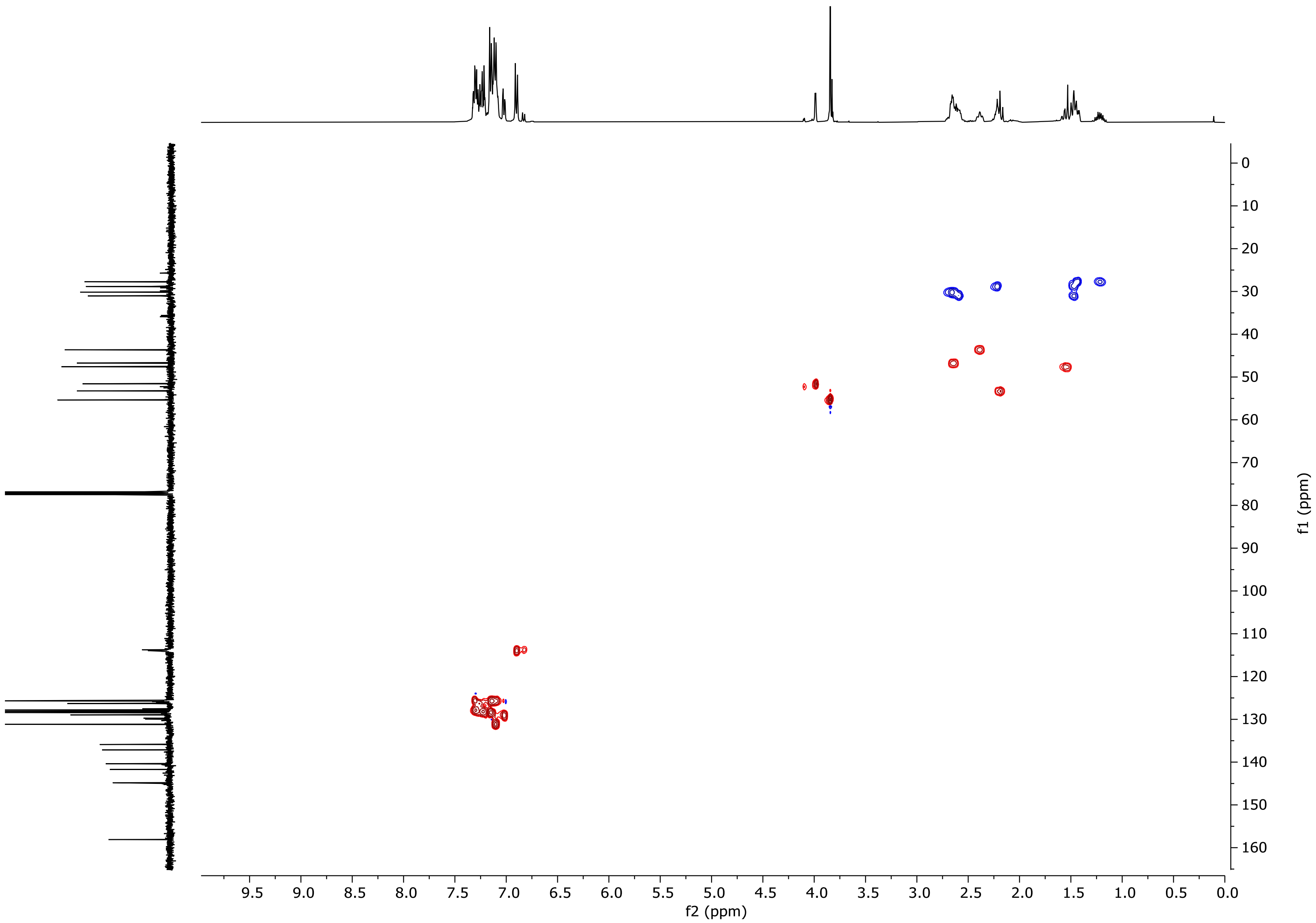

NOESY-2D (600 MHz, $CDCl_3$)

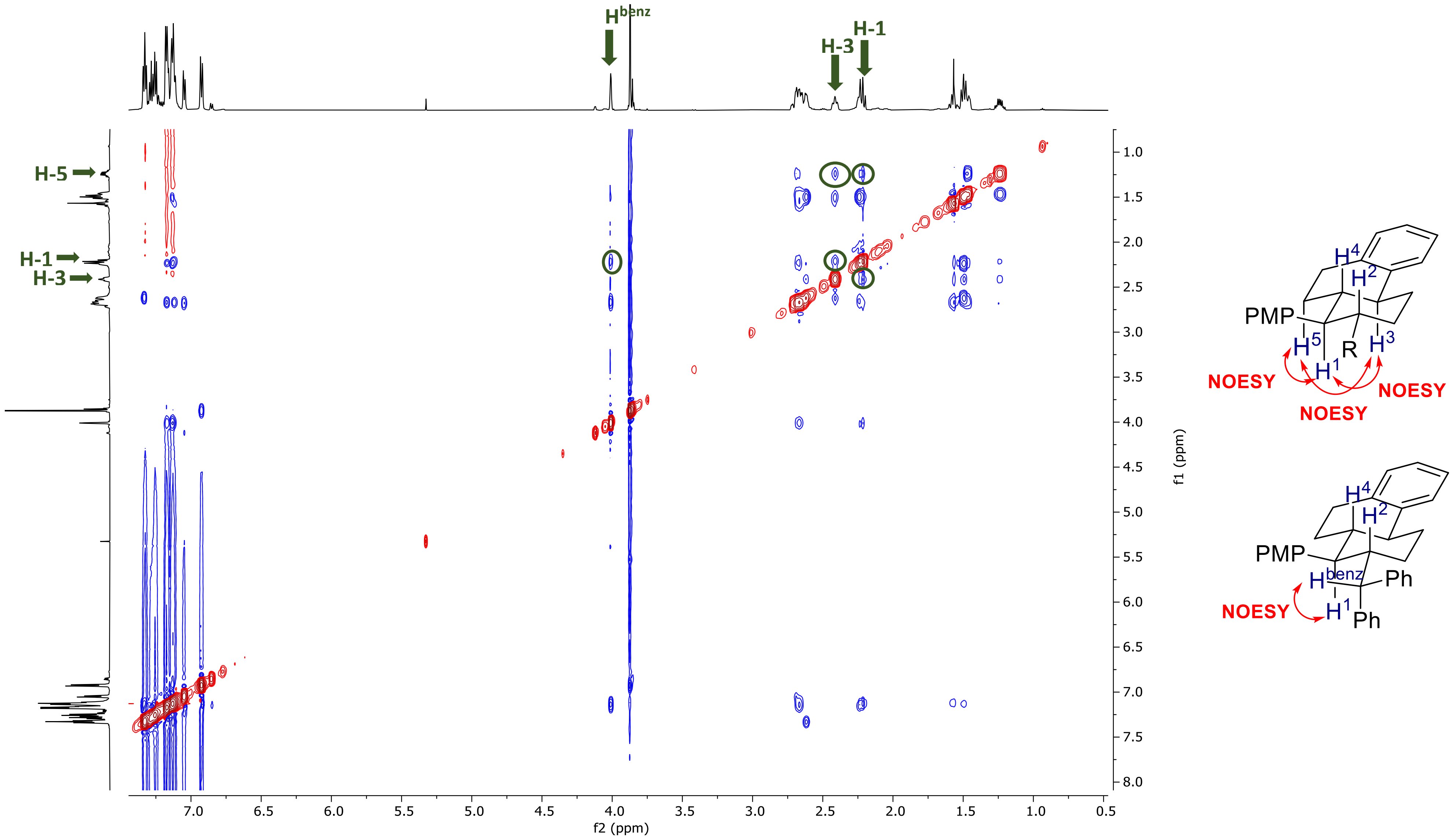

(±)-4-((*E*)-3-((1*R*,2*R*,4*aS*,10*aR*)-1-(4-methoxyphenyl)-1,2,3,4,4*a*,9,10,10*a*-octahydrophenanthren-2-yl)prop-1-en-1-yl)phenol 2u.

^{1}H NMR (400 MHz, $CDCl_3$)

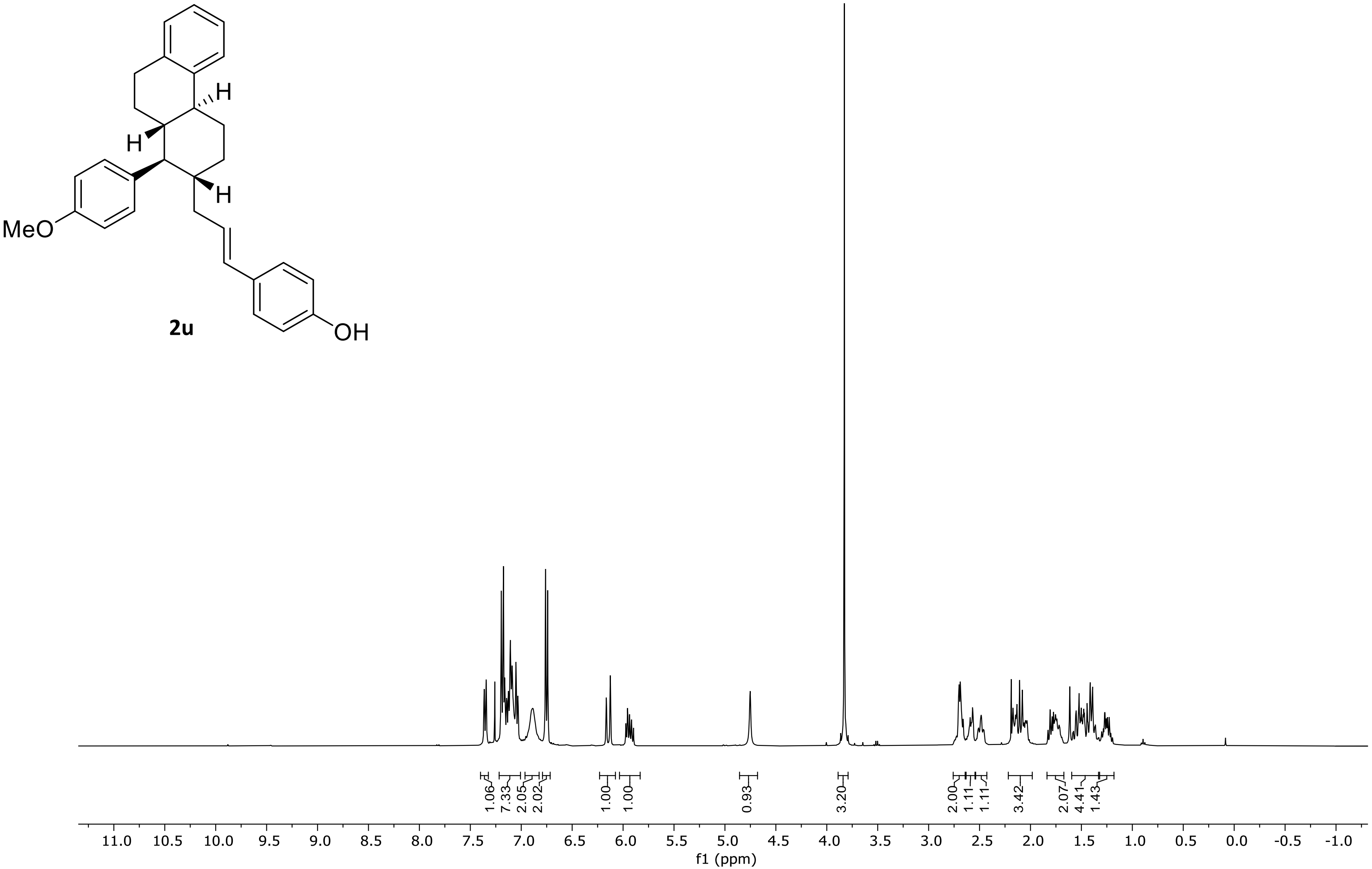

2u

^{13}C NMR (101 MHz, $CDCl_3$)

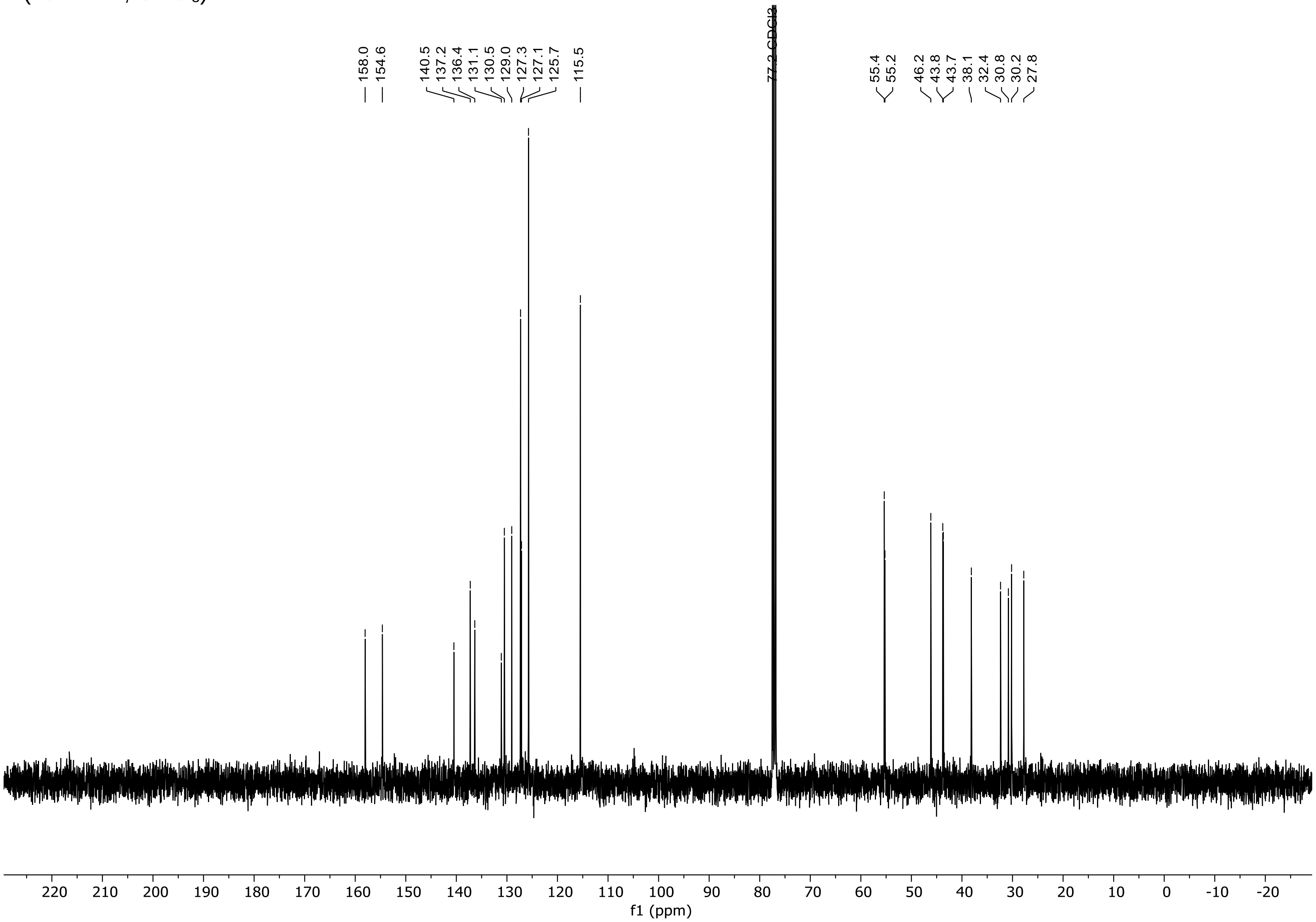

NOESY-2D (600 MHz, $CDCl_3$)

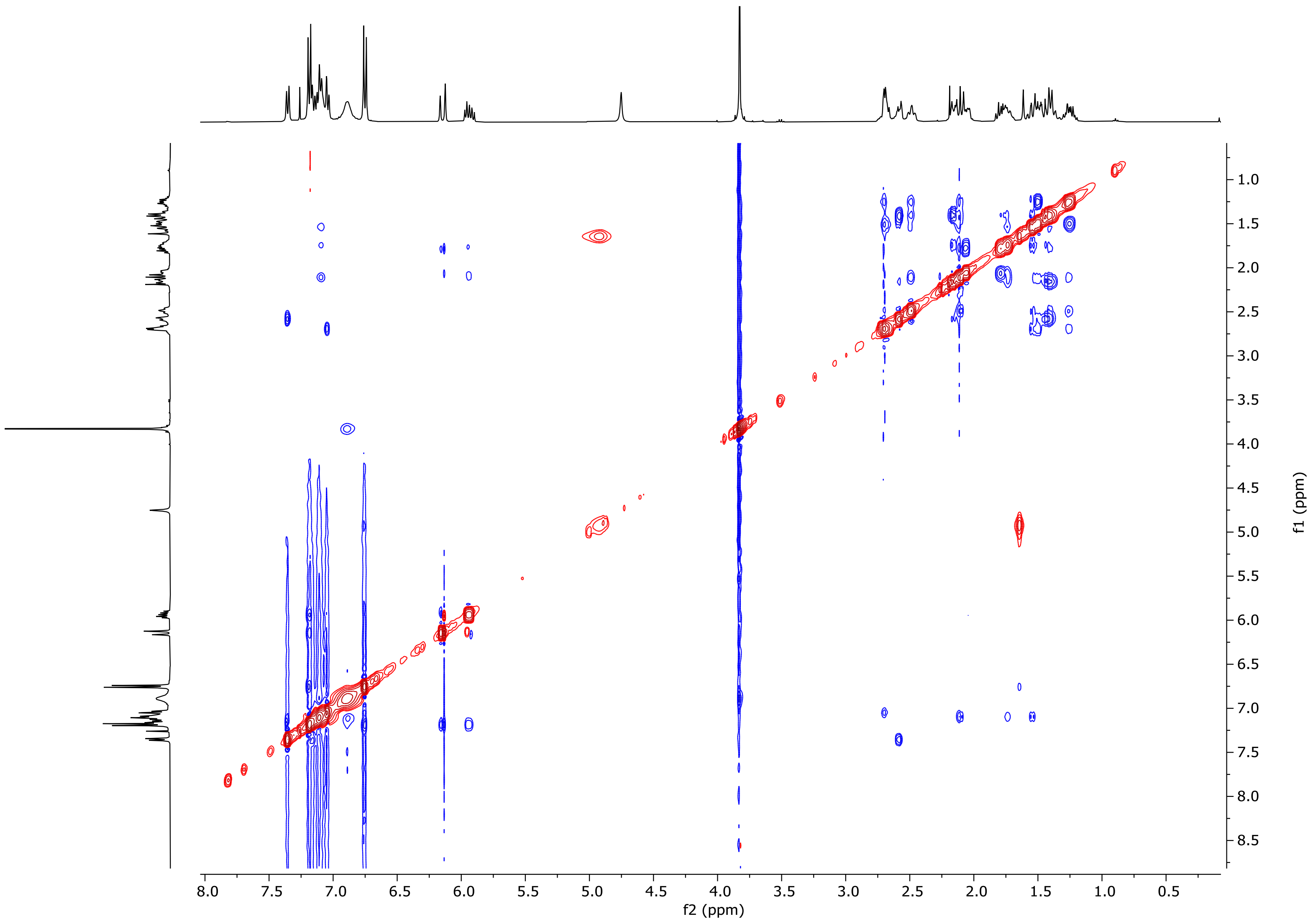

(±)-Methyl 2-hydroxy-5-((*S*)-1-((1*R*,2*R*,4*aS*,10*aS*)-1-(4-methoxyphenyl)-4*a*-methyl-1,2,3,4,4*a*,9,10,10*a*-octahydrophenanthren-2-yl)ethyl)benzoate (major)-2v and (±)-Methyl 2-hydroxy-5-((*R*)-1-((1*R*,2*R*,4*aS*,10*aS*)-1-(4-methoxyphenyl)-4*a*-methyl-1,2,3,4,4*a*,9,10,10*a*-octahydrophenanthren-2-yl)ethyl)benzoate (minor)-2v.

^{1}H NMR (400 MHz, $CDCl_3$)

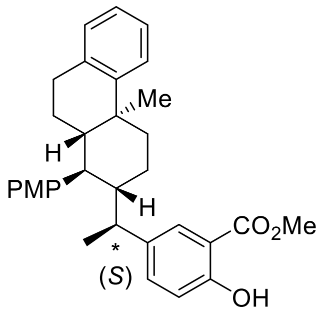

2v (major)

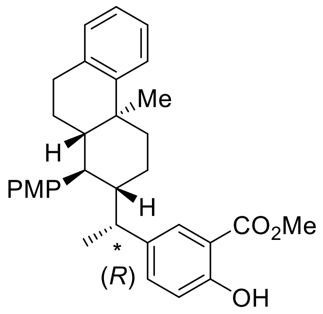

2v (minor)

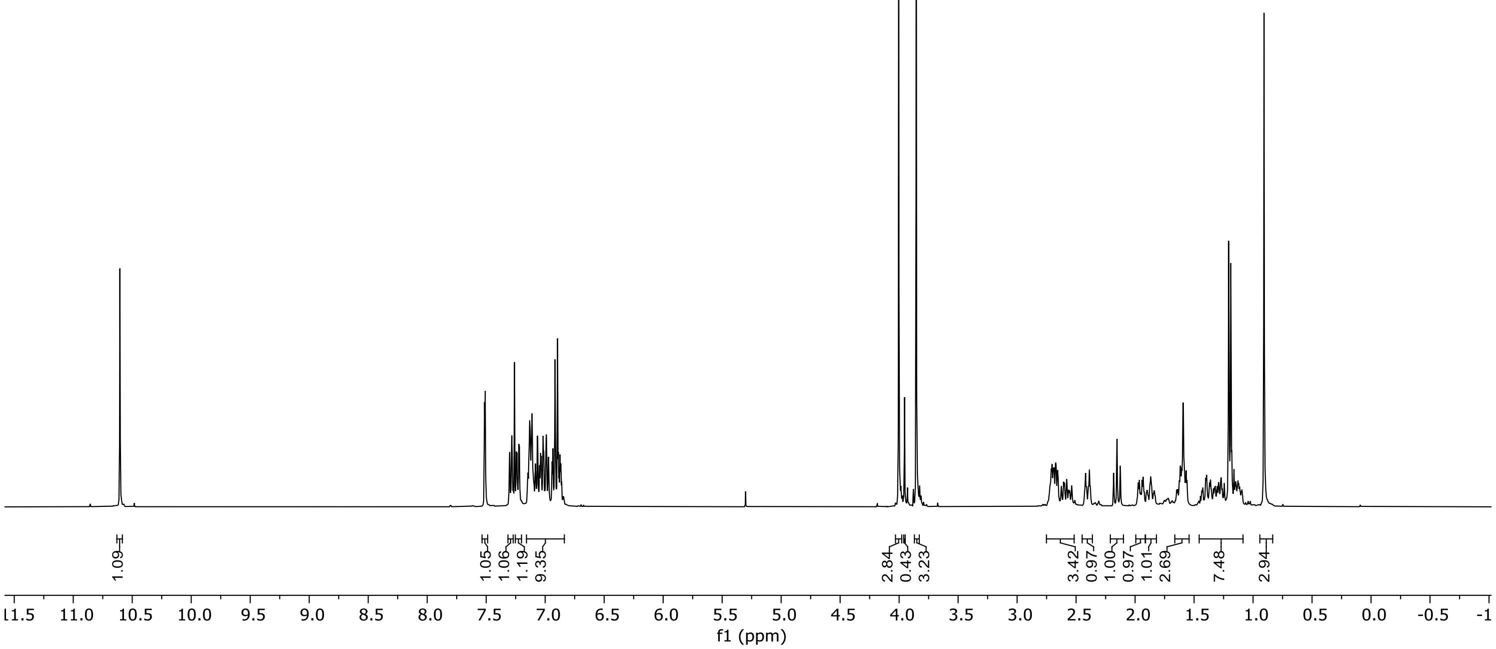

^{13}C NMR (101 MHz, $CDCl_3$)

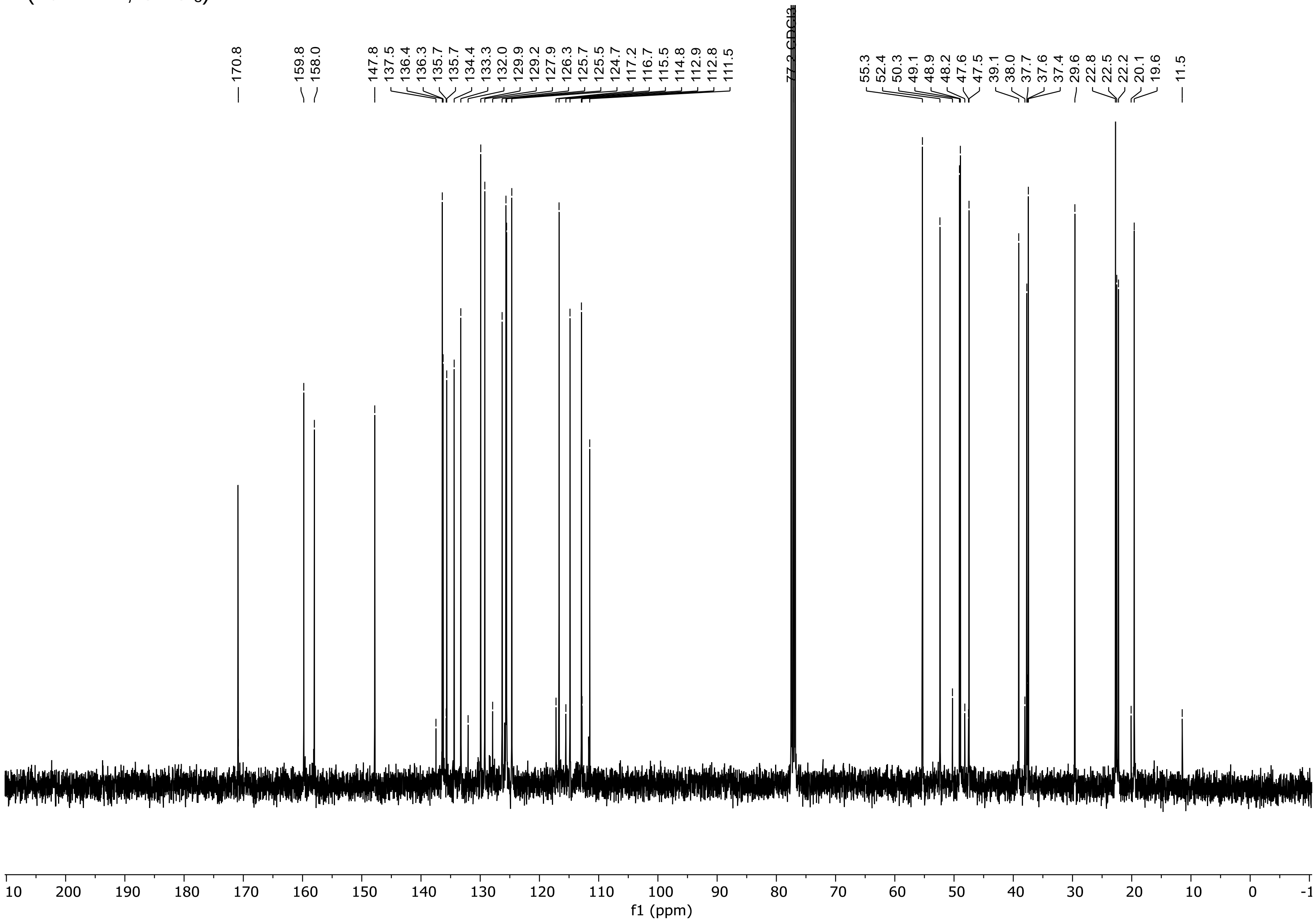

^{1}**H** (400 MHz)-^{13}**C** (101 MHz) **HSQC-2D** ($CDCl_3$)

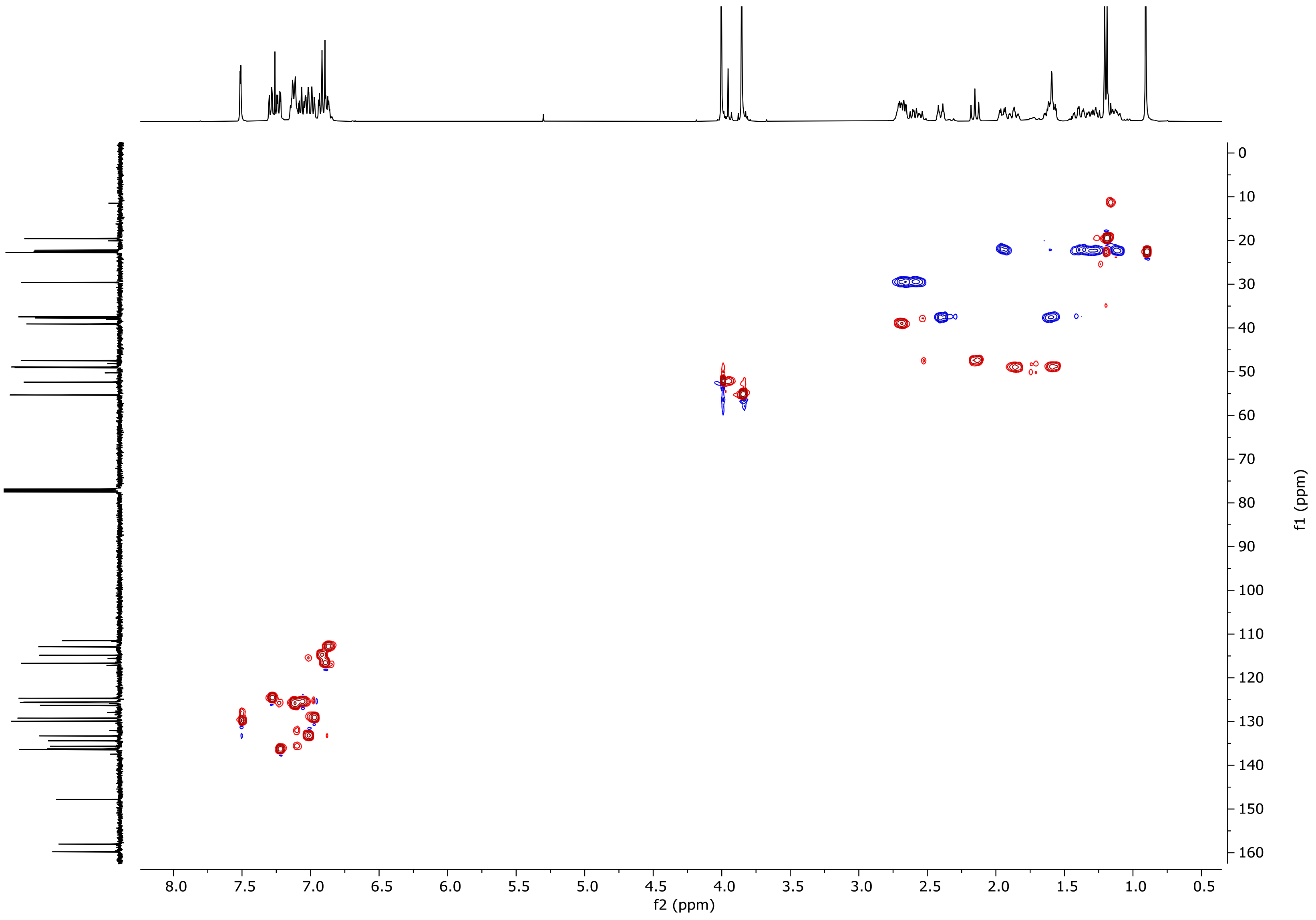

(±)-(1*R*,2*R*,4a*S*,10a*S*)-2-(3-(4-chlorophenyl)cyclopent-2-en-1-yl)-1-(4-methoxyphenyl)-4a-methyl-1,2,3,4,4a,9,10,10a-octahydrophenanthrene 2w.

^{1}H NMR (400 MHz, $CDCl_3$)

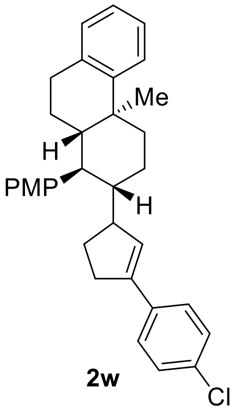

2w

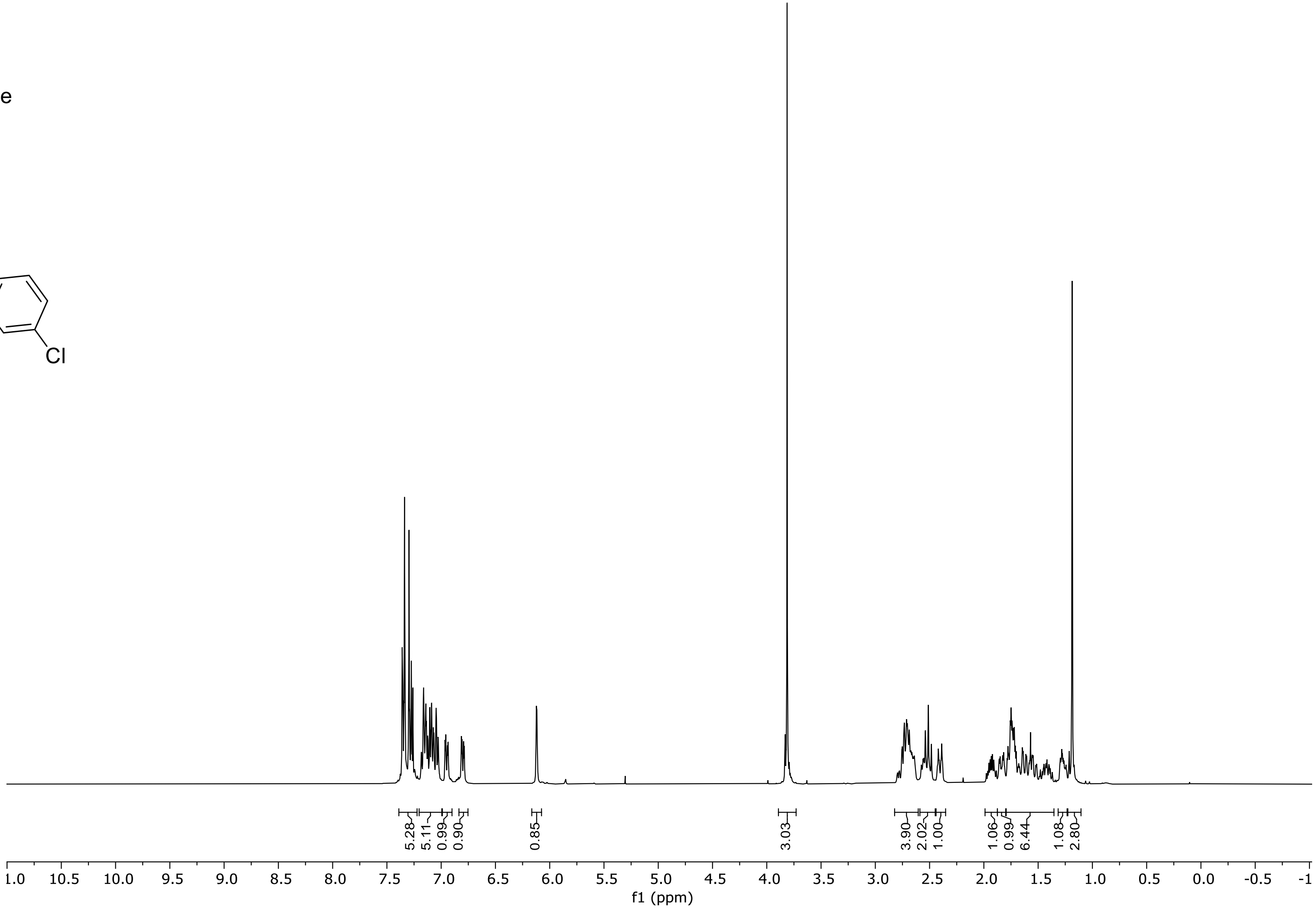

^{13}**C NMR** (101 MHz, $CDCl_3$)

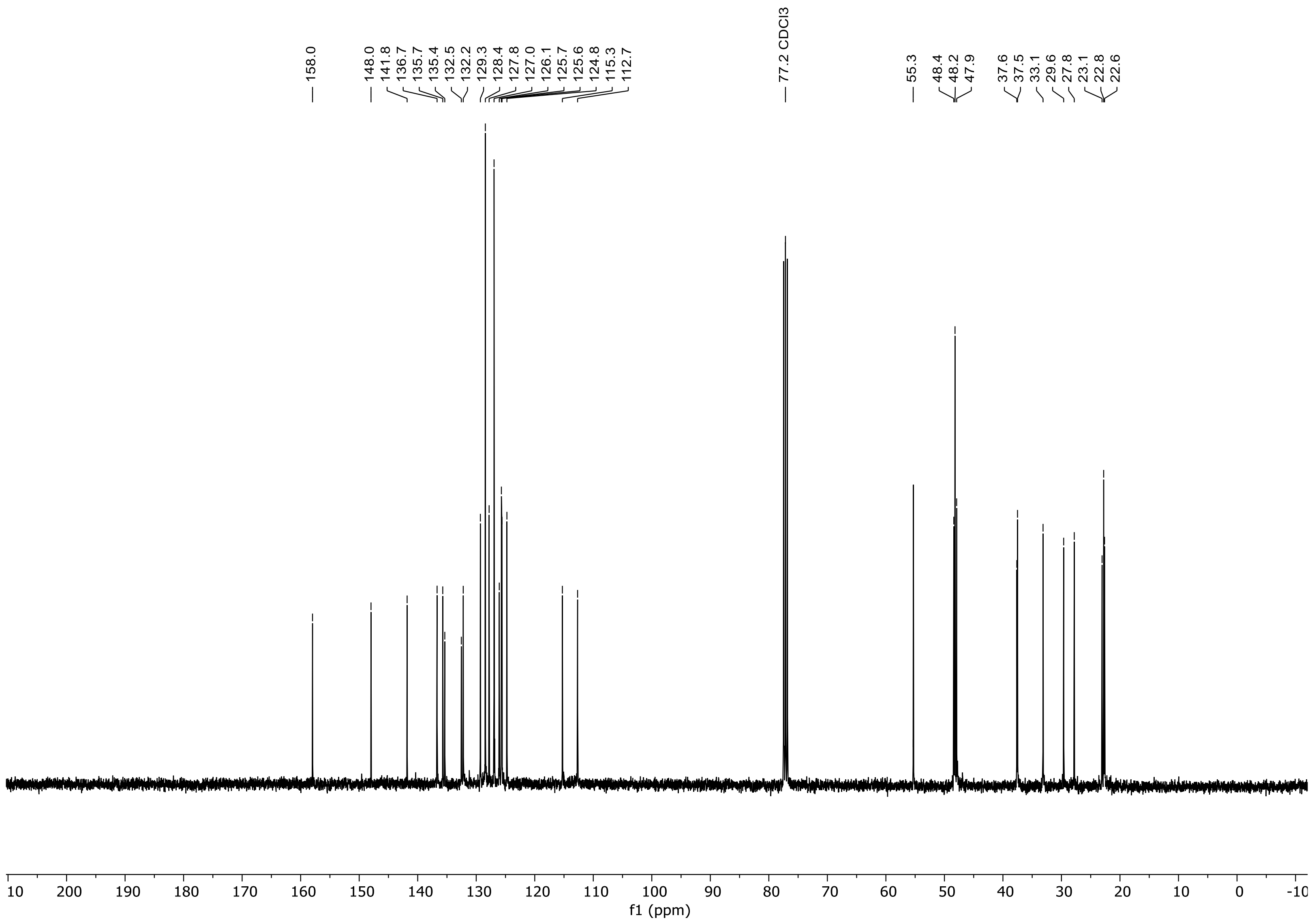

7.3 Products **6a**-**6f**

(±)-(1*R*,2S,4aS,10aS)-2-benzhydryl-1-((E)-4-methoxystyryl)-4a-methyl-1,2,3,4,4a,9,10,10a-octahydrophenanthrene (major)-6a and (±)-(1*S*,2S,4aS,10aS)-2-benzhydryl-1-((*E*)-4-methoxystyryl)-4a-methyl-1,2,3,4,4a,9,10,10a-octahydrophenanthrene (minor)-6a.

^{1}H NMR (600 MHz, $CDCl_3$)

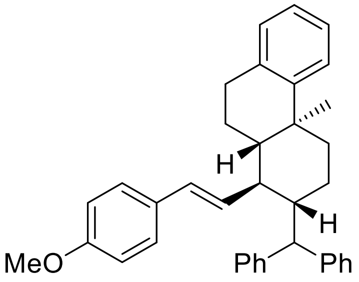

Major 6a

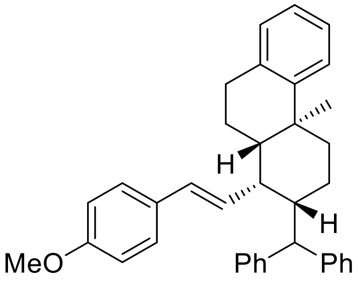

Minor 6a

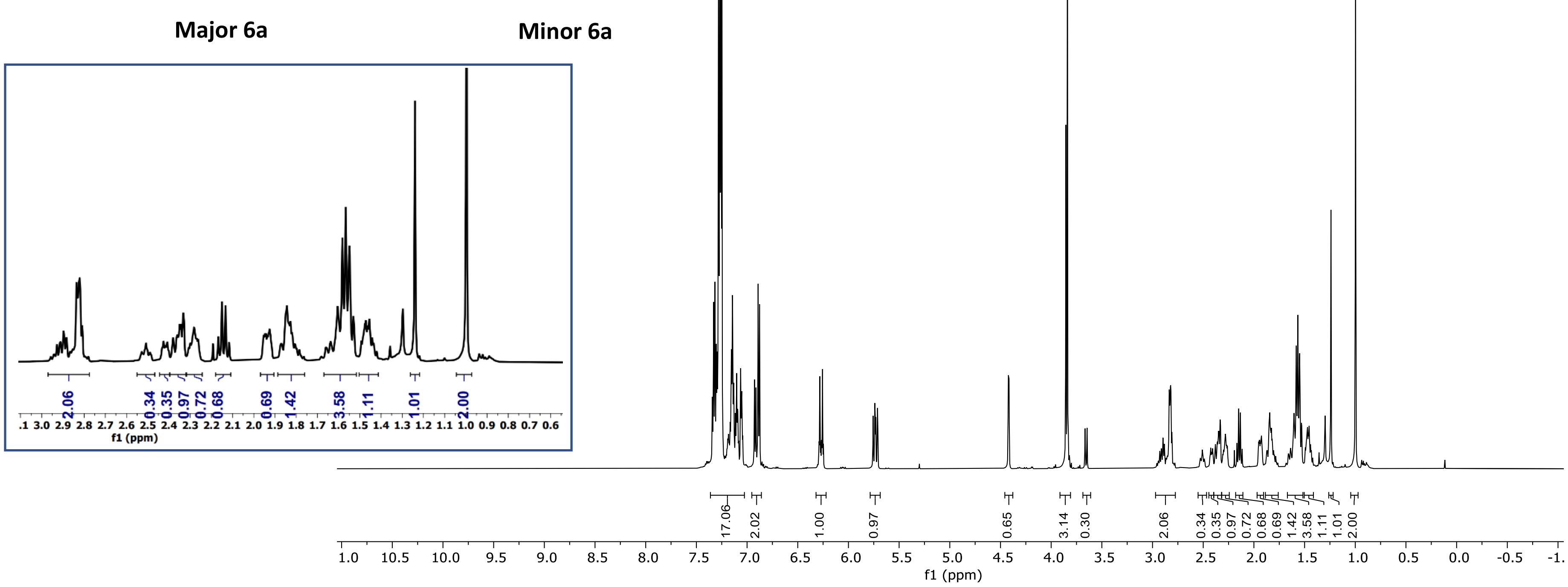

^{13}C NMR (151 MHz, $CDCl_3$)

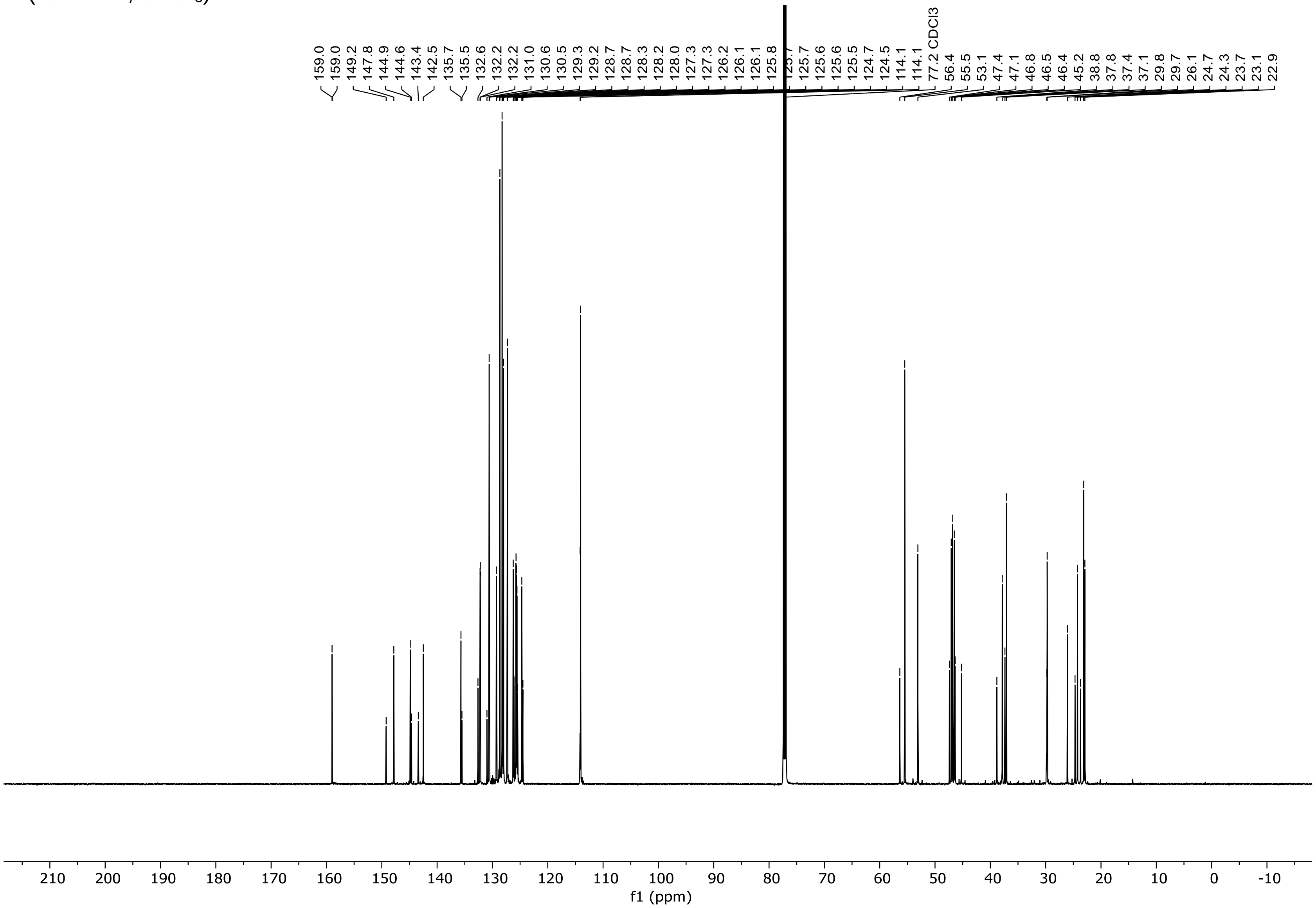

^{1}H (400 MHz)-**^{13}C** (101 MHz) **HSQC-2D** (CDCl$_3$)

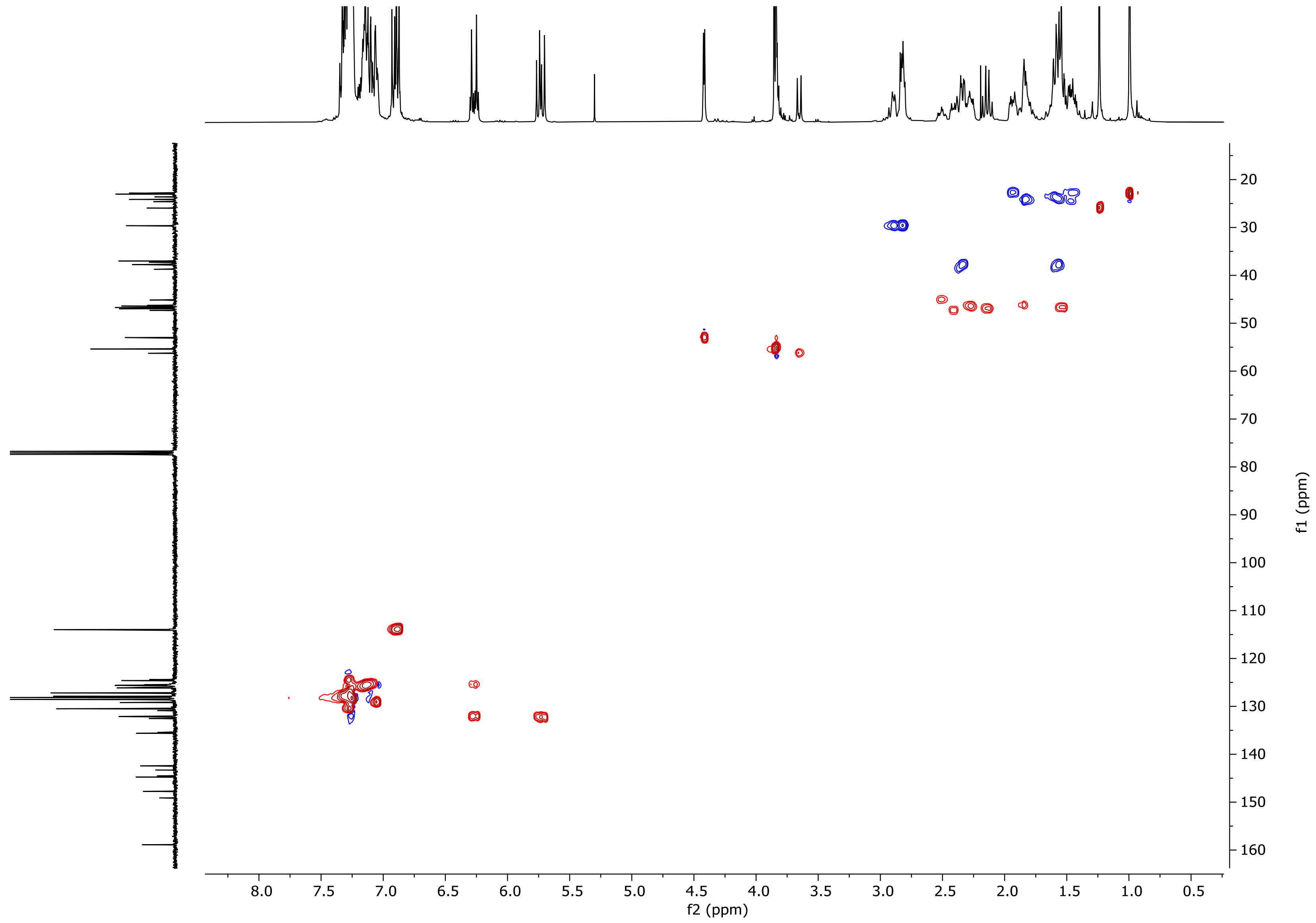

COSY-2D (400 MHz, $CDCl_3$)

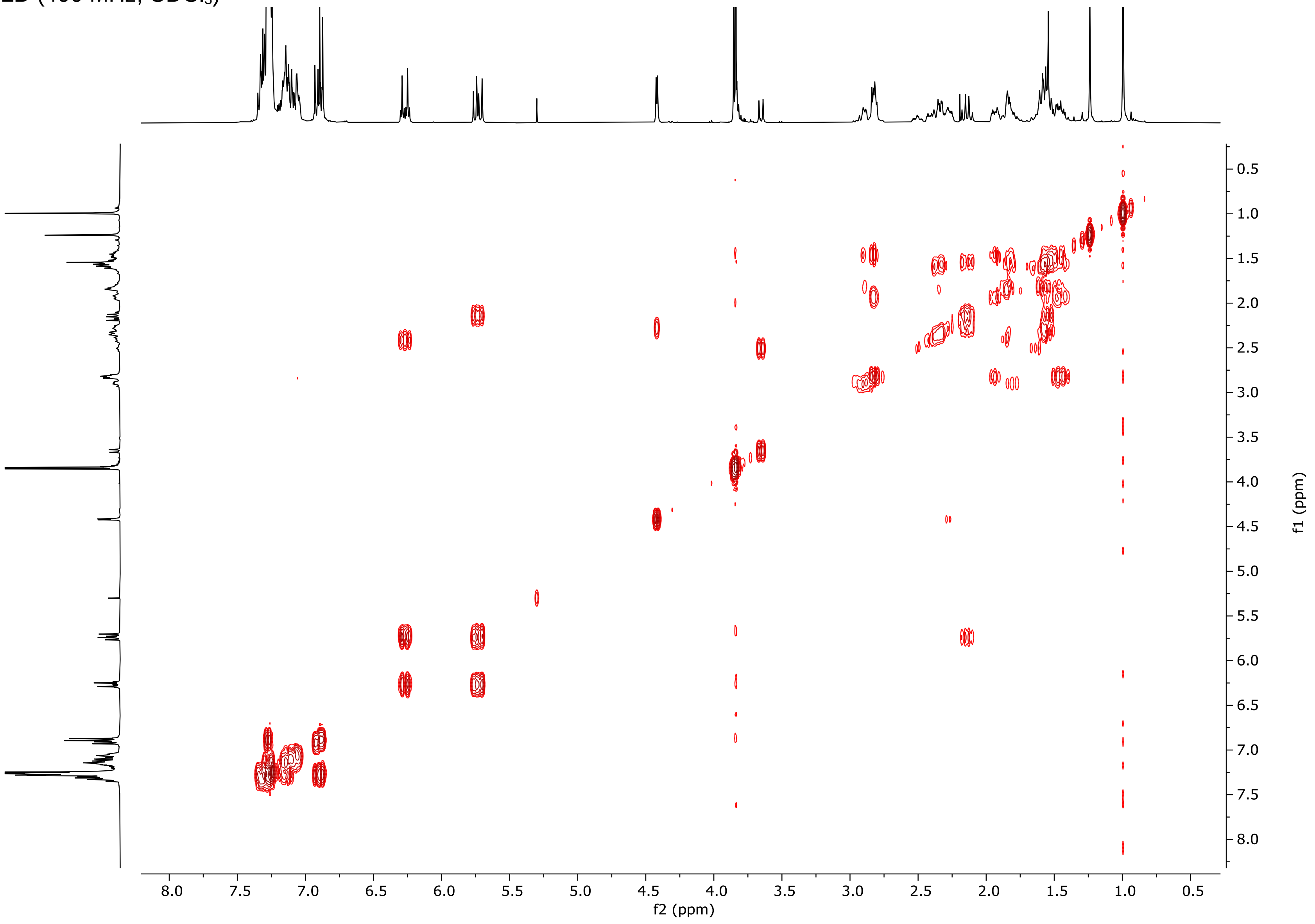

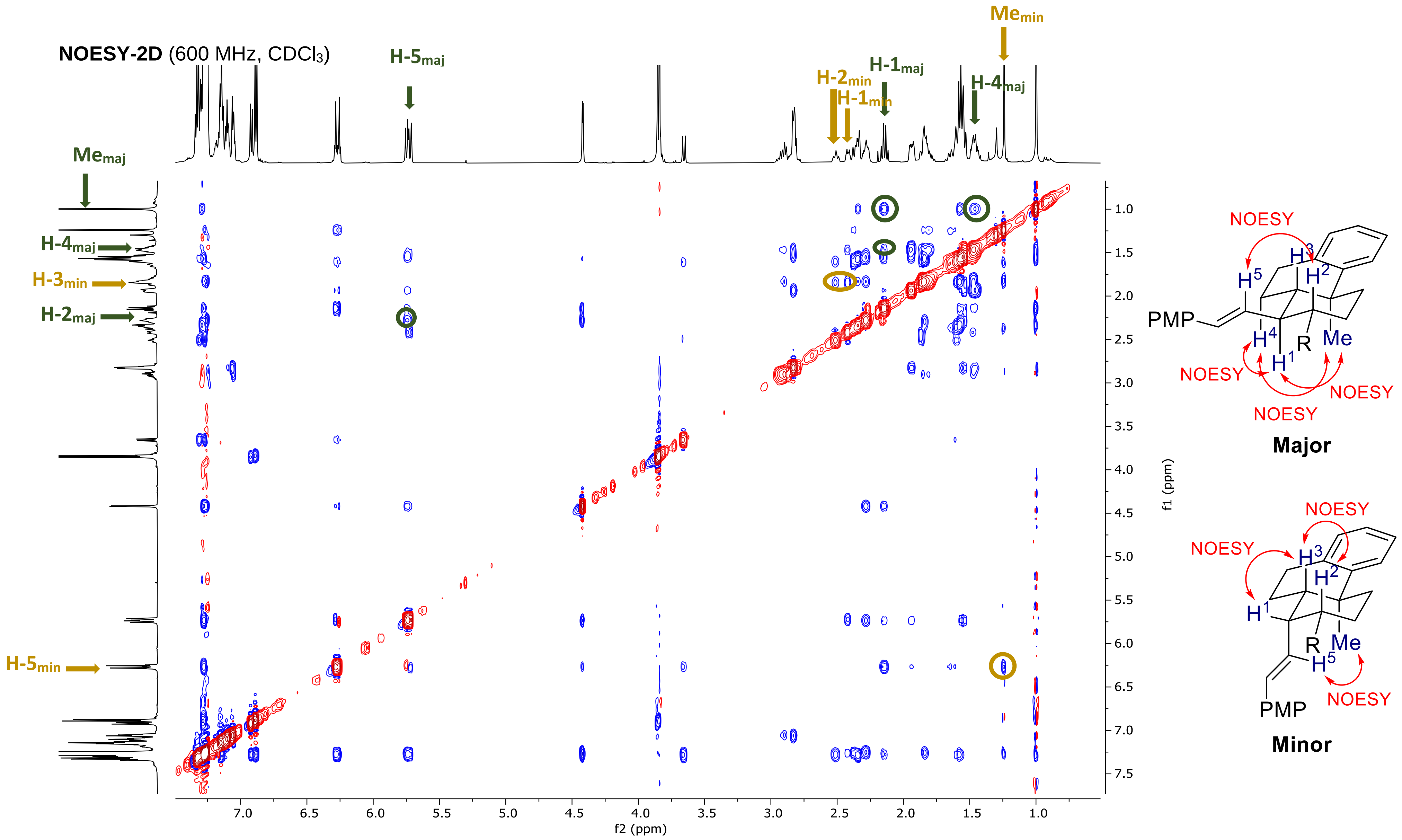
NOESY-2D (600 MHz, CDCl3)
H-5maj
H-2min
H-1min
H-1maj
H-4maj
Memin
Memaj
H-4maj
H-3min
H-2maj
H-5min
f2 (ppm)
f1 (ppm)
NOESY
PMP
R
Me
Major
Minor

^{1}**H** (600 MHz)-^{13}**C** (151 MHz) **HMBC-2D** ($CDCl_3$)

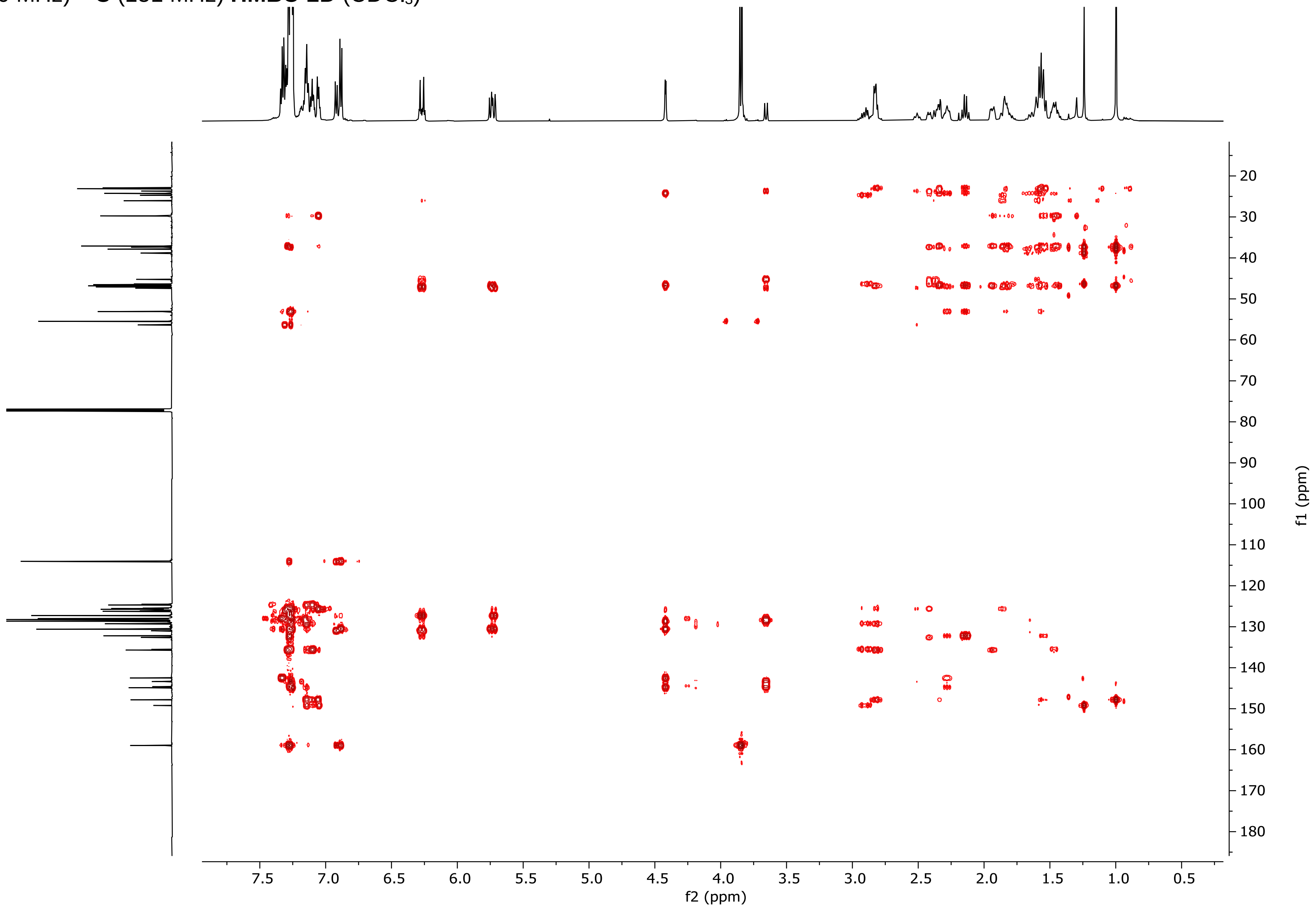

(±)-(1*R*,2*S*,4*aS*,10*aS*)-2-(bis(4-methoxyphenyl)methyl)-1-((*E*)-4-methoxystyryl)-4*a*-methyl-1,2,3,4,4*a*,9,10,10*a*-octahydrophenanthrene (major)-6b and (±)-(1*S*,2*S*,4*aS*,10*aS*)-2-(bis(4-methoxyphenyl)methyl)-1-((*E*)-4-methoxystyryl)-4*a*-methyl-1,2,3,4,4*a*,9,10,10*a*-octahydrophenanthren (minor)-6b.

^{1}H NMR (600 MHz, $CDCl_3$)

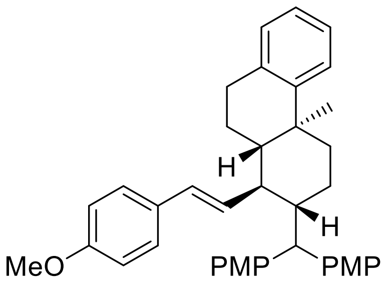

Major 6b

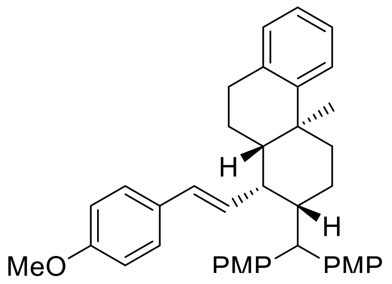

Minor 6b

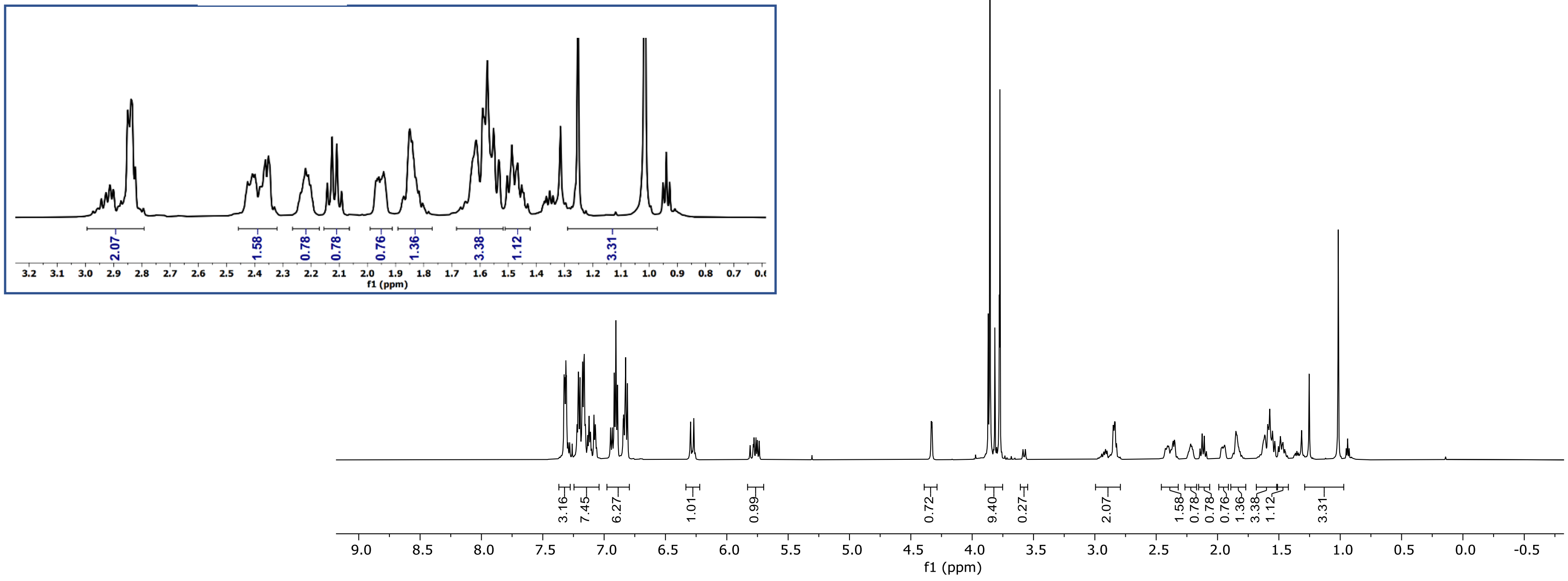

^{13}C NMR (101 MHz, $CDCl_3$)

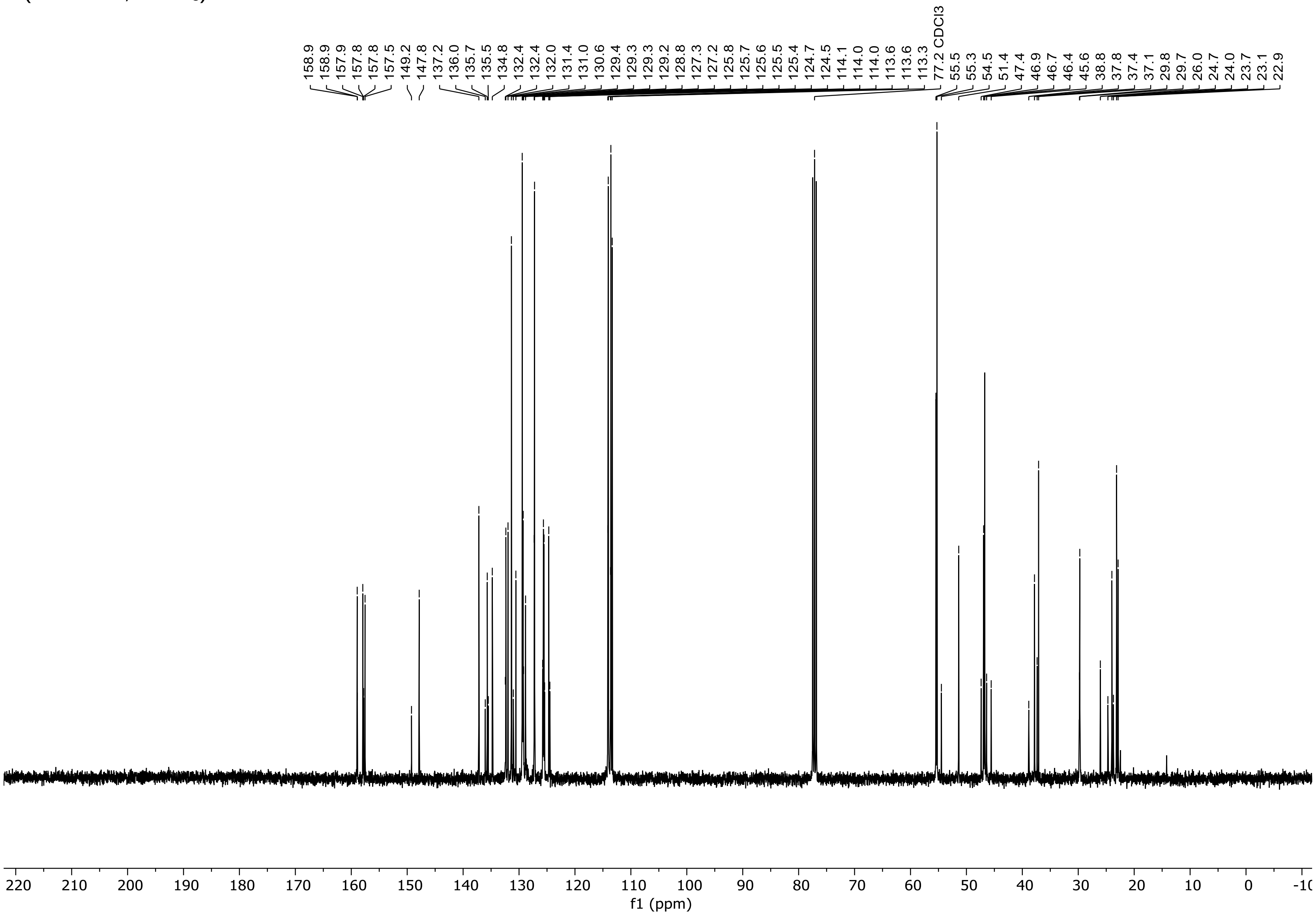

NOESY-2D (600 MHz, $CDCl_3$)

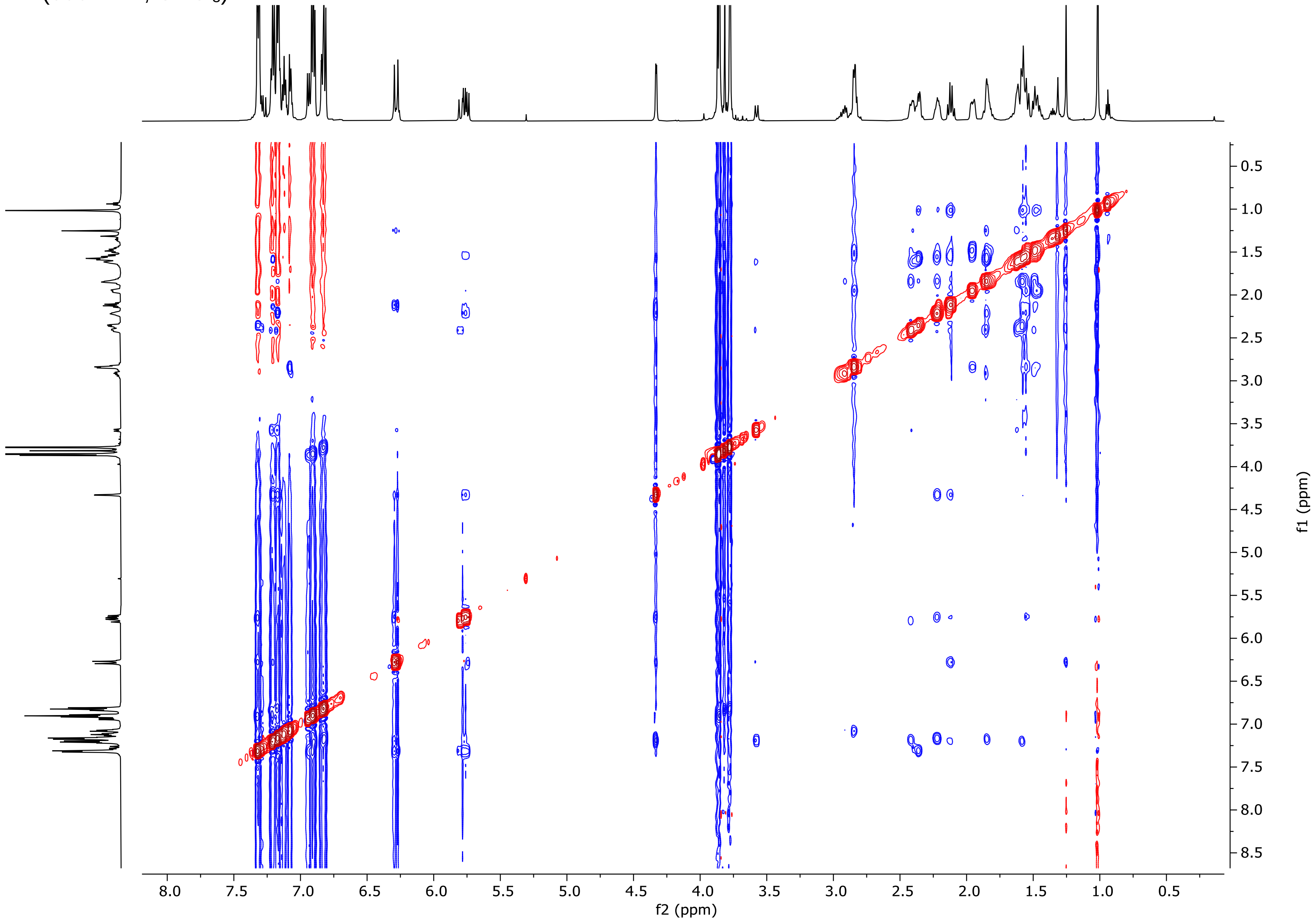

(±)-(1*S*,2*R*,4*aS*,10*aS*)-2-((*E*)-3-(4-methoxyphenyl)allyl)-1-((*E*)-4-methoxystyryl)-4$_a$-methyl-1,2,3,4,4*a*,9,10,10*a*-octahydrophenanthrene 6c.

^{1}H NMR (400 MHz, $CDCl_3$)

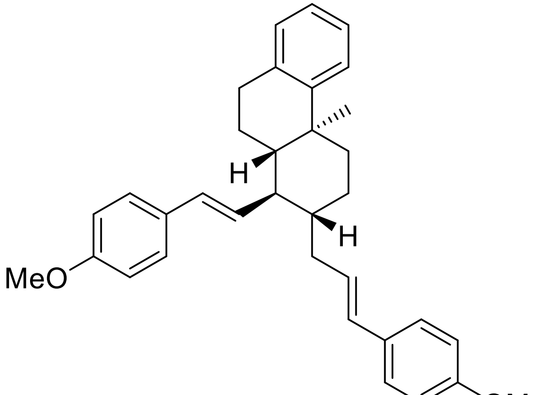

6c

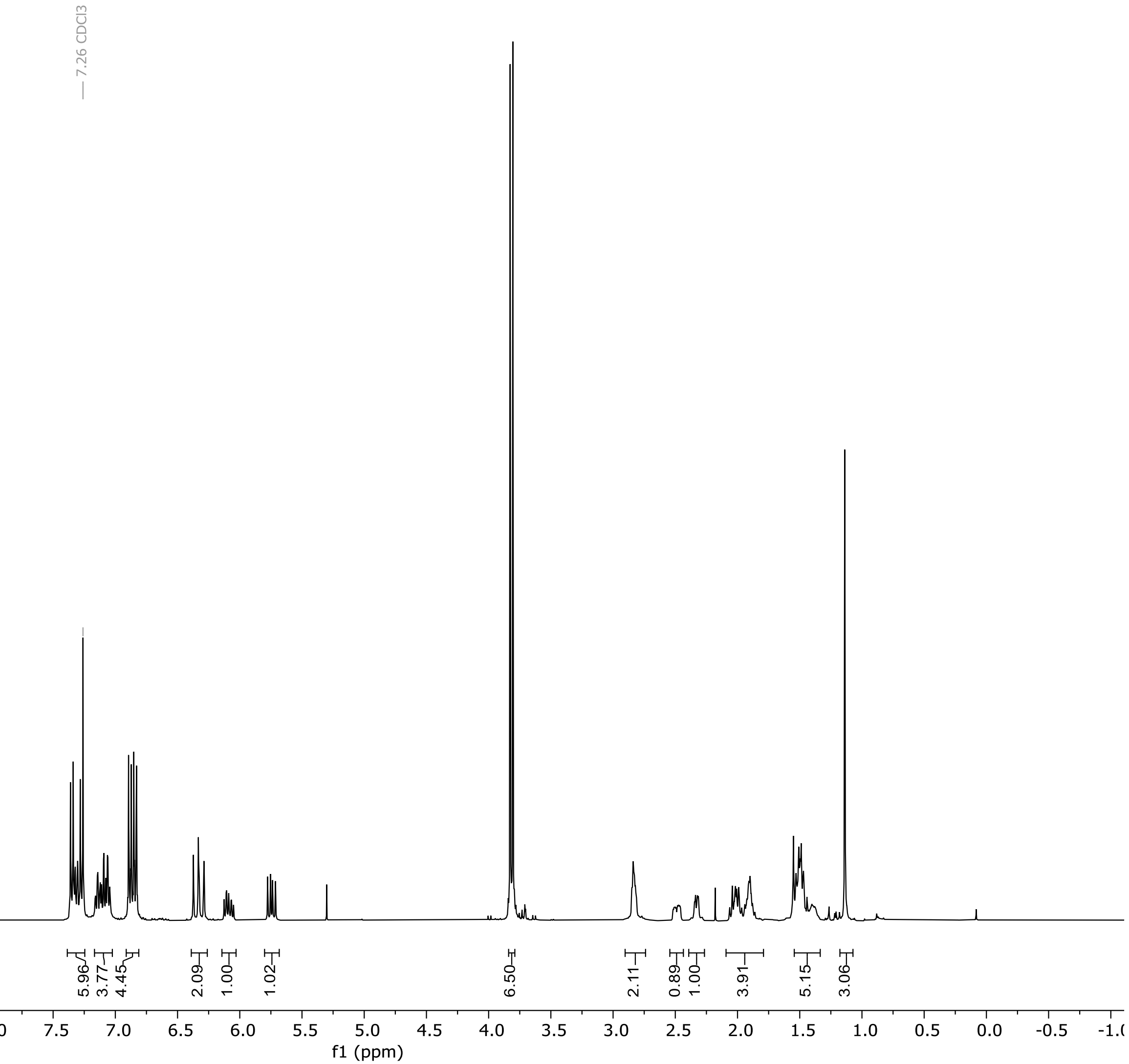

^{13}C NMR (101 MHz, $CDCl_3$)

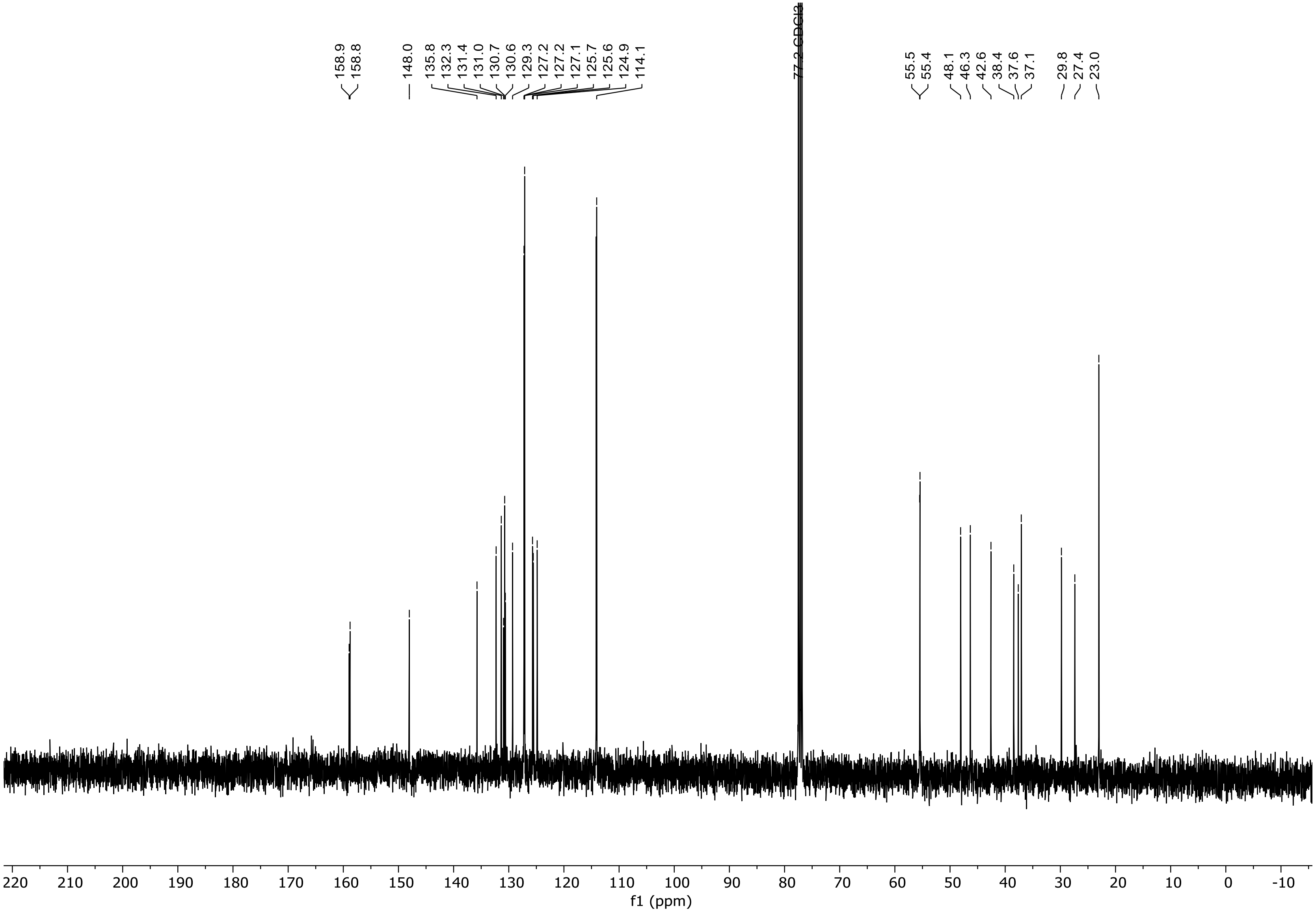

(±)-(1*S*,2*R*,4*aS*,10*aS*)-2-((*E*)-3-(4-methoxyphenyl)allyl)-1-((*E*)-4-methoxystyryl)-1,2,3,4,4*a*,9,10,10*a*-octahydrophenanthrene 6d.

^{1}H NMR (400 MHz, $CDCl_3$)

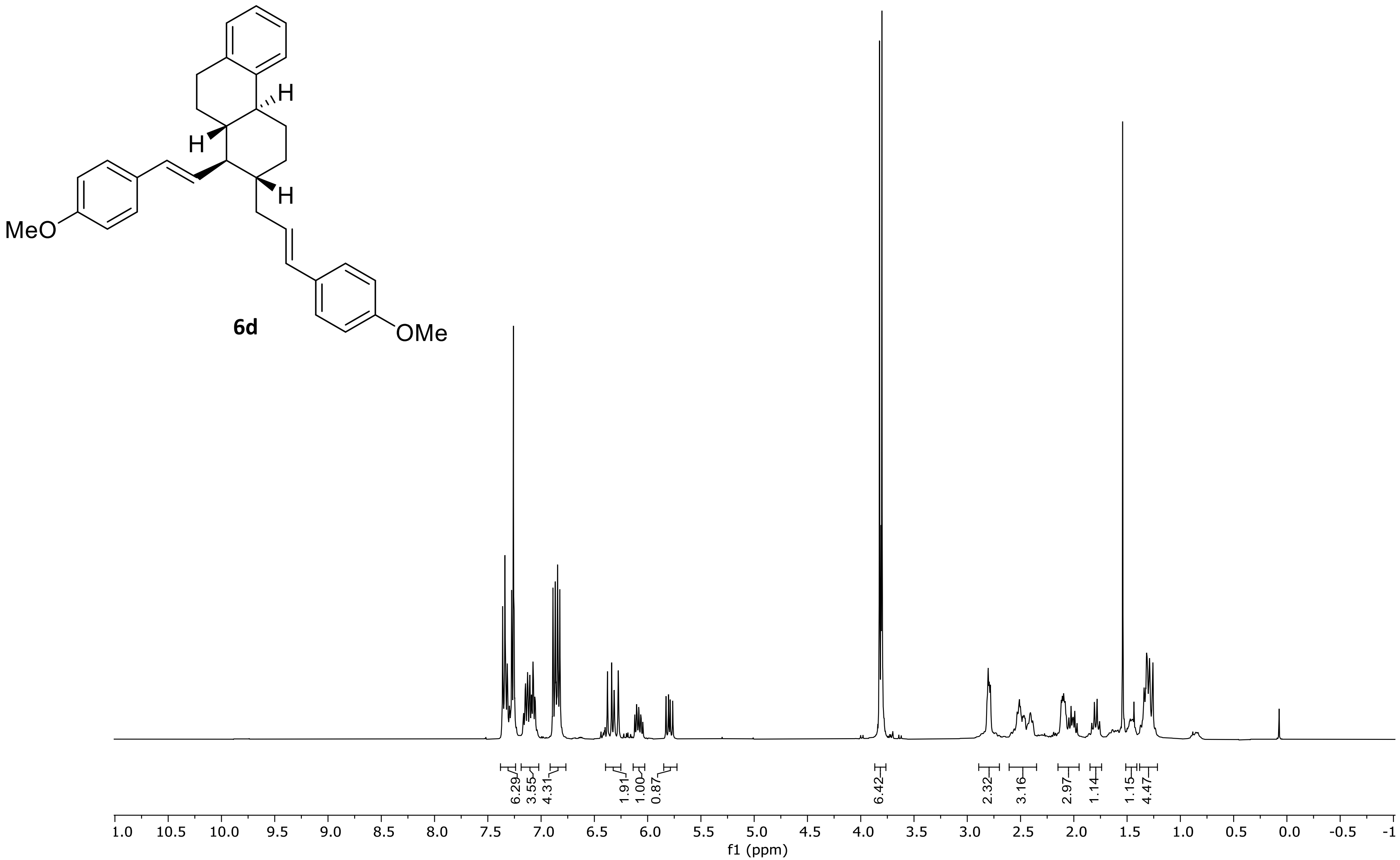

¹³C NMR (101 MHz, $CDCl_3$)

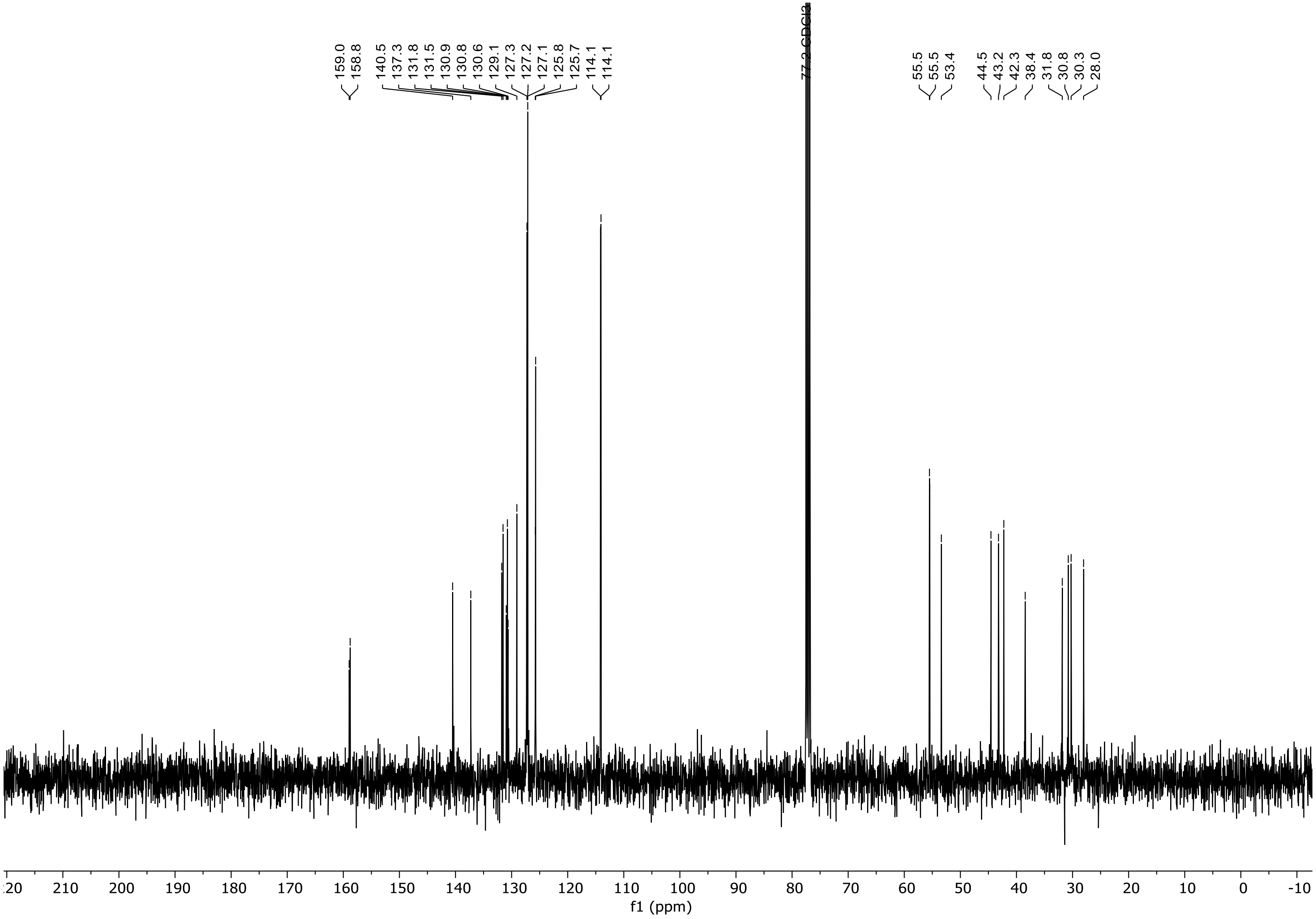

(±)-(1*S*,2*R*,4*aS*,10*aS*)-2-(4-methoxybenzyl)-1-((*E*)-4-methoxystyryl)-4*a*-methyl-1,2,3,4,4*a*,9,10,10*a*-octahydrophenanthrene 6e.

^{1}H NMR (400 MHz, $CDCl_3$)

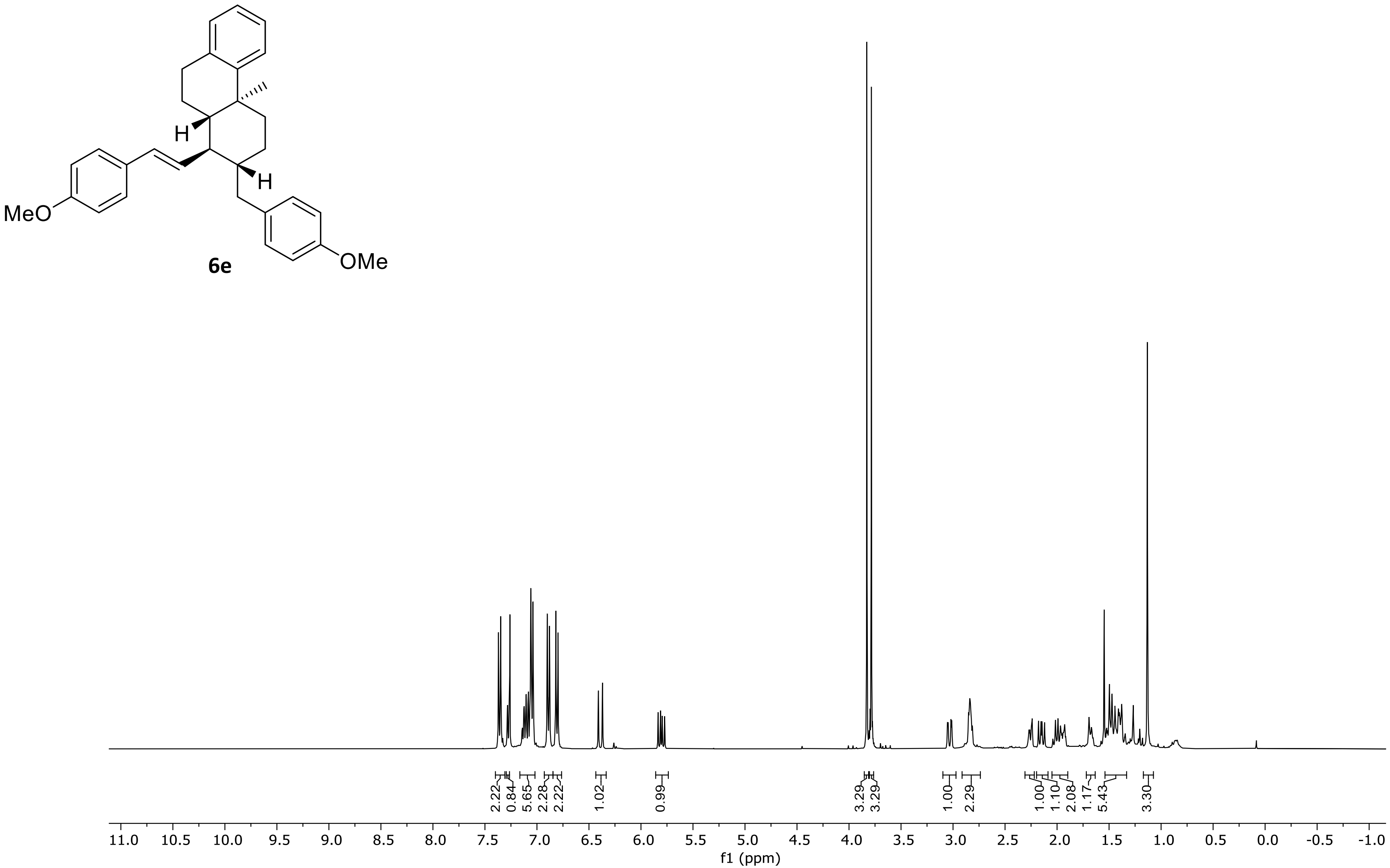

6e

^{13}C NMR (101 MHz, $CDCl_3$)

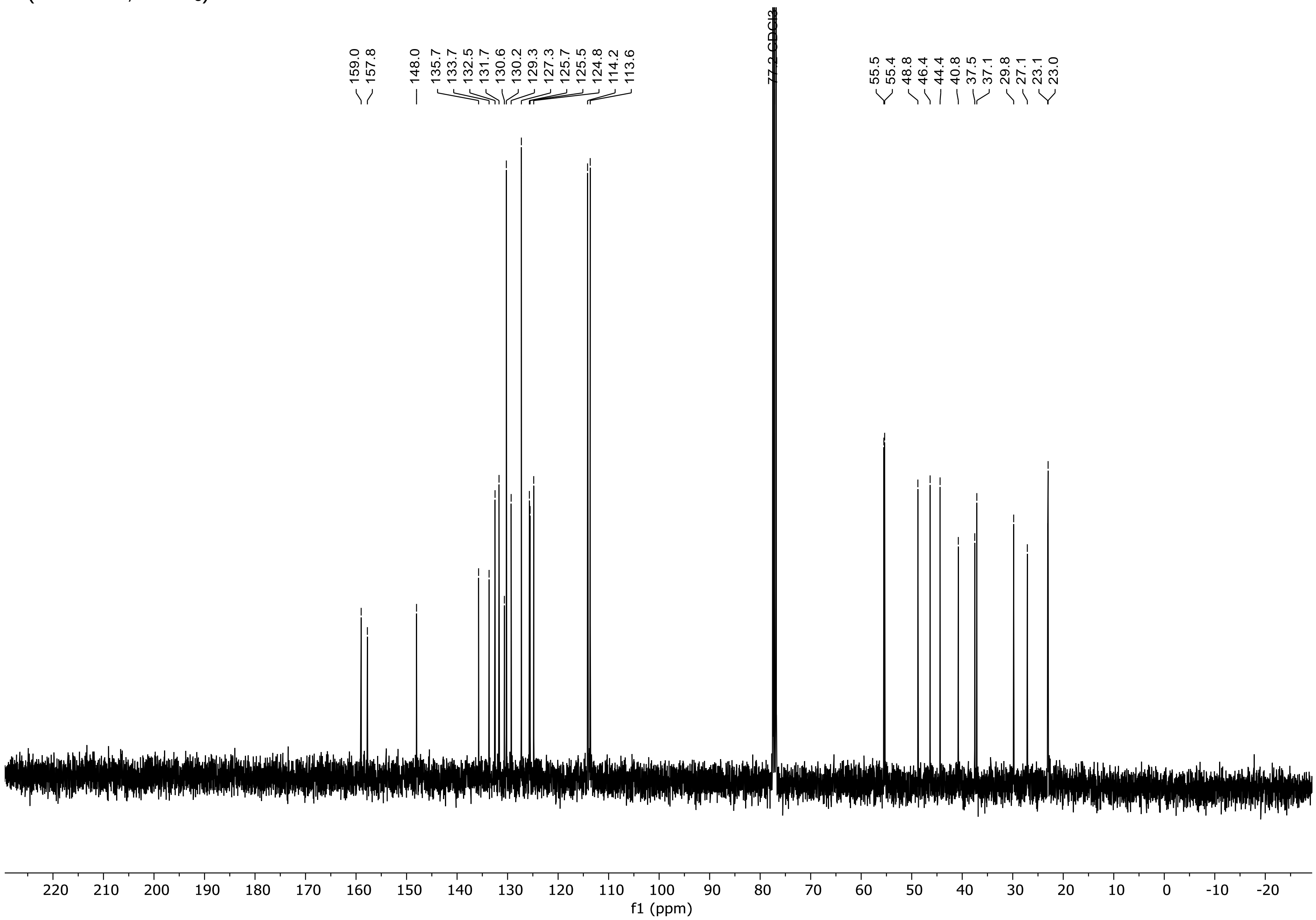

NOESY-2D (600 MHz, $CDCl_3$)

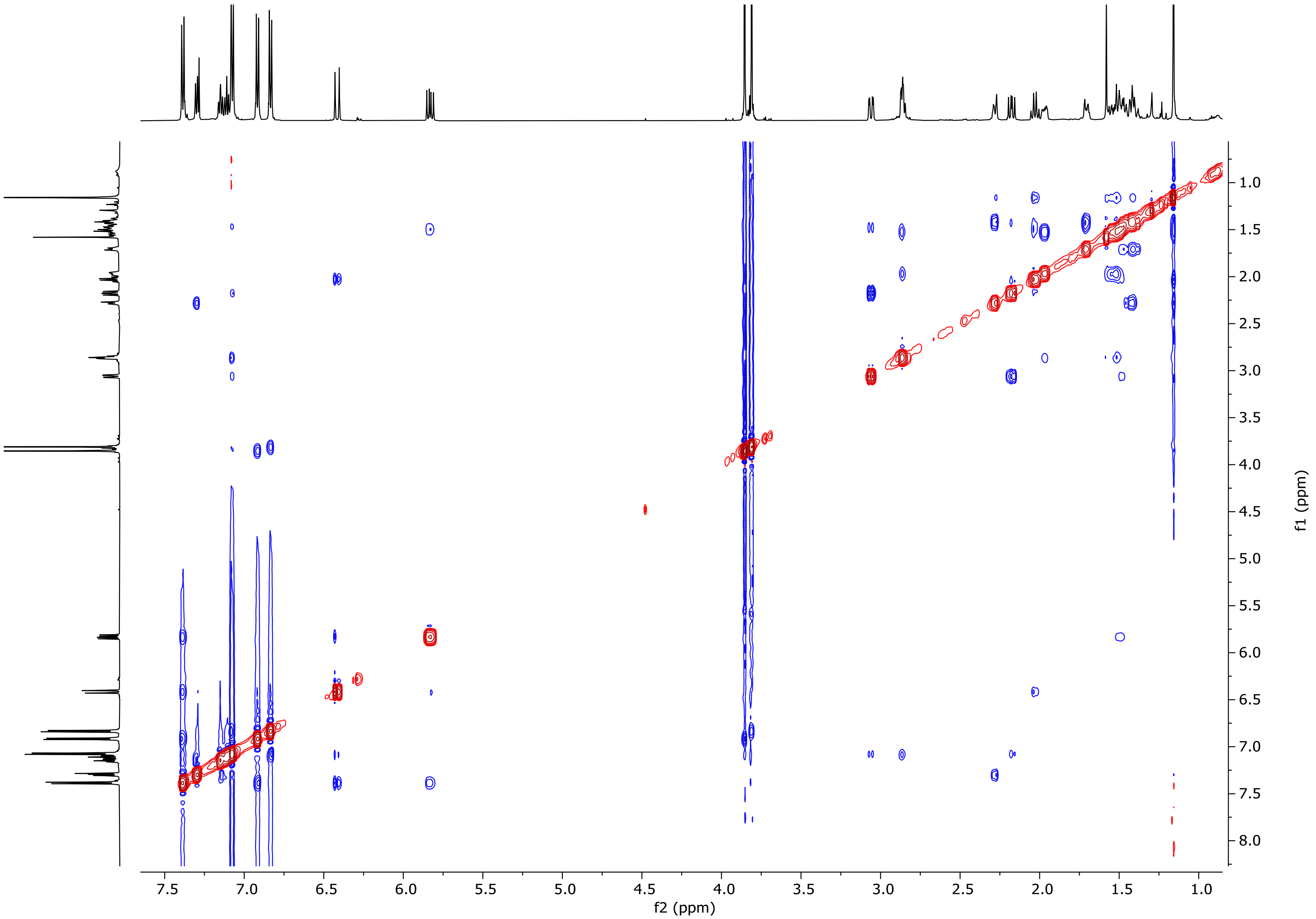

(±)-3-(((1*S*,2*R*,4*aS*,10*aS*)-1-((*E*)-4-methoxystyryl)-4*a*-methyl-1,2,3,4,4_a,9,10,10*a*-octahydrophenanthren-2-yl)methyl)-1-methyl-1H-indole 6f.

^{1}H NMR (400 MHz, $CDCl_3$)

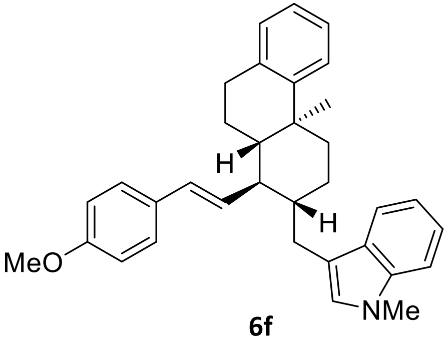

6f

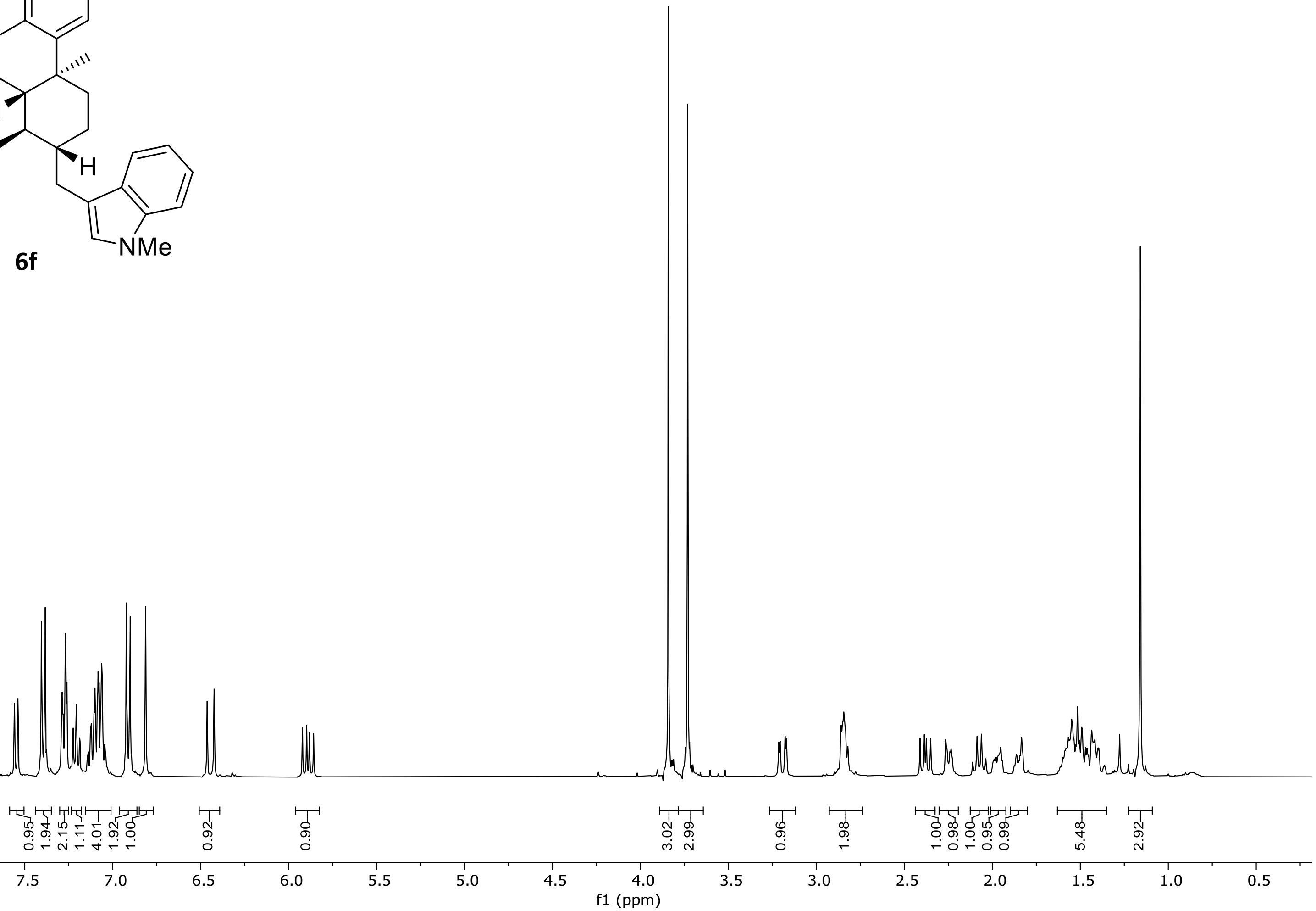

^{13}C NMR (101 MHz, $CDCl_3$)

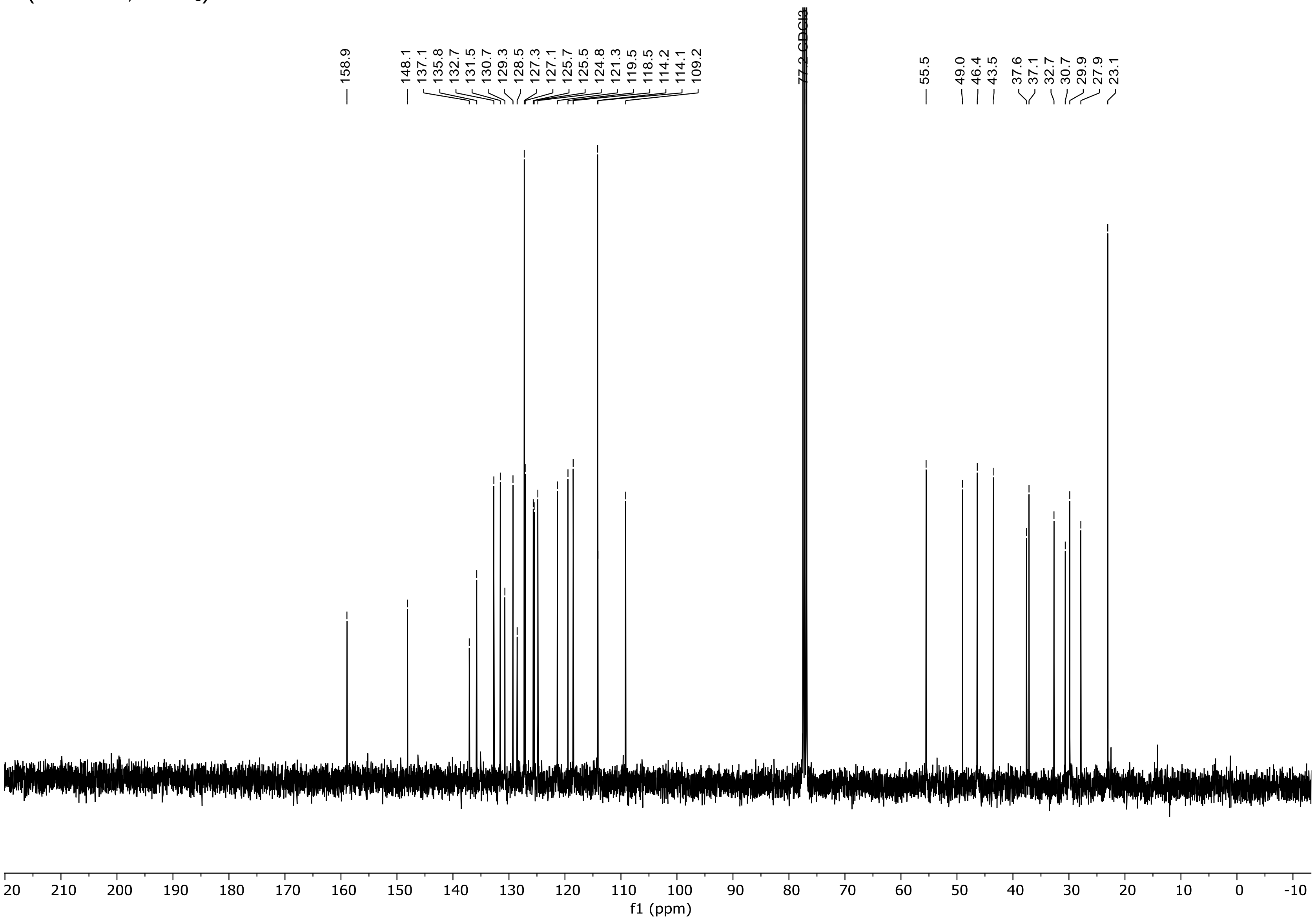

NOESY-2D (600 MHz, $CDCl_3$)

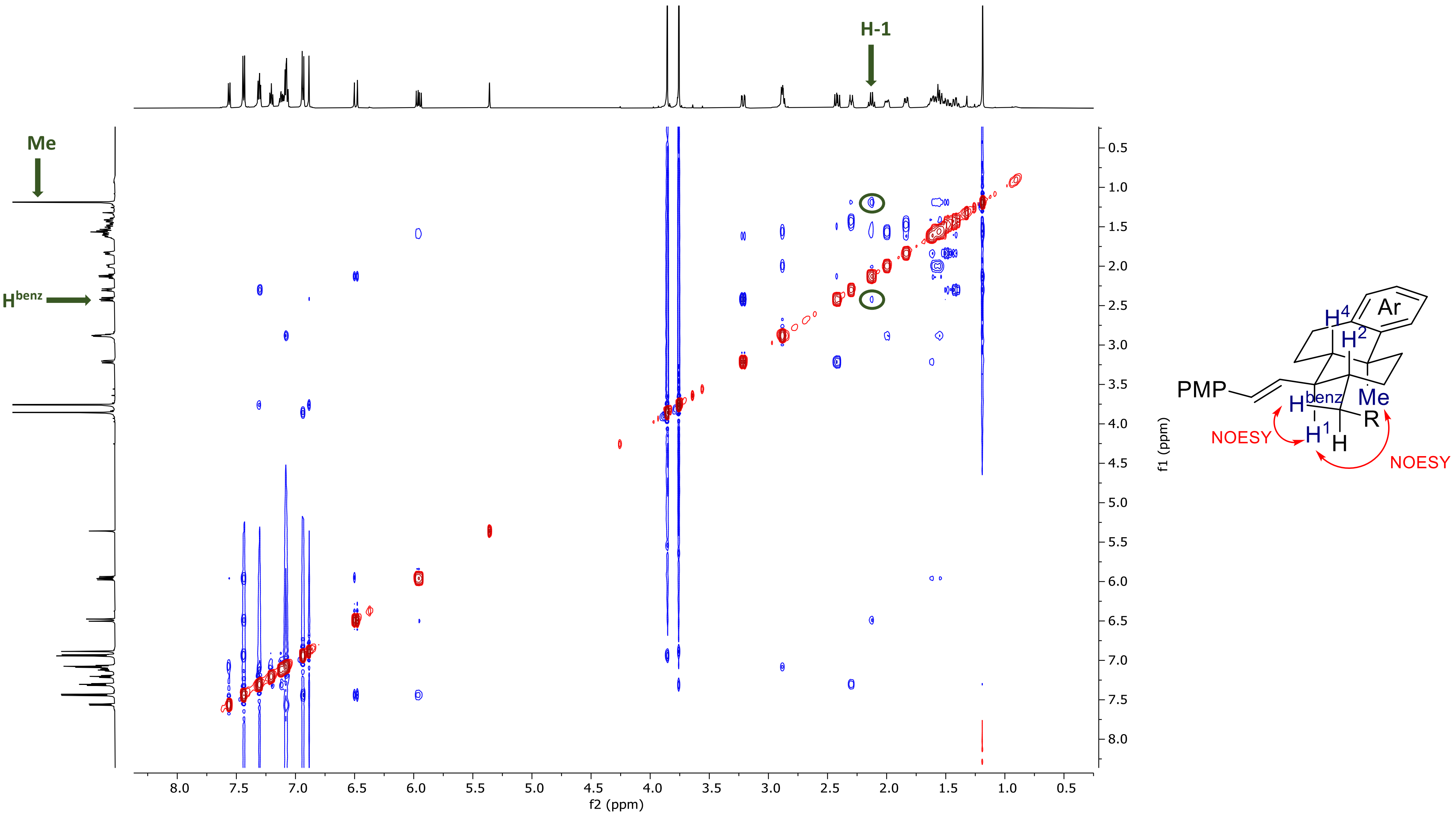

7.4 Products ***12-16***

(±)-(1*R*,2*S*,4*aS*,10*aS*)-2-benzhydryl-6-methoxy-1-(4-methoxyphenyl)-4*a*-methyl-1,2,3,4,4*a*,9,10,10*a*-octahydrophenanthrene 12.

^{1}H NMR (400 MHz, $CDCl_3$)

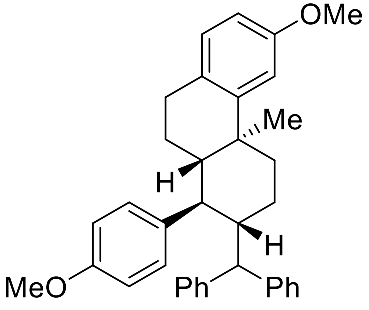

12

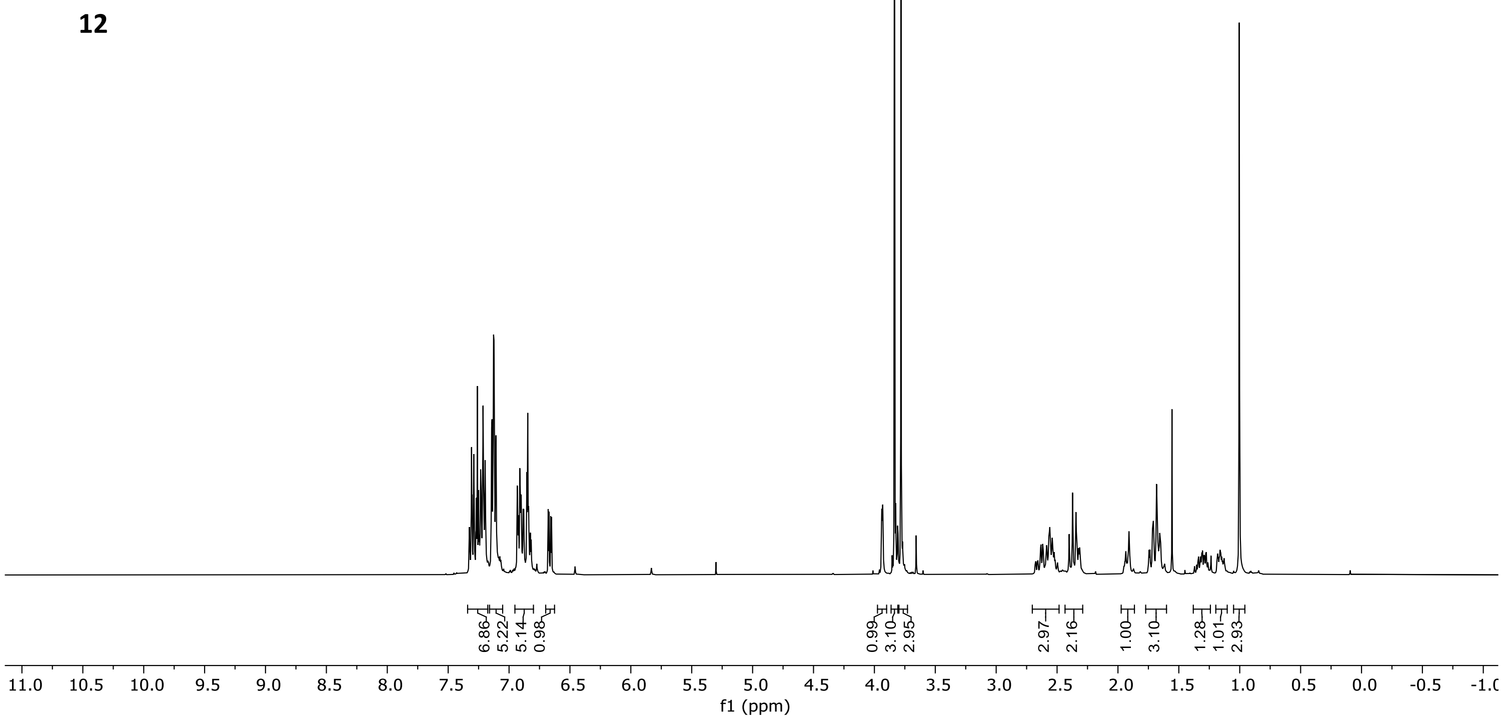

^{13}C NMR (101 MHz, $CDCl_3$)

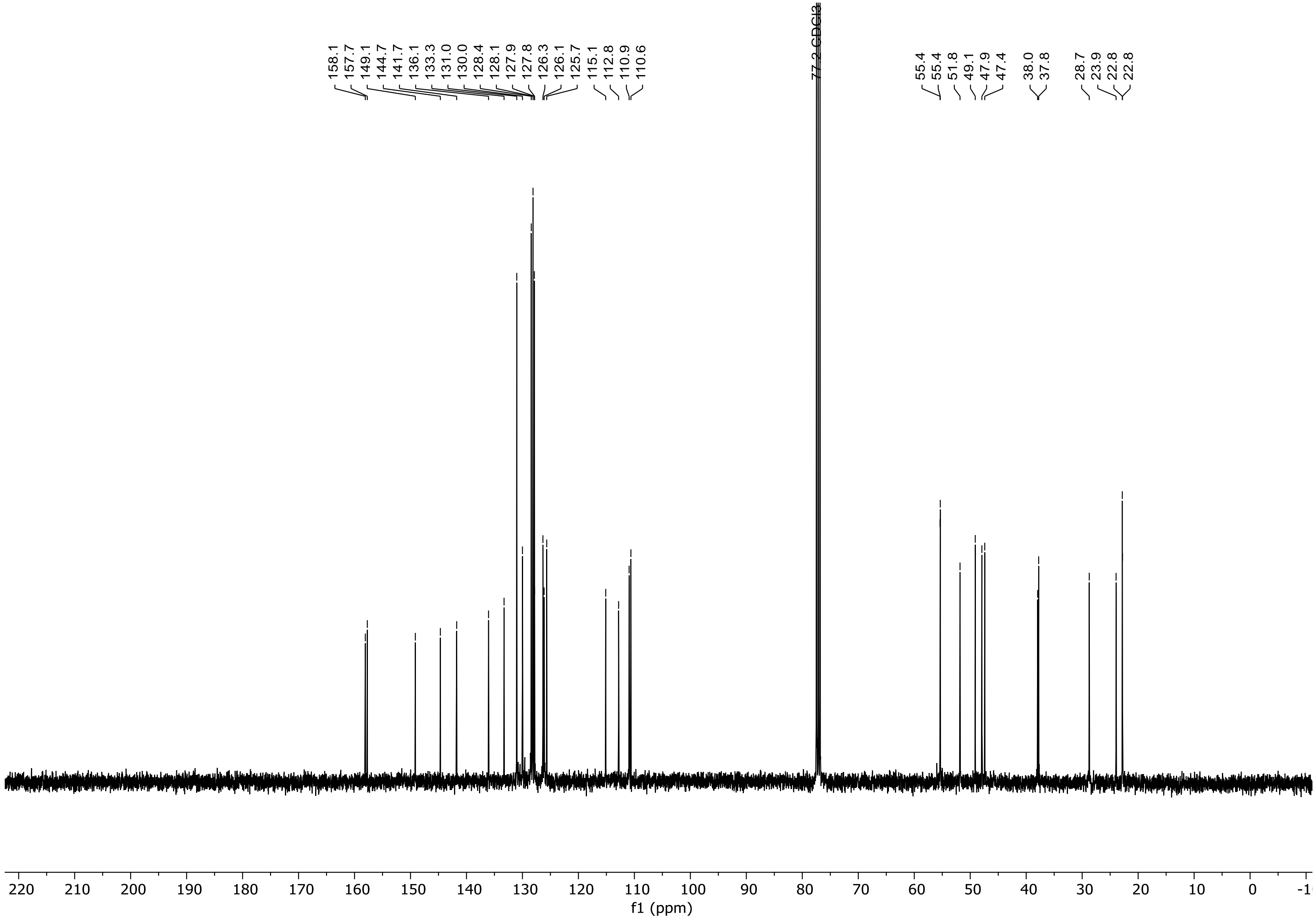

NOESY-2D (600 MHz, $CDCl_3$)

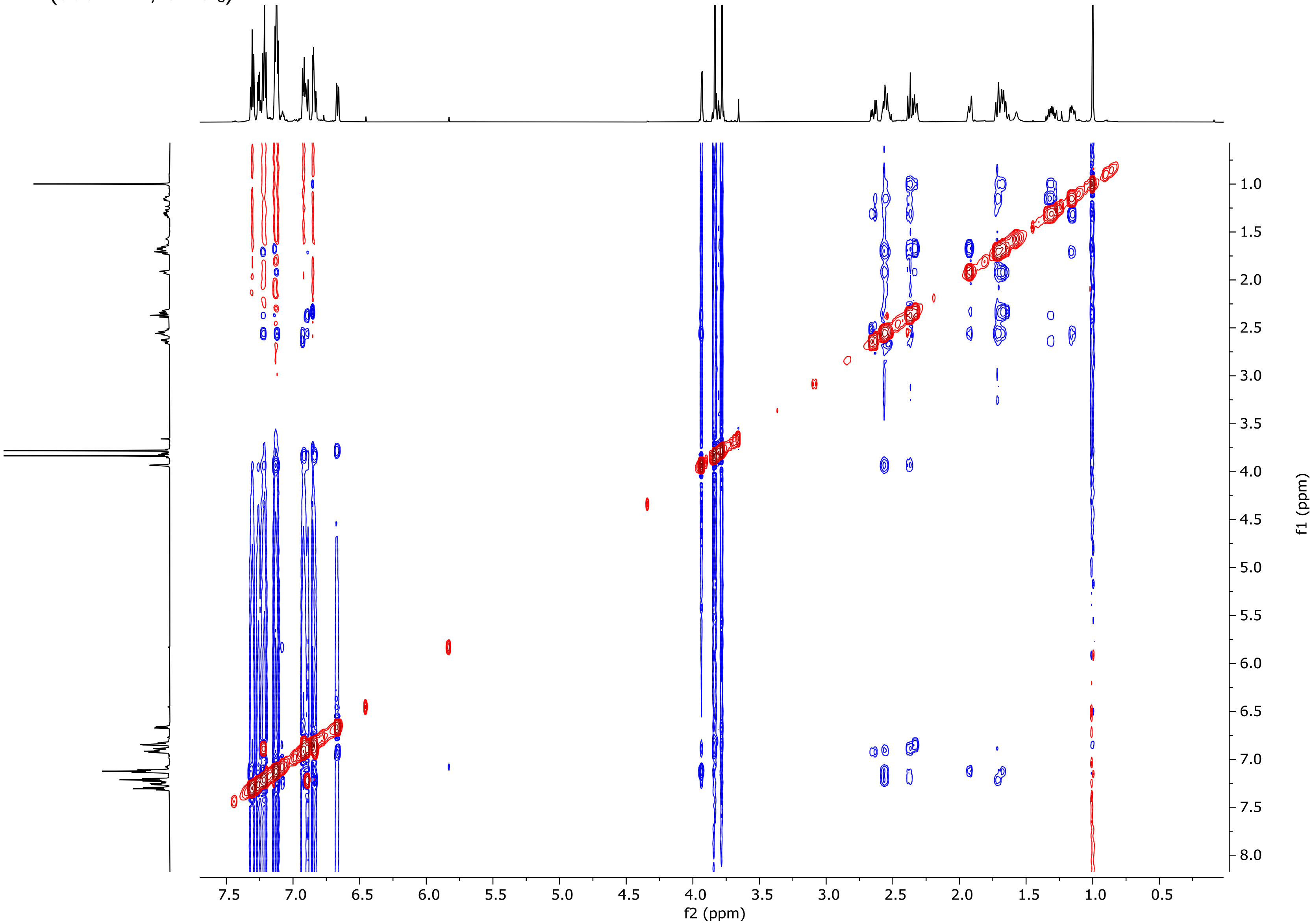

(±)-(1*R*,2*S*,4*aS*,10*aS*)-2-benzhydryl-5,8-dimethoxy-1-(4-methoxyphenyl)-4*a*-methyl-1,2,3,4,4*a*,9,10,10*a*-octahydrophenanthrene 13.

^{1}H NMR (400 MHz, $CDCl_3$)

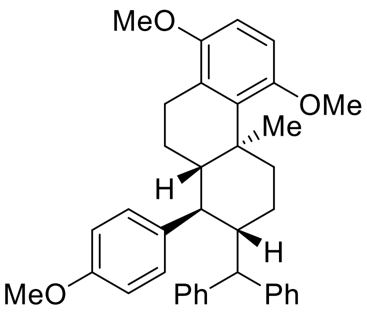

13

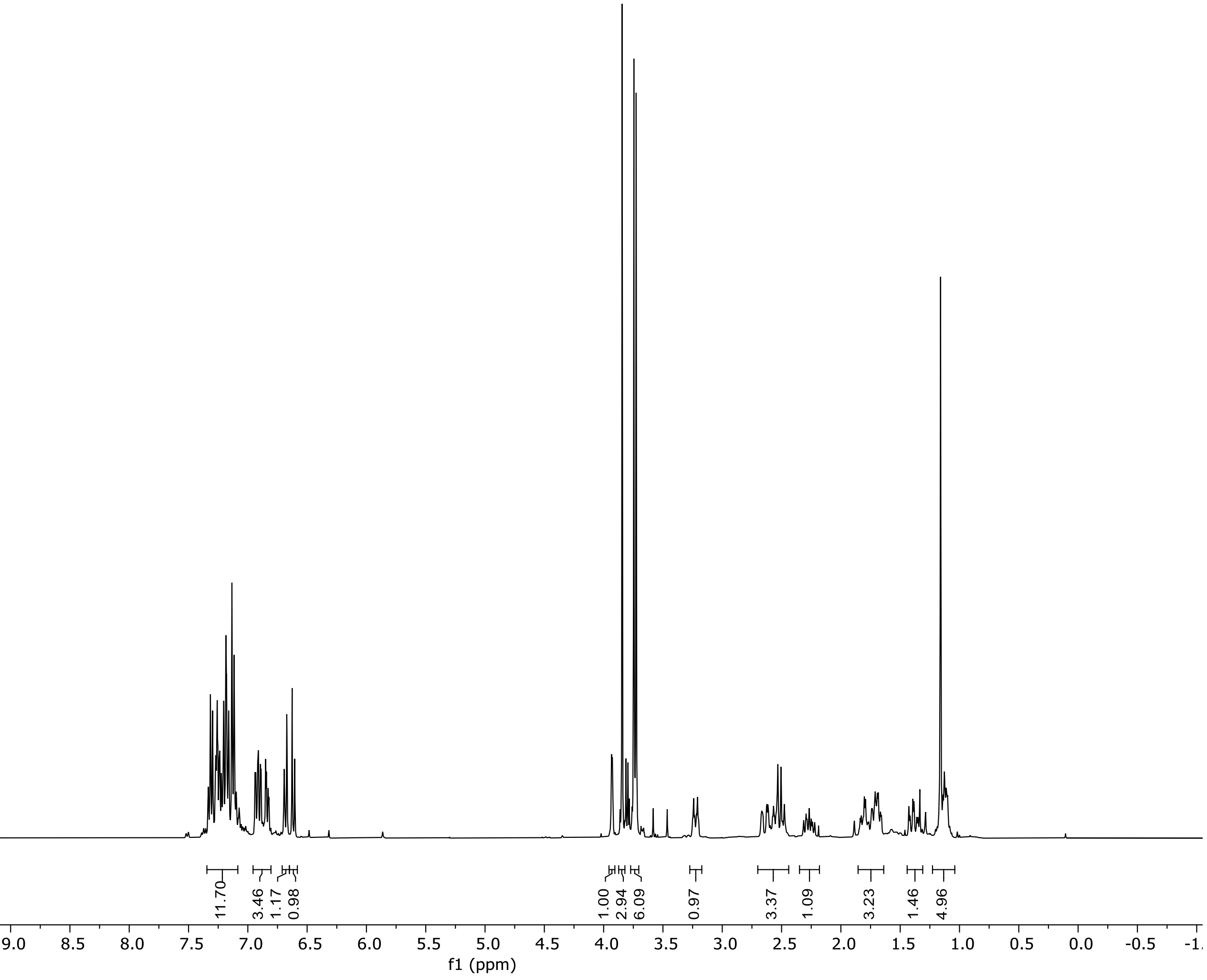

^{13}C NMR (101 MHz, $CDCl_3$)

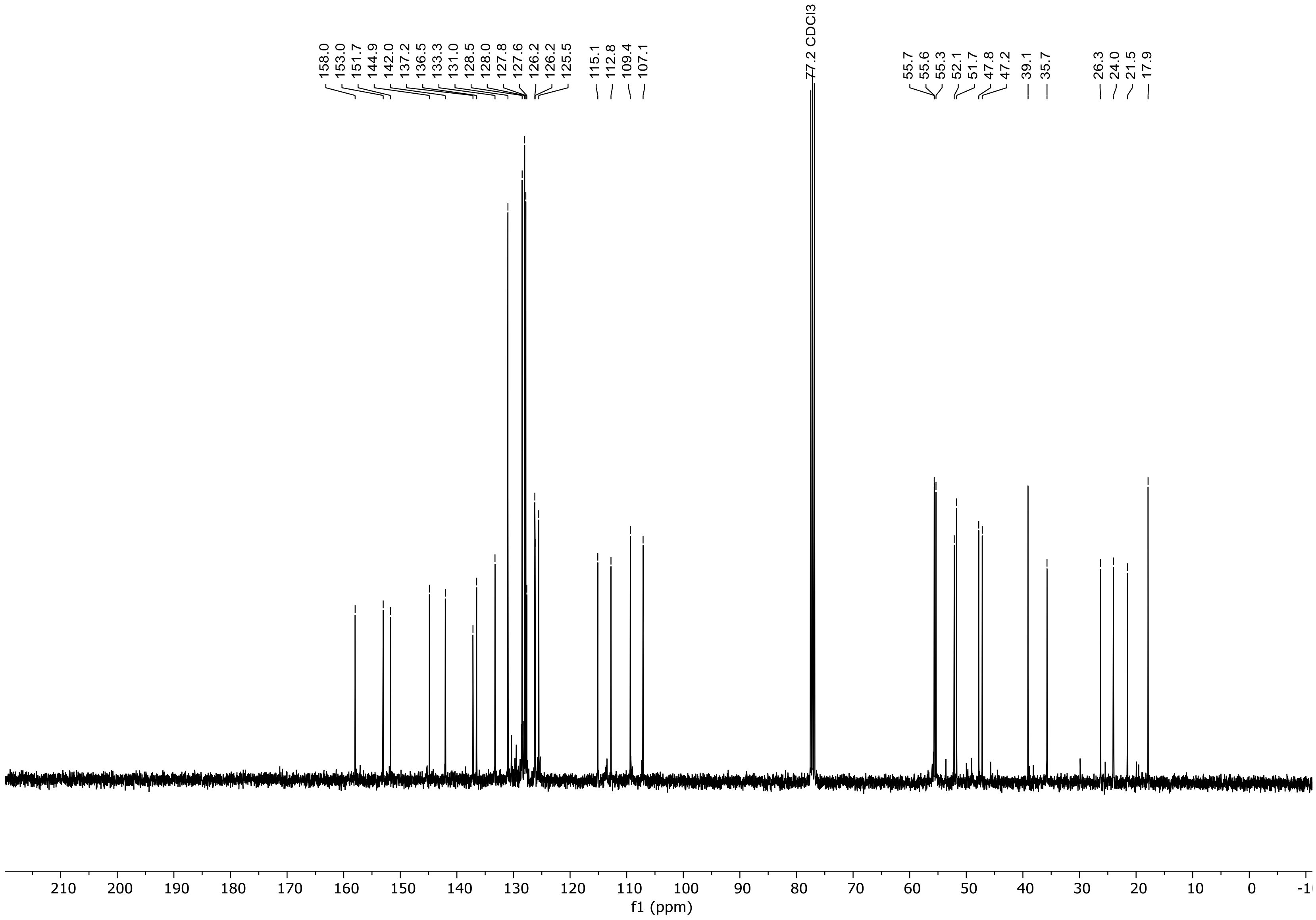

(±)-(1*R*,2*S*,4*aS*,10*aS*)-2-benzhydryl-1-(4-methoxyphenyl)-4*a*,6-dimethyl-1,2,3,4,4*a*,9,10,10*a*-octahydrophenanthrene 14.

[1]**H NMR** (400 MHz, $CDCl_3$)

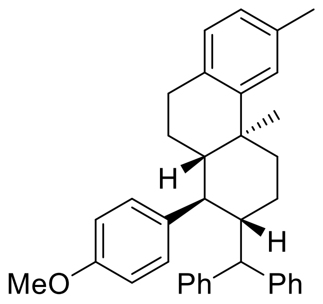

14

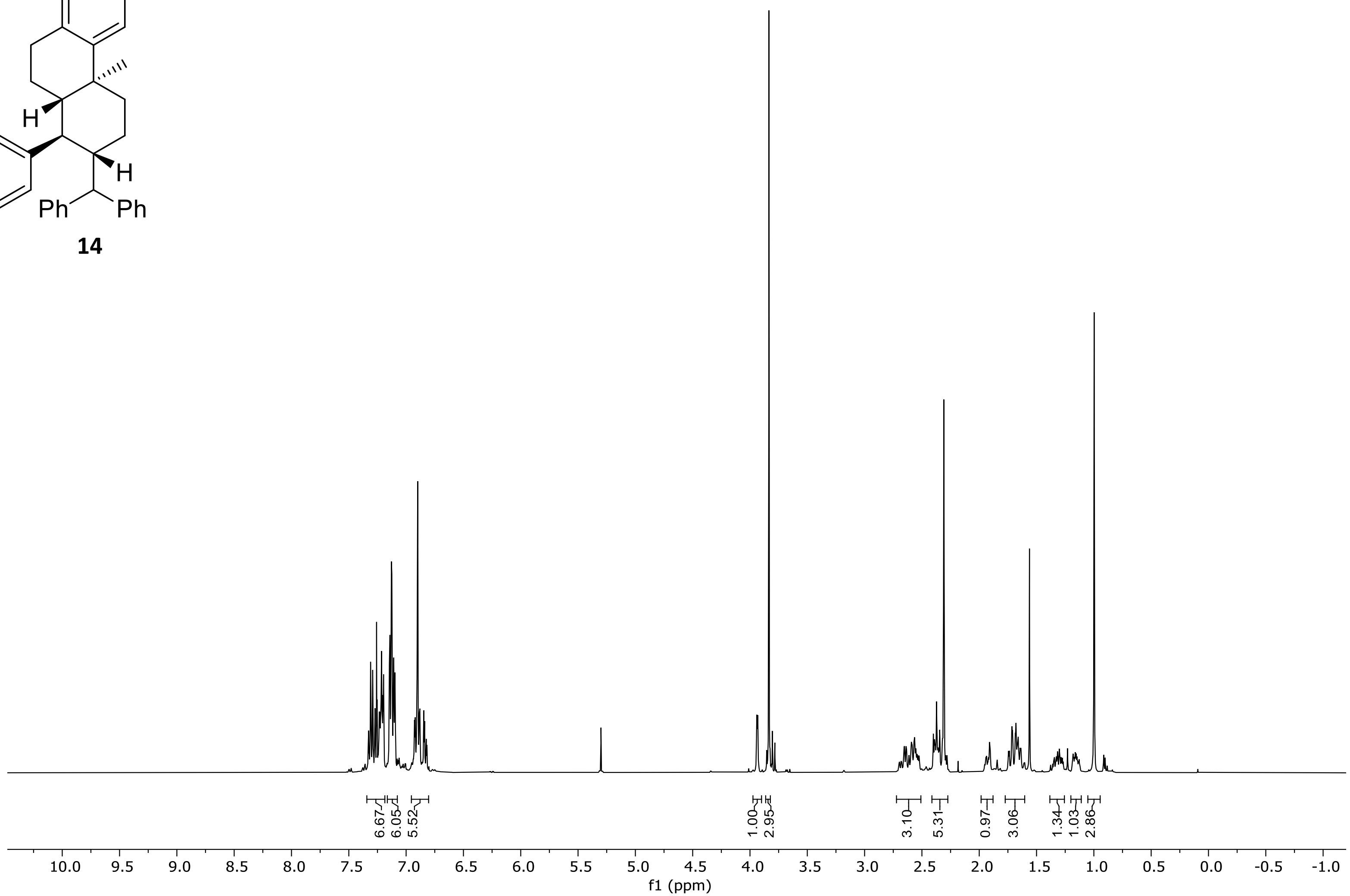

^{13}C NMR (101 MHz, $CDCl_3$)

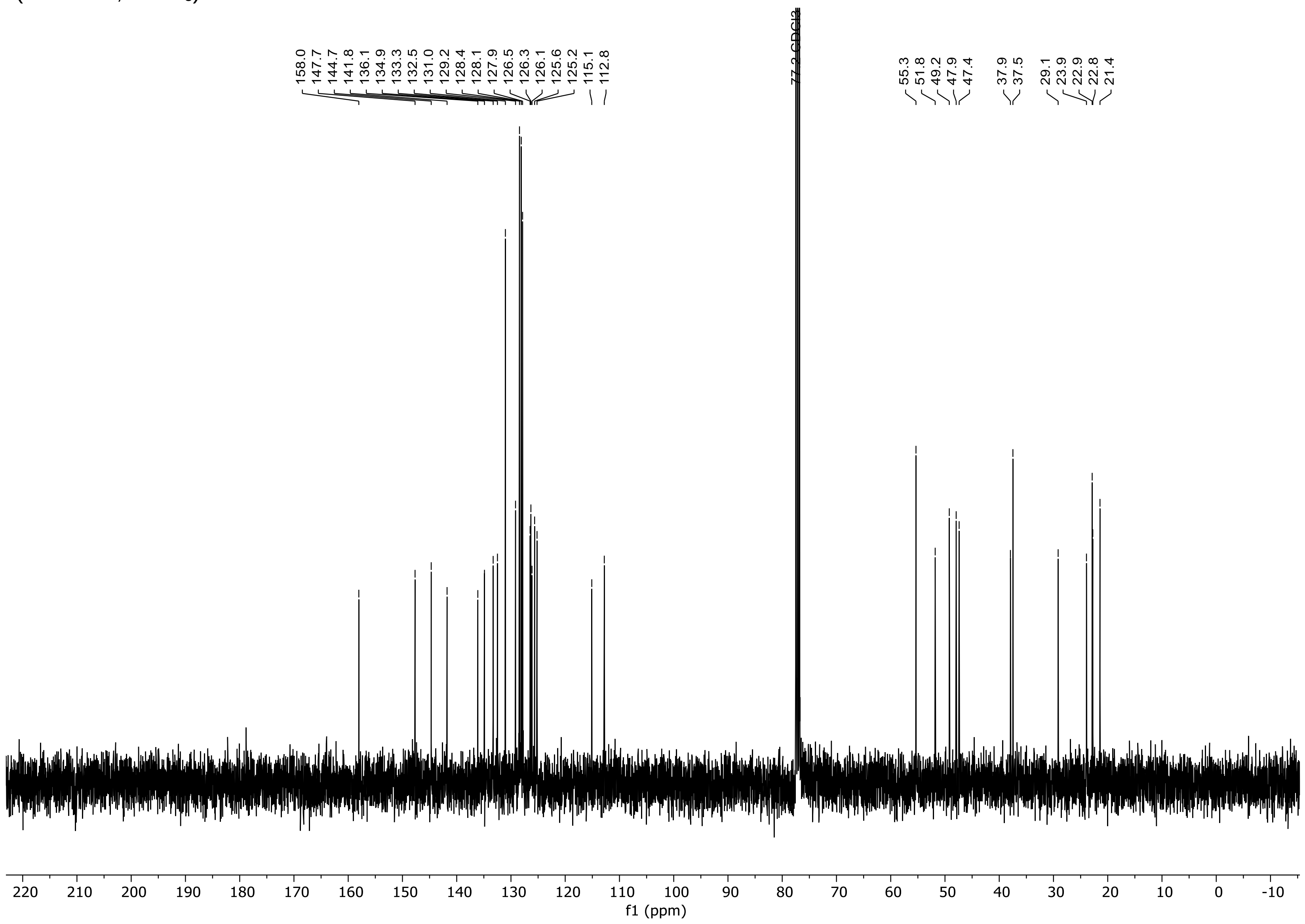

Analysis for (±)-(1*R*,2*S*,4*aS*,10*aS*)-2-benzhydryl-6-chloro-1-(4-methoxyphenyl)-4*a*-methyl-1,2,3,4,4*a*,9,10,10*a*-octahydrophenanthrene 15.

^{1}H NMR (600 MHz, CD_2Cl_2)

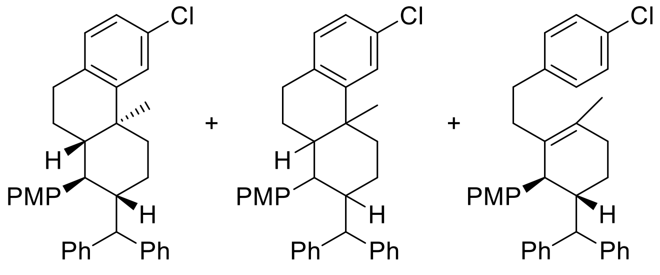

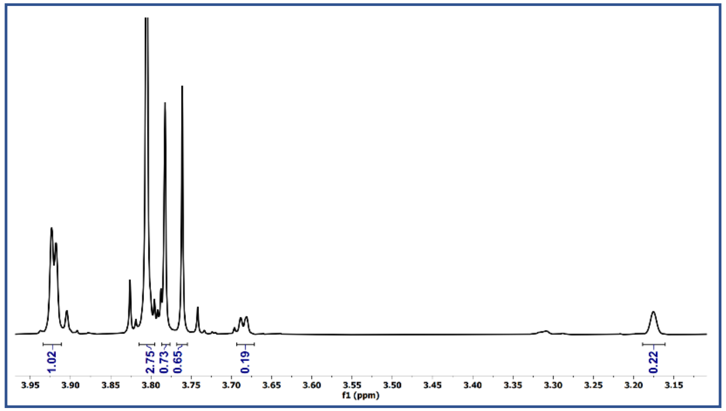

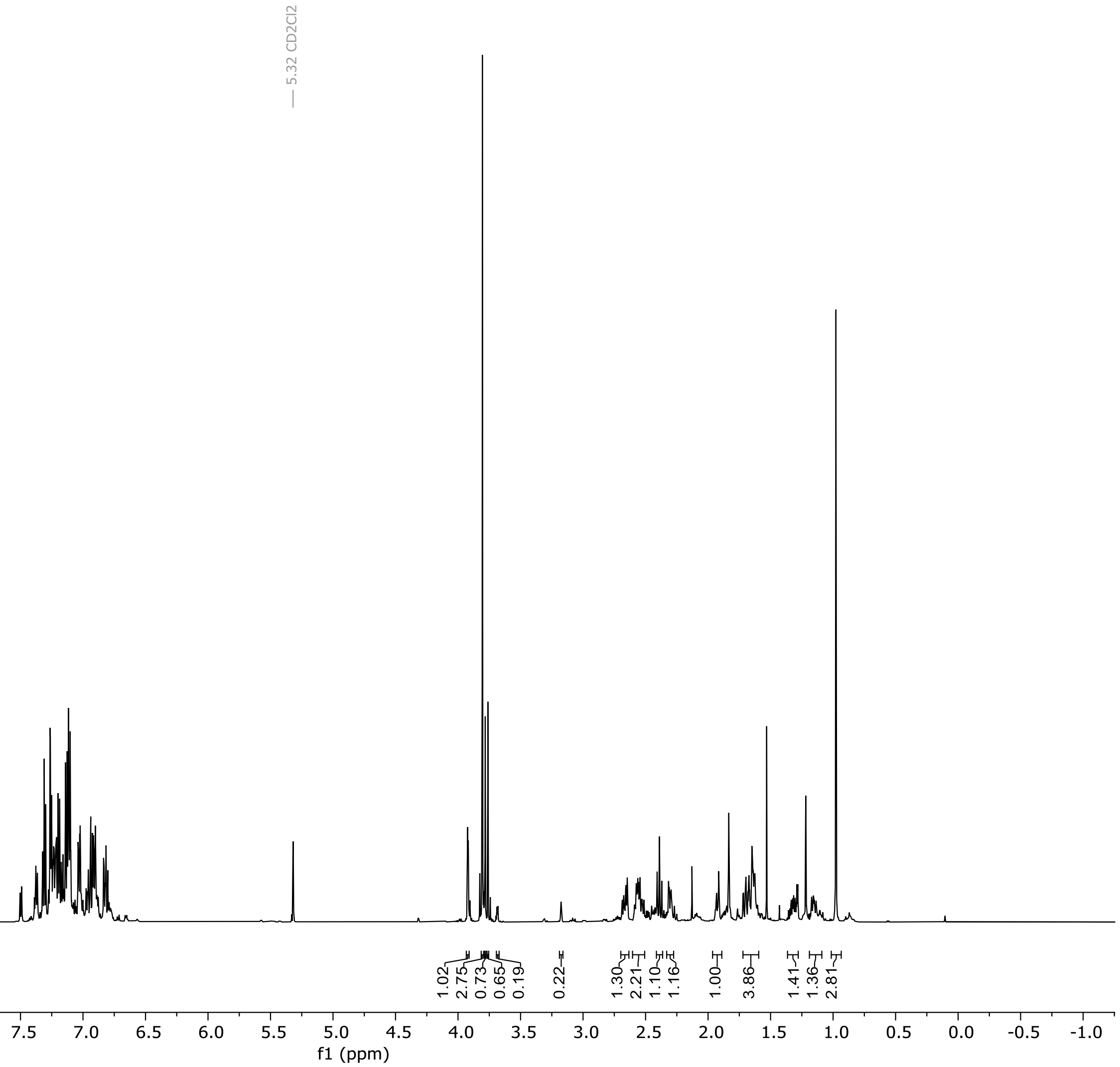

^{13}C NMR (101 MHz, CD_2Cl_2)

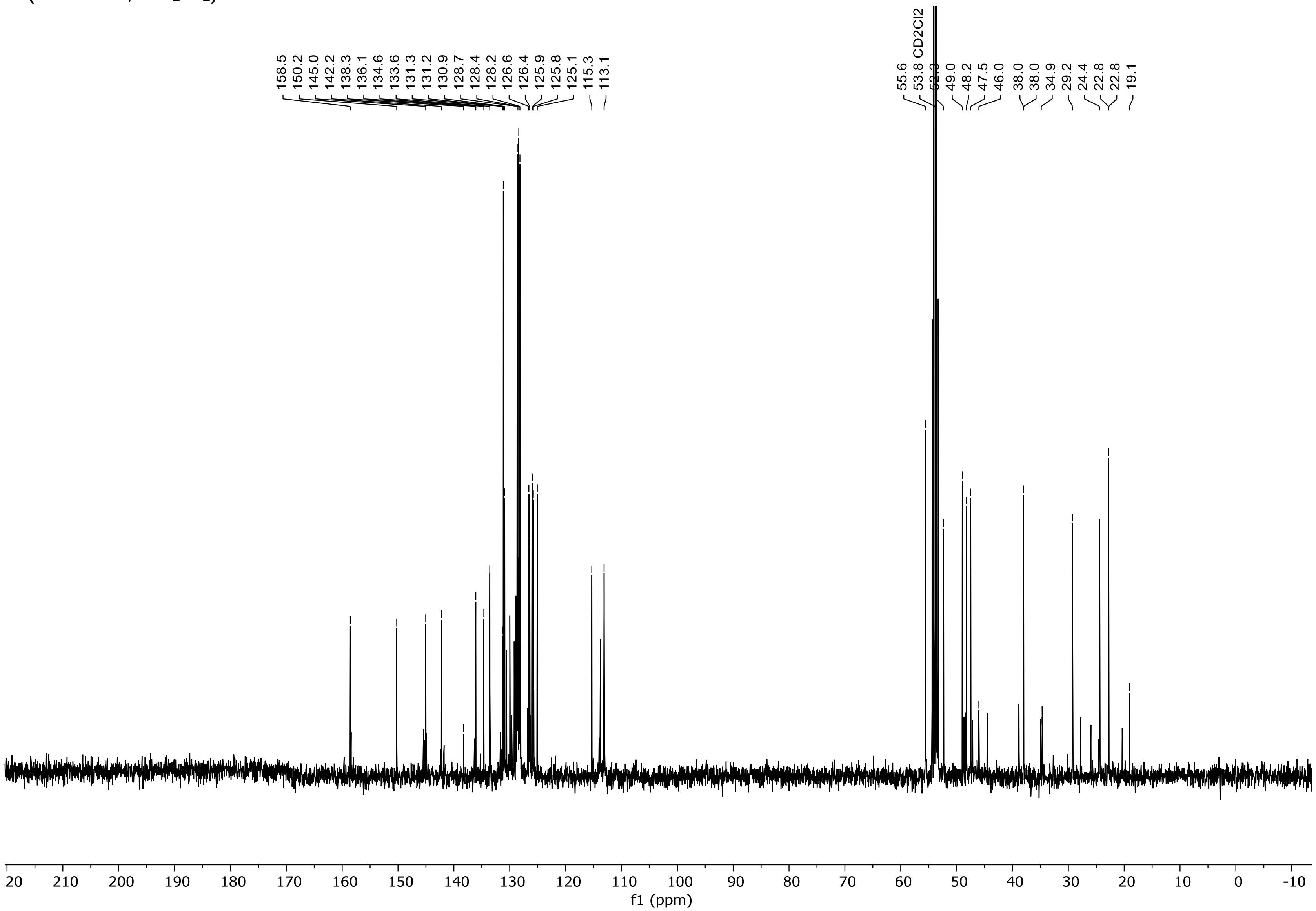

(±)-(1*R*,2*S*)-2-Benzhydryl-4'-methoxy-5-methyl-1,2,3,4-tetrahydro-1,1'-biphenyl (Major) 16 and (±)- (1*S*,2*S*)-2-Benzhydryl-4'-methoxy-5-methyl-1,2,3,6-tetrahydro-1,1'-biphenyl (Minor) 16'.

^{1}**H NMR** (400 MHz, $CDCl_3$)

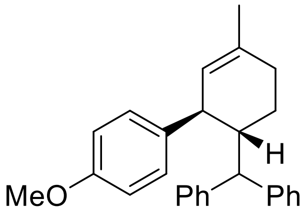

16

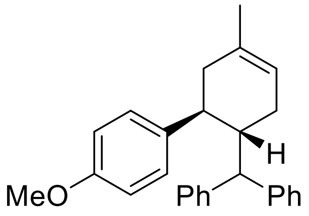

16'

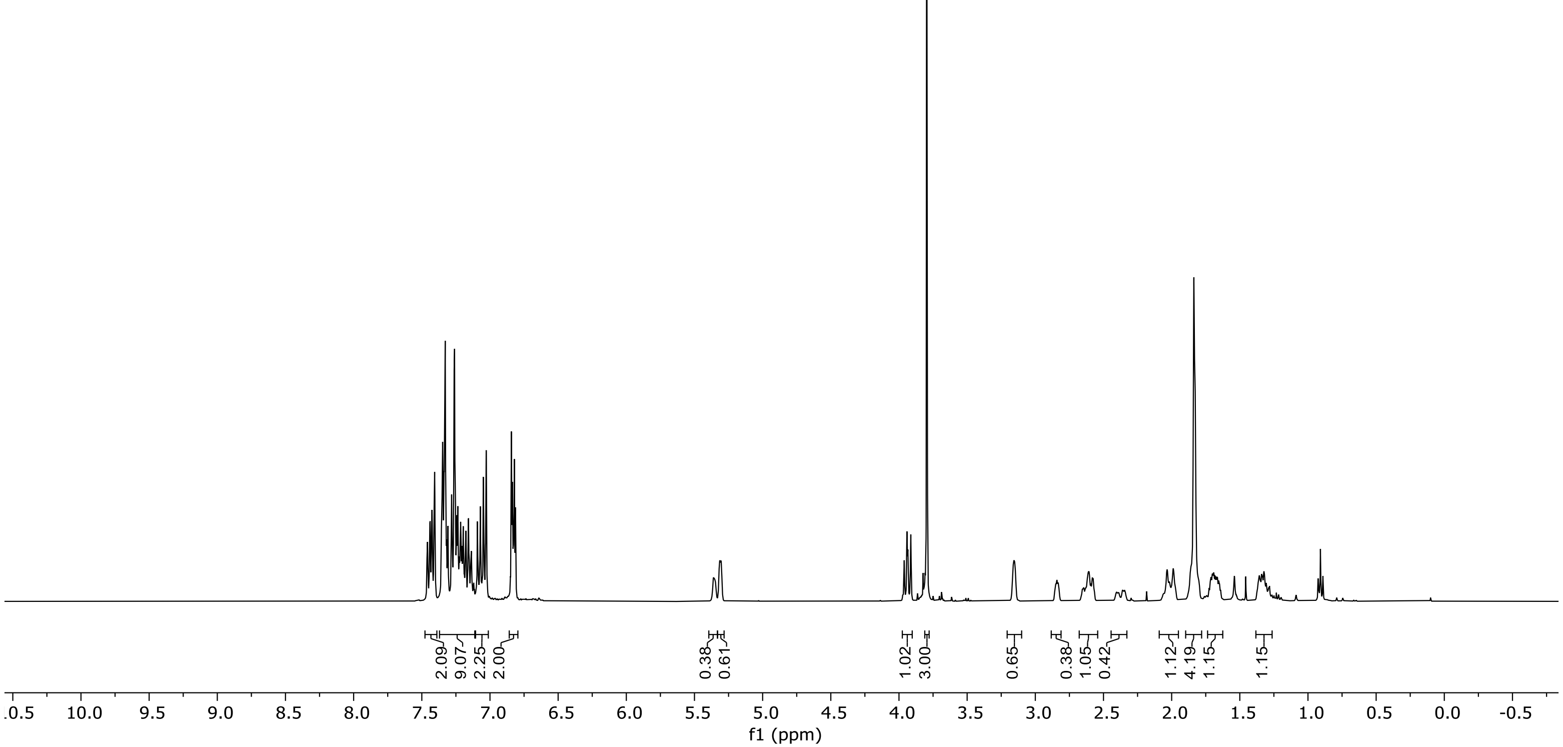

^{13}C NMR (101 MHz, $CDCl_3$)

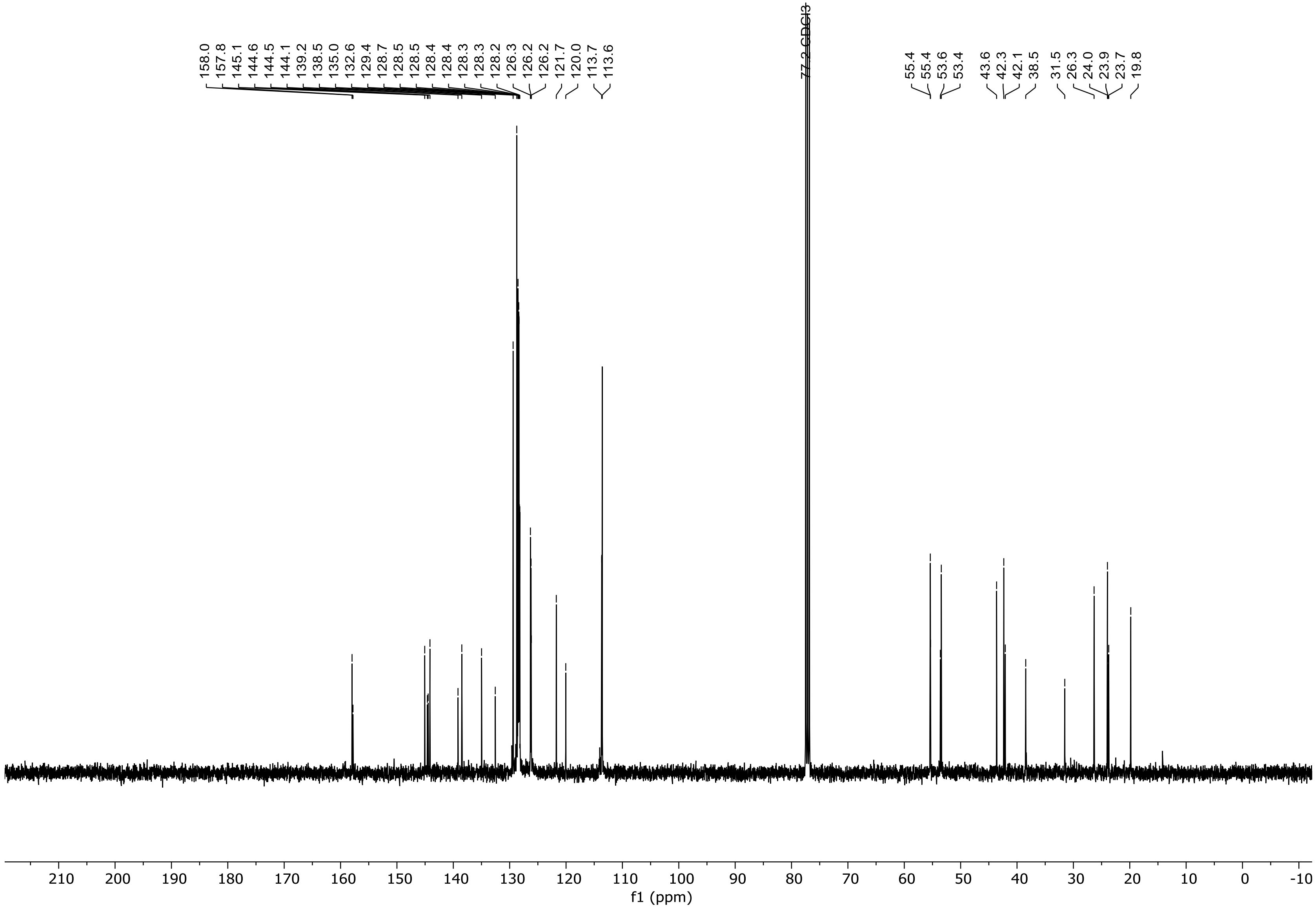

NOESY (600 MHz, $CDCl_3$)

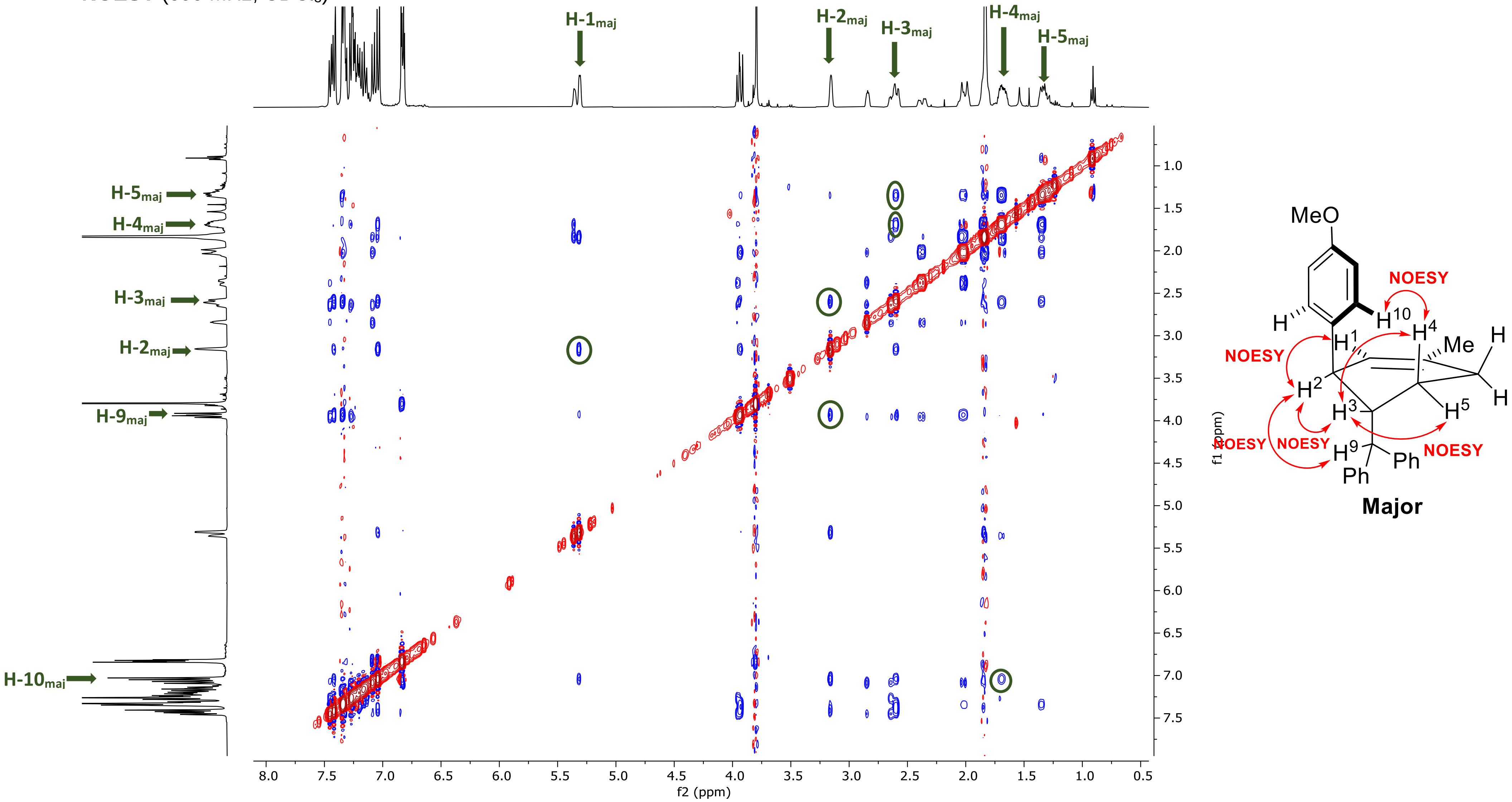

^{1}H (400 MHz)-^{13}C (101 MHz) **HSQC-2D** ($CDCl_3$)

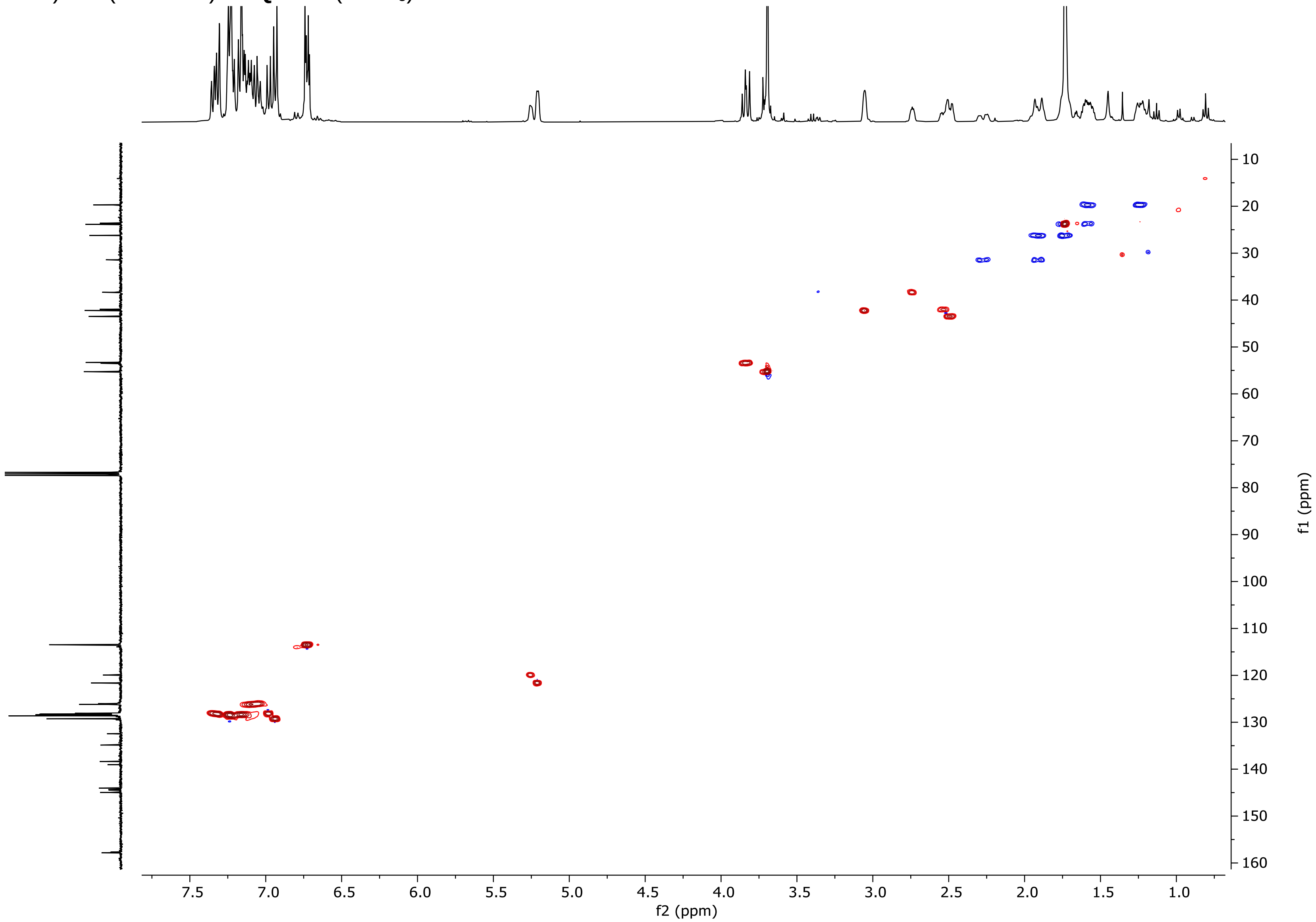

*7.5 Derivatisation: products **17** and **18**.*

(±)-(8*S*,9*S*,14*S*)-7,8,9,11,12,14,15,16-octahydro-6H-cyclopenta[*a*]phenanthrene 17.

^{1}H NMR (400 MHz, $CDCl_3$)

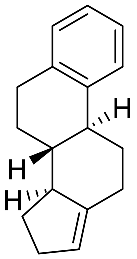

17

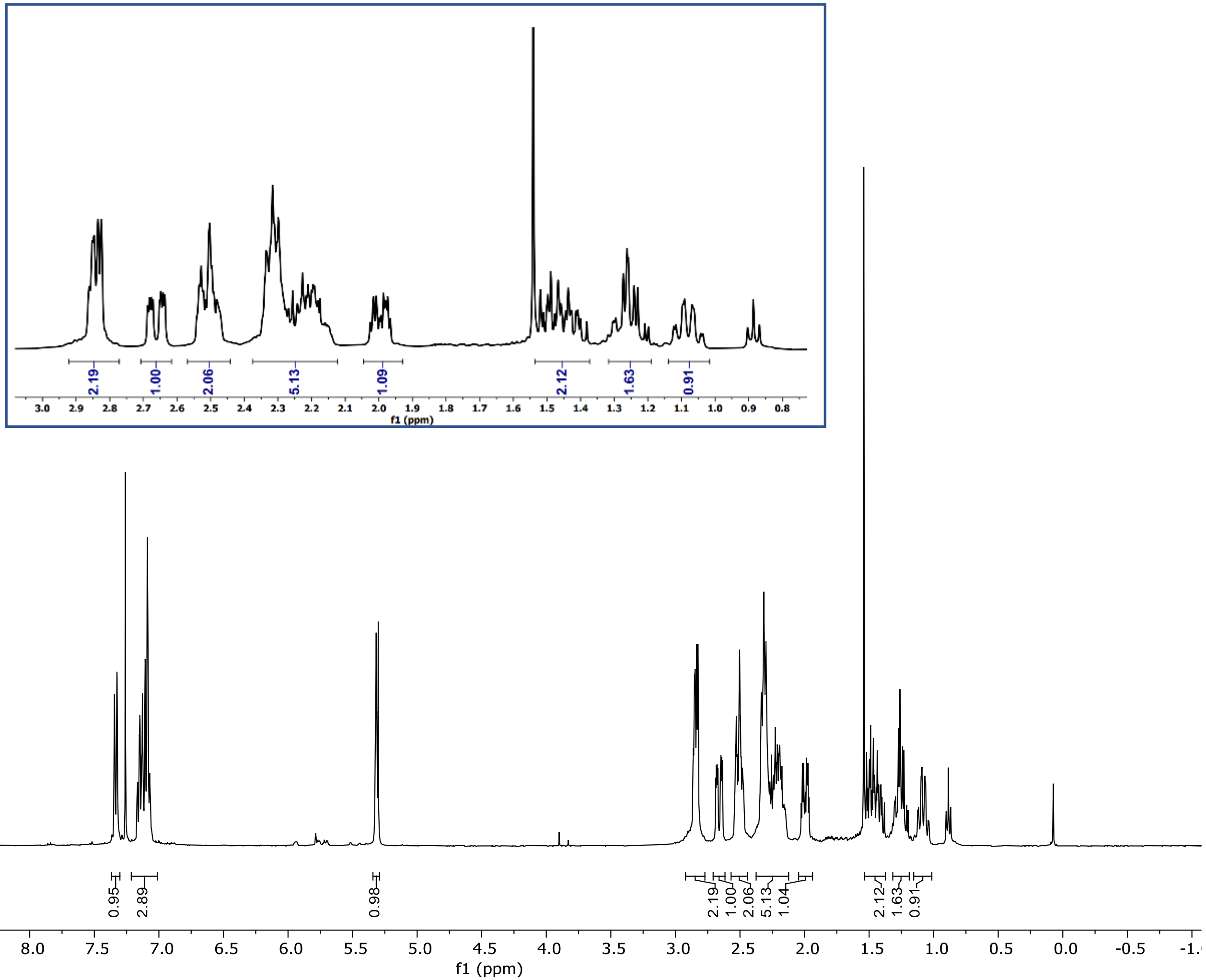

^{13}C NMR (101 MHz, $CDCl_3$)

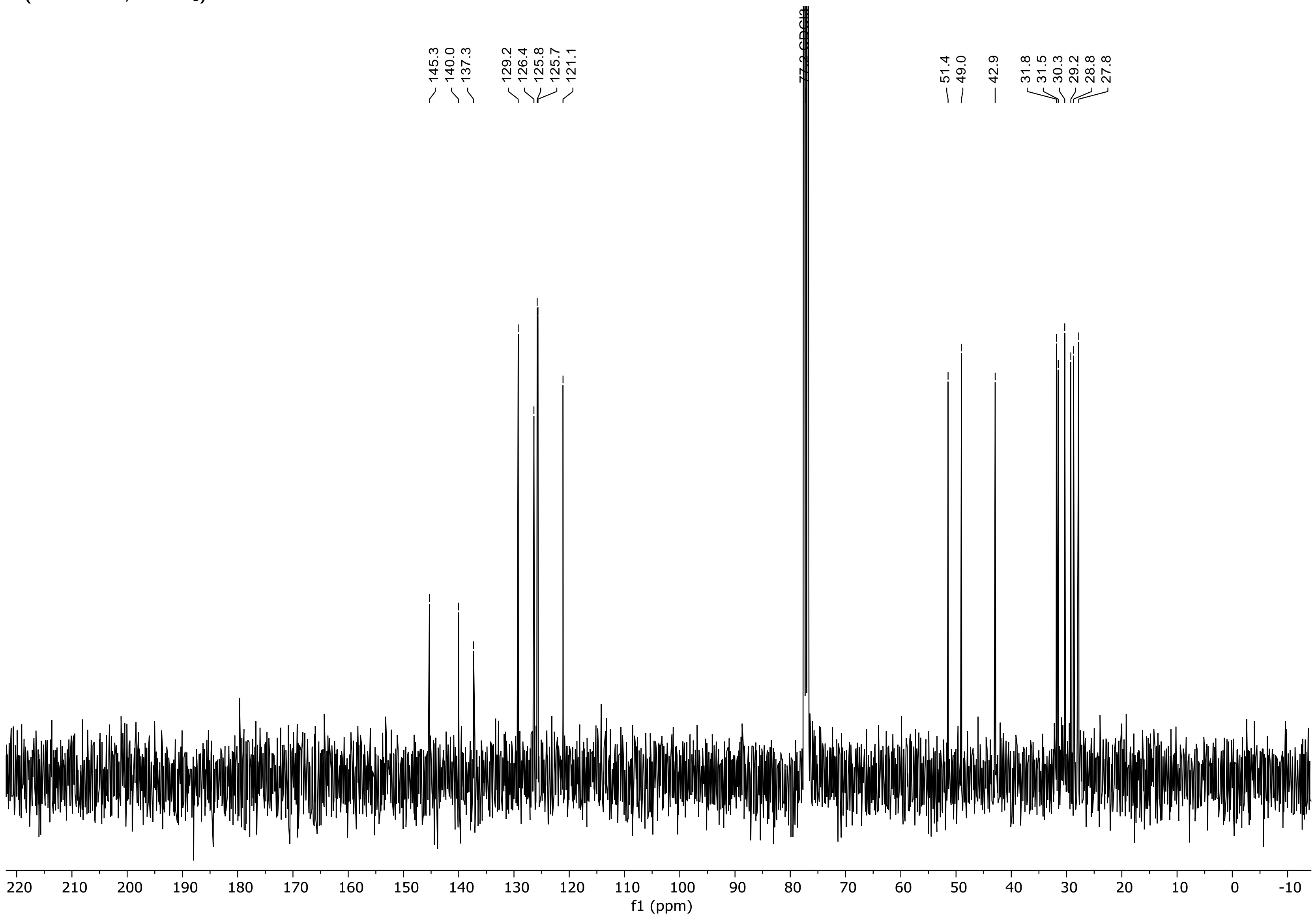

(±)-(8*S*,9*S*,13*S*,14*S*,17*S*)-7,8,9,11,12,13,14,15,16,17-Decahydro-6H-cyclopenta[a]phenanthren-17-ol 18 (major) and (±)-(8*S*,9*S*,13*R*,14*S*,17*R*)-7,8,9,11,12,13,14,15,16,17-Decahydro-6H-cyclopenta[a]phenanthren-17-ol 18 (minor)

^{1}H NMR (600 MHz, $CDCl_3$)

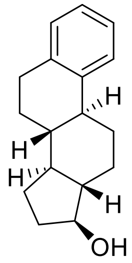

Major
18

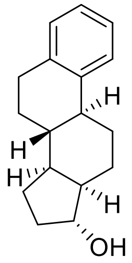

Minor
18

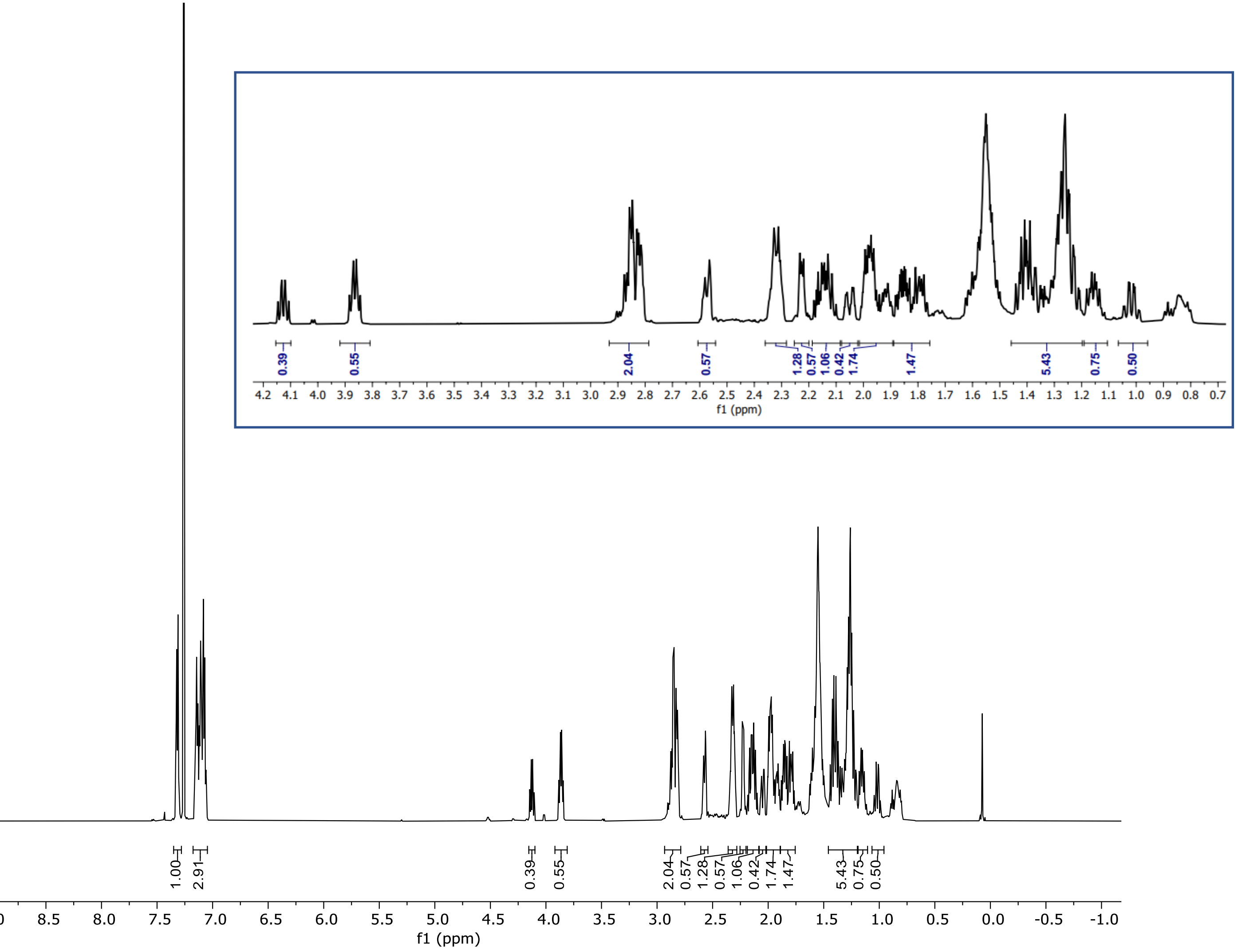

^{13}C NMR (151 MHz, $CDCl_3$)

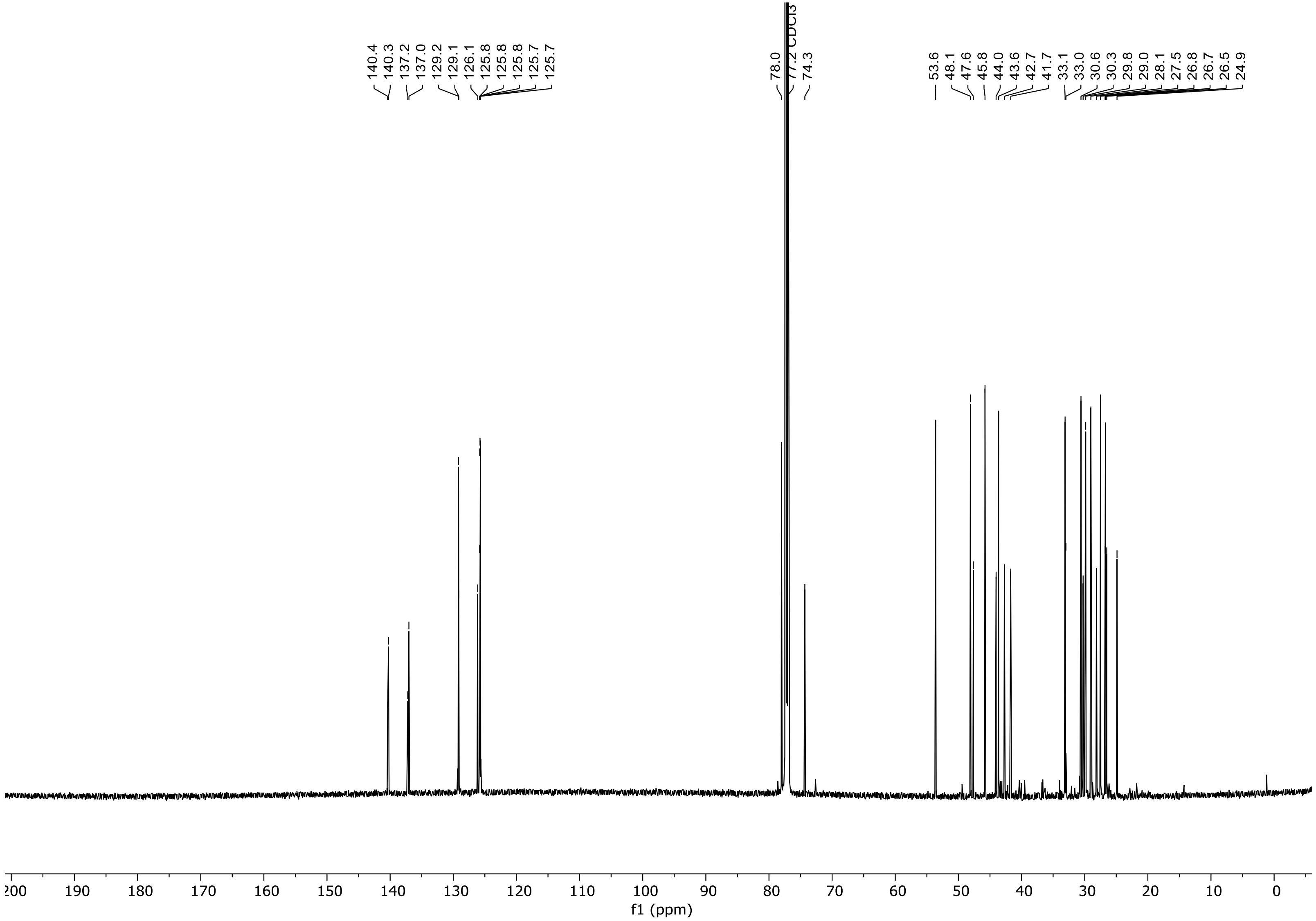

NOESY (600 MHz, $CDCl_3$)

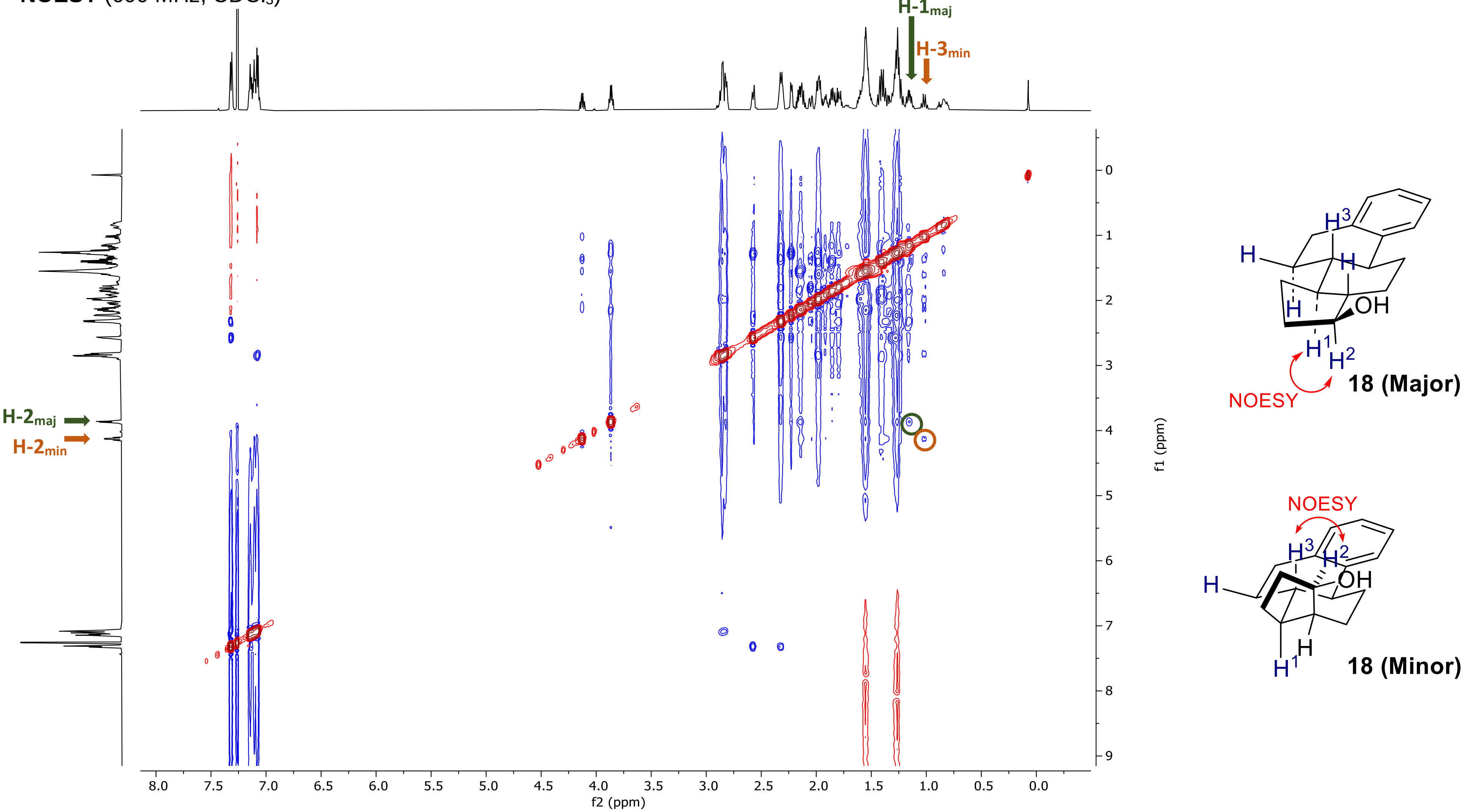

^{1}H (600 MHz)-**^{13}C** (151 MHz) **HSQC-2D** ($CDCl_3$)

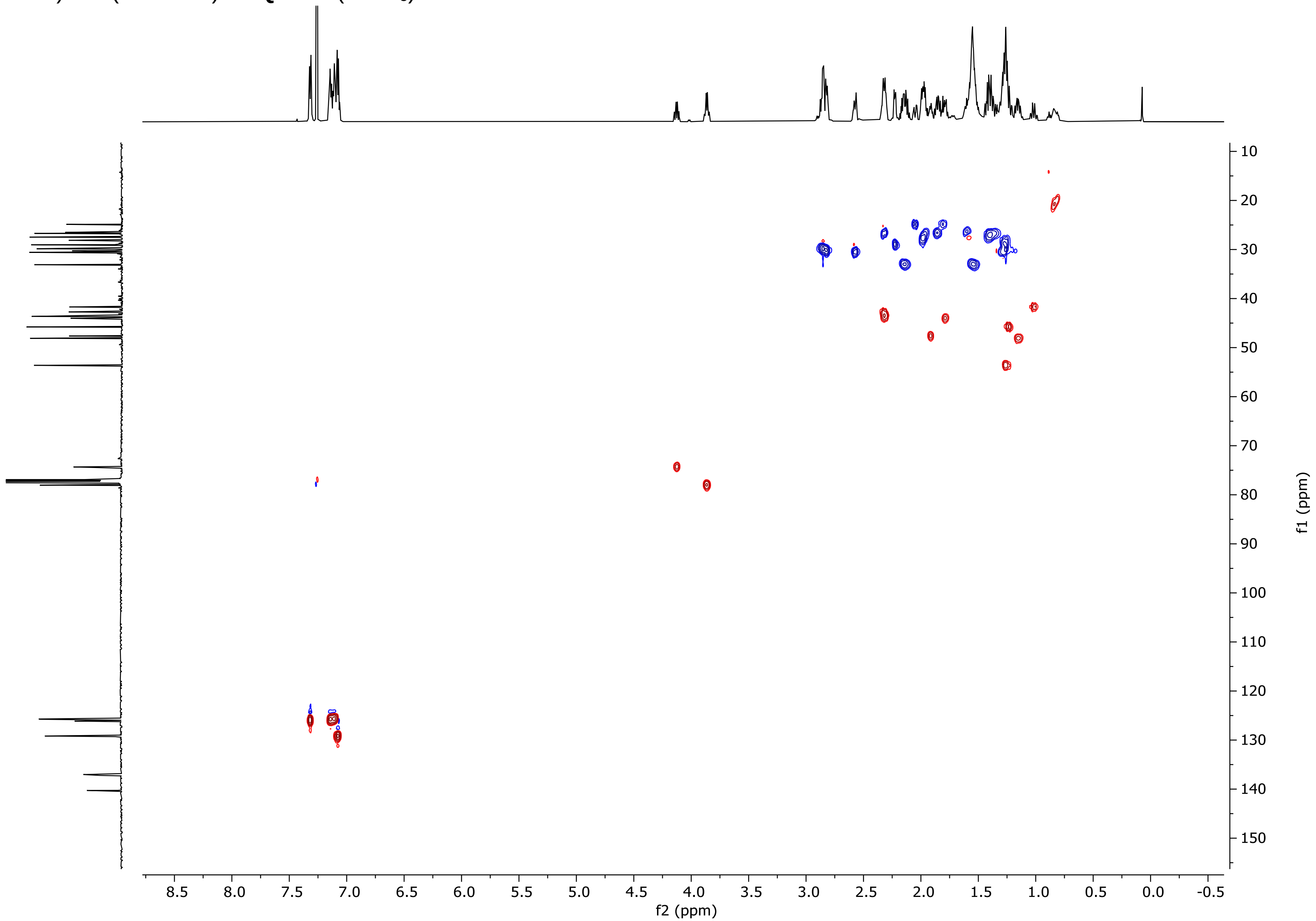

COSY-2D (600 MHz, $CDCl_3$)

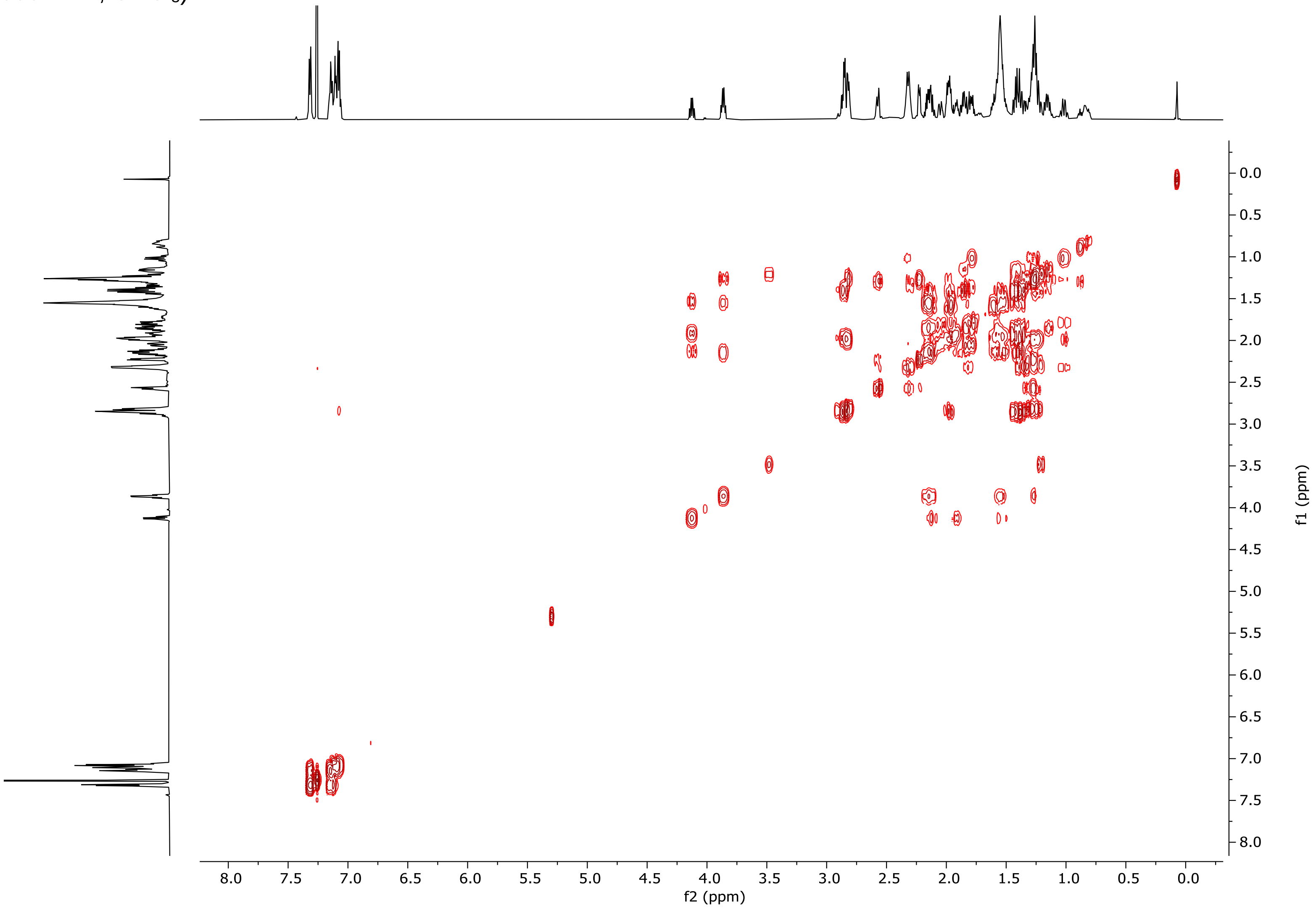

^{1}H (600 MHz)-^{13}C (151 MHz) **HMBC-2D** ($CDCl_3$)

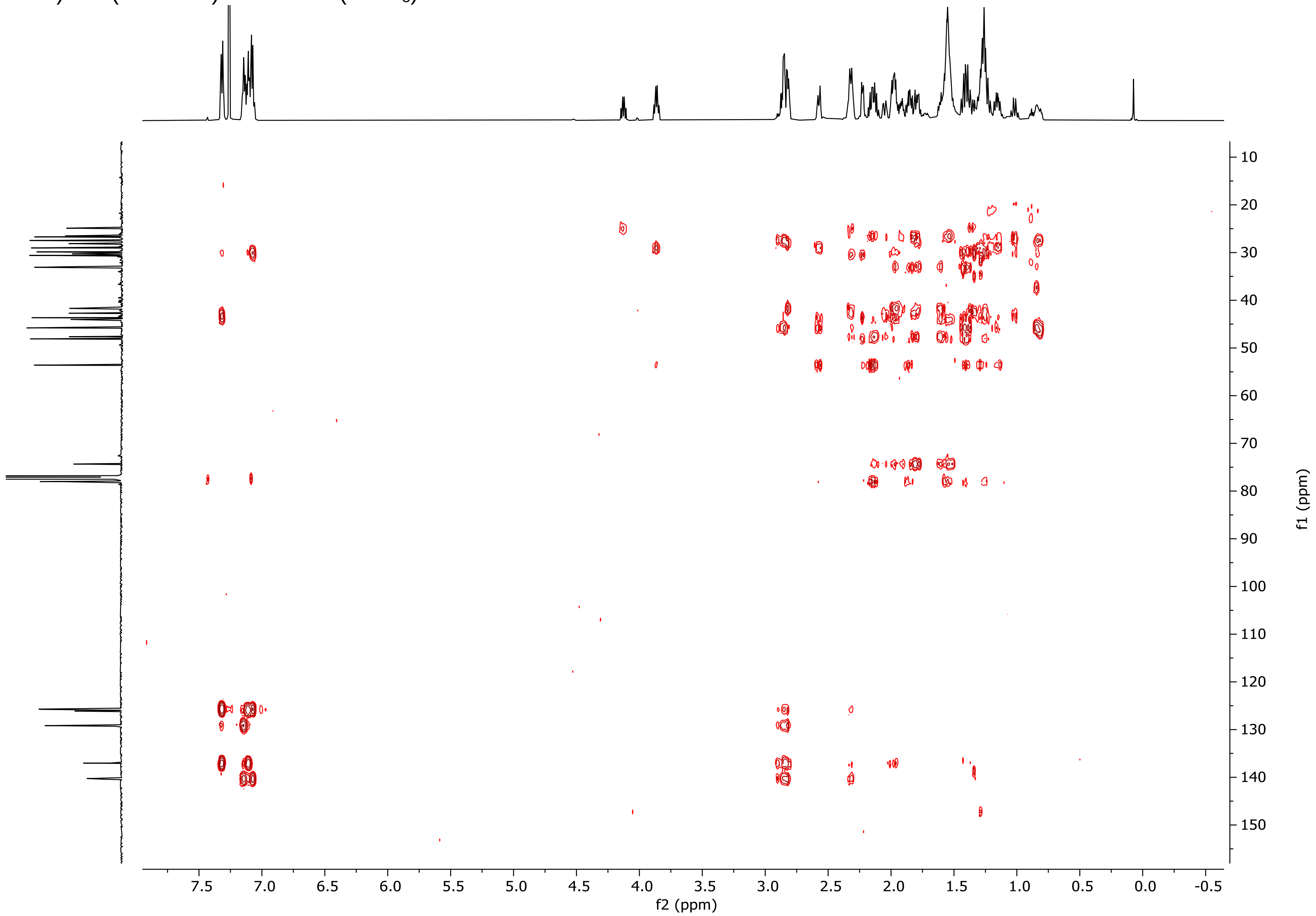

*7.6 Formal synthesis of a 18-nor-estradiol precursor **19**.*

5-(3-Methoxyphenyl)pent-1-en-3-ol S17.

^{1}H NMR (400 MHz, $CDCl_3$)

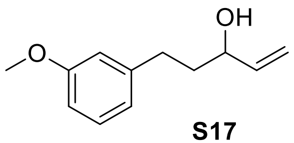

S17

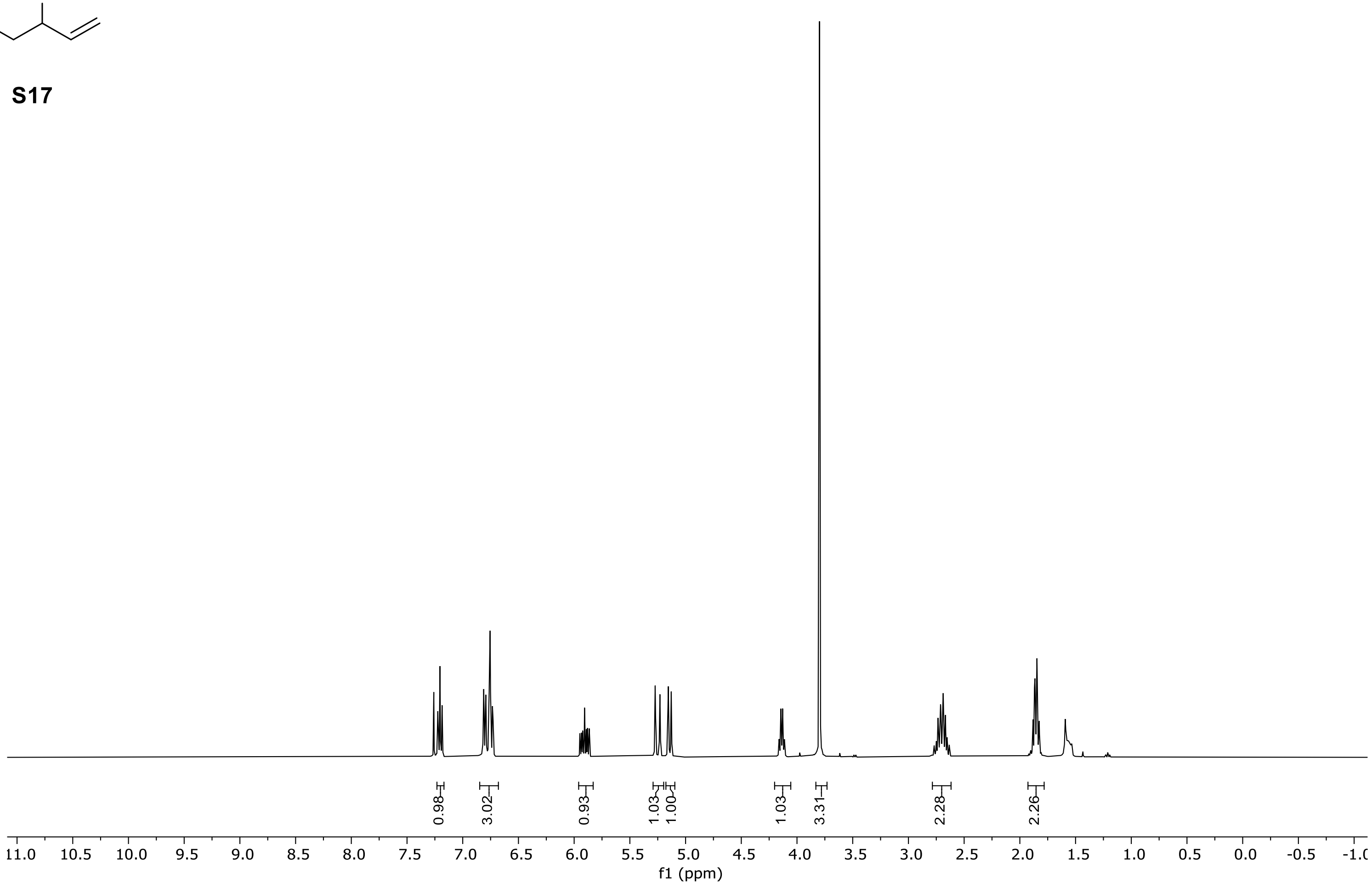

^{13}C NMR (101 MHz, $CDCl_3$)

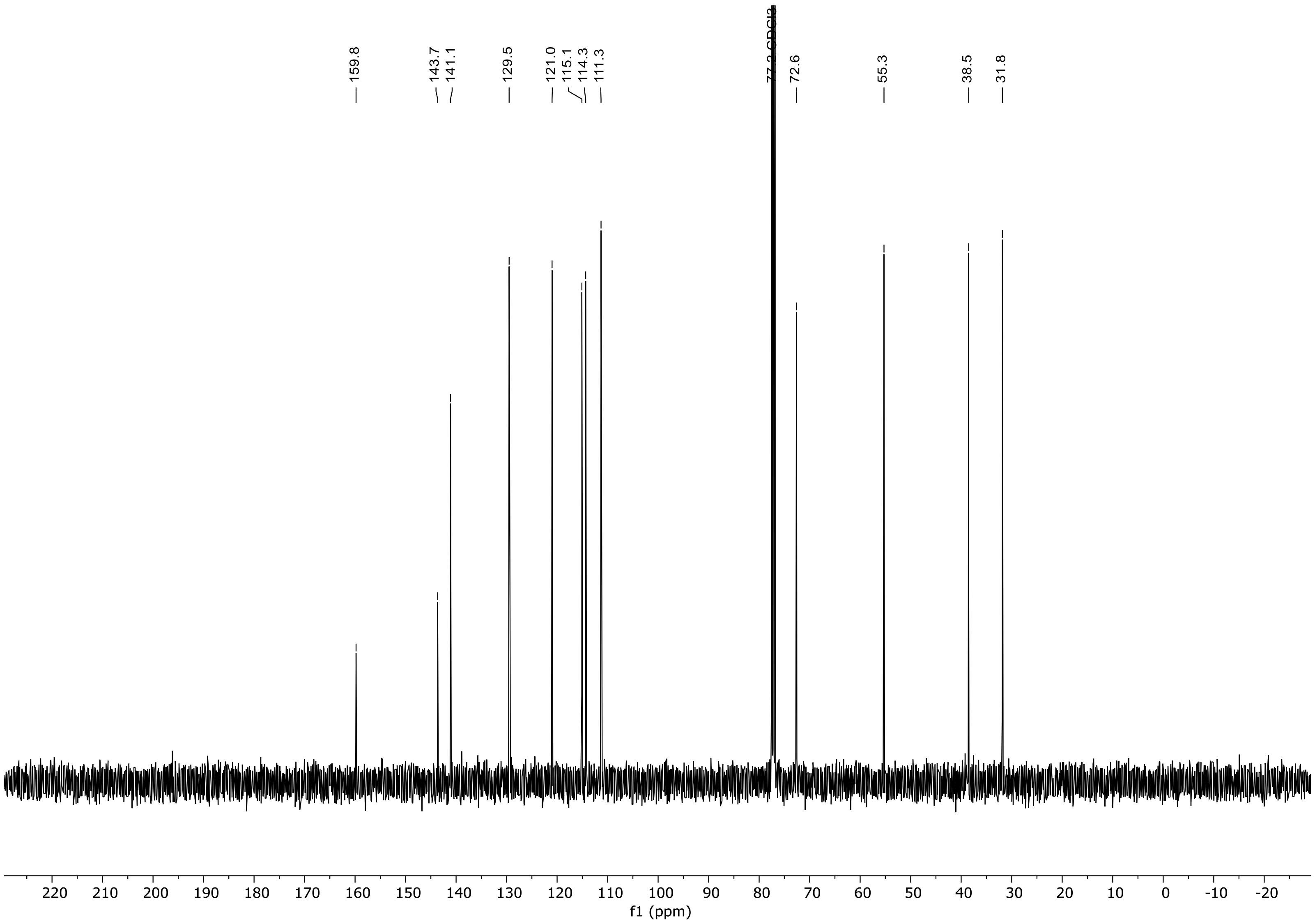

Ethyl (*E*)-7-(3-methoxyphenyl)hept-4-enoate S18.

^{1}H NMR (400 MHz, $CDCl_3$)

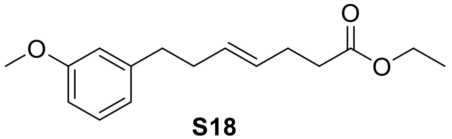

S18

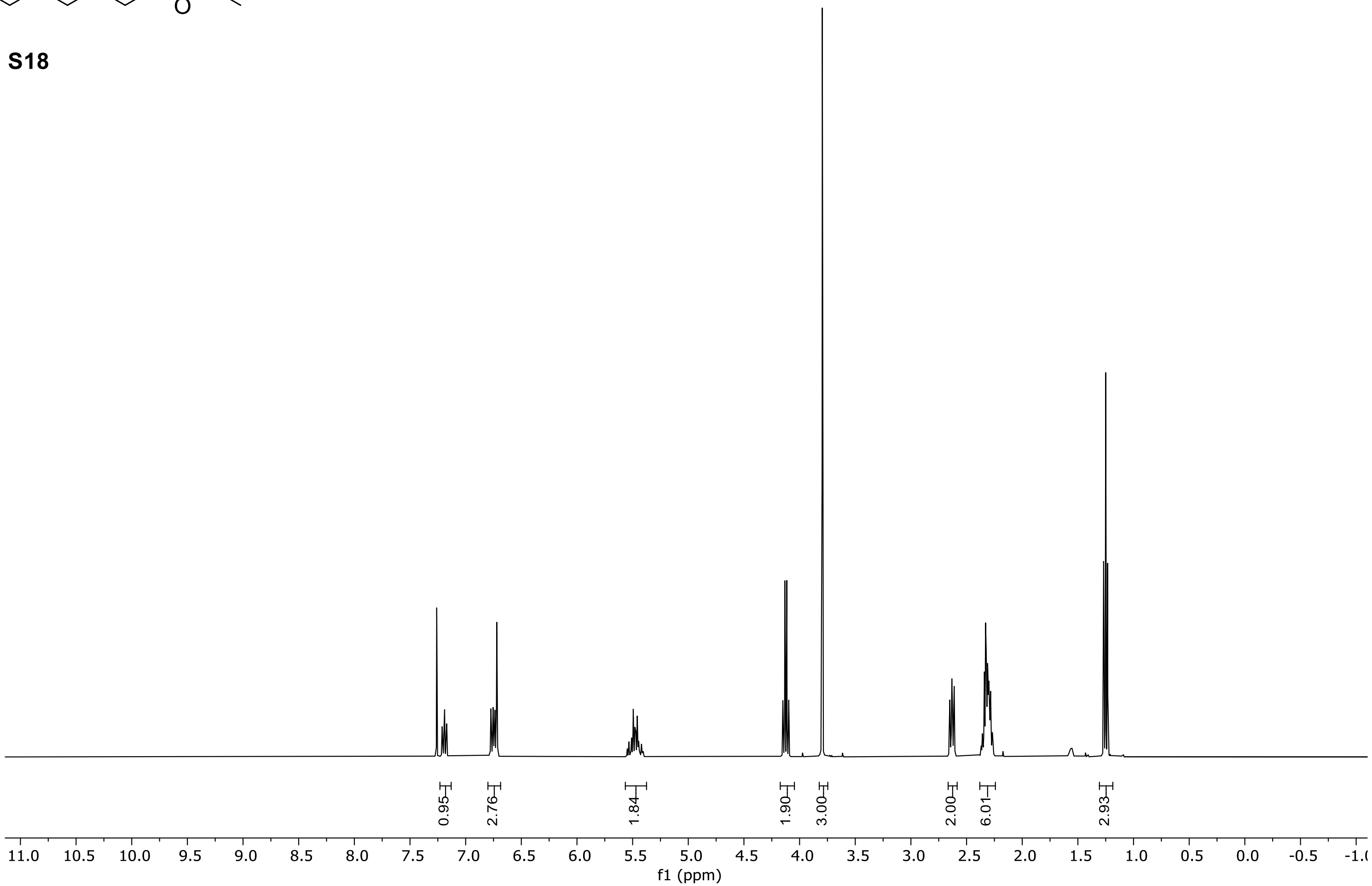

^{13}C NMR (101 MHz, $CDCl_3$)

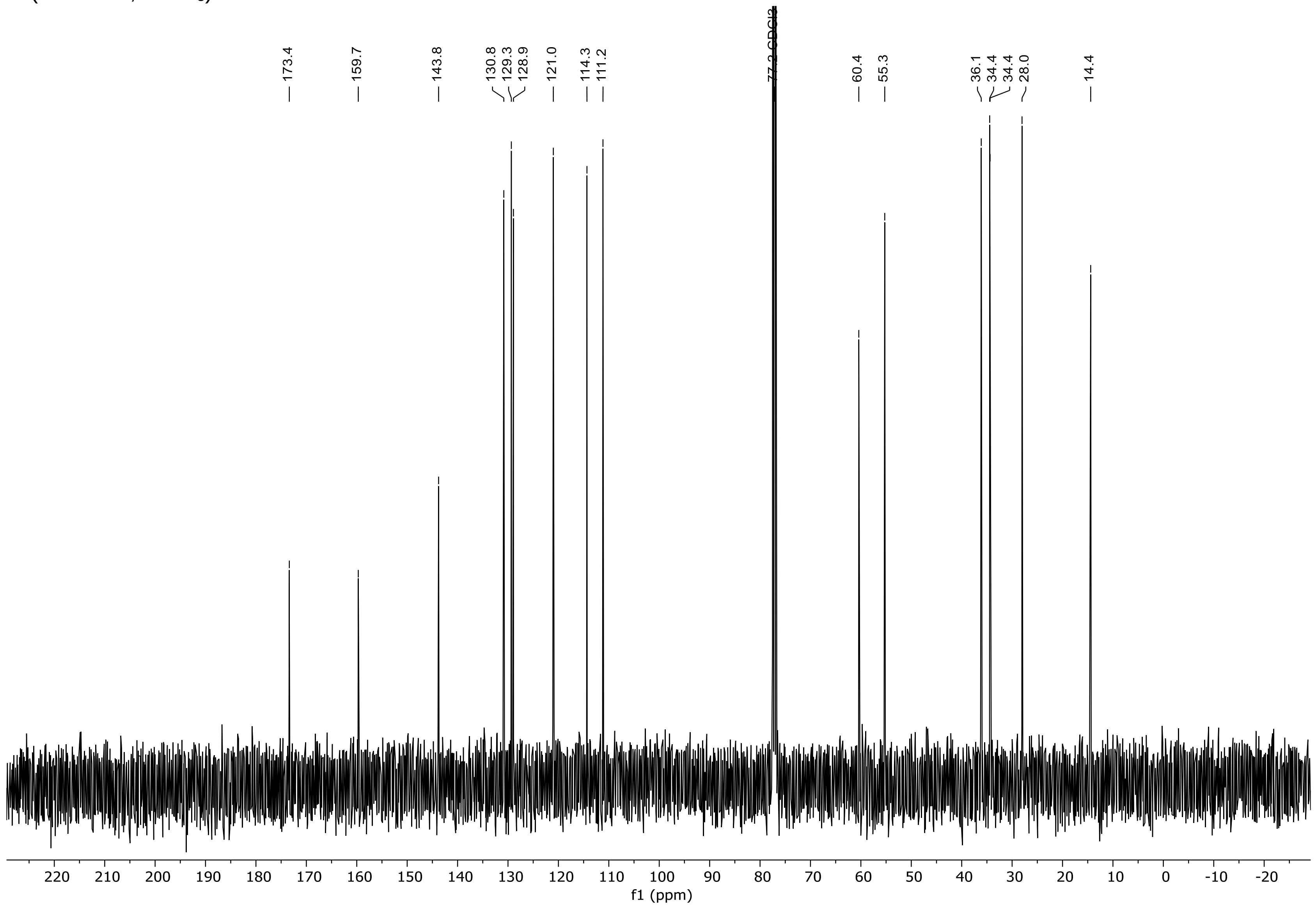

(*E*)-7-(3-Methoxyphenyl)hept-4-en-1-ol S19.

^{1}H NMR (400 MHz, $CDCl_3$)

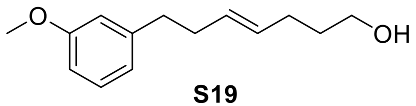

S19

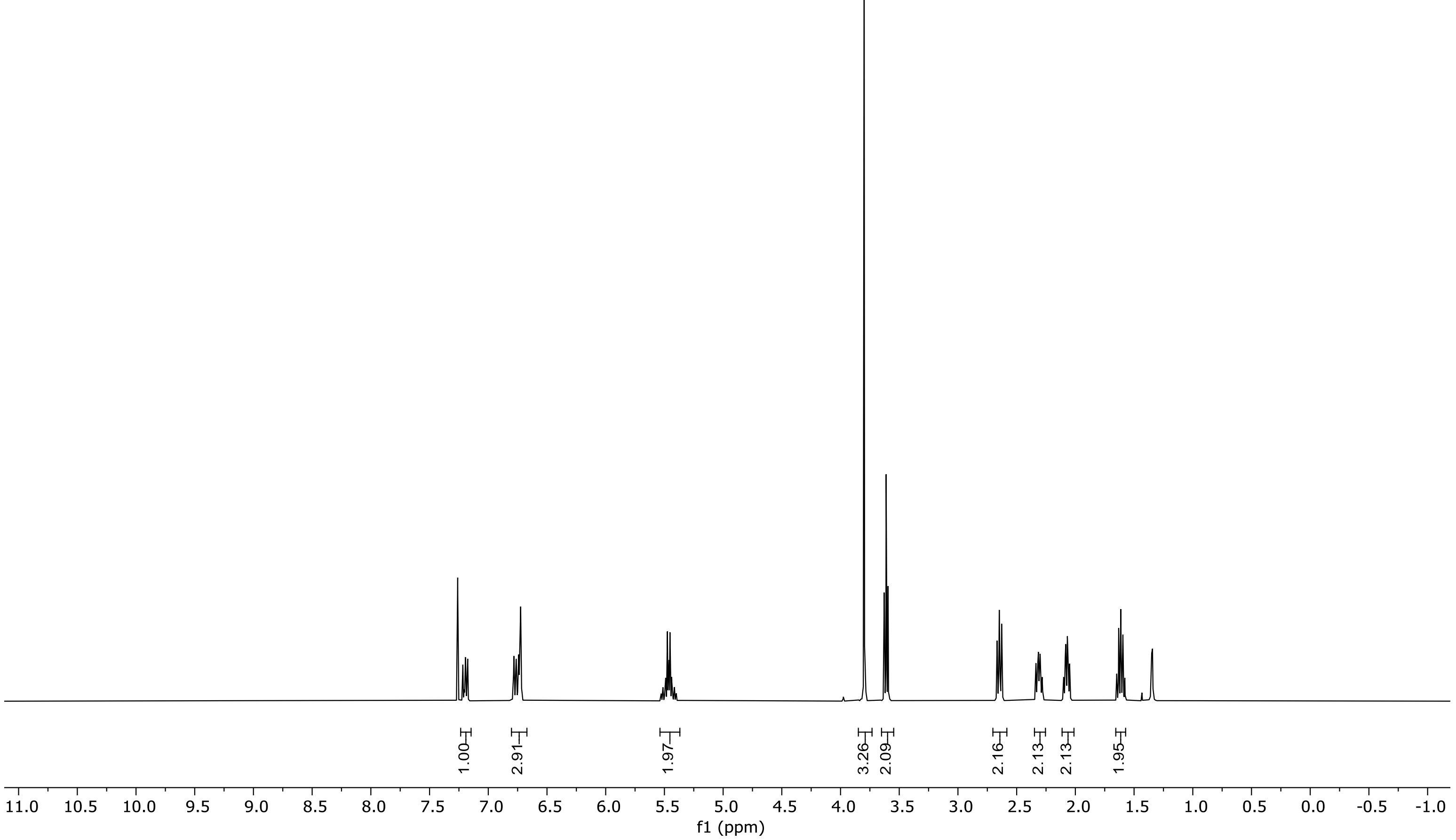

^{13}C NMR (101 MHz, $CDCl_3$)

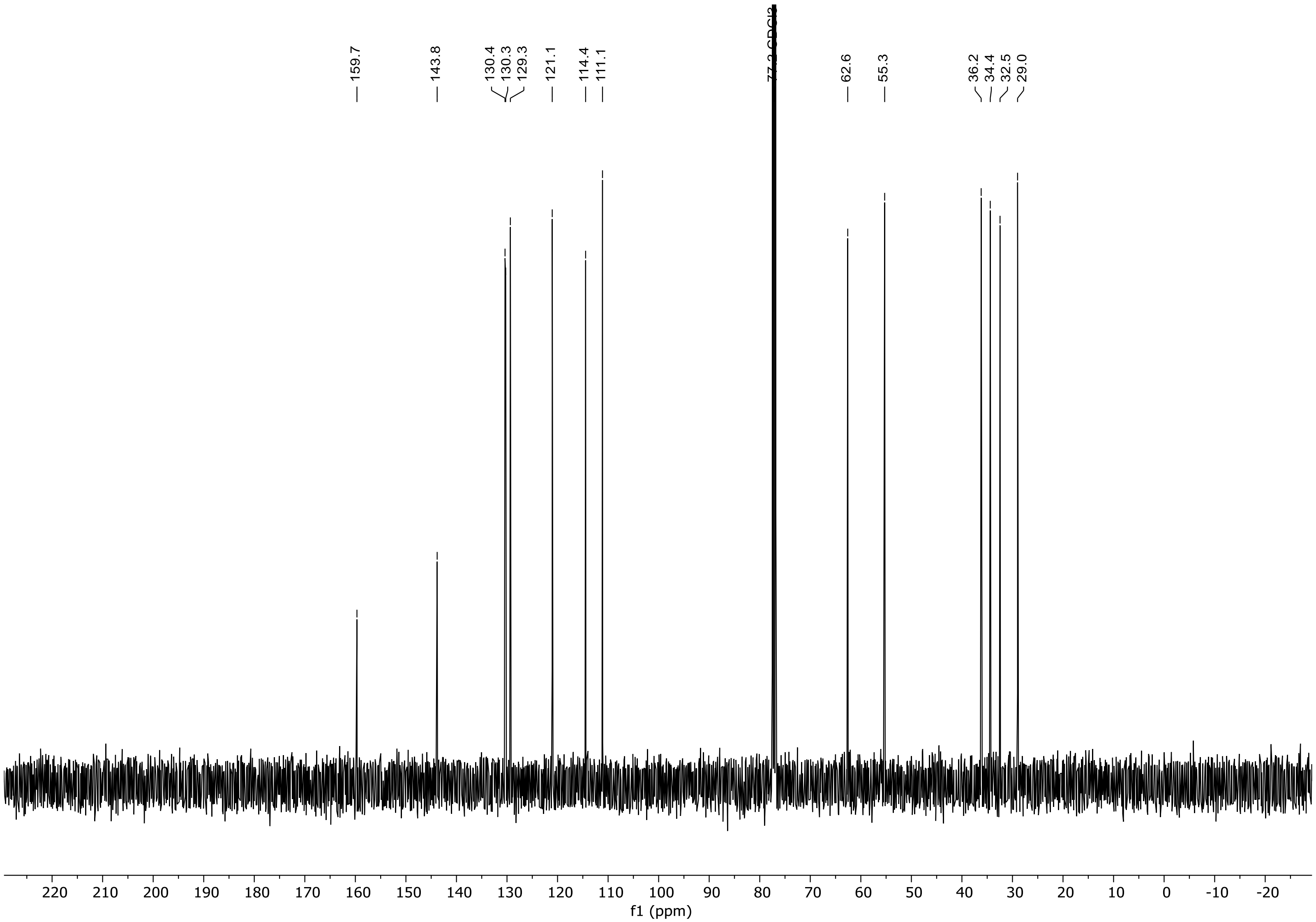

(*E*)-7-(3-methoxyphenyl)hept-4-enal S20.

^{1}H NMR (400 MHz, $CDCl_3$)

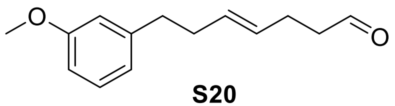

S20

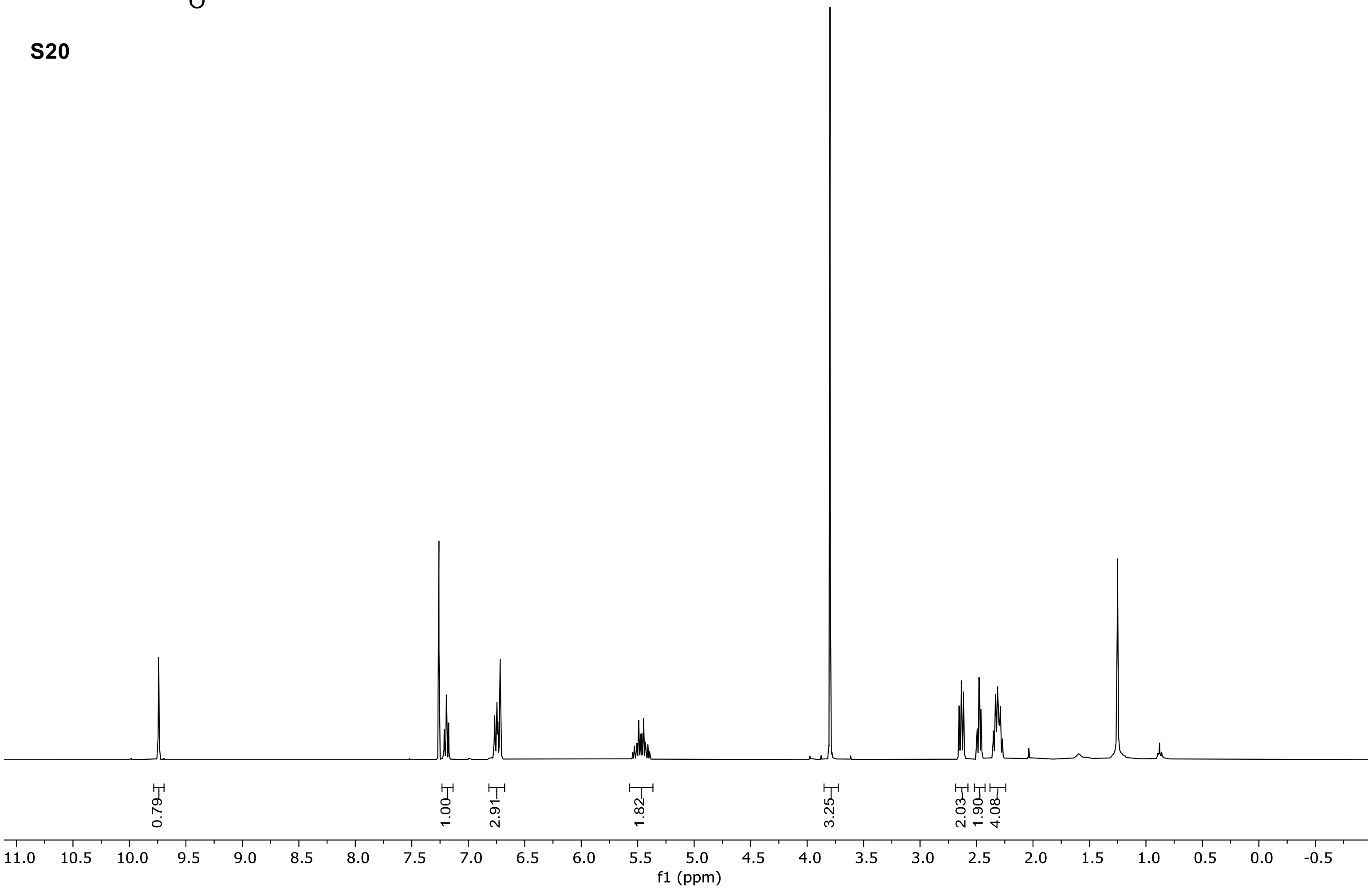

^{13}C NMR (101 MHz, $CDCl_3$)

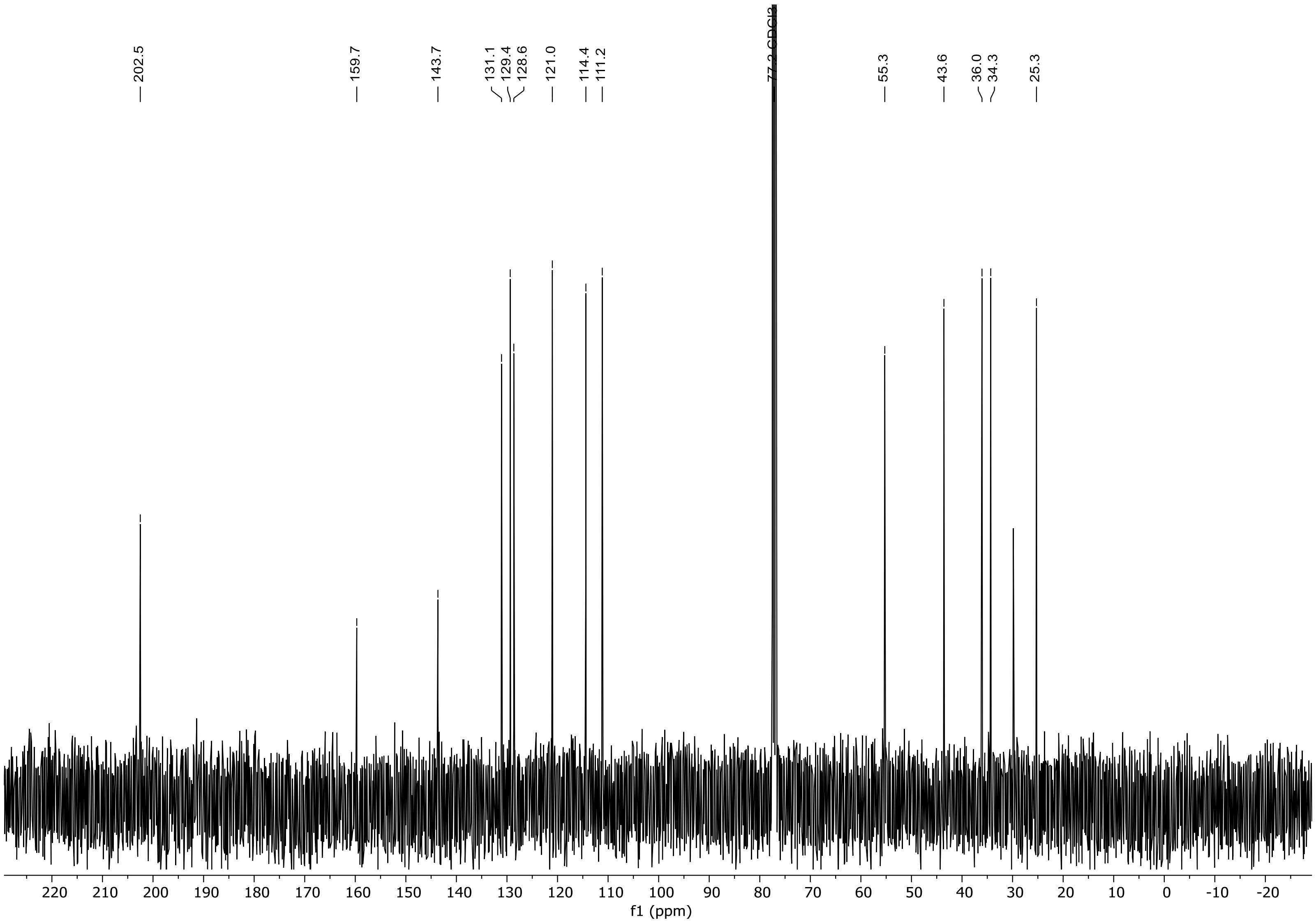

1-Methoxy-3-((3*E*,7*E*,9*E*)-10-(4-methoxyphenyl)deca-3,7,9-trien-1-yl)benzene (*E*)-S21 and 1-methoxy-3-((3*E*,7*Z*,9*E*)-10-(4-methoxyphenyl)deca-3,7,9-trien-1-yl)benzene (*Z*)-S21.

^{1}H NMR (400 MHz, $CDCl_3$)

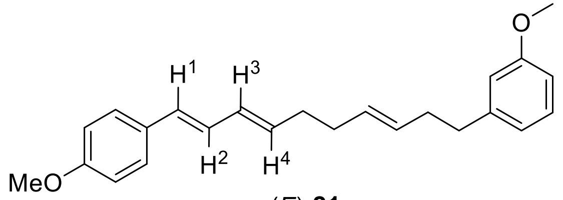

(*E*)-**21**
Major

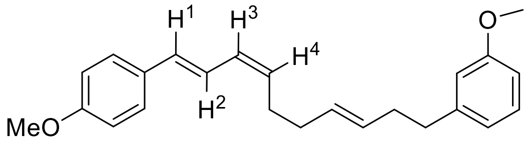

(*Z*)-**21**
Minor

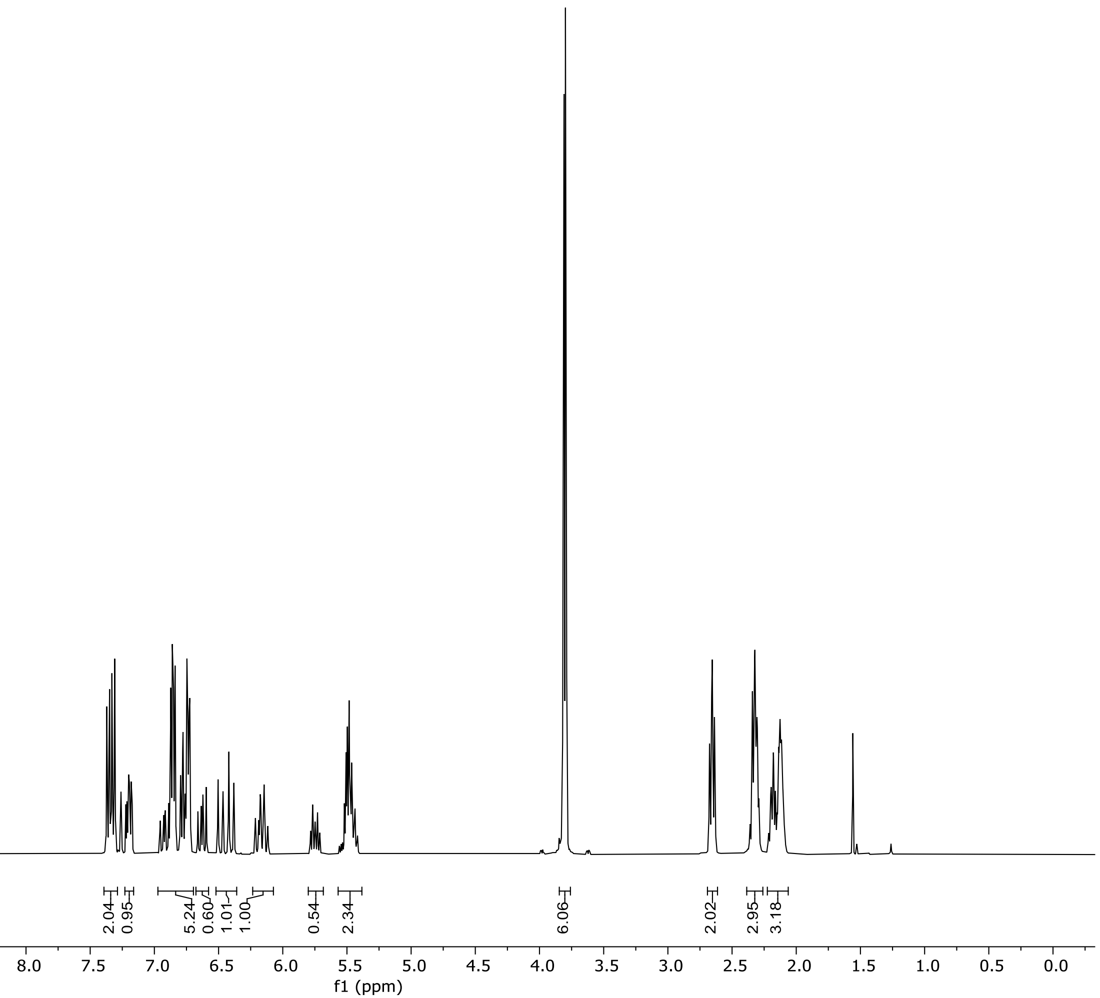

^{13}C NMR (101 MHz, $CDCl_3$)

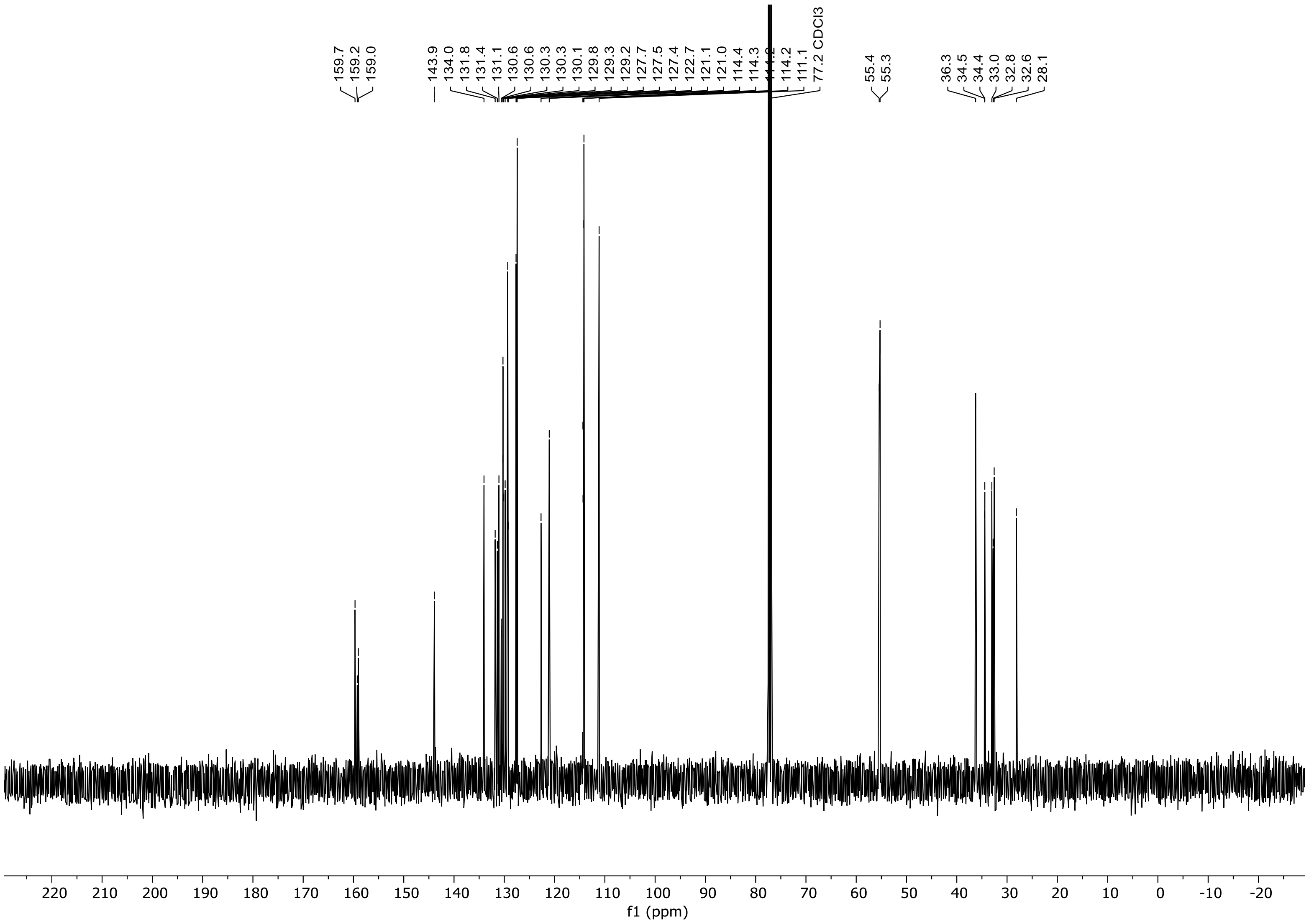

(±)-(1*S*,2*R*,4a*S*,10a*S*)-7-Methoxy-2-((*E*)-3-(4-methoxyphenyl)allyl)-1-((*E*)-4-methoxystyryl)-1,2,3,4,4a,9,10,10a-octahydrophenanthrene S22 (major).

^{1}H NMR (500 MHz, $CDCl_3$)

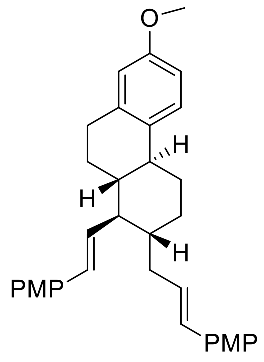

S22 (Major)

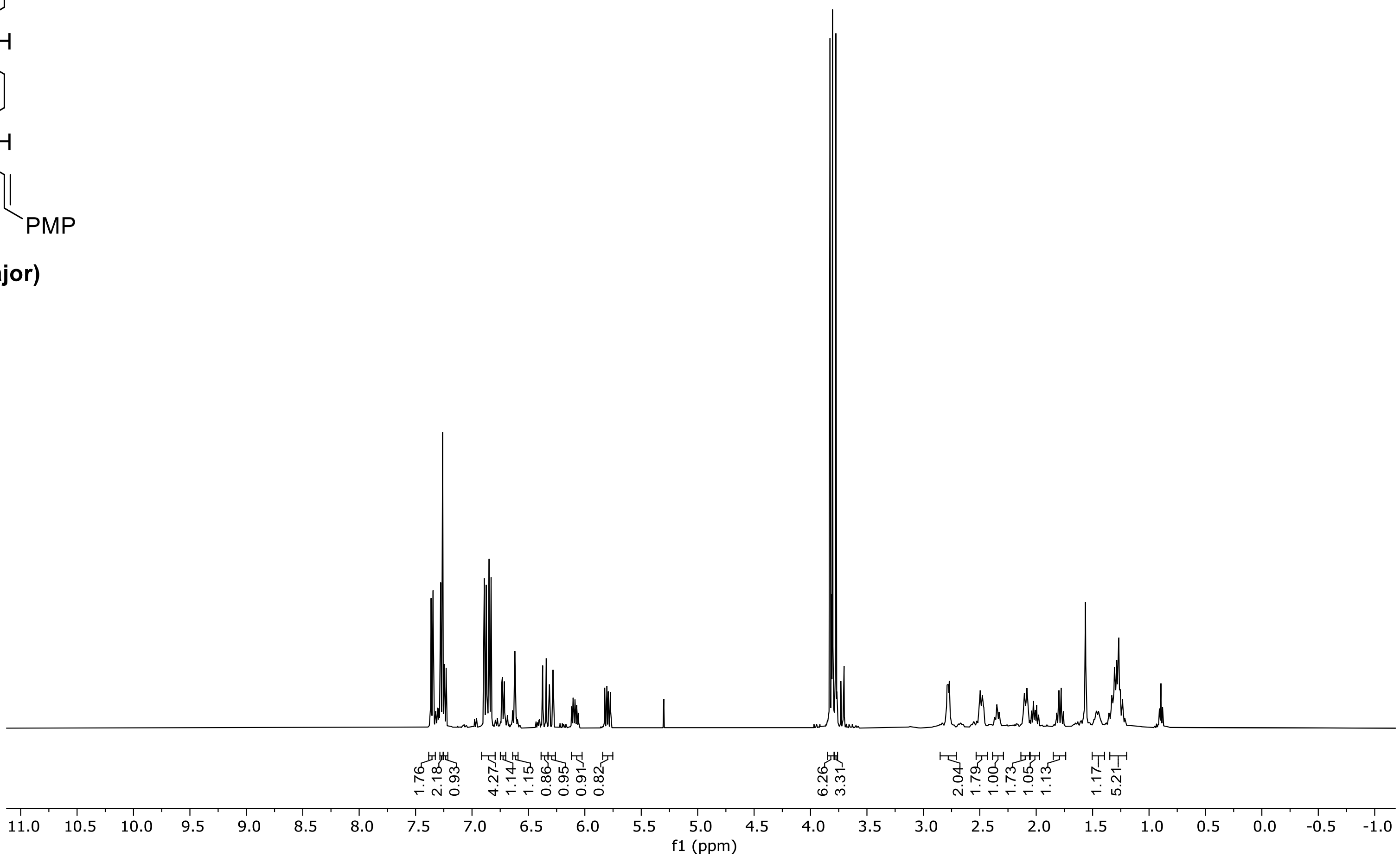

^{13}C NMR (125 MHz, $CDCl_3$):

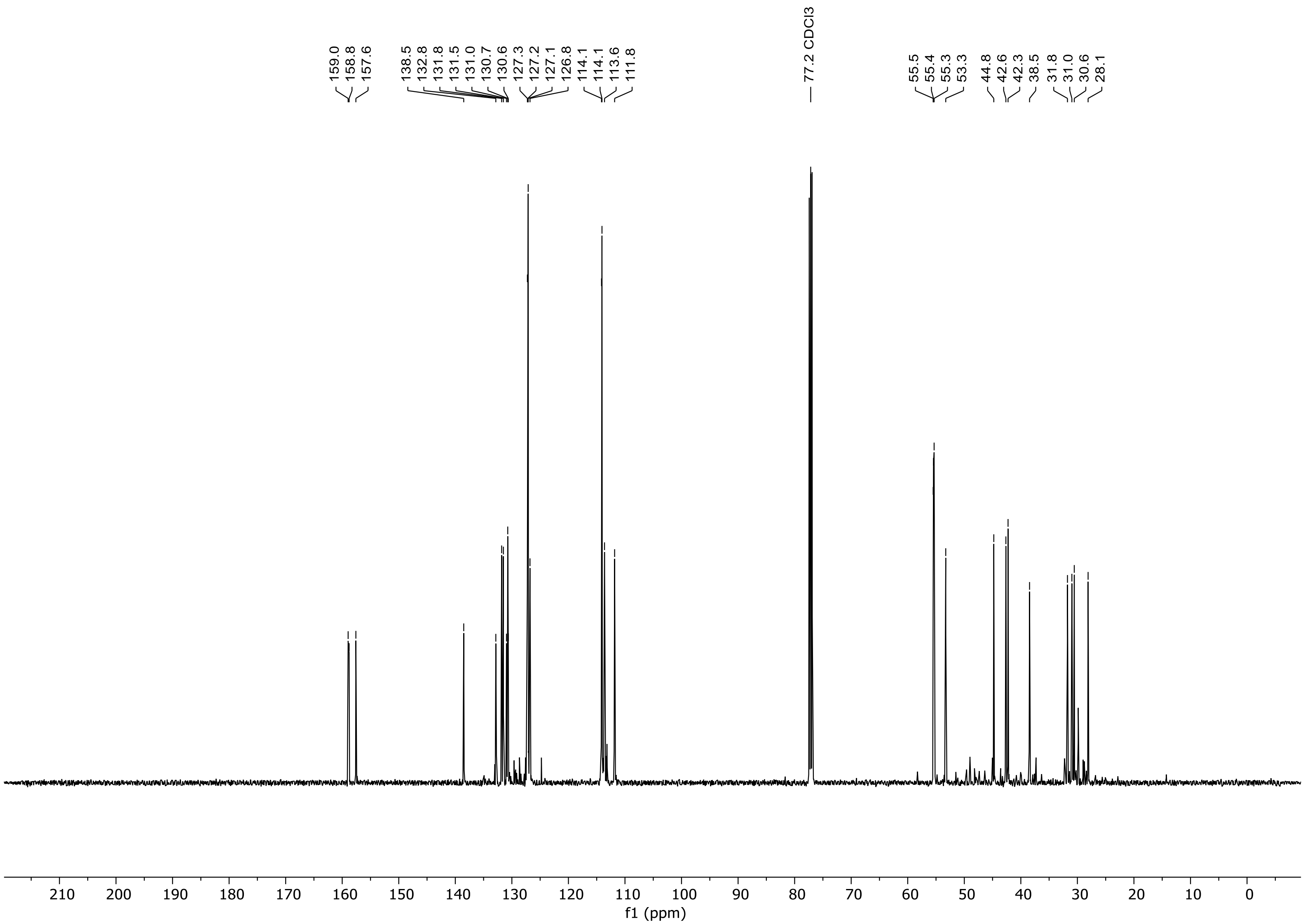

NOESY (500 MHz, $CDCl_3$)

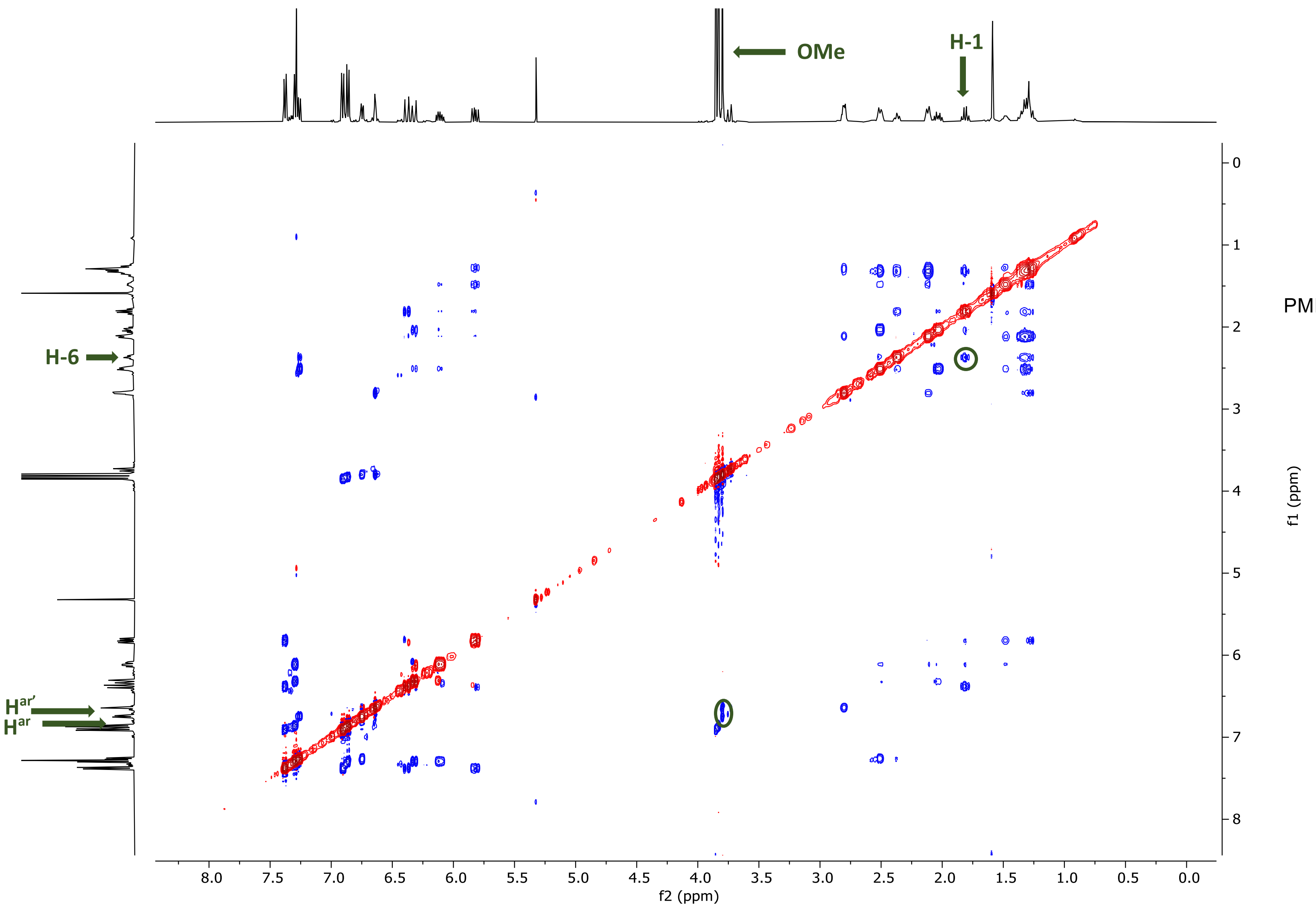

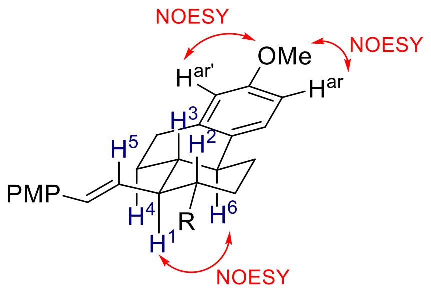

S22 (Major)

(1*S*,2*R*,4*aS*,10*aS*)-5-Methoxy-2-((*E*)-3-(4-methoxyphenyl)allyl)-1-((*E*)-4-methoxystyryl)-1,2,3,4,4*a*,9,10,10*a*-octahydrophenanthrene *S*22' (minor).

^{1}H NMR (400 MHz, $CDCl_3$)

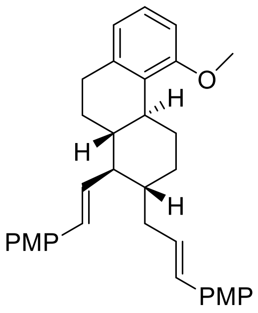

S22' (Minor)

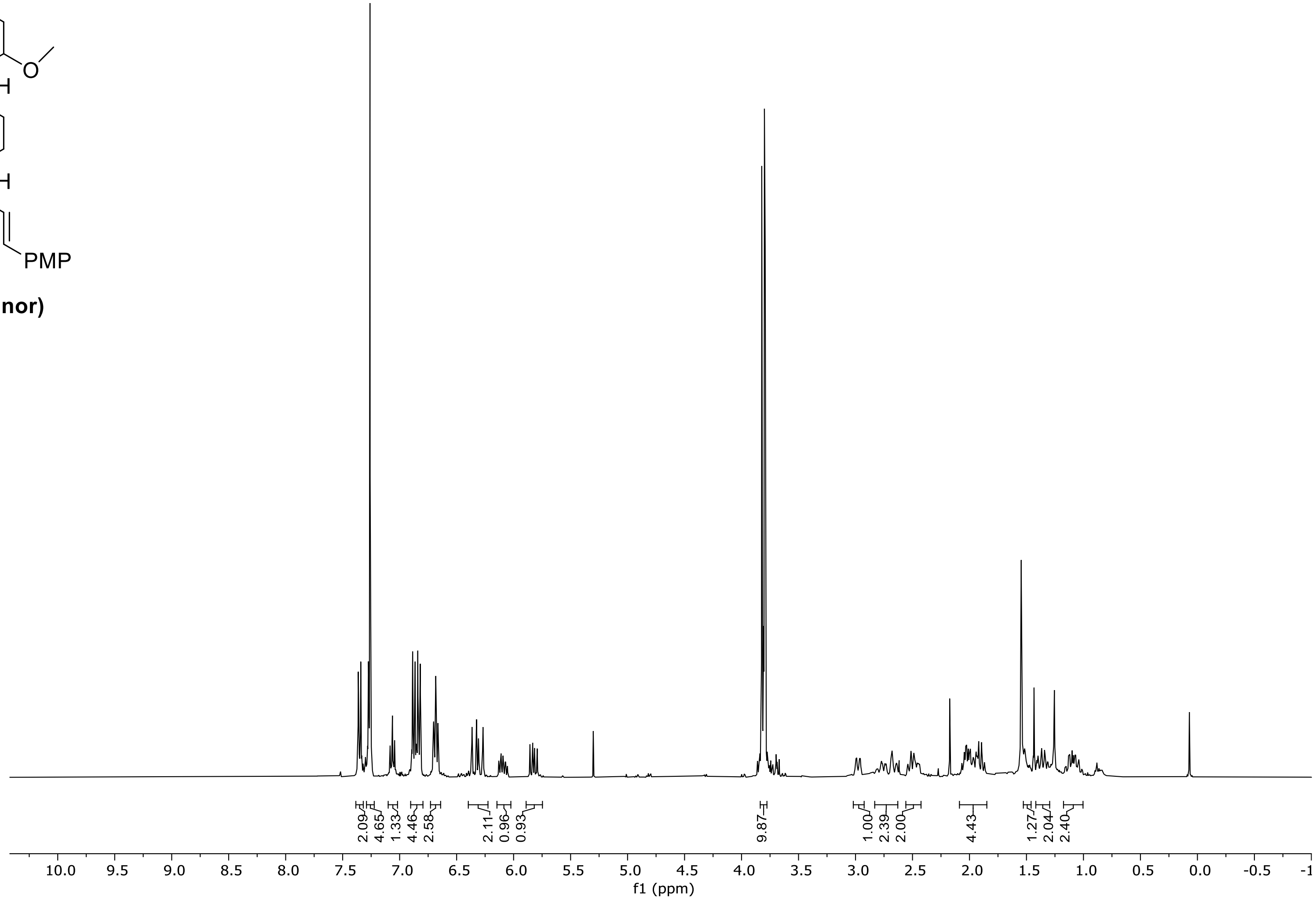

^{13}C NMR (125 MHz, $CDCl_3$):

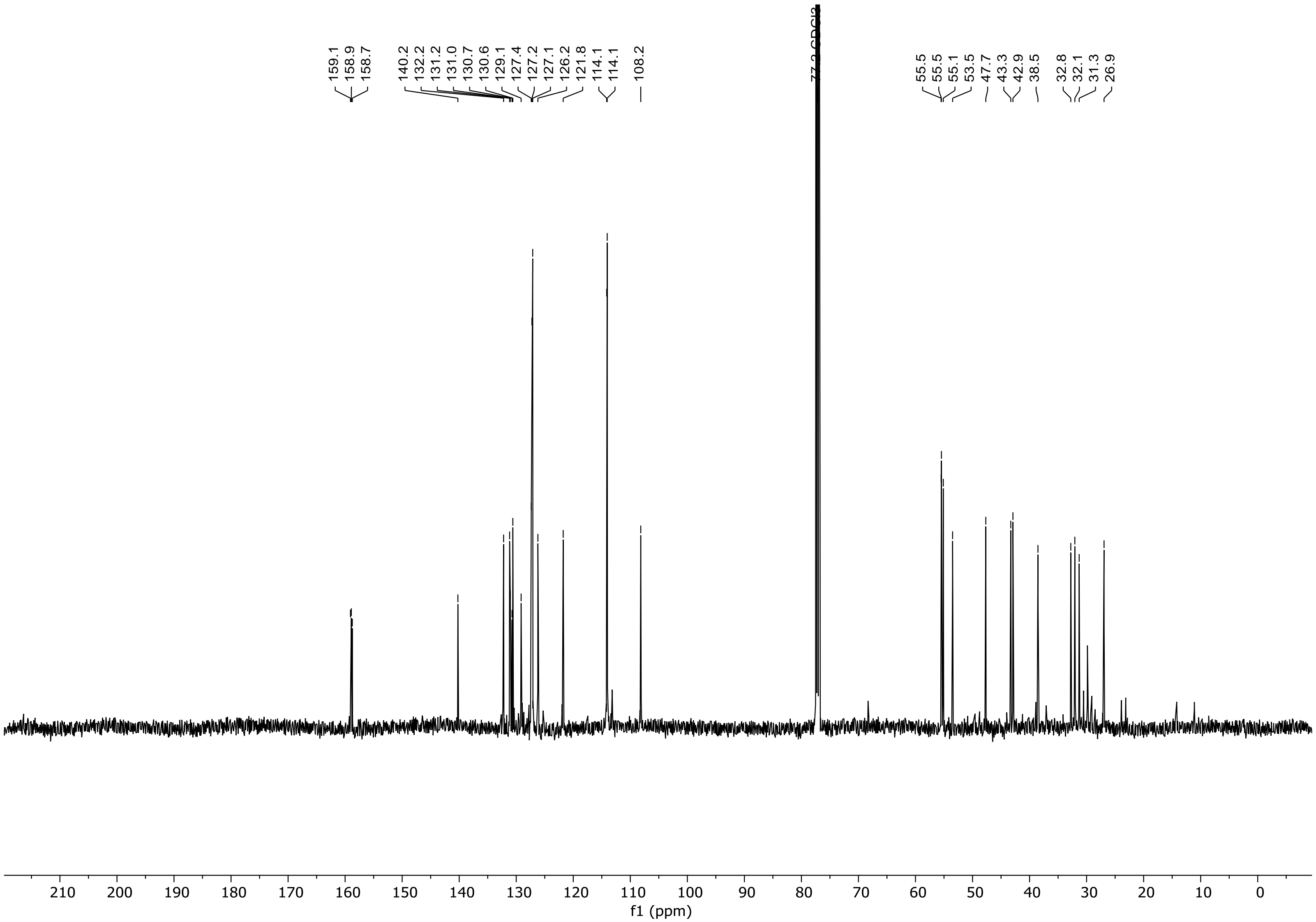

NOESY (500 MHz, $CDCl_3$)

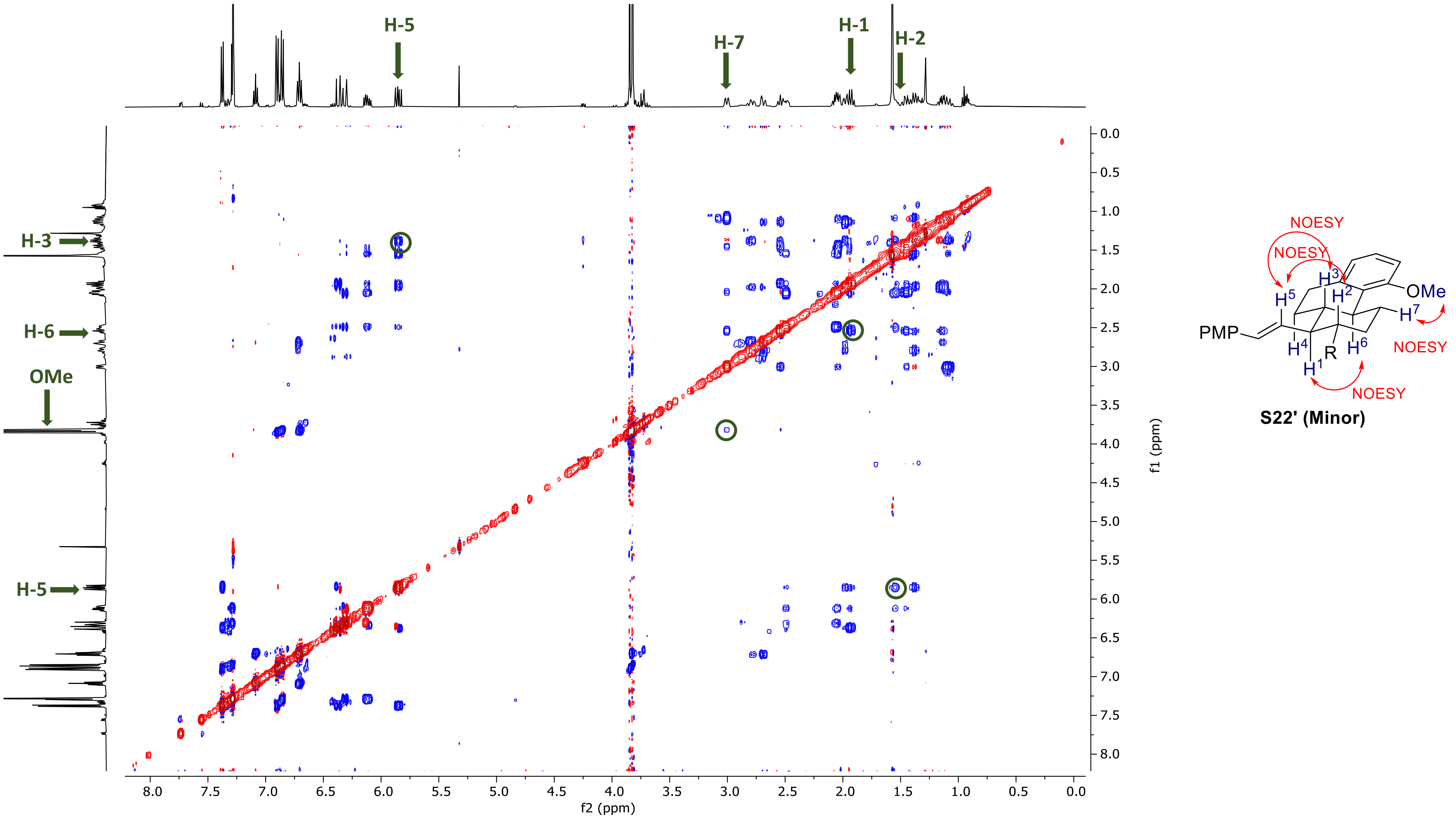

S22' (Minor)

^{1}H (500 MHz)-**^{13}C** (125 MHz) **HSQC-2D** ($CDCl_3$)

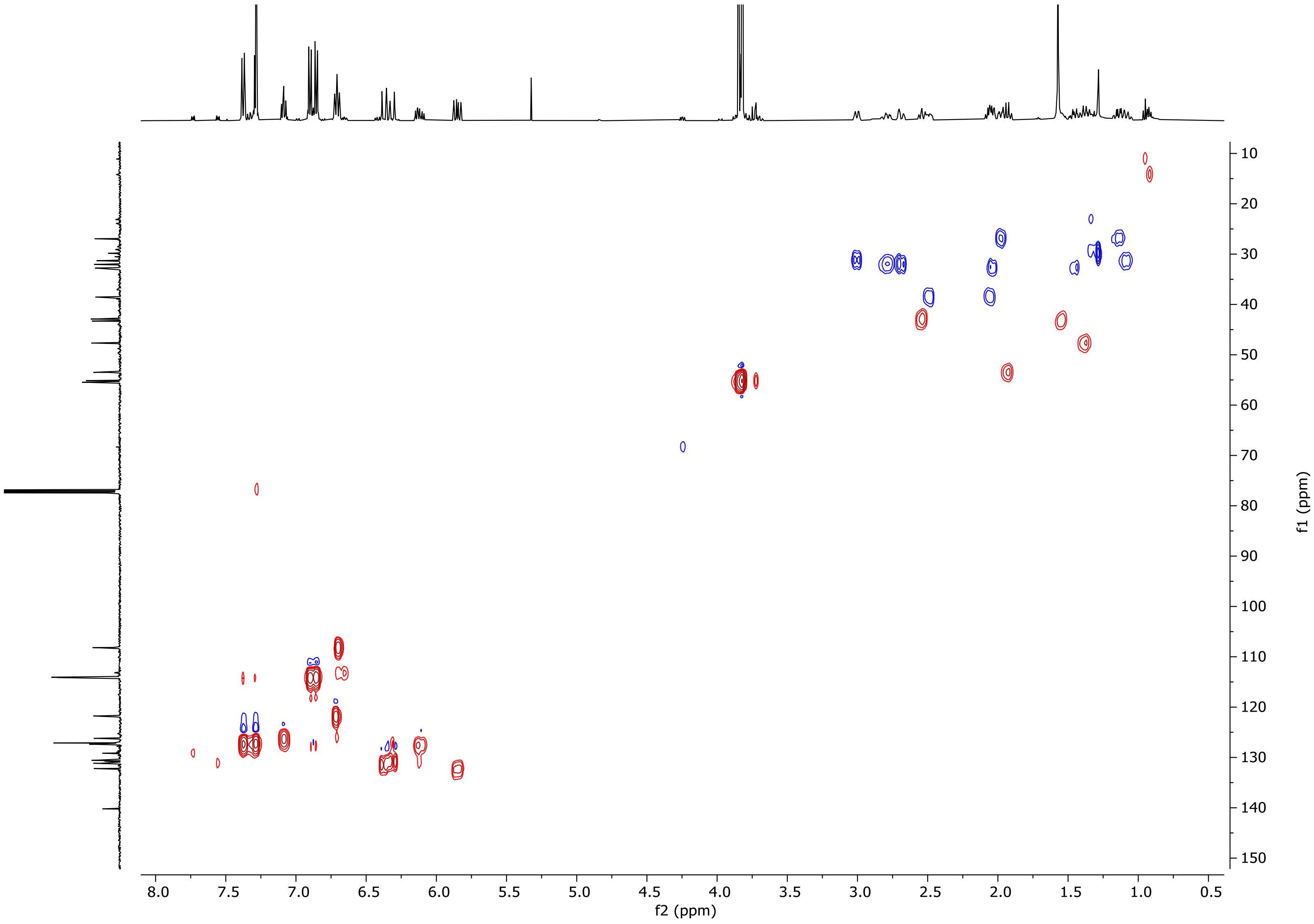

(±)-(8*S*,9*S*,14*S*)-3-Methoxy-7,8,9,11,12,14,15,16-octahydro-6H-cyclopenta[*a*]phenanthrene 19.

^{1}H NMR (600 MHz, $CDCl_3$)

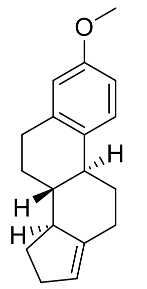

19

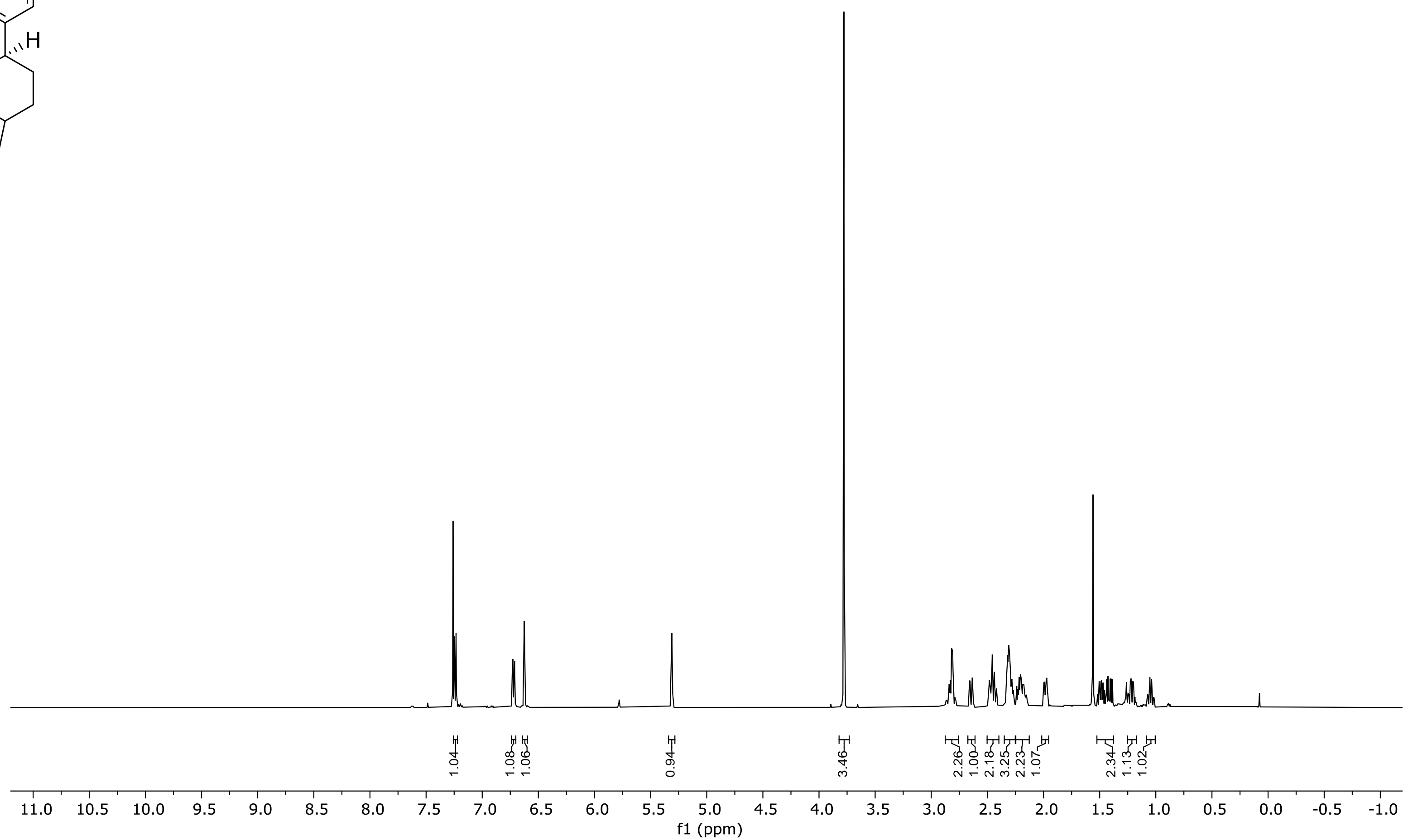

^{13}C NMR (101 MHz, $CDCl_3$)

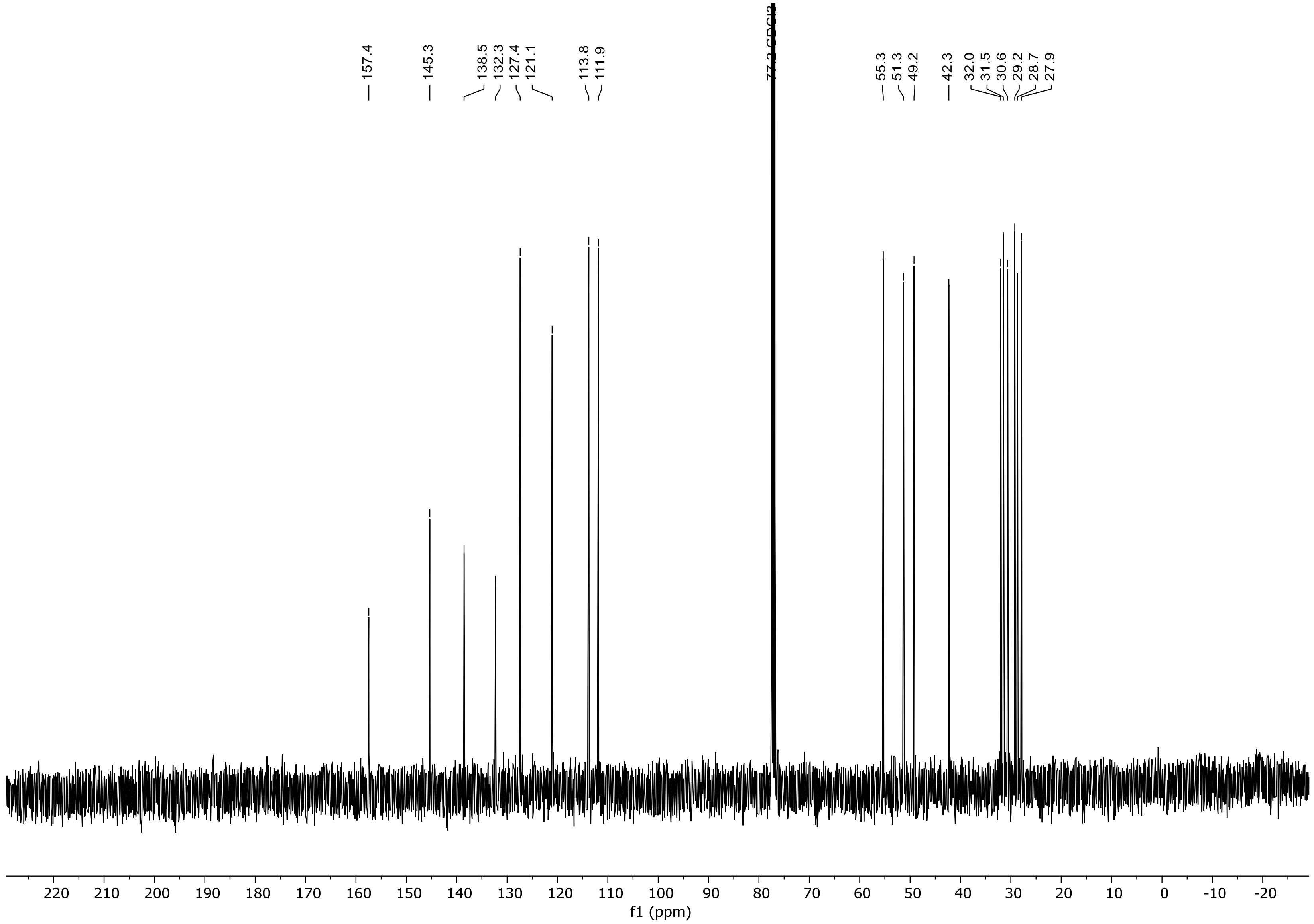

NOESY (600 MHz, $CDCl_3$)

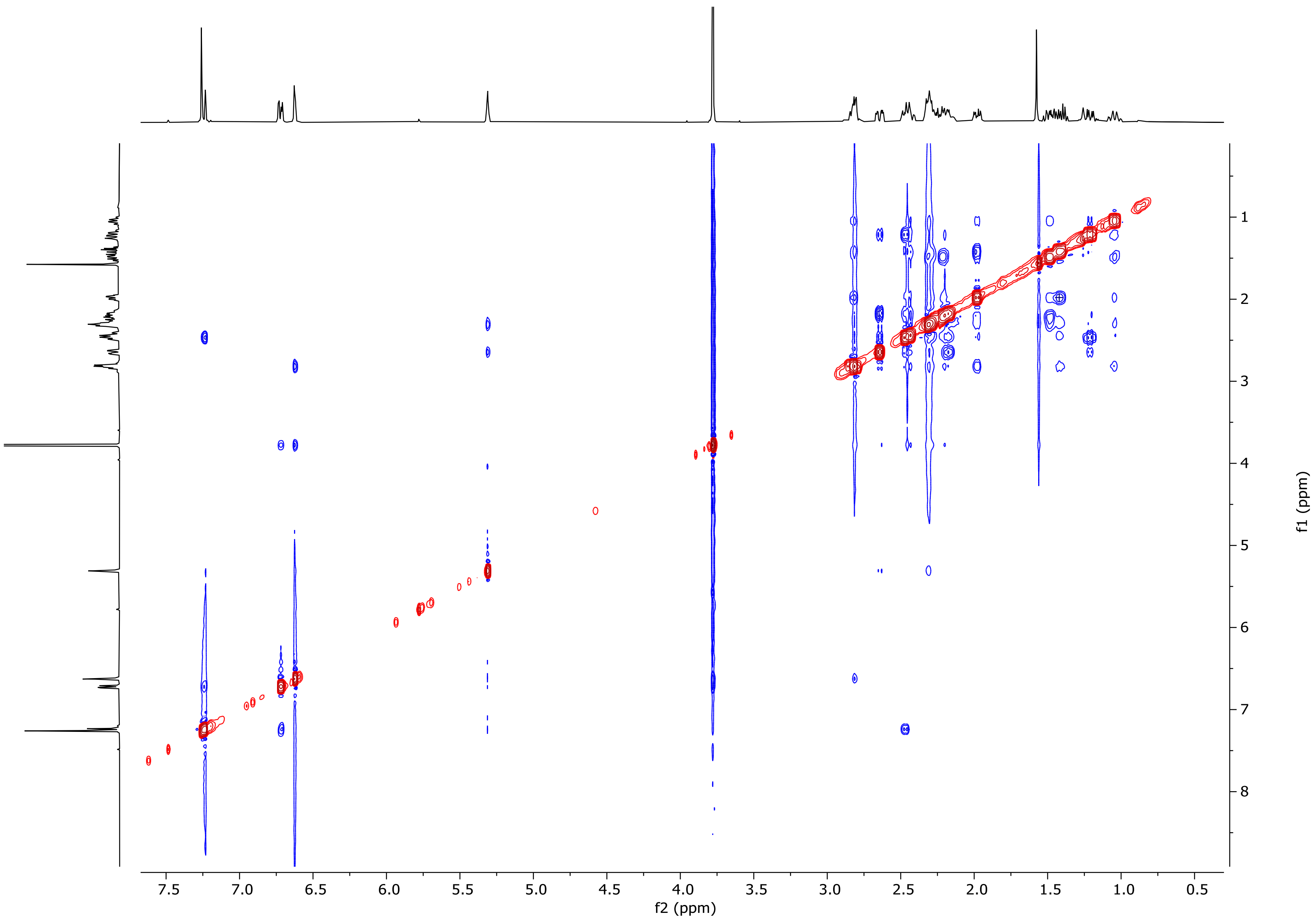

^{1}H (400 MHz)-**^{13}C** (101 MHz) **HSQC-2D** ($CDCl_3$)

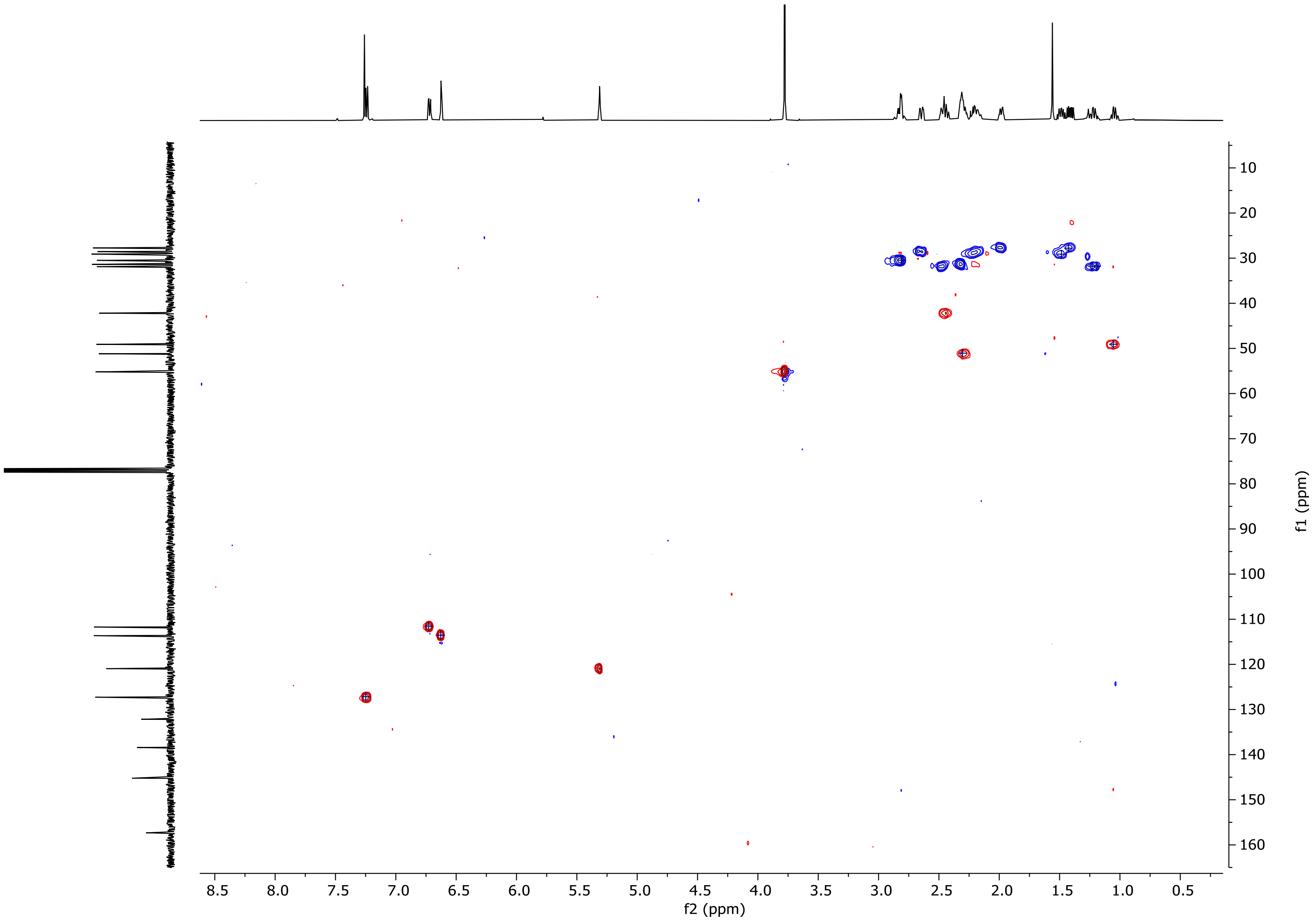

COSY-2D (400 MHz, $CDCl_3$)

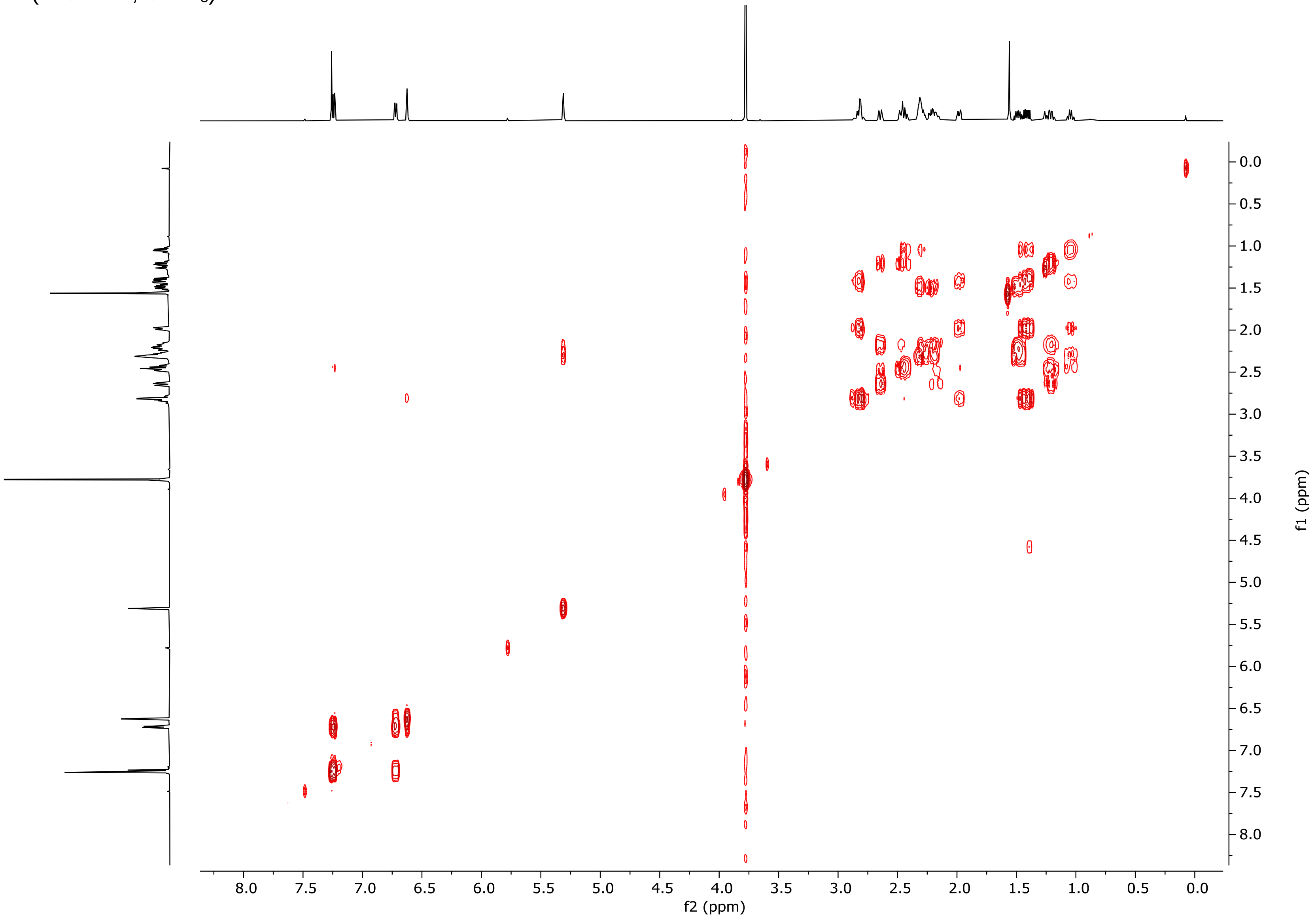

^{1}**H** (600 MHz)-^{13}**C** (151 MHz) **HMBC-2D** ($CDCl_3$)

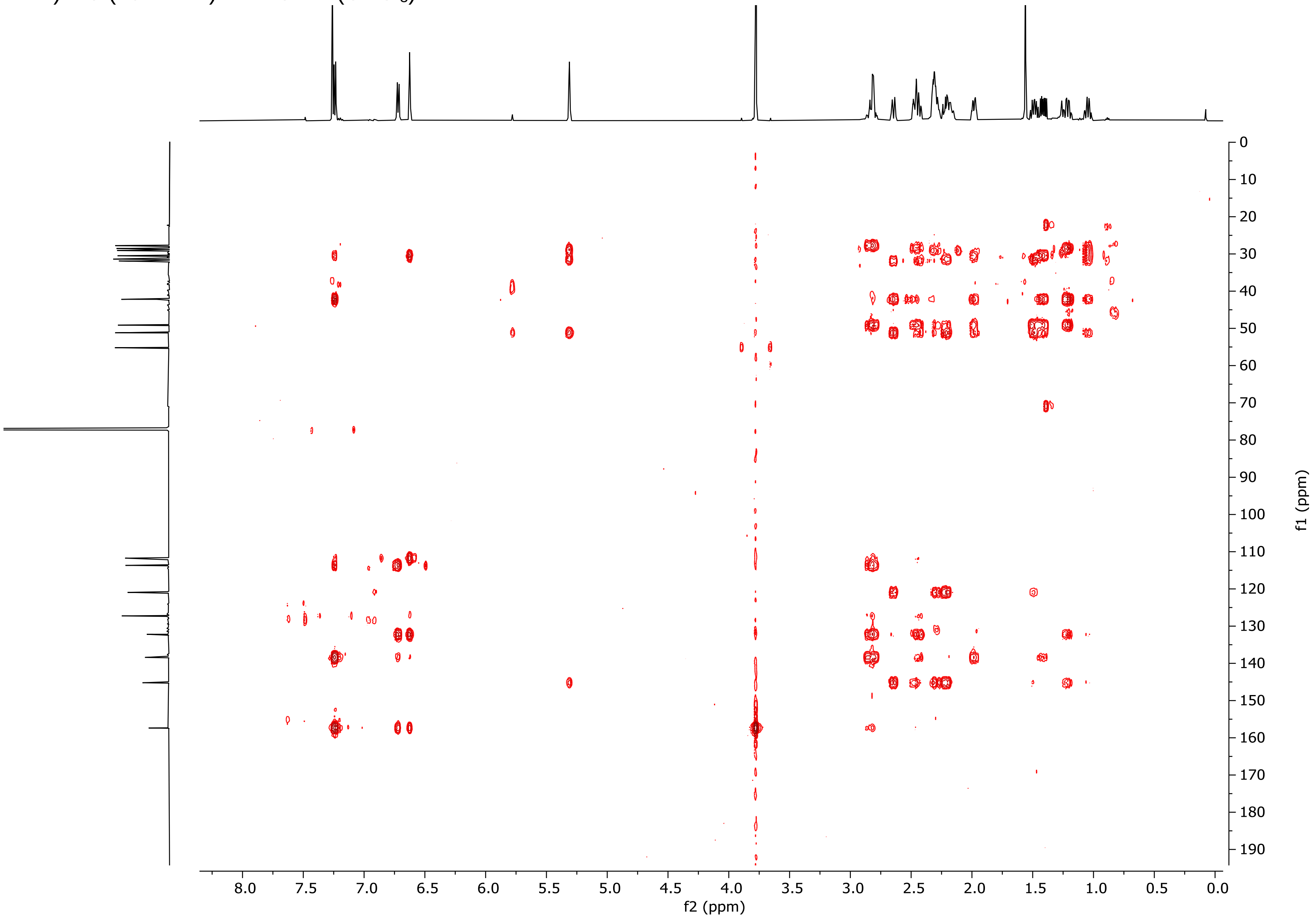

*7.7 Steroid analogue with a C-9 methyl group **S23** and aldehyde **S24**.*

(±)-(8*S*,9*S*,14*S*)-9-methyl-7,8,9,11,12,14,15,16-octahydro-6H-cyclopenta[*a*]phenanthrene S23.

^{1}H NMR (600 MHz, $CDCl_3$)

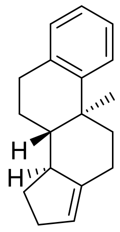

S23

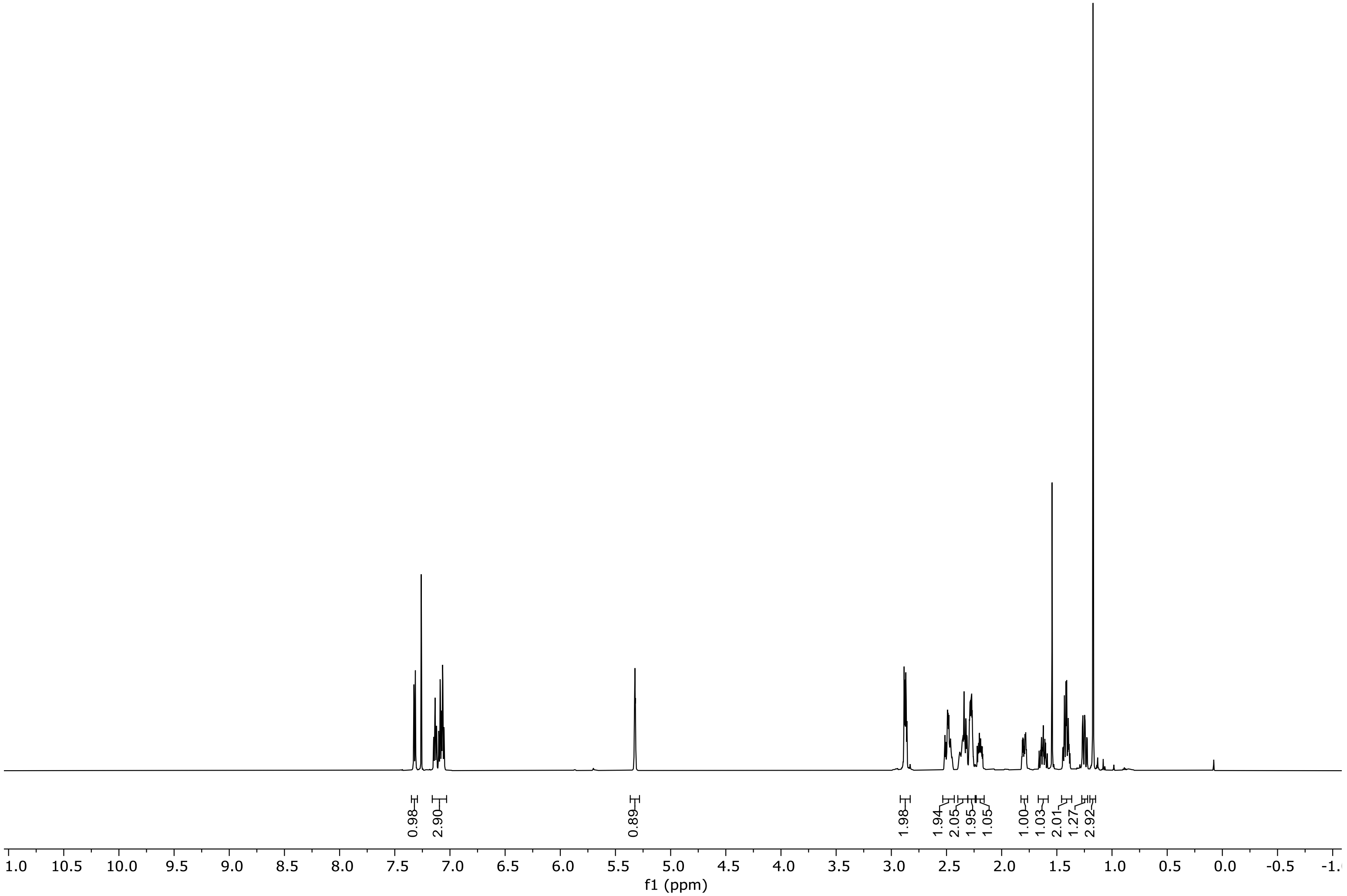

^{13}C NMR (101 MHz, $CDCl_3$)

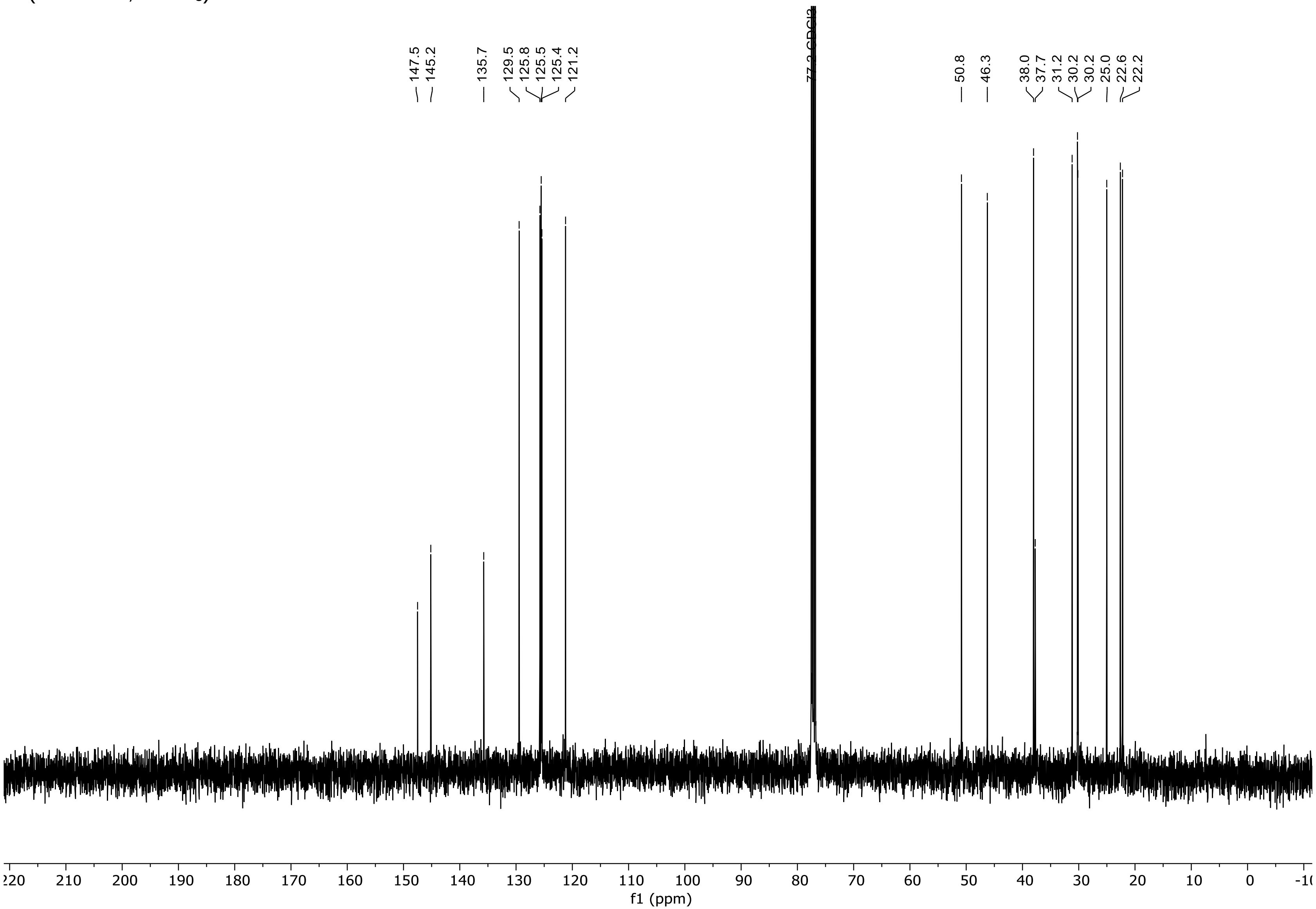

NOESY-2D (600 MHz, $CDCl_3$)

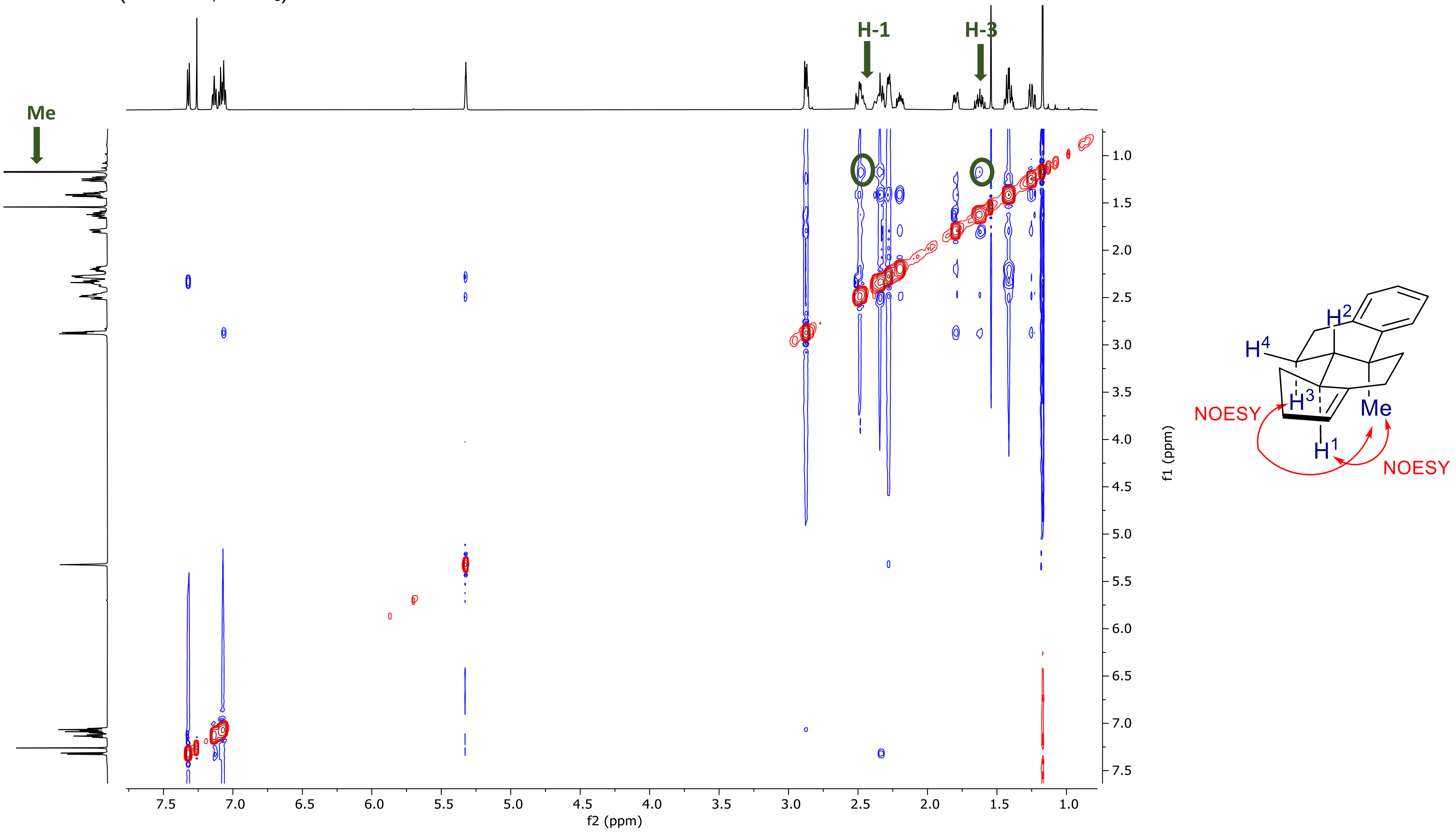

(±)-2-((1*R*,2*R*,4*aS*,10*aS*)-1-(4-methoxyphenyl)-4a-methyl-1,2,3,4,4*a*,9,10,10*a*-octahydrophenanthren-2-yl)acetaldehyde S24.

^{1}H NMR (400 MHz, $CDCl_3$)

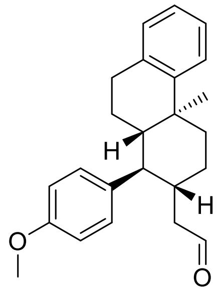

S24

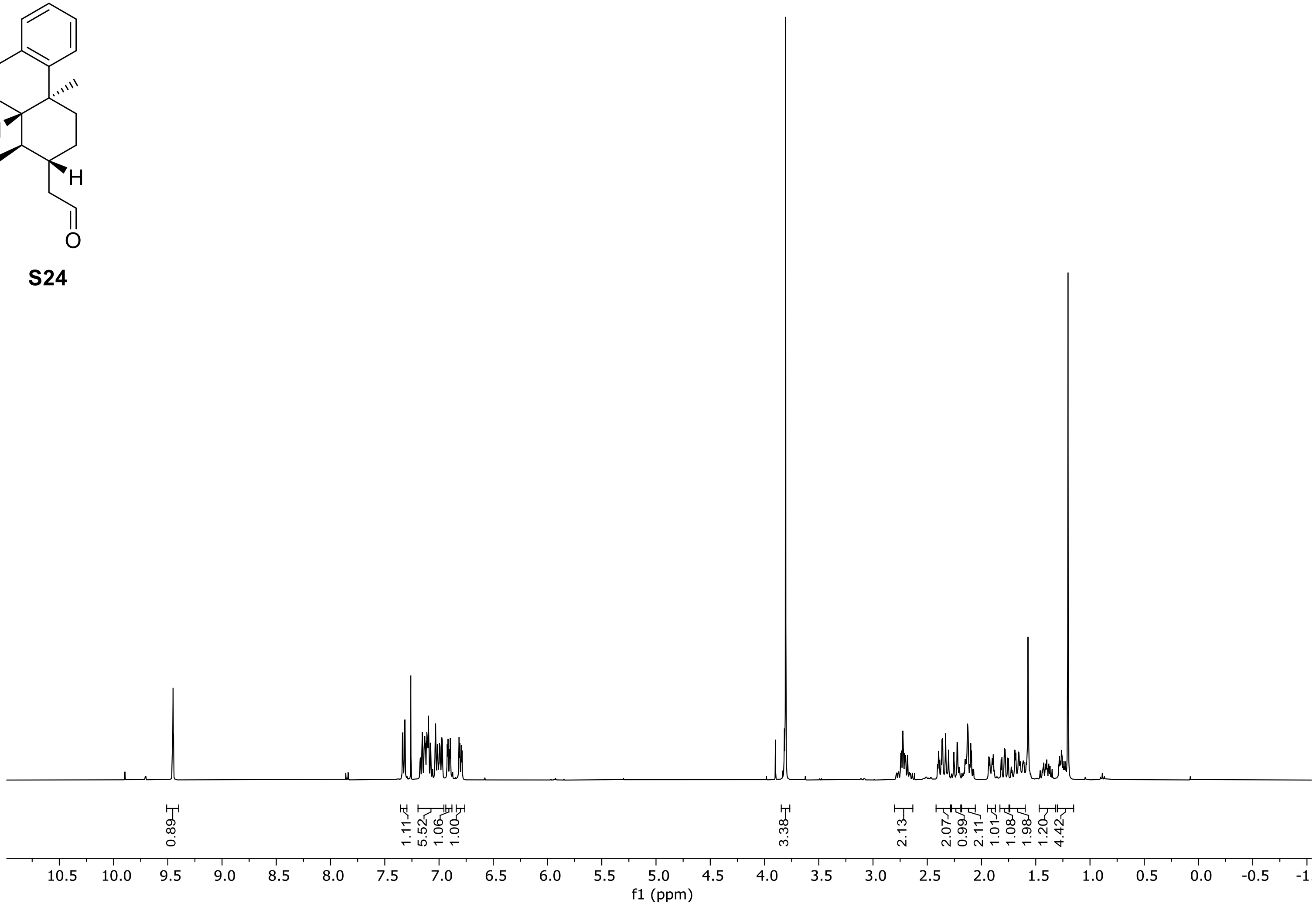

^{13}C NMR (101 MHz, $CDCl_3$)

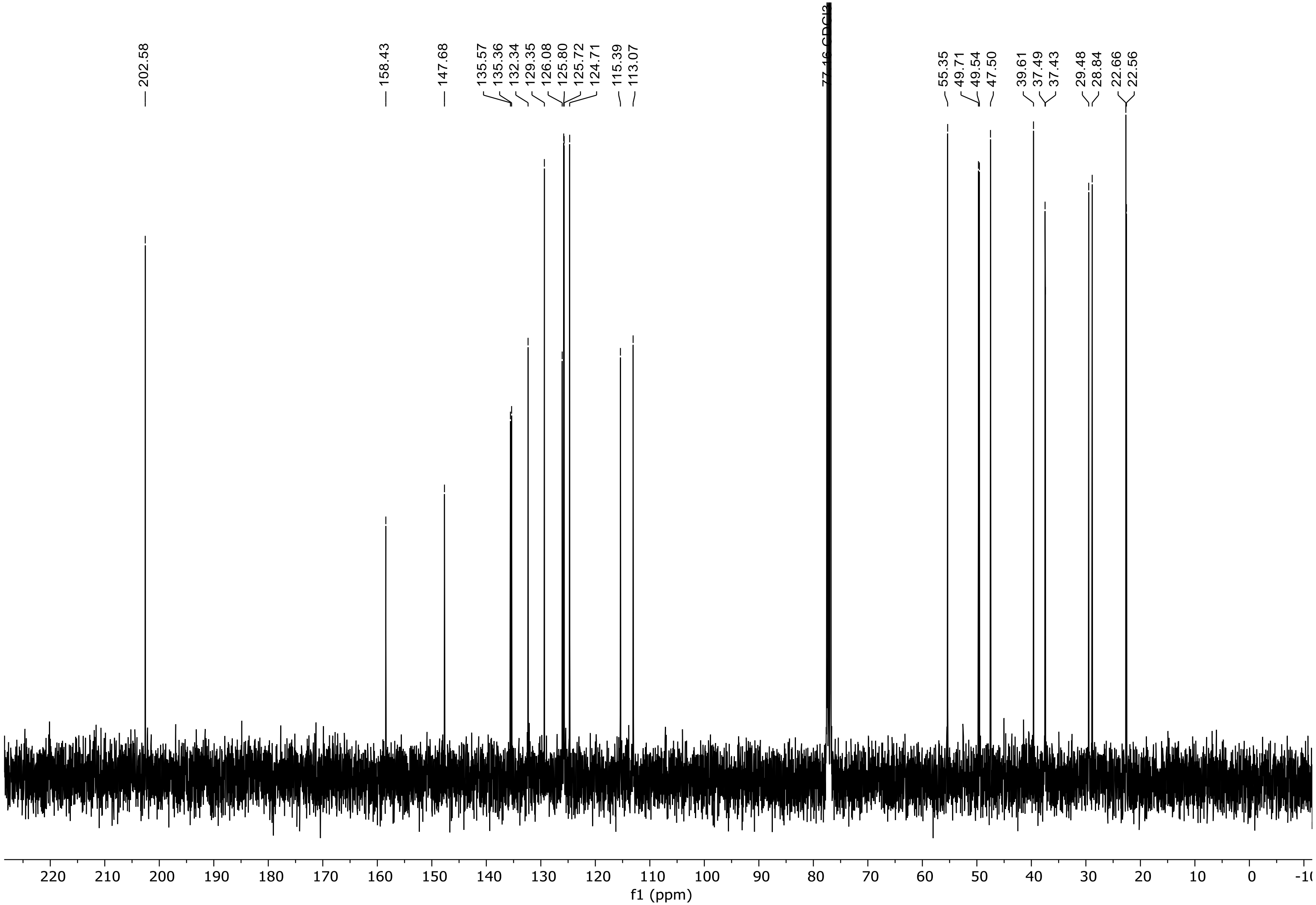

*7.6 Reaction of E/Z-**1** with benzhydrol.*

Crude obtained following the reaction of *E*/*Z*-1 (31:69 *E*:*Z*) with benzhydrol to obtain product 2m.

^{1}H NMR (400 MHz, $CDCl_3$, **crude**)

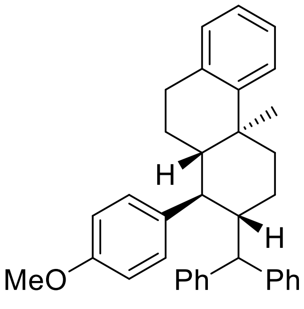

Crude **2m**

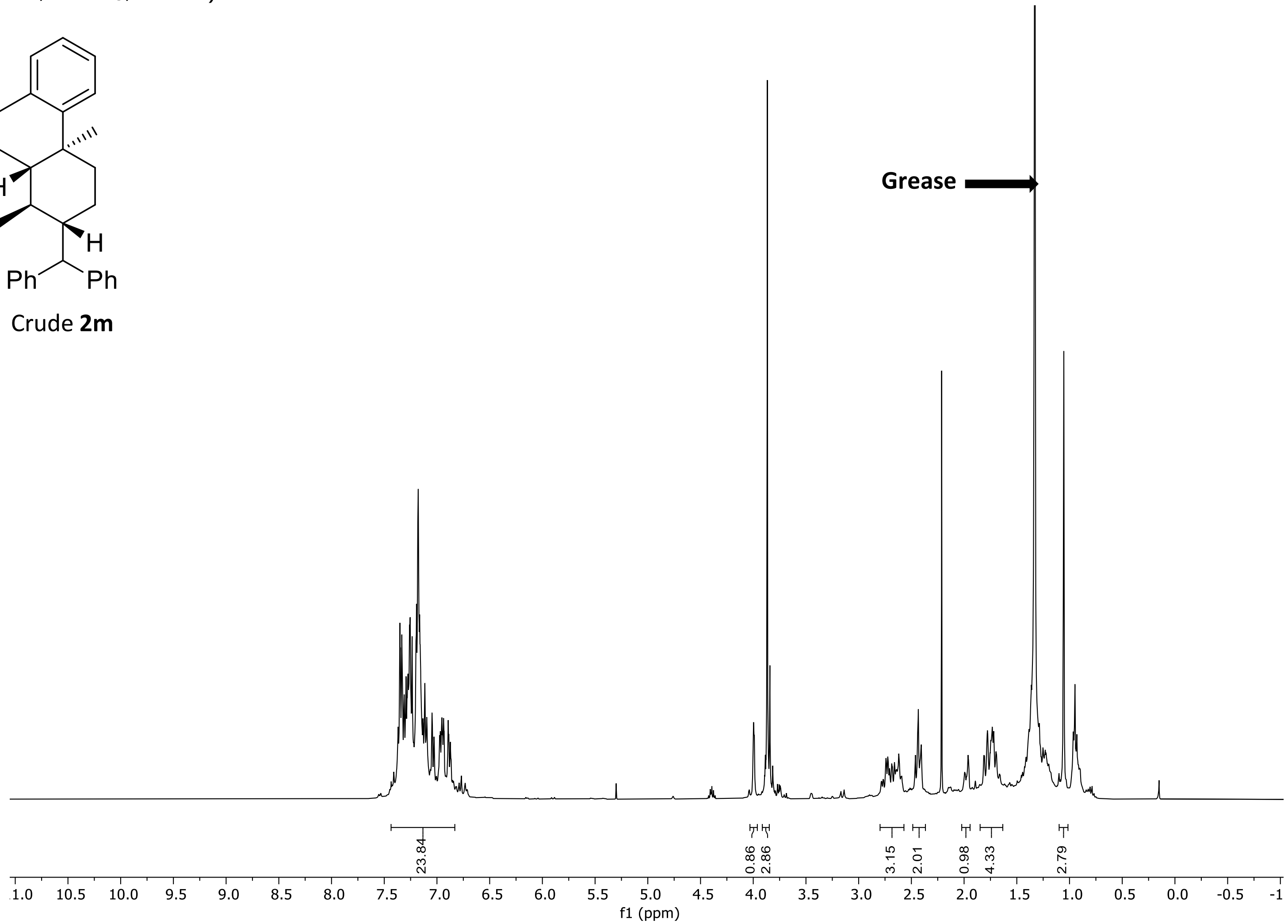

^{13}C NMR (101 MHz, $CDCl_3$, **crude**)

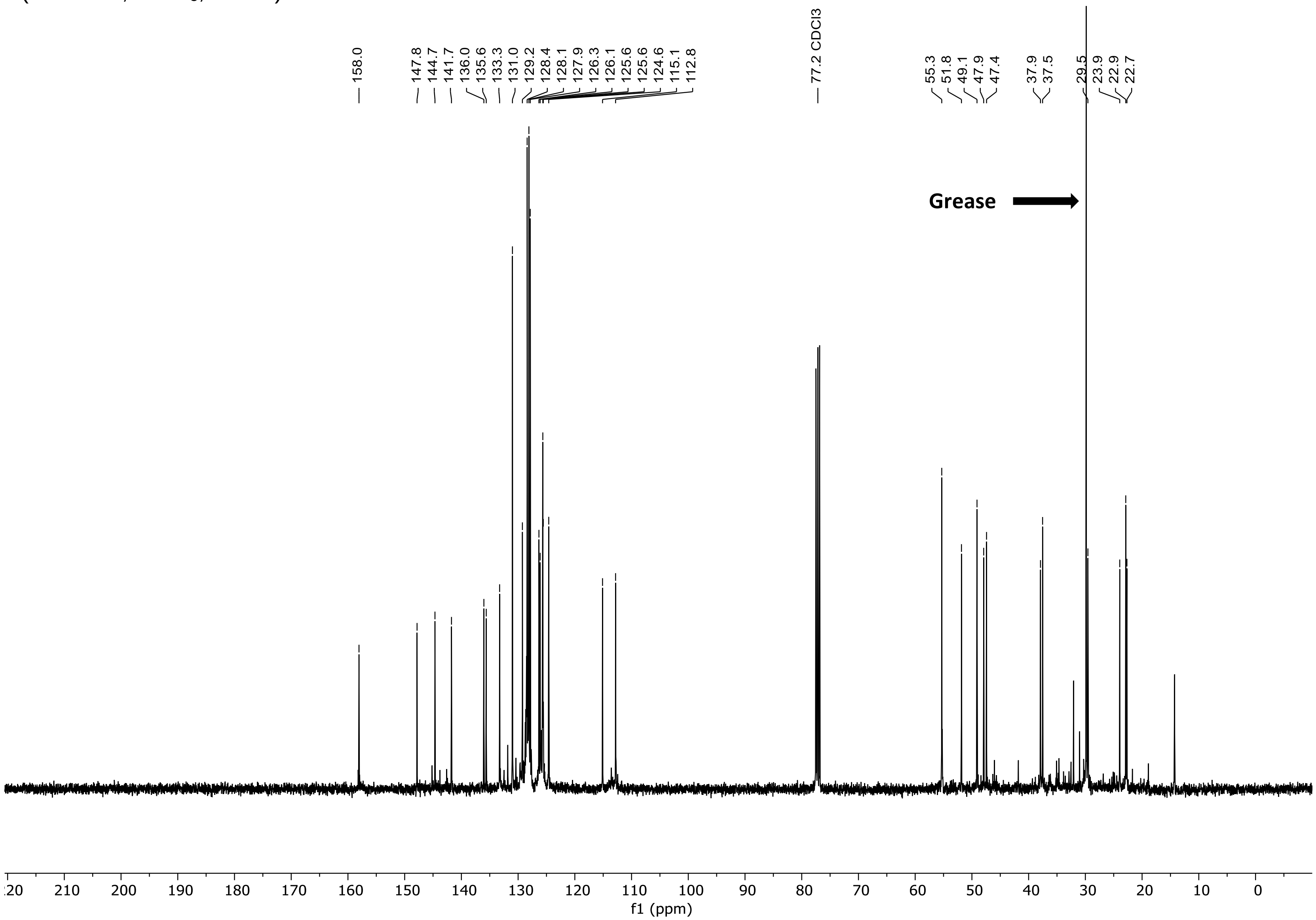

13C NMR comparison between the **crude** product **2m** from the reaction of *E/Z*-**1** (31:69 *E*:*Z*) with benzhydrol with the isolated product **2m** from the reaction of *E*-**1** (>95:5 E:Z) with benzhydrol.

^{13}C NMR (101 MHz, $CDCl_3$)

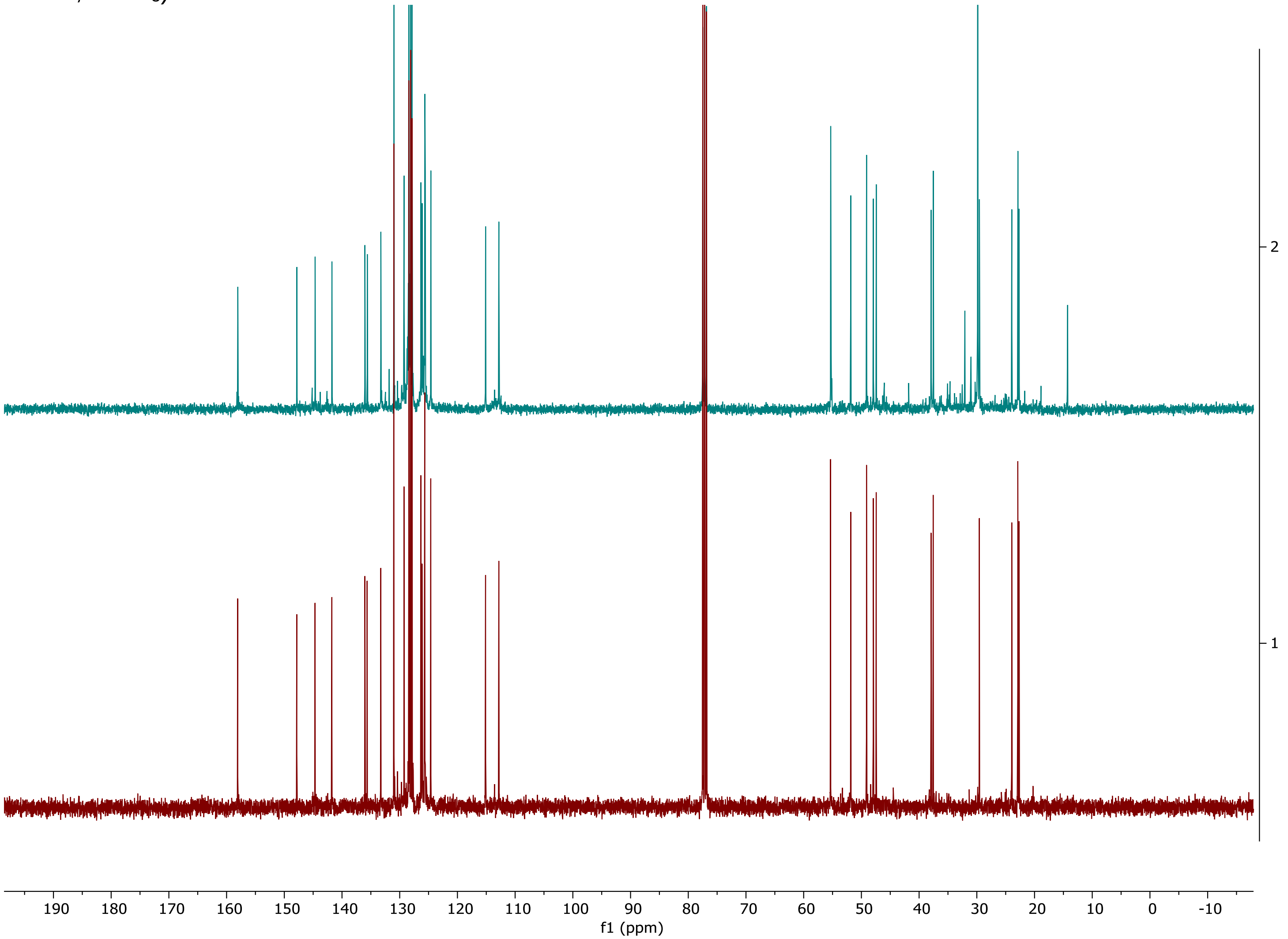

Supplement: Supplementary file 1 — Supporting Information [file CHEM-29-0-s001.pdf]
